# *N*‑Aryl‑*N*‑Lactosylamides as Potent and Highly Selective Inhibitors of Galectin‑3 with Antifibrotic Activity

**DOI:** 10.1021/acs.jmedchem.5c02604

**Published:** 2025-11-11

**Authors:** Jakub Zýka, Jaroslav Kozák, Lenka Vanekova, Marketa Pimkova Polidarova, Vít Prouza, Nina Habanová, Timotej Strmeň, Martin Zavřel, Petr Pachl, Jan Choutka, Klara Grantz Saskova, Andrea Brazdova, Kamil Parkan, Radek Pohl

**Affiliations:** † Gilead Sciences & IOCB Research Centre, 89220 Institute of Organic Chemistry and Biochemistry of the Czech Academy of Sciences, Flemingovo náměstí 2, 166 10 Prague, Czech Republic; ‡ Department of Chemistry of Natural Compounds, 52735 University of Chemistry and Technology Prague, Technická 5, 166 28 Prague, Czech Republic; § Department of Genetics and Microbiology, Faculty of Science, BIOCEV, 37740 Charles University, Průmyslová 595, 252 50 Vestec, Czech Republic; ∥ Department of Public Health and Clinical Medicine, 8075 Umeå University, 901 87 Umeå, Sweden

## Abstract

Galectin-3 (Gal-3) is a galactose-binding lectin involved in pathologies such as inflammation, fibrosis, heart disease, and tumor progression. Here, we report *N*-aryl-*N*-(thio)­lactosylamides as a novel class of Gal-3 inhibitors. A structure–activity study identified 6-carboxyindol-4-yl amide as a key pharmacophoric motif within this series. The most potent inhibitor based on this motif, compound **11**, binds to Gal-3 with excellent affinity (*K*_d_ = 5.7 nM) and selectivity (390-fold over Gal-1). Further in vitro characterization of this compound demonstrated high metabolic stability and no cytotoxicity (CC_50_ > 300 μM). Compound **11** effectively engages Gal-3 with greater activity in macrophage-like than monocyte-like THP1 cells, without affecting inflammation via LPS-induced release of TNFα. In TGFβ-stimulated LX2 hepatic stellate cells, it downregulates profibrotic signaling as assessed by the reduced expression of *ACTA2*, *COL1A2*, and *FN1*. These findings implicate compound **11** as a promising candidate for further preclinical development in the context of fibrotic disease.

## Graphical abstract

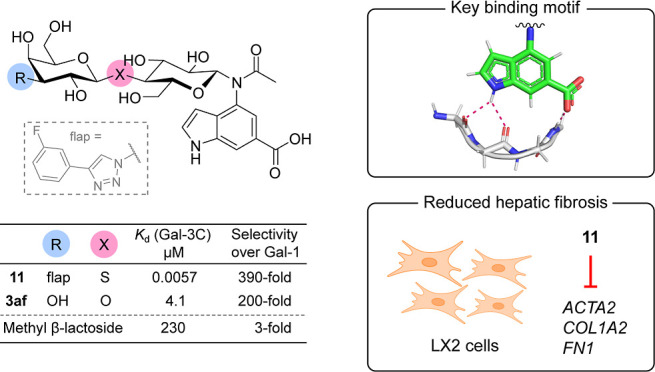

## Introduction

Galectins are a family of lectins defined by the presence of a conserved carbohydrate recognition domain (CRD) that specifically binds β-d-galactosides.^1,2^ Overall, 15 types of galectins have been identified in mammals, of which 12 are expressed in humans. The biological roles of galectins are multifaceted, involving both intra- and extracellular functions. Yet, the most typical galectin-mediated processes are often related to cell–cell or cell–matrix adhesion and signal transduction.

Among all the galectins, galectin-3 (Gal-3) is one of the most widely expressed and, consequently, one of the most medically relevant. Unlike other members of the galectin family, Gal-3 is a chimeric protein consisting of a C-terminal CRD and an intrinsically disordered N-terminal region.^1,3^ In an unbound state, Gal-3 remains monomeric even at high concentrations; however, binding of oligosaccharides can trigger self-association behavior and formation of Gal-3 oligomers.^4,5^ The N-terminal region gives Gal-3 the ability to aggregate via liquid–liquid phase separation (type-N self-association).^3,6,7^ Moreover, Gal-3 also appears to be capable of oligomerization through its C-terminal CRD domain (type-C self-association), analogous to the homodimerization of prototype galectins.^8,9^

Gal-3 is a multifunctional regulator of immunity, inflammation, fibrosis, and tumor progression. Its functional versatility is largely dictated by its subcellular localization. Extracellular Gal-3 mediates cell–cell adhesion, affects immune synapse and cytokine secretion, while intracellular Gal-3 regulates key processes such as apoptosis, signal transduction, and gene transcription through interactions with cytoplasmic and nuclear proteins.^10,11^ In the immune system, Gal-3 promotes monocyte-to-macrophage differentiation, modulates toll-like receptor signaling, and facilitates immune cell recruitment and crosstalk. These activities place Gal-3 at the intersection of both acute and chronic inflammation. In fibrotic disorders, it enhances transforming growth factor beta (TGFβ) signaling, contributing to myofibroblast activation and extracellular matrix remodeling.^12,13^

In cancer, Gal-3 is frequently overexpressed and contributes to multiple hallmarks of malignancy.^14^ It fosters immune evasion by inhibiting T cell activation and promoting regulatory T cell function, while also enhancing metastasis through tumor–endothelium adhesion and extracellular matrix interactions. Elevated Gal-3 expression has been associated with poor prognosis in several cancers, including nonsmall-cell lung cancer, melanoma, and colorectal carcinoma.^15,16^ These compartment-specific and context-dependent roles make Gal-3 an attractive, yet mechanistically complex, therapeutic target.

Over the past two decades, considerable efforts have focused on developing small-molecule galectin inhibitors. These efforts largely take inspiration from the endogenous ligands known to bind the galectin CRD, of which *N*-acetyllactosamine (LacNAc) is the most important (*K*_d_ ∼ 100 μM). The galactose unit from LacNAc binds subsite C of the galectin CRD, while the *N*-acetylglucosamine unit engages the neighboring subsite D.^17,18^ Analogous binding has also been observed for some other disaccharides such as lactose and thiodigalactoside (TDG). Thus, the canonical disaccharide binding motif is also a logical starting point for the design of synthetic inhibitors. Major advancements have been made by Nilsson and co-workers, who discovered that the introduction of aromatic flaps on one or both disaccharide units dramatically enhances affinity. This discovery was used to develop potent 3,3′-disubstituted TDG derivatives bearing coumarylether,^19^ arylester,^20^ arylamide,^21,22^ amidotriazole,^23^ and aryltriazole^24^ flaps. This line of research culminated in the development of the **GB0139** compound,^24^ which inhibits Gal-3C with *K*_d_ in the low nanomolar range (Figure 1A). This compound was evaluated by Galecto Biotech in an unsuccessful phase 2b clinical trial (NCT03832946) for idiopathic pulmonary fibrosis. However, it recently showed promise in a phase 1b/2a clinical trial (NCT04473053)^25^ for COVID-19 pneumonitis.

**Figure 1:**
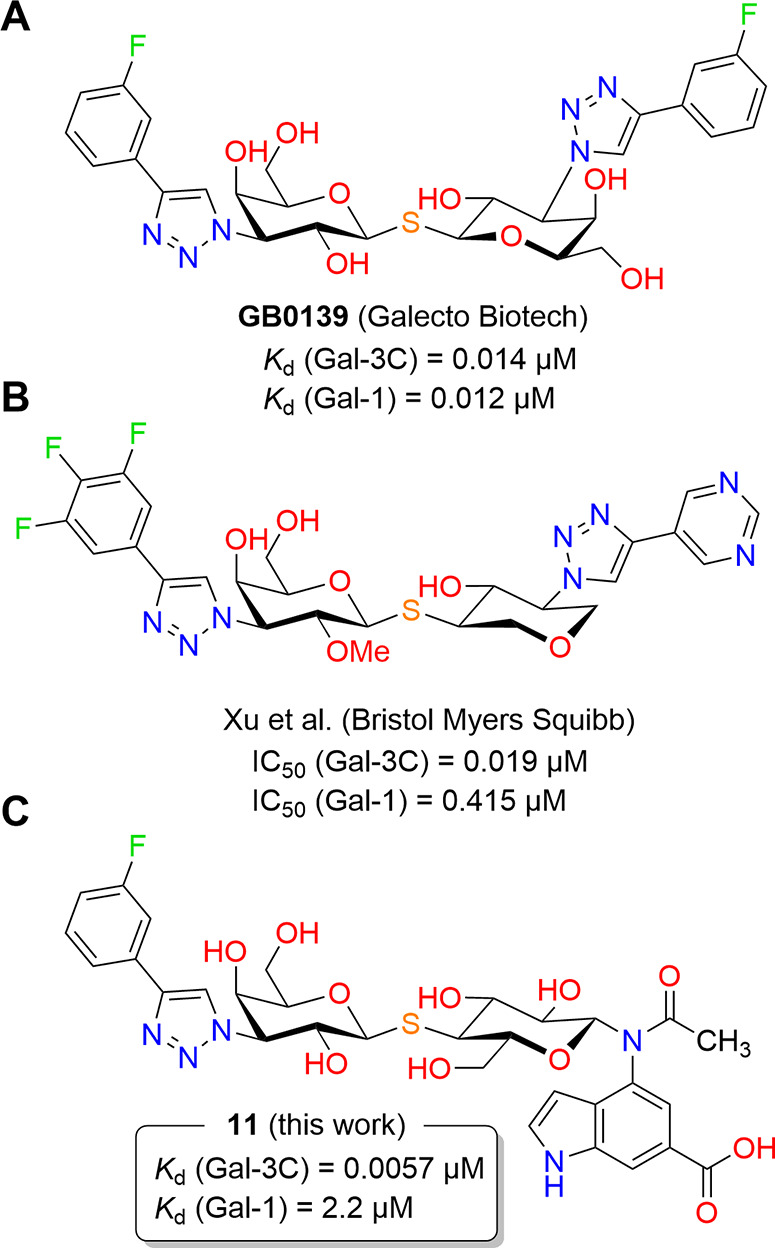
Disaccharide-based galectin inhibitors. Previously reported inhibitors are shown in (A, B). The newly developed lead compound reported here is shown in (C).

Another disaccharide-based inhibitor was reported by Xu et al.^26^ Their work is based on a scaffold similar to that of **GB0139** but with an overhaul aiming at lower polarity. To achieve this, all nonessential hydroxyl groups were either removed or methylated, leading to preserved affinity as well as improved drug-likeness (Figure 1B).

In the present study, we report the discovery of a novel class of disaccharide-based inhibitors of Gal-3. Our approach focused on modifying the lactose anomeric position with aromatic amines, yielding a series of *N*-aryl-*N*-lactosylamides. The exploration of the structure–activity relationship in this series led to the identification of the 6-carboxyindol-4-yl acetamide moiety as a highly potent and selective binding motif. Based on these results, we synthesized a fully decorated thiolactose-based inhibitor that achieved single-digit nanomolar *K*_d_ values for Gal-3. This affinity rivals the most potent inhibitors reported to date, while displaying unprecedented selectivity for Gal-3 (Figure 1C). Beyond affinity and selectivity profiling, we demonstrate the potential of this newly developed inhibitor class by delineating intracellular engagement of Gal-3 along with its immunomodulatory profile and antifibrotic activity in cell-based assays.

## Results and Discussion

### Synthesis of *N*-Aryl-*N*-Lactosylamides

The general approach to the synthesis of *N*-aryl-*N*-lactosylamides utilized in this study is outlined in Scheme 1, and builds on our previously described procedure.^27^ Starting from selectively protected hepta-*O*-acetyl lactose **1**,^28^ the protected *N*-aryl-*N*-lactosylamides **2a–af** were obtained via reaction of **1** with aromatic amines, followed by *N*-acylation. As the intermediate hemiaminal formed in the first reaction step proved unreactive under commonly used acylation conditions (e.g., Ac_2_O in pyridine or AcCl with Et_3_N), a Lewis acid-catalyzed acylation in nitromethane was employed. Although a similar strategy can also be applied using free lactose instead of acetate **1**, this alternative offered no significant advantage and actually increased the number of synthetic steps required for each target compound. Finally, the acetates **2a–ae** were deprotected under alkaline conditions, yielding *N*-aryl-*N*-lactosylamides **3a–af**. In several cases, the deprotection conditions had to be carefully optimized due to the competing formation of the *N*-deacylated byproduct. This undesired side reaction was particularly prominent in derivatives bearing multiple halogen substituents, and even careful pH adjustment and lowering of the reaction temperature resulted in partial cleavage of the amide bond.

**Scheme 1:**
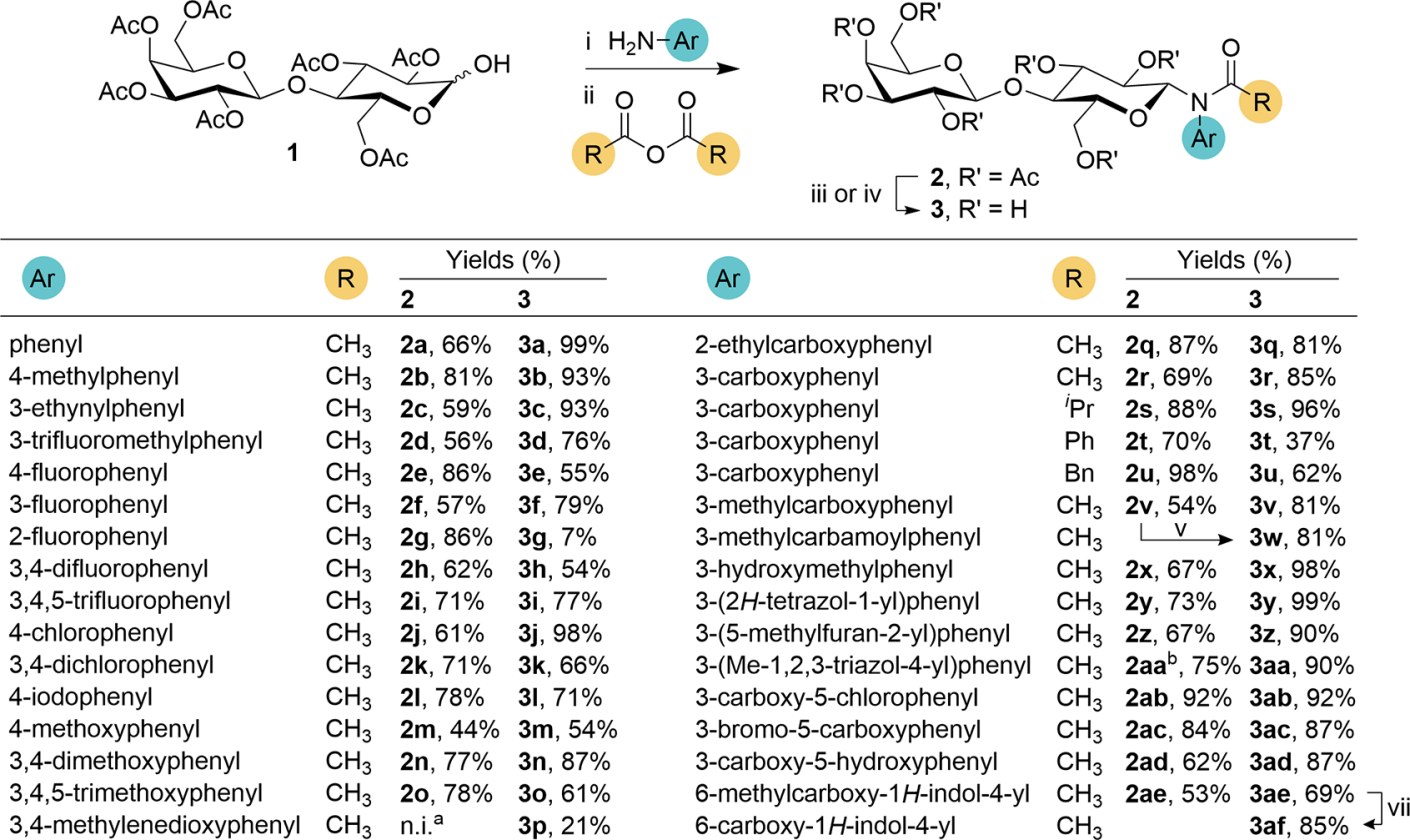
Synthesis of *N*-Aryl-*N*-Lactosylamides **3a–af** ^c^

a Not isolated.
b Obtained from **2c** via (vi).
c Reagents and conditions: (i) aromatic amine, CH_3_OH, 67 °C, 72–168 h; (ii) acyl anhydride, ZnCl_2_, CH_3_NO_2_, rt to 40 °C, 30 min; (iii) 7 N NH_3_ in CH_3_OH, rt, 3–72 h; (iv) cat. CH_3_ONa in CH_3_OH, rt, 30 min to 3 h; (v) CH_3_NH_2_ in EtOH, rt, 72 h; (vi) CH_3_I, NaN_3_ in DMF, rt, 1 h, followed by **2c**, CuI, DIPEA, 2 h; (vii) LiOH, dioxane/H_2_O 1:1, rt, 2 h.

In select cases, target compounds were not obtained directly from **1** via *N*-glycosylation but rather through the derivatization of intermediate *N*-aryl-*N*-lactosylamides. Specifically, the triazole derivative **2aa** was synthesized by a copper­(I)-catalyzed click reaction between **2c** and azidomethane; the amide **3w** was obtained by ammonolysis of **2v** with excess of methylamine; and the indole carboxylic acid **3af** was prepared by the hydrolysis of its methyl ester **3ae**.

*N*-Aryl-*N*-lactosylamides **2** and **3** exist in solution as an equilibrium mixture of rotamers originating from the hindered rotation of the N–CO bond in tertiary amides. This conformational behavior manifests in nuclear magnetic resonance (NMR) spectra measured at room temperature by a substantial line-broadening or the presence of an additional set of signals caused by the chemical exchange between the rotamers.^29–31^ This effect is demonstrated in the Supporting Information in Figure S6, where a comparison of 1D NMR spectra at room temperature and 100 °C is shown.

### Initial Structure–Activity Screening

Our investigation of the structure–activity relationship of *N*-aryl-*N*-lactosylamides started with an initial screening of substituted acetanilides (Table 1). These compounds can be readily synthesized from a wide range of commercially available substituted anilines. Affinity was evaluated using a fluorescence polarization assay, which enables reliable determination of dissociation constants (*K*_d_) between small molecules and galectins.^32^ All inhibitors in this series were tested against the two most common human galectins: Gal-1 and Gal-3. Gal-1 is a prototype galectin consisting of a single CRD and was used in full length. In the case of Gal-3, which consists of the C-terminal CRD and N-terminal unfolded region, only the C-terminal domain was used (Gal-3C). Affinities were compared to methyl β-lactoside (β-LacOMe) as a baseline.

**Table 1:**
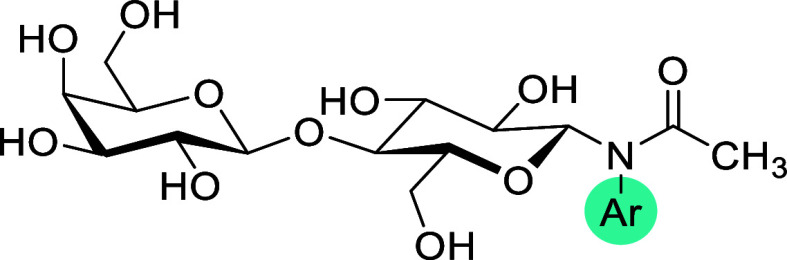

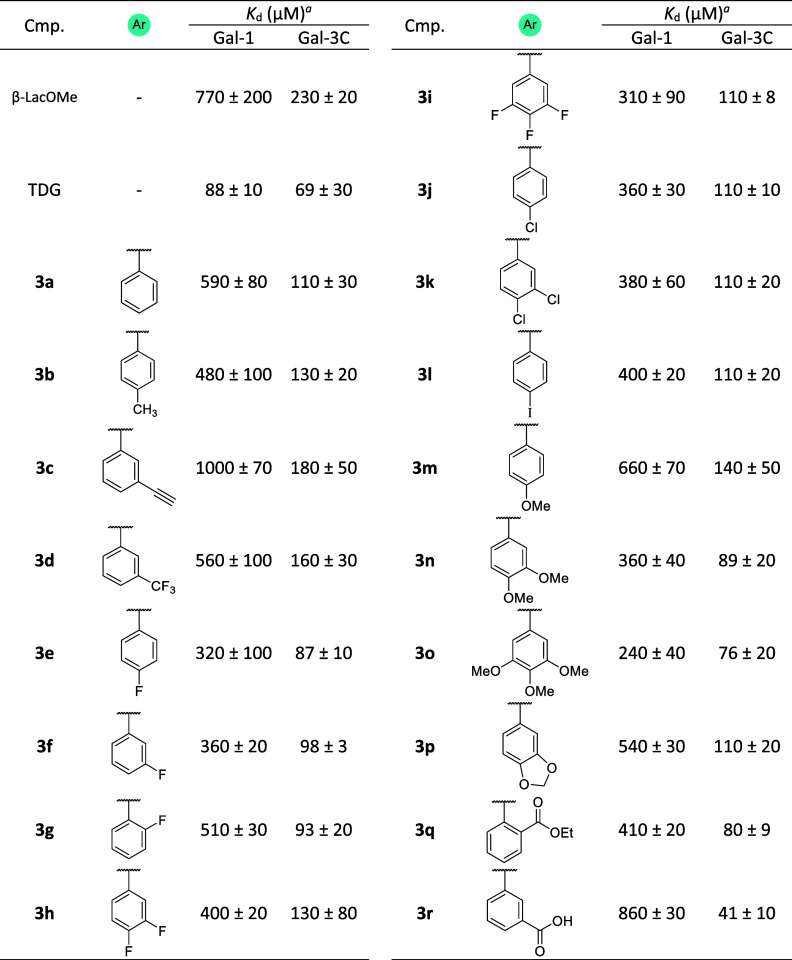
Affinities of Compounds **3a–r** for Human Galectin-1 and -3C Determined by Competitive Fluorescence Polarization

a *K*_d_ values are reported as the mean ± SD from two independent experiments, each performed in triplicate.

The introduction of an unsubstituted phenyl moiety in the form of acetamide **3a** led to an approximately 2-fold improvement in affinity toward both Gal-1 and Gal-3C compared to β-LacOMe. For the most part, substitution of the phenyl ring with various electron-donating and electron-withdrawing groups in compounds **3b–3r** did not result in major affinity changes compared to **3a**. However, there were two notable exceptions: 3,4,5-trimethoxyphenyl amide **3o** exhibited a 2-fold improvement in affinity toward Gal-1, and 3-carboxyphenyl amide **3r** provided a 2-fold improvement toward Gal-3C. Based on this initial screening, compound **3r** was chosen as the most promising early hit, showing the greatest improvement in affinity toward Gal-3C (*K*_d_ = 41 μM) along with reduced affinity toward Gal-1 (*K*_d_ = 860 μM), thereby achieving a promising 20-fold selectivity in favor of Gal-3C.

The X-ray structure of **3r** in complex with human Gal-3C was solved at 1.06 Å resolution (PDB 9S62) and is shown in Figure 2. Analysis of the binding mode revealed that the lactose scaffold binds in the canonical position in subsites C and D as expected, while the 3-carboxyphenyl moiety resides in a receptor region defined by the backbone of residues Trp-181, Gly-182, Arg-183, and Glu-184. A hydrogen bond between the carboxylate of **3r** and the backbone NH of Glu-184 can clearly be detected. Additionally, a crystallographic water molecule appears to mediate a network of hydrogen bonds involving the carboxylate and OH-2′ groups of **3r** together with the side-chain carboxylate of Glu-184, potentially further stabilizing the binding of **3r**.

**Figure 2:**
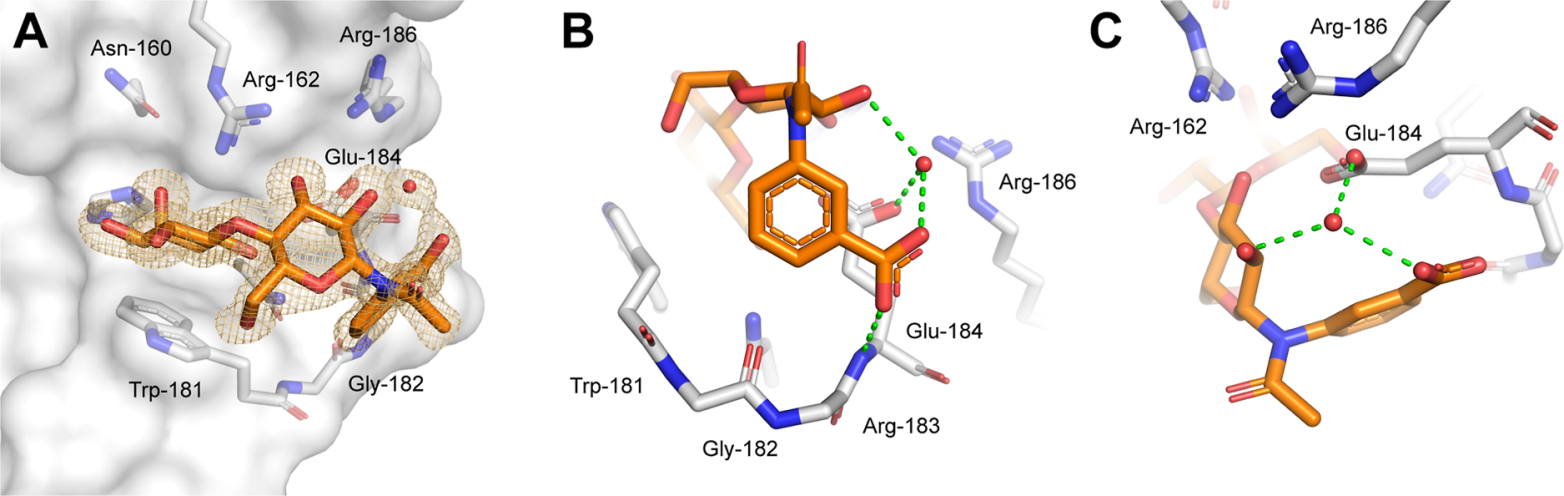
X-ray structure of **3r** in complex with Gal-3C (PDB 9S62). (A) Overall view of **3r** in the binding site. The 2*F*_o_–*F*_c_ density map of the ligand is shown at isovalue 1σ. (B) Detailed view of the interaction between the *m*-carboxyphenyl moiety of **3r** and the receptor backbone. (C) Crystallographic water bridging the carboxylate and OH-2′ groups of **3r** with the side-chain carboxylate of Glu-184.

### Hit Optimization

After characterizing the binding mode of the initial hit, we focused on optimizing the structure of **3r** to increase its potency. We first attempted to modify the *N*-acetyl substituent in **3r** (Table 2) to introduce bulkier or more hydrophobic acyl moieties. Specifically, we replaced the acetyl group in **3r** with isobutanoyl, benzoyl, and phenylacetyl groups, generating compounds **3s**, **3t**, and **3u**, respectively. However, none of these modifications led to improved affinity, suggesting that the acyl group projects away from the binding pocket and does not engage in favorable interactions with the receptor. Therefore, further optimization of the *N*-acyl substituent was not pursued.

**Table 2:**
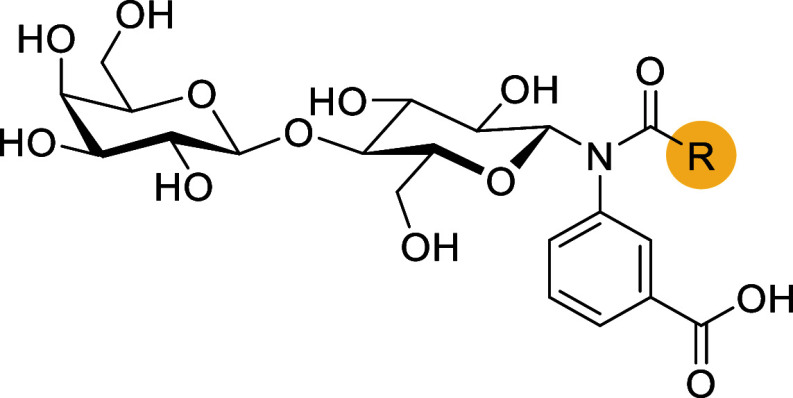

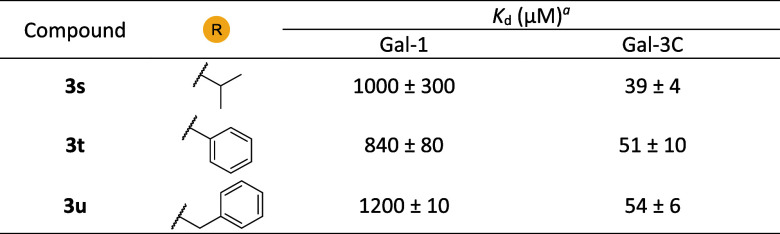
Affinities of Compounds **3s–u** for Human Galectin-1 and -3C Determined by Competitive Fluorescence Polarization

a *K*_d_ values are reported as the mean ± SD from two independent experiments, each performed in triplicate.

Next, we turned our attention to the *m*-carboxyphenyl motif of **3r**, with the aim of either replacing the carboxylate with an isosteric group or retaining the carboxylate and adding further groups to the phenyl ring that might enhance potency. The results of this investigation are summarized in Table 3. We first attempted to modify the carboxylate to methyl ester **3v** and methylamide **3w**. These derivatives exhibited a modestly increased potency toward Gal-1 but diminished affinity toward Gal-3C, resulting in eroded selectivity. The reduction of the carboxylate group yielded hydroxymethyl derivative **3x**, which led to a 2-fold decrease of affinity toward Gal-3C compared to **3r**. Next, we explored bioisosteric replacement of the carboxylate group with five-membered heterocycles, resulting in tetrazole **3y**, methylfuran **3z**, and methyltriazole **3aa**. Among these, tetrazole **3y** was able to match the affinity of **3r** toward Gal-3C, while **3z** and **3aa** again both showed a reduction in potency. These results demonstrate that a negatively charged carboxylate or tetrazole group at the meta position is crucial for increased affinity toward Gal-3C, whereas attempts to replace it with a neutral group revert the potency back to the level of the unsubstituted phenyl amide **3a**. In contrast, the presence of a charged group appears detrimental for Gal-1 binding, underscoring its potential for achieving selectivity for Gal-3C over Gal-1.

**Table 3:**
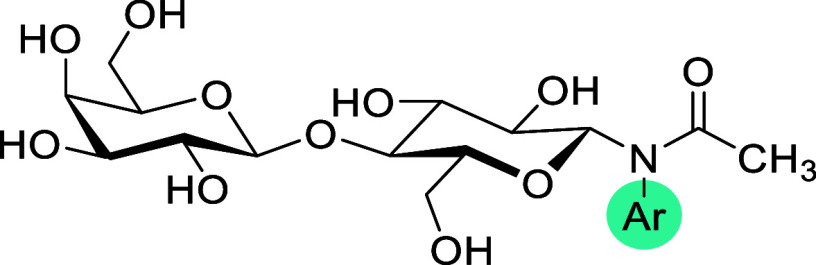

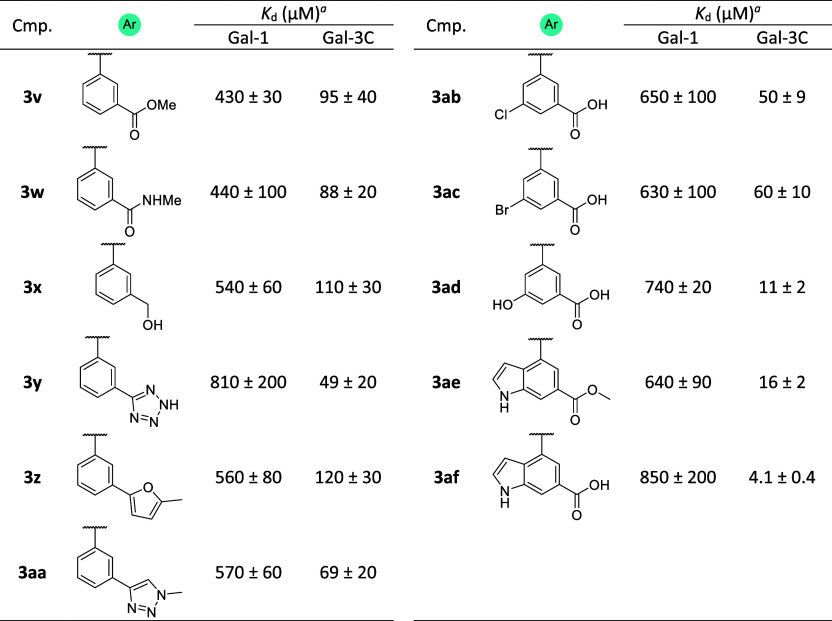
Affinities of Compounds **3v–af** for Human Galectin-1 and -3C Determined by Competitive Fluorescence Polarization

a *K*_d_ values are reported as the mean ± SD from two independent experiments, each performed in triplicate.

Based on these findings, we retained the *m*-carboxylate substituent and focused on exploring additional modifications on the aromatic ring. Structural analysis of the Gal-3C binding site revealed that the second meta position is favorably oriented toward the backbone of Trp-181 and Gly-182, making it a promising hotspot for modification. Disappointingly, the chloro and bromo derivatives **3ab** and **3ac** did not yield any affinity enhancement. In contrast, the introduction of a hydrogen bond donor emerged as a key potency-increasing modification. Specifically, the *m*-carboxy-*m*-hydroxyphenyl derivative **3ad** achieved an affinity of 11 μM toward Gal-3C, while the 6-carboxyindole derivative **3af** further improved affinity to 4.1 μM. As observed previously, conversion of **3af** to its methyl ester **3ae** resulted in a notable loss of potency, reinforcing the importance of the free carboxylate group.

Overall, the affinity of **3af** for Gal-3C was enhanced 10-fold compared to **3r**, 27-fold compared to the unsubstituted phenyl derivative **3a**, and 56-fold compared to β-LacOMe. Notably, this improvement in affinity toward Gal-3C was not accompanied by a corresponding improvement toward Gal-1. Instead, the affinity toward Gal-1 remained practically unchanged compared to β-LacOMe. As a result, the optimized hit **3af** displays more than 200-fold selectivity for Gal-3C over Gal-1, representing a significant advance in the development of selective Gal-3 inhibitors. The competitive fluorescence polarization titration curves for all *N*-aryl-*N*-lactosylamides **3a–3af**, along with their corresponding selectivity profiles, are shown in Figure 3.

**Figure 3:**
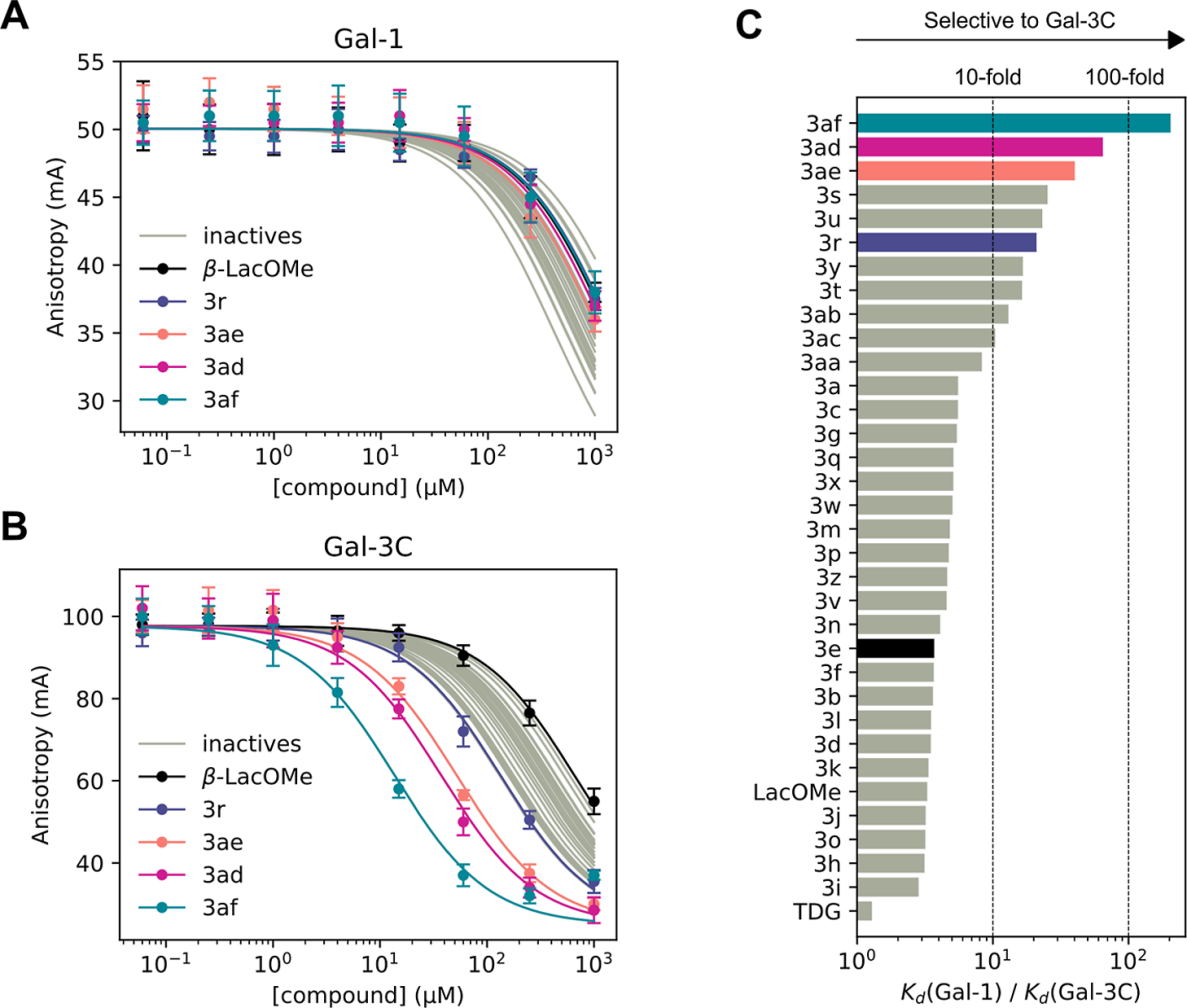
Binding curves of compounds **3a–af** with (A) Gal-1 and (B) Gal-3C obtained by competitive fluorescence polarization. Data are plotted as means with error bars representing SD for each concentration point. (C) Fold selectivity of compounds **3a–af** calculated as the ratio of *K*_d_ (Gal-1)/*K*_d_ (Gal-3C).

### Synthesis of Fully Decorated Inhibitor **11**

To compare the *N*-aryl-*N*-lactosylamide derivatives with those based on the thiodigalactoside scaffold (such as **GB0139**), we prepared a fully decorated inhibitor based on a thiolactose core bearing a 4-(3-fluorophenyl)-1,2,3-triazol-1-yl substituent on the galactose unit. A reaction of previously described monosaccharide precursors, thiol **4** ^33^ and triflate **5**,^34^ resulted in the formation of protected thiolactose **6**. Deprotection of this intermediate then provided the reducing thiolactose derivative **7**, which served as the starting material for the consequent *N*-glycosylation chemistry (Scheme 2).

**Scheme 2:**
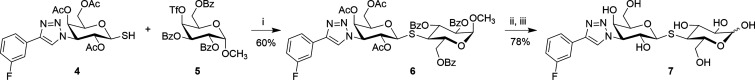
Synthesis of Substituted Thiolactose **7** ^a^

a Reagents and conditions: (i) 1,4-dithiothreitol, Et_3_N, THF, rt, 15 h; (ii) H_2_SO_4_, AcOH, Ac_2_O, 40 °C, 10 min; (iii) cat. CH_3_ONa in CH_3_OH, rt, 18 h.

Heating compound **7** with methyl 4-amino-1*H*-indole-6-carboxylate in methanol at 80 °C in a sealed pressure tube led to the rapid formation of hemiaminal **8**. Elevated temperature was required due to the poor solubility of compound **7** in methanol and other lower alcohols. A two-stage acetylation—first with acetic anhydride in pyridine to acetylate the hydroxyl groups, followed by acetic anhydride and ZnCl_2_ in CH_3_NO_2_—yielded protected intermediate **9**. Final deprotection of hydroxyl groups and hydrolysis of the methyl ester furnished the target indolyl amide **11**. Attempts to introduce the acetyl group directly using excess acetic anhydride with ZnCl_2_ and CH_3_NO_2_ under heating led instead to overacetylation of the starting material, yielding the 3-acetyl-1*H*-indolecarboxylic derivative **10** (Scheme 3).

**Scheme 3:**
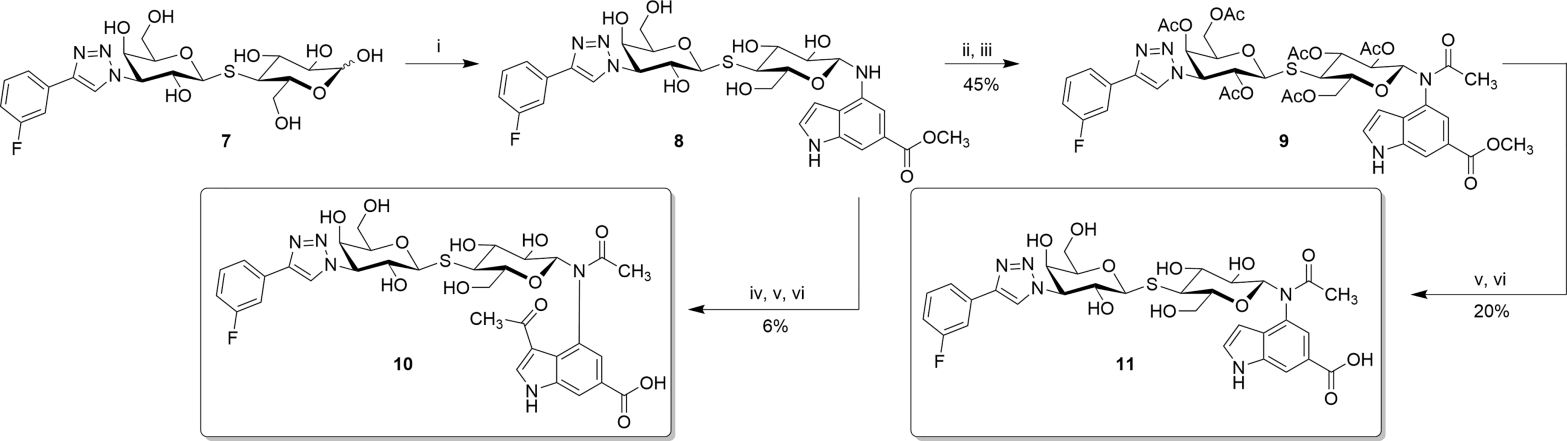
Synthesis of Indolyl Amides **10** and **11** ^a^

a Reagents and conditions: (i) methyl 4-amino-1*H*-indole-6-carboxylate, cat. AcOH, CH_3_OH, 80 °C, sealed tube, 4 h; (ii) Ac_2_O, pyridine, rt, 4 h; (iii) ZnCl_2_, Ac_2_O, CH_3_NO_2_, 35 °C, 3 h; (iv) Ac_2_O, ZnCl_2_, CH_3_NO_2_, 50 °C, 3 h, followed by pyridine, rt, 2 h; (v) cat. CH_3_ONa in CH_3_OH, rt, 18 h; (vi) LiOH, dioxane/H_2_O, rt, 2 h.

### Indolyl Amides **10** and **11** Reach Low-Nanomolar Affinity and High Selectivity toward Galectin-3

As with the *N*-aryl-*N*-lactosylamide series **3a–af**, the affinities of the fully decorated inhibitors **10** and **11** were determined by competitive fluorescence polarization assay. Their potencies were compared with **GB0139**, one of the most potent and widely studied disaccharide-based galectin inhibitors.^24^ As previously, galectin-3 was used only in the form of its C-terminal CRD (denoted as Gal-3C), without the unfolded N-terminal region. The affinities of **10**, **11**, and **GB0139** are summarized in Table 4. Encouragingly, the inhibitors **10** and **11** displayed excellent affinity toward Gal-3C, both in the low-nanomolar range of dissociation constants. The 3-acetylindole derivative **10** inhibited Gal-3C with *K*_d_ = 31 nM, while the indole derivative **11** bound even more potently with *K*_d_ = 5.7 nM. Moreover, both inhibitors exhibited poor affinity for Gal-1 (*K*_d_ = 3600 nM and 2200 nM, respectively), resulting in remarkable selectivity for Gal-3C over Gal-1 (approximately 390-fold in the case of **11**).

**Table 4:** Affinities of Compounds **10**, **11**, and **GB0139** for Human Galectin-1 and -3C Determined by Competitive Fluorescence Polarization

	*K*_d_ (μM)^a^	
compound	Gal-1	Gal-3C
**GB0139**	0.16 ± 0.05	0.0097 ± 0.003
**10**	3.6 ± 0.4	0.031 ± 0.004
**11**	2.2 ± 0.3	0.0057 ± 0.002

a *K*_d_ values are reported as the mean ± SD from three independent experiments, each performed in triplicate.

Comparison of our most potent inhibitor **11** with the previously reported **GB0139** revealed nearly identical affinity toward Gal-3C (*K*_d_ = 9.7 nM for **GB0139** vs *K*_d_ = 5.7 nM for **11**). However, a clear distinction emerged in selectivity: **GB0139** binds Gal-1 with *K*_d_ = 160 nM, whereas compound **11** exhibits much weaker binding with *K*_d_ = 2200 nM. Thus, inhibitor **11** matches the affinity of **GB0139** for Gal-3C while offering a superior selectivity profile, making it an attractive candidate for selective Gal-3 inhibition.

### In Silico Modeling of the Binding Mode

To elucidate the binding of the prepared inhibitors to the Gal-3C, molecular dynamics (MD) simulations were employed. Complexes of Gal-3C with inhibitors **3a**, **3r**, **3af**, and **11** were simulated in explicit solvent in three independent replicas for a total of 3 μs per compound. The binding pose of **3r** from the X-ray structure (see Figure 2) was used as a reference representing a bound conformation of the *N*-aryl-*N*-lactosylamide scaffold in the Gal-3C binding site. Analogous reference poses were also constructed for **3a**, **3af**, and **11** from the *m*-carboxyphenyl ring of **3r**. These X-ray-like poses were used to represent the putative bound conformation.

To compare simulated trajectories with these reference poses, we calculated the root-mean-square deviation (RMSD) of ligand atoms between simulation frames and the reference pose. These RMSD values enabled us to quantify the closeness of the conformations sampled during the MD simulation to the reference pose, where the aryl moiety is considered bound. The representative 30 frames for each complex, colored by RMSD, are shown in Figure 4A,B. Additionally, we performed dihedral principal component analysis (dPCA), which is analogous to conventional PCA but performed in the space of sine- and cosine-transformed dihedral angles (for details, see the Supporting Information). Results of the dPCA, performed on 10,000 representative frames, are depicted in Figure 4C.

**Figure 4:**
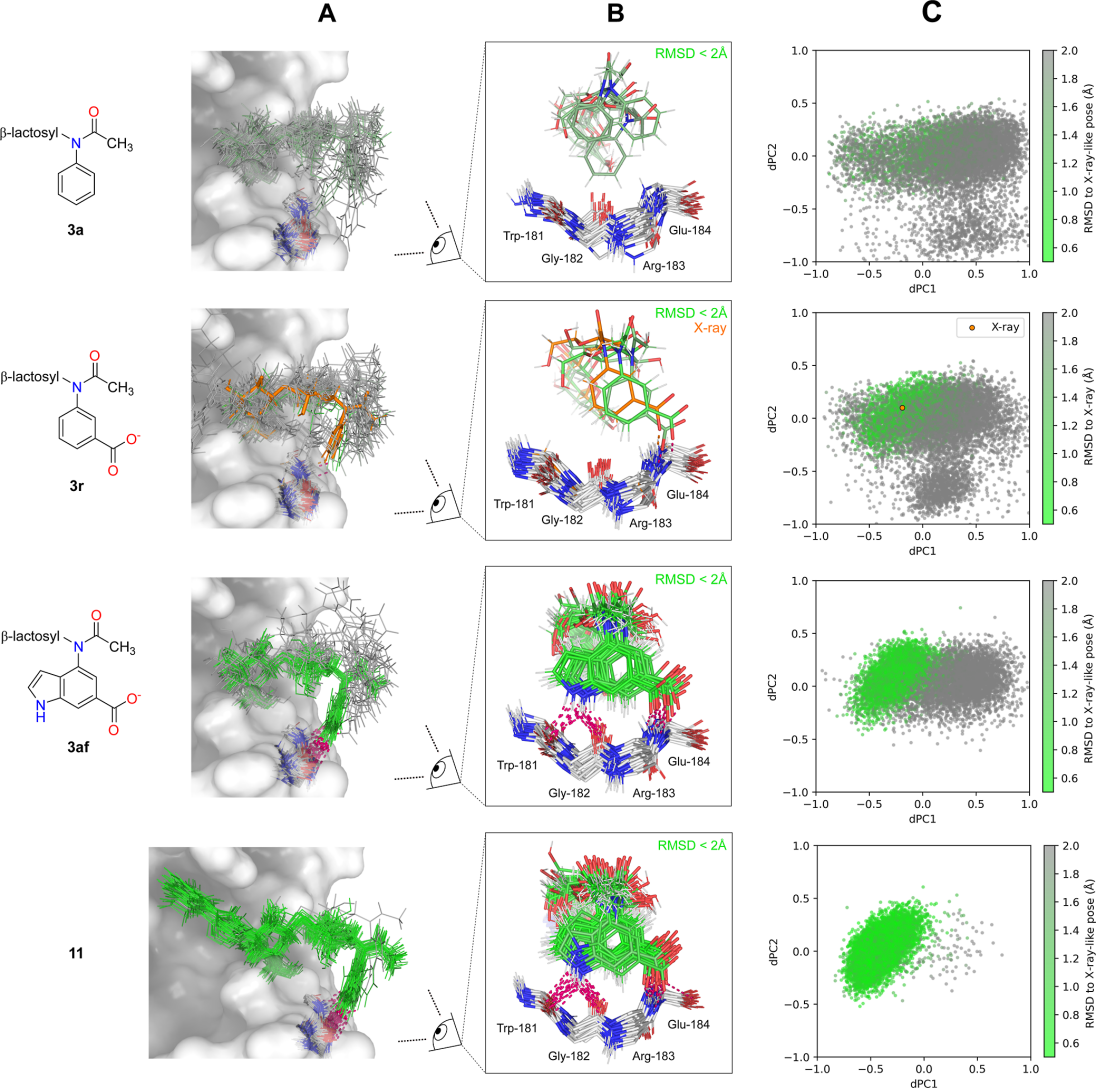
MD simulations of compounds **3a**, **3r**, **3af**, and **11**. (A) Thirty representative frames from a total of 3 μs of simulations (each frame represents 100 ns of simulation). Frames are colored by RMSD relative to the X-ray-derived pose of **3r**. The X-ray structure of **3r** is shown in orange. (B) Same as in (A), but only frames with RMSD <2 Å are shown from a different view. Hydrogen bonding with the key backbone region is highlighted. (C) dPCA based on 10,000 frames. Coloring is consistent with the rest of the figure.

Simulation of **3a** revealed no clear ordering of the phenyl moiety near the favorable backbone region of the receptor, consistent with its weak experimental affinity. The *m*-carboxyphenyl amide **3r** still exhibited mostly disordered behavior; however, occasional ordering into the X-ray-like pose was detected (2 of 30 frames with RMSD <2 Å). Reflecting the crystallographic observations, a charge-assisted hydrogen bond between the carboxylate group of **3r** and the backbone NH of Glu-184 was detected in the simulation, likely accounting for its enhanced affinity compared with **3a**.

For the indolyl amide **3af**, a strong preference for the X-ray-like pose was evident (16 of 30 frames with RMSD <2 Å). The 6-carboxyindolyl moiety formed a characteristic interaction pattern with the receptor, consisting of hydrogen bonding between the ligand carboxylate and the backbone NH of Glu-184 (as in the case of **3r**) and bifurcated hydrogen bonding between the indole NH group and the backbone carbonyls of Trp-181 and Gly-182.

Finally, the most potent inhibitor **11** exhibited even stronger ordering into the favorable X-ray-like conformation (29 of 30 frames with RMSD <2 Å). dPCA identified this pose as a dominant conformational cluster, supporting a well-defined binding mode that combines the previously described binding of the 4-(3-fluorophenyl)-1,2,3-triazol-1-yl group with the newly identified hydrogen-bonding motif of the 6-carboxyindole moiety. The binding mode of **11** in Gal-3C is further illustrated in Figure 5A as a single structure obtained by optimizing a representative frame from the MD simulation.

**Figure 5:**
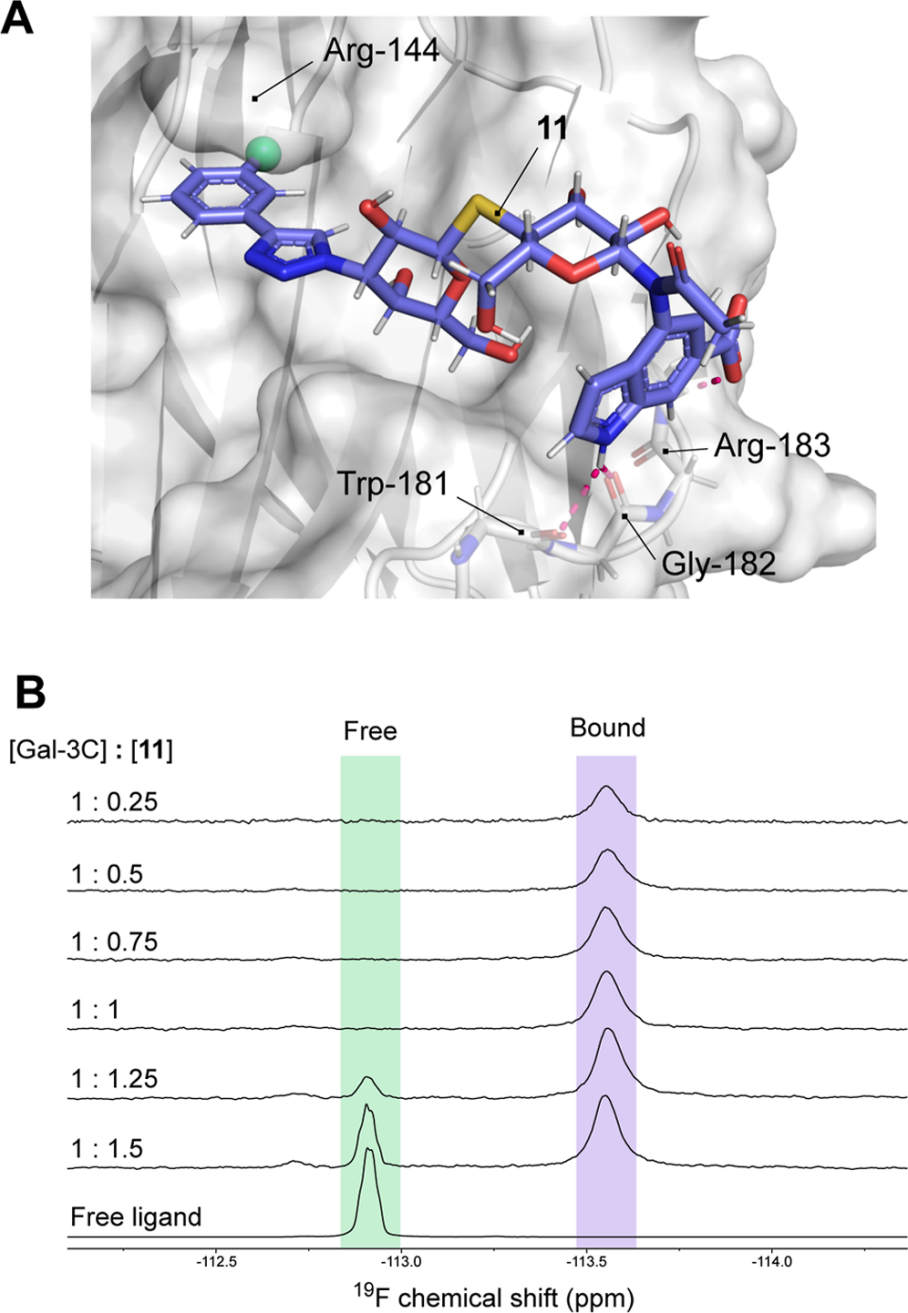
Binding of compound **11** to Gal-3C. (A) Representative MD frame optimized by QM/MM. The fluorine atom is shown as a green sphere. (B) Binding of **11** to Gal-3C elucidated by ^19^F NMR.

To further investigate the interaction of **11** with the receptor, we also took advantage of the presence of a fluorine atom in **11** and examined its binding to the receptor by ^19^F NMR spectroscopy (Figure 5B). Titration of **11** to Gal-3C in PBS revealed a clear transition from the signal of the free ligand to a single, well-defined resonance corresponding to the bound state, indicating localization of the fluorine atom at a unique site within the receptor. The observed ^19^F chemical shift of the bound state is consistent with binding in subsite B, defined by the Arg-144 residue, as ascertained previously.^35,36^

### Assessment of In Vitro Pharmacokinetics and Cytotoxicity

Compounds **3ad**, **3af**, **10**, and **11**, identified as the most potent Gal-3C binders, were selected for evaluation of their ADME properties (Table 5). Compounds **3ad** and **3af** retain the native *O*-glycosidic linkage between the galactose and glucose subunits of the lactose scaffold, whereas compounds **10** and **11** feature a non-natural *S*-glycosidic linkage together with an additional 3-fluorophenyltriazolyl modification. All four compounds demonstrated high metabolic stability in human, mouse, and rat plasma, as well as in liver microsomes across species. Only indolyl amide **3af** exhibited slightly reduced stability in mouse plasma (76% remaining after 2 h). When comparing against **GB0139**, all compounds **3ad**, **3af**, **10**, and **11** demonstrated comparable stability in plasma and somewhat better stability in human and mouse liver microsomes, suggesting higher resistance to cytochrome P450 metabolism. However, the overall differences in metabolic between **GB0139** and our lead compound **11** are not dramatic.

**Table 5:** Metabolic Stability of Compounds **3ad**, **3af**, **10**, and **11** ^a^ ^,^ ^d^

	plasma stability			microsomal stability			plasma protein binding		
	% remaining at 2 h			CL_int_ (μL/min/mg)			*F*_b_ (%)		
compound	human	mouse	rat	human	mouse	rat	human	mouse	rat
**GB0139**	110 ± 5	68 ± 3	78 ± 8	12 ± 6	6 ± 5	<2	-^b^	-^b^	-^b^
**3ad**	99 ± 8	110 ± 2	140 ± 7	<2	<2	<2	6	7	6
**3af** ^c^	100	76	94	3	<2	<2	<5	<5	27
**10**	92 ± 6	89 ± 5	110 ± 5	<2	<2	<2	34	22	25
**11**	110 ± 6	100 ± 3	87 ± 6	<2	<2	<2	45	43	90

a Quantification was performed by Echo-MS unless indicated otherwise. Values represent means from three independent experiments. Uncertainties are given as SD where applicable.

b Not obtained.

c Data for this compound were obtained by LC–MS instead of Echo-MS and come from a single experiment.

d CL_int_: intrinsic clearance; *F*_b_: fraction bound.

The monosubstituted compounds **3ad** and **3af** showed low plasma protein binding (<10%), which can be attributed to their polarity, except for **3af** in rat plasma, which showed somewhat higher binding (27%). In contrast, compounds **10** and **11** exhibited moderate plasma protein binding (22–90%), reflecting their increased lipophilicity and aromatic surface area. Altogether, these ADME results are in line with the precedent results for **GB0139** that were reported in vivo,^37,38^ as well as with in vitro data for monosaccharide-based^10^ galectin inhibitors, indicating a favorable ADME profile.

To establish a nontoxic concentration range suitable for downstream efficacy studies, in vitro cytotoxicity of **11** was evaluated in five human cell lines: HEK293, HepG2, THP-1, Jurkat E6.1, and LX2 (Table 6). Results were benchmarked against the reference inhibitor **GB0139**. No cytotoxicity was detected for either **11** or **GB0139** across this diverse panel of human cell lines (CC_50_ > 300 μM), highlighting their favorable in vitro safety profiles and supporting their use at pharmacologically relevant concentrations in cell-based assays. Importantly, the tolerance of all tested cell types to high compound concentrations reduces the likelihood of off-target cytotoxic mechanisms, suggesting a selective mode of action.^39,40^ These findings align with expectations for Gal-3-targeting glycomimetics and underscore the potential of **11** as a lead candidate.

**Table 6:** In Vitro Cytotoxicity of Compounds **11** and **GB0139** in Relevant Cell Lines

	CC_50_ (μM)^a^				
compound	Hek293	HepG2	THP1	Jurkat	LX2
**GB0139**	>300	>300	>300	>300	>300
**11**	>300	>300	>300	>300	>300

a Data are reported as means from two independent experiments, each performed in duplicate.

### Engagement of Gal-3 by Compound **11** in a THP1 Cell Model

Given the central role of Gal-3 in macrophage–myofibroblast crosstalk under inflammatory conditions such as fibrosis, we assessed the intracellular target engagement by compound **11** in a THP1 cell model. Gal-3 signals were quantified as median fluorescence intensity (MFI) in both undifferentiated monocyte-like cells and PMA-differentiated macrophage-like THP1 cells (Figure 6A). Treatment of these cell lines with **11** resulted in a clear, concentration-dependent reduction in the intracellular Gal-3 signal across both phenotypes, with a more pronounced effect in macrophage-like cells. In contrast, the reference inhibitor **GB0139** reduced the Gal-3 signal only in macrophage-like cells and exerted a minimal effect in the monocyte-like phenotype. Importantly, compound **11** exhibited superior target engagement compared with **GB0139**, which acted on both phenotypes. Although the precise reasons for this observation are not known to us at this point, this functional advantage could be a result of the unique physicochemical properties of **11** that may enhance membrane interaction, passive diffusion and/or endosomal escape, potentially even stability in the intracellular environment or stronger binding to intracellular Gal-3, which could result in its enhanced degradation. The precise mechanism will be a subject of further investigation.^10,18,41^

**Figure 6:**
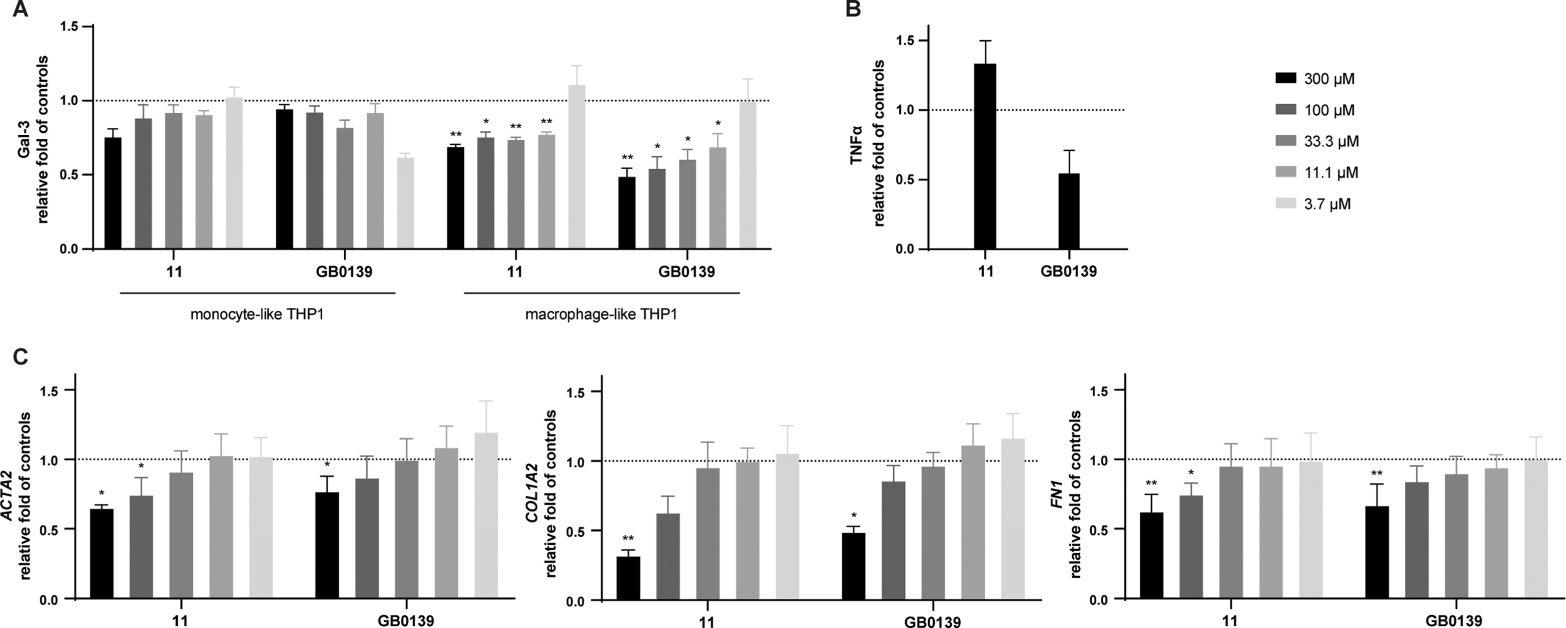
Compound **11** selectively engages intracellular Gal-3 and inhibits fibrotic signaling independently of acute inflammatory modulation. (A) Compound **11** shows effective intracellular engagement of Gal-3 in macrophage-like THP1 cells. Intracellular engagement of Gal-3 in (−)­PMA (monocyte-like) and (+)­PMA (macrophage-like) THP1 cells by inhibitors **11** and **GB0139**. Data are expressed as MFI of Gal-3 relative to untreated controls, evaluated using mixed-effects analysis with Fisher’s LSD test (*P < 0.05, **P < 0.01). (B) Compound **11** does not reduce tumor necrosis factor-α (TNFα) secretion in LPS-stimulated macrophage-like THP1 cells. LPS-stimulated (+)­PMA THP1 cells treated with 300 μM **11** and **GB0139** to assess maximal inhibitory potential. Secreted TNFα was evaluated as a fold change relative to untreated LPS-stimulated control cells. (C) Compound **11** exhibits enhanced antifibrotic activity in TGFβ-stimulated LX2 hepatic stellate cells. LX2 cells were stimulated with TGFβ to induce a fibrotic phenotype and treated with **11** or **GB0139** for 48 h to inhibit fibrotic progression. Expression of profibrotic genes (*ACTA2*, *COL1A2*, and *FN1*) was quantified using RT-qPCR. Data are presented as relative mRNA expression relative to compound-untreated TGFβ-stimulated controls normalized to *ACTB* expression, evaluated using the Friedman test with uncorrected Dunn’s test (*P < 0.05, **P < 0.01). All data are expressed as the mean ± SEM from two independent experiments.

To model an acute inflammatory environment, we next investigated the anti-inflammatory effects of **11** in PMA-differentiated THP1 macrophage-like cells stimulated with LPS (Figure 6B). TNFα secretion was used as a biomarker of proinflammatory activation, reflecting TLR-mediated signaling. Despite prior evidence of effective intracellular Gal-3 engagement by **11** in the same cellular model (Figure 6A), we observed no suppression of LPS-induced TNFα secretion, even at the highest tested concentration (300 μM). TNFα levels remained indistinguishable from those in the LPS-stimulated control group, indicating that compound **11** fails to exert a detectable anti-inflammatory effect under these acute stimulation conditions, in contrast to **GB0139**, which effectively reduced TNFα secretion. This outcome suggests that while **11** efficiently engages Gal-3, this interaction may not disrupt early TLR4-driven proinflammatory signaling cascades.^42^ Moreover, this finding underscores the importance of the cellular and microenvironmental context in Gal-3-mediated immune regulation. The immunomodulatory role of Gal-3 might be more prominent in chronic inflammatory settings and intercellular crosstalk, such as in tissue-resident macrophages or multicellular immune environments, rather than in acutely stimulated monocytic models. The THP1 macrophage-like model, while suitable for studying acute cytokine responses, may lack the complexity required to observe Gal-3-dependent modulation of immune circuits.^42,43^

### Inhibition of TGFβ-Induced Fibrosis in LX2 Cells by Compound **11**

As part of the functional characterization of compound **11**, its antifibrotic activity was examined in a TGFβ-stimulated LX2 hepatic stellate cell model, a widely used in vitro system for mimicking liver fibrosis.^10,44^ TGFβ-stimulated cells were treated with either **11** or **GB0139** for 48 h (Figure 6C), and the expression of key profibrotic genes was assessed by quantitative reverse transcription polymerase chain reaction (RT-qPCR). Specifically, we measured mRNA levels of *ACTA2* (encoding α-smooth muscle actin, α-SMA), *COL1A2* (collagen, type I, α-2 chain), and *FN1* (fibronectin 1), all of which are hallmark indicators of hepatic stellate cell activation and extracellular matrix deposition (ECM).

Treatment of the LX2 cells with **11** led to a dose-dependent downregulation of all three profibrotic markers, reflecting its capacity to counteract TGFβ-driven fibrotic signaling at the transcriptional level. The overall response magnitude was comparable to that of **GB0139**; however, compound **11** achieved greater suppression of *COL1A2*, suggesting enhanced inhibition of collagen type I production, a key driver of fibrotic matrix accumulation. This enhanced potency on *COL1A2* expression may reflect differential modulation of intracellular Gal-3 activity, potentially impacting SMAD signaling or transcriptional regulation of ECM-related genes.^45,46^

Associating antifibrotic efficacy with the lack of TNFα suppression in LPS-stimulated THP1 macrophage-like cells, we hypothesize that the activity of compound **11** may preferentially affect pathways directly linked to fibrosis rather than acute inflammatory signaling. The lack of TNFα inhibition likely reflects the dominant role of extracellular Gal-3 and other costimulatory factors in acute cytokine release. In contrast, the fibrotic phenotype in LX2 cells may be more sensitive to the intracellular Gal-3 blockade, potentially involving nuclear or cytoplasmic pools of Gal-3 that regulate TGFβ-dependent transcription.^10,47,48^ These findings highlight a potential mechanistic selectivity of compound **11**, favoring fibrosis-associated pathways over classical cytokine release in such in vitro settings.

## Conclusions

In summary, we report *N*-aryl-*N*-lactosylamides as a new class of disaccharide-based inhibitors of Gal-3. These compounds are readily accessible from lactose via *N*-glycosylation chemistry using a rich pool of commercially available aromatic amines, enabling efficient exploration of chemical space within this series. Thorough investigation of the structure–activity relationship identified the 6-carboxyindol-4-yl acetamide moiety as a highly potent binding motif with excellent selectivity for Gal-3. The presence of a negatively charged carboxylic (or tetrazole) group at the C-6 position of the indole was found to be the key determinant of selectivity. The 6-carboxyindol-4-yl motif interacts with Gal-3 through a combination of charge-assisted hydrogen bonding of the carboxylate with the backbone NH of Glu-184, and bifurcated hydrogen bonding of the indole NH with the backbone carbonyls of Trp-181 and Gly-182.

Based on results from the exploratory lactose-derived series, we synthesized the doubly decorated inhibitor **11** based on thiolactose, combining the newly discovered 6-carboxyindol-4-yl motif with a previously known 3-fluorophenyltriazole modification of the galactose subunit. This compound achieves low-nanomolar affinity toward Gal-3 while maintaining excellent selectivity. Inhibitor **11** also exhibits high in vitro metabolic stability in both plasma and microsomes and shows no cytotoxicity across a diverse panel of human cell lines at pharmacologically relevant concentrations.

Compound **11** effectively engages Gal-3 in both monocyte- and macrophage-like THP1 cells, with stronger activity in the latter. At the same time, it showed limited impact on acute proinflammatory signaling, given that TNFα secretion remained unaffected in LPS-driven macrophage-like cells. Yet, compound **11** exhibits robust in vitro antifibrotic activity in TGFβ-stimulated LX2 hepatic stellate cells by downregulating profibrotic markers, outperforming the reference inhibitor **GB0139**. This functional divergence points to a mechanistic selectivity of **11** favoring intracellular Gal-3 modulation in fibrotic signaling over acute cytokine-mediated inflammation, underscoring its therapeutic promise in the context of fibrotic disease.

## Experimental Section

### Chemistry

Yields were determined based on the amount of isolated compound. All reactions requiring anhydrous conditions were carried out under an argon atmosphere. Tetrahydrofuran and dichloromethane were dried by distillation over LiAlH_4_ and CaH_2_, respectively. Other commercially available solvents and reagents were used as received, unless otherwise indicated. Column chromatography was performed using silica gel (60 Å pore size, 40–63 μm particle size). Thin-layer chromatography (TLC) was conducted on silica gel 60 F_254_-coated aluminum sheets (Merck) and visualized under UV light, by staining with cerium­(IV) sulfate solution (1% in 10% H_2_SO_4_) or using a combination of both methods. Preparative HPLC separations were performed on a puriFlash 5.250 system (Advion Interchim Scientific, France) using a Luna Omega 5 μm Polar C18 column (100 Å, 250 × 21.5 mm). Analytical HPLC was conducted on a Waters Alliance 2695 system with UV detection (Waters 2996) using a Kromasil 5 μm Polar C18 LC column (100 Å, 250 × 4.6 mm). All compounds are >95% pure by HPLC analysis. HPLC traces for representative compounds are included in the Supporting Information. All ^1^H and ^13^C NMR spectra were recorded using the following instruments: Bruker Avance III HD 400 MHz, Bruker Avance III HD 500 MHz, Bruker Avance III HD 600 MHz, JEOL EZCR 400 MHz, and JEOL JNM-ECZR 500 MHz. Chemical shifts (δ) are reported in ppm and referenced to residual solvent signals or internal standards (1,4-dioxane or *tert*-butanol). Signal assignments were confirmed by 2D experiments: ^13^C-APT, ^1^H–^1^H COSY, ^1^H–^13^C HSQC, ^1^H–^13^C HMBC, and ^1^H–^1^H ROESY. Infrared (IR) spectra were recorded on a Nicolet 6700 spectrometer (Thermo Fisher Scientific). High-resolution mass spectroscopy (HRMS) was performed on an LTQ Orbitrap XL (Thermo Fisher Scientific) spectrometer using electrospray ionization (ESI) in positive or negative mode. Nominal and exact *m*/*z* values are reported in daltons. Optical rotations were measured at 20 °C using an AUTOPOL IV polarimeter (Rudolph Research Analytical, USA) at 589 nm (sodium D-line) with a path length of 1.0 dm. Melting points (mp) were recorded using an MPM-HV3 melting point apparatus (Schorpp-Gerätetechnik, Germany).

#### General Procedure A: Synthesis of *N*-Lactosyl Anilines

Hepta-*O*-acetyl lactose **1** (0.320 g, 0.5 mmol) and corresponding aniline (0.85 mmol) were dissolved in dry CH_3_OH (5 mL) and glacial AcOH (0.05 mL, 0.87 mmol). The reaction mixture was stirred at 67 °C for 1 to 7 days, until TLC analysis (CyH/EtOAc 1:2) indicated complete consumption of the starting material. The reaction mixture was then concentrated in vacuo; the resulting residue containing the crude *N*-lactosylaniline was used in subsequent steps without purification, unless otherwise specified.

#### General Procedure B: N-Acylation of Lactosyl Anilines

Hepta-*O*-acetyl-*N*-lactosylaniline (0.5 mmol) was dissolved in CH_3_NO_2_ (5 mL) and Ac_2_O (0.1 mL, 1.1 mmol), followed by the addition of ZnCl_2_ (0.082 g, 0.6 mmol). The reaction mixture was stirred at room temperature for 30 min, after which the TLC analysis confirmed complete conversion of the starting material. The reaction mixture was diluted with EtOAc (30 mL) and washed sequentially with saturated aqueous solution of NaHCO_3_ (10 mL) and brine (10 mL). The organic phase was separated and dried over anhydrous MgSO_4_, filtered, and concentrated in vacuo. The residue was purified by liquid column chromatography on silica gel (25% → 90% EtOAc in CyH), affording the N-acylated products. For derivatives containing carboxy or tetrazole functional groups, the NaHCO_3_ wash step was omitted, and chromatography was performed using mobile phase 20:1 → 15:1 CHCl_3_/EtOH.

#### General Procedure C: Removal of *O*-Acetyl Protecting Groups by Zemplén Deacetylation

Acetylated *N*-lactosylamide (0.5 mmol) was dissolved in CH_3_OH (5 mL), and the pH of the solution was adjusted to 8–9 by dropwise addition of 1 M CH_3_ONa solution in CH_3_OH (typically 0.05 mL for neutral compounds; up to 0.55 mL for carboxylic or tetrazole derivatives). The reaction mixture was stirred at room temperature for 3 h, after which Dowex 50WX8 (H^+^ form) was added to neutralize the reaction. The resulting slurry was filtered, and the resin was washed with CH_3_OH. The filtrate was concentrated in vacuo, and the residue purified by liquid column chromatography on silica gel (9:1 CH_3_CN/H_2_O), affording the deprotected product.

#### General Procedure D: Removal of *O*-Acetyl Protecting Groups with Ammonia

Acetylated *N*-lactosylamide (0.5 mmol) was dissolved in methanolic NH_3_ (7 N, 5 mL) and stirred at room temperature overnight. The reaction mixture was then concentrated in vacuo, and the resulting residue was purified by liquid column chromatography on silica gel (9:1 CH_3_CN/H_2_O), affording the deprotected product. Compounds **2a**–**2ae** were synthesized according to general procedures A and B. Full experimental details, including the synthesis of noncommercially available aniline precursors, are provided in the Supporting Information.

##### *N*-[4-*O*-(β-d-Galactopyranosyl)-β-d-glucopyranosyl]-*N*-phenylacetamide (**3a**)

Following general procedure C, lactosylamide **2a** (0.450 g, 0.597 mmol) gave **3a** (0.290 g, 99%) as a white foam. The analytical sample was obtained by HPLC (A: H_2_O, B: CH_3_OH; 30% to 90% B in 30 min, followed by isocratic 90% B for 10 min). [α]_D_^20^ = +42.8 (*c* 0.2 in H_2_O); ^1^H NMR (500 MHz, DMSO-*d*_6_, *T* = 100 °C): δ 1.79 (s, 3H, CH_3_CON), 2.79 (ddd, *J*_2,3_ = 9.4, *J*_2,1_ = 8.7, *J*_2,OH_ = 5.4 Hz, 1H, H-2), 3.16 (dd, *J*_4,5_ = 9.7, *J*_4,3_ = 8.8 Hz, 1H, H-4), 3.33 (ddd, *J*_3′,2′_ = 9.4, *J*_3′,OH_ = 4.9, *J*_3′,4′_ = 3.4 Hz, 1H, H-3′), 3.37 (ddd, *J*_2′,3′_ = 9.4, *J*_2′,1′_ = 7.3, *J*_2′,OH_ = 3.7 Hz, 1H, H-2′), 3.41 (ddd, *J*_5,4_ = 9.6, *J*_5,6a_ = 4.6, *J*_5,6b_ = 2.8 Hz, 1H, H-5), 3.43 (ddd, *J*_5′,6a′_ = 6.7, *J*_5′,6b′_ = 5.6, *J*_5′,4′_ = 1.1 Hz, 1H, H-5′), 3.48 (td, *J*_3,2_ = *J*_3,4_ = 8.8, *J*_3,OH_ = 1.8 Hz, 1H, H-3), 3.52 (ddd, *J*_gem_ = 10.8, *J*_6′a,5′_ = 6.6, *J*_6′a,OH_ = 5.3 Hz, 1H, H-6′a), 3.57 (dt, *J*_gem_ = 10.9, *J*_6′b,5′_ = *J*_6′b,OH_ = 5.5 Hz, 1H, H-6′b), 3.64 (ddd, *J*_gem_ = 11.9, *J*_6a,5_ = 6.3, *J*_6a,OH_ = 4.7 Hz, 1H, H-6a), 3.67 (ddd, *J*_4′,OH_ = 4.4, *J*_4′,3′_ = 3.3, *J*_4′,5′_ = 1.2 Hz, 1H, H-4′), 3.79 (ddd, *J*_gem_ = 11.9, *J*_6b,OH_ = 5.7, *J*_6b,5_ = 2.7 Hz, 1H, H-6b), 4.06 (d, *J*_OH,4′_ = 4.5 Hz, 1H, OH-4′), 4.10 (t, *J*_OH,6_ = 6.1 Hz, 1H, OH-6), 4.20 (t, *J*_OH,6′_ = 5.4 Hz, 1H, OH-6′), 4.21 (d, *J*_1′,2′_ = 7.5 Hz, 1H, H-1′), 4.27 (d, *J*_OH,3′_ = 5.4 Hz, 1H, OH-3′), 4.46 (d, *J*_OH,3_ = 1.9 Hz, 1H, OH-3), 4.65 (d, *J*_OH,2_ = 5.4 Hz, 1H, OH-2), 4.66 (d, *J*_OH,2′_ = 4.6 Hz, 1H, OH-2′), 5.51 (d, *J*_1,2_ = 8.6 Hz, 1H, H-1), 7.35–7.44 (m, 5H, H-2″,3″,4″); ^13^C NMR (126 MHz, DMSO-*d*_6_, *T* = 100 °C): δ 22.35 (**C**H_3_CON), 60.10 (CH_2_-6′), 60.53 (CH_2_-6), 67.89 (CH-4′), 69.39 (CH-2), 70.38 (CH-2′), 73.04 (CH-3′), 75.14 (CH-5′), 75.41 (CH-3), 76.63 (CH-5), 79.51 (CH-4), 82.23 (CH-1), 103.05 (CH-1′), 127.36 (CH-4″), 128.06 (CH-2″), 129.82 (CH-3″), 138.14 (C-1″), 169.89 (CH_3_ **C**ON); IR (KBr): 3381, 3068, 2974, 2929, 1652, 1596, 1494, 1452, 1399, 1376, 1333, 1283, 1160, 1117, 1081, 1050, 914, 754, 703 cm^–1^; HRMS (ESI): [M + Na]^+^ *m*/*z* calcd for C_20_H_29_O_11_NNa: 482.1633, found: 482.1635; [M + H]^+^ *m*/*z* calcd for C_20_H_30_O_11_N: 460.1813, found: 460.1817.

##### *N*-[4-*O*-(β-d-Galactopyranosyl)-β-d-glucopyranosyl]-*N*-(4-methylphenyl)­acetamide (**3b**)

Following general procedure C, lactosylamide **2b** (0.420 g, 0.547 mmol) gave **3b** (0.240 g, 93%) as a white foam. The analytical sample was obtained by HPLC (A: H_2_O, B: CH_3_OH; 30% to 90% B in 30 min, followed by isocratic 90% B for 10 min). [α]_D_^20^ = +40.6 (*c* 0.2 in CH_3_OH); ^1^H NMR (500 MHz, D_2_O, *T* = 100 °C): δ 1.88 (s, 3H, CH_3_CON), 2.39 (s, 3H, CH_3_-Ph), 3.06 (t, *J*_2,1_ = *J*_2,3_ = 9.2 Hz, 1H, H-2), 3.47 (dd, *J*_4,5_ = 9.8, *J*_4,3_ = 9.2 Hz, 1H, H-4), 3.52 (dd, *J*_2′,3′_ = 10.0, *J*_2′,1′_ = 7.7 Hz, 1H, H-2′), 3.63 (dd, *J*_3′,2′_ = 10.0, *J*_3′,4′_ = 3.4 Hz, 1H, H-3′), 3.66 (ddd, *J*_5′,6a′_ = 8.0, *J*_5′,6b′_ = 3.8, *J*_5′,4′_ = 1.0 Hz, 1H, H-5′), 3.59–3.77 (m, 1H, H-5), 3.70 (dd, *J*_gem_ = 11.8, *J*_6b′,5′_ = 3.8 Hz, 1H, H-6b′), 3.71 (t, *J*_3,2_ = *J*_3,4_ = 9.2 Hz, 1H, H-3), 3.74 (dd, *J*_gem_ = 11.8, *J*_6a′,5′_ = 8.1 Hz, 1H, H-6a′), 3.80 (dd, *J*_gem_ = 12.3, *J*_6a,5_ = 5.1 Hz, 1H, H-6a), 3.89 (dd, *J*_4′,3′_ = 3.4, *J*_4′,5′_ = 1.0 Hz, 1H, H-4′), 3.97 (dd, *J*_gem_ = 12.3, *J*_6b,5_ = 2.2 Hz, 1H, H-6b), 4.38 (d, *J*_1′,2′_ = 7.8 Hz, 1H, H-1′), 5.68 (d, *J*_1,2_ = 9.2 Hz, 1H, H-1), 7.29 (d, *J*_2″,3′′_ = 8.5 Hz, 2H, H-2″), 7.35 (d, *J*_3″,2′′_ = 8.5 Hz, 2H, H-3″); ^13^C NMR (126 MHz, D_2_O, *T* = 100 °C): δ 20.89 (CH_3_Ph), 23.42 (**C**H_3_CON), 60.82 (CH_2_-6), 61.74 (CH_2_-6′), 69.25 (CH-4′), 69.95 (CH-2), 71.62 (CH-2′), 73.18 (CH-3′), 76.02 (CH-5′), 76.29 (CH-3), 77.52 (CH-5), 78.37 (CH-4), 82.63 (CH-1), 103.50 (CH-1′), 130.23 (CH-2″), 130.65 (CH-3″), 135.09 (C-4″), 140.57 (C-1″), 177.00 (CH_3_ **C**ON); IR (CH_3_OH): 3369, 2923, 2889, 1653, 1606, 1576, 1512, 1373, 1075, 1041, 832, 783, 703, 650, 607, 566 cm^–1^; HRMS (ESI): [M + Na]^+^ *m*/*z* calcd for C_21_H_31_O_11_NNa: 496.1789, found: 496.1797.

##### *N*-[4-*O*-(β-d-Galactopyranosyl)-β-d-glucopyranosyl]-*N*-(3-ethynylphenyl)­acetamide (**3c**)

Following general procedure C, lactosylamide **2c** (0.220 g, 0.283 mmol) gave **3c** (0.127 g, 93%) as a white foam. The analytical sample was obtained by HPLC (A: H_2_O, B: CH_3_OH; 30% to 90% B in 30 min, followed by isocratic 90% B for 10 min). [α]_D_^20^ = +49.3 (*c* 0.4 in CH_3_OH); ^1^H NMR (500 MHz, DMSO-*d*_6_, *T* = 100 °C): δ 1.84 (s, 3H, CH_3_CON), 2.77 (td, *J*_2,1_ = *J*_2,3_ = 9.1, *J*_2,OH_ = 5.3 Hz, 1H, H-2), 3.17 (dd, *J*_4,5_ = 9.8, *J*_4,3_ = 8.8 Hz, 1H, H-4), 3.30–3.36 (m, 1H, H-3′), 3.38 (ddd, *J*_2′,3′_ = 9.6, *J*_2′,1′_ = 7.7, *J*_2′,OH_ = 2.4 Hz, 1H, H-2′), 3.42 (ddd, *J*_5,4_ = 9.7, *J*_5,6a_ = 4.9, *J*_5,6b_ = 2.8 Hz, 1H, H-5), 3.44 (ddd, *J*_5′,6a′_ = 6.7, *J*_5′,6b′_ = 5.6, *J*_5′,4′_ = 1.2 Hz, 1H, H-5′), 3.48 (td, *J*_3,2_ = *J*_3,4_ = 8.7, *J*_3,OH_ = 1.7 Hz, 1H, H-3), 3.51–3.55 (m, 1H, H-6a′), 3.57 (dt, *J*_gem_ = 10.5, *J*_6b′,5′_ = *J*_6b′,OH_ = 5.1 Hz, 1H, H-6b′), 3.63 (ddd, *J*_gem_ = 11.9, *J*_6a,OH_ = 6.0, *J*_6a,5_ = 5.0 Hz, 1H, H-6a), 3.67 (ddd, *J*_4′,OH_ = 4.3, *J*_4′,3′_ = 3.0, *J*_4′,5′_ = 1.1 Hz, 1H, H-4′), 3.80 (ddd, *J*_gem_ = 11.9, *J*_6b,OH_ = 5.6, *J*_6a,5_ = 2.6 Hz, 1H, H-6b), 3.99 (s, 1H, C≡CH), 4.06 (d, *J*_OH,4′_ = 4.3 Hz, 1H, OH-4′), 4.14 (t, *J*_OH,6a_ = *J*_OH,6b_ = 5.9 Hz, 1H, OH-6), 4.20 (t, *J*_OH,6′a_ = *J*_OH,6′b_ = 5.3 Hz, 1H, OH-6′), 4.22 (d, *J*_1′,2′_ = 7.5 Hz, 1H, H-1′), 4.27 (br s, 1H, OH-3′), 4.49 (d, *J*_OH,3_ = 1.8 Hz, 1H, OH-3), 4.65 (d, *J*_OH,2′_ = 3.6 Hz, 1H, OH-2′), 4.77 (d, *J*_OH,2_ = 5.4 Hz, 1H, OH-2), 5.47 (d, *J*_1,2_ = 8.6 Hz, 1H, H-1), 7.33–7.44 (m, 2H, Ar-H), 7.45–7.52 (m, 2H, Ar-H); ^13^C NMR (126 MHz, DMSO-*d*_6_, *T* = 100 °C): δ 22.24 (**C**H_3_CON), 60.09 (CH_2_-6′), 60.49 (CH_2_-6), 67.88 (CH-4′), 69.41 (CH-2), 70.37 (CH-2′), 73.04 (CH-3′), 75.13 (CH-3), 75.34 (CH-5′), 76.71 (CH-5), 79.48 (CH-4), 80.17 (C≡**C**H), 82.26 (CH-1), 82.41 (**C**≡CH), 103.05 (CH-1′), 121.85 (C-3″), 128.48 (Ar-H), 130.72 (Ar-H), 130.79 (Ar-H), 132.88 (Ar-H), 138.32 (C-1″), 169.82 (CH_3_ **C**ON); IR (CH_3_OH): 3370, 3070, 2931, 2887, 2833, 2106, 1660, 1597, 1577, 1481, 1425, 1374, 1165, 1116, 1077, 1039, 1001, 698, 668, 627 cm^–1^; HRMS (ESI): [M + Na]^+^ *m*/*z* calcd for C_22_H_29_O_11_NNa: 506.1633, found: 506.1635.

##### *N*-[4-*O*-(β-d-Galactopyranosyl)-β-d-glucopyranosyl]-*N*-(3-trifluoromethylphenyl)­acetamide (**3d**)

Following general procedure D, lactosylamide **2d** (0.411 g, 0.5 mmol) gave **3d** (0.200 g, 76%) as a white foam. The analytical sample was obtained by HPLC (A: H_2_O, B: CH_3_OH; 30% to 90% B in 30 min, followed by isocratic 90% B for 10 min). [α]_D_^20^ = +44.1 (*c* 0.3 in CH_3_OH); ^1^H NMR (500 MHz, D_2_O, *T* = 100 °C): δ 2.01 (s, 3H, CH_3_CON), 3.03 (dd, *J*_2,1_ = 9.5, *J*_2,3_ = 8.8 Hz, 1H, H-2), 3.43 (dd, *J*_4,5_ = 9.8, *J*_4,3_ = 8.9 Hz, 1H, H-4), 3.55 (dd, *J*_2′,3′_ = 9.9, *J*_2′,3′_ = 7.7 Hz, 1H, H-2′), 3.63 (dd, *J*_3′,2′_ = 9.8, *J*_3′,4′_ = 3.4 Hz, 1H, H-3′), 3.66 (ddd, *J*_5′,6′a_ = 7.3, *J*_5′,6′a_ = 4.7, *J*_5′,4′_ = 1.1 Hz, 1H, H-5′), 3.69–3.77 (m, 4H, H-3,5,6′a,6′b), 3.80 (dd, *J*_gem_ = 12.3, *J*_6a,5_ = 5.2 Hz, 1H, H-6a), 3.92 (dd, *J*_4′,3′_ = 3.5, *J*_4′,5′_ = 1.1 Hz, 1H, H-4′), 3.99 (dd, *J*_gem_ = 12.3, *J*_6b,5_ = 2.4 Hz, 1H, H-6b), 4.40 (d, *J*_1′,2′_ = 7.8 Hz, 1H, H-1′), 5.60 (br s, 1H, H-1), 7.67 (ddd, *J*_6″,5′′_ = 7.7, *J*_6″,2′′_ = 1.8, *J*_6″,4′′_ = 1.4 Hz, 1H, H-6″), 7.71 (td, *J*_5″,6′′_ = *J*_5″,4′′_ = 7.7, *J*_5″,2′′_ = 0.8 Hz, 1H, H-5″), 7.73–7.74 (m, 1H, H-2″), 7.84 (ddd, *J*_4″,5′′_ = 7.7, *J*_4″,6′′_ = 1.4, *J*_4″,5′′_ = 0.8 Hz, 1H, H-4″); ^13^C NMR (126 MHz, D_2_O, *T* = 100 °C): δ 22.83 (**C**H_3_CON), 61.20 (CH_2_-6), 61.47 (CH_2_-6′), 69.20 (CH-4′), 70.13 (CH-2), 71.59 (CH-2′), 73.32 (CH-3′), 75.81 (CH-5′), 76.06 (CH-3), 77.40 (CH-5), 79.01 (CH-4), 83.54 (CH-1), 103.39 (CH-1′), 124.18 (q, *J*_C,F_ = 271.9 Hz, **C**F_3_), 126.35 (q, *J*_C,F_ = 4.3 Hz, CH-4″), 127.17 (q, *J*_C,F_ = 3.9 Hz, C-2″), 130.77 (CH-5″), 131.38 (q, *J*_C,F_ = 32.4 Hz, C-3″), 134.25 (q, *J*_C,F_ = 1.3 Hz, CH-6″), 138.29 (q, *J*_C,F_ = 1.3 Hz, C-1″), 176.03 (CH_3_ **C**ON); ^19^F NMR (470 MHz, D_2_O, *T* = 100 °C): δ −57.73 (br s, 3F, CF_3_); IR (CH_3_OH): 3363, 1665, 1612, 1593, 1492, 1446, 1331, 1267, 1167, 1127, 1071, 1038, 705, 580 cm^–1^; HRMS (ESI): [M + Na]^+^ *m*/*z* calcd for C_21_H_28_O_11_F_3_NNa: 550.1507, found: 550.1502; [M + H]^+^ *m*/*z* calcd for C_21_H_29_O_11_NF_3_: 528.1687, found: 528.1684.

##### *N*-[4-*O*-(β-d-Galactopyranosyl)-β-d-glucopyranosyl]-*N*-(4-fluorophenyl)­acetamide (**3e**)

Following general procedure C, lactosylamide **2e** (0.290 g, 0.376 mmol) gave **3e** (0.098 g, 55%) as a white solid. The analytical sample was obtained by HPLC (A: H_2_O, B: CH_3_OH; 30% to 90% B in 30 min, followed by isocratic 90% B for 10 min). [α]_D_^20^ = +45.1 (*c* 0.2 in CH_3_OH); ^1^H NMR (500 MHz, DMSO-*d*_6_, *T* = 100 °C): δ 1.82 (s, 3H, CH_3_CON), 2.76 (td, *J*_2,1_ = *J*_2,3_ = 9.0, *J*_2,OH_ = 4.5 Hz, 1H, H-2), 3.17 (dd, *J*_4,5_ = 9.7, *J*_4,3_ = 8.8 Hz, 1H, H-4), 3.31–3.35 (m, 1H, H-3′), 3.38 (ddd, *J*_2′,3′_ = 9.2, *J*_2′,1′_ = 7.4, *J*_2′,OH_ = 3.2 Hz, 1H, H-2′), 3.41 (ddd, *J*_5,4_ = 9.6, *J*_5,6a_ = 4.7, *J*_5,6b_ = 2.7 Hz, 1H, H-5), 3.44 (ddd, *J*_5′,6a′_ = 6.7, *J*_5′,6b′_ = 5.6, *J*_5′,4′_ = 1.2 Hz, 1H, H-5′), 3.48 (td, *J*_3,2_ = *J*_3,4_ = 8.8, *J*_3,OH_ = 1.6 Hz, 1H, H-3), 3.53 (ddd, *J*_gem_ = 10.8, *J*_6′a,5′_ = 6.7, *J*_6′a,OH_ = 4.7 Hz, 1H, H-6′a), 3.57 (dt, *J*_gem_ = 10.6, *J*_6′b,5′_ = *J*_6′b,OH_ = 5.3 Hz, 1H, H-6′b), 3.63 (ddd, *J*_gem_ = 11.8, *J*_6a,5_ = 6.0, *J*_6a,OH_ = 4.9 Hz, 1H, H-6a), 3.68 (ddd, *J*_4′,OH_ = 3.8, *J*_4′,3′_ = 3.0, *J*_4′,5′_ = 0.8 Hz, 1H, H-4′), 3.79 (ddd, *J*_gem_ = 11.7, *J*_6b,OH_ = 5.0, *J*_6b,5_ = 2.5 Hz, 1H, H-6b), 4.06 (d, *J*_OH,4′_ = 4.0 Hz, 1H, OH-4′), 4.11 (t, *J*_OH,6_ = 6.0 Hz, 1H, OH-6), 4.20 (t, *J*_OH,6′_ = 5.4 Hz, 1H, OH-6′), 4.21 (d, *J*_1′,2′_ = 7.5 Hz, 1H, H-1′), 4.27 (d, *J*_OH,3′_ = 4.8 Hz, 1H, OH-3′), 4.48 (d, *J*_OH,3_ = 2.0 Hz, 1H, OH-3), 4.65 (d, *J*_OH,2′_ = 3.4 Hz, 1H, OH-2′), 4.71 (d, *J*_OH,2_ = 5.0 Hz, 1H, OH-2), 5.47 (br s, 1H, H-1), 7.19 (t, *J*_3″,2′′_ = *J*_3″,F_ = 8.8 Hz, 2H, H-3″), 7.40 (dd, *J*_2″,3′′_ = 8.8, *J*_2″,F_ = 5.2 Hz, 2H, H-2″); ^13^C NMR (126 MHz, DMSO-*d*_6_, *T* = 100 °C): δ 22.28 (**C**H_3_CON), 60.11 (CH_2_-6′), 60.48 (CH_2_-6), 67.89 (CH-4′), 69.39 (CH-2), 70.38 (CH-2′), 73.05 (CH-3′), 75.16 (CH-5′), 75.32 (CH-3), 76.64 (CH-5), 79.45 (CH-4), 82.36 (CH-1), 103.06 (CH-1′), 114.75 (d, *J*_C,F_ = 22.5 Hz, CH-3″), 131.83 (d, *J*_C,F_ = 8.6 Hz, CH-2″), 134.25 (C-1″), 161.11 (d, *J*_C,F_ = 233.8 Hz, CF-4″), 174.52 (CH_3_ **C**ON); ^19^F NMR (471 MHz, DMSO-*d*_6_, *T* = 100 °C): δ −114.21 (br s); IR (CH_3_OH): 3368, 3077, 2928, 2892, 1653, 1601, 1508, 1374, 1220, 1154, 1113, 1075, 1042, 849, 824, 757, 703, 647, 508 cm^–1^; HRMS (ESI): [M + Na]^+^ *m*/*z* calcd for C_20_H_28_O_11_NFNa: 500.1539, found: 500.1547.

##### *N*-[4-*O*-(β-d-Galactopyranosyl)-β-d-glucopyranosyl]-*N*-(3-fluorophenyl)­acetamide (**3f**)

Following general procedure C, lactosylamide **2f** (0.300 g, 0.389 mmol) gave **3f** (0.103 g, 89%) as a white solid. The analytical sample was obtained by HPLC (A: H_2_O, B: CH_3_OH; 30% to 90% B in 30 min, followed by isocratic 90% B for 10 min). [α]_D_^20^ = +40.0 (*c* 0.2 in CH_3_OH); ^1^H NMR (500 MHz, DMSO-*d*_6_, *T* = 100 °C): δ 1.86 (s, 3H, CH_3_CON), 2.79 (td, *J*_2,1_ = *J*_2,3_ = 9.2, *J*_2,OH_ = 4.2 Hz, 1H, H-2), 3.19 (dd, *J*_4,5_ = 9.6, *J*_4,3_ = 8.9 Hz, 1H, H-4), 3.30–3.40 (m, 2H, H-2′,3′), 3.42 (ddd, *J*_5,4_ = 9.8, *J*_5,6a_ = 4.8, *J*_5,6b_ = 2.7 Hz, 1H, H-5), 3.44 (ddd, *J*_5′,6a′_ = 6.7, *J*_5′,6b′_ = 5.5, *J*_5′,4′_ = 1.2 Hz, 1H, H-5′), 3.48 (t, *J*_3,2_ = *J*_3,4_ = 8.8 Hz, 1H, H-3), 3.53 (ddd, *J*_gem_ = 11.1, *J*_6′a,5′_ = 6.4, *J*_6′a,OH_ = 4.0 Hz, 1H, H-6′a), 3.57 (dt, *J*_gem_ = 11.0, *J*_6′b,5′_ = *J*_6′b,OH_ = 5.6 Hz, 1H, H-6′b), 3.64 (dt, *J*_gem_ = 11.8, *J*_6a,5_ = *J*_6a,OH_ = 5.2 Hz, 1H, H-6a), 3.67 (dd, *J*_4′,3′_ = 4.5, *J*_4′,5′_ = 2.0 Hz, 1H, H-4′), 3.79 (ddd, *J*_gem_ = 11.9, *J*_6b,OH_ = 4.8, *J*_6b,5_ = 2.6 Hz, 1H, H-6b), 4.06 (br s, 1H, OH-4′), 4.15 (t, *J*_OH,6_ = 5.7 Hz, 1H, OH-6), 4.20 (t, *J*_OH,6′_ = 5.3 Hz, 1H, H-6′), 4.22 (d, *J*_1′,2′_ = 7.5 Hz, 1H, H-1′), 4.26–4.29 (m, 1H, OH-3′), 4.48 (br s, 1H, OH-3), 4.64–4.70 (m, 1H, OH-2′), 4.76 (d, *J*_OH,2_ = 5.2 Hz, 1H, OH-2), 5.47 (d, *J*_1,2_ = 9.1 Hz, 1H, H-1), 7.17–7.26 (m, 3H, H-2″,4″,6″), 7.40–7.48 (m, 1H, H-5″); ^13^C NMR (126 MHz, DMSO-*d*_6_, *T* = 100 °C): δ 22.25 (**C**H_3_CON), 60.12 (CH_2_-6′), 60.43 (CH_2_-6), 67.90 (CH-4′), 69.42 (CH-2), 70.39 (CH-2′), 73.06 (CH-3′), 75.16 (CH-3), 75.33 (CH-5′), 76.69 (CH-5), 79.40 (CH-4), 82.29 (CH-1), 103.06 (CH-1′), 114.40 (d, *J*_C,F_ = 20.4 Hz, CH-4″), 116.98 (d, *J*_C,F_ = 21.9 Hz, CH-2″), 126.12 (d, *J*_C,F_ = 3.0 Hz, CH-6″), 129.40 (d, *J*_C,F_ = 9.3 Hz, CH-5″), 139.68 (d, *J*_C,F_ = 10.1 Hz, C-1″), 161.35 (d, *J*_C,F_ = 244.9 Hz, CF-3″), 169.78 (CH_3_ **C**ON); ^19^F NMR (471 MHz, DMSO-*d*_6_, *T* = 100 °C): δ −112.81 to −112.53 (m, 1F, F-3″); IR (CH_3_OH): 3369, 3074, 2928, 2889, 1662, 1607, 1592, 1487, 1373, 1118, 1074, 1041, 1005, 747, 699 cm^–1^; HRMS (ESI): [M + Na]^+^ *m*/*z* calcd for C_20_H_28_O_11_NFNa: 500.1539, found: 500.1541; [M + H]^+^ *m*/*z* calcd for C_20_H_29_O_11_NF: 478.1719, found: 478.1721.

##### *N*-[4-*O*-(β-d-Galactopyranosyl)-β-d-glucopyranosyl]-*N*-(2-fluorophenyl)­acetamide (**3g**)

Following general procedure C, lactosylamide **2g** (0.300 g, 0.389 mmol) gave **3g** (12.71 mg, 7%) as a white solid. The analytical sample was obtained by HPLC (A: H_2_O, B: CH_3_OH; 30% to 90% B in 30 min, followed by isocratic 90% B for 10 min). ^1^H NMR (500 MHz, D_2_O, *T* = 100 °C): δ 2.56 (s, 3H, CH_3_CON), 3.71 (br s, 1H, H-2), 4.11 (br s, 1H, H-4), 4.17 (dd, *J*_2′,3′_ = 9.9, *J*_2′,3′_ = 7.7 Hz, 1H, H-2′), 4.25 (dd, *J*_3′,2′_ = 9.8, *J*_3′,4′_ = 3.4 Hz, 1H, H-3′), 4.29 (ddd, *J*_5′,6′a_ = 7.4, *J*_5′,6′a_ = 4.7, *J*_5′,4′_ = 1.1 Hz, 1H, H-5′), 4.30–4.35 (m, 2H, H-3,5), 4.35 (dd, *J*_gem_ = 11.7, *J*_6′b,5_ = 4.6 Hz, 1H, H-6′b), 4.38 (dd, *J*_gem_ = 11.8, *J*_6′a,5_ = 7.3 Hz, 1H, H-6′a), 4.42 (s, 1H, H-6b), 4.54 (dd, *J*_4′,3′_ = 3.5, *J*_4′,5′_ = 1.1 Hz, 1H, H-4′), 4.56–4.64 (m, 1H, H-6a), 5.03 (d, *J*_1′,2′_ = 7.6 Hz, 1H, H-1′), 6.28 (br s, 1H, H-1), 7.79–8.04 (m, 2H, Ar-H), 8.05–8.27 (m, 2H, Ar-H); carbon shifts reported only for the major of the four present rotamers: ^13^C NMR (126 MHz, D_2_O, *T* = 25 °C): δ 21.62 (s, 3H, **C**H_3_CON), 60.77 (CH_2_-6), 61.77 (CH_2_-6′), 69.27 (CH-4′), 70.17 (CH-2), 71.63 (CH-2′), 73.17 (CH-3′), 76.05 (CH-5′), 76.37 (CH-3), 77.53 (CH-5), 78.19 (CH-4), 83.13 (CH-1), 103.49 (CH-1′), 117.48 (d, *J*_C,F_ = 20.4 Hz, CH-3″), 125.63 (d, *J*_C,F_ = 12.6 Hz, C-1″), 125.81 (d, *J*_C,F_ = 3.7 Hz, CH-6″), 131.60 (CH-5″), 132.19 (d, *J*_C,F_ = 2.7 Hz, CH-4″), 159.00 (d, *J*_C,F_ = 248.8 Hz, CF-2″), 176.71 (CH_3_ **C**ON); HRMS (ESI): [M + Na]^+^ *m*/*z* calcd for C_20_H_28_O_11_NFNa: 500.1539, found: 500.1535; [M + H]^+^ *m*/*z* calcd for C_20_H_29_O_11_NF: 478.1719, found: 478.1716.

##### *N*-[4-*O*-(β-d-Galactopyranosyl)-β-d-glucopyranosyl]-*N*-(3,4-difluorophenyl)­acetamide (**3h**)

Following general procedure C, lactosylamide **2h** (0.200 g, 0.253 mmol) gave **3h** (0.068 g, 54%) as a white solid. The analytical sample was obtained by HPLC (A: H_2_O, B: CH_3_OH; 30% to 90% B in 30 min, followed by isocratic 90% B for 10 min). [α]_D_^20^ = +33.5 (*c* 0.2 in CH_3_OH); ^1^H NMR (500 MHz, DMSO-*d*_6_, *T* = 100 °C): δ 1.88 (s, 3H, CH_3_CON), 2.75 (td, *J*_2,1_ = *J*_2,3_ = 9.1, *J*_2,OH_ = 2.9 Hz, 1H, H-2), 3.19 (dd, *J*_4,5_ = 9.7, *J*_5,3_ = 8.9 Hz, 1H, H-4), 3.33 (dd, *J*_3′,2′_ = 9.6, *J*_3′,4′_ = 3.0 Hz, 1H, H-3′), 3.37 (dd, *J*_2′,3′_ = 9.5, *J*_2′,1′_ = 7.4 Hz, 1H, H-2′), 3.41 (ddd, *J*_5,4_ = 9.6, *J*_5,6a_ = 4.7, *J*_5,6b_ = 2.7 Hz, 1H, H-5), 3.43 (ddd, *J*_5′,6′a_ = 6.7, *J*_5′,6′b_ = 5.6, *J*_5′,4′_ = 1.2 Hz, 1H, H-5′), 3.48 (t, *J*_3,2_ = *J*_3,4_ = 8.7 Hz, 1H, H-4), 3.47–3.55 (m, 1H, H-6′a), 3.54–3.59 (m, 1H, H-6′b), 3.64 (dt, *J*_gem_ = 11.5, *J*_6a,5_ = 4.6 Hz, 1H, H-6a), 3.66–3.68 (m, 1H, H-4′), 3.78 (dt, *J*_gem_ = 11.9, *J*_6b,5_ = 3.2 Hz, 1H, H-6b), 4.15 (br s, 1H, OH-4′), 4.22 (br s, 1H, OH-6), 4.22 (d, *J*_1′,2′_ = 7.4 Hz, 1H, H-1′), 4.28 (br s, 1H, OH-6′), 4.38 (br s, 1H, OH-3′), 4.53 (br s, 1H, OH-3), 4.73 (br s, 1H, OH-2′), 4.88 (br s, 1H, OH-2), 5.44 (br s, 1H, H-1), 7.23 (ddt, *J*_6″,5′′_ = 8.3, *J*_6″,F4′′_ = 3.9, *J*_6″,F3′′_ = 2.1 Hz, 1H, H-6″), 7.37–7.42 (m, 1H, H-2″), 7.42 (dt, *J*_5″,F4′′_ = 10.7, *J*_5″,6′′_ = *J*_5″,F3′′_ = 8.9 Hz, 1H, H-5″); ^13^C NMR (126 MHz, DMSO-*d*_6_, *T* = 100 °C): δ 22.30 (CH_3_CON), 60.10 (CH_2_-6′), 60.35 (CH_2_-6), 67.87 (CH-4′), 69.42 (CH-2), 70.42 (CH-2′), 73.09 (CH-3′), 75.20 (CH-3), 75.24 (CH-5′), 76.71 (CH-5), 79.35 (CH-4), 82.10 (CH-1), 103.12 (CH-1′), 116.51 (d, *J*_C,F_ = 17.7 Hz, CH-5″), 119.29 (d, *J*_C,F_ = 17.4 Hz, CH-2″), 127.10 (dd, *J*_C,F_ = 6.4, *J*_C,F_ = 3.2 Hz, CH-6″), 134.64 (dd, *J*_C,F_ = 7.8, *J*_C,F_ = 3.4 Hz, C-1″), 148.41 (dd, *J*_C,F_ = 247.1, *J*_C,F_ = 13.3 Hz, CF-4″), 148.86 (dd, *J*_C,F_ = 247.3, *J*_C,F_ = 12.2 Hz, CF-3″), 169.99 (CH_3_CON); ^19^F NMR (470 MHz, DMSO-*d*_6_, *T* = 100 °C): δ −139.02 (br s, 1F), −137.28 (br s, 1F); IR (CH_3_OH): 3363, 2924, 2889, 1659, 1609, 1516, 1433, 1375, 1114, 1076, 1041 cm^–1^; HRMS (ESI): [M + Na]^+^ *m*/*z* calcd for C_20_H_27_O_11_NF_2_Na: 518.1444, found: 518.1436; [M + H]^+^ *m*/*z* calcd for C_20_H_28_O_11_NF_2_: 496.1625, found: 496.1620.

##### *N*-[4-*O*-(β-d-Galactopyranosyl)-β-d-glucopyranosyl]-*N*-(3,4,5-trifluorophenyl)­acetamide (**3i**)

Following general procedure C, lactosylamide **2i** (0.255 g, 0.316 mmol) gave **3i** (0.125 g, 77%) as a white solid. The analytical sample was obtained by HPLC (A: H_2_O, B: CH_3_OH; 30% to 90% B in 30 min, followed by isocratic 90% B for 10 min). [α]_D_^20^ = +38.8 (*c* 0.2 in CH_3_OH); ^1^H NMR (500 MHz, DMSO-*d*_6_, *T* = 100 °C): δ 1.93 (s, 3H, CH_3_CON), 2.78 (td, *J*_2,1_ = *J*_2,3_ = 9.0, *J*_2,OH_ = 5.11 Hz, 1H, H-2), 3.21 (dd, *J*_4,5_ = 9.8, *J*_4,3_ = 8.8 Hz, 1H, H-4), 3.33 (ddd, *J*_3′,2′_ = 9.2, *J*_3′,4′_ = 4.8, *J*_3′,OH_ = 3.4 Hz, 1H, H-3′), 3.37 (ddd, *J*_2′,3′_ = 9.7, *J*_2′,1′_ = 7.3, *J*_2′,OH_ = 3.9 Hz, 1H, H-2′), 3.37 (ddd, *J*_5,4_ = 9.7, *J*_5,6a_ = 5.2, *J*_5,6b_ = 2.9 Hz, 1H, H-5), 3.44 (ddd, *J*_5′,6a′_ = 6.6, *J*_5′,6′b_ = 5.5, *J*_5′,4′_ = 1.2 Hz, 1H, H-5′), 3.48 (td, *J*_3,2_ = *J*_3,4_ = 8.7, *J*_3,OH_ = 1.7 Hz, 1H, H-3), 3.49–3.61 (m, 2H, H-6′), 3.65 (dt, *J*_gem_ = 12.0, *J*_6a,OH_ = 6.2, *J*_6a,5_ = 4.9 Hz, 1H, H-6a), 3.67 (td, *J*_4′,3′_ = *J*_4′,OH_ = 4.1, *J*_4′,5′_ = 1.3 Hz, 1H, H-4′), 3.79 (ddd, *J*_gem_ = 12.0, *J*_6b,OH_ = 6.0, *J*_6b,5_ = 2.7 Hz, 1H, H-6b), 4.11 (br s, 1H, OH-4′), 4.22 (d, *J*_1′,2′_ = 7.4 Hz, 1H, H-1′), 4.26 (br s, 2H, OH-6,6′), 4.33 (s, 1H, OH-3′), 4.52 (s, 1H, OH-3), 4.69 (s, 1H, OH-2′), 4.94 (s, 1H, OH-2), 5.41 (d, *J*_1,2_ = 9.1 Hz, 1H, H-1), 7.29 (dd, *J*_2″,F3′′_ = 8.8, *J*_2″,F4′′_ = 6.6 Hz, 2H, H-2″); ^13^C NMR (126 MHz, DMSO-*d*_6_, *T* = 100 °C): δ 22.40 (CH_3_CON), 60.37 (CH_2_-6′), 60.39 (CH_2_-6), 68.13 (CH-4′), 69.57 (CH-2), 70.62 (CH-2′), 73.24 (CH-3′), 75.36 (2 × C, CH-3, CH-5′), 76.89 (CH-5), 79.33 (CH-4), 82.73 (CH-1), 103.28 (CH-1′), 115.46 (dd, *J*_C,F_ = 22.0, *J*_C,F_ = 4.6 Hz, CH-2″), 133.96 (td, *J*_C,F_ = 10.4, *J*_C,F_ = 4.6 Hz, C-1″), 138.79 (dt, *J*_C,F_ = 251.2, *J*_C,F_ = 15.9 Hz, CF-4″), 149.55 (ddd, *J*_C,F_ = 247.9, *J*_C,F_ = 10.0, *J*_C,F_ = 4.9 Hz, CF-3″), 170.46 (CH_3_ **C**ON); ^19^F NMR (470 MHz, DMSO-*d*_6_, *T* = 100 °C): δ −161.92 (br s, 1F, F-4″), −135.22 (br s, 2F, F-3″); IR (KBr): 3399, 3090, 2930, 1661, 1623, 1594, 1532, 1443, 1411, 1375, 1330, 1238, 1167, 1115, 1076, 1044, 1017, 995, 829, 783, 703, 662, 599, 576, 513 cm^–1^; HRMS (ESI): [M + Na]^+^ *m*/*z* calcd for C_20_H_26_O_11_NF_3_Na: 536.1350, found: 536.1351; [M + H]^+^ *m*/*z* calcd for C_20_H_27_O_11_NF_3_: 514.1531, found: 514.1532.

##### *N*-[4-*O*-(β-d-Galactopyranosyl)-β-d-glucopyranosyl]-*N*-(4-chlorophenyl)­acetamide (**3j**)

Following general procedure C, lactosylamide **2j** (0.213 g, 0.389 mmol) gave **3j** (0.131 g, 98%) as a white solid. The analytical sample was obtained by HPLC (A: H_2_O, B: CH_3_OH; 30% to 90% B in 30 min, followed by isocratic 90% B for 10 min). [α]_D_^20^ = +35.6 (*c* 0.2 in H_2_O); ^1^H NMR (500 MHz, D_2_O, *T* = 25 °C): δ 1.90 (s, 3H, CH_3_CON), 3.04 (t, *J*_2,3_ = *J*_2,1_ = 9.3 Hz, 1H, H-2), 3.48 (t, *J*_4,5_ = *J*_4,3_ = 9.4 Hz, 1H, H-4), 3.52 (dd, *J*_2′,3′_ = 10.0, *J*_2′,1′_ 7.8 Hz, 1H, H-2′), 3.63 (dd, *J*_3′,2′_ = 10.0, *J*_3′,4′_ = 3.4 Hz, 1H, H-3′), 3.63–3.73 (m, 2H, H-5,5′), 3.66–3.74 (m, 2H, H-3,6′b), 3.74 (dd, *J*_gem_ = 12.8, *J*_6′a,5′_ = 9.2 Hz, 1H, H-6′a), 3.80 (dd, *J*_gem_ = 12.3, *J*_6a,5_ = 5.0 Hz, 1H, H-6a), 3.89 (dd, *J*_4′,3′_ = 3.5, *J*_4′,5′_ = 0.8 Hz, 1H, H-4′), 3.97 (dd, *J*_gem_ = 12.3, *J*_6b,5_ = 1.9 Hz, 1H, H-6′a), 4.39 (d, *J*_1′,2′_ = 7.8 Hz, 1H, H-1′), 5.68 (d, *J*_1,2_ = 9.5 Hz, 1H, H-1), 7.40 (d, *J*_2″,3′′_ = 8.8 Hz, 2H, H-2″), 7.53 (d, *J*_3″,2′′_ = 8.8 Hz, 2H, H-3″); ^13^C NMR (126 MHz, D_2_O, *T* = 25 °C): δ 23.48 (**C**H_3_CON), 60.78 (CH_2_-6), 61.78 (CH_2_-6′), 69.27 (CH-4′), 69.87 (CH-2), 71.63 (CH-2′), 73.17 (CH-3′), 76.05 (CH-5′), 76.28 (CH-3), 77.55 (CH-5), 78.31­(CH-4), 82.55 (CH-1), 103.51­(CH-1′), 130.26 (CH-3″), 131.95 (CH-2″), 135.42 (C-4′), 136.36 (C-1′), 176.61 (CH_3_ **C**ON); IR (CHCl_3_): 3362, 2920, 2850, 1662, 1601, 1592, 1492, 1428, 1373, 1115, 1076, 1040, 1018, 729 cm^–1^; HRMS (ESI): [M + Na]^+^ *m*/*z* calcd for C_20_H_28_O_11_NClNa: 516.1249, found: 516.1242; [M + H]^+^ *m*/*z* calcd for C_20_H_29_O_11_NCl: 494.1429, found: 494.1423.

##### *N*-[4-*O*-(β-d-Galactopyranosyl)-β-d-glucopyranosyl]-*N*-(3,4-dichlorophenyl)­acetamide (**3k**)

Following general procedure D, lactosylamide **2k** (0.411 g, 0.5 mmol) gave **3k** (0.174 g, 66%) as a white foam. The analytical sample was obtained by HPLC (A: H_2_O, B: CH_3_OH; 30% to 90% B in 30 min, followed by isocratic 90% B for 10 min). [α]_D_^20^ = +47.2 (*c* 0.2 in CH_3_OH); ^1^H NMR (500 MHz, D_2_O, *T* = 100 °C): δ 2.02 (s, 3H, CH_3_CON), 3.08 (dd, *J*_2,1_ = 9.5, *J*_2,3_ = 9.0 Hz, 1H, H-2), 3.46 (dd, *J*_4,5_ = 9.8, *J*_4,3_ = 8.9 Hz, 1H, H-4), 3.56 (dd, *J*_2′,3′_ = 9.8, *J*_2′,3′_ = 7.8 Hz, 1H, H-2′), 3.64 (dd, *J*_3′,2′_ = 9.8, *J*_3′,4′_ = 3.4 Hz, 1H, H-3′), 3.67 (ddd, *J*_5′,6′a_ = 7.4, *J*_5′,6′a_ = 4.7, *J*_5′,4′_ = 1.1 Hz, 1H, H-5′), 3.71 (ddd, *J*_5,4_ = 9.8, *J*_5,6a_ = 5.2, *J*_5,6b_ = 2.4 Hz, 1H, H-5), 3.68–3.76 (m, 2H, H-3,6′b), 3.77 (dd, *J*_gem_ = 11.8, *J*_6′a,5′_ = 7.4 Hz, 1H, H-6′a), 3.80 (dd, *J*_gem_ = 12.3, *J*_6a,5_ = 5.2 Hz, 1H, H-6a), 3.93 (dd, *J*_4′,3′_ = 3.4, *J*_4′,5′_ = 1.1 Hz, 1H, H-4′), 3.98 (dd, *J*_gem_ = 12.3, *J*_6b,5_ = 2.4 Hz, 1H, H-6b), 4.41 (d, *J*_1′,2′_ = 7.8 Hz, 1H, H-1′), 5.55 (br s, 1H, H-1), 7.35 (dd, *J*_6″,5′′_ = 8.5, *J*_6″,2′′_ = 2.4 Hz, 1H, H-6″), 7.61 (d, *J*_2″,6′′_ = 2.4 Hz, 1H, H-2″), 7.67 (d, *J*_5″,6′′_ = 8.5 Hz, 1H, H-5″); ^13^C NMR (126 MHz, D_2_O, *T* = 100 °C): δ 22.79 (**C**H_3_CON), 61.16 (CH_2_-6), 61.48 (CH_2_-6′), 69.21 (CH-4′), 70.07 (CH-2), 71.59 (CH-2′), 73.32 (CH-3′), 75.81 (CH-5′), 76.07 (CH-3), 77.41 (CH-5), 78.97 (CH-4), 83.50 (CH-1), 103.39 (CH-1′), 130.29 (CH-6″), 131.43 (CH-5″), 132.14 (CH-2″), 132.61 (CCl-4″), 133.10 (CCl-3″), 137.30 (C-1″), 175.92 (CH_3_ **C**ON); IR (CH_3_OH): 3365, 1666, 1585, 1560, 1398, 1163, 1127, 1075, 1034, 824, 682, 437 cm^–1^; HRMS (ESI): [M + Na]^+^ *m*/*z* calcd for C_20_H_27_O_11_NCl_2_Na: 550.0853, found: 550.0850; [M + H]^+^ *m*/*z* calcd for C_20_H_28_O_11_NCl_2_: 528.1034, found: 528.1031.

##### *N*-[4-*O*-(β-d-Galactopyranosyl)-β-d-glucopyranosyl]-*N*-(4-iodophenyl)­acetamide (**3l**)

Following general procedure C, lactosylamide **2l** (0.200 g, 0.227 mmol) gave **3l** (0.094 g, 71%) as a white solid. The analytical sample was obtained by HPLC (A: H_2_O, B: CH_3_OH; 30% to 90% B in 30 min, followed by isocratic 90% B for 10 min). [α]_D_^20^ = +46.2 (*c* 0.2 in CH_3_OH); ^1^H NMR (500 MHz, DMSO-*d*_6_, *T* = 100 °C): δ 1.84 (s, 3H, CH_3_CON), 2.75 (t, *J*_2,1_ = *J*_2,3_ = 9.0 Hz, 1H, H-2), 3.18 (dd, *J*_4,5_ = 9.7, *J*_4,3_ = 8.8 Hz, 1H, H-4), 3.33 (dd, *J*_3′,2′_ = 9.5, *J*_3′,4′_ = 3.4 Hz, 1H, H-3′), 3.38 (dd, *J*_2′,3′_ = 9.5, *J*_2′,1′_ = 7.4 Hz, 1H, H-2′), 3.41 (ddd, *J*_5,4_ = 9.1, *J*_5,6a_ = 4.4, *J*_5,6b_ = 1.6 Hz, 1H, H-5), 3.44 (ddd, *J*_5′,6a′_ = 6.7, *J*_5′,6b′_ = 5.6, *J*_5′,4′_ = 1.3 Hz, 1H, H-5′), 3.47 (t, *J*_3,2_ = *J*_3,4_ = 8.7 Hz, 1H, H-3), 3.52 (dd, *J*_gem_ = 10.9, *J*_6′a,5′_ = 6.3 Hz, 1H, H-6′a), 3.58 (dd, *J*_gem_ = 10.8, *J*_6′b,5′_ = 5.5 Hz, 1H, H-6′b), 3.63 (dd, *J*_gem_ = 11.6, *J*_6a,5_ = 4.8 Hz, 1H, H-6a), 3.68 (dd, *J*_4′,3′_ = 3.4, *J*_4′,5′_ = 0.9 Hz, 1H, H-4′), 3.78 (dd, *J*_gem_ = 10.7, *J*_6b,5_ = 1.7 Hz, 1H, H-6b), 4.07 (br s, 1H, OH-4′), 4.11 (br s, 1H, OH-6), 4.20 (br s, 1H, OH-6′), 4.22 (d, *J*_1′,2′_ = 7.4 Hz, 1H, H-1′), 4.27 (br s, 1H, OH-3′), 4.47 (br s, 1H, OH-3), 4.65 (br s, 1H, OH-2′), 4.72 (br s, 1H, OH-2), 5.46 (d, *J*_1,2_ = 8.5 Hz, 1H, H-1′), 7.16 (d, *J*_2″,3′′_ = 8.5 Hz, 1H, H-2″), 7.75 (d, *J*_3″,2′′_ = 8.5 Hz, 1H, H-3″); ^13^C NMR (126 MHz, DMSO-*d*_6_, *T* = 100 °C): δ 22.29 (**C**H_3_CON), 60.11 (CH_2_-6′), 60.44 (CH_2_-6), 67.89 (CH-4′), 69.41 (CH-2), 70.38 (CH-2′), 73.05 (CH-3′), 75.16 (CH-5′), 75.29 (CH-3), 76.66 (CH-5), 79.37 (CH-4), 81.37 (CH-1), 93.09 (C-4″), 103.06 (CH-1′), 132.14 (CH-2″), 137.08 (CH-3″), 137.93 (C-1″), 169.73 (CH_3_ **C**ON); IR (CH_3_OH): 3364, 2926, 2888, 1659, 1582, 1484, 1393, 1372, 1116, 1074, 1060, 1039, 1010, 571, 406 cm^–1^; HRMS (ESI): [M + Na]^+^ *m*/*z* calcd for C_20_H_28_O_11_INNa: 608.0599, found: 608.0596; [M + H]^+^ *m*/*z* calcd for C_20_H_29_O_11_NI: 586.0780, found: 586.0775.

##### *N*-[4-*O*-(β-d-Galactopyranosyl)-β-d-glucopyranosyl]-*N*-(4-methoxyphenyl)­acetamide (**3m**)

Following general procedure C, lactosylamide **2m** (0.300 g, 0.383 mmol) gave **3m** (0.100 g, 54%) as a white foam. The analytical sample was obtained by HPLC (A: H_2_O, B: CH_3_OH; 30% to 90% B in 30 min, followed by isocratic 90% B for 10 min). [α]_D_^20^ = +37.2 (*c* 0.3 in CH_3_OH); ^1^H NMR (500 MHz, D_2_O, *T* = 100 °C): δ 1.92 (br s, 3H, CH_3_CON), 3.09 (t, *J*_2,1_ = *J*_2,3_ = 9.0 Hz, 1H, H-2), 3.44 (dd, *J*_4,5_ = 9.8, *J*_4,3_ = 9.0 Hz, 1H, H-4), 3.55 (dd, *J*_2′,3′_ = 9.9, *J*_2′,1′_ = 7.7 Hz, 1H, H-2′), 3.63 (dd, *J*_3′,2′_ = 9.9, *J*_3′,4′_ = 3.4 Hz, 1H, H-3′), 3.67 (ddd, *J*_5′,6a′_ = 7.3, *J*_5′,6b′_ = 4.7, *J*_5′,4′_ = 1.1 Hz, 1H, H-5′), 3.68–3.71 (m, 1H, H-5), 3.73 (t, *J*_3,2_ = *J*_3,4_ = 9.0 Hz, 1H, H-3), 3.74 (dd, *J*_gem_ = 11.8, *J*_6′b,5′_ = 4.7 Hz, 1H, H-6b), 3.76 (dd, *J*_gem_ = 11.8, *J*_6′a,5′_ = 7.3 Hz, 1H, H-6′a), 3.79 (dd, *J*_gem_ = 12.3, *J*_6a,5_ = 5.2 Hz, 1H, H-6a), 3.88 (s, 3H, Ar-OCH_3_), 3.92 (dd, *J*_4′,3′_ = 3.4, *J*_4′,5′_ = 1.1 Hz, 1H, H-4′), 3.97 (dd, *J*_gem_ = 12.3, *J*_6b,5_ = 2.4 Hz, 1H, H-6b), 4.40 (d, *J*_1′,2′_ = 7.7 Hz, 1H, H-1′), 5.61 (br s, 1H, H-1), 7.09 (d, *J*_2″,3′′_ = 8.4 Hz, 2H, H-2″), 7.34 (d, *J*_3″,2′′_ = 8.4 Hz, 2H, H-3″); ^13^C NMR (126 MHz, D_2_O, *T* = 100 °C): δ 25.05 (**C**H_3_CON), 58.60 (Ar-O**C**H_3_), 63.36 (CH_2_-6), 63.61 (CH_2_-6′), 71.34 (CH-4′), 72.27 (CH-2), 73.73 (CH-2′), 75.46 (CH-3′), 77.94 (CH-5′), 78.27 (CH-3), 79.45 (CH-5), 81.16 (CH-4), 84.75 (CH-1), 105.53 (CH-1′), 117.39 (CH-2″), 133.69 (CH-3″), 162.01 (C-4″), 178.84 (CH_3_ **C**ON); IR (CH_3_OH): 3367, 2839, 1653, 1608, 1583, 1511, 1443, 1373, 1249, 1075, 1050, 1033, 719 cm^–1^; HRMS (ESI): [M + Na]^+^ *m*/*z* calcd for C_21_H_31_O_12_NNa: 512.1739, found: 512.1735; [M + H]^+^ *m*/*z* calcd for C_21_H_32_O_12_N: 490.1919, found: 490.1918.

##### *N*-[4-*O*-(β-d-Galactopyranosyl)-β-d-glucopyranosyl]-*N*-(3,4-dimethoxyphenyl)­acetamide (**3n**)

Following general procedure C, lactosylamide **2n** (0.137 g, 0.168 mmol) gave **3n** (0.076 g, 87%) as a white foam. The analytical sample was obtained by HPLC (A: H_2_O, B: CH_3_OH; 30% to 90% B in 30 min, followed by isocratic 90% B for 10 min). [α]_D_^20^ = +47.0 (*c* 0.2 in CH_3_OH); ^1^H NMR (500 MHz, DMSO-*d*_6_, *T* = 100 °C): δ 1.81 (s, 3H, CH_3_CON), 2.82 (td, *J*_2,1_ = *J*_2,3_ = 9.1, *J*_2,OH_ = 2.0 Hz, 1H, H-2), 3.17 (dd, *J*_4,5_ = 9.6, *J*_4,3_ = 8.9 Hz, 1H, H-4), 3.31–3.38 (m, 2H, H-2′,3′), 3.40 (ddd, *J*_5,3_ = 9.8, *J*_5,6a_ = 4.7, *J*_5,6b_ = 2.9 Hz, 1H, H-5), 3.44 (ddd, *J*_5′,6a′_ = 6.7, *J*_5′,6b′_ = 5.6, *J*_5′,4′_ = 1.2 Hz, 1H, H-5′), 3.47 (t, *J*_3,2_ = *J*_3,4_ = 8.8 Hz, 1H, H-3), 3.50–3.61 (m, 2H, H-6′), 3.61–3.66 (m, 1H, H-6a), 3.68 (ddd, *J*_4′,3′_ = 3.0, *J*_4′,OH_ = 1.6, *J*_4′,5′_ = 0.9 Hz, 1H, H-4′), 3.78 (s, 12H), 3.79–3.80 (m, 1H, H-6b), 3.81 (s, 3H, OCH_3_), 4.04–4.12 (m, 2H, OH), 4.19–4.21 (m, 1H, OH), 4.22 (d, *J*_1′,2′_ = 7.5 Hz, 1H, H-1′), 4.25–4.29 (m, 1H, OH), 4.48 (s, 1H, OH), 4.65–4.68 (m, 2H, OH-2,2′), 5.50 (br s, 1H, H-1), 6.92 (dd, *J*_6″,5′′_ = 8.5, *J*_6″,2′′_ = 2.2 Hz, 1H, H-6″), 6.95 (d, *J*_5″,6′′_ = 8.5 Hz, 1H, H-5″), 6.97 (d, *J*_2″,6′′_ = 2.1 Hz, 1H, H-2″); ^13^C NMR (126 MHz, DMSO-*d*_6_, *T* = 100 °C): δ 22.31 (**C**H_3_CON), 55.56 (2C, OCH_3_), 60.12 (CH_2_-6′), 60.57 (CH_2_-6), 67.90 (CH-4′), 69.37 (CH-2), 70.40 (CH-2′), 73.06 (CH-3′), 75.16 (CH-3), 75.39 (CH-5′), 76.48 (CH-5), 79.55 (CH-4), 80.94 (CH-1), 103.08 (CH-1′), 111.80 (CH-5″), 114.83 (CH-2″), 122.23 (CH-6″), 131.03 (C-1″), 148.46, 148.52 (C-3″,4″), 170.26 (CH_3_ **C**ON); IR (CH_3_OH): 3371, 2932, 2841, 1654, 1595, 1512, 1465, 1452, 1444, 1373, 1266, 1239, 1076, 1027, 877, 747, 702, 558 cm^–1^; HRMS (ESI): [M + Na]^+^ *m*/*z* calcd for C_22_H_33_O_13_NNa: 542.1844, found: 542.1852.

##### *N*-[4-*O*-(β-d-Galactopyranosyl)-β-d-glucopyranosyl]-*N*-(3,4,5-trimethoxyphenyl)­acetamide (**3o**)

Following general procedure C, lactosylamide **2o** (0.329 g, 0.390 mmol) gave **3o** (0.131 g, 61%) as a white foam. The analytical sample was obtained by HPLC (A: H_2_O, B: CH_3_OH; 30% to 90% B in 30 min, followed by isocratic 90% B for 10 min). [α]_D_^20^ = +49.4 (*c* 0.2 in CH_3_OH); ^1^H NMR (500 MHz, D_2_O, *T* = 25 °C): δ 1.97 (s, 3H, CH_3_CON), 3.14 (t, *J*_2,3_ = *J*_2,1_ = 9.2 Hz, 1H, H-2), 3.47 (t, *J*_4,5_ = *J*_4,3_ = 9.4 Hz, 1H, H-4), 3.53 (dd, *J*_2′,3′_ = 9.9, *J*_2′,1′_ = 7.8 Hz, 1H, H-2′), 3.64 (dd, *J*_3′,2′_ = 10.0, *J*_3′,4′_ = 3.4 Hz, 1H, H-3′), 3.67 (ddd, *J*_5′,6′a_ = 7.9, *J*_5′,6′b_ = 3.8, *J*_5′,4′_ = 0.9 Hz, 1H, H-5′), 3.67–3.75 (m, 2H, H-3,5), 3.71 (dd, *J*_gem_ = 11.8, *J*_6′b,5′_ = 3.9 Hz, 1H, H-6′b), 3.75 (dd, *J*_gem_ = 11.8, *J*_6′a,5′_ = 8.2 Hz, 1H, H-6′a), 3.80 (dd, *J*_gem_ = 12.4, *J*_6a,5_ = 5.1 Hz, 1H, H-6a), 3.82 (s, 3H, OCH_3_-4″), 3.85–3.88 (m, 6H, OCH_3_-3″), 3.90 (dd, *J*_4′,3′_ = 3.4, *J*_4′,5′_ = 0.9 Hz, 1H, H-4′), 3.99 (dd, *J*_gem_ = 12.2, *J*_6b,5_ = 2.0 Hz, 1H, H-6b), 4.39 (d, *J*_1′,2′_ = 7.8 Hz, 1H, H-1′), 5.67 (d, *J*_1,2_ = 9.5 Hz, 1H, H-1), 6.81 (s, 2H, H-2″); ^13^C NMR (126 MHz, D_2_O, *T* = 25 °C): δ 23.15 (**C**H_3_CON), 56.96 (OCH_3_-3″), 60.84 (CH_2_-6), 61.72 (OCH_3_-4″), 61.74 (CH_2_-6″), 69.25 (CH-4′), 70.04 (CH-2), 71.62 (CH-2′), 73.18 (CH-3′), 76.05 (CH-5′), 76.29 (CH-3), 77.39 (CH-5), 78.55 (CH-4), 82.72 (CH-1), 103.55 (CH-1′), 108.45 (CH-3″), 134.18 (C-1″), 138.05 (C-4″), 153.40 (C-3″), 176.82 (CH_3_ **C**ON); IR (CH_3_OH): 3370, 2934, 2883, 2841, 1658, 1594, 1504, 1454, 1417, 1372, 1232, 1125, 1075, 1050, 1036, 904, 874, 711, 524 cm^–1^; HRMS (ESI): [M + H]^+^ *m*/*z* calcd for C_23_H_36_O_14_N: 550.2130, found: 550.2138.

##### *N*-[4-*O*-(β-d-Galactopyranosyl)-β-d-glucopyranosyl]-*N*-(1,3-benzodioxol-5-yl)­acetamide (**3p**)

Following general procedure C, crude lactosylamide **2p** (∼0.5 mmol) gave **3p** (0.052 g, 21%) as a white foam. The analytical sample was obtained by HPLC (A: H_2_O, B: CH_3_OH; 30% to 90% B in 30 min, followed by isocratic 90% B for 10 min). [α]_D_^20^ = +44.8 (*c* 0.2 in CH_3_OH); ^1^H NMR (500 MHz, D_2_O, *T* = 100 °C): δ 1.97 (s, 3H, CH_3_CON), 3.15 (t, *J*_2,1_ = *J*_2,3_ = 9.2 Hz, 1H, H-2), 3.46 (dd, *J*_4,5_ = 9.8, *J*_4,3_ = 8.9 Hz, 1H, H-4), 3.56 (dd, *J*_2′,3′_ = 9.9, *J*_2′,3′_ = 7.7 Hz, 1H, H-2′), 3.64 (dd, *J*_3′,2′_ = 9.9, *J*_3′,4′_ = 3.4 Hz, 1H, H-3′), 3.67 (ddd, *J*_5′,6′a_ = 7.3, *J*_5′,6′a_ = 4.7, *J*_5′,4′_ = 1.2 Hz, 1H, H-5′), 3.67–3.77 (m, 3H, H-3,5,6′b), 3.77 (dd, *J*_gem_ = 11.6, *J*_6′a,5_ = 7.3 Hz, 1H, H-6′b), 3.79 (dd, *J*_gem_ = 12.3, *J*_6a,5_ = 5.2 Hz, 1H, H-6a), 3.93 (dd, *J*_4′,3′_ = 3.4, *J*_4′,5′_ = 1.1 Hz, 1H, H-4′), 3.97 (dd, *J*_gem_ = 12.3, *J*_6b,5_ = 2.4 Hz, 1H, H-6b), 4.41 (d, *J*_1′,2′_ = 7.7 Hz, 1H, H-1′), 5.58 (br s, 1H, H-1), 6.05 (s, 2H, CH_2_), 6.91 (dd, *J*_6″,5′′_ = 8.2, *J*_6″,2′′_ = 2.1 Hz, 1H, H-6″), 6.94 (d, *J*_2″,6′′_ = 2.1 Hz, 1H, H-2″), 6.98 (d, *J*_5″,6′′_ = 8.2 Hz, 1H, H-5″); ^13^C NMR (126 MHz, D_2_O, *T* = 100 °C): δ 22.72 (**C**H_3_CON), 61.20 (CH_2_-6), 61.48 (CH_2_-6′), 69.21 (CH-4′), 70.10 (CH-2), 71.59 (CH-2′), 73.33 (CH-3′), 75.81 (CH-5′), 76.15 (CH-3), 77.31 (CH-5), 79.02 (CH-4), 82.16 (CH-1), 102.41 (O**C**H_2_O), 103.39 (CH-1′), 108.76 (CH-5″), 111.07 (CH-2″), 124.19 (CH-6″), 131.45 (C-1″), 148.02 (C-3″,4″), 176.69 (CH_3_ **C**ON); IR (CH_3_OH): 3369, 1655, 1609, 1503, 1487, 1446, 1247, 1219, 1155, 1074, 1035, 1035, 931, 760 cm^–1^; HRMS (ESI): [M + Na]^+^ *m*/*z* calcd for C_21_H_29_O_13_NNa: 526.1531, found: 526.1527; [M + H]^+^ *m*/*z* calcd for C_21_H_30_O_13_N: 504.1712, found: 504.1709.

##### *N*-[4-*O*-(β-d-Galactopyranosyl)-β-d-glucopyranosyl]-*N*-[2-(ethylcarboxy)­phenyl]­acetamide (**3q**)

Following general procedure D, lactosylamide **2q** (0.081 g, 0.098 mmol) gave **3q** (0.042 g, 81%) as a white waxy solid. The analytical sample was obtained by HPLC (A: H_2_O, B: CH_3_OH; 30% to 90% B in 30 min, followed by isocratic 90% B for 10 min). [α]_D_^20^ = +5.9 (*c* 0.2 in H_2_O); ^1^H NMR (500 MHz, D_2_O, *T* = 100 °C): δ 1.32 (t, *J*_CH_3_,CH_2__ = 7.1 Hz, 3H, OCH_2_C**H**_**3**_), 1.95 (s, 3H, CH_3_CON), 2.91 (t, *J*_2,3_ = *J*_2,1_ = 9.2 Hz, 1H, H-2), 3.34 (t, *J*_4,5_ = *J*_4,3_ = 9.4 Hz, 1H, H-4), 3.53 (dd, *J*_2′,3′_ = 9.9, *J*_2′,3′_ = 7.7 Hz, 1H, H-2′), 3.61 (dd, *J*_3′,2′_ = 9.9, *J*_3′,4′_ = 3.4 Hz, 1H, H-3′), 3.60–3.64 (m, 1H, H-5), 3.64 (ddd, *J*_5′,6′a_ = 7.1, *J*_5′,6′a_ = 4.8, *J*_5′,4′_ = 1.2 Hz, 1H, H-5′), 3.69–3.76 (m, 4H, H-6′,6a,3), 3.83 (dd, *J*_gem_ = 12.6, *J*_6b,5_ = 2.5 Hz, 1H, H-6b), 3.91 (dd, *J*_4′,3′_ = 3.4, *J*_4′,5′_ = 1.2 Hz, 1H, H-4′), 4.27–4.38 (m, 2H, OC**H**_**2**_CH_3_), 4.36 (d, *J*_1′,2′_ = 7.8 Hz, 1H, H-1′), 5.75 (d, *J*_1,2_ = 9.4 Hz, 1H, H-1), 7.54 (d, *J*_6″,5′′_ = 7.8 Hz, 1H, H-6″), 7.66 (t, *J*_4″,5′′_ = *J*_4″,3′′_ = 7.4 Hz, 1H, H-4″), 7.75 (td, *J*_5″,6′′_ = *J*_5″,4′′_ = 7.7, *J*_5″,3′′_ = 1.5 Hz, 1H, H-5″), 7.85 (dd, *J*_3″,4′′_ = 7.7, *J*_3″,5′′_ = 1.5 Hz, 1H, H-3″); ^13^C NMR (126 MHz, D_2_O, *T* = 100 °C): δ 13.46 (OCH_2_ **C**H_3_), 23.32 (**C**H_3_CON), 61.14 (CH_2_-6), 61.45 (CH_2_-6′), 63.24 (O**C**H_2_CH_3_), 69.20 (CH-4′), 70.27 (CH-2), 71.57 (CH-2′), 73.30 (CH-3′), 75.77 (CH-5′), 75.90 (CH-3), 77.34 (CH-5), 78.64 (CH-4), 82.10 (CH-1), 103.37 (CH-1′), 130.35 (CH-4″), 130.83 (CH-3″), 132.22 (CH-6″), 133.38 (C-2″), 133.51 (CH-5″), 135.16 (C-1″), 170.05 (COOEt), 176.08 (CH_3_ **C**ON); IR (CH_3_OH): 3362, 2980, 2924, 2851, 1708, 1664, 1599, 1489, 1452, 1393, 1368, 1299, 1260, 1162, 1074, 1039, 759, 715 cm^–1^; HRMS (ESI): [M + Na]^+^ *m*/*z* calcd for C_23_H_33_O_13_NNa: 554.1850, found: 554.1840; [M + H]^+^ *m*/*z* calcd for C_23_H_34_O_13_N: 532.2030, found: 532.2023.

##### *N*-[4-*O*-(β-d-Galactopyranosyl)-β-d-glucopyranosyl]-*N*-(3-carboxyphenyl)­acetamide (**3r**)

Following general procedure C, lactosylamide **2r** (0.164 g, 0.206 mmol) gave **3r** (0.088 g, 85%) as a white solid. The analytical sample was obtained by HPLC (A: H_2_O, B: CH_3_OH; 30% to 90% B in 30 min, followed by isocratic 90% B for 10 min). [α]_D_^20^ = +38.5 (*c* 0.1 in H_2_O); ^1^H NMR (500 MHz, D_2_O, *T* = 25 °C): δ 1.90 (s, 3H, CH_3_CON), 3.03 (t, *J*_2,3_ = *J*_2,1_ = 9.4 Hz, 1H, H-2), 3.52 (dd, *J*_2′,3′_ = 10.0, *J*_2′,1′_ 7.8 Hz, 1H, H-2′), 3.44–3.56 (m, 1H, H-4), 3.63 (dd, *J*_3′,2′_ = 10.0, *J*_3′,4′_ = 3.4 Hz, 1H, H-3′), 3.66 (ddd, *J*_5′,6′a_ = 8.2, *J*_5′,6′b_ = 3.9, *J*_5′,4′_ = 0.9 Hz, 1H, H-5′), 3.68–3.74 (m, 3H, H-3,5,6′a), 3.73 (dd, *J*_gem_ = 11.8, *J*_6′a,5′_ = 8.1 Hz, 1H, H-6′a), 3.78–3.87 (m, 1H, H-6a), 3.89 (dd, *J*_4′,3′_ = 3.4, *J*_4′,5′_ = 1.0 Hz, 1H, H-4′), 3.99 (dd, *J*_gem_ = 12.4, *J*_6b,5_ = 2.2 Hz, 1H), 4.38 (d, *J*_1′,2′_ = 7.8 Hz, 1H, H-1′), 5.71 (d, *J*_1,2_ = 9.6 Hz, 1H, H-1), 7.54 (dt, *J*_6″,5′′_ = 7.9, *J*_6″,2′′_ = *J*_6″,4′′_ = 1.8 Hz, 1H, H-6″), 7.58 (t, *J*_5″,6′′_ = *J*_5″,4′′_ = 7.6 Hz, 1H, H-5″), 7.84 (dd, *J*_2″,6′′_ = 2.4, *J*_2″,4′′_ = 1.7 Hz, 1H, H-2″), 7.96 (dd, *J*_4″,5′′_ = 7.4, *J*_4″,2′′_ = 2.8 Hz, 1H, H-4″); ^13^C NMR (126 MHz, D_2_O, *T* = 25 °C): δ 23.55 (**C**H_3_CON), 60.79 (CH_2_-6), 61.73 (CH_2_-6′), 69.27 (CH-4′), 69.88 (CH-2), 71.64 (CH-2′), 73.17 (CH-3′), 76.00 (CH-5′), 76.32 (CH-3), 77.58 (CH-5), 78.18 (CH-4), 82.57 (CH-1), 103.49 (CH-1′), 129.88 (CH-5″), 130.31 (CH-4″), 130.75 (CH-2″), 133.20 (CH-6″), 137.68 (C-1″), 138.12 (C-3″), 174.94 (COOH), 176.75 (CH_3_ **C**ON); IR (CH_3_OH): 3346, 2924, 2895, 2852, 1661, 1608, 1598, 1567, 1434, 1383, 1164, 1078, 1039, 786, 709 cm^–1^; HRMS (ESI): [M + Na]^+^ *m*/*z* calcd for C_28_H_39_O_18_NNa: 526.1537, found: 526.1528; [M + H]^+^ *m*/*z* calcd for C_28_H_40_O_18_N: 504.1717, found: 504.1709.

##### *N*-[4-*O*-(β-d-Galactopyranosyl)-β-d-glucopyranosyl]-*N*-(3-carboxyphenyl)-2-methylpropanamide (**3s**)

Following general procedure D, lactosylamide **2s** (0.161 g, 0.195 mmol) gave **3s** (0.100 g, 96%) as a white solid. The analytical sample was obtained by HPLC (A: H_2_O, B: CH_3_OH; 30% to 90% B in 30 min, followed by isocratic 90% B for 10 min). [α]_D_^20^ = +32.6 (*c* 0.3 in CH_3_OH); ^1^H NMR (500 MHz, D_2_O, *T* = 100 °C): δ 0.99 (d, *J*_CH_3_,CH_ = 5.9 Hz, 3H, CH­(C**H**_**3**_)_**a**,b_), 1.07 (d, *J*_CH_3_,CH_ = 6.6 Hz, 3H, CH­(C**H**_**3**_)_a,**b**_), 2.50–2.53 (m, 1H, C**H**(CH_3_)_a,b_), 3.03 (dd, *J*_2,3_ = 9.6, *J*_2,1_ = 9.0 Hz, 1H, H-2), 3.43 (dd, *J*_4,5_ = 9.8, *J*_4,3_ = 8.9 Hz, 1H, H-4), 3.55 (dd, *J*_2′,3′_ = 9.9, *J*_2′,1′_ = 7.7 Hz, 1H, H-2′), 3.63 (dd, *J*_3′,2′_ = 9.9, *J*_3′,4′_ = 3.4 Hz, 1H, H-3′), 3.66 (ddd, *J*_5′,6′a_ = 7.3, *J*_5′,6′b_ = 4.7, *J*_5′,4′_ = 1.1 Hz, 1H, H-5′), 3.70 (ddd, *J*_5,4_ = 10.6, *J*_5,6a_ = 5.1, *J*_5,6b_ = 2.4 Hz, 1H, H-5), 3.73 (dd, *J*_gem_ = 11.7, *J*_6′b,5′_ = 4.7 Hz, 1H, H-6′b), 3.73 (t, *J*_3,4_ = *J*_3,2_ = 9.0 Hz, 1H, H-3), 3.76 (dd, *J*_gem_ = 11.8, *J*_6′a,5′_ = 7.3 Hz, 1H, H-6′a), 3.80 (dd, *J*_gem_ = 12.3, *J*_6a,5_ = 5.2 Hz, 1H, H-6a), 3.92 (dd, *J*_4′,3′_ = 3.4, *J*_4′,5′_ = 1.0 Hz, 1H, H-4′), 3.98 (dd, *J*_gem_ = 12.3, *J*_6b,5_ = 2.4 Hz, 1H, H-6b), 4.39 (d, *J*_1′,2′_ = 7.7 Hz, 1H, H-1′), 5.65 (d, *J*_1,2_ = 8.6 Hz, 1H, H-1), 7.66 (td, *J*_5″,6′′_ = *J*_5″,4′′_ = 7.9, *J*_5″,2′′_ = 0.5 Hz, 1H, H-5″), 7.69 (dt, *J*_6″,5′′_ = 7.9, *J*_6″,2′′_ = *J*_6″,4′′_ = 1.8 Hz, 1H, H-6″), 7.99 (td, *J*_2″,6′′_ = *J*_2″,4′′_ = 2.1, *J*_2″,5′′_ = 0.5 Hz, 1H, H-2″), 8.11 (dt, *J*_4″,5′′_ = 7.3, *J*_4″,2′′_ = *J*_4″,6′′_ = 1.6 Hz, 1H, H-4″); ^13^C NMR (126 MHz, D_2_O, *T* = 100 °C): δ 18.96 (CH­(**C**H_3_)_**a**,b_), 19.05 (CH­(**C**H_3_)_a,**b**_), 32.50 (**C**H­(CH_3_)_a,b_), 61.21 (CH_2_-6), 61.46 (CH_2_-6′), 69.20 (CH-4′), 70.07 (CH-2), 71.59 (CH-2′), 73.32 (CH-3′), 75.79 (CH-5′), 76.15 (CH-3), 77.39 (CH-5), 79.00 (CH-4), 83.12 (CH-1), 103.38 (CH-1′), 130.20 (CH-5″), 130.51 (CH-4″), 131.35 (CH-2″), 131.74 (C-3″), 135.52 (CH-6″), 137.74 (C-1‴), 169.76 (COOH), 182.58 (CON); IR (CH_3_OH): 3377, 3080, 2926, 1783, 1719, 1657, 1603, 1586, 1487, 1448, 1415, 1372, 1291, 1218, 1167, 1119, 1078, 1043, 991, 894, 708 cm^–1^; HRMS (ESI): [M – H]^−^ *m*/*z* calcd for C_23_H_32_O_13_N: 530.1879, found: 530.1877.

##### *N*-[4-*O*-(β-d-Galactopyranosyl)-β-d-glucopyranosyl]-*N*-(3-carboxyphenyl)­benzamide (**3t**)

Following general procedure D, lactosylamide **2t** (0.133 g, 0.155 mmol) gave **3t** (0.032 g, 37%) as a white solid. The analytical sample was obtained by HPLC (A: H_2_O, B: CH_3_OH; 30% to 90% B in 30 min, followed by isocratic 90% B for 10 min). [α]_D_^20^ = +56.6 (*c* 0.2 in CH_3_OH); ^1^H NMR (500 MHz, D_2_O, *T* = 100 °C): δ 3.18 (t, *J*_2,1_ = 9.2 Hz, 1H, H-2), 3.49 (dd, *J*_4,5_ = 9.8, *J*_4,3_ = 8.9 Hz, 1H, H-4), 3.55 (dd, *J*_2′,3′_ = 9.9, *J*_2′,1′_ = 7.7 Hz, 1H, H-2′), 3.63 (dd, *J*_3′,2′_ = 9.9, *J*_3′,4′_ = 3.4 Hz, 1H, H-3′), 3.66 (ddd, *J*_5′,6′a_ = 7.3, *J*_5′,6′b_ = 4.8, *J*_5′,4′_ = 1.2 Hz, 1H, H-5′), 3.59–3.71 (m, 2H, H-3,5), 3.73 (dd, *J*_gem_ = 11.7, *J*_6′b,5′_ = 4.8 Hz, 1H, H-6′b), 3.75 (dd, *J*_gem_ = 11.8, *J*_6′a,5′_ = 7.3 Hz, 1H, H-6′a), 3.85 (dd, *J*_gem_ = 12.4, *J*_6a,5_ = 5.2 Hz, 1H, H-6a), 3.92 (dd, *J*_4′,3′_ = 3.4, *J*_4′,5′_ = 0.9 Hz, 1H, H-4′), 4.03 (dd, *J*_gem_ = 12.4, *J*_6b,5_ = 2.4 Hz, 1H, H-6b), 4.40 (d, *J*_1′,2′_ = 7.7 Hz, 1H, H-1′), 5.54 (br s, 1H, H-1), 7.35–7.41 (m, 2H, Ar-H), 7.42–7.48 (m, 3H, Ar-H), 7.49–7.55 (m, 1H, Ar-H), 7.63–7.66 (m, 1H, Ar-H), 7.95–7.98 (m, 2H, Ar-H); ^13^C NMR (126 MHz, D_2_O, *T* = 100 °C): δ 61.20 (CH_2_-6), 61.46 (CH_2_-6′), 69.20 (CH-4′), 70.11 (CH-2), 71.58 (CH-2′), 73.32 (CH-3′), 75.79 (CH-5′), 76.05 (CH-3), 77.55 (CH-5), 78.93 (CH-4), 85.13 (CH-1), 103.36 (CH-1′), 127.91, 128.84, 129.88, 130.01, 130.92, 131.37, 131.44, 135.33, 135.51, 138.01 (10 × C-Ar), 169.63 (COOH), 175.50 (CON); IR (CH_3_OH): 3363, 3063, 2916, 1783, 1711, 1650, 1601, 1587, 1579, 1492, 1448, 1415, 1291, 1216, 1166, 1119, 1077, 1044, 1025, 1002, 891, 702, 702, 674, 578 cm^–1^; HRMS (ESI): [M – H]^−^ *m*/*z* calcd for C_26_H_30_O_13_N: 564.1723, found: 564.1720.

##### *N*-[4-*O*-(β-d-Galactopyranosyl)-β-d-glucopyranosyl]-*N*-(3-carboxyphenyl)-2-phenylacetamide (**3u**)

Following general procedure D, lactosylamide **2u** (0.214 g, 0.245 mmol) gave **3u** (0.088 g, 62%) as a white solid. The analytical sample was obtained by HPLC (A: H_2_O, B: CH_3_OH; 30% to 90% B in 30 min, followed by isocratic 90% B for 10 min). [α]_D_^20^ = +23.1 (*c* 0.3 in CH_3_OH); ^1^H NMR (500 MHz, D_2_O, *T* = 100 °C): δ 3.04 (t, *J*_2,3_ = *J*_2,1_ = 9.2 Hz, 1H, H-2), 3.42 (dd, *J*_4,5_ = 9.9, *J*_4,3_ = 8.9 Hz, 1H, H-4), 3.54 (dd, *J*_2′,3′_ = 9.9, *J*_2′,1′_ = 7.7 Hz, 1H, H-2′), 3.62 (dd, *J*_3′,2′_ = 9.9, *J*_3′,4′_ = 3.4 Hz, 1H, H-3′), 3.59–3.69 (m, 3H, CH_2_CON, H-5), 3.65 (ddd, *J*_5′,6′a_ = 7.3, *J*_5′,6′b_ = 4.9, *J*_5′,4′_ = 1.2 Hz, 1H, H-5′), 3.70–3.73 (m, 1H, H-3), 3.72 (dd, *J*_gem_ = 11.7, *J*_6′b,5′_ = 4.8 Hz, 1H, H-6′b), 3.75 (dd, *J*_gem_ = 11.8, *J*_6′a,5′_ = 7.2 Hz, 1H, H-6′a), 3.77 (dd, *J*_gem_ = 12.3, *J*_6a,5_ = 5.1 Hz, 1H, H-6a), 3.91 (dd, *J*_4′,3′_ = 3.4, *J*_4′,5′_ = 0.8 Hz, 1H, H-4′), 3.94 (dd, *J*_gem_ = 12.2, *J*_6b,5_ = 1.9 Hz, 1H, H-6b), 4.38 (d, *J*_1′,2′_ = 7.7 Hz, 1H, H-1′), 5.68 (br s, 1H, H-1), 7.03 (br s, 2H, Ar-H), 7.27–7.33 (m, 3H, Ar-H), 7.59–7.62 (m, 2H, Ar-H), 7.80 (br s, 1H, Ar-H), 8.06–8.09 (m, 1H, Ar-H); ^13^C NMR (126 MHz, D_2_O, *T* = 100 °C): δ 42.29 (**C**H_2_CON), 61.16 (CH_2_-6), 61.45 (CH_2_-6′), 69.20 (CH-4′), 70.04 (CH-2), 71.58 (CH-2′), 73.31 (CH-3′), 75.78 (CH-5′), 76.12 (CH-3), 77.39 (CH-5), 78.95 (CH-4), 83.52 (CH-1), 103.36 (CH-1′), 127.47, 129.10, 129.37, 130.14, 130.58, 131.65, 131.81, 134.90, 135.65, 137.34 (10 × C-Ar) 169.69 (COOH), 175.98 (CH_2_ **C**ON); IR (CH_3_OH): 3373, 3088, 3088, 3065, 3031, 2928, 1783, 1710, 1661, 1602, 1586, 1496, 1487, 1454, 1415, 1362, 1289, 1217, 1167, 1119, 1077, 1077, 1044, 1035, 1000, 939, 893, 737, 698, 698, 547 cm^–1^; HRMS (ESI): [M – H]^−^ *m*/*z* calcd for C_27_H_32_O_13_N: 578.1879, found: 578.1875.

##### *N*-[4-*O*-(β-d-Galactopyranosyl)-β-d-glucopyranosyl]-*N*-[3-(methylcarboxy)­phenyl]­acetamide (**3v**)

Following general procedure C, lactosylamide **2v** (0.380 g, 0.468 mmol) gave **3v** (0.196 g, 81%) as a white solid. The analytical sample was obtained by HPLC (A: H_2_O, B: CH_3_OH; 30% to 90% B in 30 min, followed by isocratic 90% B for 10 min). [α]_D_^20^ = +35.6 (*c* 0.3 in H_2_O); ^1^H NMR (500 MHz, methanol-*d*_4_, *T* = 25 °C): δ 1.83 (s, 3H, CH_3_CON), 2.84 (t, *J*_2,3_ = *J*_2,1_ = 9.0 Hz, 1H, H-2), 3.34 (t, *J*_4,5_ = *J*_4,3_ = 9.2 Hz, 1H, H-4), 3.45 (dd, *J*_3′,2′_ = 9.7, *J*_3′,4′_ = 3.3 Hz, 1H, H-3′), 3.49–3.55 (m, 1H, H-5′), 3.52 (dd, *J*_2′,3′_ = 9.7, *J*_2′,1′_ = 7.6 Hz, 1H, H-2′), 3.54–3.57 (m, 1H, H-5), 3.59 (t, *J*_3,4_ = *J*_3,2_ = 8.9 Hz, 1H, H-3), 3.64 (dd, *J*_gem_ = 11.5, *J*_6′b,5′_ = 4.4 Hz, 1H, H-6′a), 3.72 (dd, *J*_gem_ = 11.5, *J*_6′a,5′_ = 7.6 Hz, 1H, H-6′a), 3.77 (dd, *J*_4′,3′_ = 3.3, *J*_4′,5′_ = 1.0 Hz, 1H, H-4′), 3.81 (dd, *J*_gem_ = 12.1, *J*_6a,5_ = 4.6 Hz, 1H, H-6a), 3.92 (s, 3H, OCH_3_), 3.91–3.97 (m, 1H, H-6b), 4.28 (d, *J*_1′,2′_ = 7.6 Hz, 1H, H-1′), 5.73 (d, *J*_1,2_ = 9.4 Hz, 1H, H-1), 7.58 (t, *J*_5″,6′′_ = *J*_5″,4′′_ = 7.6 Hz, 1H, H-5″), 7.72–7.78 (m, 1H, H-6″), 8.02–8.09 (m, 1H, H-2″), 8.09 (br s, 1H, H-4″); ^13^C NMR (126 MHz, methanol-*d*_4_, *T* = 25 °C): δ 23.61 (**C**H_3_CON), 52.90 (OCH_3_), 62.07 (CH_2_-6), 62.53 (CH_2_-6′), 70.30 (CH-4′), 71.08 (CH-2), 72.51 (CH-2′), 74.77 (CH-3′), 77.06 (CH-5′), 77.65 (CH-3), 78.77 (CH-5), 80.06 (CH-4), 83.35 (CH-1), 104.97 (CH-1′), 130.63 (CH-5″), 130.84 (CH-2″), 132.53 (CH-4″), 132.60 (C-3″), 136.45 (CH-6″), 139.91 (C-1″), 167.73 (COOH), 174.18 (CH_3_ **C**ON); IR (CH_3_OH): 3375, 2951, 2931, 2835, 1722, 1663, 1602, 1586, 1486, 1438, 1438, 1298, 1258, 1116, 1079, 1079, 992, 892, 781, 707 cm^–1^; HRMS (ESI): [M + Na]^+^ *m*/*z* calcd for C_22_H_31_O_13_NNa: 540.1688, found: 540.1686; [M + H]^+^ *m*/*z* calcd for C_22_H_32_O_13_N: 518.1868, found: 518.1686.

##### *N*-[4-*O*-(β-d-Galactopyranosyl)-β-d-glucopyranosyl]-*N*-[3-(methylcarbamoyl)­phenyl]­acetamide (**3w**)

Lactosylamide **2v** (0.091 g, 0.113 mmol) was dissolved in ethanolic CH_3_NH_2_ (33%; 2 mL), and the reaction mixture was stirred for 72 h. The reaction mixture was concentrated in vacuo, after which the residue was purified by liquid column chromatography on silica gel (9:1 CH_3_CN/H_2_O) to give product **3w** (0.039 g, 66%) as a white foam. The analytical sample was obtained by HPLC (A: H_2_O, B: CH_3_OH; 30% to 90% B in 30 min, followed by isocratic 90% B for 10 min). [α]_D_^20^ = +42.0 (*c* 0.3 in H_2_O); ^1^H NMR (500 MHz, methanol-*d*_4_, *T* = 25 °C): δ 1.84 (s, 3H, CH_3_CON), 2.84 (t, *J*_2,3_ = *J*_2,1_ = 8.7 Hz, 1H, H-2), 2.93 (s, 3H, NHCH_3_), 3.37 (br s, 1H, H-4), 3.45 (dd, *J*_3′,2′_ = 9.8, *J*_3′,4′_ = 2.6 Hz, 1H, H-3′), 3.52 (dd, *J*_2′,3′_ = 9.7, *J*_2′,1′_ = 7.6 Hz, 1H, H-2′), 3.50–3.57 (m, 2H, H-5,5′), 3.59 (t, *J*_3,4_ = *J*_3,2_ = 8.9 Hz, 1H, H-3), 3.64 (dd, *J*_gem_ = 11.5, *J*_6′b,5′_ = 4.5 Hz, 1H, H-6′a), 3.72 (dd, *J*_gem_ = 11.5, *J*_6′a,5′_ = 7.6 Hz, 1H, H-6′a), 3.75–3.80 (m, 1H, H-4′), 3.85 (s, 1H, H-6a), 3.93 (d, *J*_gem_ = 12.0 Hz, 1H, H-6b), 4.29 (d, *J*_1′,2′_ = 7.7 Hz, 1H, H-1′), 5.73 (d, *J*_1,2_ = 9.4 Hz, 1H, H-1), 7.56 (t, *J*_5″,6′′_ = *J*_5″,4′′_ = 7.7 Hz, 1H, H-5″), 7.59–7.68 (m, 1H, H-6″), 7.84 (br s, 1H, H-2″), 7.86–7.91 (m, 1H, H-4″); ^13^C NMR (126 MHz, methanol-*d*_4_, *T* = 25 °C): δ 23.62 (**C**H_3_CON), 27.00 (NHCH_3_), 61.88 (CH_2_-6), 62.54 (CH_2_-6′), 70.31 (CH-4′), 71.06­(CH-2), 72.51 (CH-2′), 74.77 (CH-3′), 77.06 (CH-5′), 77.64 (CH-3), 78.67 (CH-5), 79.78 (CH-4), 83.31 (CH-1), 104.92 (CH-1′), 128.68 (CH-4″), 130.30 (CH-2″), 130.60 (CH-5″), 134.77 (CH-6″), 136.78 (C-3″), 139.77 (C-1″), 169.68 (**C**ONHCH_3_), 174.32 (CH_3_ **C**ON); IR (CH_3_OH): 3353, 2931, 1654, 1603, 1582, 1547, 1483, 1429, 1304, 1114, 1077, 1000, 893, 782, 707 cm^–1^; HRMS (ESI): [M + Na]^+^ *m*/*z* calcd for C_22_H_32_O_12_N_2_Na: 539.1848, found: 539.1846; [M + H]^+^ *m*/*z* calcd for C_22_H_33_O_12_N_2_: 517.2028, found: 517.2027.

##### *N*-[4-*O*-(β-d-Galactopyranosyl)-β-d-glucopyranosyl]-*N*-[3-(hydroxymethyl)­phenyl]­acetamide (**3x**)

Following general procedure C, lactosylamide **2x** (0.552 g, 0.669 mmol) gave **3x** (0.321 mg, 98%) as a white solid. The analytical sample was obtained by HPLC (A: H_2_O, B: CH_3_OH; 30% to 90% B in 30 min, followed by isocratic 90% B for 10 min). [α]_D_^20^ = +44.8 (*c* 0.4 in CH_3_OH); ^1^H NMR (500 MHz, DMSO-*d*_6_, *T* = 100 °C): δ 1.79 (s, 3H, CH_3_CON), 2.81 (td, *J*_2,1_ = *J*_2,3_ = 9.2, *J*_2,OH_ = 4.3 Hz, 1H, H-2), 3.15 (dd, *J*_4,5_ = 9.6, *J*_4,3_ = 8.9 Hz, 1H, H-4), 3.30–3.41 (m, 2H, H-2′,3′), 3.41 (ddd, *J*_5,4_ = 9.7, *J*_5,6a_ = 4.8, *J*_5,6b_ = 2.8 Hz, 1H, H-5), 3.43 (ddd, *J*_5′,6a′_ = 6.8, *J*_5′,6b′_ = 5.6, *J*_5′,4′_ = 1.3 Hz, 1H, H-5′), 3.48 (t, *J*_3,2_ = *J*_3,4_ = 8.8 Hz, 1H, H-3), 3.50–3.60 (m, 2H, H-6′), 3.64 (dt, *J*_gem_ = 11.7, *J*_6a,5_ = *J*_6b,OH_ = 4.9 Hz, 1H, H-6a), 3.66–3.68 (m, 1H, H-4′), 3.80 (dt, *J*_gem_ = 11.6, *J*_6b,5_ = *J*_6b,OH_ = 3.2 Hz, 1H, H-6b), 4.04–4.09 (m, 2H, OH-6,4′), 4.20 (br s, 1H, H-6″), 4.21 (d, *J*_1′,2′_ = 7.5 Hz, 1H, H-1′), 4.27 (br s, 1H, OH-3′), 4.47 (br s, 1H, OH-3), 4.54 (d, *J*_CH_2_,OH_ = 4.5 Hz, 2H, C**H**_**2**_OH), 4.63 (d, *J*_OH,2′_ = 5.3 Hz, 1H, OH-2′), 4.66 (d, *J*_OH,2_ = 2.2 Hz, 1H, OH-2), 4.84 (t, *J*_OH,CH_2__ = 4.7 Hz, 1H, CH_2_O**H**), 5.50 (d, *J*_1,2_ = 8.6 Hz, 1H, H-1), 7.21–7.26 (m, 1H, Ar-H), 7.31–7.33 (m, 1H, Ar-H), 7.33–7.36 (m, 2H, Ar-H); ^13^C NMR (126 MHz, DMSO-*d*_6_, *T* = 100 °C): δ 22.36 (**C**H_3_CON), 60.10 (CH_2_-6′), 60.57 (CH_2_-6), 62.25 (CH_2_OH), 67.88 (CH-4′), 69.34 (CH-2), 70.37 (CH-2′), 73.03 (CH-3′), 75.14 (CH-3), 75.41 (CH-5′), 76.60 (CH-5), 79.57 (CH-4), 82.15 (CH-1), 103.06 (CH-1′), 125.51, 127.70, 127.86, 127.99 (CH-2″,4″,5″,6″), 137.94 (C-1″), 142.86 (C-3″), 169.90 (CH_3_ **C**ON); IR (CHCl_3_): 3368, 2929, 2884, 2833, 1653, 1605, 1589, 1488, 1444, 1374, 1165, 1114, 1077, 1036, 1003, 705, 567 cm^–1^; HRMS (ESI): [M + Na]^+^ *m*/*z* calcd for C_21_H_31_O_12_NNa: 512.1739, found: 512.1739.

##### *N*-[4-*O*-(β-d-Galactopyranosyl)-β-d-glucopyranosyl]-*N*-[3-(1*H*-tetrazol-5-yl)­phenyl]­acetamide (**3y**)

Following general procedure C, lactosylamide **2y** (0.470 g, 0.572 mmol) gave **3y** (0.302 mg, 99%) as a white solid. The analytical sample was obtained by HPLC (A: H_2_O, B: CH_3_OH; 30% to 90% B in 30 min, followed by isocratic 90% B for 10 min). [α]_D_^20^ = +40.3 (*c* 0.3 in CH_3_OH); ^1^H NMR (500 MHz, DMSO-*d*_6_, *T* = 100 °C): δ 1.91 (s, 3H, CH_3_CON), 2.81 (dd, *J*_2,1_ = 9.5, *J*_2,3_ = 8.6 Hz, 1H, H-2), 3.18 (*J*_4,5_ = 9.8, *J*_4,3_ = 8.8 Hz, 1H, H-4), 3.32 (dd, *J*_3′,2′_ = 9.5, *J*_3′,4′_ = 3.3 Hz, 1H, H-3′), 3.37 (dd, *J*_2′,3′_ = 9.5, *J*_2′,1′_ = 7.5 Hz, 1H, H-2′), 3.42 (ddd, *J*_5′,6′a_ = 6.7, *J*_5′,6′b_ = 5.6, *J*_5′,4′_ = 1.2 Hz, 1H, H-5′), 3.46 (ddd, *J*_5,4_ = 9.8, *J*_5,6a_ = 4.7, *J*_5,6b_ = 2.8 Hz, 1H, H-5), 3.50 (t, *J*_3,2_ = *J*_3,4_ = 8.8 Hz, 1H, H-3), 3.51 (dd, *J*_gem_ = 11.0, *J*_6′a,5′_ = 6.6 Hz, 1H), 3.56 (dd, *J*_gem_ = 10.9, *J*_6′b,5′_ = 5.5 Hz, 1H, H-5′), 3.66 (dd, *J*_gem_ = 12.0, *J*_6a,5_ = 4.7 Hz, 1H, H-6a), 3.67 (dd, *J*_4′,3′_ = 3.2, *J*_4′,5′_ = 1.2 Hz, 1H, H-4′), 3.82 (dd, *J*_gem_ = 11.9, *J*_6b,5_ = 2.8 Hz, 1H, H-6b), 4.21 (d, *J*_1′,2′_ = 7.5 Hz, 1H, H-1′), 5.52 (d, *J*_1,2_ = 8.9 Hz, 1H, H-1), 7.57 (ddd, *J*_6″,5′′_ = 7.9, *J*_6″,2′′_ = 2.1, *J*_6″,4′′_ = 1.2 Hz, 1H, H-6″), 7.63 (td, *J*_5″,6′′_ = *J*_5″,4′′_ = 7.7, *J*_5″,2′′_ = 0.6 Hz, 1H, H-5″), 8.02 (td, *J*_2″,4′′_ = *J*_2″,6′′_ = 1.9, *J*_2″,5′′_ = 0.5 Hz, 1H, H-2″), 8.05 (ddd, *J*_4″,5′′_ = 7.6, *J*_4″,2′′_ = 1.8, *J*_4″,6′′_ = 1.3 Hz, 1H, H-4″); ^13^C NMR (126 MHz, DMSO-*d*_6_, *T* = 100 °C): δ 22.34 (**C**H_3_CON), 60.08 (CH_2_-6′), 60.51 (CH_2_-6), 67.87 (CH-4′), 69.43 (CH-2), 70.37 (CH-2′), 73.03 (CH-3′), 75.13 (CH-3), 75.31 (CH-5′), 76.73 (CH-5), 79.50 (CH-4), 82.89 (CH-1), 103.05 (CH-1′), 124.91 (C-1″), 126.04 (CH-4″), 128.58 (CH-2″), 129.14 (CH-5″), 132.26 (CH-6″), 138.92 (C-3″), 155.26 (C-Tz), 169.89 (CH_3_ **C**ON); IR (CH_3_OH): 3371, 3077, 2927, 2890, 2836, 1661, 1616, 1589, 1559, 1486, 1375, 1164, 1115, 1077, 1033, 1003, 784, 708 cm^–1^; HRMS (ESI): [M + Na]^+^ *m*/*z* calcd for C_21_H_29_O_11_N_5_Na: 550.1756, found: 550.1758.

##### *N*-[4-*O*-(β-d-Galactopyranosyl)-β-d-glucopyranosyl]-*N*-[3-(5-methylfuran-2-yl)­phenyl]­acetamide (**3z**)

Following general procedure C, lactosylamide **2z** (0.550 g, 0.660 mmol) gave **3z** (0.320 mg, 90%) as a white solid. The analytical sample was obtained by HPLC (A: H_2_O, B: CH_3_OH; 30% to 90% B in 30 min, followed by isocratic 90% B for 10 min). [α]_D_^20^ = +40.3 (*c* 0.4 in CH_3_OH); ^1^H NMR (500 MHz, DMSO-*d*_6_, *T* = 100 °C): δ 1.86 (s, 3H, CH_3_CON), 2.36 (d, *J*_CH_3_,4furan_ = 1.2 Hz, 3H, CH_3_), 2.85 (td, *J*_2,1_ = *J*_2,3_ = 9.1, *J*_2,OH_ = 5.2 Hz, 1H, H-2), 3.17 (dd, *J*_4,5_ = 9.7, *J*_4,3_ = 8.8 Hz, 1H, H-4), 3.29–3.36 (m, 1H, H-3′), 3.38 (ddd, *J*_2′,3′_ = 9.4, *J*_2′,1′_ = 7.5, *J*_2′,OH_ = 4.1 Hz, 1H, H-2′), 3.43 (ddd, *J*_5′,6′a_ = 6.6, *J*_5′,6′b_ = 5.5, *J*_5′,4′_ = 1.1 Hz, 1H, H-5′), 3.41–3.47 (m, 1H, H-5), 3.49 (td, *J*_3,2_ = *J*_3,4_ = 8.8, *J*_3,OH_ = 2.1 Hz, 1H, H-3), 3.51–3.59 (m, 2H, H-6′a,6′b), 3.64 (ddd, *J*_gem_ = 11.8, *J*_6a,OH_ = 6.0, *J*_6a,5_ = 4.8 Hz, 1H, H-6a), 3.67 (ddd, *J*_4′,OH_ = 4.3, *J*_4′,3′_ = 3.3, *J*_4′,5′_ = 1.2 Hz, 1H, H-4′), 3.81 (ddd, *J*_gem_ = 11.9, *J*_6b,OH_ = 5.6, *J*_6b,5_ = 2.8 Hz, 1H, H-6b), 4.05 (d, *J*_OH,4′_ = 4.5 Hz, 1H, OH-4′), 4.12 (t, *J*_OH,6_ = 6.0 Hz, 1H, OH-6), 4.18 (t, *J*_OH,6′_ = 5.4 Hz, 1H, OH-6′), 4.22 (d, *J*_1′,2′_ = 7.5 Hz, 1H, H-1′), 4.26 (d, *J*_OH,3′_ = 5.6 Hz, 1H, OH-3′), 4.48 (d, *J*_OH,3_ = 2.1 Hz, 1H, OH-3), 4.65 (d, *J*_OH,2′_ = 4.3 Hz, 1H, OH-2′), 4.73 (d, *J*_OH,2_ = 5.4 Hz, 1H, OH-2), 5.52 (d, *J*_1,2_ = 8.2 Hz, 1H, H-1), 6.18 (dd, *J*_4,3‴_ = 3.2, *J*_4,CH_3__ = 1.1 Hz, 3H, H-4-furan), 6.77 (d, *J*_3,4_ = 3.2 Hz, 1H, H-3-furane), 7.25 (ddd, *J*_6″,5′′_ = 7.9, *J*_6″,2′′_ = 1.9, *J*_6″,4′′_ = 1.2 Hz, 1H, H-6″), 7.42 (t, *J*_5″,6′′_ = *J*_5″,4′′_ = 8.1 Hz, 1H, H-5″), 7.60–7.64 (m, 2H, H-2″,4″); ^13^C NMR (126 MHz, DMSO-*d*_6_, *T* = 100 °C): δ 12.65 (CH_3_), 22.33 (CH_3_CON), 60.08 (CH_2_-6′), 60.54 (CH_2_-6), 67.87 (CH-4′), 69.42 (CH-2), 70.38 (CH-2′), 73.04 (CH-3′), 75.12 (CH-3), 75.38 (CH-5′), 76.63 (CH-5), 79.52 (CH-4), 82.18 (CH-1), 103.05 (CH-1′), 106.75 (CH-3-furan), 107.54 (CH-4-furan), 122.03 (CH-4″), 124.48 (CH-2″), 128.18 (CH-6″), 128.54 (CH-5″), 130.89 (C-3″), 138.67 (C-1″), 150.47 (C-2-furan), 151.53 (C-5-furan), 169.87 (CH_3_CON); IR (CH_3_OH): 3380, 3074, 2928, 2889, 2833, 1659, 1612, 1597, 1583, 1547, 1486, 1475, 1430, 1395, 1373, 1165, 1114, 1075, 1031, 999, 956, 787, 714 cm^–1^; HRMS (ESI): [M + Na]^+^ *m*/*z* calcd for C_25_H_33_O_12_NNa: 562.1895, found: 562.1898.

##### *N*-[4-*O*-(β-d-Galactopyranosyl)-β-d-glucopyranosyl]-*N*-[3-(1-methyl-1*H*-1,2,3-triazol-4-yl)­phenyl]­acetamide (**3aa**)

Following general procedure C, lactosylamide **2aa** (0.150 g, 0.180 mmol) gave **3aa** (0.087 mg, 90%) as a white solid. The analytical sample was obtained by HPLC (A: H_2_O, B: CH_3_OH; 30% to 90% B in 30 min, followed by isocratic 90% B for 10 min). [α]_D_^20^ = +38.1 (*c* 0.3 in CH_3_OH); ^1^H NMR (500 MHz, DMSO-*d*_6_, *T* = 100 °C): δ 1.86 (s, 3H, CH_3_CON), 2.85 (td, *J*_2,1_ = *J*_2,3_ = 9.0, *J*_2,OH_ = 5.0 Hz, 1H, H-2), 3.17 (dd, *J*_4,5_ = 9.8, *J*_4,3_ = 8.8 Hz, 1H, H-4), 3.32 (dt, *J*_3′,2′_ = 9.4, *J*_3′,4′_ = 3.4 Hz, 1H, H-3′), 3.38 (td, *J*_2′,3′_ = 9.5, *J*_2′,1′_ = 7.6, *J*_2′,OH_ = 3.2 Hz, 1H, H-2′), 3.43 (ddd, *J*_5′,6a′_ = 6.6, *J*_5′,6b′_ = 5.5, *J*_5′,4′_ = 1.2 Hz, 1H, H-5′), 3.45 (ddd, *J*_5,3_ = 9.7, *J*_5,6a_ = 4.7, *J*_5,6b_ = 2.8 Hz, 1H, H-5), 3.50 (td, *J*_3,2_ = *J*_3,4_ = 8.8, *J*_3,OH_ = 1.5 Hz, 1H, H-3), 3.51 (ddd, *J*_gem_ = 10.7, *J*_6′a,5′_ = 6.5, *J*_6′a,OH_ = 4.7 Hz, 1H, H-6′a), 3.56 (dt, *J*_gem_ = 10.6, *J*_6b′,5′_ = *J*_6b′,OH_ = 5.3 Hz, 1H, H-6b′), 3.63–3.69 (m, 2H, H-4′,6a), 3.81 (ddd, *J*_gem_ = 11.8, *J*_6b,OH_ = 5.4, *J*_6a,5_ = 2.8 Hz, 1H, H-6b), 4.06 (d, *J*_OH,4′_ = 4.4 Hz, 1H, OH-4′), 4.10 (s, 3H, CH_3_), 4.12 (t, *J*_OH,6a_ = *J*_OH,6b_ = 6.2 Hz, 1H, OH-6), 4.18 (t, *J*_OH,6′a_ = *J*_OH,6′b_ = 5.4 Hz, 1H, OH-6′), 4.21 (d, *J*_1′,2′_ = 7.5 Hz, 1H, H-1′), 4.27 (d, *J*_OH,3′_ = 4.0 Hz, 1H, OH-3′), 4.47 (d, *J*_OH,3_ = 2.0 Hz, 1H, OH-3), 4.66 (d, *J*_OH,2′_ = 3.7 Hz, 1H, OH-2′), 4.73 (d, *J*_OH,2_ = 5.4 Hz, 1H, OH-2), 5.53 (d, *J*_1,2_ = 8.4 Hz, 1H, H-1), 7.34 (ddd, *J*_6″,5′′_ = 7.9, *J*_6″,2′′_ = 2.0, *J*_6″,4′′_ = 1.2 Hz, 1H, H-6″), 7.47 (t, *J*_5″,6′′_ = *J*_5″,4′′_ = 7.8 Hz, 1H, H-5″), 7.79 (t, *J*_2″,6′′_ = *J*_2″,4′′_ = 1.9 Hz, 1H, H-2″), 7.85 (dt, *J*_4″,5′′_ = 7.7, *J*_4″,6′′_ = *J*_4″,2′′_ = 1.4 Hz, 1H, H-4″), 8.37 (s, 3H, H-5-triazole); ^13^C NMR (126 MHz, DMSO-*d*_6_, *T* = 100 °C): δ 22.36 (**C**H_3_CON), 35.73 (CH_3_), 60.08 (CH_2_-6′), 60.53 (CH_2_-6), 67.87 (CH-4′), 69.43 (CH-2), 70.37 (CH-2′), 73.03 (CH-3′), 75.12 (CH-3), 75.37 (CH-5′), 76.63 (CH-5), 79.51 (CH-4), 82.51 (CH-1), 103.04 (CH-1′), 121.92 (CH-5-triazole), 124.28 (CH-4″), 126.80 (CH-2″), 128.57 (CH-5″), 129.04 (CH-6″), 131.19 (C-3″), 138.66 (C-1″), 145.52 (C-4-triazole), 169.87 (CH_3_ **C**ON); IR (CH_3_OH): 3367, 3136, 3084, 2931, 2887, 2833, 1659, 1611, 1585, 1557, 1486, 1374, 1165, 1114, 1079, 1033, 1002, 702 cm^–1^; HRMS (ESI): [M + Na]^+^ *m*/*z* calcd for C_23_H_32_O_11_NNa: 563.1960, found: 563.1957.

##### *N*-[4-*O*-(β-d-Galactopyranosyl)-β-d-glucopyranosyl]-*N*-(3-carboxy-5-chlorophenyl)­acetamide (**3ab**)

Following general procedure C, lactosylamide **2ab** (0.270 g, 0.324 mmol) gave **3ab** (0.161 mg, 92%) as a white solid. The analytical sample was obtained by HPLC (A: H_2_O, B: CH_3_OH; 30% to 90% B in 30 min, followed by isocratic 90% B for 10 min). [α]_D_^20^ = +39.1 (*c* 0.3 in H_2_O); ^1^H NMR (500 MHz, DMSO-*d*_6_, *T* = 100 °C): δ 1.93 (s, 3H, CH_3_CON), 2.77 (dd, *J*_2,1_ = 9.4, *J*_2,3_ = 8.8 Hz, 1H, H-2), 3.19 (dd, *J*_4,5_ = 9.6, *J*_4,3_ = 8.9 Hz, 1H, H-4), 3.33 (dd, *J*_3′,2′_ = 9.5, *J*_3′,4′_ = 3.3 Hz, 1H, H-3′), 3.38 (dd, *J*_2′,3′_ = 9.5, *J*_2′,1′_ = 7.5 Hz, 1H, H-2′), 3.42–3.48 (m, 2H, H-5,5′), 3.50 (t, *J*_3,4_ = *J*_3,2_ = 8.7 Hz, 1H, H-3), 3.52 (dd, *J*_gem_ = 10.9, *J*_6a′,5′_ = 6.5 Hz, 1H, H-6a′), 3.57 (dd, *J*_gem_ = 10.8, *J*_6b′,5′_ = 5.6 Hz, 1H, H-6b′), 3.63 (dd, *J*_gem_ = 11.9, *J*_6a,5_ = 5.0 Hz, 1H, H-6a), 3.68 (dd, *J*_4′,3′_ = 3.3, *J*_4′,5′_ = 1.2 Hz, 1H, H-4′), 3.82 (dd, *J*_gem_ = 11.9, *J*_6b,5_ = 2.7 Hz, 1H, H-6b), 4.01–4.35 (m, 3H, OH), 4.23 (d, *J*_1′,2′_ = 7.5 Hz, 1H, H-1′), 4.51 (br s, 2H, OH), 4.64 (br s, 1H, OH), 4.88 (br s, 1H, OH), 5.42 (d, *J*_1,2_ = 9.3 Hz, 1H, H-1), 7.64 (t, *J*_6″,4′′_ = *J*_6″,2′′_ = 1.9 Hz, 1H, H-6″), 7.84 (t, *J*_2″,4′′_ = *J*_2″,6′′_ = 1.7 Hz, 1H, H-2″), 7.90 (t, *J*_4″,6′′_ = *J*_4″,2′′_ = 1.7 Hz, 1H, H-4″); ^13^C NMR (126 MHz, DMSO-*d*_6_, *T* = 100 °C): δ 22.16 (**C**H_3_CON), 60.10 (CH_2_-6′), 60.52 (CH_2_-6), 67.89 (CH-4′), 69.48 (CH-2), 70.39 (CH-2′), 73.06 (CH-3′), 75.15 (CH-3), 75.26 (CH-5′), 76.84 (CH-5), 79.52 (CH-4), 83.25 (CH-1), 103.07 (CH-1′), 127.90 (CH-4″), 129.45 (CH-2″), 132.56, 133.01 (2 × C-3″,5″), 133.60 (CH-6″), 139.62 (C-1″), 164.95 (COOH), 169.89 (CH_3_ **C**ON); IR (CH_3_OH): 3366, 3083, 2931, 2627, 1713, 1666, 1597, 1578, 1448, 1119, 1076, 1041, 892, 825, 717, 670 cm^–1^; HRMS (ESI): [M + H]^+^ *m*/*z* calcd for C_21_H_27_O_13_NCl: 536.1176, found: 536.1174.

##### *N*-[4-*O*-(β-d-Galactopyranosyl)-β-d-glucopyranosyl]-*N*-(3-bromo-5-carboxyphenyl)­acetamide (**3ac**)

Following general procedure C, lactosylamide **2ac** (0.260 g, 0.297 mmol) gave **3ac** (0.150 mg, 87%) as a white solid. The analytical sample was obtained by HPLC (A: H_2_O, B: CH_3_OH; 30% to 90% B in 30 min, followed by isocratic 90% B for 10 min). [α]_D_^20^ = +34.8 (*c* 0.2 in H_2_O); ^1^H NMR (500 MHz, DMSO-*d*_6_, *T* = 100 °C): δ 1.93 (s, 3H, CH_3_CON), 2.77 (t, *J*_2,1_ = *J*_2,3_ = 9.0 Hz, 1H, H-2), 3.19 (dd, *J*_4,5_ = 9.6, *J*_4,3_ = 8.9 Hz, 1H, H-4), 3.33 (dd, *J*_3′,2′_ = 9.5, *J*_3′,4′_ = 3.3 Hz, 1H, H-3′), 3.38 (dd, *J*_2′,3′_ = 9.4, *J*_2′,1′_ = 7.5 Hz, 1H, H-2′), 3.43–3.48 (m, 2H, H-5,5′), 3.50 (t, *J*_3,4_ = *J*_3,2_ = 8.7 Hz, 1H, H-3), 3.52 (dd, *J*_gem_ = 10.8, *J*_6a′,5′_ = 6.5 Hz, 1H, H-6a′), 3.57 (dd, *J*_gem_ = 10.9, *J*_6b′,5′_ = 5.6 Hz, 1H, H-6b′), 3.63 (dd, *J*_gem_ = 11.9, *J*_6a,5_ = 5.0 Hz, 1H, H-6a), 3.68 (dd, *J*_4′,3′_ = 3.3, *J*_4′,5′_ = 1.1 Hz, 1H, H-4′), 3.82 (dd, *J*_gem_ = 11.9, *J*_6b,5_ = 2.7 Hz, 1H, H-6b), 3.99–4.12 (m, 3H, OH), 4.23 (d, *J*_1′,2′_ = 7.5 Hz, 1H, H-1′), 4.52 (br s, 1H, OH), 4.57–4.75 (m, 2H, OH), 4.88 (br s, 1H, OH), 5.41 (d, *J*_1,2_ = 8.4 Hz, 1H, H-1), 7.77 (t, *J*_6″,4′′_ = *J*_6″,2′′_ = 1.9 Hz, 1H, H-6″), 7.88 (t, *J*_2″,4′′_ = *J*_2″,6′′_ = 1.6 Hz, 1H, H-2″), 8.04 (t, *J*_4″,6′′_ = *J*_4″,2′′_ = 1.7 Hz, 1H, H-4″); ^13^C NMR (126 MHz, DMSO-*d*_6_, *T* = 100 °C): δ 22.17 (**C**H_3_CON), 60.11 (CH_2_-6′), 60.55 (CH_2_-6), 67.89 (CH-4′), 69.49 (CH-2), 70.40 (CH-2′), 73.07 (CH-3′), 75.15 (CH-3), 75.27 (CH-5′), 76.86 (CH-5), 79.56 (CH-4), 83.12 (CH-1), 103.08 (CH-1′), 120.44 (C-5″), 129.84 (CH-2″), 130.83 (CH-4″), 133.24 (C-3″), 136.39 (CH-6″), 139.71 (C-1″), 164.86 (COOH), 169.91 (CH_3_ **C**ON); IR (CH_3_OH): 3369, 3083, 2932, 2624, 1712, 1666, 1596, 1571, 1446, 1119, 1076, 1042, 892, 800, 774, 711 cm^–1^; HRMS (ESI): [M + H]^+^ *m*/*z* calcd for C_21_H_27_O_13_NBr: 580.0671, found: 580.0669.

##### *N*-[4-*O*-(β-d-Galactopyranosyl)-β-d-glucopyranosyl]-*N*-(3-carboxy-5-hydroxyphenyl)­acetamide (**3ad**)

Following general procedure C, lactosylamide **2ad** (0.171 g, 0.20 mmol) gave **3ad** (0.090 mg, 87%) as a white solid. The analytical sample was obtained by HPLC (A: H_2_O, B: CH_3_OH; 30% to 90% B in 30 min, followed by isocratic 90% B for 10 min). [α]_D_^20^ = +39.1 (*c* 0.3 in H_2_O); ^1^H NMR (500 MHz, DMSO-*d*_6_, *T* = 100 °C): δ 1.84 (s, 3H, CH_3_CON), 2.84 (dd, *J*_2,1_ = 9.4, *J*_2,3_ = 8.7 Hz, 1H, H-2), 3.17 (dd, *J*_4,5_ = 9.7, *J*_4,3_ = 8.8 Hz, 1H, H-4), 3.33 (dd, *J*_3′,2′_ = 9.5, *J*_3′,4′_ = 3.3 Hz, 1H, H-3′), 3.37 (dd, *J*_2′,3′_ = 9.5, *J*_2′,1′_ = 7.4 Hz, 1H, H-2′), 3.41 (ddd, *J*_5,4_ = 9.7, *J*_5,6a_ = 5.0, *J*_5,6b_ = 2.8 Hz, 1H, H-5), 3.44 (ddd, *J*_5′,6′a_ = 6.6, *J*_5′,6′b_ = 5.5, *J*_5′,4′_ = 1.2 Hz, 1H, H-5′), 3.47 (t, *J*_3,4_ = *J*_3,2_ = 8.8 Hz, 1H, H-3), 3.51 (dd, *J*_gem_ = 10.9, *J*_6a′,5′_ = 6.5 Hz, 1H, H-6a′), 3.56 (dd, *J*_gem_ = 10.9, *J*_6b′,5′_ = 5.6 Hz, 1H, H-6b′), 3.62 (dd, *J*_gem_ = 11.9, *J*_6a,5_ = 5.0 Hz, 1H, H-6a), 3.67 (dd, *J*_4′,3′_ = 3.2, *J*_4′,5′_ = 1.2 Hz, 1H, H-4′), 3.80 (dd, *J*_gem_ = 11.9, *J*_6b,5_ = 2.8 Hz, 1H, H-6b), 4.22 (d, *J*_1′,2′_ = 7.4 Hz, 1H, H-1′), 5.44 (d, *J*_1,2_ = 9.3 Hz, 1H, H-1), 7.00 (t, *J*_6″,4′′_ = *J*_6″,2′′_ = 2.1 Hz, 1H, H-6″), 7.34–7.38 (m, 2H, H-2″,4″); ^13^C NMR (126 MHz, DMSO-*d*_6_, *T* = 100 °C): δ 22.34 (**C**H_3_CON), 60.16 (CH_2_-6′), 60.71 (CH_2_-6), 67.92 (CH-4′), 69.46 (CH-2), 70.44 (CH-2′), 73.03 (CH-3′), 75.22 (CH-3), 75.48 (CH-5′), 76.86 (CH-5), 79.74 (CH-4), 82.64 (CH-1), 103.18 (CH-1′), 115.48 (CH-4″), 121.13 (CH-6″), 121.74 (CH-2″), 132.75 (C-3″), 139.18 (C-1″), 156.94 (C-5″), 166.45 (COOH), 170.25 (CH_3_ **C**ON); IR (CH_3_OH): 3363, 3078, 2935, 2643, 1709, 1659, 1597, 1493, 1452, 1323, 1114, 1078, 1039, 1001, 954, 855, 697, 555 cm^–1^; HRMS (ESI): [M + Na]^+^ *m*/*z* calcd for C_21_H_28_O_14_NNa: 518.1515, found: 518.1512.

##### *N*-[4-*O*-(β-d-Galactopyranosyl)-β-d-glucopyranosyl]-*N*-[6-(methylcarboxy)-1*H*-indol-4-yl]­acetamide (**3ae**)

Following general procedure C, lactosylamide **2ae** (0.390 g, 0.46 mmol) gave **3ae** (0.175 g, 69%) as a white solid. The analytical sample was obtained by HPLC (A: H_2_O, B: CH_3_OH; 30% to 90% B in 30 min, followed by isocratic 90% B for 10 min). [α]_D_^20^ = +25.4 (*c* 0.3 in CH_3_OH); ^1^H NMR (500 MHz, DMSO, *T* = 100 °C): δ 1.72 (s, 3H, CH_3_CON), 2.92 (td, *J*_2,3_ = *J*_2,1_ = 9.0, *J*_2,OH_ = 5.6 Hz, 1H, H-2), 3.08 (br s, 1H, H-4), 3.31 (ddd, *J*_3′,2′_ = 9.3, *J*_3′,OH_ = 5.5, *J*_3′,4′_ = 3.5 Hz, 1H, H-3′), 3.36 (ddd, *J*_2′,3′_ = 9.6, *J*_2′,1′_ = 7.5, *J*_2′,OH_ = 4.1 Hz, 1H, H-2′), 3.38–3.44 (m, 1H, H-5′), 3.44 (ddd, *J*_5,4_ = 9.9, *J*_5,6a_ = 4.9, *J*_5,6b_ = 3.0 Hz, 1H, H-5), 3.47–3.52 (m, 2H, H-3,6′a), 3.55 (dt, *J*_gem_ = 10.9, *J*_6′b,5′_ = *J*_6′b,OH_ = 5.5 Hz, 1H, H-6′b), 3.59 (br s, 1H, H-6a), 3.66 (ddd, *J*_4′,OH_ = 4.6, *J*_4′,3′_ = 3.2, *J*_4′,5′_ = 0.9 Hz, 1H, H-4′), 3.81 (br s, 1H, H-6b), 3.88 (s, 3H, OCH_3_), 4.05 (d, *J*_OH,4′_ = 4.4 Hz, 1H, OH-4′), 4.17 (t, *J*_OH,6′_ = 5.3 Hz, 1H, OH-6′), 4.19 (br s, 1H, H-1′), 4.26 (d, *J*_OH,3′_ = 5.5 Hz, 1H, OH-3′), 4.43 (br s, 1H, OH-3), 4.66 (d, *J*_OH,2′_ = 4.0 Hz, 1H, OH-2′), 5.60 (br s, 1H, H-1′), 6.64 (d, *J*_3_ind_,2_ind__ = 2.6 Hz, 1H, H-3_ind_), 7.53 (d, *J*_2_ind_,3_ind__ = 3.0 Hz, 1H, H-2_ind_), 7.70 (s, 1H, H-5_ind_), 8.10 (s, 1H, H-7_ind_), 11.31 (s, 1H, H-1_ind_); ^13^C NMR (126 MHz, DMSO, *T* = 100 °C): δ 21.91 (**C**H_3_CON), 51.07 (OCH_3_), 60.06 (CH_2_-6′), 60.94 (CH_2_-6), 67.86 (CH-4′), 69.68 (CH-2), 70.37 (CH-2′), 73.02 (CH-3′), 75.12 (CH-5′), 75.59 (CH-5), 76.63 (CH-3), 79.82 (CH-4), 83.51 (CH-1), 101.02 (CH-3_ind_), 103.06 (CH-1′), 112.94 (CH-7_ind_), 120.56 (CH-5_ind_), 122.14 (C-6_ind_), 128.28 (C-4_ind_), 128.78 (CH-2_ind_), 131.12 (C-3a_ind_), 135.89 (C-7a_ind_), 166.39 (COOH), 170.20 (CH_3_ **C**ON); IR (CH_3_OH): 3361, 3023, 2930, 2837, 1702, 1657, 1573, 1510, 1488, 1434, 1373, 1249, 1077, 1077, 1040, 689 cm^–1^; HRMS (ESI): [M + Na]^+^ *m*/*z* calcd for C_24_H_32_O_13_N_2_Na: 579.1797, found: 579.1798; [M + H]^+^ *m*/*z* calcd for C_24_H_33_O_13_N_2_: 557.1977, found: 557.1979.

##### *N*-[4-*O*-(β-d-Galactopyranosyl)-β-d-glucopyranosyl]-*N*-(6-carboxy-1*H*-indol-4-yl)­acetamide (**3af**)

Methyl ester **3ae** (0.095 g, 0.17 mmol) was dissolved in 1:1 dioxane/H_2_O (5 mL), and LiOH·H_2_O (0.011 g, 0.256 mmol) was added. The reaction was stirred at room temperature for 2 h, after which Dowex 50WX8 (H^+^ form) was added. The slurry was filtered, and the resin was washed with CH_3_OH. The combined filtrate was concentrated in vacuo to give product **3af** (0.078 g, 85%) as a white foam. The analytical sample was obtained by HPLC (A: H_2_O, B: CH_3_OH; 30% to 90% B in 30 min, followed by isocratic 90% B for 10 min). [α]_D_^20^ = +23.9 (*c* 0.4 in CH_3_OH); ^1^H NMR (500 MHz, methanol-*d*_4_, *T* = 25 °C): δ 1.77 (s, 3H, CH_3_CON), 1.82 (s, 3H, CH_3_CON), 3.01 (t, *J*_2,3_ = *J*_2,1_ = 9.1 Hz, 1H, H-2), 3.11 (dd, *J*_2,1_ = 9.4, *J*_2,3_ = 9.0 Hz, 1H, H-2), 3.21 (dd, *J*_4,5_ = 9.7, *J*_4,3_ = 8.9 Hz, 1H, H-4), 3.38 (dd, *J*_4,5_ = 9.8, *J*_4,3_ = 8.8 Hz, 1H, H-4), 3.38 (dd, *J* = 9.8, 8.8 Hz, 1H, H-4), 3.41 (dd, *J*_3′,2′_ = 9.7, *J*_3′,4′_ = 3.3 Hz, 1H, H-3′), 3.44 (dd, *J*_3′,2′_ = 9.7, *J*_3′,4′_ = 3.3 Hz, 1H, H-3′), 3.47 (ddd, *J*_5′,6′a_ = 7.7, *J*_5′,6′b_ = 4.4, *J*_5′,4′_ = 1.0 Hz, 1H, H-5′), 3.50 (dd, *J*_2′,3′_ = 9.7, *J*_2′,1′_ = 7.7 Hz, 1H, H-2′), 3.47–3.53 (m, 1H, H-5′), 3.51 (dd, *J*_2′,3′_ = 9.9, *J*_2′,1′_ = 7.5 Hz, 1H, H-2′), 3.55 (t, *J*_3,4_ = *J*_3,2_ = 8.9 Hz, 1H, H-3), 3.54–3.59 (m, 2H, 2 × H-5), 3.61 (t, *J*_3,4_ = *J*_3,2_ = 8.9 Hz, 1H, H-3), 3.61 (dd, *J*_gem_ = 11.6, *J*_6′b,5′_ = 4.7 Hz, 1H, H-6′b), 3.61 (dd, *J*_gem_ = 11.5, *J*_6′b,5′_ = 4.4 Hz, 1H, H-5′), 3.69 (dd, *J*_gem_ = 11.6, *J*_6′a,5′_ = 7.6 Hz, 1H, H-6′a), 3.70 (dd, *J*_gem_ = 11.5, *J*_6′a,5′_ = 7.7 Hz, 1H, H-6′a), 3.69–3.73 (m, 1H, H-6a), 3.74 (dd, *J*_4′,3′_ = 3.3, *J*_4′,5′_ = 1.1 Hz, 1H, H-4′), 3.76 (dd, *J*_4′,3′_ = 3.3, *J*_4′,5′_ = 1.1 Hz, 1H, H-4′), 3.85 (dd, *J*_gem_ = 12.3, *J*_6a,5_ = 4.6 Hz, 1H, H-6a), 3.91 (dd, *J* = 12.0, *J*_6b,5_ = 2.6 Hz, 1H, H-6a), 3.99 (dd, *J*_gem_ = 12.2, *J*_6b,5_ = 2.4 Hz, 1H, H-6b), 4.21 (d, *J*_1′,2′_ = 7.7 Hz, 1H, H-1′), 4.29 (d, *J*_1′,2′_ = 7.6 Hz, 1H, H-1′), 5.78 (d, *J*_1,2_ = 9.5 Hz, 1H, H-1), 5.82 (d, *J*_1,2_ = 9.5 Hz, 1H, H-1), 6.69 (dd, *J*_3_ind_,2_ind__ = 3.1, *J*_3_ind_,7_ind__ = 0.8 Hz, 1H, H-3_ind_), 6.75 (dd, *J*_3_ind_,2_ind__ = 3.1, *J*_3_ind_,7_ind__ = 0.8 Hz, 1H, H-3_ind_), 7.49 (d, *J*_2_ind_,3_ind__ = 3.1 Hz, 1H, H-2_ind_), 7.52 (d, *J*_2_ind_,3_ind__ = 3.1 Hz, 1H, H-2_ind_), 7.83 (d, *J*_5_ind_,7_ind__ = 1.3 Hz, 1H, H-5_ind_), 7.85 (d, *J*_5_ind_,7_ind__ = 1.3 Hz, 1H, H-5_ind_), 8.20 (t, *J*_7_ind_,5_ind__ = *J*_7_ind_,3_ind__ = 1.2 Hz, 1H, H-7_ind_), 8.21 (t, *J*_7_ind_,5_ind__ = *J*_7_ind_,3_ind__ = 1.2 Hz, 1H, H-7_ind_); ^13^C NMR (126 MHz, methanol-*d*_4_, *T* = 25 °C): δ 23.04 (**C**H_3_CON), 23.05 (**C**H_3_CON), 62.32 (CH_2_-6′), 62.52 (CH_2_-6), 62.56 (CH_2_-6), 62.64 (CH_2_-6′), 70.30 (CH-4′), 70.34 (CH-4′), 71.24 (CH-2), 71.45 (CH-2), 72.49 (CH-2′), 72.56 (CH-2′), 74.76 (CH-3′), 74.77 (CH-3′), 77.02 (CH-5′), 77.05 (CH-5′), 77.80 (CH-5), 78.05 (CH-5), 78.62 (CH-3), 78.79 (CH-3), 80.08 (CH-4), 80.52 (CH-4), 84.32 (CH-1), 84.67 (CH-1), 102.06 (CH-3_ind_), 102.77 (CH-3_ind_), 104.92 (CH-1′), 105.01 (CH-1′), 115.53 (CH-7_ind_), 115.62 (CH-7_ind_), 122.82 (CH-5_ind_), 123.46 (CH-5_ind_), 124.81 (C-6_ind_), 124.86 (C-6_ind_), 130.44 (CH-2_ind_), 130.54 (CH-2_ind_), 130.85 (C-4_ind_), 131.39 (C-4_ind_), 132.32 (C-3a_ind_), 133.00 (C-3a_ind_), 138.01 (C-7a_ind_), 138.24­(C-7a_ind_), 170.64 (COOH), 170.79 (COOH), 174.98 (CH_3_ **C**ON), 175.32 (CH_3_ **C**ON); IR (CH_3_OH): 3354, 3200, 2931, 2837, 2636, 1694, 1658, 1572, 1511, 1488, 1416, 1375, 1247, 1077, 1039, 787, 717 cm^–1^; HRMS (ESI): [M + Na]^+^ *m*/*z* calcd for C_23_H_30_O_13_N_2_Na: 565.1640, found: 565.1641.

##### *N*-[2,3,6-Tri-*O*-acetyl-4-*S*-(2,4,6-tri-*O*-acetyl-3-deoxy-3-(4-(3-fluorophenyl)-1*H*-1,2,3-triazol-1-yl)-β-d-galactopyranosyl)-4-thio-β-d-glucopyranosyl]-*N*-[6-(methylcarboxy)-1*H*-indol-4-yl]­acetamide (**9**)

In a sealed pressure tube, thiolactose **7** (0.107 g, 0.2 mmol) and 4-amino-6-(methylcarboxy)-1*H*-indole (0.076 g, 0.4 mmol) were dissolved in CH_3_OH (4 mL) and AcOH (0.01 mL). The solution was stirred at 80 °C for 4 h, cooled to room temperature, and concentrated in vacuo. The residue containing compound **8** was dissolved in pyridine (5 mL, 62 mmol), followed by the addition of Ac_2_O (4 mL, 42 mmol). After stirring at room temperature for 4 h, the reaction mixture was concentrated in vacuo. The residue was then dissolved in EtOAc (20 mL) and washed with a saturated solution of NaHCO_3_ (5 mL) and brine (5 mL). The organic phase was dried over anhydrous MgSO_4_, filtered, and concentrated in vacuo. The residue was purified by liquid column chromatography on silica gel (20:1 → 10:1 CHCl_3_/EtOH). The obtained crude solid residue (0.097 g, ∼0.1 mmol) was dissolved in CH_3_NO_2_ (2 mL), followed by the addition of Ac_2_O (0.1 mL; 1.1 mmol) and ZnCl_2_ (0.16 g, 0.12 mmol). The reaction mixture was stirred at 35 °C for 3 h, at which point the TLC indicated complete conversion of the starting material. The reaction mixture was diluted with EtOAc (30 mL) and washed with a saturated solution of NaHCO_3_ (5 mL) and brine (5 mL). The organic phase was separated and dried over anhydrous MgSO_4_, filtered, and concentrated in vacuo. The residue was purified by liquid column chromatography on silica gel (20:1 → 10:1 CHCl_3_/EtOH) to give product **9** (0.087 g, 45%) as a red powder.

^1^H NMR (500 MHz, DMSO-*d*_6_, *T* = 100 °C): δ 1.75 (s, 3H, CH_3_CO), 1.83 (s, 3H, CH_3_CO), 2.00 (s, 3H, CH_3_CO), 1.63–2.22 (m, 6H, CH_3_CO, CH_3_CON), 2.04 (s, 3H, CH_3_CO), 2.83–2.89 (m, 1H, H-4), 3.89 (s, 3H, OCH_3_), 3.96 (dd, *J*_gem_ = 11.6, *J*_6′a,5′_ = 7.7 Hz, 1H, H-6′a), 4.13 (dd, *J*_gem_ = 11.6, *J*_6′b,5′_ = 4.5 Hz, 1H, H-6′b), 4.19 (ddd, *J*_5,4_ = 10.9, *J*_5,6a_ = 4.5, *J*_5,6b_ = 2.2 Hz, 1H, H-5), 4.49 (ddd, *J*_5′,6′a_ = 7.6, *J*_5′,6′b_ = 4.5, *J*_5′,4′_ = 1.3 Hz, 1H, H-5′), 4.41–4.56 (m, 1H, H-4,6a), 4.60 (d, *J*_gem_ = 11.5 Hz, 1H, H-6b), 5.25 (d, *J*_1′,2′_ = 9.8 Hz, 1H, H-1′), 5.39 (dd, *J*_3′,2′_ = 10.8, *J*_3′,4′_ = 3.3 Hz, 1H, H-3′), 5.40–5.43 (m, 1H, H-3), 5.50 (dd, *J*_2′,3′_ = 10.8, *J*_2′,1′_ = 9.7 Hz, 1H, H-2′), 5.52 (dd, *J*_4′,3′_ = 3.3, *J*_4′,5′_ = 1.3 Hz, 1H, H-4′), 6.11 (br s, 1H, H-1), 6.27–6.73 (m, 1H, H-3_ind_), 7.13 (dddd, *J*_4″,F_ = 9.9, *J*_4″,5′′_ = 8.3, *J*_4″,2′′_ = 2.7, *J*_4″,6′′_ = 1.1 Hz, 1H, H-4″), 7.29–7.71 (m, 1H, H-5_ind_), 7.47 (td, *J*_5″,4′′_ = *J*_5″,6′′_ = 8.0, *J*_5″,F_ = 6.1 Hz, 1H, H-5″), 7.57 (dd, *J*_2_ind_,3_ind__ = 3.0, *J*_2_ind_,NH_ = 2.5 Hz, 1H, H-2_ind_), 7.61 (ddd, *J*_2″,F_ = 10.3, *J*_2″,4′′_ = 2.6, *J*_2″,6′′_ = 1.5 Hz, 1H, H-2″), 7.67 (ddd, *J*_6″,5′′_ = 7.7, *J*_6″,2′′_ = 1.5, *J*_6″,4′′_ = 1.0 Hz, 1H, H-6″), 8.11 (dd, *J*_7_ind_,3_ind__ = 1.3, *J*_7_ind_,5_ind__ = 0.9 Hz, 1H, H-7_ind_), 8.56 (s, 1H, H-5_Tz_), 11.42 (s, 1H, NH); ^13^C NMR (126 MHz, DMSO-*d*_6_, *T* = 100 °C): δ 19.21, 19.57, 19.58, 19.64, 19.79 (6 × **C**H_3_CO), 21.88 (**C**H_3_CON), 45.45 (CH-4), 51.12 (OCH_3_), 61.43 (CH_2_-6′), 61.73 (CH-3′), 62.59 (CH_2_-6), 66.32 (CH-2′), 68.50 (CH-4′), 69.98 (CH-2), 72.00 (CH-3), 73.92 (CH-5′), 74.21 (CH-5), 80.11 (CH-1), 81.13 (CH-1′), 111.40 (d, *J*_C,F_ = 23.0 Hz, CH-2″), 113.38 (CH-7_ind_), 114.04 (d, *J*_C,F_ = 21.3 Hz, CH-4″), 120.83 (*J*_C,F_ = 2.5 Hz, CH-6″), 121.04 (CH-5_Tz_), 124.47 (C-6_ind_), 128.57 (CH-5_ind_), 129.25 (C-4_ind_), 130.33 (d, *J*_C,F_ = 8.6 Hz, CH-5″), 132.41 (d, *J*_C,F_ = 8.5 Hz, C-1″), 136.00 (C-3a_ind_), 136.45 (C-7a_ind_), 144.84 (d, *J*_C,F_ = 2.5 Hz, C-4_Tz_), 162.18 (d, *J*_C,F_ = 243.6 Hz, CF-3″), 166.76 (COOCH_3_), 167.97, 168.65, 168.73, 169.28, 169.38 (6 × CH_3_CO), 169.57 (CH_3_ **C**ON); ^19^F NMR (471 MHz, DMSO-*d*_6_, *T* = 100 °C): δ −112.88 (ddd, *J*_F,2″_ = 10.3, *J*_F,4″_ = 8.9, *J*_F,5″_ = 6.1 Hz, 1F, F-3″).

##### *N*-[4-*S*-(3-Deoxy-3-(4-(3-fluorophenyl)-1*H*-1,2,3-triazol-1-yl)-β-d-galactopyranosyl)-4-thio-β-d-glucopyranosyl]-*N*-(3-acetyl-6-carboxy-1*H*-indol-4-yl)­acetamide (**10**)

In a sealed pressure tube, thiolactose **7** (0.252 g, 0.5 mmol) and 4-amino-6-(methylcarboxy)-1*H*-indole (0.140 g, 0.73 mmol) were dissolved in CH_3_OH (4 mL) and AcOH (0.01 mL). The solution was stirred at 80 °C for 4 h, cooled to room temperature, and concentrated in vacuo. The residue containing compound **8** was dissolved in Ac_2_O (2 mL, 21 mmol), followed by the addition of ZnCl_2_ (0.273 g, 2.0 mmol). The reaction mixture was stirred at 50 °C for 3 h, after which pyridine (5 mL, 62 mmol) was added. After stirring for further 2 h at room temperature, the mixture was concentrated in vacuo, and the residue was dissolved in EtOAc (20 mL) and washed with a saturated solution of NaHCO_3_ (5 mL) and brine (5 mL). The organic phase was separated and dried over anhydrous MgSO_4_, filtered, and concentrated in vacuo. The residue was purified by liquid column chromatography on silica gel (50% → 100% EtOAc in Cy) to give crude acetylated product (0.110 g, 22%).

The crude product (0.110 g, 0.109 mmol) was deprotected following general procedure C, and the methyl ester was hydrolyzed as described for **3af** to give product **10** (20.38 mg, 26%) as a white powder. The analytical sample was obtained by HPLC (A: H_2_O, B: CH_3_CN; 30% to 90% B in 30 min, followed by isocratic 90% B for 10 min). [α]_D_^20^ = +15.9 (*c* 0.2 in CH_3_OH); ^1^H NMR (500 MHz, methanol-*d*_4_, *T* = 25 °C): δ 1.85 (s, 3H, CH_3_CON), 2.55 (s, 3H, CH_3_CO-6_ind_), 2.64 (t, *J*_4,5_ = *J*_4,3_ = 10.8 Hz, 1H, H-4), 3.20 (dd, *J*_2,1_ = 9.2, *J*_2,3_ = 8.7 Hz, 1H, H-2), 3.49 (ddd, *J*_5,4_ = 11.0, *J*_5,6a_ = 4.6, *J*_5,6b_ = 2.1 Hz, 1H, H-5), 3.54 (dd, *J*_3,4_ = 10.6, *J*_3,2_ = 8.6 Hz, 1H, H-3), 3.61 (dd, *J*_gem_ = 11.5, *J*_6′a,5′_ = 5.2 Hz, 1H, H-6′a), 3.68 (dd, *J*_gem_ = 11.4, *J*_6′b,5′_ = 6.8 Hz, 1H, H-6′b), 3.75 (dd, *J*_gem_ = 12.1, *J*_6a,5_ = 4.6 Hz, 1H, H-6a), 3.80 (ddd, *J*_5′,6′b_ = 6.5, *J*_5′,6′a_ = 5.2, *J*_5′,4′_ = 1.1 Hz, 1H, H-5′), 3.84 (dd, *J*_gem_ = 12.0, *J*_6b,5_ = 2.0 Hz, 1H, H-6b), 4.07 (dd, *J*_4′,3′_ = 3.0, *J*_4′,5′_ = 1.1 Hz, 1H, H-4′), 4.24 (dd, *J*_2′,3′_ = 10.7, *J*_2′,1′_ = 9.3 Hz, 1H, H-2′), 4.61 (d, *J*_1′,2′_ = 9.4 Hz, 1H, H-1′), 4.83 (dd, *J*_3′,2′_ = 10.7, *J*_3′,4′_ = 3.0 Hz, 1H, H-3′), 5.78 (d, *J*_1,2_ = 9.3 Hz, 1H, H-1), 7.06 (tdd, *J*_4″,F_ = *J*_4″,5′′_ = 8.5, *J*_4″,2′′_ = 2.6, *J*_4″,6′′_ = 1.0 Hz, 1H, H-4″), 7.43 (td, *J*_5″,6′′_ = *J*_5″,4′′_ = 8.0, *J*_5″,F_ = 6.0 Hz, 1H, H-5″), 7.57 (ddd, *J*_2″,F_ = 10.2, *J*_2″,4′′_ = 2.6, *J*_5″,6′′_ = 1.5 Hz, 1H, H-2″), 7.63 (ddd, *J*_6″,5′′_ = 7.6, *J*_6″,4′′_ = 1.6, *J*_6″,2′′_ = 1.0 Hz, 1H, H-6″), 8.01 (d, *J*_5_ind_,7_ind__ = 1.4 Hz, 1H, H-5_ind_), 8.25 (d, *J*_7_ind_,5_ind__ = 1.4 Hz, 1H, H-7_ind_), 8.38 (s, 1H, H-2_ind_), 8.44 (s, 1H, H-5_Tz_); ^13^C NMR (126 MHz, methanol-*d*_4_, *T* = 25 °C): δ 24.29 (**C**H_3_CON), 28.58 (**C**H_3_CO-6_ind_), 46.56 (CH-4), 62.30 (CH_2_-6′), 63.51 (CH_2_-6), 67.54 (CH-2′), 68.43 (CH-3′), 69.75 (CH-4′), 73.18 (CH-2), 77.25 (CH-3), 80.43 (CH-5), 81.33 (CH-5′), 84.99 (CH-1), 87.05 (CH-1′), 113.21 (d, *J*_C,F_ = 23.3 Hz, CH-2″), 115.72 (d, *J*_C,F_ = 21.4 Hz, CH-4″), 116.25 (CH-7_ind_), 120.33 (C-3_ind_), 122.40 (d, *J*_C,F_ = 2.7 Hz, CH-6″), 122.52 (CH-5_Tz_), 126.61 (CH-5_ind_), 127.09 (C-6_ind_), 129.93 (C-3a_ind_), 131.83 (d, *J*_C,F_ = 8.5 Hz, CH-5″), 132.65 (C-4_ind_), 134.34 (d, *J*_C,F_ = 8.5 Hz, C-1″), 139.14 (CH-2_ind_), 140.05 (C-7a_ind_), 147.11 (d, *J*_C,F_ = 2.5 Hz, C-4_Tz_), 164.66 (d, *J*_C,F_ = 244.2 Hz, CF-3″), 169.69 (**C**OOH), 175.04 (CH_3_ **C**ON), 195.29 (CH_3_ **C**O-6_ind_); ^19^F NMR (471 MHz, DMSO-*d*_6_, *T* = 100 °C): δ −114.86 (td, *J*_F,2″_ = *J*_F,4″_ = 9.4, *J*_F,5″_ = 6.1 Hz, 1F, F-3″); IR (CH_3_OH): 3320, 3200, 2939, 2885, 2836, 2599, 2461, 1700, 1662, 1623, 1589, 1566, 1566, 1525, 1506, 1486, 1457, 1424, 1265, 1085, 1052, 980, 865, 788, 774, 750, 720, 687, 526 cm^–1^; HRMS (ESI): [M – H]^−^ *m*/*z* calcd for C_33_H_35_O_12_N_5_FS: 744.1992, found: 744.1996.

##### *N*-[4-*S*-(3-Deoxy-3-(4-(3-fluorophenyl)-1*H*-1,2,3-triazol-1-yl)-β-d-galactopyranosyl)-4-thio-β-d-glucopyranosyl]-*N*-(6-carboxy-1*H*-indol-4-yl)­acetamide (**11**)

The indole derivative **9** (0.080 g, 0.0825 mmol) was deprotected following general procedure C, and the methyl ester was hydrolyzed as described for **3af** to give product **11** (11.32 mg, 20%) as a white powder. The analytical sample was obtained by HPLC (A: H_2_O, B: CH_3_CN; 30% to 90% B in 30 min, followed by isocratic 90% B for 10 min). [α]_D_^20^ = +36.0 (*c* 0.1 in CH_3_OH); signals of the major conformer: ^1^H NMR (500 MHz, methanol-*d*_4_, *T* = 25 °C): δ 1.77 (s, 3H, CH_3_CON), 2.63 (t, *J*_4,5_ = *J*_4,3_ = 10.8 Hz, 1H, H-4), 3.15 (dd, *J*_2,1_ = 9.4, *J*_2,3_ = 8.5 Hz, 1H, H-2), 3.57 (dd, *J*_3,4_ = 10.6, *J*_3,2_ = 8.4 Hz, 1H, H-3), 3.60–3.68 (m, 2H, H-5,6′a), 3.75 (dd, *J*_gem_ = 11.4, *J*_6′b,5′_ = 6.8 Hz, 1H, H-6′b), 3.80 (dd, *J*_gem_ = 12.2, *J*_6a,5_ = 5.3 Hz, 1H, H-6a), 3.82–3.86 (m, 1H, H-5′), 4.07 (dd, *J*_4′,3′_ = 3.1, *J*_4′,5′_ = 1.0 Hz, 1H, H-4′), 4.15 (dd, *J*_gem_ = 12.1, *J*_6b,5_ = 1.7 Hz, 1H, H-6b), 4.23 (dd, *J*_2′,3′_ = 10.8, *J*_2′,1′_ = 9.2 Hz, 1H, H-2′), 4.65 (d, *J*_1′,2′_ = 9.4 Hz, 1H, H-1′), 4.84 (dd, *J*_3′,2′_ = 10.7, *J*_3′,4′_ = 3.1 Hz, 1H, H-3′), 5.86 (d, *J*_1,2_ = 9.5 Hz, 1H, H-1), 6.75–6.81 (m, 1H, H-3_ind_), 7.06 (tdd, *J*_4″,F_ = *J*_4″,5′′_ = 8.4, *J*_4″,2′′_ = 2.6, *J*_4″,6′′_ = 0.9 Hz, 1H, H-4″), 7.43 (td, *J*_5″,6′′_ = *J*_5″,4′′_ = 8.1, *J*_5″,F_ = 5.9 Hz, 1H, H-5″), 7.49–7.53 (m, 1H, H-2_ind_), 7.54–7.60 (m, 1H, H-2″), 7.63 (ddd, *J*_6″,5′′_ = 7.8, *J*_6″,4′′_ = 1.7, *J*_6″,2′′_ = 0.9 Hz, 1H, H-6″), 7.90 (d, *J*_5_ind_,7_ind__ = 1.4 Hz, 1H, H-5_ind_), 8.23 (dd, *J*_7_ind_,5_ind__ = 1.4, *J*_7_ind_,NH_ = 0.9 Hz, 8H), 8.43 (s, 1H, H-5_Tz_), 11.22–11.27 (m, 1H, NH_ind_); ^13^C NMR (126 MHz, methanol-*d*_4_, *T* = 25 °C): δ 23.01 (**C**H_3_CON), 47.19 (CH-4), 62.44 (CH_2_-6′), 63.89 (CH_2_-6), 67.69 (CH-2′), 68.53 (CH-3′), 69.79 (CH-4′), 72.63 (CH-2), 77.21 (CH-3), 80.42 (CH-5), 81.28 (CH-5′), 84.17 (CH-1), 86.93 (CH-1′), 102.93 (CH-3_ind_), 113.19 (d, *J*_C,F_ = 23.2 Hz, CH-2″), 115.54 (CH-7_ind_), 115.73 (d, *J*_C,F_ = 21.4 Hz, CH-4″), 122.4 (2 × C, CH-5_Tz_, CH-6″), 123.45 (CH-5_ind_), 124.82 (C-6_ind_), 130.69 (CH-2_ind_), 130.93 (C-4_ind_), 131.85 (d, *J*_C,F_ = 8.5 Hz, CH-5″), 133.30 (C-3a_ind_), 134.32 (d, *J*_C,F_ = 8.5 Hz, C-1″), 138.43 (C-7a_ind_), 147.12 (d, *J*_C,F_ = 1.3 Hz, C-4_Tz_), 164.66 (d, *J*_C,F_ = 244.1 Hz, CF-3″), 170.70 (**C**OOH), 175.28 (CH_3_ **C**ON); ^19^F NMR (471 MHz, DMSO-*d*_6_, *T* = 100 °C): δ −113.06 (ddd, *J*_F,2″_ = 10.3, *J*_F,4″_ = 8.9, *J*_F,5″_ = 6.1 Hz, 1F, F-3″); IR (CH_3_OH): 3336, 3140, 2929, 2885, 2856, 2640, 1690, 1653, 1621, 1589, 1576, 1560, 1512, 1487, 1457, 1437, 1410, 1265, 1086, 1047, 982, 865, 786, 751, 718, 686, 526 cm^–1^; HRMS (ESI): [M – H]^−^ *m*/*z* calcd for C_31_H_33_O_11_N_5_FS: 702.1887, found: 702.1890.

### Protein Preparation and Purification

Both galectin-1 and galectin-3 CRD genes were amplified from commercial plasmid pCMV6-LGALS1 (Origene; #MR200810) and cloned into pET-15b plasmid with an N-terminal 6× His-tag and sequence recognized by TEV protease (tobacco etch virus nuclear-inclusion–an endopeptidase). Plasmids were transformed into BL21 *Escherichia coli*. Freshly transformed bacteria were grown overnight at 37 °C on LB medium (10 g/L tryptone, 5 g/L yeast extract, 5 g/L NaCl) agar plates supplemented with ampicillin (100 μg/mL). Grown colonies were used to inoculate a total volume of 5.5 L of LB medium each (supplemented with ampicillin) and cultured for 2 to 3 h until reaching optical density of *A*_600_ ≃0.5. Protein expression was induced by the addition of ethyl-β-d-thiogalactopyranoside to the culture (final concentration 1 mM). The bacterial cultures were further incubated overnight at 16 °C and then pelleted by centrifugation (4000*g*, 4 °C, 10 min). Bacterial pellets were resuspended in PBS buffer, supplemented with 5 mM β-mercaptoethanol and 1 mM PMSF. The cell suspension was processed 3 times using an Avestin Emulsiflex C3 at a disruption peak pressure of 1000 psi. Bacterial lysates were centrifuged for 30 min (10,000*g*, 4 °C).

Galectins were purified using Ni^2+^-affinity chromatography columns (HisTrap HP, Cytiva), with the His tag cleaved by TEV protease between two rounds of purification, in crystallization buffer (PBS with 5 mM β-mercaptoethanol). To obtain well-folded functional protein, affinity chromatography using α-lactose-agarose (Sigma-Aldrich) was performed. Proteins were eluted using a lactose gradient and then extensively dialyzed against crystallization buffer. Final purity was achieved by size exclusion chromatography (Superdex 75 column, Cytiva), typically yielding ∼90 mg of pure protein from 1 L of bacterial culture.

To prepare full-length galectin-3, the DNA sequence of galectin-3 glycodomain (AA 1–250) was amplified by PCR (oligonucleotides pET_Gal3_F 5′-AAGAAGGAGATATACATATGGCAGACAATTTTTCGCT-3′ and pET_Gal3_R 5′-GGTGCTCGAGTGCGGCCGCATCATATCATGGTATATGAAGCAC-3′) from plasmid LGALS3 (Origene; RC208785) and subcloned into *Nde*I and *Hin*dIII restricted vector pET-22b­(+) using Gibson assembly. Protein expression and purification followed a previously described procedure.^33^

### Fluorescence Polarization Assay

#### Direct Binding Experiments

Fluorescently labeled probe 3-deoxy-3-[4-(fluorescein-5-yl-carbonylaminomethyl)-1*H*-1,2,3-triazol-1-yl]-β-d-galactopyranosyl-3-deoxy-3-[4-(3-fluorophenyl)-1*H*-1,2,3-triazol-1-yl]-1-thio-β-d-galactopyranoside was synthesized as previously described.^36,49^ To determine the affinity of this probe for Gal-1 and Gal-3C, proteins were titrated against a fixed probe concentration in PBS containing 4% DMSO and 0.01% Triton X-100. Fluorescence polarization measurements were performed at 25 °C using a Tecan Spark multiplate reader (Tecan, Switzerland) with excitation at 485 ± 20 nm and emission at 535 ± 20 nm in a 384-well black polystyrene microplate (Corning 4514, USA). Dissociation constants of the probe (*K*_d_^*^) were determined by fitting the binding data with a quadratic model for direct binding using the BindCurve package.^50^ Additional details on the model, probe concentrations, and fitting parameters are provided in the Supporting Information.

#### Competitive Binding Experiments (Determination of Inhibitor Affinities)

Fluorescence polarization experiments for determining inhibitor affinities were performed using the same instrumentation and settings as described for the direct binding experiments. Inhibitors were dissolved at 10 mM concentration in Hybri-Max DMSO (Merck, Germany), serially diluted in Echo 384-well polypropylene plates using a Bravo automated pipetting system (Agilent, USA), and then prespotted into 384-well plates (Corning 4514, USA) using an Echo 650 acoustic liquid handler (Beckman Coulter, USA). The galectin–probe mixture was subsequently added using a Certus Flex liquid handler (Fritz Gyger AG, Switzerland) to reach a final volume of 5 μL. Anisotropy was measured after 30–60 min incubation at 25 °C. Inhibitor dissociation constants (*K*_d_) were calculated using Wang’s exact cubic model^51^ for competitive binding implemented in the BindCurve package.^50^ Protein and probe concentrations used in these competitive experiments are provided in the Supporting Information.

### X-ray Crystallography

#### Crystallization

For crystallization of compound **3r** with Gal-3C, the protein was kept in PBS crystallization buffer supplemented with 5 mM β-mercaptoethanol and concentrated to approximately 15 g/L. Initial crystallization trials were performed using the sitting drop vapor diffusion technique with the JCSG++ HTS screen (Jena Bioscience). Drops were set in a 1:1 ratio of protein/precipitant with a total volume of 0.4 mL. Gal-3C crystallized in JCSG++ HTS G10 (150 mM potassium bromide, 30% w/v polyethylene glycol MME 2000). Crystals appeared within approximately 1 week and were used to prepare seed stocks.

Final crystals of Gal-3C were grown by sitting drop vapor diffusion. Drops (2 mL) were prepared by mixing protein solution with precipitating condition and seed stock in 1:0.8:0.2 ratio. Grown crystals were soaked overnight in a 10 mM solution of **3r** in the precipitating solution. For cryoprotection, crystals were briefly soaked for a few seconds in reservoir solution with concentration of PEG 2000 MME raised to 35%, flash-cooled by plunging into liquid nitrogen, and then stored in liquid nitrogen until used for the X-ray diffraction experiments.

#### Data Collection and Structure Determination

Diffraction data were collected at 100 K on the MX14.1 beamline at the BESSY synchrotron radiation facility (Berlin, Germany) at a wavelength of 0.9184 Å.^52^ Data integration and reduction were performed using XDS^53^ with the XDSGUI^54^ graphical interface. The crystal exhibited the symmetry of space group *P*2_1_2_1_2_1._ The structure was solved by the molecular replacement method using MOLREP^55^ with coordinates from the identical protein (PDB: 5ODY). Refinement was performed using REFMAC 5.5,^56,57^ and model building was carried out in Coot.^58^ Crystal parameters, data collection statistics, and refinement statistics are summarized in the Supporting Information. The quality of the final model was validated using MolProbity.^59^ Atomic coordinates and experimental structure factors have been deposited to the Protein Data Bank under the accession code 9S62.

### In Vitro ADME and Pharmacokinetics

#### Plasma Stability Assay

Test compounds (10 μM) were incubated with mouse, rat, or human pooled plasma from 50 donors (Biowest) at 37 °C for 20, 60, and 120 min. Reactions were terminated by adding four volumes of ice-cold methanol, followed by vigorous mixing. Samples were kept at −20 °C for 30 min and then stored at 4 °C overnight. The next day, samples were centrifuged, and supernatants were diluted with four volumes of 30% methanol in water. Zero time points were prepared by adding ice-cold methanol to the compound prior to the addition of plasma. Samples were then analyzed on an Echo MS system (SCIEX) using multiple reaction monitoring (MRM). The only exception was compound **3af**, which was analyzed by LC–MS/MS (SCIEX 6500 triple quadrupole) due to its weak ionization in Echo-MS.

#### Microsomal Stability Assay

Microsomal stability was determined using 0.5 mg/mL of pooled human, mouse, or rat liver microsomes (Thermo Fisher Scientific) in 90 mM TRIS-Cl buffer (pH 7.4) containing 2 mM NADPH and 2 mM MgCl_2_. Test compounds (10 μM) were added, and the resulting mixture was incubated at 37 °C for 10, 60, 120, and 180 min. The reactions were terminated by the addition of four volumes of ice-cold methanol, followed by vigorous mixing. The samples were left at −20 °C for 30 min and then stored at 4 °C overnight. The next day, samples were centrifuged, and supernatants were diluted with four volumes of 30% methanol in water. Zero time points were prepared by adding ice-cold methanol to the mixture of compounds and cofactors prior to the addition of microsomes. The samples were then analyzed using the Echo-MS system (SCIEX) with MRM transition monitoring, except for compound **3af**, which was analyzed by LC–MS/MS as described in the plasma stability assay.

#### Plasma Protein Binding Assay

Test compound (10 μM) was incubated at 37 °C for 4 h with mouse, rat, or human plasma (Biowest) in the donor well of a rapid equilibrium dialysis plate (Thermo Fisher Scientific). PBS was placed in the acceptor well. To stop the assay, samples were collected from both wells, and an equal volume of plasma was added to the PBS sample, and vice versa. Proteins were then precipitated by adding three volumes of ice-cold methanol. Samples were mixed gently, kept at −20 °C for 30 min, and then stored at 4 °C overnight. The next day, the samples were centrifuged, and the supernatants were diluted with four volumes of 30% methanol in water. The samples were then analyzed using the Echo-MS system (SCIEX) with MRM transition monitoring, except for compound **3af**, which was analyzed by LC–MS/MS as described in the plasma stability assay.

#### Solubility Assay

Kinetic solubility was determined by diluting the test compound stocks in DMSO with PBS to achieve a final compound concentration of 100 μM. The solutions were incubated at room temperature for 2 h and filtered by centrifugation. Filtrates were diluted 100-fold with 50% acetonitrile in water and then analyzed on the Echo-MS system (SCIEX) using MRM transition monitoring, except for compound **3af**, which was analyzed by LC–MS/MS as described in the plasma stability assay. Compound concentrations were quantified against calibration curves prepared in the identical matrix as the samples.

### Cell-Based Assays

#### Cell Cultivation

Human HEK293, HepG2, THP1, LX2, and Jurkat E6.1 cells were used for in vitro studies. HEK293 (ATCC CRL-1573) and HepG2 (ATCC HB-8065) cells were used as a general screening platform and were cultured in minimum essential medium (MEM; Thermo Fisher Scientific, Gibco), supplemented with 10% (v/v) fetal bovine serum (FBS; Thermo Fisher Scientific, Gibco), 1 mM sodium pyruvate (Thermo Fisher Scientific, Gibco), 1× MEM nonessential amino acid (Thermo Fisher Scientific, Gibco), and 1% (v/v) penicillin–streptomycin (P/S, Thermo Fisher Scientific, Gibco). THP1 cells (kindly provided by the group of Dr. Maletinska, IOCB Prague, Czech Republic) were cultured in RPMI-1640 (Thermo Fisher Scientific, Gibco) supplemented with 5% (v/v) FBS, 2.5 g/L glucose (Thermo Fisher Scientific, Gibco), 2.38 g/L HEPES (Thermo Fisher Scientific, Gibco), 0.11 g/L sodium pyruvate, 1% (v/v) P/S, and 0.05 mM 2-mercaptoethanol (Thermo Fisher Scientific, Gibco). Jurkat E6.1 cells (ATCC TIB-152) were cultured in RPMI-1640 (Thermo Fisher Scientific, Gibco) supplemented with 10% (v/v) FBS, 2.5 g/L glucose, 2.38 g/L HEPES, 0.11 g/L sodium pyruvate, and 1% (v/v) P/S. LX2 cells (kindly provided by the group of Dr. Maletinska, IOCB Prague, Czech Republic) were cultured in Dulbecco’s modified Eagle’s medium (DMEM; Thermo Fisher Scientific, Gibco) supplemented with 2% (v/v) FBS and 1% (v/v) P/S. THP1 and LX2 cells were used for downstream functional assays to explore the immunomodulatory and antifibrotic properties of **11** (see below). Jurkat E6.1 cells were also used in the cytotoxicity study (see below) as an additional immune cell model complementary to THP1. All cells were cultivated in their respective media at 37 °C at 5% CO_2_ atmosphere (referred to as standard conditions).

#### Cytotoxicity Assay

To evaluate in vitro cytotoxicity and support its potential application in inflammatory and fibrotic disease models, the following panel of cell lines, representing the relevant biological systems, was used: HEK293, HepG2, THP1, Jurkat E6.1, and LX2. HEK293, HepG2, and Jurkat E6.1 cells were seeded at 400,000 cells/mL, THP1 cells at 500,000 cells/mL, and LX2 cells at 200,000 cells/mL in 96-well plates (cell culture-treated, VWR) in 100 μL/well. Cells were treated with the tested compounds in a serial dilution (300, 100, 33.3, 11.1, and 3.7 μM) for 24 h. Untreated cells served as negative controls to establish the baseline viability threshold. Cell viability was assessed using a viability dye (Zombie Violet Fixable Viability Kit, BioLegend) following the manufacturer’s instructions and analyzed by flow cytometry (see below). The concentration of compound that reduced cell viability by 50% (CC_50_) was determined by nonlinear regression analysis using GraphPad Prism 10 (San Diego).

#### Determination of Gal-3 Target Engagement

THP1 cells were used to assess the binding of the tested compounds to Gal-3. Cells were either maintained in suspension as undifferentiated monocyte-like cells or differentiated into macrophage-like cells by overnight treatment with phorbol 12-myristate 13-acetate (PMA, Sigma-Aldrich) at a final concentration of 10 ng/mL. Cells were then washed with PBS (Gibco, Thermo Fisher Scientific) and treated with compounds in a serial dilution (300, 100, 33.3, 11.1, and 3.7 μM) for 2 h. Untreated cells served as negative and baseline controls representing maximum intracellular Gal-3 levels. Cells were then harvested and intracellularly stained either with galectin-3 monoclonal antibody eF660 (Invitrogen, Thermo Fisher Scientific) or a relevant isotype antibody (Invitrogen, Thermo Fisher Scientific) for flow cytometric analyses (see below).^60^ A dose-dependent decrease in Gal-3 signal upon compound treatment was used to assess intracellular target engagement expressed as median fluorescent intensity (MFI).

#### Anti-inflammatory Potential Testing

To evaluate the anti-inflammatory potential of **11** in comparison with **GB0139**, THP1 human monocytic cells were seeded at 500,000 cells/mL into 96-well plates (100 μL/well). Cells were differentiated overnight into macrophage-like phenotypes by treatment with 10 ng/mL PMA and subsequently stimulated with 1 μg/mL lipopolysaccharide (LPS; Invitrogen, Thermo Fisher Scientific) to induce a proinflammatory response. Simultaneously, cells were cotreated with the tested compounds **11** or **GB0139** in a serial dilution (300, 100, 33.3, 11.1, and 3.7 μM). After 24 h of LPS and compound exposure, cell culture supernatants were collected and analyzed for TNFα levels using a bead-based multiplex immunoassay (ProcartaPlex Human Basic Kit and TNFα Human ProcartaPlex Simplex Kit, both Invitrogen, Thermo Fisher Scientific). Flow cytometry readout was used as previously described.^61^ TNFα concentrations were calculated using a standard curve generated in GraphPad Prism 10 (GraphPad Software, San Diego, CA).

#### Flow Cytometry Assay Readout

Cells or beads from screening assays prepared for flow cytometry were analyzed by the CytoFlex S instrument (Beckman Coulter). Data acquisition was performed using CytExpert software (v2.5, Beckman Coulter). Debris was excluded by forward and side scatter gating, followed by doublet exclusion. Data were analyzed using FlowJo software (v10, BD Biosciences).

#### Antifibrotic Efficacy

To assess the antifibrotic potential of **11** in comparison with the reference inhibitor **GB0139**, LX2 human hepatic stellate cells were seeded at 200,000 cells/mL into 96-well plates (100 μL/well) and allowed to adhere overnight. LX2 cells were then stimulated with transforming growth factor-β1 (TGFβ1; PeproTech, Thermo Fisher Scientific) at 5 ng/mL to induce profibrotic signaling. Compounds (**11** or **GB0139**) were added simultaneously with TGFβ1 stimulation in a serial dilution (300, 100, 33.3, 11.1, and 3.7 μM). After 48 h of treatment, cells were harvested into 100 μL RA1 buffer, and total RNA was then isolated using the NucleoSpin 96 RNA Kit (Macherey-Nagel, Düren, Germany) and reverse-transcribed into cDNA using the LunaScript RT SuperMix Kit (New England Biolabs, Ipswich, MA, USA).

Quantitative real-time PCR (RT-qPCR)^62^ was used to determine mRNA expression levels of selected profibrotic markers: αSMA (*ACTA2*), collagen α-2­(I) chain (*COL1A2*), and fibronectin 1 (*FN1*), and the housekeeping gene β-actin (*ACTB*). Amplification was carried out using Luna Universal qPCR Master Mix (New England Biolabs) and primer pairs for individual genes as follows: *ACTA2*: forward primer: 5′-AATGCAGAAGGAGATCACGG-3′, reverse primer: 5′-TCCTGTTTGCTGATCCACATC-3′; *COL1A2*: forward primer: 5′-AGGACAAGAAACACGTCTGG-3′, reverse primer: 5′-GGTGATGTTCTGAGAGGCATAG-3′; *FN1*: forward primer: 5′-ACTGTACATGCTTCGGTCAG-3′, reverse primer: 5′-AGTCTCTGAATCCTGGCATTG-3′; *ACTB*: forward primer: 5′-AAGGAGATCACTGCCCTGGC-3′, reverse primer: 5′-CCACACGGAGTACTTGCGC-3′ (Eurofins Genomics). Data were normalized to *ACTB* expression and analyzed using the ΔΔCt method.

### ^19^F NMR Experiments

^19^F NMR titration spectra of compound **11** were recorded on a Bruker Avance III HD 500 MHz spectrometer (^1^H at 500.0 MHz, ^19^F at 470.4 MHz). Hexafluorobenzene (δ −164.9 ppm) was used as an external standard for referencing ^19^F chemical shifts. A stock solution of compound **11** was prepared in D_2_O. Titrations were performed in 5 mm NMR tubes by incrementally adding aliquots of the stock solution to a 200 μM solution of Gal-3C in PBS (pH 7.5), covering protein/ligand ratios ranging from 1:0.25 to 1:1.5. This setup ensured detection of a distinct signal corresponding to the excess unbound ligand. Additionally, a control spectrum of compound **11** in PBS containing 10 μL of D_2_O was recorded to confirm the chemical shift of the free ligand in the titration series.

### Molecular Modeling

The PDB 5H9P structure was used as a model of the receptor. The protein was prepared using the Protein Preparation Workflow in the Schrödinger suite (version 2025-1) and then further modified with PDBFixer. Initial ligand geometries were built based on the X-ray conformation of **3r** (PDB 9S62).

MD simulations were performed in OpenMM 8.2.0.^63^ The protein was parametrized with the ff14SB^64^ force field, and small molecules were parametrized with gaff-2.11.^65^ For each simulation, the system was placed in a cubic box with a 10 Å buffer and solvated with TIP3P water. Long-range interactions were treated using the particle-mesh Ewald method with a 10 Å cutoff. All bonds involving hydrogens were constrained. The system was first equilibrated for 0.5 ns under *NVT* conditions using a Langevin thermostat (300 K), followed by 0.5 ns of further equilibration under *NPT* conditions with a Monte Carlo barostat (1 bar). Production simulations comprised three replicas of 1000 ns each (total simulation time 3000 ns per system) with a 3 fs time step and frames saved every 10,000 steps. The resulting trajectories were processed in MDTraj 1.9.8.^66^ The three replicas for each system were combined, imaged, and superposed to the initial conformation via the alignment of C_α_ protein atoms. Equally spaced samples (30 for visualization, 10,000 for dPCA) were extracted from the combined trajectory for downstream processing. RMSD values were calculated between ligand conformations in the target trajectory and the reference conformation of the ligand, which was built directly from the X-ray conformation of **3r**.

A representative frame from the simulation of compound **11** in complex with Gal-3C was manually selected and optimized by QM/MM using the QSite program^67,68^ from the Schrödinger suite (v2025-1). The ligand was modeled at the level of B3LYP-D3­(BJ) with the LACVP* basis set, and the protein was treated using the OPLS4 force field.

dPCA was conducted using scikit-learn.^69^ Four exocyclic dihedral angles were chosen to represent the conformation of the ligands **3a**, **3r**, **3af**, and **11** (see the Supporting Information). These dihedral angles were transformed into their sine and cosine values to handle periodicity,^70^ and the transformed angles of all four ligands were used to fit the principal components (dPC1 and dPC2). The dihedrals of the individual ligands were then transformed using the same dPCA transformation.

## Supplementary Material





## Notes

Fluorescence polarization, ADME data, synthetic procedures, NMR spectra, and HPLC traces (PDF)
Molecular formula strings (CSV)

## Abbreviations

αSMA: α-smooth muscle actin
ACTA2: gene encoding α-smooth muscle actin
ACTB: gene encoding β-actin
CC_50_: cytotoxic concentration at which 50% of cells are viable
CI: confidence interval
CL_int_: intrinsic clearance
COL1A2: gene encoding collagen type 1 chain α2
CRD: carbohydrate recognition domain
dPCA: dihedral principal component analysis
ECM: extracellular matrix
*F*_b_: fraction bound
FN1: gene encoding fibronectin 1
Gal: galectin
HRMS: high-resolution mass spectrometry
IPF: idiopathic pulmonary fibrosis
IR: infrared spectroscopy
LPS: lipopolysaccharide
MD: molecular dynamics
MFI: median fluorescence intensity
NMR: nuclear magnetic resonance
PMA: phorbol 12-myristate 13-acetate
qPCR: quantitative polymerase chain reaction
RMSD: root-mean-square deviation
RT-qPCR: reverse transcription-quantitative polymerase chain reaction
SD: standard deviation
SEM: standard error of the mean
TGFβ: transforming growth factor-β
TLC: thin-layer chromatography
TLR: Toll-like receptor
TNFα: tumor necrosis factor-α
