# Supplementary material for: N‑Aryl‑N‑Lactosylamides as Potent and Highly Selective Inhibitors of Galectin‑3 with Antifibrotic Activity
Source: J Med Chem. 2025 Nov 11;68(22):24624–48. doi: 10.1021/acs.jmedchem.5c02604 (PMC12670426; doi:10.1021/acs.jmedchem.5c02604)
Supplement: Supplementary file 1 [file jm5c02604_si_001.pdf]

Supporting Information

N-Aryl-*N*-lactosylamides as Potent and Highly Selective Inhibitors of Galectin-3 with Antifibrotic Activity

Jakub Zýka[1,2], Jaroslav Kozák[1], Lenka Vanekova[3], Marketa Pimkova Polidarova[3], Vít Prouza[1,2], Nina Habanová[1], Timotej Strmeň[1,4], Martin Zavřel[1], Petr Pachl[1], Jan Choutka[1,*], Klara Grantz Saskova[3], Andrea Brazdova[3,§,*], Kamil Parkan[1,2,§,*], and Radek Pohl[1,§]

[1] Institute of Organic Chemistry and Biochemistry of the Czech Academy of Sciences, Gilead Sciences & IOCB Research Centre, Flemingovo náměstí 2, 166 10 Prague, Czech Republic

[2] Department of Chemistry of Natural Compounds, University of Chemistry and Technology Prague, Technická 5, 166 28 Prague, Czech Republic

[3] Department of Genetics and Microbiology, Faculty of Science, Charles University, BIOCEV, Průmyslová 595, 252 50 Vestec, Czech Republic

[4] Department of Public Health and Clinical Medicine, Umeå University, 901 87 Umeå, Sweden

[§] These authors share senior authorship

* Corresponding authors

Emails: jan.choutka@uochb.cas.cz, andrea.brazdova@natur.cuni.cz, kamil.parkan@vscht.cz

Contents

Fluorescence polarization 3
Direct binding 3
Competitive binding 5
X-ray crystallographic data for the Gal-3C:**3r** complex 7
Metabolic stability, toxicity, and solubility 8
Molecular modeling 11
Synthetic procedures of precursor compounds 12
NMR spectra of prepared compounds 35
Representative HPLC traces for key investigated compounds........ 138
References 144

Fluorescence polarization

Direct binding

Direct binding was modeled by a quadratic model, which is written as

$$r = r_{min} + (r_{max} - r_{min})\frac{[R]}{K_d^* + [R]} \tag{1}$$

where r corresponds to the measured anisotropy, r_{min} corresponds to the anisotropy of the free probe (labeled ligand), and r_{max} corresponds to maximal anisotropy, which is achieved when all molecules of the probe are bound to the receptor. K_d^* is the dissociation constant of the probe and $[R]$ is the concentration of free receptor. $[R]$ is determined by a quadratic equation

$$[R]^2 + a[R] + b = 0 \tag{2}$$

The coefficients are defined as

$$a = K_d^* + [L^*]_T - [R]_T \tag{3}$$

$$b = -K_d^*[R]_T \tag{4}$$

where $[L^*]_T$ is the total concentration of the probe and $[R]_T$ is the total concentration of the receptor. Using quadratic formula, the positive root is calculated as

$$[R] = \frac{-a + \sqrt{a^2 - 4b}}{2} \tag{5}$$

In the direct binding experiments, the value of $[L^*]_T$ is known and $[R]_T$ is titrated. The remaining variables K_d^*, r_{min} and r_{max} are fitted. The structure of the fluorescent probe is depicted in **Figure S1**, the fitted variables K_d^*, r_{min} and r_{max} are summarized in **Table S1**, and the direct binding curves of the probe are shown in **Figure S2**.

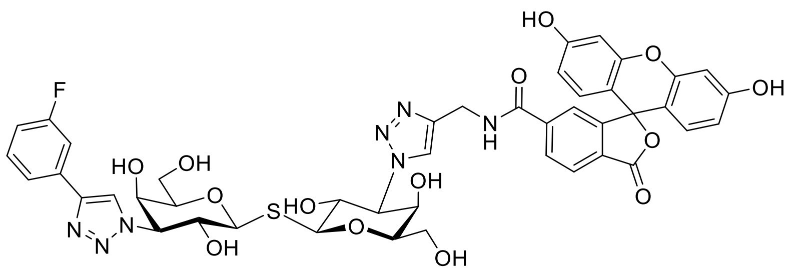

Figure S1. Structure of the fluorescent probe *3-deoxy-3-[4-(fluorescein-5-yl-carbonylaminomethyl)-1H-1,2,3-triazol-1-yl]-β-D-galactopyranosyl 3-deoxy-3-[4-(3-fluorophenyl)-1H-1,2,3-triazol-1-yl]-1-thio-β-D-galactopyranoside* used in fluorescence polarization assays.[1, 2]

Table S1. Calculated K_d^* values, total concentrations of the fluorescent probe, and model asymptotes from direct binding experiments.

	K_d^* (nM)[a]	[probe] (nM)	r_{min} (mA)	r_{max} (mA)
Gal-3C	22 ± 2[a]	5	25	130
Gal-1	740 ± 9[a]	100	20	91

[a] K_d^* values are expressed as mean ± SD from two independent experiments, each performed in triplicate.

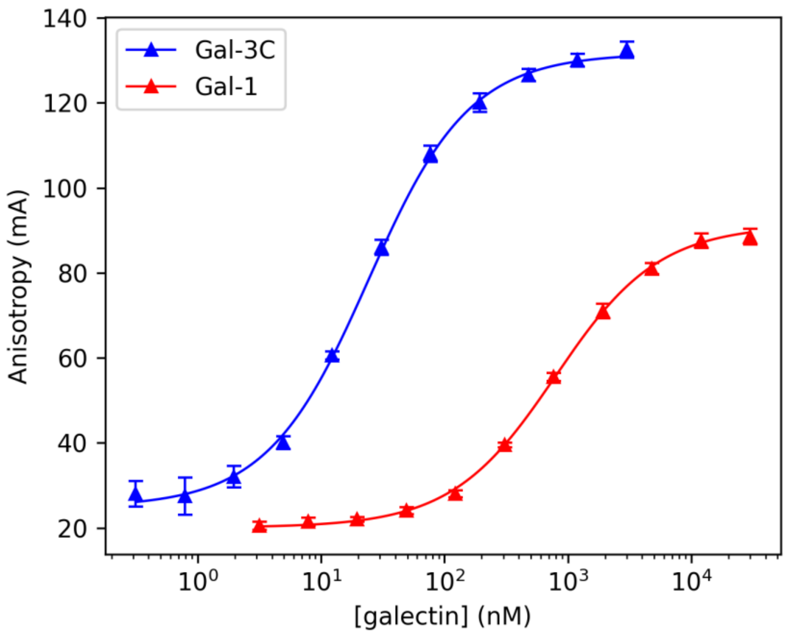

Figure S2. Direct binding curves of the fluorescent probe with Gal-1 and Gal-3C obtained by fluorescence polarization. Two independent experiments were performed, each in triplicate. Error bars indicate SD for each concentration point.

Competitive binding

Competitive binding was modeled by Wang's exact cubic model,[3] which is written as

$$r = r_{min} + (r_{max} - r_{min}) \frac{2\sqrt{(a^2 - 3b)}\cos\frac{\theta}{3} - a}{3K_d^* + 2\sqrt{(a^2 - 3b)}\cos\frac{\theta}{3} - a} \quad (6)$$

where r, r_{min} and r_{max} have the same meaning as in **Equation 1**. The coefficients a, b, and c are defined as

$$a = K_d^* + K_d + [L^*]_T + [L]_T - [R]_T \quad (7)$$

$$b = K_d^*([L]_T - [R]_T) + K_d([L^*]_T - [R]_T) + K_d^* K_d \quad (8)$$

$$c = -K_d^* K_d [R]_T \quad (9)$$

where K_d^* is the dissociation constant of the probe, K_d is the dissociation constant of the unlabeled ligand, $[L^*]_T$ is the total concentration of the probe, $[L]_T$ is the total concentration of the unlabeled ligand, and $[R]_T$ is the total concentration of the receptor. The angle θ is defined as

$$\theta = \arccos\left(\frac{-2a^3 + 9ab - 27c}{2\sqrt{(a^2 - 3b)^3}}\right) \quad (10)$$

In the competitive binding experiments, the values of $[L^*]_T$, $[R]_T$ and K_d^* are known and $[L]_T$ is titrated. The values of minimal and maximal anisotropy r_{min} and r_{max} were fixed during fitting at the values of the minimal and maximal asymptotes obtained from the direct binding experiments. The value of K_d was calculated as the only unfixed parameter in the model. Experimental constants used for the competitive fluorescence polarization experiments are summarized in **Table S2**. The K_d values of all inhibitors, together with their IC_{50} values obtained by fitting a four-parameter logistic model are given in **Table S3** and **Table S4**.

Table S2. Experimental constants used for competitive fluorescence polarization experiments.

	[galectin] (μM)	[probe] (μM)
Gal-1	0.500	0.100
Gal-3C	0.050	0.005

Table S3. Comparison of IC_{50} and K_d values for derivatives **3a – 3af**.[a]

Compound	Gal-1		Gal-3C	
	IC_{50} (μM)	K_d (μM)	IC_{50} (μM)	K_d (μM)
β-LacOMe	1600 ± 70	770 ± 200	760 ± 90	230 ± 20
TDG	130 ± 5	88 ± 10	180 ± 50	69 ± 30
**3a**	910 ± 20	590 ± 80	290 ± 1	110 ± 30
**3b**	920 ± 90	480 ± 100	400 ± 50	130 ± 20
**3c**	1600 ± 300	1000 ± 70	500 ± 30	180 ± 50
**3d**	1000 ± 90	560 ± 100	490 ± 60	160 ± 30
**3e**	590 ± 70	320 ± 100	270 ± 10	87 ± 10
**3f**	670 ± 30	360 ± 20	300 ± 50	98 ± 3
**3g**	930 ± 8	510 ± 30	280 ± 20	93 ± 20
**3h**	600 ± 20	400 ± 20	280 ± 100	130 ± 80
**3i**	570 ± 10	310 ± 90	330 ± 5	110 ± 8
**3j**	650 ± 30	360 ± 30	360 ± 10	110 ± 10
**3k**	660 ± 8	380 ± 60	350 ± 40	110 ± 20
**3l**	720 ± 40	400 ± 20	360 ± 20	110 ± 20
**3m**	970 ± 50	660 ± 70	350 ± 30	140 ± 50
**3n**	680 ± 10	360 ± 40	280 ± 10	89 ± 20
**3o**	460 ± 80	240 ± 40	240 ± 40	76 ± 20
**3p**	980 ± 10	540 ± 30	350 ± 10	110 ± 20
**3q**	770 ± 70	410 ± 20	240 ± 5	80 ± 9
**3r**	1400 ± 100	860 ± 30	130 ± 20	41 ± 10
**3s**	1800 ± 200	1000 ± 300	110 ± 0.2	39 ± 4
**3t**	1300 ± 30	840 ± 80	120 ± 9	51 ± 10
**3u**	2100 ± 800	1200 ± 10	130 ± 20	54 ± 6
**3v**	660 ± 100	430 ± 30	240 ± 30	95 ± 40
**3w**	670 ± 90	440 ± 100	220 ± 6	88 ± 20
**3x**	840 ± 90	540 ± 60	270 ± 0.9	110 ± 30
**3y**	1300 ± 100	810 ± 200	120 ± 9	49 ± 20
**3z**	860 ± 100	560 ± 80	330 ± 1	120 ± 30
**3aa**	900 ± 100	570 ± 60	190 ± 9	69 ± 20
**3ab**	960 ± 40	650 ± 100	130 ± 3	50 ± 9
**3ac**	970 ± 30	630 ± 100	160 ± 20	60 ± 10
**3ad**	1200 ± 300	740 ± 20	32 ± 2	11 ± 2
**3ae**	1000 ± 100	640 ± 90	43 ± 4	16 ± 2
**3af**	1500 ± 300	850 ± 200	11 ± 0.9	4.1 ± 0.4

[a]All values are reported as mean ± SD from two independent experiments, each performed in triplicate.

Table S4. Comparison of IC_{50} and K_d values for derivatives **GB0139**, **10**, and **11**.[a]

Compound	Gal-1		Gal-3C	
	IC_{50} (μM)	K_d (μM)	IC_{50} (μM)	K_d (μM)
**GB0139**	0.64 ± 0.02	0.16 ± 0.05	0.079 ± 0.008	0.0097 ± 0.003
**10**	7.2 ± 0.6	3.6 ± 0.4	0.15 ± 0.009	0.031 ± 0.004
**11**	4.3 ± 0.2	2.2 ± 0.3	0.058 ± 0.01	0.0057 ± 0.002

[a]All values are reported as mean ± SD from three independent experiments, each performed in triplicate.

X-ray crystallographic data for the Gal-3C:**3r** complex

Table S5. Crystal parameters, data collection, and refinement statistics.

**Data collection statistics**	
Protein	**human Galectin-3C**
Compound	**3r**
PDB code	9S62
Space group	$P2_12_12_1$
Cell parameters (Å, º)	a = 36.82<br>b = 58.07<br>c = 63.38<br>α=β=γ= 90
Number of molecules in AU	1
Wavelength (Å)	0.9184
Resolution (Å)	31.12 – 1.059<br>(1.1 – 1.059)
Number of unique reflections	57,197<br>(7,912)
Multiplicity	3.55 (3.50)
Completeness (%)	91.5 (79.7)
R_{meas}[a]	0.066 (1.171)
$CC_{1/2}$	0.997 (0.622)
Average $I/\sigma(I)$	9.84 (1.23)
Wilson B ($Å^2$)[b]	10.9
**Refinement statistics**	
Resolution range (Å)	31.12 – 1.059<br>(1.087 – 1.059)
No. of reflections in working set	55,481<br>(3,918)
No. of reflections in test set	3,155<br>(97)
R value (%)[c]	0.151<br>(0.355)
R_{free} value (%)[d]	0.167<br>(0.365)
RMSD bond length (Å)	0.012
RMSD angle (º)	1.788
Number of atoms in AU	1,501
Number of protein atoms in AU	1,194
Number of water molecules in AU	275
Mean B value ($Å^2$)	15.26
Residues in favored regions (%)	96.6
Residues in allowed regions (%)	2.5

Values in parentheses refer to the highest-resolution shell. [a]R_{meas} defined in ref[4]. [b]Wilson B by CCP4.[5]
[c]R-value = $||F_o| - |F_c||/|F_o|$, where F_o and F_c are the observed and calculated structure factors, respectively.
[d]As determined by Refmac5.[6]

Metabolic stability, toxicity, and solubility

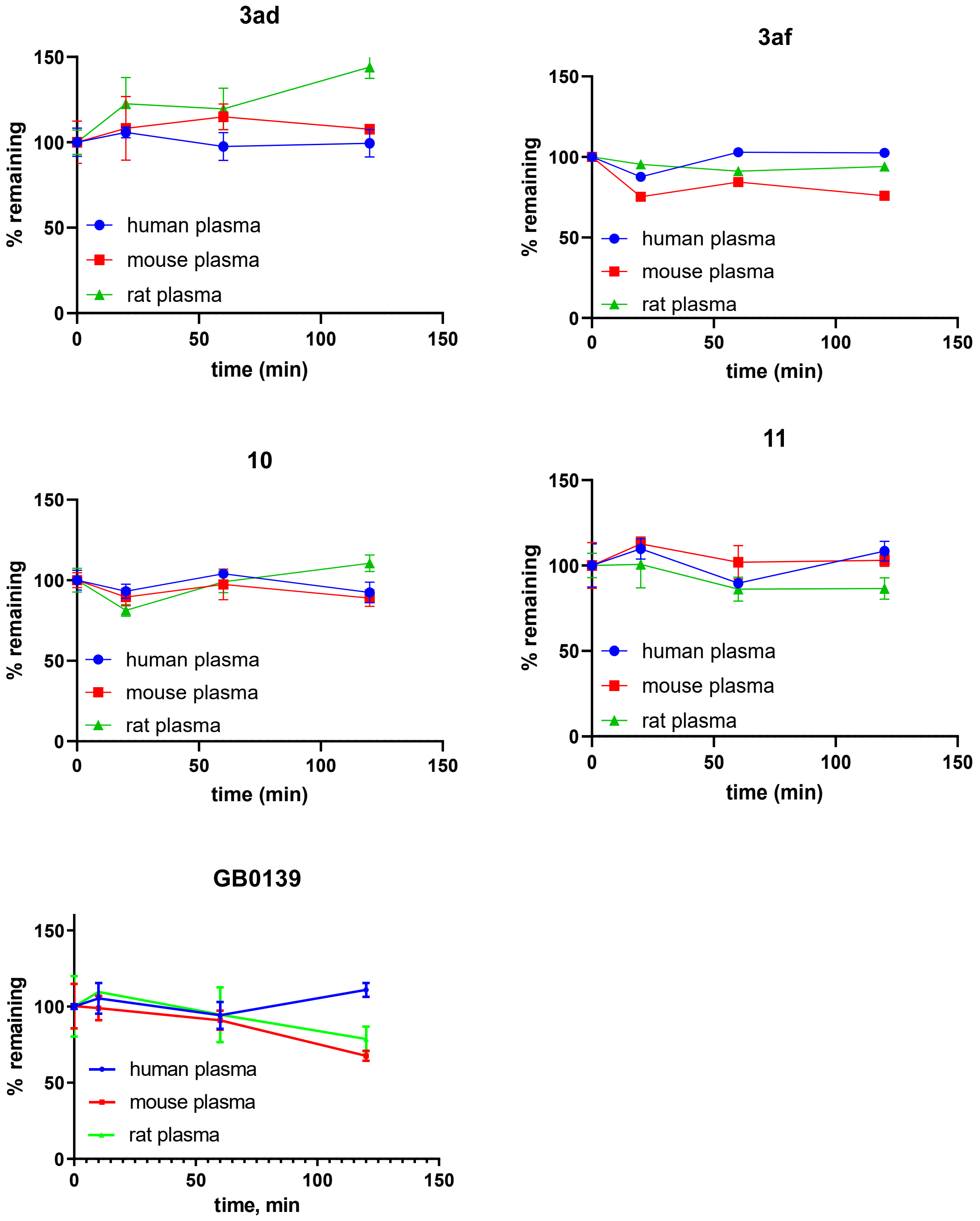

Figure S3. Metabolic stability of compounds **3ad**, **3af**, **10**, **11**, and **GB0139** in mouse, rat, and human plasma after incubation at 37 °C. Concentrations were normalized to time zero. Data are presented as mean ± SD (n = 3).

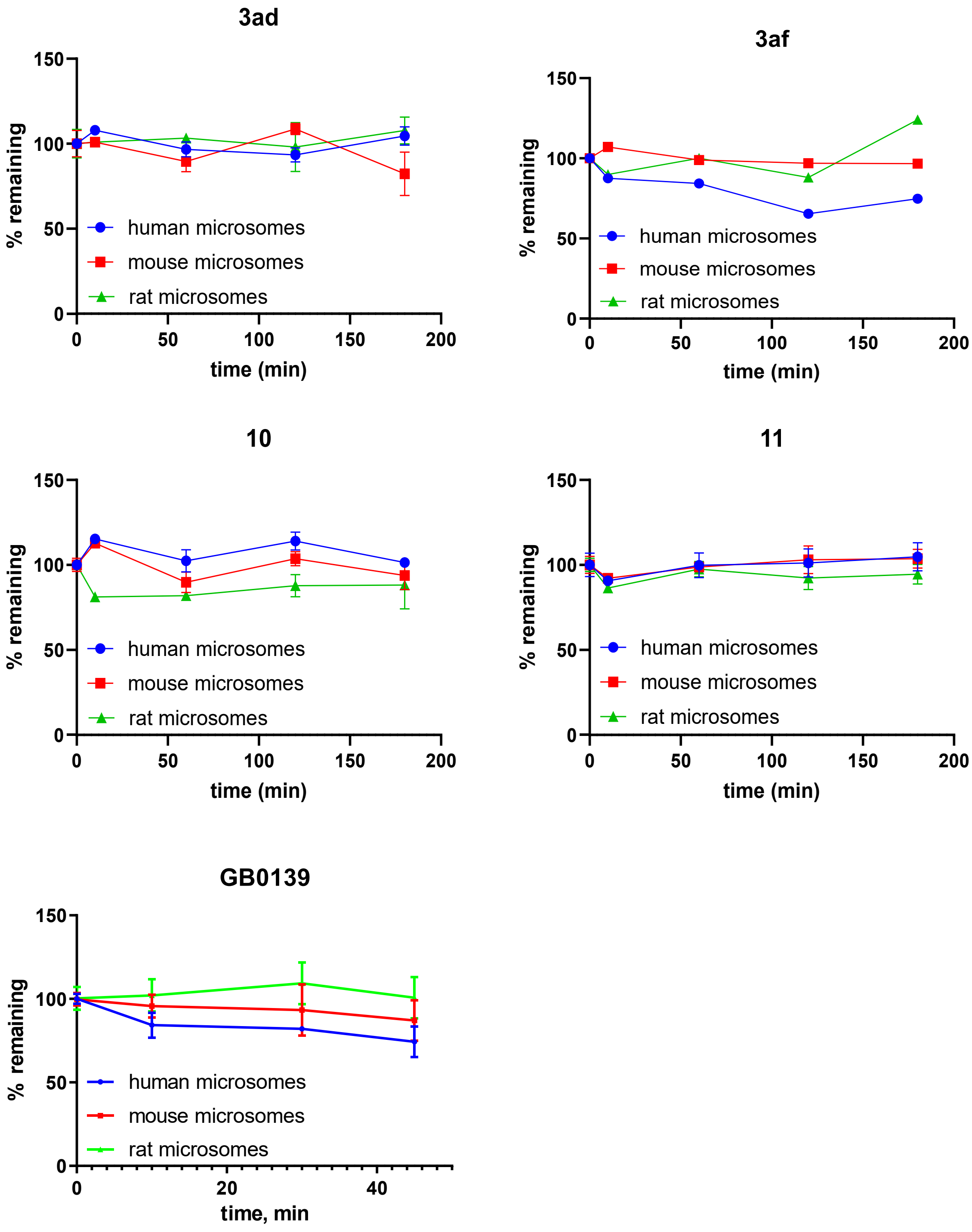

Figure S4. Metabolic stability of compounds **3ad**, **3af**, **10**, **11**, and **GB0139** in liver microsomes from mouse, rat, and human after incubation at 37 °C. Concentrations were normalized to time zero. Data are presented as mean ± SD (n = 3).

Table S6: Multiple reaction monitoring (MRM) used for quantifications of compounds **3ad**, **3af**, **10**, and **11** by Echo MS analysis.

Compound	MRM Transition	Note
**3ad**	520.1 → 196.1	Quantifying
	520.1 → 358.1	Confirming
**3af**	543.1 → 219.1	Quantifying
	543.1 → 381.1	Confirming
**10**	746.1 → 486.1	Quantifying
	746.1 → 308.0	Confirming
**11**	704.2 → 308.1	Quantifying
	704.2 → 219.1	Confirming

Table S7. Kinetic solubility of compounds **3ad**, **3af**, **10**, and **11** in PBS at pH 7.4.

**Compound**	**Solubility (μM)**
**3ad**	> 100
**3af**	> 100
**10**	> 100
**11**	> 100

Molecular modeling

A

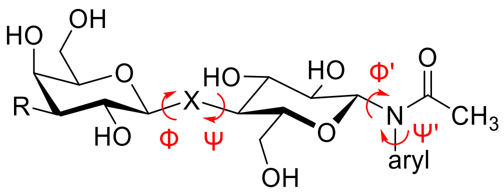

B

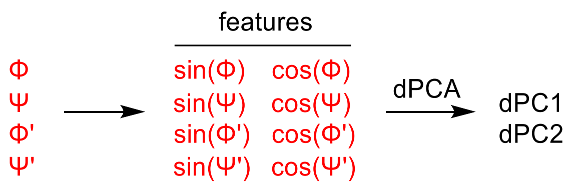

Figure S5. Dihedral principal component analysis (dPCA). (**A**) Definition of dihedral angles used for dPCA for compounds **3a**, **3r**, **3af**, and **11**. (**B**) Overall workflow of dPCA. The dihedral angles are first transformed into their sine and cosine values. These features are then used for dimensionality reduction into two dihedral principal components (dPC1 and dPC2).

Synthetic procedures of precursor compounds

General procedures A, B, C, and D are reported in the manuscript in the Experimental section. To simplify the characterization of prepared compounds, the majority of NMR spectra of compounds **2** and **3** were recorded at elevated temperatures in order to coalesce signals of individual rotamers (see **Figure S6**). In cases of compounds with significantly higher rotational barriers, room temperature NMR data were used, and the signals of the major rotamer are assigned and reported. NMR signals of compounds **2a-ae**, **3a-af** and **6-11** were assigned according to the patterns depicted in **Figure S7**.

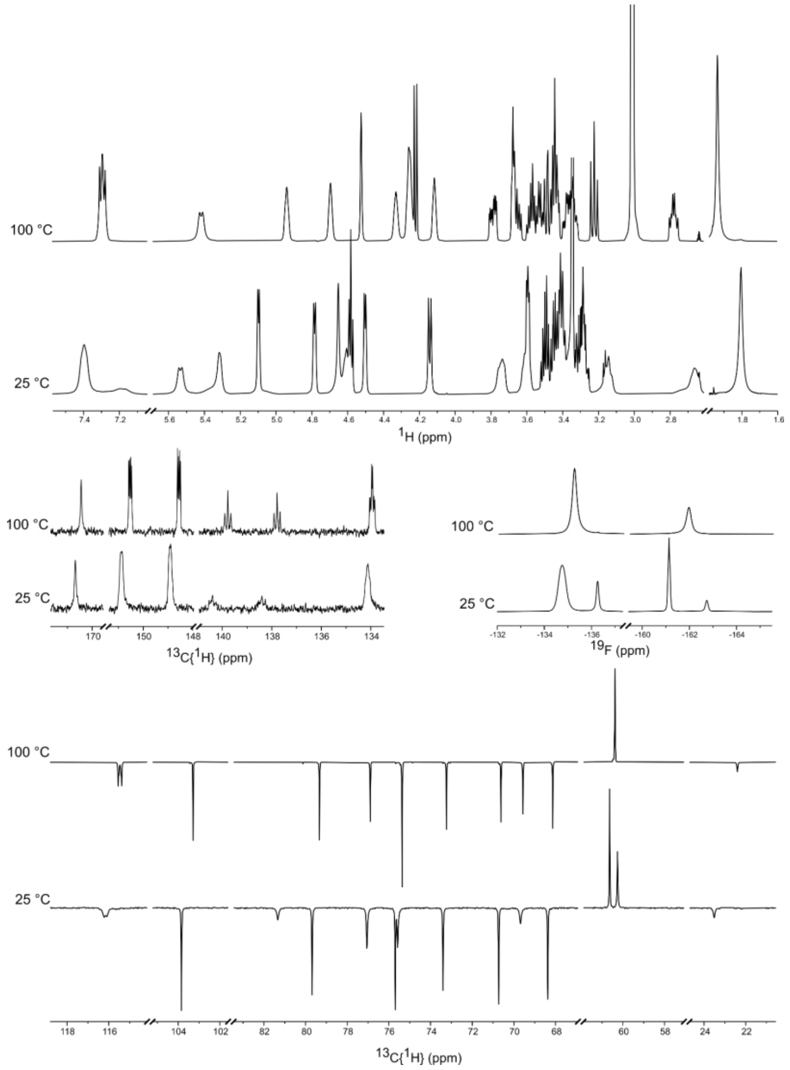

Figure S6. Comparison of ^{1}H, ^{13}C APT, and ^{19}F NMR spectra of compound **3h** at 25 and 100 °C.

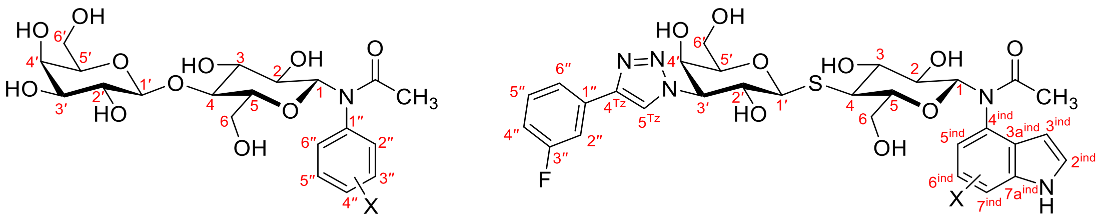

Figure S7. NMR assignment patterns.

*2,3,6-Tri-O-acetyl-4-O-(2,3,4,6-tetra-O-acetyl-β-D-galactopyranosyl)-β-D-glucopyranose (**1**)*

Compound **1** was prepared from lactose following a published procedure.[7]

*N-[2,3,6-Tri-O-acetyl-4-O-(2,3,4,6-tetra-O-acetyl-β-D-galactopyranosyl)-β-D-glucopyranosyl]-N-phenylacetamide (**2a**)*

Following General procedure A, compound **1** (0.636 g, 1.0 mmol) was reacted with aniline (0.153 mL, 1.7 mmol) for 3 days. The crude product was then subjected to General procedure B, affording product **2a** (0.499 g, 66%) as a white foam. $[\alpha]_D^{20}$ = +34.7 (*c* 0.5 in $CHCl_3$); **^{1}H NMR** (500 MHz, DMSO-d_6; T = 100 °C) δ 1.84 (s, 3H, CH_3CON), 1.89 (s, 3H, CH_3CO), 1.90 (s, 3H, CH_3CO), 1.96 (s, 3H, CH_3CO), 1.97 (s, 3H, CH_3CO), 2.00 (s, 3H, CH_3CO), 2.07 (s, 3H, CH_3CO), 2.08 (s, 3H, CH_3CO), 3.67 (dd, $J_{4,5}$ = 9.9, $J_{4,3}$ = 9.2 Hz, 1H, H-4), 3.92 (ddd, $J_{5,4}$ = 9.9, $J_{5,6a}$ = 5.3, $J_{5,6b}$ = 2.2 Hz, 1H, H-5), 4.00 (dd, J_{gem} = 11.2, $J_{6a',5'}$ = 6.8 Hz, 1H, H-6a'), 4.03 (dd, J_{gem} = 11.1, $J_{6b',5'}$ = 6.1 Hz, 1H, H-6b'), 4.09 (dd, J_{gem} = 12.1, $J_{6a,5}$ = 5.3 Hz, 1H, H-6a), 4.15 (ddd, $J_{5',6a'}$ = 7.3, $J_{5',6b'}$ = 6.2, $J_{5',4'}$ = 1.3 Hz, 1H, H-5'), 4.44 (dd, $J_{2,1}$ = 9.8, $J_{2,3}$ = 9.1 Hz, 1H, H-2), 4.48 (dd, J_{gem} = 12.1, $J_{6b,5}$ = 2.3 Hz, 1H, H-6b), 4.73 (d, $J_{1',2'}$ = 7.9 Hz, 1H, H-1'), 4.87 (dd, $J_{2',3'}$ = 10.3, $J_{2',1'}$ = 7.9 Hz, 1H, H-2'), 5.12 (dd, $J_{3',2'}$ = 10.3, $J_{3',4'}$ = 3.7 Hz, 1H, H-3'), 5.20 (t, $J_{3,4}$ = $J_{3,2}$ = 9.2 Hz, 1H, H-3), 5.24 (dd, $J_{4',3'}$ = 3.6, $J_{4',5'}$ = 1.3 Hz, 1H, H-4'), 5.97 (d, $J_{1,2}$ = 9.6 Hz, 1H, H-1), 7.10 – 7.20 (m, 2H, Ar-H), 7.36 – 7.43 (m, 3H, Ar-H); **^{13}C NMR** (126 MHz, DMSO-d_6; T = 100 °C) δ 19.49, 19.54, 19.61, 19.62, 19.68, 19.74, 19.84 (7 × **C**H_3CO-2,3,6,2',3',4',6'), 22.19 (**C**H_3CON), 60.49 (CH_2-6'), 61.36 (CH_2-6), 66.92 (CH-4'), 68.60 (CH-2), 68.91 (CH-2'), 69.59 (CH-5'), 70.12 (CH-3'), 73.44 (CH-3), 73.58 (CH-5), 74.94 (CH-4), 80.04 (CH-1), 99.20 (CH-1'), 127.79 (CH-Ar), 128.38 (CH-Ar), 129.18 (CH-Ar), 137.15 (C-1''), 167.99, 168.26, 168.62, 169.04, 169.05, 169.32 (7 × CH_3**C**O-2,3,6,2',3',4',6'), 170.03 (CH_3**C**ON); **IR** ($CHCl_3$) 2937, 2874, 1754, 1678, 1597, 1494, 1429, 1370, 1230, 1172, 1075, 1055, 1021, 702, 602 cm^{-1}; **HRMS** (ESI) $[M+Na]^+$ *m/z* calcd for $C_{34}H_{43}O_{18}NNa$: 776.2372, found: 776.2375; $[M+H]^+$ *m/z* calcd for $C_{34}H_{44}O_{18}N$: 754.2553, found: 754.2559.

*N-[2,3,6-Tri-O-acetyl-4-O-(2,3,4,6-tetra-O-acetyl-β-D-galactopyranosyl)-β-D-glucopyranosyl]-N-(4-methylphenyl)acetamide (**2b**)*

Following General procedure A, compound **1** (0.636 g, 1.0 mmol) was reacted with 4-methylaniline (0.182 g, 1.7 mmol) for 3 days. The crude product was then subjected to General procedure B, affording product **2b** (0.621 g, 81%) as a white foam. $[\alpha]_D^{20}$ = +28.2 (*c* 0.3 in $CHCl_3$); **^{1}H NMR** (500 MHz, DMSO-d_6; T = 100 °C) δ 1.81 (s, 3H, CH_3CON), 1.89 (s, 3H, CH_3CO), 1.90 (s, 3H, CH_3CO), 1.96 (s, 3H, CH_3CO), 1.97 (s, 3H, CH_3CO), 2.00 (s, 3H, CH_3CO), 2.07 (s, 3H, CH_3CO), 2.08 (s, 3H, CH_3CO), 2.35 (s, 3H, CH_3), 3.65 (t, $J_{4,3}$ = $J_{4,5}$ = 9.5 Hz, 1H, H-4), 3.90 (ddd, $J_{5,4}$ = 9.8, $J_{5,6a}$ = 5.2, $J_{5,6b}$ = 2.2 Hz, 1H, H-5), 4.00 (dd, J_{gem} = 11.2, $J_{6'a,5'}$ = 6.8 Hz, 1H, H-6'a), 4.03 (dd, J_{gem} = 11.2, $J_{6'b,5'}$ = 6.0 Hz, 1H, H-6'b), 4.07 (dd, J_{gem} = 11.8, $J_{6a,5}$ = 4.9 Hz, 1H, H-6a), 4.15 (td, $J_{5',6'}$ = 6.5, $J_{5',4'}$ = 1.3 Hz, 1H, H-5'), 4.43 (t, $J_{2,1}$ = $J_{2,3}$ = 9.3 Hz, 1H, H-2), 4.47 (dd, J_{gem} = 12.0, $J_{6b,5}$ = 2.2 Hz, 1H, H-6b), 4.73 (d, $J_{1',2'}$ = 8.0 Hz, 1H, H-1'), 4.87 (dd, $J_{2',3'}$ = 10.3, $J_{2',1'}$ = 7.9 Hz, 1H, H-2'), 5.12 (dd, $J_{3',2'}$ = 10.3,

$J_{3',4'}$ = 3.6 Hz, 1H, H-3'), 5.19 (t, $J_{3,4}$ = $J_{3,2}$ = 9.1 Hz, 1H, H-3), 5.24 (dd, $J_{4',3'}$ = 3.6, $J_{4',5'}$ = 1.3 Hz, 1H, H-4'), 5.96 (d, $J_{1,2}$ = 9.6 Hz, 1H, H-1), 7.02 (d, $J_{2'',3''}$ = 8.0 Hz, 2H, H-2''), 7.20 (d, $J_{3'',2''}$ = 8.0 Hz, 2H, H-3''); **^{13}C NMR** (126 MHz, DMSO-d_6; T = 100 °C) δ 19.49, 19.54, 19.60, 19.61, 19.68, 19.76, 19.84, 19.95 (7 × **C**H$_3$CO-2,3,6,2',3',4',6'; 1 × CH$_3$), 22.18 (**C**H$_3$CON), 60.49 (CH$_2$-6'), 61.34 (CH$_2$-6), 66.92 (CH-4'), 68.91 (CH-2), 69.59 (CH-2'), 70.12 (CH-5'), 73.45 (CH-3), 73.53 (CH-5), 74.92 (CH-4), 79.88 (CH-1), 99.19 (CH-1'), 128.90 (CH-3''), 128.96 (CH-2''), 134.47 (C-1''), 137.34 (C-4''), 168.01, 168.26, 168.61, 169.04, 169.05, 169.31, (7 × CH$_3$**C**O-2,3,6,2',3',4',6'), 170.14 (CH$_3$**C**ON); **IR** (CHCl$_3$) 2960, 2874, 1753, 1672, 1604, 1583, 1580, 1429, 1371, 1331, 1290, 1230, 1172, 1055, 1023, 980, 598 cm^{-1}; **HRMS** (ESI) [M+Na]$^+$ *m/z* calcd for $C_{35}H_{45}O_{18}NNa$: 790.2529, found: 790.2528.

*N-[2,3,6-Tri-O-acetyl-4-O-(2,3,4,6-tetra-O-acetyl-β-D-galactopyranosyl)-β-D-glucopyranosyl]-N-(3-ethynylphenyl)acetamide (**2c**)*

Following General procedure A, compound **1** (0.636 g, 1.0 mmol) was reacted with 4-ethynylaniline (0.19 mL, 1.7 mmol) for 3 days. The crude product was then subjected to General procedure B, affording product **2c** (0.456 g, 59%) as a white foam. $[\alpha]_D^{20}$ = +27.6 (*c* 0.4 in CHCl$_3$); **^{1}H NMR** (500 MHz, DMSO-d_6; 100°C) δ 1.89 (s, 3H, CH$_3$CON), 1.90 (s, 3H, CH$_3$CO), 1.90 (s, 3H, CH$_3$CO), 1.97 (s, 3H, CH$_3$CO), 1.97 (s, 3H, CH$_3$CO), 2.00 (s, 3H, CH$_3$CO), 2.08 (s, 3H, CH$_3$CO), 2.09 (s, 3H, CH$_3$CO), 3.70 (t, $J_{4,5}$ = $J_{4,3}$ = 9.5 Hz, 1H, H-4), 3.94 (ddd, $J_{5,4}$ = 9.9, $J_{5,6a}$ = 5.0, $J_{5,6b}$ = 2.2 Hz, 1H, H-5), 4.00 (dd, J_{gem} = 11.2, $J_{6'a,5'}$ = 6.7 Hz, 1H, H-6'a), 4.01 (s, 1H, C≡CH), 4.03 (dd, J_{gem} = 11.2, $J_{6'b,5'}$ = 6.1 Hz, 1H, H-6'b), 4.09 (dd, J_{gem} = 12.1, $J_{6a,5}$ = 4.9 Hz, 1H, H-6a), 4.15 (ddd, $J_{5',6'a}$ = 6.7, $J_{5',6'b}$ = 6.1, $J_{5',4'}$ = 1.3 Hz, 1H, H-5'), 4.42 (t, $J_{2,1}$ = $J_{2,3}$ = 9.3 Hz, 1H, H-2), 4.48 (dd, J_{gem} = 12.1, $J_{6b,5}$ = 2.3 Hz, 1H, H-6b), 4.73 (d, $J_{1',2'}$ = 7.9 Hz, 1H, H-1'), 4.87 (dd, $J_{2',3'}$ = 10.2, $J_{2',1'}$ = 7.9 Hz, 1H, H-2'), 5.12 (dd, $J_{3',2'}$ = 10.2, $J_{3',4'}$ = 3.6 Hz, 1H, H-3'), 5.21 (t, $J_{3,4}$ = $J_{3,2}$ = 9.2 Hz, 1H, H-3), 5.25 (dd, $J_{4',3'}$ = 3.7, $J_{4',5'}$ = 1.2 Hz, 1H, H-4'), 5.95 (d, $J_{1,2}$ = 9.5 Hz, 1H, H-1), 7.18 (ddd, $J_{6'',5''}$ = 7.9, $J_{6'',2''}$ = 2.1, $J_{6'',4''}$ = 1.2 Hz, 1H, H-6''), 7.27 (ddd, $J_{2'',6''}$ = 2.2, $J_{2'',4''}$ = 1.5, $J_{2'',5''}$ = 0.7 Hz, 1H, H-2''), 7.41 (td, $J_{5'',6''}$ = $J_{5'',4''}$ = 7.9, $J_{5'',2''}$ = 0.5 Hz, 1H, H-5''), 7.48 (dt, $J_{4'',5''}$ = 7.7, $J_{4'',2''}$ = $J_{4'',6''}$ = 1.4 Hz, H-4''); **^{13}C NMR** (126 MHz, DMSO-d_6; 100°C) δ 19.49, 19.54, 19.61, 19.62, 19.66, 19.67, 19.84 (7 × **C**H$_3$CO-2,3,6,2',3',4',6'), 22.11 (**C**H$_3$CON), 60.49 (CH$_2$-6'), 61.21 (CH$_2$-6), 66.93 (CH-4'), 68.48 (CH-2), 68.91 (CH-2'), 69.60 (CH-5'), 70.13 (CH-3'), 73.32 (CH-3), 73.56 (CH-5), 74.82 (CH-4), 80.21 (CH-1), 80.34 (C≡**C**H), 82.11 (**C**≡CH), 99.20 (CH-1'), 122.25 (C-3''), 128.77 (CH-5''), 130.02 (CH-6''), 131.11 (CH-4''), 132.23 (CH-2''), 137.35 (C-1''), 168.00, 168.26, 168.60, 168.61, 169.04, 169.05, 169.30 (7 × CH$_3$**C**O-2,3,6,2',3',4',6'), 169.95 (CH$_3$**C**ON); **IR** (CHCl$_3$) 3306, 2114, 1754, 1682, 1597, 1579, 1482, 1371, 1231, 1172, 1055, 699, 601 cm^{-1}; **HRMS** (ESI) [M+Na]$^+$ *m/z* calcd for $C_{36}H_{43}O_{18}NNa$: 800.2372, found: 800.2375.

*N-[2,3,6-Tri-O-acetyl-4-O-(2,3,4,6-tetra-O-acetyl-β-D-galactopyranosyl)-β-D-glucopyranosyl]-N-(3-trifluoromethylphenyl)acetamide (**2d**)*

Following General procedure A, compound **1** (0.320 g, 0.5 mmol) was reacted with 3-trifluoromethylaniline (0.105 mL, 0.85 mmol) for 3 days. The crude product was then subjected to General procedure B, affording product **2d** (0.230 g, 56%) as a white foam. $[\alpha]_D^{20}$ = +36.0 (*c* 0.2 in $CHCl_3$); **^{1}H NMR** (500 MHz, DMSO-d_6, T = 100 °C) δ 1.89, 1.90 (2 × s, 6H CH_3CO-3,3'), 1.94 (s, 3H, CH_3COC), 1.96 (s, 3H, CH_3CO), 1.97 (s, 3H, CH_3CO), 2.00 (s, 3H, CH_3CO), 2.06 (s, 3H, CH_3CO), 2.08 (s, 3H, CH_3CO), 3.70 (t, $J_{4,3}$ = $J_{4,5}$ = 9.5 Hz, 1H, H-4), 3.95 – 4.04 (m, 2H, H-5,6b'), 4.03 (dd, J_{gem} = 11.2, $J_{6a',5'}$ = 6.2 Hz, 1H, H-6a'), 4.10 (dd, J_{gem} = 12.1, $J_{6a,5}$ = 5.2 Hz, 1H, H-6a), 4.15 (td, $J_{5',6a'}$ = $J_{5',6b'}$ = 6.2, $J_{5',4'}$ = 1.4 Hz, 1H, H-4'), 4.39 (t, $J_{2,3}$ = $J_{2,1}$ = 9.5 Hz, 1H, H-2), 4.45 (dd, J_{gem} = 12.1, $J_{6b,5}$ = 2.2 Hz, 1H, H-6b), 4.73 (d, $J_{1',2'}$ = 7.9 Hz, 1H, H-1'), 4.87 (dd, $J_{2',3'}$ = 10.3, $J_{2',1'}$ = 7.9 Hz, 1H, H-2'), 5.12 (dd, $J_{3',2'}$ = 10.3, $J_{3',4'}$ = 3.7 Hz, 1H, H-3'), 5.23 (t, $J_{3,2}$ = $J_{3,4}$ = 9.5 Hz, 1H, H-3), 5.24 (dd, $J_{4',3'}$ = 3.7, $J_{4',5'}$ = 1.2 Hz, 1H, H-4'), 5.98 (d, $J_{1,2}$ = 9.5 Hz, 1H, H-1), 7.46 (d, $J_{6'',5''}$ = 7.9 Hz, 1H, H-6''), 7.48 (s, 1H, H-2''), 7.65 (t, $J_{5'',6''}$ = $J_{5'',4''}$ = 7.9 Hz, 1H, H-5''), 7.73 (d, $J_{4'',5''}$ = 7.9 Hz, 1H, H-4''); **^{13}C NMR** (126 MHz, DMSO-d_6, T = 100 °C) δ 19.53, 19.58, 19.61, 19.64, 19.67, 19.71 (**C**H_3CO-2,3,6,2',3',4',6'), 22.11 (**C**H_3CON), 60.51 (CH_2-6'), 61.35 (CH_2-6), 66.92 (CH-4'), 68.34 (CH-2), 68.90 (CH-2'), 69.60 (CH-5'), 70.12 (CH-3'), 73.22 (CH-3), 73.60 (CH-5), 74.82 (CH-4), 80.50 (CH-1), 99.21 (CH-1'), 119.71 – 126.56 (m, **C**F_3), 124.53 (q, $J_{C,F}$ = 3.1 Hz, CH-4''), 125.95 (q, $J_{C.F}$ = 3.9 Hz, CH-2''), 129.60 (q, $J_{C,F}$ = 31.9 Hz, C-3''), 129.62 (CH-5''), 133.29 (CH-6''), 137.77 (C-1''), 168.06 (CH_3**C**O-2), 168.29 (CH_3**C**O-2'), 168.62, 168.66 (2 x CH_3**C**O-3,3'), 169.06, 169.09 (2 x CH_3**C**O-4',6'), 169.33 (CH_3**C**O-6), 170.03 (CH_3**C**ON); **^{19}F NMR** (471 MHz, DMSO-d_6, T = 100 °C) δ -61.28 (bs, 3F, CF_3); **IR** ($CHCl_3$) 1754, 1687, 1613, 1593, 1492, 1449, 1371, 1327, 1231, 1175, 1137, 1055, 980, 704, 601, 548 cm^{-1}; **HRMS** (ESI) $[M+Na]^+$ *m/z* calcd for $C_{35}H_{42}O_{18}NF_3Na$: 844.2246, found: 844.2245.

*N-[2,3,6-Tri-O-acetyl-4-O-(2,3,4,6-tetra-O-acetyl-β-D-galactopyranosyl)-β-D-glucopyranosyl]-N-(4-fluorophenyl)acetamide (**2e**)*

Following General procedure A, compound **1** (0.350 g, 0.55 mmol) was reacted with 4-fluroaniline (0.089 mL, 0.94 mmol) for 3 days. The crude product was then subjected to General procedure B, affording product **2e** (0.363 g, 86%) as a white foam. $[\alpha]_D^{20}$ = +28.8 (*c* 0.3 in $CHCl_3$); **^{1}H NMR** (500 MHz, DMSO-d_6, T = 100 °C) δ 1.87 (s, 3H, CH_3CON), 1.89 (s, 3H, CH_3CO), 1.90 (s, 3H, CH_3CO), 1.97 (s, 3H, CH_3CO), 1.97 (s, 3H, CH_3CO), 2.00 (s, 3H, CH_3CO), 2.07 (s, 3H, CH_3CO), 2.08 (s, 3H, CH_3CO), 3.68 (t, $J_{4,3}$ = $J_{4,5}$ = 9.5 Hz, 1H, H-4), 3.94 (ddd, $J_{5,4}$ = 9.4, $J_{5,6a}$ = 5.1, $J_{5,6b}$ = 1.8 Hz, 1H, H-5), 4.00 (dd, J_{gem} = 11.2, $J_{6'a,5'}$ = 6.8 Hz, 1H, H-6'a), 4.03 (dd, J_{gem} = 11.2, $J_{6'b,5'}$ = 6.1 Hz, 1H, H-6'b), 4.08 (dd, J_{gem} = 12.1, $J_{6a,5}$ = 5.3 Hz, 1H, H-6a), 4.16 (td, $J_{5',6'a}$ = $J_{5',6'b}$ = 6.5, $J_{5',4'}$ = 1.2 Hz, 1H, H-5'), 4.42 (t, $J_{2,1}$ = $J_{2,3}$ = 9.4 Hz, 1H, H-2), 4.46 (dd, J_{gem} = 12.0, $J_{6b,5}$ = 2.1 Hz, 1H, H-6b), 4.73 (d, $J_{1',2'}$ = 7.9 Hz, 1H, H-1'), 4.87 (dd, $J_{2',3'}$ = 10.3, $J_{2',1'}$ = 7.9 Hz, 1H, H-2'), 5.12 (dd, $J_{3',2'}$ = 10.3, $J_{3',4'}$ = 3.6 Hz, 1H, H-3'), 5.21 (t, $J_{3,4}$ = $J_{3,2}$ = 9.2 Hz, 1H, H-3), 5.24 (dd, $J_{4',3'}$ = 3.6, $J_{4',5'}$ = 1.0 Hz, 1H, H-4'), 5.96 (d, $J_{1,2}$ = 9.5 Hz, 1H, H-1), 7.15 – 7.23 (m, 4H, H-2'',3''); **^{13}C NMR** (126 MHz, DMSO-d_6, T = 100 °C) δ 19.57, 19.62, 19.67, 19.69, 19.73, 19.80, 19.91 (7 × **C**H_3CO-2,3,6,2',3',4',6'), 22.20 (**C**H_3CON),

60.51 (CH_2-6'), 61.39 (CH_2-6), 66.93 (CH-4'), 68.49 (CH-2), 68.91 (CH-2'), 69.59 (CH-5'), 70.13 (CH-3'), 73.37 (CH-3), 73.57 (CH-5), 74.99 (CH-4), 80.06 (CH-1), 99.26 (CH-1'), 115.17 (d, $J_{C,F}$ = 22.6 Hz, CH-3''), 131.31 (d, $J_{C,F}$ = 8.8 Hz, CH-2''), 133.28 (d, $J_{C,F}$ = 2.9 Hz, C-1''), 161.24 (d, $J_{C,F}$ = 245.8 Hz, C-4''), 168.16, 168.32, 168.66, 168.68, 169.10, 169.11, 169.42 (7 × CH_3**C**O-2,3,6,2',3',4',6'), 170.20 (CH_3**C**ON); **^{19}F NMR** (471 MHz DMSO-*d*$_6$, T = 100 °C) δ -109.58 (m, 1F, F-4''); **IR** ($CHCl_3$) 2985, 2940, 2874, 1753, 1681, 1601, 1510, 1429, 1372, 1234, 1154, 1055, 1018, 980, 825 cm^{-1}; **HRMS** (ESI) $[M+Na]^+$ *m/z* calcd for $C_{34}H_{42}O_{18}NFNa$: 794.2278, found: 794.2276.

*N-[2,3,6-Tri-O-acetyl-4-O-(2,3,4,6-tetra-O-acetyl-β-D-galactopyranosyl)-β-D-glucopyranosyl]-N-(3-fluorophenyl)acetamide (**2f**)*

Following General procedure A, compound **1** (0.350 g, 0.55 mmol) was reacted with 3-fluroaniline (0.090 mL, 0.94 mmol) for 3 days. The crude product was then subjected to General procedure B, affording product **2f** (0.244 g, 57%) as a yellow foam. $[\alpha]_D^{20}$ = +43.2 (*c* 0.2 in $CHCl_3$); **^{1}H NMR** (500 MHz, DMSO-*d*$_6$, T = 100 °C) δ 1.90 (s, 3H, CH_3CO), 1.90 (s, 3H, CH_3CO), 1.91 (s, 3H, CH_3CON), 1.97 (s, 3H, CH_3CO), 1.97 (s, 3H, CH_3CO), 2.00 (s, 3H, CH_3CO), 2.07 (s, 3H, CH_3CO), 2.08 (s, 3H, CH_3CO), 3.71 (t, $J_{4,3} = J_{4,5}$ = 9.5 Hz, 1H, H-4), 3.95 (ddd, $J_{5,4}$ = 9.9, $J_{5,6a}$ = 5.1, $J_{5,6b}$ = 2.2 Hz, 1H, H-5), 4.00 (dd, J_{gem} = 11.2, $J_{6'a,5'}$ = 6.8 Hz, 1H, H-6'a), 4.03 (dd, J_{gem} = 11.2, $J_{6'b,5'}$ = 6.1 Hz, 1H, H-6'b), 4.10 (dd, J_{gem} = 12.1, $J_{6a,5}$ = 5.1 Hz, 1H, H-6a), 4.16 (td, $J_{5',6'a} = J_{5',6'b}$ = 6.5, $J_{5',4'}$ = 1.4 Hz, 1H, H-5'), 4.44 (t, $J_{2,1} = J_{2,3}$ = 9.5 Hz, 1H, H-2), 4.47 (dd, J_{gem} = 12.1, $J_{6b,5}$ = 2.3 Hz, 1H, H-6b), 4.74 (d, $J_{1',2'}$ = 7.9 Hz, 1H, H-1'), 4.87 (dd, $J_{2',3'}$ = 10.2, $J_{2',1'}$ = 7.9 Hz, 1H, H-2'), 5.13 (dd, $J_{3',2'}$ = 10.3, $J_{3',4'}$ = 3.6 Hz, 1H, H-3'), 5.21 (t, $J_{3,4} = J_{3,2}$ = 9.2 Hz, 1H, H-3), 5.24 (dd, $J_{4',3'}$ = 3.7, $J_{4',5'}$ = 1.2 Hz, 1H, H-4'), 5.97 (d, $J_{1,2}$ = 9.6 Hz, 1H, H-1), 7.00 (dt, $J_{2'',F}$ = 10.3, $J_{2'',4''} = J_{2'',6''}$ = 2.1 Hz, 1H, H-2''), 7.01 (ddd, $J_{6'',5''}$ = 8.5, $J_{6'',2''}$ = 1.9, $J_{6'',F}$ = 0.9 Hz, 1H, H-6''), 7.22 (tdd, $J_{4'',F} = J_{4'',5''}$ = 8.6, $J_{4'',2''}$ = 2.6, $J_{4'',6''}$ = 0.9 Hz, 1H, H-4''), 7.44 (td, $J_{5'',4''} = J_{5'',6''}$ = 8.2, $J_{5'',F}$ = 6.5 Hz, 1H, H-5''); **^{13}C NMR** (126 MHz, DMSO-*d*$_6$, T = 100 °C) δ 19.56, 19.61, 19.72, 19.75, 19.83 (7 × **C**H_3CO-2,3,6,2',3',4',6'), 22.18 (CH_3CON), 60.50 (CH_2-6'), 61.28 (CH_2-6), 66.92 (CH-4'), 68.41 (CH-2), 68.90 (CH-2'), 69.58 (CH-5'), 70.12 (CH-3'), 73.33 (CH-3), 73.57 (CH-5), 74.90 (CH-4), 80.13 (CH-1), 99.25 (CH-1'), 114.83 (d, $J_{C,F}$ = 20.9 Hz, CH-4''), 116.40 (d, $J_{C,F}$ = 22.4 Hz, CH-2''), 125.49 (d, $J_{C,F}$ = 3.0 Hz, CH-6''), 129.82 (d, $J_{C,F}$ = 9.2 Hz, CH-5''), 138.63 (d, $J_{C,F}$ = 9.9 Hz, C-1''), 161.46 (d, $J_{C,F}$ = 245.5 Hz, CF-3''), 168.12, 168.32, 168.64, 168.67, 169.09, 169.10, 169.37 (7 × CH_3**C**O-2,3,6,2',3',4',6'), 169.94 (CH_3CON); **^{19}F NMR** (470 MHz, DMSO-*d*$_6$, T = 100 °C) δ -108.07 (m, 1F, F-3''); **IR** ($CHCl_3$) 1754, 1684, 1608, 1594, 1488, 1450, 1371, 1234, 1172, 1055, 1022, 886, 699, 601 cm^{-1}; **HRMS** (ESI) $[M+Na]^+$ *m/z* calcd for $C_{34}H_{42}O_{18}NFNa$: 794.2278, found: 794.2280.

*N-[2,3,6-Tri-O-acetyl-4-O-(2,3,4,6-tetra-O-acetyl-β-D-galactopyranosyl)-β-D-glucopyranosyl]-N-(2-fluorophenyl)acetamide (**2g**)*

Following General procedure A, compound **1** (0.320 g, 0.50 mmol) was reacted with 2-fluroaniline (0.082 mL, 0.85 mmol) for 7 days. The crude product was then subjected to General procedure B, affording product **2g** (0.244 g, 57%) as a white foam. $[\alpha]_D^{20}$ = +42.9 (*c* 0.2 in $CHCl_3$);

^{1}H NMR (500 MHz, DMSO-d_6, T = 100 °C) δ 1.89 (s, 3H, CH_3CO), 1.90 (s, 3H, CH_3CO), 1.92 (s, 6H, CH_3CO, CH_3CON), 1.97 (s, 3H, CH_3CO), 2.00 (s, 3H, CH_3CO), 2.08 (s, 6H, 2 × CH_3CO), 3.70 (t, $J_{4,3}$ = $J_{4,5}$ = 9.3 Hz, 1H, H-4), 3.94 – 3.99 (m, 1H, H-5), 4.00 (dd, J_{gem} = 11.2, $J_{6'a,5'}$ = 6.7 Hz, 1H, H-6'a), 4.03 (dd, J_{gem} = 11.2, $J_{6'b,5'}$ = 6.1 Hz, 1H, H-6'b), 4.08 – 4.14 (m, 1H, H-6a), 4.16 (td, $J_{5',6'a}$ = $J_{5',6'b}$ = 6.5, $J_{5',4'}$ = 1.4 Hz, 1H, H-5'), 4.36 – 4.57 (m, 2H, H-2,6b), 4.74 (d, $J_{1',2'}$ = 7.9 Hz, 1H, H-1'), 4.87 (dd, $J_{2',3'}$ = 10.3, $J_{2',1'}$ = 7.9 Hz, 1H, H-2'), 5.13 (dd, $J_{3',2'}$ = 10.3, $J_{3',4'}$ = 3.6 Hz, 1H, H-3'), 5.22 (t, $J_{3,4}$ = $J_{3,2}$ = 9.2 Hz, 1H, H-3), 5.25 (dd, $J_{4',3'}$ = 3.6, $J_{4',5'}$ = 1.0 Hz, 1H, H-4'), 5.97 (bs, 1H, H-1), 7.20 – 7.23 (m, 1H, H-3''), 7.25 (t, $J_{6'',5''}$ = $J_{6'',F}$ =7.9 Hz, 1H, H-6''), 7.29 – 7.40 (m, 1H, H-3''), 7.45 (q, $J_{4'',F}$ = $J_{4'',5''}$ = $J_{4'',3''}$ = 6.9 Hz, 1H, H-4''); **^{13}C NMR** (126 MHz, DMSO-d_6, T = 100 °C) δ 19.53, 19.58, 19.64, 19.67, 19.70, 19.85 (7 × **C**H_3CO-2,3,6,2',3',4',6'), 21.21 (**C**H_3CON), 60.49 (CH_2-6'), 61.41 (CH_2-6), 66.92 (CH-4'), 68.89 (CH-2), 69.58, 70.11 (CH-3'), 73.37 (CH-3), 73.53 (CH-5), 74.97 (CH-4), 80.96 (CH-1), 99.24 (CH-1'), 115.79 (d, $J_{C,F}$ = 22.1 Hz, H-3''), 124.31 (d, $J_{C,F}$ = 8.6 Hz, H-6''), 130.31 (d, $J_{C,F}$ = 12.3 Hz, H-4''), 130.31 (d, $J_{C,F}$ = 3.7 Hz, H-5''), 168.28, 168.61, 168.65, 169.06, 169.08, 169.36, (7 × CH_3**C**O-2,3,6,2',3',4',6'), 169.83 (CH_3**C**ON); quaternary carbons were not detected due to extensive signal broadening by amide rotation and fluorine coupling; **^{19}F NMR** (471 MHz, DMSO-d_6, T = 100 °C) δ -119.61 (bs); **IR** ($CHCl_3$) 1754, 1689, 1611, 1587, 1502, 1459, 1372, 1297, 1240, 1139, 1077, 1055, 1055, 1021, 863, 828, 593, 537, 447, 405 cm^{-1}; **HRMS** (ESI) $[M+Na]^+$ *m/z* calcd for $C_{34}H_{42}O_{18}NFNa$: 794.22781, found: 794.22635.

*N-[2,3,6-Tri-O-acetyl-4-O-(2,3,4,6-tetra-O-acetyl-β-D-galactopyranosyl)-β-D-glucopyranosyl]-N-(3,4-difluorophenyl)acetamide (**2h**)*

Following General procedure A, compound **1** (0.320 g, 0.5 mmol) was reacted with 3,4-difluoroaniline (0.084 mL, 0.85 mmol) for 3 days. The crude product was then subjected to General procedure B, affording product **2h** (0.245 g, 62%) as a yellow foam. $[\alpha]_D^{20}$ = +28.9 (*c* 0.3 in $CHCl_3$); **^{1}H NMR** (500 MHz, DMSO-d_6, T = 100 °C): 1.90 (s, 3H, CH_3CO-3'), 1.90 (s, 3H, CH_3CO-3), 1.93 (s, 3H, CH_3CON), 1.97 (s, 3H, CH_3CO-6'), 1.98 (s, 3H, CH_3CO-2), 2.00 (s, 3H, CH_3CO-2'), 2.07 (s, 3H, CH_3CO-6), 2.08 (s, 3H, CH_3CO-4'), 3.72 (dd, $J_{4,5}$ = 9.8, $J_{4,3}$ = 9.5 Hz, 1H, H-4), 3.96 (ddd, $J_{5,4}$ = 9.8, $J_{5,6a}$ =5.2, $J_{5,6b}$ = 2.2 Hz, 1H, H-5), 4.00 (dd, J_{gem} = 11.2, $J_{6'a,5'}$ = 6.8 Hz, 1H, H-6'a), 4.03 (dd, J_{gem} = 11.2, $J_{6'b,5'}$ =6.1 Hz, 1H, H-6'b), 4.10 (dd, J_{gem} = 12.1, $J_{6a,5}$ = 5.2 Hz, 1H, H-6a), 4.16 (ddd, $J_{5',6'a}$ = 6.8, $J_{5',6'b}$ = 6.1, $J_{5',4'}$ = 1.3 Hz, 1H, H-5'), 4.44 (t, $J_{2,3}$ = $J_{2,1}$ = 9.5 Hz, 1H, H-2), 4.46 (dd, J_{gem} = 12.1, $J_{6'b,5'}$ = 2.2 Hz, 1H, H-6'b), 4.73 (d, $J_{1',2'}$ = 7.9 Hz, 1H, H-1'), 4.87 (dd, $J_{2',3'}$ = 10.3, $J_{2',1'}$ = 7.9 Hz, 1H, H-2'), 5.13 (dd, $J_{3',2'}$ = 10.3, $J_{3',4'}$ =3.6 Hz, 1H, H-3'), 5.22 (t, $J_{3,2}$ = $J_{3,4}$ = 9.5 Hz, 1H, H-3), 5.25 (dd, $J_{4',3'}$ = 3.6, $J_{4',5'}$ = 1.3 Hz, 1H, H-4'), 5.95 (d, $J_{1,2}$ = 9.5 Hz, 1H, H-1), 7.02 (dddd, $J_{6'',5''}$ = 8.7, $J_{6'',F4''}$ = 4.1, $J_{6'',2''}$ = 2.5, $J_{6'',F3''}$ = 1.7 Hz, 1H, H-6''), 7.21 (ddd, $J_{2'',F3''}$ = 11.5, $J_{2'',F4''}$ = 7.4, $J_{2'',6''}$ = 2.5 Hz, 1H, H-2''), 7.42 (dt, $J_{5'',F4''}$ = 10.7, $J_{5'',F3''}$ = $J_{5'',6''}$ = 8.7 Hz, 1H, H-5''); **^{13}C NMR** (126 MHz, DMSO-d_6, T = 100 °C) δ 19.56, 19.61, 19.67, 19.72, 19.77, 19.84 (**C**H_3CO-2,3,6,2',3',4',6'), 22.14 (**C**H_3CON), 60.51 (CH_2-6'), 61.29 (CH_2-6), 66.93 (CH-4'), 68.34 (CH-2), 68.91 (CH-2'), 69.59 (CH-5'), 70.12 (CH-3'), 73.25 (CH-3), 73.59 (CH-5), 74.89 (CH-4), 80.15 (CH-1), 99.27 (CH-1'), 116.83 (d, $J_{C,F4''}$ = 18.0 Hz, CH-5''), 118.78 (d, $J_{C,F3''}$ = 17.9 Hz, CH-2''), 126.54 (dd, $J_{C,F3''}$ = 6.4, $J_{C,F4''}$ = 3.2 Hz, CH-6''), 133.64 (dd, $J_{C,F4''}$ = 7.9, $J_{C,F3''}$ = 3.5 Hz, C-1''), 148.54 (dd, $J_{C,F3''}$ = 247.7, $J_{C,F4''}$ = 13.3 Hz, CF-3''), 148.98 (dd, $J_{C,F3''}$

= 247.7, 12.3 Hz), 168.28, 168.32 (CH_3**C**O-2,2'), 168.63, 168.68 (CH_3**C**O-3,3'), 169.09, 169.11 (CH_3**C**O-4',6'), 169.41 (CH_3**C**O-6), 170.08 (CH_3**C**ON); **^{19}F NMR** (471 MHz, DMSO-d_6, T = 100 °C) δ -134.83 – -134.33 (bs, 1F), -133.14 – -132.54 (bs, 1F); **IR** ($CHCl_3$) 1753, 1687, 1609, 1516, 1434, 1371, 1140, 1115, 1075, 1055, 601 cm^{-1}; **HRMS** (ESI) $[M+Na]^+$ *m/z* calcd for $C_{34}H_{41}O_{18}NF_2Na$: 812.2184, found: 812.2178; $[M+H]^+$ *m/z* calcd for $C_{34}H_{42}O_{18}NF_2$: 790.2365, found: 790.2361.

*N-[2,3,6-Tri-O-acetyl-4-O-(2,3,4,6-tetra-O-acetyl-β-D-galactopyranosyl)-β-D-glucopyranosyl]-N-(3,4,5-trifluorophenyl)acetamide (**2i**)*

Following General procedure A, compound **1** (0.636 g, 1.0 mmol) was reacted with 3,4,5-trifluoroaniline (0.250 g, 1.7 mmol) for 3 days. The crude product was then subjected to General procedure B, affording product **2h** (0.571 g, 71%) as a yellow foam. $[\alpha]_D^{20}$ = +38.5 (*c* 0.5 in $CHCl_3$); **^{1}H NMR** (500 MHz, DMSO-d_6, T = 100 °C) δ 1.90 (s, 3H, CH_3CO), 1.91 (s, 3H, CH_3CO), 1.98 (s, 3H, CH_3CO), 1.99 (s, CH_3CON), 2.01 (s, CH_3CO), 2.07 (s, CH_3CO), 2.09 (s, CH_3CO), 3.76 (dd, $J_{4,5}$ = 9.7, $J_{4,3}$ = 9.3 Hz, 1H, H-4), 3.96 (ddd, $J_{5,4}$ = 9.8, $J_{5,6a}$ = 5.1, $J_{5,6b}$ = 2.2 Hz, 1H, H-5), 4.00 (dd, J_{gem} = 11.2, $J_{6'a,5'}$ = 6.7 Hz, 1H, H-6'a), 4.04 (dd, J_{gem} = 11.2, $J_{6'b,5'}$ = 6.1 Hz, 1H, H-6'b), 4.12 (dd, J_{gem} = 12.1, $J_{6a,5}$ = 5.1 Hz, 1H, H-6a), 4.16 (ddd, $J_{5',6'a}$ = 6.8, $J_{5',6'b}$ = 6.2, $J_{5',4'}$ = 1.3 Hz, 1H, H-5'), 4.46 (dd, J_{gem} = 12.1, $J_{6b,5}$ = 2.2 Hz, 1H, H-6b), 4.48 (t, $J_{2,3}$ = $J_{2,1}$ = 9.4 Hz, 1H, H-2), 4.74 (d, $J_{1',2'}$ = 7.9 Hz, 1H, H-1'), 4.87 (dd, $J_{2',3'}$ = 10.3, $J_{2',1'}$ = 7.9 Hz, 1H, H-2'), 5.13 (dd, $J_{3',2'}$ = 10.3, $J_{3',4'}$ = 3.6 Hz, 1H, H-3'), 5.23 (t, $J_{3,4}$ = $J_{3,2}$ = 9.2 Hz, 1H, H-3), 5.26 (dd, $J_{4',3'}$ = 4.1, $J_{4',5'}$ = 1.4 Hz, 1H, H-4'), 5.94 (d, $J_{1,2}$ = 9.6 Hz, 1H, H-1), 7.11 (dd, $J_{2'',F3''}$ = 8.8, $J_{2'',F4''}$ = 6.5 Hz, 2H, H-2''); **^{13}C NMR** (126 MHz, DMSO-d_6, T = 100 °C) δ 19.51, 19.56, 19.61, 19.63, 19.67, 19.72, 19.74 (7 × **C**H_3CO-2,3,6,2',3',4',6'), 22.05 (**C**H_3CON), 60.51 (CH_2-6'), 61.18 (CH_2-6), 66.94 (CH-4'), 68.24 (CH-2), 68.94 (CH-2'), 69.62 (CH-5'), 70.12 (CH-3'), 73.14 (CH-3), 73.66 (CH-5), 74.75 (CH-4), 80.29 (CH-1), 99.26 (CH-1'), 114.82 (d, $J_{C,F}$ = 21.9 Hz, CH-2''), 132.84 – 132.95 (m, C-1''), 137.53 – 139.67 (m, CF-4''), 149.42 (ddd, J_{CF} = 248.0, J_{CF} = 9.6, J_{CF} = 4.7 Hz, CF-3''), 168.28, 168.41, 168.57, 168.64, 169.05, 169.07, 169.36 (7 × CH_3**C**O-2,3,6,2',3',4',6'), 169.93 (CH_3**C**ON); **^{19}F NMR** (471 MHz, DMSO-d_6, T = 100 °C) δ -161.86 – -161.51 (m, 2F, F-3''), -134.94 – -134.64 (m, 1F, F-4''); **IR** ($CHCl_3$) 3091, 2984, 2939, 2876, 1754, 1695, 1622, 1528, 1445, 1428, 1369, 1230, 1172, 1052, 1021, 975, 837, 699, 635, 601, 583 cm^{-1}; **HRMS** (ESI) $[M+Na]^+$ *m/z* calcd for $C_{34}H_{40}O_{18}NF_3Na$: 830.2090, found: 830.2088.

*N-[2,3,6-Tri-O-acetyl-4-O-(2,3,4,6-tetra-O-acetyl-β-D-galactopyranosyl)-β-D-glucopyranosyl]-N-(4-chlorophenyl)acetamide (**2j**)*

Following General procedure A, compound **1** (0.320 g, 0.50 mmol) was reacted with 4-chloroaniline (0.130 g, 0.85 mmol) for 3 days. The crude product was then subjected to General procedure B, affording product **2j** (0.241 g, 61%) as a white foam. $[\alpha]_D^{20}$ = +24.0 (*c* 0.2 in $CHCl_3$); **^{1}H NMR** (500 MHz, DMSO-d_6, T = 100 °C) δ 1.89 (s, 3H, CH_3CON), 1.90 (s, 6H, CH_3CO-3,3'), 1.97 (s, 6H, CH_3CO-2,4'), 2.00 (s, 3H, CH_3CO-2'), 2.08 (s, 3H, CH_3CO-6), 2.08 (s, 3H, CH_3CO-6'), 3.69 (t, $J_{4,5}$ = $J_{4,3}$ = 9.5 Hz, 1H, H-4), 3.95 (ddd, $J_{5,4}$ = 9.5, $J_{5,6a}$ = 5.3, $J_{5,6b}$ = 2.1 Hz, 1H, H-5), 4.00 (dd, J_{gem} = 11.2, $J_{6a',5'}$ =6.5 Hz, 1H, H-6a'), 4.03 (dd, J_{gem} = 11.2, $J_{6b,5'}$ = 6.5 Hz, 1H, H-6b'), 4.08 (dd, J_{gem} = 12.0,

$J_{6a,5}$ = 5.3 Hz, 1H, H-6a), 4.16 (td, $J_{5',6a'}$ = $J_{5',6b'}$ = 6.5, $J_{5',4'}$ =1.4 Hz, 1H, H-5'), 4.43 (t, $J_{2,1}$ = $J_{2,3}$ = 9.6 Hz, 1H, H-2), 4.46 (dd, J_{gem} = 12.0, $J_{6b,5}$ =2.1 Hz, 1H, H-6b), 4.73 (d, $J_{1',2'}$ = 8.0 Hz, 1H, H-1'), 4.87 (dd, $J_{2',3'}$ = 10.3, $J_{2',1'}$ = 8.0 Hz, 1H, H-2'), 5.13 (dd, $J_{3',2'}$ = 10.3, $J_{3',4'}$ = 3.6 Hz, 1H, H-3'), 5.21 (t, $J_{3,2}$ = $J_{3,4}$ =9.5 Hz, 1H, H-3), 5.25 (dd, $J_{4',3'}$ = 3.6, $J_{4',5'}$ =1.4 Hz, 1H, H-4'), 5.95 (d, $J_{1,2}$ = 9.5 Hz, 1H, H-1), 7.17 (d, $J_{3'',2''}$ = 8.6 Hz, 2H, H-3''), 7.44 (d, $J_{2'',3''}$ = 8.6 Hz, 1H, H-2''); **^{13}C NMR** (126 MHz, DMSO-d_6, T = 100 °C) δ 19.53, 19.58, 19.64, 19.65, 19.70, 19.75, 19.87 (**C**H_3CO-2,3,6,2',3',4',6'), 22.17 (**C**H_3CON), 60.50 (CH_2-6'), 61.38 (CH_2-6), 66.92 (CH-4'), 68.47 (CH-2), 68.90 (CH-2'), 69.59 (CH-5'), 70.11 (CH-3'), 73.32 (CH-3), 73.58 (CH-5), 74.94 (CH-4), 80.15 (CH-1), 99.25 (CH-1'), 128.41 (CH-2''), 130.97 (CH-3''), 132.65 (C-4''), 135.94 (C-1''), 168.13 (CH_3**C**O-2), 168.29 (CH_3**C**O-2'), 168.63 (CH_3**C**O-3), 168.65 (CH_3**C**O-3'), 169.07 (CH_3**C**O-6'), 169.08 (CH_3**C**O-4'), 169.39 (CH_3**C**O-6), 169.99 (CH_3**C**ON); **IR** ($CHCl_3$) 1753, 1682, 1493, 1371, 1230, 1172, 1055, 1018, 980, 590, 494 cm^{-1}; **HRMS** (ESI) $[M+Na]^+$ *m/z* calcd for $C_{34}H_{42}O_{18}NClNa$: 810.1983, found: 810.1982; $[M+H]^+$ *m/z* calcd for $C_{34}H_{43}O_{18}NCl$: 788.2163, found: 788.2163.

*N-[2,3,6-Tri-O-acetyl-4-O-(2,3,4,6-tetra-O-acetyl-β-D-galactopyranosyl)-β-D-glucopyranosyl]-N-(3,4-dichlorophenyl)acetamide (**2k**)*

Following General procedure A, compound **1** (0.320 g, 0.50 mmol) was reacted with 3,4-dichloroaniline (0.130 g, 0.85 mmol) for 3 days. The crude product was then subjected to General procedure B, affording product **2k** (0.290 g, 71%) as a white foam. $[\alpha]_D^{20}$ = +22.3 (*c* 0.3 in $CHCl_3$); **^{1}H NMR** (500 MHz, DMSO-d_6, T = 100 °C): 1.90, 1.90 (2 × s, 6H CH_3CO-3,3'), 1.95 (s, 3H, CH_3CON), 1.97, 1.98, 2.00, 2.08 (4 × s, 15H, CH_3CO-2,6,2',4',6'), 3.73 (t, $J_{4,5}$ = $J_{4,3}$ = 9.5 Hz, 1H, H-4), 3.97 (ddd, $J_{5,4}$ = 9.5, $J_{5,6b}$ = 5.0, $J_{5,6a}$ = 2.1 Hz, 1H, H-5), 4.01 (dd, J_{gem} = 11.2, $J_{6'b,5'}$ = 7.0 Hz, 1H, H-6'b), 4.04 (dd, J_{gem} =11.2, $J_{6'a,5'}$ = 6.2 Hz, 1H, H-6'a), 4.10 (dd, J_{gem} =12.1, $J_{6b,5}$ = 5.0 Hz, 1H, H-6b), 4.16 (ddd, $J_{5',6'b}$ = 7.0, $J_{5',6'a}$ = 6.2, $J_{5',4'}$ = 1.3 Hz, 1H, H-5'), 4.44 (t, $J_{2,3}$ = $J_{2,1}$ = 9.5 Hz, 1H, H-2), 4.46 (dd, J_{gem} = 12.1, $J_{6a,5}$ = 2.1, Hz 1H, H-6a), 4.73 (d, $J_{1',2'}$ = 7.9 Hz, 1H, H-1'), 4.87 (dd, $J_{2',3'}$ = 10.3, $J_{2',1'}$ = 7.9 Hz, 1H, H-2'), 5.13 (dd, $J_{3',2'}$ = 10.3, $J_{3',4'}$ = 3.7 Hz, 1H, H-3'), 5.23 (t, $J_{3,2}$ = $J_{3,4}$ = 9.5 Hz, 2H, H-3), 5.25 (dd, $J_{4',3'}$ = 3.7 $J_{4',5'}$ = 1.3 Hz, 1H, H-4'), 5.95 (d, $J_{1,2}$ = 9.5 Hz, 1H, H-1), 7.15 (dd, $J_{6'',5''}$ = 8.5, $J_{6'',2''}$ = 2.4 Hz, 1H, H-6''), 7.41 (d, $J_{2'',6''}$ = 2.4 Hz, 1H, H-2''), 7.63 (d, $J_{5'',6''}$ = 8.5 Hz, 1H, H-5''); **^{13}C NMR** (126 MHz, DMSO-d_6, T = 100 °C) δ 19.53, 19.58, 19.65, 19.68, 19.70, 19.86 (**C**H_3CO-2,3,6,2',3',4',6'), 22.10 (**C**H_3CON), 60.49 (CH_2-6'), 61.26 (CH_2-6), 66.92 (CH-4'), 68.33 (CH-2), 68.90 (CH-2'), 69.59 (CH-5'), 70.11 (CH-3'), 73.19 (CH-3), 73.60 (CH-5), 74.83 (CH-4), 80.32 (CH-1), 99.25 (CH-1'), 129.58 (CH-6''), 130.14 (CH-5''), 130.88 (C-3'', C-4''), 131.23 (CH-2''), 136.93 (C-1''), 168.22, 168.29, 168.61, 168.65, 169.06, 169.08 (CH_3**C**O-2,3,2'3',4',6'), 169.37 (CH_3**C**O-6), 169.93 (CH_3**C**ON); **IR** ($CHCl_3$) 1754, 1686, 1590, 1561, 1473, 1371, 1230, 1133, 1056, 980, 606, 547 cm^{-1}; **HRMS** (ESI) $[M+Na]^+$ *m/z* calcd for $C_{34}H_{41}O_{18}NCl_2Na$: 844.1593, found: 844.1591; $[M+H]^+$ *m/z* calcd for $C_{34}H_{42}O_{18}NCl_2$: 822.1774, found: 822.1772.

*N-[2,3,6-Tri-O-acetyl-4-O-(2,3,4,6-tetra-O-acetyl-β-D-galactopyranosyl)-β-D-glucopyranosyl]-N-(4-iodophenyl)acetamide (**2l**)*

Following General procedure A, compound **1** (0.350 g, 0.55 mmol) was reacted with 4-iodoaniline (0.206 mL, 0.94 mmol) for 3 days. The crude product was then subjected to General procedure B, affording product **2l** (0.241 g, 61%) as a white foam. $[\alpha]_D^{20}$ = +13.1 (*c* 0.3 in $CHCl_3$); ^{1}**H NMR** (500 MHz, DMSO-d_6, T = 100 °C) δ 1.88 (bs, 3H, CH_3CON), 1.90 (s, 6H, CH_3CO), 1.96 (s, 3H, CH_3CO), 1.97 (s, 3H, CH_3CO), 2.00 (s, 3H, CH_3CO), 2.07 (s, 3H, CH_3CO), 2.08 (s, 3H, CH_3CO), 3.69 (t, $J_{4,3} = J_{4,5}$ = 9.5 Hz, 1H, H-4), 3.94 (ddd, $J_{5,4}$ = 9.9, $J_{5,6a}$ = 5.3, $J_{5,6b}$ = 2.2 Hz, 1H, H-5), 4.00 (dd, J_{gem} = 11.2, $J_{6'a,5'}$ = 6.8 Hz, 1H, H-6'a), 4.03 (dd, J_{gem} = 11.2, $J_{6'b,5'}$ = 6.1 Hz, 1H, H-6'b), 4.07 (dd, J_{gem} = 12.5, $J_{6a,5}$ = 5.7 Hz, 1H, H-6a), 4.16 (td, $J_{5',6a'} = J_{5',6b'}$ = 6.4, $J_{5',4'}$ =1.3 Hz, 1H, H-5'), 4.41 (t, $J_{2,3} = J_{2,1}$ = 9.2 Hz, 1H, H-2), 4.45 (dd, J_{gem} = 12.1, $J_{6b,5}$ = 2.2 Hz, 1H, H-6b), 4.73 (d, $J_{1',2'}$ = 8.0 Hz, 1H, H-1'), 4.87 (dd, $J_{2',3'}$ = 10.2, $J_{2',1'}$ = 7.9 Hz, 1H, H-2'), 5.13 (dd, $J_{3',2'}$ = 10.2, $J_{3',4'}$ = 3.6 Hz, 1H, H-3'), 5.21 (t, $J_{3,4} = J_{3,2}$ = 9.2 Hz, 1H, H-3), 5.24 (dd, $J_{4',3'}$ = 3.6, $J_{4',5'}$ = 1.2 Hz, 1H, H-4'), 5.95 (d, $J_{1,2}$ = 9.6 Hz, 1H, H-1), 6.95 (d, $J_{2'',3''}$ = 8.6 Hz, 1H, H-2''), 7.75 (d, $J_{3'',2''}$ = 8.6 Hz, 1H, H-3''); ^{13}**C NMR** (126 MHz, , DMSO-d_6, T = 100 °C) δ 19.56, 19.61, 19.66, 19.70, 19.72, 19.77, 19.91 (7 × **C**H_3CO-2,3,6,2',3',4',6'), 22.21 (**C**H_3CON), 60.49 (CH_2-6'), 61.37 (CH_2-6), 66.91 (CH-4'), 68.49 (CH-2), 68.89 (CH-2'), 69.58 (CH-5'), 70.11 (CH-3'), 73.31 (CH-3), 73.56 (CH-5), 74.94 (CH-4), 80.10 (CH-1), 93.63 (C-4''), 99.25 (CH-1'), 131.42 (CH-2''), 136.91 (C-1''), 137.40 (CH-3''), 168.13, 168.31, 168.64, 168.67, 169.08, 169.10, 169.40 (7 × CH_3**C**O-2,3,6,2',3',4',6'), 169.90 (CH_3**C**ON); **IR** ($CHCl_3$) 2874, 1753, 1684, 1486, 1430, 1371, 1229, 1056, 1011, 405 cm^{-1}; **HRMS** (ESI) $[M+Na]^+$ *m/z* calcd for $C_{34}H_{42}O_{18}NINa$: 902.1339, found: 902.1337.

*N-[2,3,6-Tri-O-acetyl-4-O-(2,3,4,6-tetra-O-acetyl-β-D-galactopyranosyl)-β-D-glucopyranosyl]-N-(4-methoxyphenyl)acetamide (**2m**)*

Following General procedure A, compound **1** (0.636 g, 1.0 mmol) was reacted with 4-methoxyaniline (0.182 g, 1.7 mmol) for 3 days. The crude product was then subjected to General procedure B, affording product **2m** (0.345 g, 44%) as a white foam. $[\alpha]_D^{20}$ = +18.7 (*c* 0.3 in $CHCl_3$); ^{1}**H NMR** (500 MHz, DMSO-d_6, T = 100 °C): δ 1.80 (s, 3H, CH_3CON), 1.89, 1.89, 1.96, 1.97, 1.99, 2.06, 2.07 (s, 7 x 3H, CH_3CO-2,3,6,2',3',4',6'), 3.64 (dd, $J_{4,5}$ = 9.8 $J_{4,3}$ = 9.3 Hz, 1H, H-4), 3.79 (s, 3H, Ar-OCH_3), 3.89 (ddd, $J_{5,3}$ = 9.8, $J_{5,6a}$ = 5.2, $J_{5,6b}$ = 2.2 Hz, 1H, H-5), 3.99 (dd, J_{gem} = 11.2, $J_{6'a,5'}$ = 6.8 Hz, 1H, H-6'a), 4.02 (dd, J_{gem} = 11.2, $J_{6'b,5'}$ = 6.2 Hz, 1H, H-6'b), 4.06 (dd, J_{gem} = 12.1, $J_{6a,5}$ = 5.2 Hz, 1H, H-6a), 4.14 (ddd, $J_{5',6'a}$ = 6.8, $J_{5',6'b}$ = 6.1, $J_{5',4'}$ = 1.2 Hz, 1H, H-5'), 4.42 (t, $J_{2,1} = J_{2,3}$ = 9.3 Hz, 1H, H-2), 4.46 (dd, J_{gem} = 12.1, $J_{6b,5}$ = 2.2 Hz, 1H, H-6b), 4.72 (d, $J_{1',2'}$ = 7.9 Hz, 1H, H-1'), 4.86 (dd, *J* = 10.3, 7.9 Hz, 1H,), 5.11 (dd, *J* = 10.3, 3.6 Hz, 1H), 5.18 (t, *J* = 9.3 Hz, 1H), 5.23 (dd, *J* = 3.6, 1.2 Hz, 1H,), 5.94 (d, $J_{1,2}$ = 9.3 Hz, 1H, H-1), 6.92 (d, $J_{2'',3''}$ = 9.0 Hz, 2H, H-2''), 7.04 (d, $J_{3'',2''}$ = 9.0 Hz, 2H, H-3''); ^{13}**C NMR** (126 MHz, DMSO-d_6, T = 100 °C): δ 19.64, 19.69, 19.75, 19.78, 19.83, 19.92, 20.01 (7 x **C**H_3CO-2,3,6,2',3',4',6'), 22.31 (**C**H_3CON), 55.06 (Ar-O**C**H_3), 60.61 (CH_2-6'), 61.45 (CH_2-6), 67.03 (CH-4'), 68.72 (CH-2), 69.01 (CH-2'), 69.69 (CH-5'), 70.23 (CH-3'), 73.58 (CH-3), 73.63 (CH-5), 75.08 (CH-4), 79.93 (CH-1), 99.34 (CH-1'), 113.93 (CH-2''), 129.69 (C-1''), 130.46 (CH-3''), 158.83 (C-4''), 168.28 (CH_3**C**O-2), 168.50 (CH_3**C**O-2'), 168.84 (CH_3**C**O-3,3'), 169.27

(CH_3**C**O-4',6'), 169.57 (CH_3CO-6), 170.68 (CH_3**C**ON); **IR** ($CHCl_3$) 2917, 2840, 1753, 1671, 1608, 1584, 1512, 1465, 1443, 1429, 1370, 1249, 1055, 1040, 980, 913, 841, 593 cm^{-1}; **HRMS** (ESI) $[M+Na]^+$ *m/z* calcd for $C_{35}H_{45}O_{19}NNa$: 806.2478, found: 806.2474; $[M+H]^+$ *m/z* calcd for $C_{35}H_{46}O_{19}N$: 784.2659, found: 784.2656.

*N-[2,3,6-Tri-O-acetyl-4-O-(2,3,4,6-tetra-O-acetyl-β-D-galactopyranosyl)-β-D-glucopyranosyl]-N-(3,4-dimethoxyphenyl)acetamide (**2n**)*

Following General procedure A, compound **1** (0.320 g, 0.50 mmol) was reacted with 3,4-dimethoxyaniline (0.130 g, 0.85 mmol) for 3 days. The crude product was then subjected to General procedure B, affording product **2n** (0.313 g, 77%) as a pink foam. $[\alpha]_D^{20}$ = +11.2 (*c* 0.3 in $CHCl_3$); **^{1}H NMR** (500 MHz, DMSO-d_6, T = 100 °C) δ 1.84 (s, 3H, CH_3CON), 1.90 (s, 6H, 2 × CH_3CO), 1.97 (s, 3H, CH_3CO), 1.99 (s, 3H, CH_3CO), 2.00 (s, 3H, CH_3CO), 2.06 (s, 3H, CH_3CO), 2.08 (s, 3H, CH_3CO), 3.68 (t, $J_{4,5}$ = $J_{4,3}$ = 9.6 Hz, 1H, H-4), 3.76 (s, 3H, OCH_3-3''), 3.81 (s, 3H, OCH_3-4''), 3.86 – 3.97 (m, 1H, H-5), 4.00 (dd, J_{gem} = 11.2, $J_{6'a,5'}$ = 6.6 Hz, 1H, H-6'a), 4.02 (dd, J_{gem} = 11.5, $J_{6'b,5'}$ = 6.1 Hz, 1H, H-6'b), 4.10 (dd, J_{gem} = 12.3, $J_{6a,5}$ = 5.0 Hz, 1H, H-6a), 4.16 (td, $J_{5',6'a}$ = $J_{5',6'b}$ = 6.4, $J_{5',4'}$ = 1.2 Hz, 1H, H-5'), 4.39 – 4.50 (m, 1H, H-6b), 4.47 (t, $J_{2,3}$ = $J_{2,1}$ = 9.4 Hz, 1H, H-2), 4.73 (d, $J_{1',2'}$ = 8.0 Hz, 1H), 4.87 (dd, $J_{2',3'}$ = 10.2, $J_{2',1'}$ = 7.9 Hz, 1H, H-2'), 5.12 (dd, $J_{3',2'}$ = 10.3, $J_{3',4'}$ = 3.7 Hz, 1H, H-3'), 5.19 (t, $J_{3,4}$ = $J_{3,2}$ = 9.1 Hz, 1H, H-3), 5.24 (dd, $J_{4',3'}$ = 3.6, $J_{4',5'}$ = 1.2 Hz, 1H, H-4'), 5.96 (d, $J_{1,2}$ = 9.9 Hz, 1H, H-1), 6.66 – 6.73 (m, 2H, H-2'',6''), 6.94 (d, $J_{5'',6''}$ = 8.7 Hz, 1H, H-5''); **^{13}C NMR** (126 MHz, DMSO-d_6, T = 100 °C) δ 19.54, 19.59, 19.65, 19.67, 19.74, 19.86, 19.91 (**C**H_3CO-2,3,6,2',3',4',6'), 22.20 (**C**H_3CON), 55.34 (OCH_3-3''), 55.53 (OCH_3-4''), 60.51 (CH_2-6), 61.48 (CH_2-6'), 66.92 (CH-4'), 68.64 (CH-2), 68.90 (CH-2'), 69.58 (CH-5'), 70.13 (CH-3'), 73.54 (CH-3,5), 74.97 (CH-4), 79.59 (CH-1), 99.21 (CH-1'), 111.84 (CH-5''), 114.22 (CH-2''), 121.75 (CH-6''), 129.79 (C-1''), 148.63 (C-3''), 148.81 (C-4''), 168.22, 168.29, 168.65, 168.66, 169.07, 169.08, 169.39 (CH_3**C**O-2,3,6,2'3',4',6'), 170.44 (CH_3**C**ON); **IR** ($CHCl_3$) 2939, 2842, 1753, 1675, 1595, 1514, 1465, 1452, 1371, 1243, 1240, 1240, 1056, 1055, 601, 548 cm^{-1}; **HRMS** (ESI) $[M+Na]^+$ *m/z* calcd for $C_{36}H_{47}O_{20}NNa$: 836.2584, found: 836.2577; $[M+H]^+$ *m/z* calcd for $C_{36}H_{48}O_{20}N$: 814.2764, found: 814.2762.

*N-[2,3,6-Tri-O-acetyl-4-O-(2,3,4,6-tetra-O-acetyl-β-D-galactopyranosyl)-β-D-glucopyranosyl]-N-(3,4,5-trimethoxyphenyl)acetamide (**2o**)*

Following General procedure A, compound **1** (0.320 g, 0.50 mmol) was reacted with 3,4,5-trimethoxyaniline (0.156 g, 0.85 mmol) for 3 days. The crude product was then subjected to General procedure B, affording product **2o** (0.329 g, 78%) as a red foam. $[\alpha]_D^{20}$ = +6.2 (*c* 0.3 in $CHCl_3$); **^{1}H NMR** (500 MHz, DMSO-d_6, T = 100 °C): 1.88 (s, 3H, CH_3CON); 1.90, 1.91, 1.97, 2.00, 2.04, 2.09 (6 × s, 21H, CH_3CO-2,3,6,2',3',4',6'); 3.73 (dd, 1H, $J_{4,5}$ = 10.0, $J_{4,3}$ = 9.2, H-4); 3.74 (s, 3H, CH_3O-*p*-$C_6H_2(OMe)_3$); 3.77 (s, 6H, CH_3O-*m*-$C_6H_2(OCH_3)_3$); 3.92 (ddd, 1H, $J_{5,4}$ = 10.0, $J_{5,6}$ = 5.4, 2.3, H-5); 4.01 (dd, 1H, $J_{6'b,6'a}$ = 11.3, $J_{6'b,5'}$ = 6.8, H-6'b); 4.04 (dd, 1H, $J_{6'a,6'b}$ = 11.3, $J_{6'a,5'}$ = 6.2, H-6'a); 4.13 – 4.18 (m, 2H, H-5',6b); 4.06 – 4.15 (m, 3H, H-6b,6'); 4.40 (dd, 1H, $J_{6a,6b}$ = 12.1, $J_{6a,5}$ = 2.1,

H-6a); 4.52 (t, 1H, $J_{2,1}$ = $J_{2,3}$ = 9.2, H-2); 4.73 (d, 1H, $J_{1',2'}$ = 7.9, H-1'); 4.87 (dd, 1H, $J_{2',3'}$ = 10.2, $J_{2',1'}$ = 7.9, H-2'); 5.11 (dd, 1H, $J_{3',2'}$ = 10.2, $J_{3',4'}$ = 3.6, H-3'); 5.19 (t, 1H, $J_{3,2}$ = $J_{3,4}$ = 9.2, H-3); 5.25 (dd, 1H, $J_{4',3'}$ = 3.6, $J_{4',5'}$ = 1.0, H-4'); 5.96 (d, 1H, $J_{1,2}$ = 9.2, H-1); 6.45 (s, 2H, H-*o*-$C_6H_2(OCH_3)_3$); **^{13}C NMR** (126 MHz, DMSO-d_6, T = 100 °C): 19.72, 19.77, 19.84, 19.92, 19.97, 20.13 (**C**H_3CO-2,3,6,2',3',4',6'); 22.36 (**C**H_3CON); 55.89 (CH_3-*m*-$C_6H_2(OCH_3)_3$); 59.79 (CH_3-*p*-$C_6H_2(OCH_3)_3$); 60.69 (CH_2-6'); 61.74 (CH_2-6); 67.11 (CH-4'); 68.73 (CH-2); 69.08 (CH-2'); 69.77 (CH-5'); 70.33 (CH-3'); 73.75 (CH-3); 73.81 (CH-5); 75.10 (CH-4); 79.90 (CH-1); 99.40 (CH-1'); 108.21 (CH-*o*-$C_6H_2(OCH_3)_3$); 132.35 (C-*i*-$C_6H_2(OCH_3)_3$); 135.67 (C-*p*-$C_6H_2(OCH_3)_3$); 152.56 (C-*m*-$C_6H_2(OCH_3)_3$); 168.49, 168.50 (CH_3**C**O-2,2'); 168.85, 168.87 (CH_3**C**O-3,3'); 169.27, 169.29 (CH_3**C**O-4',6'); 169.59 (CH_3**C**O-6); 170.47 (CH_3**C**ON); **IR** ($CHCl_3$) 2941, 2841, 1753, 1682, 1603, 1598, 1507, 1465, 1433, 1411, 1370, 1240, 1132, 1056, 913, 608, 529 cm^{-1}; **HRMS** (ESI) $[M+Na]^+$ *m/z* calcd for $C_{37}H_{49}O_{21}NNa$: 866.2689, found: 866.2690.

*N-[2,3,6-Tri-O-acetyl-4-O-(2,3,4,6-tetra-O-acetyl-β-D-galactopyranosyl)-β-D-glucopyranosyl]-N-(1,3-benzodioxol-5-yl)acetamide (**2p**)*

Following General procedure A, compound **1** (0.320 g, 0.50 mmol) was reacted with 1,3-benzodioxol-5-amine (0.117 g, 0.85 mmol) for 3 days. The crude product was then subjected to General procedure B, affording crude product **2p**, which contained several aromatic impurities. The material was used without further purification in the subsequent reaction step.

*N-[2,3,6-Tri-O-acetyl-4-O-(2,3,4,6-tetra-O-acetyl-β-D-galactopyranosyl)-β-D-glucopyranosyl]-N-[2-(ethylcarboxy)phenyl]acetamide (**2q**)*

Following General procedure A, compound **1** (1.273 g, 2.0 mmol) was reacted with ethyl 2-aminobenzoate (0.368 mL, 3.0 mmol) for 7 days. The reaction afforded intermediate *N*-[2,3,6-Tri-*O*-acetyl-4-*O*-(2,3,4,6-tetra-*O*-acetyl-β-D-galactopyranosyl)-β-D-glucopyranosyl]-*N*-[2-(ethylcarboxy)phenyl]amine (0.450 g, 29%) along with unreacted starting material **1** (0.513 g, 40%). The isolated intermediate (0.102 g, 0.131 mmol) was then subjected to General procedure B, affording product **2q** (0.094 g, 87%) as a white foam. $[\alpha]_D^{20}$ = −16.2 (*c* 0.2 in $CHCl_3$); **^{1}H NMR** (500 MHz, DMSO-d_6; T = 25 °C) δ 1.21 (t, $J_{CH3,CH2}$ = 7.2 Hz, 3H, OCH_2C**H$_3$**), 1.75 (s, 3H, CH_3CO), 1.87 (s, 3H, CH_3CO), 1.88 (s, 3H, CH_3CO), 2.02 (s, 3H, CH_3CO), 2.03 (s, 3H, CH_3CO), 2.07 (s, 3H, CH_3CO), 2.12 (s, 3H, CH_3CON), 3.42 (t, $J_{4,3}$ = $J_{4,5}$ = 9.6 Hz, 1H, H-4), 3.76 (dd, J_{gem} = 12.1, $J_{6'a,5'}$ = 5.6 Hz, 1H, H-6'a), 3.92 – 3.98 (m, 2H, H-5,6'b), 4.04 (dd, J_{gem} = 12.0, $J_{6a,5}$ = 7.1 Hz, 1H, H-6a), 4.09 – 4.20 (m, 4H, H-5',6b, OC**H$_2$**CH_3), 4.22 (t, $J_{2,1}$ = $J_{2,3}$ = 9.4 Hz, 1H, H-2), 4.67 (d, $J_{1',2'}$ = 8.1 Hz, 1H, H-1'), 4.79 (dd, $J_{2',3'}$ = 10.3, $J_{2',1'}$ = 8.0 Hz, 1H, H-2'), 5.07 (dd, $J_{3',2'}$ = 10.3, $J_{3',4'}$ = 3.6 Hz, 1H, H-3'), 5.18 (dd, $J_{4',3'}$ = 3.5, $J_{4',5'}$ = 1.3 Hz, 1H, H-4'), 5.20 (t, $J_{3,4}$ = $J_{3,2}$ = 9.2 Hz, 1H, H-3), 6.14 (d, $J_{1,2}$ = 9.6 Hz, 1H, H-1), 7.15 (dd, $J_{6'',5''}$ = 7.7, $J_{6'',4''}$ = 1.3 Hz, 1H), 7.57 (td, $J_{4'',5''}$ = $J_{4'',3''}$ = 7.5, $J_{4'',6''}$ = 1.3 Hz, 1H, H-4''), 7.62 (td, $J_{5'',6''}$ = $J_{5'',4''}$ = 7.6, $J_{5'',3''}$ = 1.8 Hz, 1H, H-5''), 7.77 (dd, $J_{3'',4''}$ = 7.6, $J_{3'',5''}$ = 1.8 Hz, 1H, H-3''); **^{13}C NMR** (126 MHz, DMSO-d_6; T = 25 °C) δ 13.44 (OCH_2**C**H_3), 20.19, 20.26, 20.32, 20.34, 20.38, 20.51, 20.60 (7 × **C**H_3CO-2,3,6,2',3',4',6'), 23.29 (**C**H_3CON), 60.65 (CH_2-6'), 60.79 (CH_2-6), 62.48

(OCH_2CH_3), 67.01 (CH-4'), 68.76 (CH-2), 69.05 (CH-2'), 69.54 (CH-5'), 70.36 (CH-3'), 73.36 (CH-3), 73.42 (CH-5), 75.34 (CH-5), 83.74 (CH-1), 99.65 (CH-1'), 129.39 (CH-6''), 130.41 (CH-4''), 132.09 (CH-3''), 132.38 (CH-5''), 133.40 (C-2''), 134.88 (C-1''), 166.05 (COOEt), 168.97, 169.29, 169.38, 169.48, 169.79, 169.86, 170.27, (7 x CH_3**C**O-2,3,6,2',3',4',6') 170.50 (CH_3**C**ON); **IR** ($CHCl_3$) 1753, 1720, 1685, 1601, 1578, 1489, 1453, 1369, 1289, 1234, 1056, 1020, 594, 549, 448 cm^{-1}; **HRMS** (ESI) $[M+Na]^+$ *m/z* calcd for $C_{37}H_{47}O_{20}NNa$: 848.2584, found: 848.2572.

*N-[2,3,6-Tri-O-acetyl-4-O-(2,3,4,6-tetra-O-acetyl-β-D-galactopyranosyl)-β-D-glucopyranosyl]-N-(3-carboxyphenyl)acetamide (**2r**)*

Following General procedure A, compound **1** (0.636 g, 1.0 mmol) was reacted with 3-aminobenzoic acid (0.233 g, 1.7 mmol) for 3 days. The crude product was then subjected to General procedure B, affording product **2r** (0.550 g, 69%) as a white foam. $[\alpha]_D^{20}$ = +27.8 (*c* 0.2 in $CHCl_3$); **^{1}H NMR** (500 MHz, DMSO-d_6, T = 100 °C) δ 1.88 (s, 3H, CH_3CO), 1.90 (s, 3H, CH_3CO), 1.91 (bs, 3H, CH_3CON), 1.96 (s, 3H, CH_3CO), 1.96 (s, 3H, CH_3CO), 2.00 (s, 3H, CH_3CO), 2.08 (s, 6H, 2 × CH_3CO), 3.70 (t, $J_{4,3} = J_{4,5}$ = 9.5 Hz, 1H, H-4), 3.96 (ddd, $J_{5,4}$ = 9.9, $J_{5,6a}$ = 5.0, $J_{5,6b}$ = 2.3 Hz, 1H, H-5), 3.99 (dd, J_{gem} = 11.2, $J_{6a',5'}$ = 6.5 Hz, 1H, H-6a'), 4.02 (dd, J_{gem} = 11.2, $J_{6b,5}$ = 6.5 Hz, 1H, H-6b'), 4.08 (dd, J_{gem} = 12.1, $J_{6a,5}$ = 5.1 Hz, 1H, H-6a), 4.15 (td, $J_{5',6a'} = J_{5',6b'}$ = 6.5, $J_{5',4'}$ = 1.2 Hz, 1H, H-5'), 4.39 (t, $J_{2,3} = J_{2,1}$ = 9.5 Hz, 1H, H-2), 4.46 (dd, J_{gem} = 12.1, $J_{6b,5}$ =2.2 Hz, 1H, H-6b), 4.73 (d, $J_{1',2'}$ = 7.9 Hz, 1H, H-1'), 4.86 (dd, $J_{2',3'}$ = 10.3, $J_{2',1'}$ = 7.9 Hz, 1H, H-2'), 5.12 (dd, $J_{3',2'}$ = 10.3, $J_{3',4'}$ = 3.6 Hz, 1H, H-3'), 5.21 (t, $J_{3,4} = J_{3,2}$ = 9.5 Hz, 1H, H-3), 5.24 (dd, $J_{4',3'}$ = 3.6, $J_{4',5'}$ = 1.2 Hz, 1H, H-4'), 5.96 (d, $J_{1,2}$ = 9.5 Hz, 1H, H-1), 7.37 (ddd, $J_{6'',5''}$ = 7.8, $J_{6'',2''}$ = 1.9, $J_{6'',4''}$ = 1.1 Hz, 1H, H-6''), 7.52 (t, $J_{5'',6''} = J_{5'',4''}$ = 7.8 Hz, 1H, H-5''), 7.73 (t, $J_{2'',6''} = J_{2'',4''}$ = 1.9 Hz, 1H, H-2''), 7.94 (ddd, $J_{4'',5''}$ = 7.8, $J_{4'',2''}$ = 1.9, $J_{4'',2''}$ = 1.1 Hz, 1H, H-4''); **^{13}C NMR** (126 MHz, DMSO-d_6, T = 100 °C) δ 19.54, 19.59, 19.66, 19.69, 19.81 (5 × **C**H_3CO-2,3,6,2',3',4',6'), 22.16 (**C**H_3CON), 60.50 (CH_2-6'), 61.33 (CH_2-6), 66.93 (CH-4'), 68.45 (CH-2), 68.91 (CH-2'), 69.59 (CH-5'), 70.14 (CH-3'), 73.33 (CH-3), 73.53 (CH-5), 74.92 (CH-4), 80.51 (CH-1), 99.24 (CH-1'), 128.58 (CH-4''), 128.68 (CH-5''), 129.89 (CH-2''), 131.70 (C-3''), 133.32 (CH-6''), 137.35 (C-1''), 165.90 (**C**OOH), 167.99 (CH_3CO-2), 168.32, 168.65, 168.68, 169.09, 169.11, 169.45 (5 x CH_3**C**O-3,6,2',3',4',6'), 170.09 (CH_3**C**ON); **IR** ($CHCl_3$) 3521, 3095, 2980, 2878, 2655, 2542, 1754, 1701, 1688, 1604, 1588, 1488, 1452, 1412, 1370, 1330, 1305, 1237, 1140, 1075, 1055, 1050, 979, 955, 914, 902, 701, 599 cm^{-1}; **HRMS** (ESI) $[M+Na]^+$ *m/z* calcd for $C_{35}H_{43}O_{20}NNa$: 820.2271, found: 820.2277.

*N-[2,3,6-Tri-O-acetyl-4-O-(2,3,4,6-tetra-O-acetyl-β-D-galactopyranosyl)f-β-D-glucopyranosyl]-N-(3-carboxyphenyl)amine (**S1**)*

Following General procedure A, compound **1** (1.23 g, 1.9 mmol) was reacted with 3-aminobenzoic acid (0.40 g, 3.0 mmol) for 5 days. The residue was purified by liquid column chromatography on silica gel (20:1 → 15:1 $CHCl_3$/EtOH) to afford product **S1** (1.27 g, 87%) as a white foam. The product was obtained as a mixture of anomers in 1:2 ratio (α/β). NMR assignments are reported for the β anomer. $[\alpha]_D^{20}$ = +12.3 (*c* 0.3 in $CHCl_3$); **^{1}H NMR** (500 MHz,

DMSO-d_6, T = 25 °C) δ 1.90 (s, 3H, CH_3CO), 1.93 (s, 3H, CH_3CO), 1.97 (s, 3H, CH_3CO), 2.00 (s, 3H, CH_3CO), 2.02 (s, 3H, CH_3CO), 2.02 (s, 3H, CH_3CO), 2.11 (s, 3H, CH_3CO), 3.77 (t, $J_{4,3}$ = $J_{4,5}$ = 9.4 Hz, 1H, H-4), 3.97 (ddd, $J_{5,4}$= 9.9, $J_{5,6a}$= 6.8, $J_{5,6b}$= 1.9 Hz, 1H, H-5), 4.01 – 4.05 (m, 3H, H-6b,6'a,6'b), 4.17 – 4.28 (m, 2H, H-6a,5'), 4.77 (d, $J_{1',2'}$ = 8.0 Hz, 1H, H-1'), 4.85 (dd, $J_{2,3}$ = 9.4, $J_{2,1}$ = 8.8 Hz, 1H, H-2), 4.87 (dd, $J_{2',3'}$ = 8.8, $J_{2',1'}$ = 7.9 Hz, 1H, H-2'), 5.11 – 5.17 (m, 2H, H-1,3'), 5.23 (t, $J_{3,2}$ = $J_{3,4}$ = 9.2 Hz, 1H, H-3), 5.24 (dd, $J_{4',3'}$ = 3.6, $J_{4',5'}$ = 0.9 Hz, 1H, H-4'), 6.71 (d, $J_{N,1}$ = 9.3 Hz, 1H, NH), 6.94 (ddd, $J_{6'',5''}$ = 8.0, $J_{6'',2''}$ = 2.4, $J_{6'',4''}$ =1.3 Hz, 1H, H-6''), 7.22 (t, $J_{5'',4''}$ = $J_{5'',6''}$ = 7.8 Hz, 1H, H-5''), 7.26 (dt, $J_{4'',5''}$ = 7.7, $J_{4'',2''}$ = 1.4 Hz, 1H, H-4''), 7.33 (dd, $J_{2'',6''}$ = 2.4, $J_{2'',4''}$ = 1.7 Hz, 1H, H-2''); **^{13}C NMR** (126 MHz, DMSO-d_6, T = 25 °C) δ 20.34 (**C**H_3CO), 20.35 (**C**H_3CO), 20.40 (**C**H_3CO), 20.49 (**C**H_3CO), 20.53 (**C**H_3CO), 20.54 (**C**H_3CO), 20.61 (**C**H_3CO), 60.93 (CH_2-6'), 62.46 (CH_2-6), 67.09 (CH-4'), 68.88 (CH-2'), 69.66 (CH-5'), 70.36 (CH-3'), 71.13 (CH-2), 72.23 (CH-5), 73.71 (CH-3), 76.66 (CH-4), 81.30 (CH-1), 99.91 (CH-1'), 114.76 (CH-2''), 117.87 (CH-6''), 119.02 (CH-4''), 128.88 (CH-5''), 131.50 (C-3''), 146.31 (C-1''), 167.66 (COOH), 169.09 (CH_3**C**O), 169.35 (CH_3**C**O), 169.48 (CH_3**C**O), 169.54 (CH_3**C**O), 169.92 (CH_3**C**O), 169.94 (CH_3**C**O), 170.24 (CH_3**C**O). **IR** ($CHCl_3$) 3523, 3418, 3373, 2958, 1751, 1696, 1611, 1594, 1528, 1489, 1451, 1414, 1370, 1280, 1235, 1058, 997, 682, 600 cm^{-1}; **HRMS** (ESI) $[M-H]^-$ *m/z* calcd for $C_{33}H_{40}O_{19}N$: 754.2200, found: 754.2194.

*N-[2,3,6-Tri-O-acetyl-4-O-(2,3,4,6-tetra-O-acetyl-β-D-galactopyranosyl)-β-D-glucopyranosyl]-N-(3-carboxyphenyl)-2-methylpropanamide (**2s**)*

Following General procedure B, using isobutyric anhydride (0.046 mL, 0.275 mmol) instead of Ac_2O and extended reaction time of 18 h, lactosylamine **S1** (0.189 g, 0.25 mmol) afforded product **2s** (0.181 g, 88%) as a white foam. $[\alpha]_D^{20}$ = +27.9 (*c* 0.2 in $CHCl_3$); **^{1}H NMR** (500 MHz, DMSO-d_6; T = 100 °C) δ 0.94 (d, $J_{CH3,CH}$ = 6.7 Hz, 3H, CH(C**H**$_3$)$_{a,b}$), 1.02 (d, $J_{CH3,CH}$ = 6.6 Hz, 3H, CH(C**H**$_3$)$_{a,b}$), 1.89, 1.90, 1.96, 1.96, 2.00, 2.05, 2.08 (7 × s, 21H CH_3CO-2,3,2',3',4',6'), 2.51 – 2.52 (m, 1H, C**H**(CH_3)$_{a,b}$), 3.67 (t, $J_{4,5}$ = $J_{4,3}$ = 9.8, $J_{4,3}$ = 9.2 Hz, 1H, H-4), 3.96 (ddd, $J_{5,4}$ = 9.8, $J_{5,6b}$ = 5.2, $J_{5,6a}$ = 2.2 Hz, 1H, H-5), 3.99 (dd, J_{gem} = 11.2, $J_{6'a,5'}$ = 6.5 Hz, 1H, H-6'a), 4.02 (dd, J_{gem} = 11.2, $J_{6'b,5'}$ = 6.5 Hz Hz, 1H, H-6'b), 4.08 (dd, J_{gem} = 12.0, $J_{6a,5}$ = 5.2 Hz, 1H, H-6a), 4.15 (td, $J_{5',6'a}$ = $J_{5',6'b}$ = 6.5, $J_{5',4'}$ = 1.3 Hz, 1H, H-5'), 4.39 – 4.45 (m, 1H, H-2), 4.43 (dd, J_{gem} = 12.0, $J_{6b,5}$ = 2.2 Hz, 1H, H-6b), 4.72 (d, $J_{1',2'}$ = 7.9 Hz, 1H, H-1'), 4.86 (dd, $J_{2',3'}$ = 10.3, $J_{2',1'}$ = 7.9 Hz, 1H, H-2'), 5.12 (dd, $J_{3',2'}$ = 10.3, $J_{3',4'}$ = 3.6 Hz, 1H, H-3'), 5.22 (t, $J_{3,2}$ = $J_{3,4}$ = 9.2 Hz, 1H, H-3), 5.24 (dd, $J_{4',3'}$ = 3.6, $J_{4',5'}$ = 1.3 Hz, 1H, H-4'), 5.98 (d, $J_{1,2}$ = 9.5 Hz, 1H ,H-1), 7.33 – 7.38 (ddd, $J_{6'',5''}$ = 7.8, $J_{6'',2''}$ = 2.2, $J_{6'',4''}$ =1.3 Hz, 1H, H-6''), 7.53 (t, $J_{5'',4''}$ = $J_{5'',6''}$ = 7.8 Hz, 1H, H-5''), 7.70 – 7.72 (m, 1H, H-2''), 7.95 (dt, $J_{4'',5''}$ = 7 .8, $J_{4'',2''}$ = 1.3 Hz, 1H, H-4''); **^{13}C NMR** (126 MHz, DMSO-d_6; T = 100 °C) δ 18.67 (CH(**C**H_3)$_{a,b}$), 18.70 (CH(**C**H_3)$_{a,b}$), 19.59, 19.64, 19.68, 19.70, 19.70, 19.74, 19.81 (**C**H_3CO-2,3,6,2',3',4',6'), 30.60 (**C**H(CH_3)$_{a,b}$), 60.52 (CH_2-6'), 61.33 (CH_2-6), 66.94 (CH-4'), 68.61 (CH-2), 68.91 (CH-2'), 69.59 (CH-5'), 70.14 (CH-3'), 73.32 (CH-3), 73.50 (CH-5), 75.06 (CH-4), 80.24 (CH-1), 99.29 (CH-1'), 128.63 (CH-5''), 128.68 (CH-4''), 130.09 (CH-2''), 131.92 (C-3''), 133.31 (CH-6''), 136.99 (C-1''), 165.93 (**C**OOH), 168.05, 168.34, 168.68, 168.71, 169.11, 169.14, 169.40 (CH_3**C**O-2,3,6,2',3',4',6'), 176.87 (**C**CH(CH_3)$_{a,b}$); **IR** ($CHCl_3$) 3516, 2980, 2936, 1754, 1710, 1682, 1604, 1587, 1489, 1451, 1412, 1387, 1369, 1297,

1235, 1049, 979, 841, 603, 556, 495 cm^{-1}; **HRMS** (ESI) $[M-H]^-$ *m/z* calcd for $C_{37}H_{46}O_{20}N$: 824.2619, found: 824.2612.

*N-[2,3,6-Tri-O-acetyl-4-O-(2,3,4,6-tetra-O-acetyl-β-D-galactopyranosyl)-β-D-glucopyranosyl]-N-(3-carboxyphenyl)benzamide (**2t**)*

Following General procedure B, using benzoic anhydride (0.062 g, 0.275 mmol) instead of Ac_2O, an extended reaction time of 18 h, and heating at 45 °C lactosylamine **S1** (0.189 g, 0.25 mmol) afforded product **2t** (0.150 g, 70%) as a white foam. $[\alpha]_D^{20}$ = +59.5 (*c* 0.3 in $CHCl_3$); **^{1}H NMR** (500 MHz, DMSO-d_6; T = 100 °C) δ 1.88 (s, 3H, CH_3CO), 1.90 (s, 3H, CH_3CO), 1.96 (s, 3H, CH_3CO), 1.97 (s, 3H, CH_3CO), 2.00 (s, 3H, CH_3CO), 2.08 (s, 3H, CH_3CO), 2.09 (s, 3H, CH_3CO), 2.12 (s, 3H, CH_3CO), 3.74 (t, $J_{4,5} = J_{4,3}$ = 9.5 Hz, 1H, H-4), 3.88 (ddd, $J_{5,4}$ = 9.6, $J_{5,6a}$ = 5.1, $J_{5,6b}$ = 1.7 Hz, 1H, H-5), 3.99 (dd, J_{gem} = 11.1, $J_{6'a,5'}$ = 6.8 Hz, 1H, H-6'a), 4.02 (dd, J_{gem} = 11.1, $J_{6'b,5'}$ = 6.2 Hz, 1H, H-6'b), 4.13 (dd, J_{gem} = 12.0, $J_{6a,5}$ = 5.3 Hz, 1H, H-6a), 4.16 (td, $J_{5',6'a} = J_{5',6'b}$ = 6.5, $J_{5',4'}$ = 1.0 Hz, 2H), 4.49 (dd, J_{gem} = 12.1, $J_{6b,5}$ = 2.1 Hz, 1H, H-6b), 4.54 (t, $J_{2,3} = J_{2,1}$ = 9.4 Hz, 1H, H-2), 4.75 (d, $J_{1',2'}$ = 7.9 Hz, 1H, H-1'), 4.86 (dd, $J_{2',3'}$ = 10.1, $J_{2',1'}$ = 8.0 Hz, 1H, H-2'), 5.12 (dd, $J_{3',2'}$ = 10.3, $J_{3',4'}$ = 3.6 Hz, 1H, H-3'), 5.20 (t, $J_{3,4} = J_{3,2}$ = 9.1 Hz, 1H, H-3), 5.24 (dd, $J_{4',3'}$ = 3.6, $J_{4',5'}$ = 1.1 Hz, 1H, H-4'), 5.91 (d, $J_{1,2}$ = 9.6 Hz, 1H, H-1), 7.29 – 7.37 (m, 1H, H-6''), 7.39 (t, $J_{5'',6''} = J_{5'',4''}$ = 7.9 Hz, 1H, H-5''), 7.49 (t, $J_{3''',4'''} = J_{3''',2'''}$ = 7.7 Hz, 2H, H-3'''), 7.60 (tt, $J_{4''',3'''}$ = 7.8, $J_{4''',2'''}$ = 1.2 Hz, 1H, H-4'''), 7.70 (t, $J_{2'',6''} = J_{2'',4''}$ = 1.8 Hz, 1H, H-2''), 7.81 (dd, $J_{4'',5''}$ = 7.7, $J_{4'',2''}$ = 1.4 Hz, 1H, H-4''), 7.95 (dd, $J_{2''',3'''}$ = 8.2, $J_{2''',4'''}$ = 1.3 Hz, 2H, H-2'''); **^{13}C NMR** (126 MHz, DMSO-d_6; T = 100 °C) δ 19.61, 19.65, 19.68, 19.72, 19.72, 19.75, 19.91 (7 × **C**H_3CO-2,3,6,2',3',4',6'), 60.51 (CH_2-6'), 61.47 (CH_2-6), 66.94 (CH-4'), 68.44 (CH-2), 68.91 (CH-2'), 69.60 (CH-5'), 70.16 (CH-3'), 73.23, 73.73, 74.99, 81.89, 99.29, 127.03, 127.60, 128.71, 129.49, 129.90, 131.62, 132.06, 133.08, 134.96, 137.38, 165.88, 168.00, 168.37, 168.69, 168.72, 169.12, 169.15, 169.52 (7 × CH_3**C**O-2,3,6,2',3',4',6'), 170.34 (**C**ON); **IR** ($CHCl_3$) 3521, 3105, 2985, 1754, 1698, 1671, 1604, 1586, 1492, 1452, 1415, 1369, 1289, 1289, 1231, 1173, 1077, 1050, 1027, 913, 879, 841, 714, 702, 602 cm^{-1}; **HRMS** (ESI) $[M+Na]^+$ *m/z* calcd for $C_{40}H_{45}O_{20}NNa$: 882.2427, found: 882.2422.

*N-[2,3,6-Tri-O-acetyl-4-O-(2,3,4,6-tetra-O-acetyl-β-D-galactopyranosyl)-β-D-glucopyranosyl]-N-(3-carboxyphenyl)-2-phenylacetamide (**2u**)*

Following General procedure B, using 2-phenylacetic anhydride (0.070 g, 0.275 mmol) instead of Ac_2O, extended reaction time of 18 h, and heating at 45 °C, lactosylamine **S1** (0.189 g, 0.25 mmol) gave product **2u** (0.214 g, 98%) as a white foam. $[\alpha]_D^{20}$ = +25.1 (*c* 0.2 in $CHCl_3$); **^{1}H NMR** (500 MHz, DMSO-d_6; T = 100 °C) δ 1.88, 1.89, 1.93, 1.95, 2.00, 2.05, 2.08 (7 × s, 21H CH_3CO-2,3,2',3',4',6'), 3.52 (bs, 2H, PhC**H_2**CON), 3.68 (dd, $J_{4,5}$ = 9.8 $J_{4,3}$ = 9.2 Hz, 1H, H-4), 3.95 (ddd, $J_{5,4}$ = 9.8, $J_{5,6b}$ = 5.2, $J_{5,6a}$ = 2.2 Hz, 1H, H-5), 3.99 (dd, J_{gem} = 11.2, $J_{6'a,5'}$ = 6.5 Hz, 1H, H-6'a), 4.02 (dd, J_{gem} = 11.2, $J_{6'b,5'}$ = 6.5 Hz Hz, 1H, H-6'b), 4.07 (dd, J_{gem} = 12.0, $J_{6a,5}$ = 5.2 Hz, 1H, H-6a), 4.14 (td, $J_{5',6'a} = J_{5',6'b}$ = 6.5, $J_{5',4'}$ = 1.3 Hz, 1H, H-5'), 4.38 – 4.43 (m, 1H, H-2), 4.42 (dd, J_{gem} = 12.0, $J_{6b,5}$ = 2.2 Hz, 1H, H-6b), 4.72 (d, $J_{1',2'}$ = 7.9 Hz, 1H, H-1'), 4.86 (dd, $J_{2',3'}$ = 10.3, $J_{2',1'}$ = 7.9 Hz, 1H, H-2'), 5.11 (dd,

$J_{3',2'}$ = 10.3, $J_{3',4'}$ = 3.6 Hz, 1H, H-3'), 5.21 (t, $J_{3,2}$ = $J_{3,4}$ = 9.2 Hz, 1H, H-3), 5.23 (dd, $J_{4',3'}$ = 3.6, $J_{4',5'}$ = 1.3 Hz, 1H, H-4'), 6.03 (d, $J_{1,2}$ = 9.6 Hz, 1H, H-1), 7.05 (d, J_{vic} = 7.4 Hz, 2H, *o*-PhCH$_2$CON), 7.17 – 7.22 (m, 1H, *p*-PhCH$_2$CON), 7.22 – 7.26 (m, 2H, *m*-PhCH$_2$CON), 7.29 (ddd, $J_{6'',5''}$ = 7.7, $J_{6'',2''}$ = 2.2, $J_{6'',4''}$ =1.4 Hz, 1H, H-6''), 7.48 (td, $J_{5'',4''}$ = $J_{5'',6''}$ = 7.7, $J_{5'',2''}$ = 0.5 Hz, 1H, H-5''), 7.67 (ddd, $J_{2'',6''}$ = 2.2, $J_{2'',4''}$ = 1.4, $J_{2'',5''}$ = 0.5 Hz, 1H, H-2''), 7.94 (dt, $J_{4'',5''}$ = 7.7, $J_{4'',2''}$ = 1.4 Hz, 1H, H-4''); **^{13}C NMR** (126 MHz, DMSO-*d*$_6$; T = 100 °C) δ 19.60, 19.65, 19.70, 19.74, 19.84 (**C**H$_3$CO-2,3,6,2',3',4',6'), 40.39 (Ph**C**H$_2$CON), 60.52 (CH$_2$-6'), 61.31 (CH$_2$-6), 66.94 (CH-4'), 68.51 (CH-2), 68.92 (CH-2'), 69.60 (CH-5'), 70.15 (CH-3'), 73.37 (CH-3), 73.54 (CH-5), 75.01 (CH-4), 80.45 (CH-1), 99.30 (CH-1'), 125.99 (*p*-PhCH$_2$CON), 127.60 (*m*-PhCH$_2$CON), 128.43 (*o*-PhCH$_2$CON), 128.53 (CH-5''), 128.77 (CH-4''), 130.33 (CH-2''), 132.35 (C-3''), 133.30 (CH-6''), 134.38 (*ipso*-PhCH$_2$CON), 136.70 (C-1''), 165.92 (**C**OOH), 168.02, 168.36, 168.70, 168.72, 169.13, 169.15, 169.44 (CH$_3$**C**O-2,3,6,2',3',4',6'), 170.84 (PhCH$_2$**C**ON); **IR** (CHCl$_3$) 3514, 3088, 3064, 2979, 1754, 1700, 1682, 1603, 1587, 1496, 1489, 1454, 1411, 1370, 1332, 1296, 1296, 1231, 1170, 1138, 1076, 1050, 983, 913, 842, 698, 620, 601 cm^{-1}; **HRMS** (ESI) [M+Na]$^+$ *m/z* calcd for $C_{41}H_{47}O_{20}NNa$: 896.2584, found: 896.2570.

*N-[2,3,6-Tri-O-acetyl-4-O-(2,3,4,6-tetra-O-acetyl-β-D-galactopyranosyl)-β-D-glucopyranosyl]-N-[3-(methylcarboxy)phenyl]acetamide (**2v**)*

Following General procedure A, compound **1** (0.636 g, 1.0 mmol) was reacted with methyl 3-aminobenzoic acid (0.227 g, 1.5 mmol) for 1 day. The crude product was then subjected to General procedure B, affording product **2v** (0.440 g, 54%) as a white foam. $[\alpha]_D^{20}$ = +32.9 (*c* 0.4 in CHCl$_3$); **^{1}H NMR** (500 MHz, DMSO-*d*$_6$; T = 100 °C) δ 1.88 (s, 3H, CH$_3$CO), 1.90 (s, 3H, CH$_3$CO), 1.91 (bs, 3H, CH$_3$CON), 1.96 (s, 3H, CH$_3$CO), 1.97 (s, 3H, CH$_3$CO), 2.00 (s, 3H, CH$_3$CO), 2.08 (s, 3H, CH$_3$CO), 2.08 (s, 3H, CH$_3$CO), 3.70 (dd, $J_{4,5}$ = 9.7, $J_{4,3}$ = 9.2 Hz, 1H, H-4), 3.89 (s, 3H, OCH$_3$), 3.96 (ddd, $J_{5,4}$ = 9.9, $J_{5,6a}$ = 5.2, $J_{5,6b}$ = 2.1 Hz, 1H, H-5), 4.00 (dd, J_{gem} = 11.2, $J_{6'a,5'}$ = 6.9 Hz, 1H, H-6'a), 4.03 (dd, J_{gem} = 11.2, $J_{6'b,5'}$ = 6.0 Hz, 1H, H-6'b), 4.09 (dd, J_{gem} =12.1, $J_{6a,5}$ = 5.2 Hz, 1H, H-6a), 4.15 (td, $J_{5',6a'}$ = $J_{5',6b'}$ = 6.5, $J_{5',4'}$ = 1.3 Hz, 1H, H-5'), 4.40 (t, $J_{2,3}$ = $J_{2,1}$ = 9.4 Hz, 1H, H-2), 4.46 (dd, J_{gem} = 12.1, $J_{6b,5}$ = 2.2 Hz, 1H, H-6b), 4.73 (d, $J_{1',2'}$ = 7.9 Hz, 1H, H-1'), 4.87 (dd, $J_{1',2'}$ = 10.3, $J_{2',1'}$ = 7.9 Hz, 1H, H-2'), 5.12 (dd, $J_{3',2'}$ = 10.2, $J_{3',4'}$ = 3.6 Hz, 1H, H-3'), 5.21 (t, $J_{3,4}$ = $J_{3,2}$ = 9.3 Hz, 1H, H-3), 5.25 (dd, $J_{4',3'}$ = 3.6, $J_{4',5'}$ = 1.2 Hz, 1H, H-4'), 5.96 (d, $J_{1,2}$ = 9.6 Hz, 1H, H-1), 7.41 (ddd, $J_{6'',5''}$ = 7.9, $J_{6'',2''}$ = 2.2, $J_{6'',4''}$ = 1.2 Hz, 1H, H-6''), 7.55 (t, $J_{5'',6''}$ = $J_{5'',4''}$ = 7.8 Hz, 1H, H-5''), 7.73 (t, $J_{2'',6''}$ = $J_{2'',4''}$ = 1.9 Hz, 1H, H-2''), 7.96 (dt, $J_{4'',5''}$ = 7.8, $J_{4'',6''}$ = $J_{4'',2''}$ = 1.4 Hz, 1H, H-4''); **^{13}C NMR** (101 MHz, DMSO-*d*$_6$; T = 100 °C) δ 19.45, 19.49, 19.56, 19.57, 19.58, 19.61, 19.70 (7 × **C**H$_3$CO-2,3,6,2',3',4',6'), 22.05 (**C**H$_3$CON), 51.46 (OCH$_3$), 60.48 (CH$_2$-6'), 61.33 (CH$_2$-6), 66.92 (CH-4'), 68.46 (CH-2), 68.91 (CH-2'), 69.59 (CH-5'), 70.11 (CH-3'), 73.27 (CH-3), 73.58 (CH-5), 74.85 (CH-4), 80.50 (CH-1), 99.17 (CH-1'), 128.36 (CH-4''), 128.81 (CH-5''), 129.86 (CH-2''), 130.44 (C-3''), 133.73 (CH-6''), 137.44 (C-1''), 164.98 (**C**OOCH$_3$), 167.95, 168.20, 168.53, 168.56, 168.98, 168.99, 169.27 (7 × CH$_3$**C**O-2,3,6,2',3',4',6'), 169.96 (CH$_3$**C**ON); **IR** (CHCl$_3$) 3028, 2955, 1754, 1725, 1604, 1587, 1488, 1448, 1438, 1371, 1299, 1232, 1171, 1077, 1055, 1004, 913, 707, 599, 571, 550 cm^{-1}; **HRMS** (ESI) [M+Na]$^+$ *m/z* calcd for $C_{36}H_{45}O_{20}NNa$: 834.2427, found: 834.2426.

N-[2,3,6-Tri-*O*-acetyl-4-*O*-(2,3,4,6-tetra-*O*-acetyl-β-D-galactopyranosyl)-β-D-glucopyranosyl]-*N*-[3-(acetoxymethyl)phenyl)]acetamide (**2x**)

Following General procedure A, compound **1** (0.636 g, 1.0 mmol) was reacted with 4-(hydroxymethyl)aniline (0.209 mL, 1.7 mmol) for 3 days. The crude product was then subjected to General procedure B, affording product **2x** (0.552 g, 67%) as a white foam. $[\alpha]_D^{20}$ = +32.0 (*c* 0.5 in $CHCl_3$); **^{1}H NMR** (500 MHz, DMSO-d_6; T = 100 °C) δ 1.86 (s, 3H, CH_3CON), 1.89 (s, 3H, CH_3CO), 1.90 (s, 3H, CH_3CO), 1.96 (s, 3H, CH_3CO), 1.97 (s, 3H, CH_3CO), 2.00 (s, 3H, CH_3CO), 2.07 (s, 3H, CH_3CO), 2.08 (s, 3H, CH_3CO), 2.09 (s, 3H, CH_3CO), 3.67 (t, $J_{4,3} = J_{4,5}$ = 9.5 Hz, 1H, H-4), 3.93 (ddd, $J_{5,4}$ = 9.8, $J_{5,6a}$ = 5.3, $J_{5,6b}$ = 2.1 Hz, 1H, H-5), 4.00 (dd, J_{gem} = 11.2, $J_{6'a,5'}$ = 6.8 Hz, 1H, H-6'a), 4.02 (dd, J_{gem} = 11.2, $J_{6'b,5'}$ = 6.1 Hz, 1H, H-6'b), 4.09 (dd, J_{gem} = 12.1, $J_{6a,5}$ = 5.3 Hz, 1H, H-6a), 4.15 (ddd, $J_{5',6'a}$ = 6.8, $J_{5',6'b}$ = 6.0, $J_{5',4'}$ = 1.3 Hz, 1H, H-5'), 4.42 (t, $J_{2,1} = J_{2,3}$ = 9.4 Hz, 1H, H-2), 4.46 (dd, J_{gem} = 12.1, $J_{6b,5}$ = 2.2 Hz, 1H, H-6b), 4.73 (d, $J_{1',2'}$ = 7.9 Hz, 1H, H-1'), 4.87 (dd, $J_{2',3'}$ = 10.3, $J_{2',1'}$ = 7.9 Hz, 1H, H-2'), 5.09 (s, 2H, CH_2OAc), 5.12 (dd, $J_{3',2'}$ = 10.3, $J_{3',4'}$ = 3.6 Hz, 1H, H-3'), 5.20 (t, $J_{3,4} = J_{3,2}$ = 9.1 Hz, 1H, H-3), 5.24 (dd, $J_{4',3'}$ = 3.6, $J_{4',5'}$ = 1.2 Hz, 1H, H-4'), 5.95 (d, $J_{1,2}$ = 9.6 Hz, 1H, H-1), 7.11 (dt, $J_{6'',5''}$ = 7.3, $J_{6'',2''} = J_{6'',4''}$ = 2.0 Hz, 1H, H-6''), 7.13 (td, $J_{2'',4''} = J_{2'',6''}$ = 1.9, $J_{2'',5''}$ = 0.7 Hz, 1H, H-2''), 7.38 (dt, $J_{4'',5''}$ = 7.7, $J_{4'',2''} = J_{4'',6''}$ = 1.6 Hz, 1H, H-4''), 7.41 (td, $J_{5'',4''} = J_{5'',6''}$ = 7.6, $J_{5'',2''}$ = 0.7 Hz, 1H, H-5''); **^{13}C NMR** (126 MHz, DMSO-d_6; T = 100 °C) δ 19.51, 19.56, 19.62, 19.64, 19.69, 19.73, 19.82, 19.92 (8 × **C**H_3CO-2,3,6,2',3',4',6'; **C**H_3COOCH_2-3''), 22.20 (**C**H_3CON), 60.50 (CH_2-6'), 61.45 (CH_2-6), 64.19 (CH_3COO**C**H_2-3''), 66.94 (CH-4'), 68.54 (CH-2), 68.93 (CH-2'), 69.60 (CH-5'), 70.13 (CH-3'), 73.43 (CH-3), 73.64 (CH-5), 74.97 (CH-4), 80.20 (CH-1), 99.22 (CH-1'), 127.09, 128.47, 128.51, 128.55 (4 × CH-2'',4'',5'',6''), 136.97 (C-3''), 137.23 (C-1''), 167.99, 168.27, 168.62, 168.63, 169.06, 169.06, 169.34, 169.41, (8 × CH_3**C**O-2,3,6,2',3',4',6'; CH_3**C**$OOCH_2$-3''), 170.02 (CH_3**C**ON); **IR** ($CHCl_3$) 1753, 1680, 1608, 1591, 1489, 1371, 1230, 1172, 1054, 706, 601 cm^{-1}; **HRMS** (ESI) $[M+Na]^+$ *m/z* calcd for $C_{37}H_{47}O_{20}NNa$: 848.2584, found: 848.2585.

*3-(1H-Tetrazol-5-yl)aniline (**S2**)*

The compound **S2** was prepared according to a previously published procedure.[8] 3-Aminobenzonitrile (5.9 g, 50 mmol), sodium azide (4.2 g, 65 mmol), and triethylammonium chloride (14 g, 100 mmol) were dissolved in toluene (100 mL) and refluxed overnight. The reaction mixture was then cooled to room temperature and extracted with H_2O (2 × 150 mL). The combined aqueous layers were adjusted to pH 4.5 and left to stand overnight. The resulting precipitate was collected by filtration, washed with cold H_2O, and dried under vacuum, affording product **S2** (5.3 g, 66%) as white crystals. NMR data were consistent with those reported previously.[8] **M.p.**: 200-200.5 °C (lit. 201.5-202 °C); **IR** (CH_3OH) 3365, 3218, 1900, 1613, 1592, 1566, 1531, 1496, 1472, 1317, 1274, 1158, 1063, 946, 862, 741, 533, 446 cm^{-1}; **HRMS** (ESI) $[M+Na]^+$ *m/z* calcd for $C_7H_7N_5Na$: 184.0594, found: 184.0593; $[M+H]^+$ *m/z* calcd for $C_7H_8N_5$: 162.0774, found: 162.0774.

*N-[2,3,6-Tri-O-acetyl-4-O-(2,3,4,6-tetra-O-acetyl-β-D-galactopyranosyl)-β-D-glucopyranosyl]-N-[3-(1H-tetrazol-5-yl)phenyl)]acetamide (**2y**)*

Following General procedure A, compound **1** (0.636 g, 1.0 mmol) was reacted with 3-(1*H*-tetrazol-5-yl)aniline **S2** (0.273 g, 1.7 mmol) for 3 days. The crude product was then subjected to General procedure B, affording product **2y** (0.603 g, 73%) as a white foam. $[\boldsymbol{\alpha}]_{\mathbf{D}}^{\mathbf{20}}$ = +9.2 (*c* 0.6 in $CHCl_3$); **^{1}H NMR** (500 MHz, DMSO-d_6; T = 100 °C) δ 1.87 (s, 3H, CH_3CO), 1.90 (s, 3H, CH_3CO), 1.93 (s, 3H, CH_3CO), 1.95 – 1.98 (m, 6H, CH_3CO, CH_3CON), 2.00 (s, 3H, CH_3CO), 2.04 (s, 3H, CH_3CO), 2.08 (s, 3H, CH_3CO), 3.71 (dd, $J_{4,5}$ = 9.8, $J_{4,3}$ = 9.1 Hz, 1H, H-4), 3.97 (ddd, $J_{5,4}$ = 9.9, $J_{5,6a}$ = 5.0, $J_{5,6b}$ = 2.3 Hz, 1H, H-5), 3.99 (dd, J_{gem} = 11.2, $J_{6'a,5'}$ = 6.8 Hz, 1H, H-6'a), 4.01 (dd, J_{gem} = 11.2, $J_{6'b,5'}$ = 6.1 Hz, 1H, H-6'b), 4.09 (dd, J_{gem} = 12.0, $J_{6a,5}$ = 5.1 Hz, 1H, H-6a), 4.14 (ddd, $J_{5',6'a}$ = 7.4, $J_{5',6'b}$ = 6.1, $J_{5',4'}$ = 1.3 Hz, 1H, H-5'), 4.47 (dd, J_{gem} = 12.1, $J_{6b,5}$ = 2.2 Hz, 1H, H-6b), 4.47 (dd, $J_{2,1}$ = 9.6, $J_{2,3}$ = 9.0 Hz, 1H, H-2), 4.72 (d, $J_{1',2'}$ = 7.9 Hz, 1H, H-1'), 4.86 (dd, $J_{2',3'}$ = 10.2, $J_{2',1'}$ = 7.9 Hz, 1H, H-2'), 5.11 (dd, $J_{3',2'}$ = 10.2, $J_{3',4'}$ = 3.6 Hz, 1H, H-3'), 5.23 (t, $J_{3,4}$ = $J_{3,2}$ = 9.1 Hz, 1H, H-3), 5.24 (dd, $J_{4',3'}$ = 3.6, $J_{4',5'}$ = 1.2 Hz, 1H, H-4'), 5.98 (d, $J_{1,2}$ = 9.6 Hz, 1H, H-1), 7.34 (ddd, $J_{6'',5''}$ = 8.0, $J_{6'',2''}$ = 2.1, $J_{6'',4''}$ = 1.1 Hz, 1H, H-6''), 7.61 (td, $J_{5'',6''}$ = $J_{5'',4''}$ = 7.9, $J_{5'',2''}$ = 0.6 Hz, 1H, H-5''), 7.86 (ddd, $J_{2'',6''}$ = 2.1, $J_{2'',4''}$ = 1.7, $J_{2'',5''}$ = 0.6 Hz, 1H, H-2''), 8.04 (ddd, $J_{4'',5''}$ = 7.9, $J_{4'',2''}$ = 1.7, $J_{4'',6''}$ = 1.2 Hz, 1H, H-4''); **^{13}C NMR** (126 MHz, DMSO-d_6; T = 100 °C) δ 19.49, 19.54, 19.59, 19.61, 19.64, 19.68, 19.75 (7 × **C**H_3CO-2,3,6,2',3',4',6'), 22.16 (**C**H_3CON), 60.48 (CH_2-6'), 61.38 (CH_2-6), 66.92 (CH-4'), 68.47 (CH-2), 68.91 (CH-2'), 69.59 (CH-5'), 70.12 (CH-3'), 73.33 (CH-3), 73.60 (CH-5), 74.87 (CH-4), 80.57 (CH-1), 99.20 (CH-1'), 125.70 (C-3''), 126.22 (CH-4''), 127.72 (CH-2''), 129.44 (CH-5''), 131.31 (CH-6''), 138.01 (C-1''), 155.72 (C-Tz), 168.01, 168.26, 168.59, 168.61, 169.02, 169.04, 169.39 (7 × CH_3**C**O-2,3,6,2',3',4',6'), 170.06 (CH_3**C**ON); **IR** ($CHCl_3$) 3216, 1754, 1688, 1602, 1588, 1555, 1486, 1371, 1234, 1171, 1056, 705, 601 cm^{-1}; **HRMS** (ESI) $[M+Na]^+$ *m/z* calcd for $C_{35}H_{43}O_{18}N_5Na$: 844.2501, found: 844.2497.

*3-(5-Methylfuran-2-yl)aniline (**S3**)*

The compound **S3** was prepared according to a previously published procedure.[9] A pressure tube was loaded with 3-iodoaniline (1.1 g, 5 mmol), 2-methylfuran (0.9 mL; 10 mmol), KOAc (1.96 g, 20 mmol), palladium(II) acetate (0.011 g, 0.05 mmol), and dimethylacetamide (20 mL). The reaction mixture was heated at 135 °C for 18 h, then cooled to room temperature, filtered, and washed with EtOAc. The filtrate was evaporated in vacuo, redissolved in EtOAc (50 mL), and washed with H_2O (2 × 20 mL) and brine (20 mL). The organic phase was separated, dried over anhydrous $MgSO_4$, filtered, and concentrated in vacuo. The residue was purified by liquid column chromatography on silica gel (10% → 60% EtOAc in CyH) to give product **S3** (0.160 g, 18%) as an orange oil. **^{1}H NMR** (400 MHz, $CDCl_3$) δ 2.36 (d, J = 1.2 Hz, 3H, CH_3), 3.79 (s, 2H. NH_2), 6.04 (dq, $J_{4',3'}$ = 3.2, $J_{4',CH3}$ = 1.2 Hz, 1H, H-4'), 6.50 (d, $J_{3',4'}$ = 3.2 Hz, 1H, H-3'), 6.60 (ddd, $J_{6,5}$ = 7.8, $J_{6,2}$ = 2.3, $J_{6,4}$ = 1.0 Hz, 1H, H-6), 7.02 (ddd, $J_{2,6}$ = 2.3, $J_{2,4}$ = 1.6, $J_{2,5}$ = 0.6 Hz, 1H, H-2), 7.07 (ddd, $J_{4,5}$ = 7.8, $J_{4,2}$ = 1.7, $J_{4,6}$ = 1.0 Hz, 1H, H-4), 7.16 (td, $J_{5,6}$ = $J_{5,4}$ = 7.8, $J_{5,2}$ = 0.6 Hz, 1H, H-5); **^{13}C NMR** (101 MHz, $CDCl_3$) δ 13.87 (CH_3), 106.08 (CH-3'), 107.75 (CH-4'), 110.37 (CH-2), 114.24 (CH-6), 114.63 (CH-4), 129.72 (CH-5), 132.28 (C-3), 145.86 (C-1), 151.93 (C-5'), 152.38 (C-2'); **IR** ($CHCl_3$) 3487,

3455, 3399, 3377, 3109, 3050, 2954, 2883, 1620, 1588, 1492, 1482, 1462, 1462, 1447, 1317, 1306, 1289, 1166, 1068, 995, 854, 690, 447 cm^{-1}; **HRMS** (ESI) $[M+H]^+$ *m/z* calcd for $C_{11}H_{12}ON$: 174.0913, found: 174.0915.

*N-[2,3,6-Tri-O-acetyl-4-O-(2,3,4,6-tetra-O-acetyl-β-D-galactopyranosyl)-β-D-glucopyranosyl]-N-[3-(5-methylfuran-2-yl)phenyl)]acetamide (**2z**)*

Following General procedure A, compound **1** (0.636 g, 1.0 mmol) was reacted with 3-(5-methylfuran-2-yl)aniline **S3** (0.209 mL, 1.7 mmol) for 3 days. The crude product was then subjected to General procedure B, affording product **2z** (0.552 g, 67%) as a white foam. $[\alpha]_D^{20}$ = +22.9 (*c* 0.7 in $CHCl_3$); **^{1}H NMR** (500 MHz, DMSO-d_6; T = 100 °C) δ 1.88 (s, 3H, CH_3CO), 1.90 (s, 6H, CH_3CO, CH_3CON), 1.94 (s, 3H, CH_3CO), 1.99 (s, 3H, CH_3CO), 2.00 (s, 3H, CH_3CO), 2.03 (s, 3H, CH_3CO), 2.08 (s, 3H, CH_3CO), 2.35 (d, $J_{CH3,4'''}$ = 1.2 Hz, 3H, CH_3-furan), 3.69 (t, $J_{4,3}$ = $J_{4,5}$ = 9.5 Hz, 1H, H-4), 3.94 (ddd, $J_{5,4}$ = 9.9, $J_{5,6a}$ = 5.2, $J_{5,6b}$ = 2.2 Hz, 1H, H-5), 3.99 (dd, J_{gem} = 11.3, $J_{6'a,5'}$ = 6.8 Hz, 1H, H-6'a), 4.02 (dd, J_{gem} = 11.1, $J_{6'b,5'}$ = 6.1 Hz, 1H, H-6'b), 4.11 (dd, J_{gem} = 12.2, $J_{6a,5}$ = 5.3 Hz, 1H, H-6a), 4.13 – 4.17 (m, 1H, H-5'), 4.43 – 4.50 (m, 1H, H-6b), 4.43 – 4.52 (m, 1H, H-2), 4.73 (d, $J_{1',2'}$ = 7.9 Hz, 1H, H-1'), 4.87 (dd, $J_{2',3'}$ = 10.3, $J_{2',1'}$ = 7.9 Hz, 1H, H-2'), 5.11 (dd, $J_{3',2'}$ = 10.3, $J_{3',4'}$ = 3.6 Hz, 1H, H-3'), 5.21 (t, $J_{3,4}$ = $J_{3,2}$ = 9.1 Hz, 1H, H-3), 5.24 (dd, $J_{4',3'}$ = 3.6, $J_{4',5'}$ = 1.3 Hz, 1H, H-4'), 5.98 (d, $J_{1,2}$ = 9.6 Hz, 1H, H-1), 6.19 (dq, $J_{4,3}$ = 3.2, $J_{4,CH3}$ = 1.0 Hz, 1H, H-4-furan), 6.76 (d, $J_{3,4}$ = 3.2 Hz, 2H, H-3-furan), 7.01 (ddd, $J_{6'',5''}$ = 7.9, $J_{6'',2''}$ = 2.1, $J_{6'',4''}$ = 1.0 Hz, 1H, H-6''), 7.38 (t, $J_{2'',6''}$ = $J_{2'',4''}$ = 1.9 Hz, 1H, H-2''), 7.41 (t, $J_{5'',6''}$ = $J_{5'',4''}$ = 7.9 Hz, 1H, H-5''), 7.63 (ddd, $J_{4'',5''}$ = 7.9, $J_{4'',6''}$ = 1.8, $J_{4'',2''}$ = 1.1 Hz, 1H, H-4''); **^{13}C NMR** (126 MHz, DMSO-d_6; T = 100 °C) δ 12.57 (CH_3), 19.48, 19.53, 19.58, 19.59, 19.66, 19.70, 19.72 (7 × **C**H_3CO-2,3,6,2',3',4',6'), 22.15 (**C**H_3CON), 60.48 (CH_2-6'), 61.40 (CH_2-6), 66.92 (CH-4'), 68.56 (CH-2), 68.90 (CH-2'), 69.58 (CH-5'), 70.12 (CH-3'), 73.43 (CH-3), 73.58 (CH-5), 74.93 (CH-4), 79.84 (CH-1), 99.19 (CH-1'), 106.85 (CH-3-furan), 107.56 (CH-4-furan), 122.41 (CH-4''), 123.95 (CH-2''), 127.42 (CH-6''), 128.87 (CH-5''), 131.20 (C-3''), 137.62 (C-1''), 150.16 (C-2-furan), 151.66 (C-5-furan), 168.00, 168.24, 168.60, 169.01, 169.03, 169.28 (7 × CH_3**C**O-2,3,6,2',3',4',6'), 169.99 (CH_3**C**ON); **IR** ($CHCl_3$) 2876, 1754, 1676, 1613, 1597, 1548, 1487, 1475, 1371, 1232, 1172, 1075, 1055, 1025, 1000, 956, 602 cm^{-1}; **HRMS** (ESI) $[M+Na]^+$ *m/z* calcd for $C_{39}H_{47}O_{19}NNa$: 856.2635, found: 856.2638; $[M+H]^+$ *m/z* calcd for $C_{39}H_{48}O_{19}N$: 834.2815, found: 834.2818.

*N-[2,3,6-Tri-O-acetyl-4-O-(2,3,4,6-tetra-O-acetyl-β-D-galactopyranosyl)-β-D-glucopyranosyl]-N-[3-(1-methyl-1H-1,2,3-triazol-4-yl)phenyl)]acetamide (**2aa**)*

Iodomethane (0.65 mL, 1.0 mmol) was dissolved in DMF (3 mL), and sodium azide (0.065 g, 1.0 mmol) was added. The reaction mixture was stirred until complete dissolution of all components. Alkyne **2q** (0.210 g, 0.270 mmol) was dissolved in DMF (3 mL) and added to the mixture. Subsequently, copper(I) iodide (0.072 g; 0.378 mmol) and DIPEA (0.250 mL) were added, and the reaction was stirred for 5 h. The reaction mixture was diluted with EtOAc (50 mL) and washed with saturated aqueous NH_4Cl (15 mL) and brine (15 mL). The organic layer was

separated, dried over anhydrous $MgSO_4$, filtered, and concentrated in vacuo. The residue was purified by liquid column chromatography on silica gel (40% → 100% EtOAc in CyH) to give product **2aa** (0.170 g, 75%) as a white foam. $[\alpha]_D^{20}$ = +22.9 (*c* 0.4 in $CHCl_3$); **^{1}H NMR** (500 MHz, DMSO-d_6; T = 100 °C) δ 1.87 (s, 3H, CH_3CO), 1.90 (s, 3H, CH_3CO), 1.91 (s, 3H, CH_3CON), 1.94 (s, 3H, CH_3CO), 1.98 (s, 3H, CH_3CO), 2.00 (s, 3H, CH_3CO), 2.05 (s, 3H, CH_3CO), 2.08 (s, 3H, CH_3CO), 3.70 (t, $J_{4,5}$ = $J_{4,3}$ = 9.5 Hz, 1H, H-4), 3.94 (ddd, $J_{5,4}$ = 9.8, $J_{5,6a}$ = 5.1, $J_{5,6b}$ = 2.2 Hz, 1H, H-5), 3.99 (dd, J_{gem} = 11.2, $J_{6'a,5'}$ = 6.8 Hz, 1H, H-6'a), 4.02 (dd, J_{gem} = 11.2, $J_{6'b,5'}$ = 6.1 Hz, 1H, H-6'b), 4.10 (s, 3H, CH_3), 4.11 (dd, J_{gem} = 12.1, $J_{6a,5}$ = 5.1 Hz, 1H, H-6a), 4.14 (ddd, $J_{5',6'a}$ = 6.7, $J_{5',6'b}$ = 6.0, $J_{5',4'}$ = 1.3 Hz, 1H, H-5'), 4.47 (dd, J_{gem} = 12.1, $J_{6b,5}$ = 2.2 Hz, 1H, H-6b), 4.49 (t, $J_{2,1}$ = $J_{2,3}$ = 9.5 Hz, 1H, H-2), 4.73 (d, $J_{1',2'}$ = 7.9 Hz, 1H, H-1'), 4.87 (dd, $J_{2',3'}$ = 10.3, $J_{2',1'}$ = 7.9 Hz, 1H, H-2'), 5.11 (dd, $J_{3',2'}$ = 10.3, $J_{3',4'}$ = 3.6 Hz, 1H, H-3'), 5.21 (t, $J_{3,4}$ = $J_{3,2}$ = 9.1 Hz, 1H, H-3), 5.24 (dd, $J_{4',3'}$ = 3.7, $J_{4',5'}$ = 1.2 Hz, 1H, H-4'), 5.98 (d, $J_{1,2}$ = 9.6 Hz, 1H, H-1), 7.11 (ddd, $J_{6'',5''}$ = 7.9, $J_{6'',2''}$ = 2.1, $J_{6'',4''}$ = 1.1 Hz, 1H, H-6''), 7.47 (t, $J_{5'',6''}$ = $J_{5'',4''}$ = 7.8 Hz, 1H, H-5''), 7.62 (t, $J_{2'',6''}$ = $J_{2'',4''}$ = 1.8 Hz, 1H, H-2''), 7.83 (ddd $J_{4'',5''}$ = 7.8, $J_{4'',2''}$ = 1.7, $J_{4'',6''}$ = 1.1 Hz, 2H), 8.35 (s, 1H, H-5-triazole); **^{13}C NMR** (126 MHz, DMSO-d_6; T = 100 °C) δ 19.48, 19.53, 19.59, 19.60, 19.65, 19.72, 19.77 (7 × **C**H_3CO-2,3,6,2',3',4',6'), 22.19 (**C**H_3CON), 35.75 (CH_3), 60.47 (CH_2-6'), 61.38 (CH_2-6), 66.91 (CH-4'), 68.53 (CH-2), 68.90 (CH-2'), 69.58 (CH-5'), 70.12 (CH-3'), 73.43 (CH-3), 73.59 (CH-5), 74.92 (CH-4), 80.40 (CH-1), 99.18 (CH-1'), 121.82 (CH-5-triazole), 124.65 (CH-4''), 126.03 (CH-2''), 128.20 (CH-6''), 128.91 (CH-5''), 131.57 (C-3''), 137.72 (C-1''), 145.23 (C-4-triazole), 168.00, 168.25, 168.59, 168.60, 169.01, 169.03, 169.40 (7 × CH_3**C**O-2,3,6,2',3',4',6'), 170.04 (CH_3**C**ON); **IR** ($CHCl_3$) 3142, 2954, 2939, 2877, 1753, 1678, 1613, 1587, 1558, 1486, 1447, 1430, 1371, 1230, 1077, 1056, 1003, 703, 601 cm^{-1}; **HRMS** (ESI) $[M+Na]^+$ *m/z* calcd for $C_{37}H_{46}O_{18}N_4Na$: 857.2699, found: 857.2702; $[M+H]^+$ *m/z* calcd for $C_{37}H_{47}O_{18}N_4$: 835.2880, found: 835.2888.

*N-[2,3,6-Tri-O-acetyl-4-O-(2,3,4,6-tetra-O-acetyl-β-D-galactopyranosyl)-β-D-glucopyranosyl]-N-(3-carboxy-5-chlorophenyl)acetamide (**2ab**)*

Following General procedure A, compound **1** (0.318 g, 0.5 mmol) was reacted with 3-amino-5-chlorobenzoic acid (0.146 g, 0.85 mmol) for 3 days. The crude product was then subjected to General procedure B, affording product **2ab** (0.382 g, 92%) as a white foam. $[\alpha]_D^{20}$ = +34.8 (*c* 0.4 in $CHCl_3$); **^{1}H NMR** (500 MHz, DMSO-d_6; T = 100 °C) δ 1.89 (s, 3H, CH_3CO), 1.90 (s, 3H, CH_3CO), 1.96 (s, 3H, CH_3CO), 1.97 (s, 3H, CH_3CO), 1.98 (s, 3H, CH_3CON), 2.00 (s, 3H, CH_3CO), 2.08 (s, 3H, CH_3CO), 2.09 (s, 3H, CH_3CO), 3.74 (t, $J_{4,3}$ = $J_{4,5}$ = 9.5 Hz, 1H, H-4), 3.98 (ddd, $J_{5,4}$ = 8.9, $J_{5,6a}$ = 4.9, $J_{5,6b}$ = 1.7 Hz, 1H, H-5), 4.01 (dd, J_{gem} = 11.2, $J_{6'a,5'}$ = 6.7 Hz, 1H, H-6'a), 4.04 (dd, J_{gem} = 11.2, $J_{6'b,5'}$ = 6.1 Hz, 1H, H-6'b), 4.09 (dd, J_{gem} = 12.2, $J_{6a,5}$ = 4.8 Hz, 1H, H-6a), 4.15 (td, $J_{5',6a'}$ = $J_{5',6b'}$ = 6.4, $J_{5',4'}$ =1.1 Hz, 1H, H-5'), 4.40 (t, $J_{2,3}$ = $J_{2,1}$ = 9.4 Hz, 1H, H-2), 4.47 (dd, J_{gem} = 12.1, $J_{6b,5}$ = 2.1 Hz, 1H, H-6b), 4.73 (d, $J_{1',2'}$ = 7.9 Hz, H-1'), 4.87 (dd, $J_{2',3'}$ = 10.3, $J_{2',1'}$ = 7.9 Hz, 1H, H-2'), 5.12 (dd, $J_{3',2'}$ = 10.3, $J_{3',4'}$ = 3.6 Hz, 1H, H-3'), 5.22 (t, $J_{3,4}$ = $J_{3,2}$ = 9.2 Hz, 1H, H-3), 5.25 (dd, $J_{4',3'}$ = 3.8, $J_{4',5'}$ = 1.3 Hz, 1H, H-4'), 5.94 (d, $J_{1,2}$ = 9.6 Hz, 1H, H-1), 7.45 (t, $J_{6'',2''}$ = $J_{6'',4''}$ = 2.0 Hz, 1H, H-6''), 7.66 (t, $J_{2'',4''}$ = $J_{2'',6''}$ = 1.6 Hz, 1H, H-2''), 7.90 (dd, $J_{4'',6''}$ = 2.0, $J_{4'',2''}$ = 1.4 Hz, 1H, H-4''); **^{13}C NMR** (126 MHz, DMSO-d_6; T = 100 °C) δ 19.51, 19.56, 19.58, 19.62, 19.63, 19.65, 19.76 (7 × **C**H_3CO-2,3,6,2',3',4',6'), 22.03

(**C**H_3CON), 60.50 (CH_2-6'), 61.18 (CH_2-6), 66.93 (CH-4'), 68.31 (CH-2), 68.92 (CH-2'), 69.62 (CH-5'), 70.14 (CH-3'), 73.17 (CH-3), 73.56 (CH-5), 74.77 (CH-4), 80.67 (CH-1), 99.23 (CH-1'), 128.20 (CH-4''), 128.67 (CH-2''), 133.00 (C-5''), 133.06 (CH-6''), 133.24 (C-3''), 138.64 (C-1''), 164.69 (**C**OOH), 168.07, 168.28, 168.59, 168.64, 169.05, 169.07, 169.37 (7 × CH_3**C**O-2,3,6,2',3',4',6), 170.00 (CH_3**C**ON); **IR** ($CHCl_3$) 3514, 2941, 2876, 1753, 1704, 1685, 1600, 1577, 1449, 1371, 1302, 1271, 1250, 1056, 828, 601 cm^{-1}; **HRMS** (ESI) $[M+Na]^+$ *m/z* calcd for $C_{35}H_{42}O_{20}NClNa$: 854.1881, found: 854.1879.

*N-[2,3,6-Tri-O-acetyl-4-O-(2,3,4,6-tetra-O-acetyl-β-D-galactopyranosyl)-β-D-glucopyranosyl]-N-(3-bromo-5-carboxyphenyl)acetamide (**2ac**)*

Following General procedure A, compound **1** (0.318 g, 0.5 mmol) was reacted with 3-amino-5-bromobenzoic acid (0.184 g, 0.85 mmol) for 3 days. The crude product was then subjected to General procedure B, affording product **2ac** (0.367 g, 84%) as a white foam. $[\alpha]_D^{20}$ = +28.7 (*c* 0.4 in $CHCl_3$); **^{1}H NMR** (500 MHz, DMSO-d_6; T = 100 °C) δ 1.89 (s, 3H, CH_3CO), 1.90 (s, 3H, CH_3CO), 1.96 (s, 3H, CH_3CO), 1.97 (s, 3H, CH_3CO), 1.98 (s, 3H, CH_3CON), 2.00 (s, 3H, CH_3CO), 2.08 (s, 3H, CH_3CO), 2.10 (s, 3H, CH_3CO), 3.74 (dd, $J_{4,5}$ = 9.8, $J_{4,3}$ = 9.2 Hz, 1H, H-4), 3.98 (ddd $J_{5,4}$ = 9.5, $J_{5,6a}$ = 4.9, $J_{5,6b}$ = 2.2 Hz, 1H, H-5), 4.00 – 4.07 (m, 2H, H-6'), 4.09 (dd, J_{gem} = 12.1, $J_{6a,5}$ = 4.8 Hz, 1H, H-6a), 4.15 (td, $J_{5',6'}$ = 6.4, $J_{5',4'}$ = 1.3 Hz, 1H, H-5'), 4.40 (t, $J_{2,3}$ = $J_{2,1}$ = 9.4 Hz, 1H, H-2), 4.46 (dd, J_{gem} = 12.1, $J_{6b,5}$ = 2.1 Hz, 1H, H-6b), 4.73 (d, $J_{1',2'}$ = 7.9 Hz, 1H, H-1'), 4.87 (dd, $J_{2',3'}$ = 10.3, $J_{2',1'}$ = 7.9 Hz, 1H, H-2'), 5.12 (dd, $J_{3',2'}$ = 10.3, $J_{3',4'}$ = 3.6 Hz, 1H, H-3'), 5.23 (t, $J_{3,4}$ = $J_{3,2}$ = 9.1 Hz, 1H, H-3), 5.24 (dd, $J_{4',3'}$ = 3.6, $J_{4',5'}$ = 1.3 Hz, 1H, H-4'), 5.94 (d, $J_{1,2}$ = 9.6 Hz, 1H, H-1), 7.58 (t, $J_{6'',2''}$ = $J_{6'',4''}$ = 1.9 Hz, 1H, H-6''), 7.70 (t, $J_{2'',4''}$ = $J_{2'',6''}$ = 1.6 Hz, 1H, H-2''), 8.04 (t, $J_{4'',2''}$ = $J_{4'',6''}$ = 1.6 Hz, 1H, H-4''); **^{13}C NMR** (126 MHz, DMSO-d_6; T = 100 °C) δ 19.51, 19.56, 19.58, 19.63, 19.63, 19.65, 19.80 (7 × **C**H_3CO-2,3,6,2',3',4',6'), 22.02 (**C**H_3CON), 60.50 (CH_2-6'), 61.18 (CH_2-6), 66.93 (CH-4'), 68.30 (CH-2), 68.92 (CH-2'), 69.61 (CH-5'), 70.13 (CH-3'), 73.16 (CH-3), 73.56 (CH-5), 74.76 (CH-4), 80.66 (CH-1), 99.22 (CH-1'), 120.86 (C-5''), 129.03 (CH-2''), 131.11 (CH-4''), 133.40 (C-3''), 135.88 (CH-6''), 138.72 (C-1''), 164.58 (COOH), 168.05, 168.27, 168.59, 168.63, 169.05, 169.07, 169.36 (7 × CH_3**C**O-2,3,6,2',3',4',6'), 170.01 (CH_3**C**ON); **IR** ($CHCl_3$) 3511, 2939, 2876, 1753, 1696, 1680, 1599, 1571, 1448, 1371, 1253, 1200, 1056, 601 cm^{-1}; **HRMS** (ESI) $[M+Na]^+$ *m/z* calcd for $C_{35}H_{42}O_{20}NBrNa$: 898.1376, found: 898.1380.

*N-[2,3,6-Tri-O-acetyl-4-O-(2,3,4,6-tetra-O-acetyl-β-D-galactopyranosyl)-β-D-glucopyranosyl]-N-[6-(methylcarboxy)-1H-indol-4-yl]acetamide (**2ae**)*

Following General procedure A, compound **1** (0.636 g, 1.0 mmol) was reacted with 6-methylcarboxy-4-amino-1*H*-indole (0.242 g, 1.27 mmol) for 3 days. The crude product was then subjected to General procedure B, affording product **2ac** (0.450 g, 53%) as a white foam. $[\alpha]_D^{20}$ = +37.2 (*c* 0.3 in $CHCl_3$); **^{1}H NMR** (500 MHz, DMSO-d_6; 100°C) δ 1.67 – 1.79 (m, 3H, CH_3CO), 1.83 (s, 6H, CH_3CO), 1.90 (s, 3H, CH_3CO), 1.94 (s, 3H, CH_3CO), 2.01 (s, 3H, CH_3CON), 2.07 (s, 3H, CH_3CO), 2.09 – 2.14 (m, 3H, CH_3CO), 3.55 – 3.72 (m, 1H, H-4), 3.88 (s, 3H, OCH_3), 3.90 – 3.95

(m, 1H, H-5), 3.96 – 4.04 (m, 2H, H-6'), 4.09 – 4.20 (m, 2H, H-5',6a), 4.37 – 4.56 (m, 2H, H-2,6b), 4.64 – 4.77 (m, 1H, H-1'), 4.86 (dd, $J_{2',3'}$ = 10.2, $J_{2',1'}$ = 8.0 Hz, 1H, H-2'), 5.11 (dd, $J_{3',2'}$ = 10.2, $J_{3',4'}$ = 3.6 Hz, 1H, H-3'), 5.16 (t, $J_{3,4}$ = $J_{3,2}$ = 9.1 Hz, 1H, H-3), 5.24 (dd, $J_{4',3'}$ = 3.6, $J_{4',5'}$ = 1.1 Hz, 1H, H-4'), 5.90 – 5.99 (m, 1H, H-1), 6.13 – 6.80 (m, 1H, H-3^{ind}), 7.56 (dd, $J_{2ind,3ind}$ = 2.8, $J_{2ind,7ind}$ = 1.8 Hz, 1H, H-2^{ind}), 7.59 – 7.66 (m, 1H, H-5^{ind}), 8.10 (t, $J_{7ind,5ind}$ = $J_{7ind,2ind}$ = 1.1 Hz, 1H, H-7^{ind}), 11.40 (s, 1H, NH); **^{13}C NMR** (126 MHz, DMSO-d_6; 100°C) δ 19.52, 19.56, 19.61, 19.64, 19.65, 19.84 (7 × **C**H_3CO-2,3,6,2',3',4',6'), 21.81 (**C**H_3CON), 51.13 (OCH_3), 60.51 (CH_2-6'), 61.47 (CH_2-6), 66.94 (CH-4'), 68.15 (CH-2), 68.91 (CH-2'), 69.59 (CH-5'), 70.15 (CH-3'), 73.47 (CH-3), 73.71 (CH-5), 75.00 (CH-4), 80.99 (CH-1), 99.18 (CH-1'), 99.78 (CH-3^{ind}), 113.34 (CH-7^{ind}), 119.04 (CH-5^{ind}), 122.25 (C-6^{ind}) 129.32 (CH-2^{ind}), 136.01 (C-7a^{ind}), 166.14, 168.28, 168.61, 168.64, 169.05, 169.07, 169.42 (7 × CH_3**C**O-2,3,6,2',3',4',6'), 170.33 (CH_3**C**ON); some quaternary signals of indole were not observed due to extensive broadening. **IR** ($CHCl_3$) 3472, 2954, 2874, 2847, 1753, 1714, 1675, 1603, 1576, 1509, 1496, 1456, 1440, 1433, 1370, 1233, 1080, 1054, 602 cm^{-1}; **HRMS** (ESI) $[M+Na]^+$ *m/z* calcd for $C_{38}H_{46}O_{20}N_2Na$: 873.2536, found: 873.2537.

*Methyl 2,3,6-tri-O-benzoyl-4-S-(2,4,6-tri-O-acetyl-3-deoxy-3-(4-(3-fluorophenyl)-1H-1,2,3-triazol-1-yl)-β-D-galactopyranosyl)-4-thio-α-D-glucopyranoside (**6**)*

Thiogalactose **4** (0.850 g, 1.8 mmol)[10] and triflate **5** (1.215 g, 1.9 mmol)[10] were dissolved in THF (20 mL), and 1,4-dithio-D-threitol (0.139 g, 0.9 mmol) and Et_3N (1 mL) were added. The mixture was stirred at room temperature for 15 h, then concentrated in vacuo. The residue was dissolved in EtOAc (150 mL) and washed with H_2O (30 mL) and brine (30 mL). The organic layer was separated, dried over anhydrous $MgSO_4$, filtered, and concentrated in vacuo. The residue was purified by trituration from a mixture of Et_2O/CH_2Cl_2 affording product **6** (1.025 g, 60%) as a white powder. $[\alpha]_D^{20}$ = +81.1 (*c* 0.2 in $CHCl_3$). **^{1}H NMR** (500 MHz, $CDCl_3$; T = 25 °C) δ 1.41 (s, 3H, CH_3CO-2'), 2.01 (s, 3H, CH_3CO-4'), 2.07 (s, 3H, CH_3CO-6'), 3.37 (t, 1H, $J_{4,3}$ = $J_{4,5}$ = 11.1, H-4), 3.49 (s, 3H, CH_3O), 4.02 (m, 1H, H-6'b), 4.09 – 4.14 (m, 2H, H-5',6'a), 4.46 (ddd, 1H, $J_{5,4}$ = 11.1, $J_{5,6}$ = 4.1, 2.1, H-5), 4.82 (dd, 1H, J_{gem} = 12.1, $J_{6b,5}$ = 2.1, H-6b), 4.91 (dd, 1H, J_{gem} = 12.1, $J_{6a,5}$ = 4.1, H-6a), 5.14 (dd, 1H, $J_{3',2'}$ = 10.9, $J_{3',4'}$ = 3.2, H-3'), 5.16 (d, 1H, $J_{1',2'}$ = 9.6, H-1'), 5.22 (d, 1H, $J_{1,2}$ = 3.6, H-1), 5.28 (dd, 1H, $J_{2,3}$ = 9.7, $J_{2,1}$ = 3.6, H-2), 5.57 (d, 1H, $J_{4',3'}$ = 3.2, H-4'), 5.61 (dd, 1H, $J_{2',3'}$ = 10.9, $J_{2',1'}$ = 9.6, H-2'), 6.06 (dd, 1H, $J_{3,4}$ = 11.1, $J_{3,2}$ = 9.7, H-3), 7.02 (dddd, 1H, $J_{H,F}$ = 11.0, $J_{4,5}$ = 8.5, $J_{4,2}$ = 2.6, $J_{4,6}$ = 1.0, H-4-C_6H_4F), 7.32 – 7.42 (m, 5H, H-5-C_6H_4F, H-*m*-Bz-2,3), 7.46 – 7.55 (m, 6H, H-2,6-C_6H_4F, H-*p*-Bz-2,3, H-*m*-Bz-6), 7.63 (m, 1H, H-*p*-Bz-6), 7.72 (s, 1H, H-5-triazole), 7.96 – 8.01 (m, 4H, H-*o*-Bz-2,3), 8.10 – 8.13 (m, 2H, H-*o*-Bz-6); **^{13}C NMR** (126 MHz, $CDCl_3$; T = 25 °C) δ 19.74 (**C**H_3CO-2'), 20.37 (**C**H_3CO-4'), 20.63 (**C**H_3CO-6'), 46.51 (CH-4), 55.84 (CH_3O), 61.38 (CH_2-6'), 62.96 (CH-3'), 63.97 (CH_2-6), 65.84 (CH-2'), 67.39 (CH-3), 68.54 (CH-4'), 69.48 (CH-5), 73.27 (CH-2), 75.07 (CH-5'), 82.74 (CH-1'), 97.36 (CH-1), 112.71 (d, $J_{C,F}$ = 23.1, CH-2-C_6H_4F), 115.29 (d, $J_{C,F}$ = 21.2, CH-4-C_6H_4F), 118.60 (CH-5-triazole), 121.29 (d, $J_{C,F}$ = 2.7, CH-6-C_6H_4F), 128.43, 128.46, 128.62 (CH-*m*-Bz-2,3,6), 128.97, 129.12 (C-*i*-Bz-2,3), 129.68, 129.83, 129.86, 129.90 (CH-*o*-Bz-2,3,6, C-*i*-Bz-6), 130.50 (d, $J_{C,F}$ = 8.3, CH-5-C_6H_4F), 132.10 (d, $J_{C,F}$ = 8.3, C-1-C_6H_4F), 133.36, 133.47, 133.49 (CH-*p*-Bz-2,3,6), 146.78 (d, $J_{C,F}$ = 2.5, C-4-triazole), 163.15 (d, $J_{C,F}$ = 246.1, C-3-C_6H_4F)

165.69 (CO-Bz-3), 165.80 (CO-Bz-2), 166.14 (CO-Bz-6), 168.77 (CH_3**C**O-4'), 169.55 (CH_3**C**O-2'), 170.14 (CH_3**C**O-6'); **^{19}F NMR** (471 MHz, $CDCl_3$; T = 25 °C): -112.27 (ddd, $J_{F,H}$ = 11.0, 7.5, 6.2); **IR** ($CHCl_3$) 3152, 3092, 3065, 3029, 2686, 1752, 1724, 1621, 1603, 1591, 1585, 1559, 1488, 1480, 1459, 1452, 1373, 1316, 1271, 1248, 1178, 1127, 1108, 1071, 1043, 1027, 1002, 919, 866, 686, 600 cm^{-1}; **HRMS** (ESI) $[M+Na]^+$ *m/z* calcd for $C_{48}H_{46}O_{15}N_3FNaS$: 978.2526, found: 978.2524.

1-*O*-Acetyl-2,3,6-tri-O-benzoyl-4-S-(2,4,6-tri-O-acetyl-3-deoxy-3-(4-(3-fluorophenyl)-1H-1,2,3-triazol-1-yl)-β-D-galactopyranosyl)-4-thio-α-D-glucopyranose (**S4**)

Thiolactoside **6** (0.955 g, 1 mmol) was dissolved in Ac_2O (18 mL) and stirring at room temperature. A solution of concentrated H_2SO_4 (2.5 mL) in glacial AcOH (6 mL) was then added, and the reaction mixture was heated to 40 °C and stirred for 10 minutes. The mixture was poured into a beaker containing pyridine (15 mL) and crushed ice (100 mL) and stirred for 1 h. The resulting solution was transferred to a separatory funnel containing CH_2Cl_2 (200 mL) and washed with H_2O (2 × 100 mL), saturated solution of $NaHCO_3$ (2 × 100 mL) and brine (100 mL). The organic layer was separated, dried over anhydrous $MgSO_4$, filtered, and concentrated in vacuo. The residue was purified by trituration from a mixture of Et_2O/CH_2Cl_2, affording the product **S4** (768 mg, 78 %) as a white solid. $[\alpha]_D^{20}$= +72.6 (*c* 0.2 in $CHCl_3$); **^{1}H NMR** (401 MHz, $CDCl_3$; T = 25 °C) δ 1.46 (s, 3H, CH_3CO-2'), 1.99 (s, 3H, CH_3CO-4'), 2.06 (s, 3H, CH_3CO-6'), 2.22 (s, 3H, CH_3CO-1), 3.49 (t, $J_{4,3} = J_{4,5}$ = 11.2 Hz, 1H, H-4), 3.97 – 4.09 (m, 3H, H-5', H-6'), 4.56 (ddd, $J_{5,4}$ =11.1, $J_{5,6a}$ = 3.2, $J_{5,6b}$ = 2.0 Hz, 1H, H-5), 4.71 (dd, J_{gem} = 12.3, $J_{6b,5}$ = 1.9 Hz, 1H, H-6b), 4.96 (dd, J_{gem} = 12.4, $J_{6a,5}$ = 3.3 Hz, 1H, H-6a), 5.10 (dd, $J_{3',2'}$ = 10.9, $J_{3',4'}$ = 3.4 Hz, 3H, H-3'), 5.10 (d, $J_{1',2'}$ = 9.7 Hz, 1H, H-1'), 5.50 (dd, $J_{2,3}$ = 9.8, $J_{2,1}$ = 3.7 Hz, 1H, H-2), 5.52 (dd, $J_{4',3'}$ = 3.4, 1.0 Hz, 1H, H-4'), 5.60 (dd, $J_{2',3'}$ = 10.9, $J_{2',1'}$ = 9.7 Hz, 1H, H-2'), 6.03 (dd, $J_{3,4}$ = 11.2, $J_{3,2}$ = 9.8 Hz, 1H, H-3), 6.62 (d, $J_{1,2}$ = 3.7 Hz, 1H, H-1), 7.02 (td, *J* = 7.9, 7.5, 1.5 Hz, 1H, H-4'-C_6H_4F), 7.32 – 7.42 (m, 5H, H-5'-C_6H_4F, 4 × H-*m*-BzO), 7.45 – 7.57 (m, 6H, H-2'-C_6H_4F, H-6'-C_6H_4F, 2xH-*m*-BzO, 2 × H-*p*-BzO), 7.62 – 7.68 (m, 1H, H-*p*-BzO), 7.88 – 7.94 (m, 2H, 2 × H-*o*-BzO), 7.98 – 8.03 (m, 2H, 2 × H-*o*-BzO), 8.09 – 8.12 (m, 2H, 2 × H-*o*-BzO); **^{13}C NMR** (101 MHz, $CDCl_3$; T = 25 °C) δ 19.94 (**C**H_3CO-2'), 20.50 (**C**H_3CO-4'), 20.72 (**C**H_3CO-6'), 21.04 (**C**H_3CO-1), 46.06 (C-4), 61.81 (C-6'), 63.04 (C-3'), 63.64 (C-6), 65.94 (C-2'), 67.38 (C-3), 68.60 (C-4'), 71.58 (C-2), 72.35 (C-5), 75.33 (C-5'), 83.17 (C-1'), 89.85 (C-1), 112.84 (d, $J_{C,F}$ = 23.1 Hz, C-2'-C_6H_4F), 115.49 (d, $J_{C,F}$ = 21.3 Hz, C-4'-C_6H_4F), 118.69 (C-5'-triazole), 121.43 (d, $J_{C,F}$ = 3.3 Hz, C-6'-C_6H_4F), 128.64, 128.68 (4 × C-*m*-OBz), 128.72 (C-*ipso*-OBz), 128.84 (2 × C-*m*-OBz), 128.94 (C-*ipso*-OBz), 129.82 (C-*ipso*-OBz), 129.87, 129.91, 130.07 (6 × C-*o*-OBz), 130.68 (d, $J_{C,F}$ = 8.4 Hz, C-5'-C_6H_4F), 132.18 (d, $J_{C,F}$ = 8.4 Hz, C-1'-C_6H_4F), 133.66, 133.79, 133.85 (3 × C-*p*-OBz), 146.99 (d, $J_{C,F}$ = 2.7 Hz, C-4'-triazole), 163.30 (d, $J_{C,F}$ = 246.1 Hz, C-3'-C_6H_4F), 165.45, 165.90, 166.13 (3 × C-OBz), 168.77 (CH_3**C**O-1), 168.90 (CH_3**C**O-4'), 169.66 (CH_3**C**O-2'), 170.31 (CH_3**C**O-6'); **^{19}F NMR** (377 MHz, $CDCl_3$; T = 25 °C) δ -112.33 (ddd, *J* = 10.1, 8.6, 5.8 Hz, C_6H_4**F**); **IR** ($CHCl_3$) 3151, 3092, 3073, 3065, 3031, 2976, 2872, 1755, 1729, 1663, 1621, 1591, 1585, 1559, 1495, 1488, 1480, 1452, 1442, 1373, 1316, 1270, 1246, 1178, 1108, 1096, 1071, 1065, 1044, 1026, 911, 888, 843, 711, 686, 617, 600, 526 cm^{-1}; **HRMS** (ESI) $[M+Na]^+$ *m/z* calcd for $C_{49}H_{47}O_{16}N_3FS$: 984.2656, found: 984.2640.

4-*S*-(3-Deoxy-3-(4-(3-fluorophenyl)-1*H*-1,2,3-triazol-1-yl)-β-D-galactopyranosyl)-4-thio-D-glucopyranose (**7**)

The compound **S4** (0.900 g, 0.915 mmol) was dissolved in CH_3OH (20 mL) and CH_2Cl_2 (5 mL), and the pH was adjusted to 9-10 by dropwise addition of 1M CH_3ONa in MeOH. The reaction mixture was stirred at room temperature for 18 h, after which Dowex 50WX8 (H^+ form) resin was added to neutralize the solution. The resulting slurry was filtered, and the resin was washed with CH_3OH. The combined filtrate was concentrated in vacuo, and the residue was purified by liquid column chromatography on silica gel (8:1 → 4:1 $CHCl_3/CH_3OH$) to give product **7** (0.380 g, 82%) as a white foam. The product was obtained as a mixture of anomers in a 53:47 (α/β) ratio. NMR data are reported for the α-anomer. $[\boldsymbol{\alpha}]_{\mathbf{D}}^{\mathbf{20}}$ = +61.5 (*c* 0.3 in CH_3OH); **^{1}H NMR** (500 MHz, Methanol-d_4; T = 25 °C) δ 2.95 (t, $J_{4,3} = J_{4,5}$ = 10.7 Hz, 1H, H-4), 3.48 (dd, $J_{2,3}$ = 9.3, $J_{2,1}$ = 3.6 Hz, 1H, H-2), 3.54 (ddd, $J_{5,4}$ = 10.9, $J_{5,6a}$ = 4.9, $J_{5,6b}$ = 2.0 Hz, 1H, H-5), 3.70 (dd, J_{gem} = 11.5, $J_{6'a,5'}$ = 5.2 Hz, 1H, H-6'b), 3.79 (dd, J_{gem} = 11.4, $J_{6'a,5}$ = 6.6 Hz, 1H, H-6'a), 3.87 (ddd, $J_{5',6'a}$ = 6.9, $J_{5',6'b}$ = 5.1, $J_{5',4'}$ = 1.2 Hz, 1H, H-5'), 3.92 – 3.99 (m, 2H, H-3,6a), 4.10 (dd, J_{gem} = 12.2, $J_{6b,5}$ = 2.0 Hz, 1H, H-5), 4.15 (dd, $J_{4',3'}$ = 3.0, $J_{4',5'}$ = 1.0 Hz, 1H, H-4'), 4.34 (dd, $J_{2',3'}$ = 11.0, $J_{2',1'}$ = 9.4 Hz, 1H, H-2'), 4.73 (d, $J_{1',2'}$ = 9.5 Hz, 1H, H-1'), 4.90 (dd, $J_{3',2'}$ = 10.8, $J_{3',4'}$ = 3.1 Hz, 1H, H-3'), 5.19 (d, $J_{1,2}$ = 3.6 Hz, 1H, H-1), 7.08 (tdd, $J_{2'',F}$ = 10.3, $J_{2'',4''}$ = 2.6, $J_{2'',6''}$ = 0.9 Hz, 1H, H-2''), 7.45 (td $J_{5'',4''} = J_{5'',6''}$ = 8.0, $J_{5'',F}$ = 5.9 Hz, 1H, H-5''), 7.61 (ddd, *J* = 10.1, 2.7, 1.5 Hz, 1H), 7.67 (dt, $J_{6'',5''}$ = 7.8, $J_{6'',2''} = J_{6'',4''}$ = 1.2 Hz, 1H, H-6''), 8.50 (s, 1H, H-5^{Tz}); **^{13}C NMR** (126 MHz, Methanol-d_4; T = 25 °C) δ 47.61 (CH-4), 62.48 (CH_2-6'), 63.26 (CH_2-6), 67.87 (CH-2'), 68.63 (CH-3'), 69.80 (CH-4'), 73.12 (CH-3), 74.86 (CH-2), 78.18 (CH-5), 81.26 (CH-5'), 87.20 (CH-1'), 94.03 (CH-1), 113.22 (d, $J_{C,F}$ = 23.4 Hz, CH-2''), 115.77 (d, $J_{C,F}$ = 21.4 Hz, CH-4''), 122.43 (d, $J_{C,F}$ = 2.8 Hz, CH-6''), 122.47 (C-4^{Tz}), 131.88 (d, $J_{C,F}$ = 8.6 Hz, CH-5''), 134.35 (d, $J_{C,F}$ = 8.5 Hz, C-1''), 147.17 (C-4^{Tz}), 164.68 (d, $J_{C,F}$ = 244.2 Hz, CF-3''); **^{19}F NMR** (470 MHz, Methanol-d_4; T = 25 °C) δ -114.81 (td, $J_{F,2''} = J_{F,4''}$ = 9.5, $J_{F,5''}$ = 5.8 Hz, 1F, F-3''). **HRMS** (ESI) $[M-H]^-$ *m/z* calcd for $C_{20}H_{25}O_9N_3FS$: 502.1301, found: 502.1303.

NMR spectra of prepared compounds

^{1}H and ^{13}C APT NMR spectra of (2a)

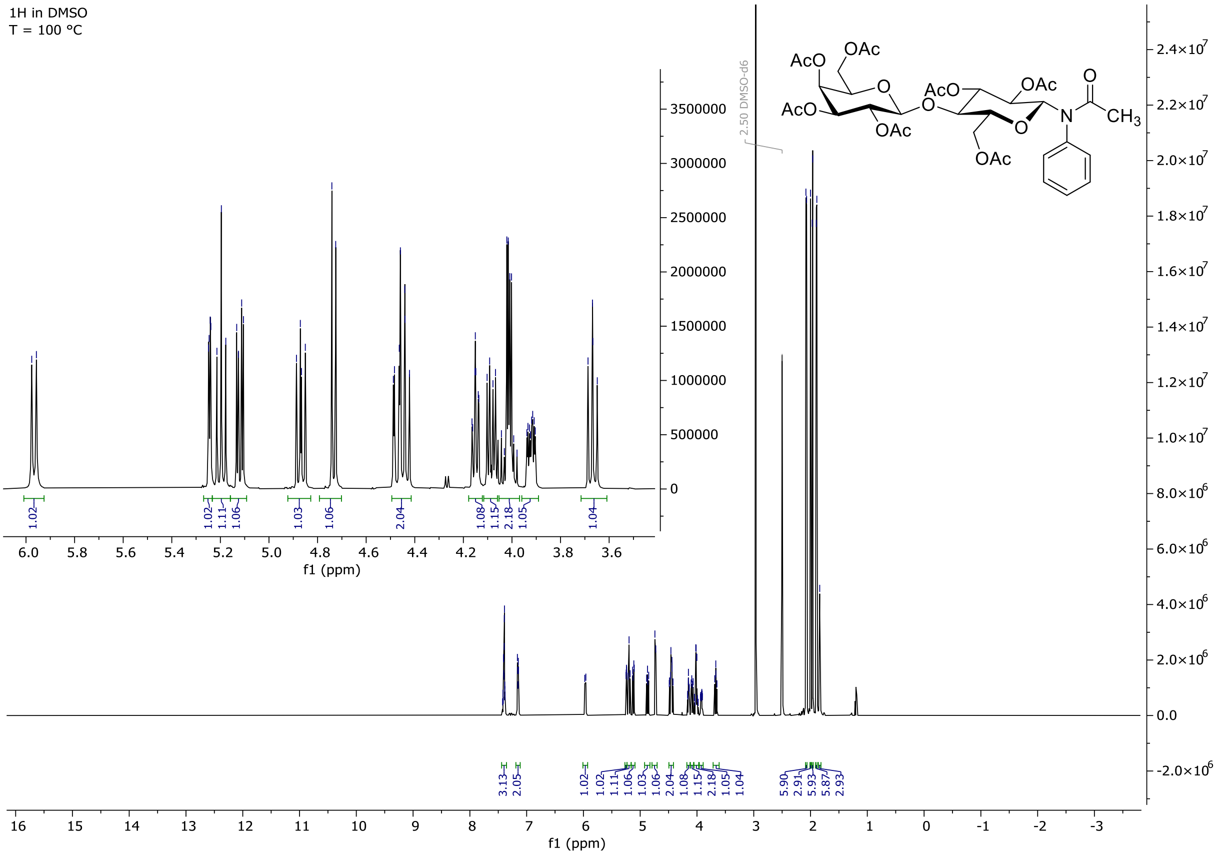

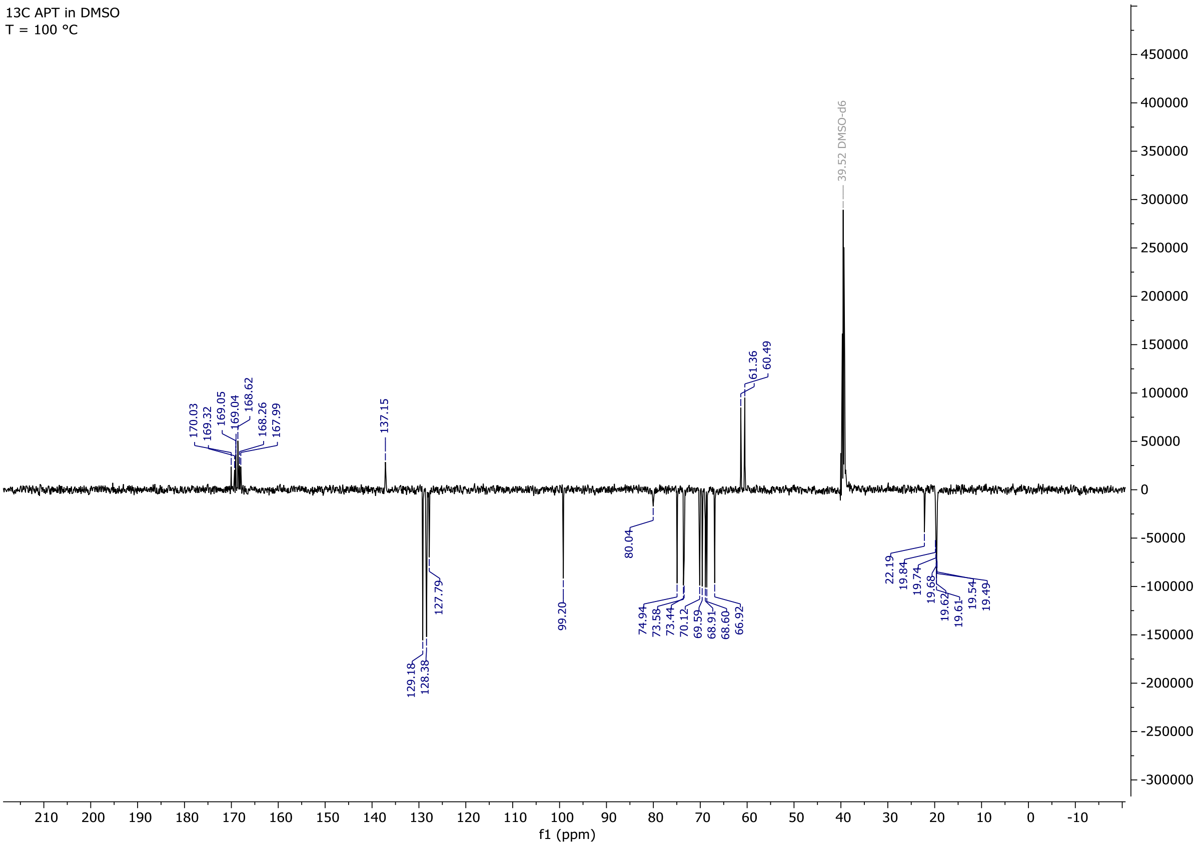

^{1}H, ^{13}C APT and HSQC NMR spectra of (2b)

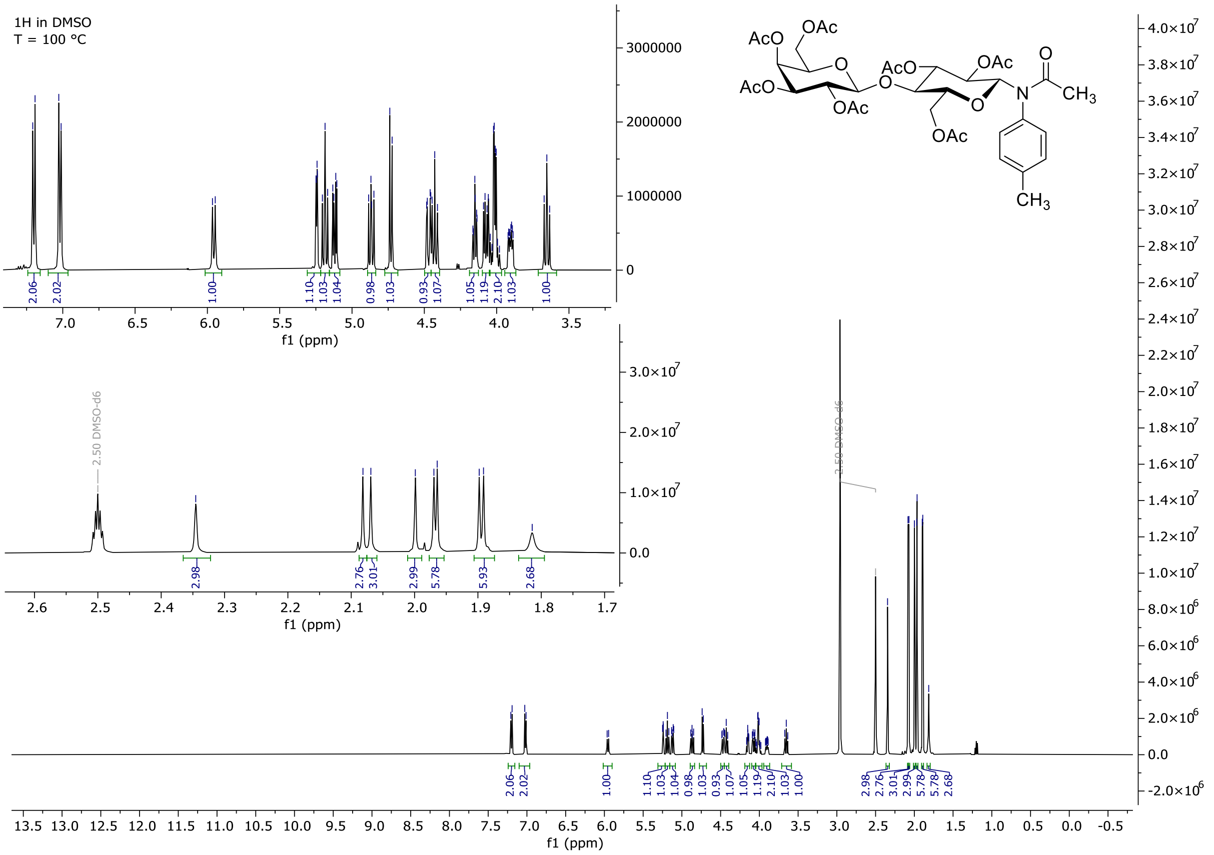

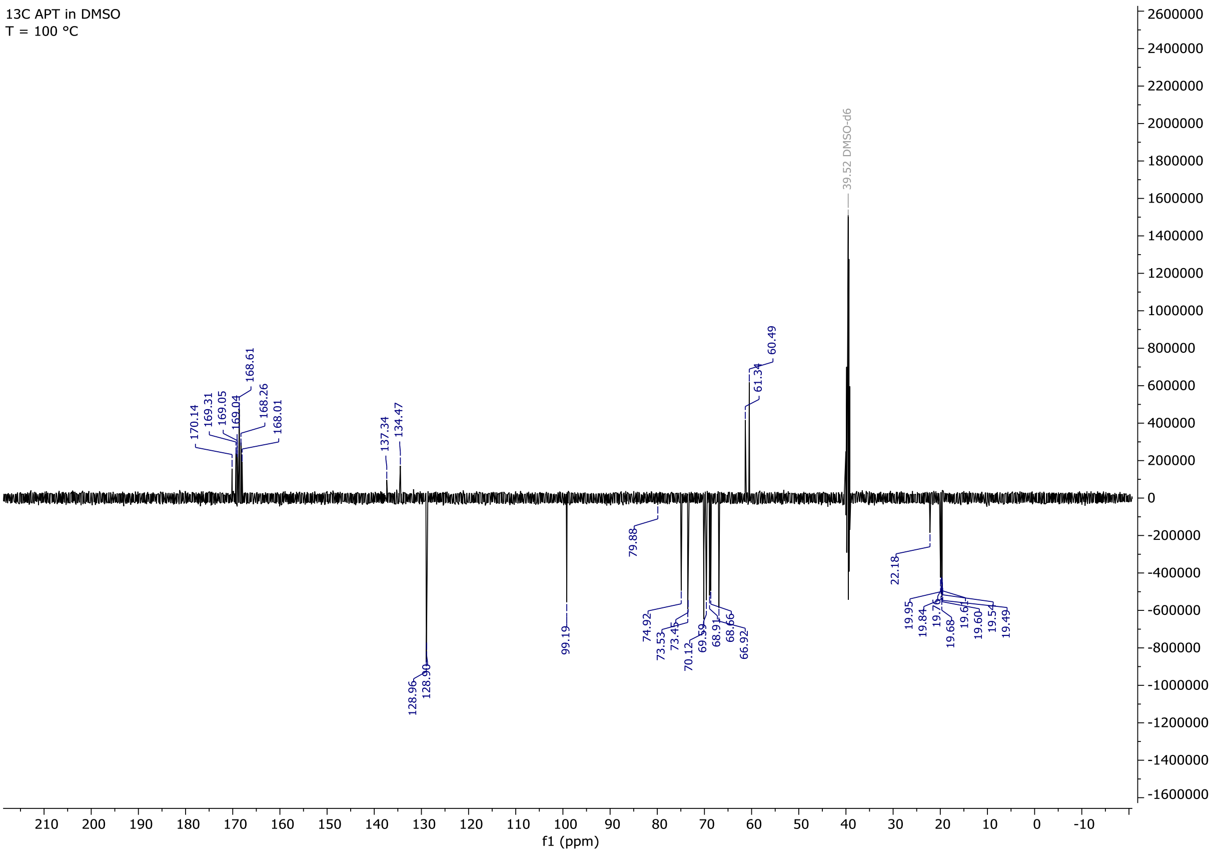

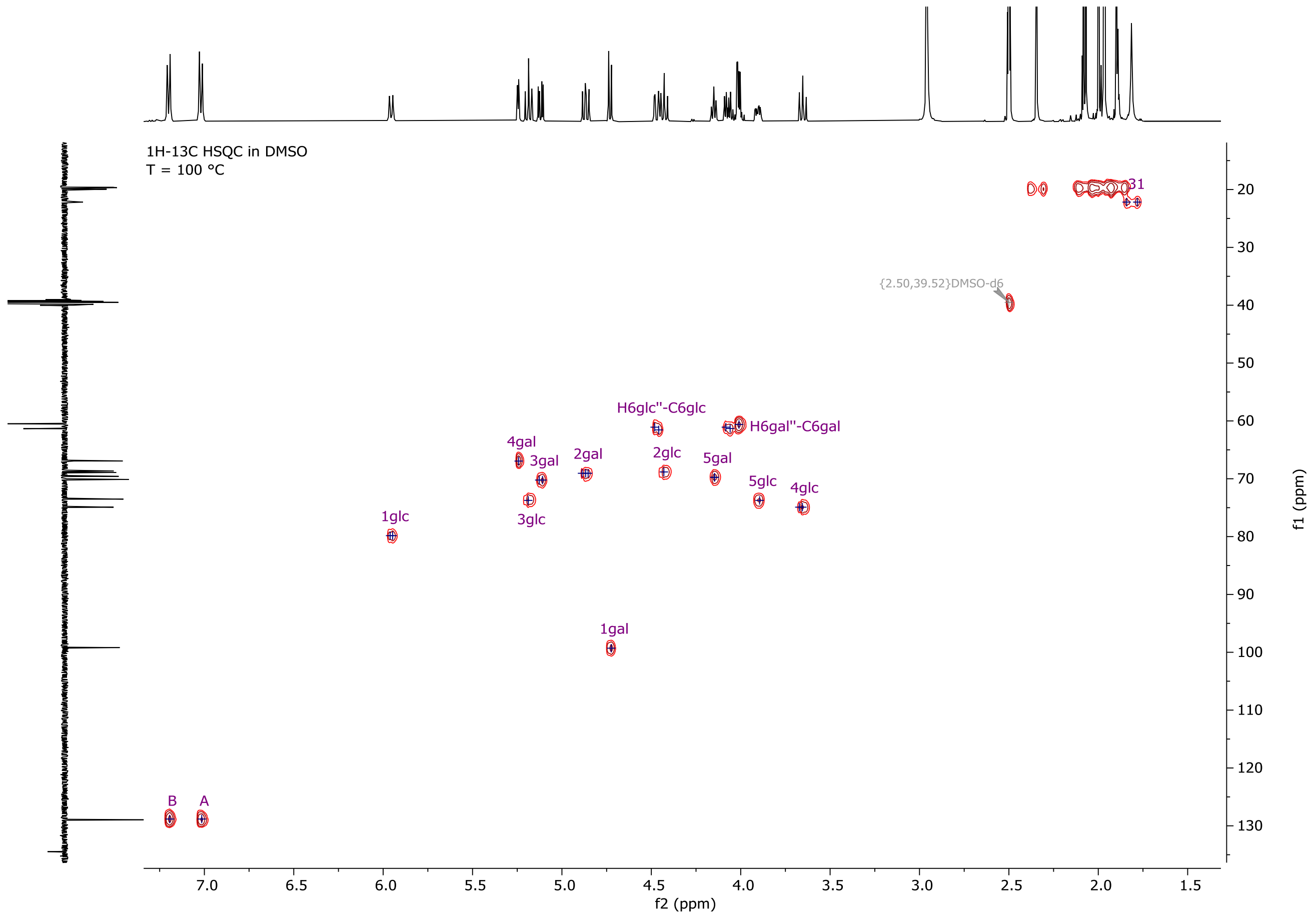

1H-13C HSQC in DMSO
T = 100 °C
31
{2.50,39.52}DMSO-d6
H6glc''-C6glc
H6gal''-C6gal
4gal
3gal
2gal
2glc
5gal
5glc
4glc
1glc
3glc
1gal
B
A
f2 (ppm)
f1 (ppm)

^{1}H, ^{13}C APT and HSQC NMR spectra of (2c)

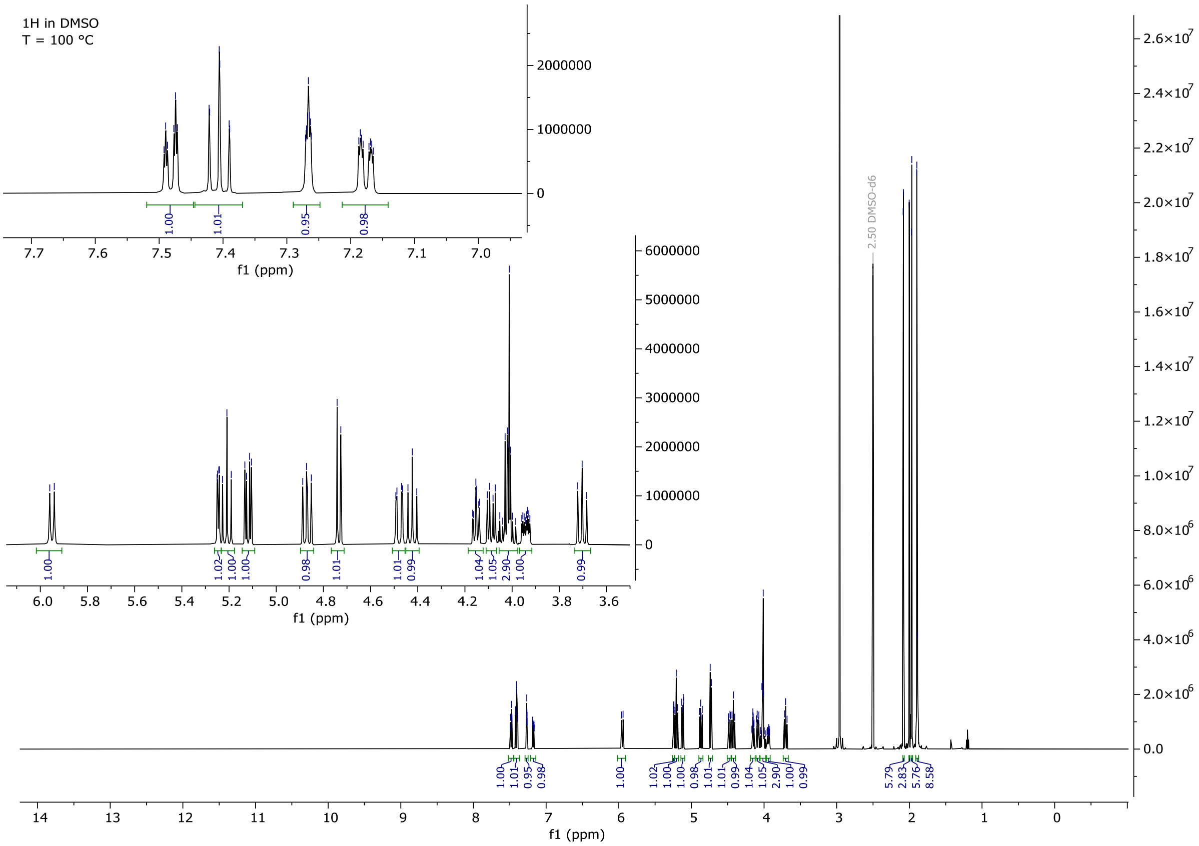

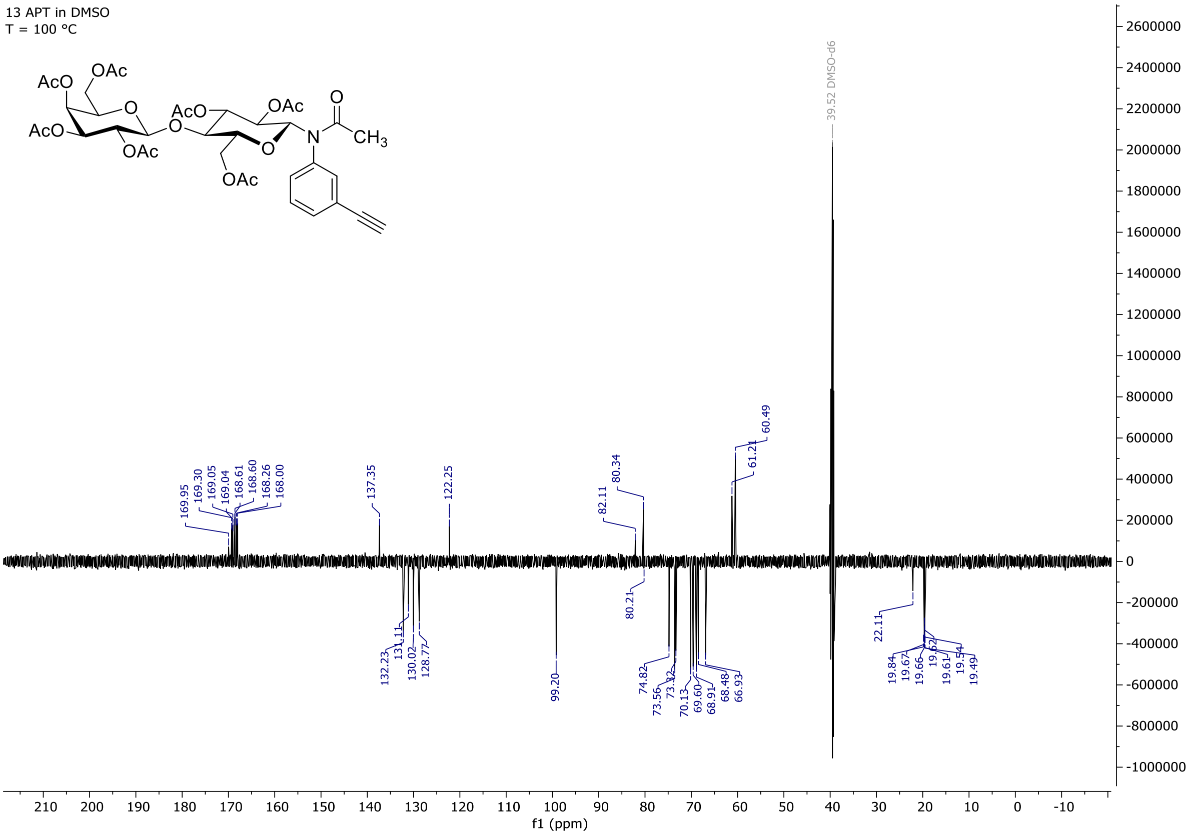

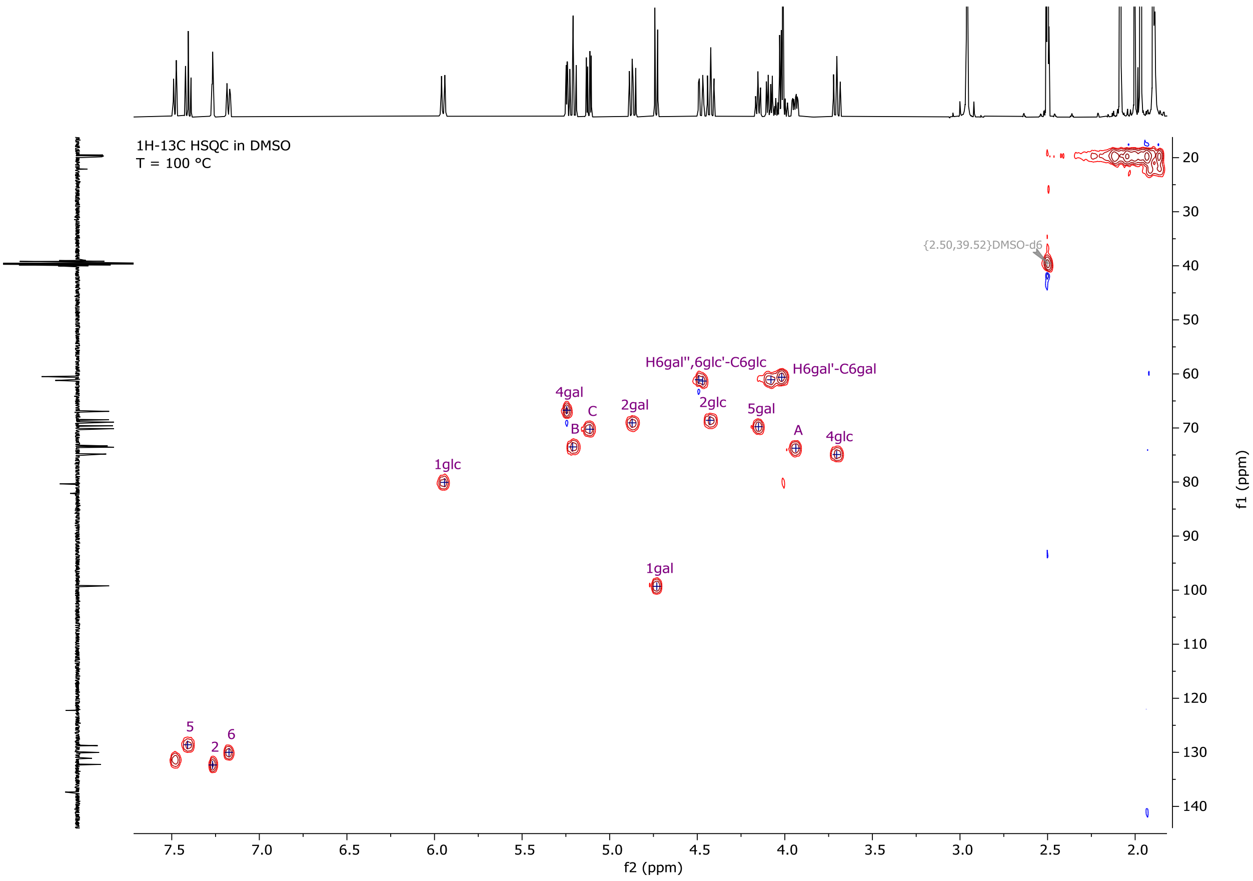
1H-13C HSQC in DMSO
T = 100 °C
{2.50,39.52}DMSO-d6
H6gal'',6glc'-C6glc
H6gal'-C6gal
4gal
C
2gal
2glc
5gal
B
A
4glc
1glc
1gal
5
2
6
f2 (ppm)
f1 (ppm)

^{1}H, ^{13}C APT and ^{19}F NMR spectra of (2d)

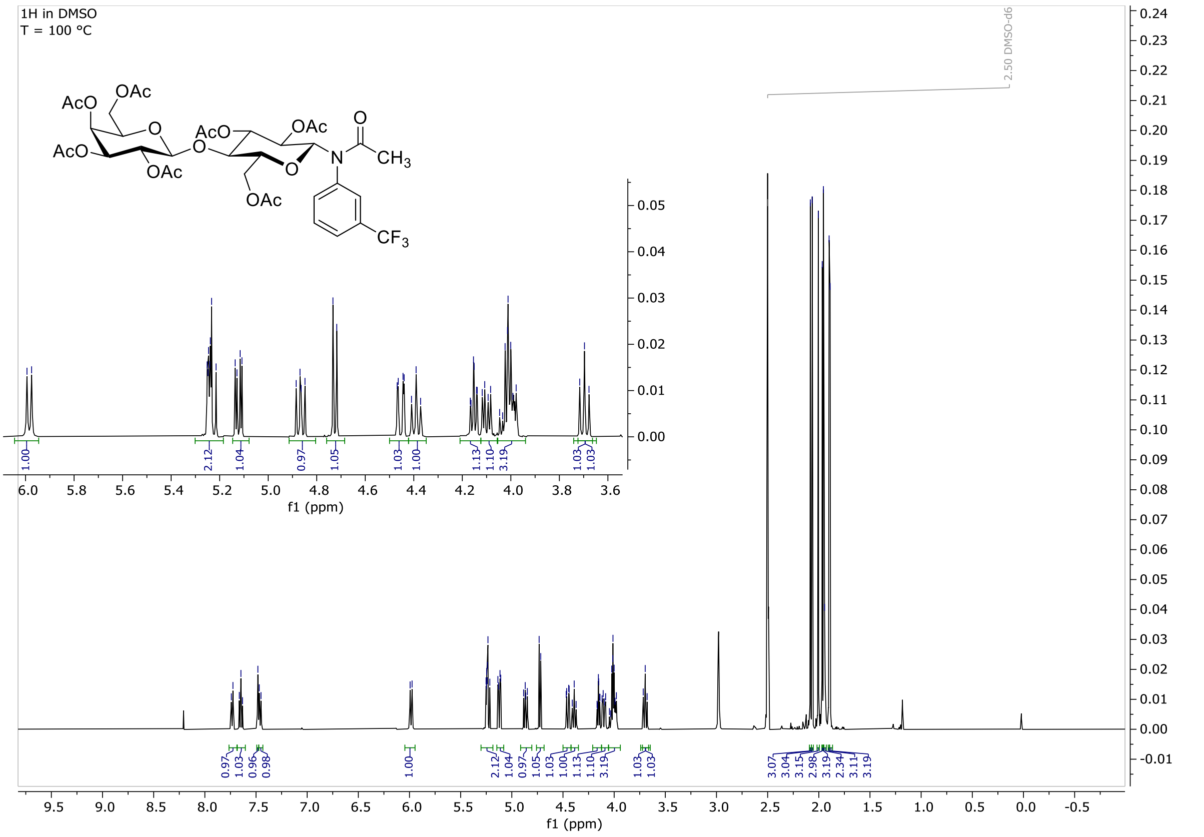

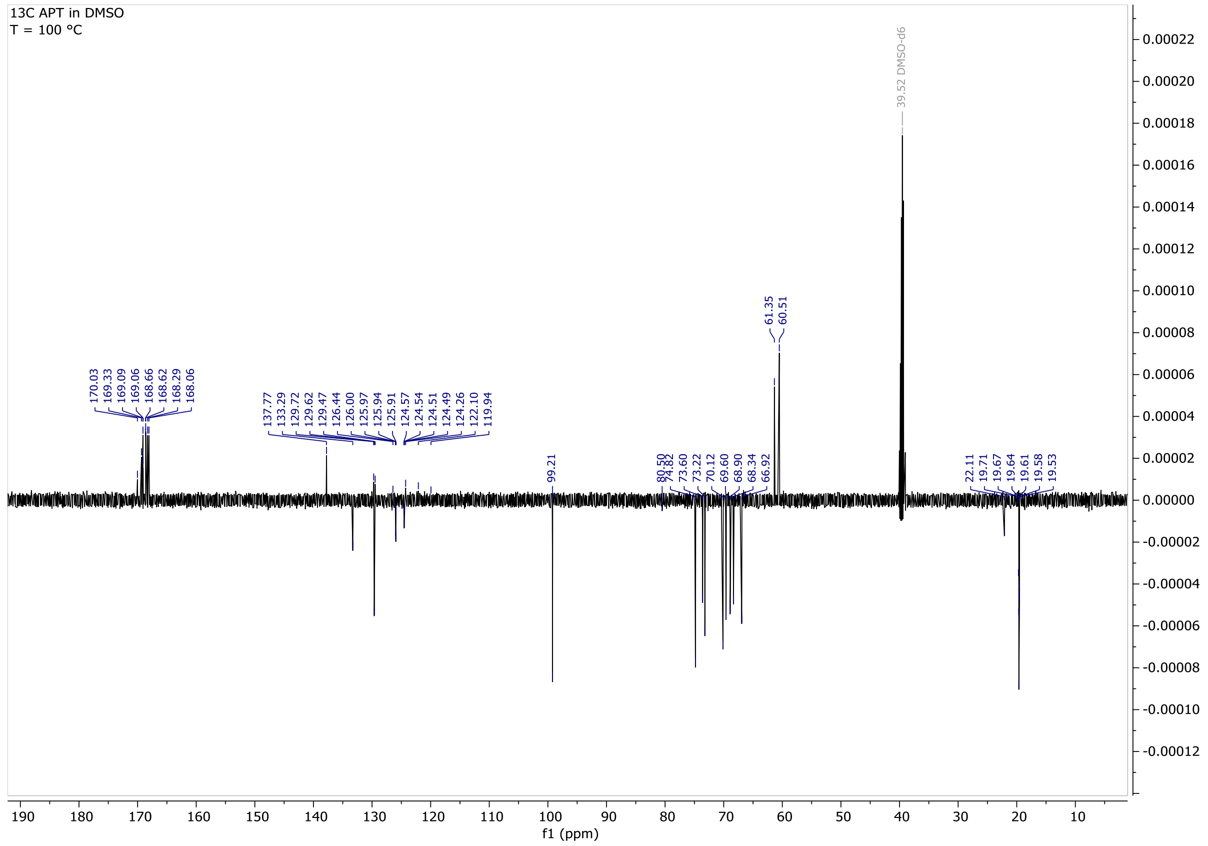

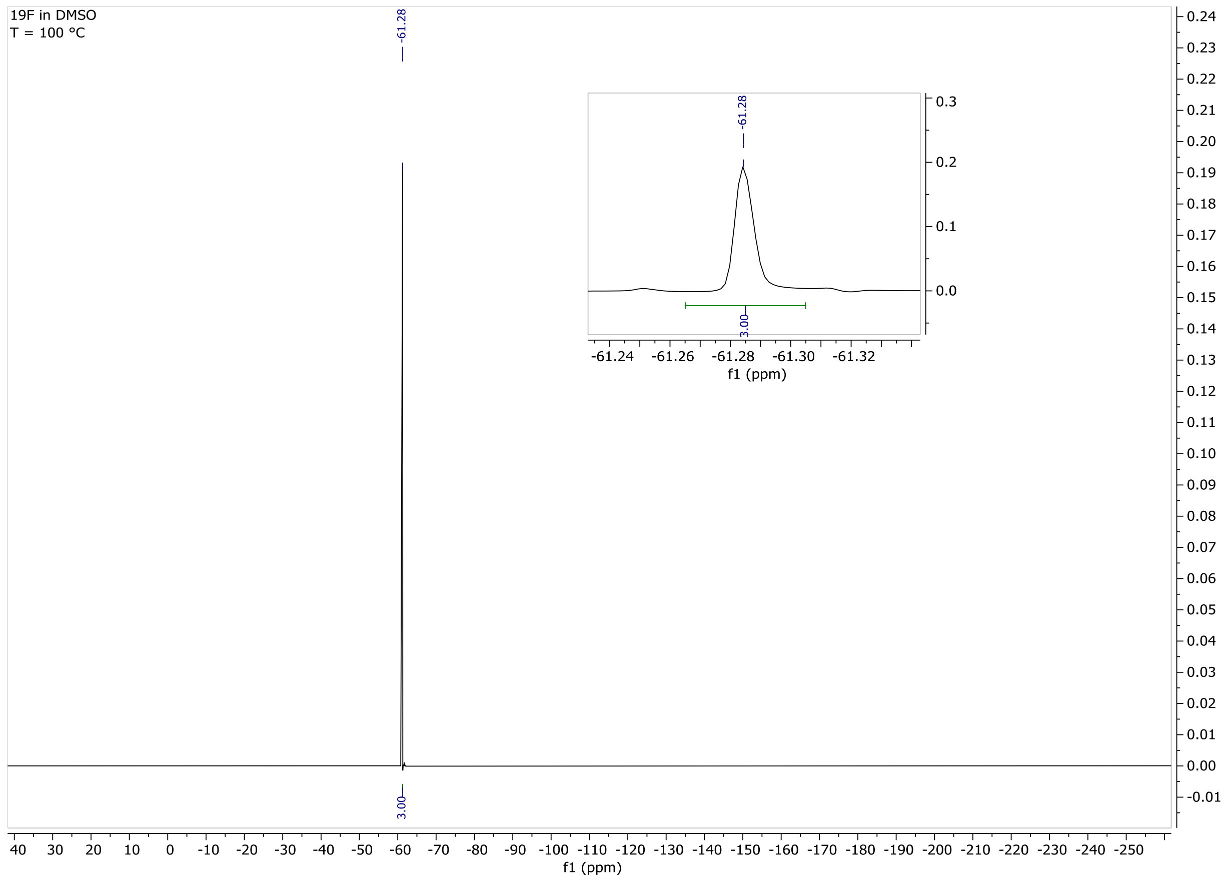
19F in DMSO
T = 100 °C
-61.28
3.00
f1 (ppm)

^1H, ^{13}C APT and ^{19}F NMR spectra of (2e)

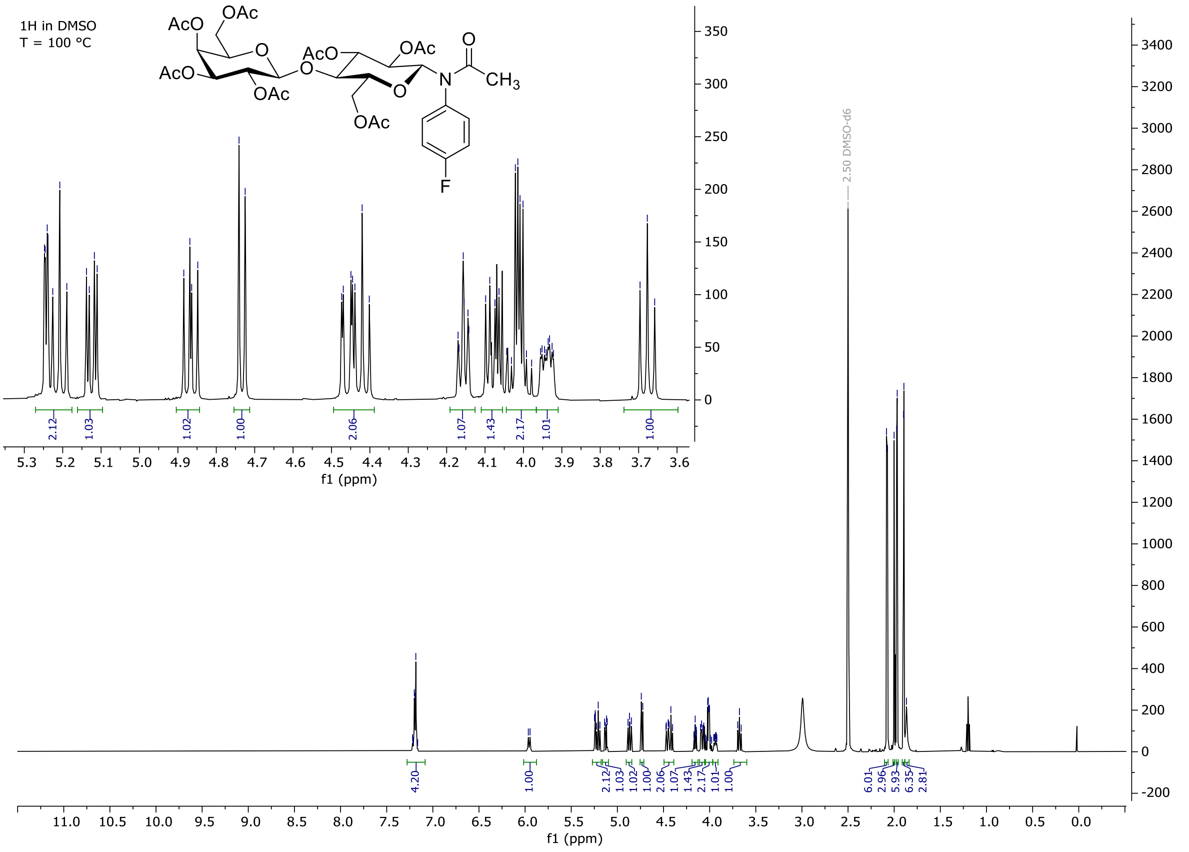

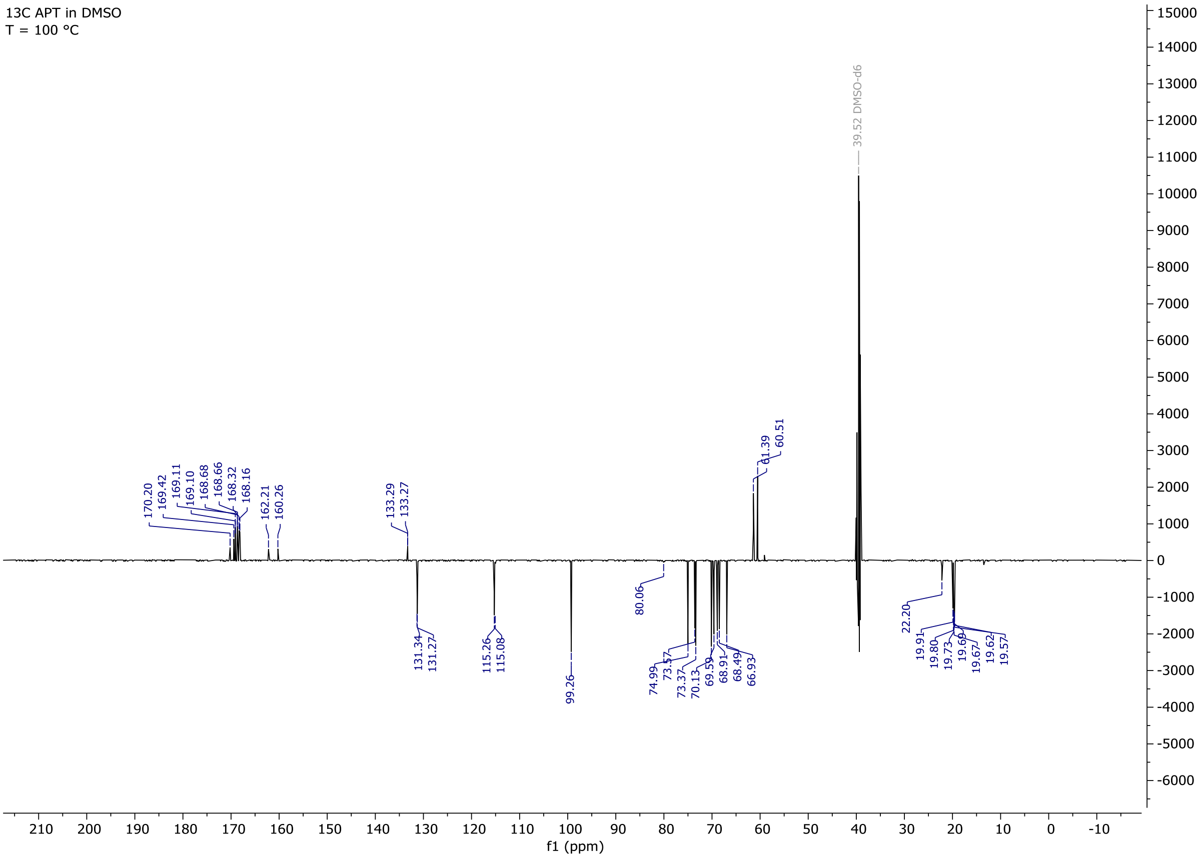

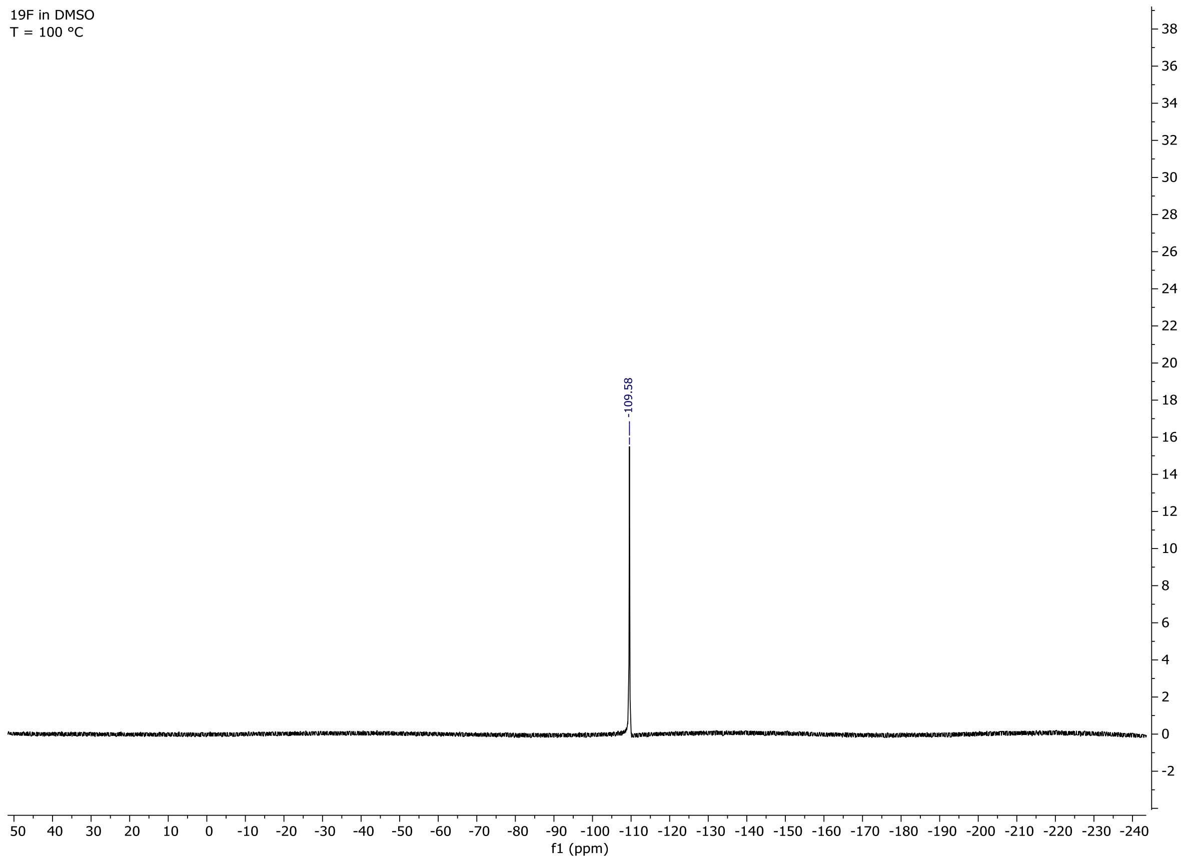
19F in DMSO
T = 100 °C
-109.58
f1 (ppm)

^{1}H, ^{13}C APT and ^{19}F NMR spectra of (2f)

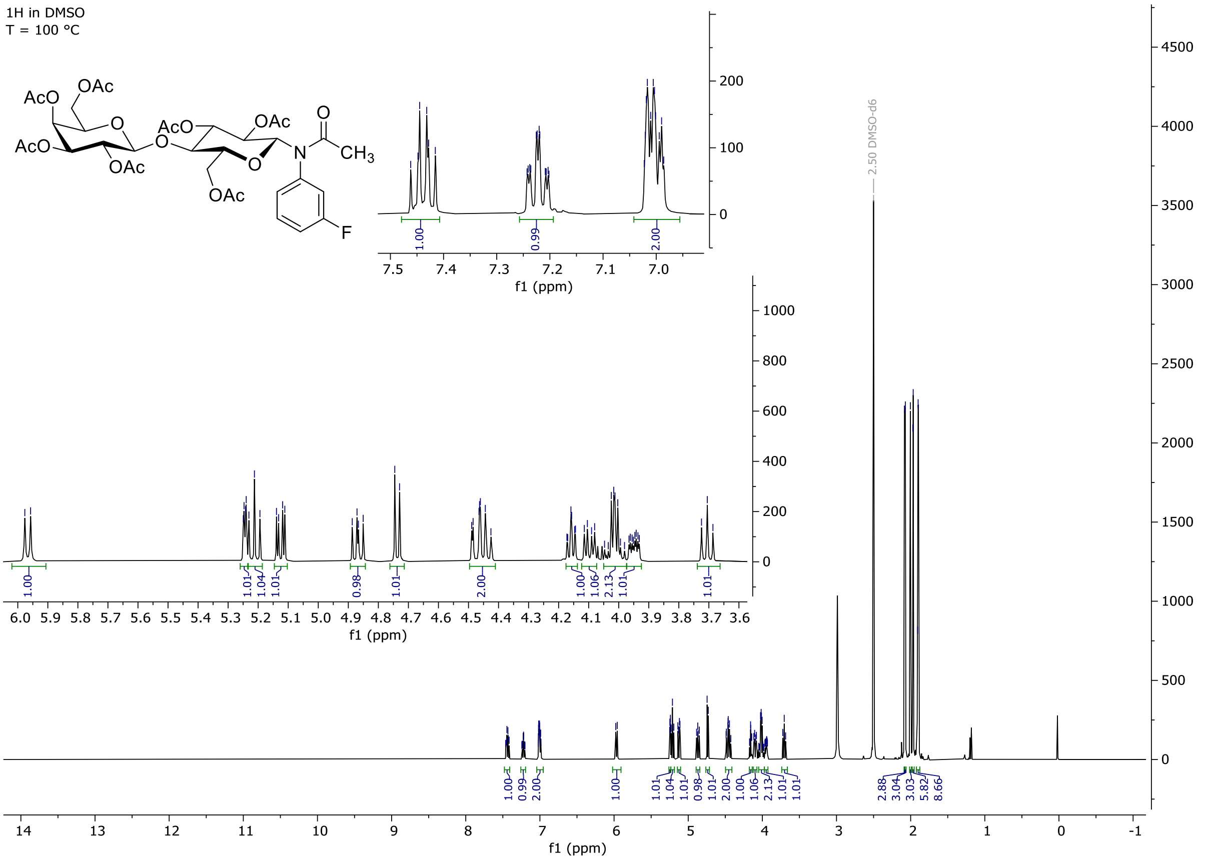

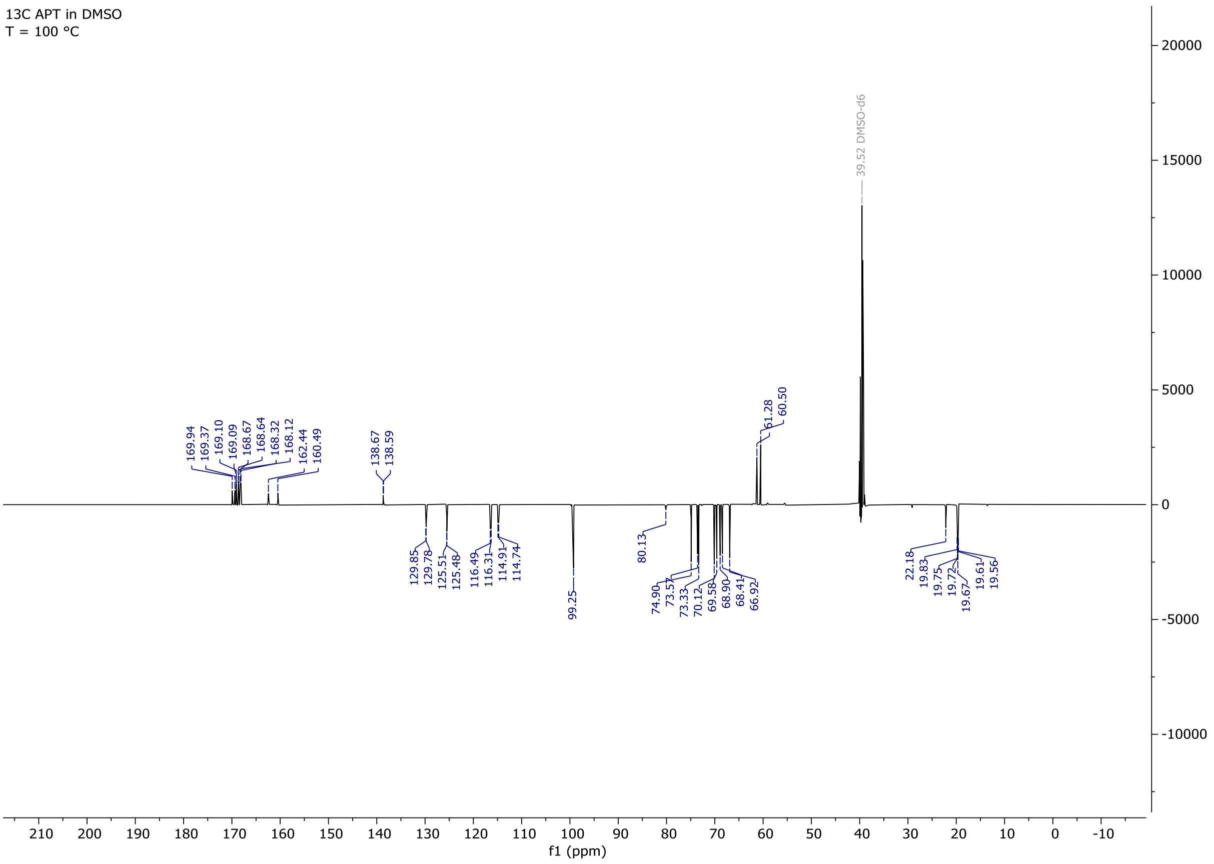

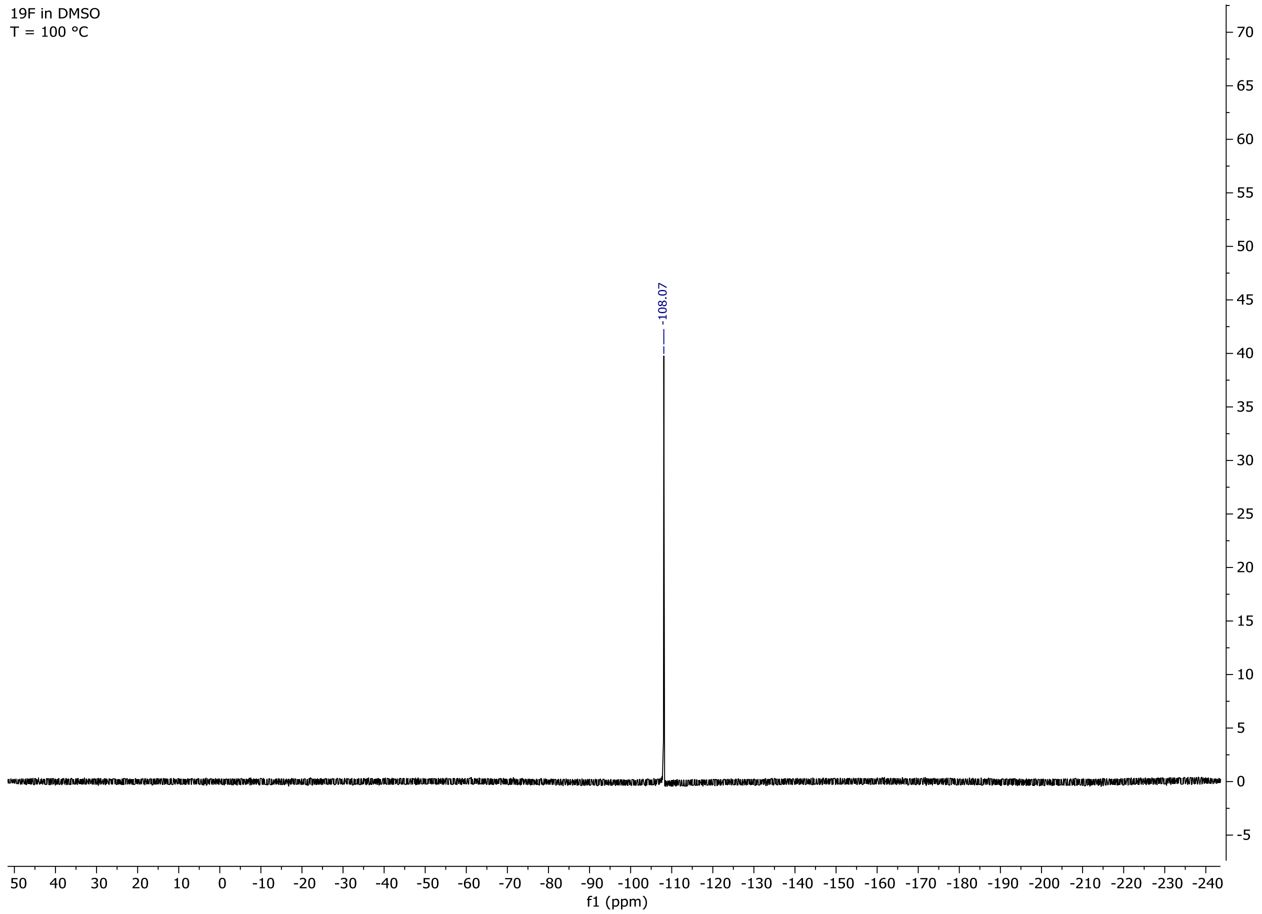
19F in DMSO
T = 100 °C
-108.07
f1 (ppm)

^1H, ^{13}C APT and ^{19}F NMR spectra of (2g)

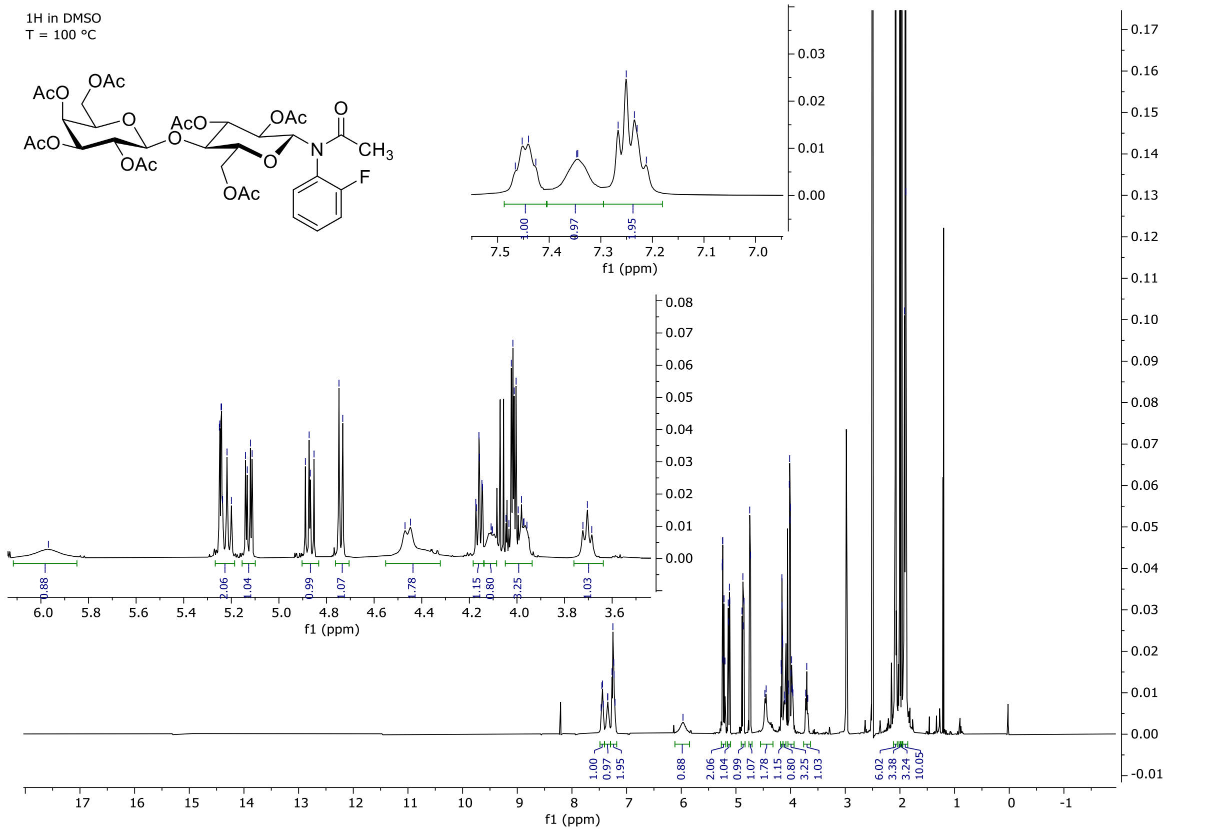

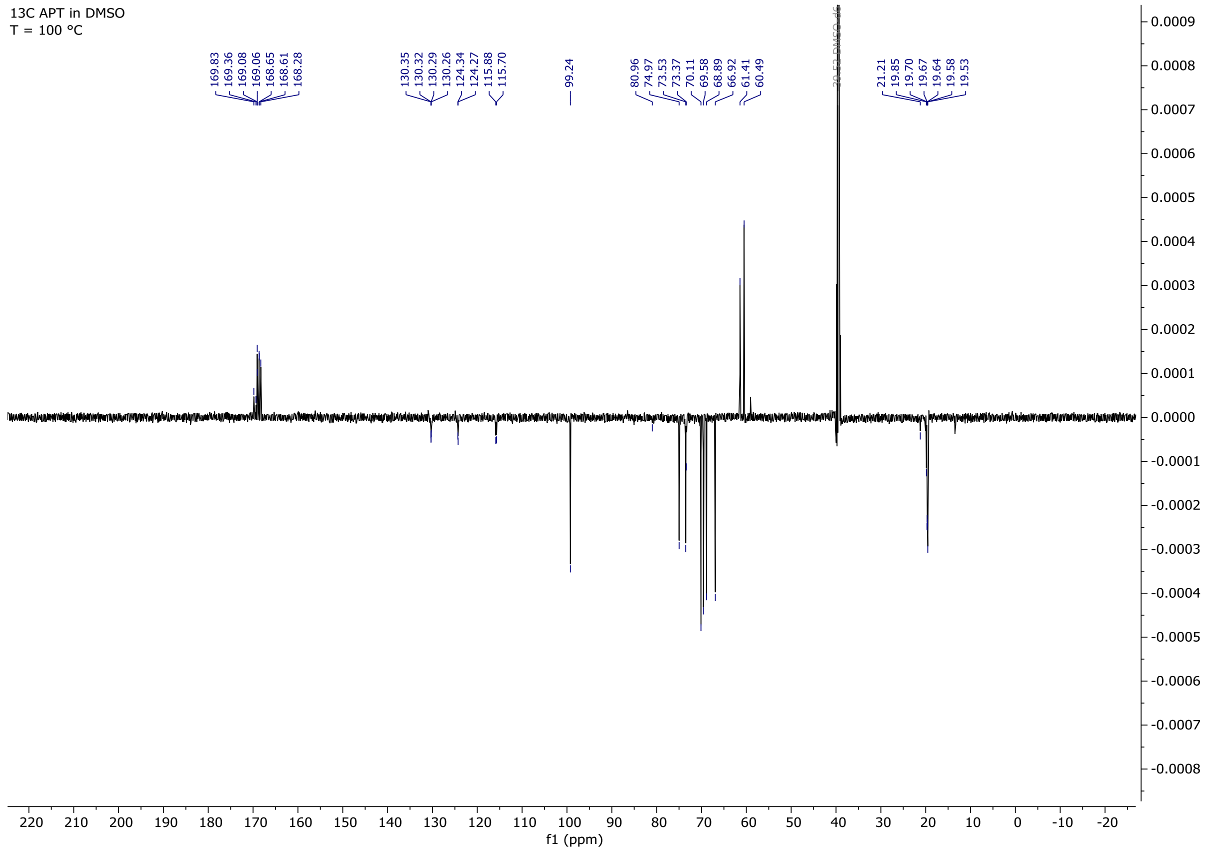

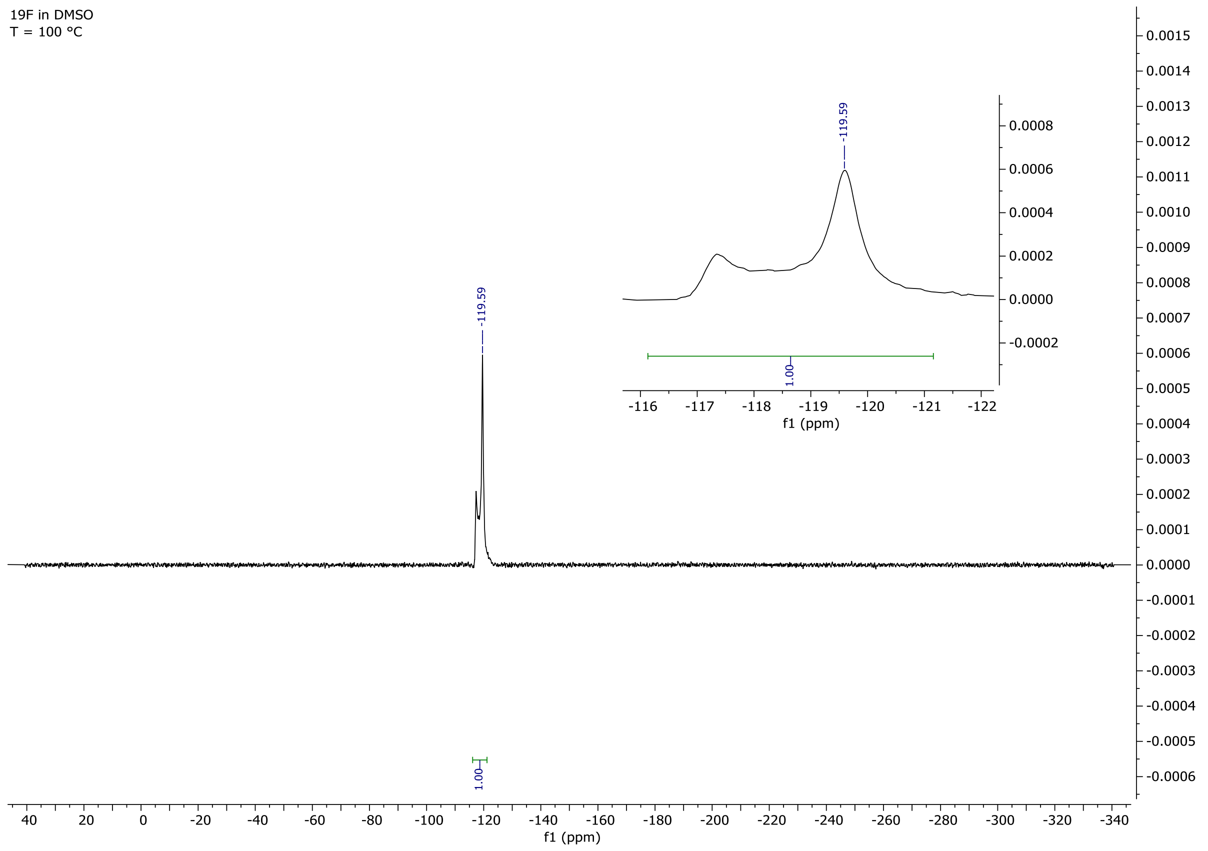

19F in DMSO
T = 100 °C
-119.59
1.00
f1 (ppm)

^{1}H, ^{13}C APT and ^{19}F NMR spectra of (2h)

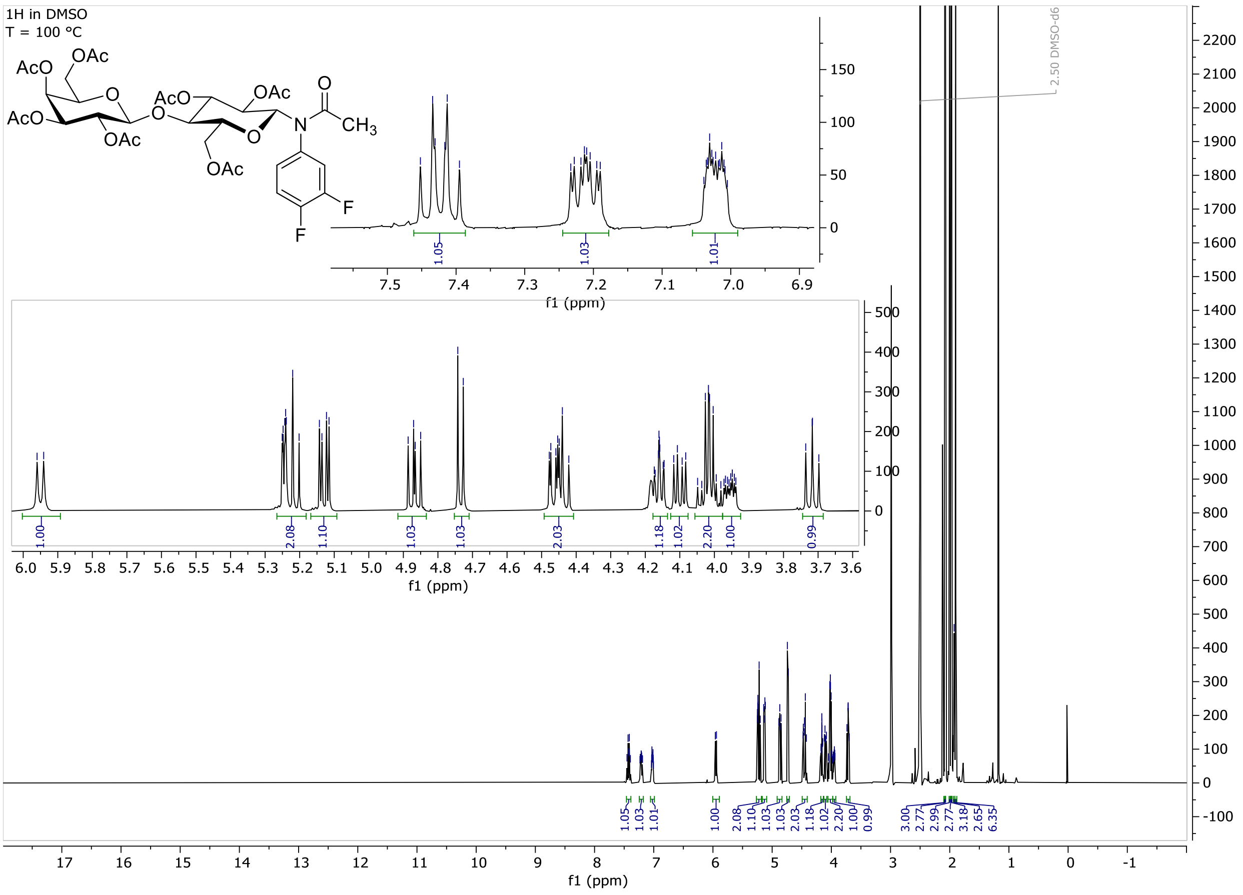

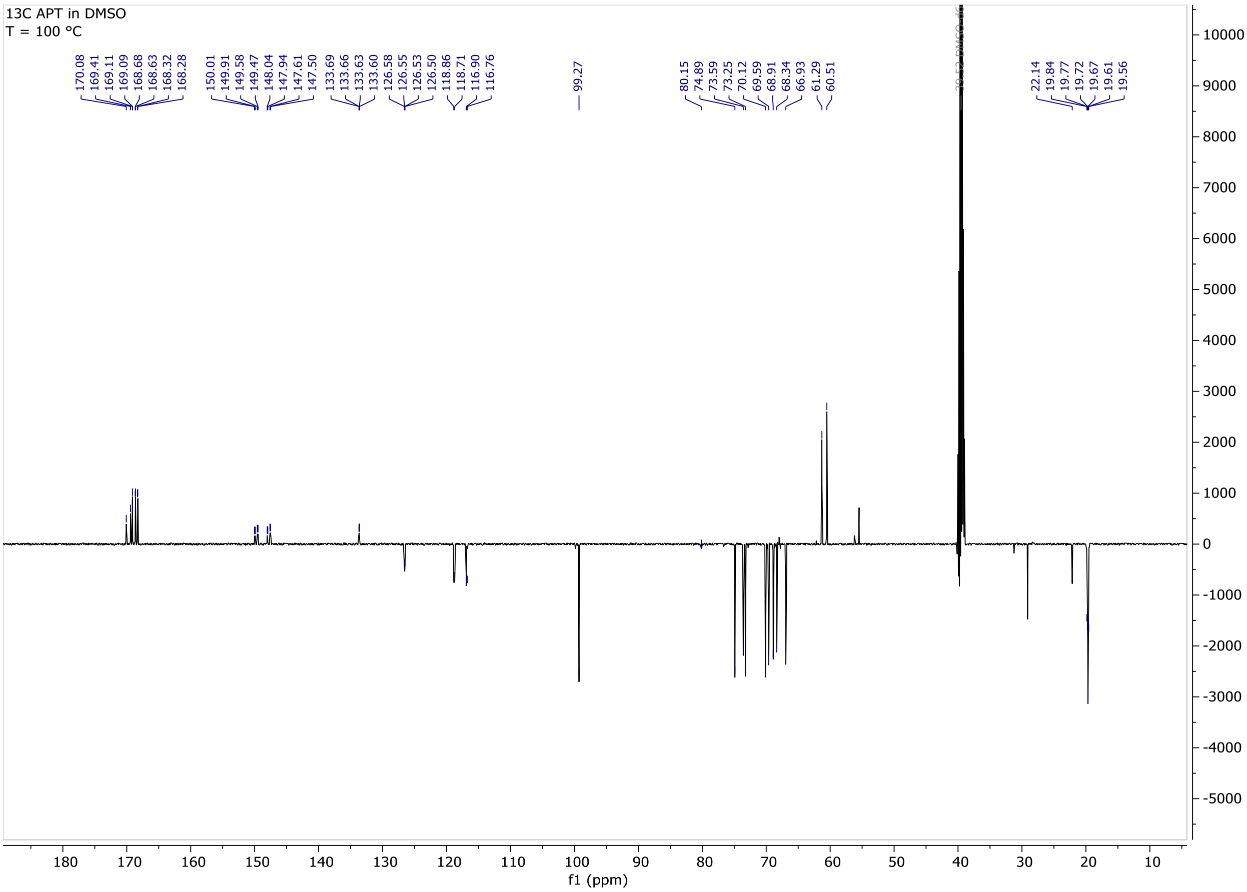

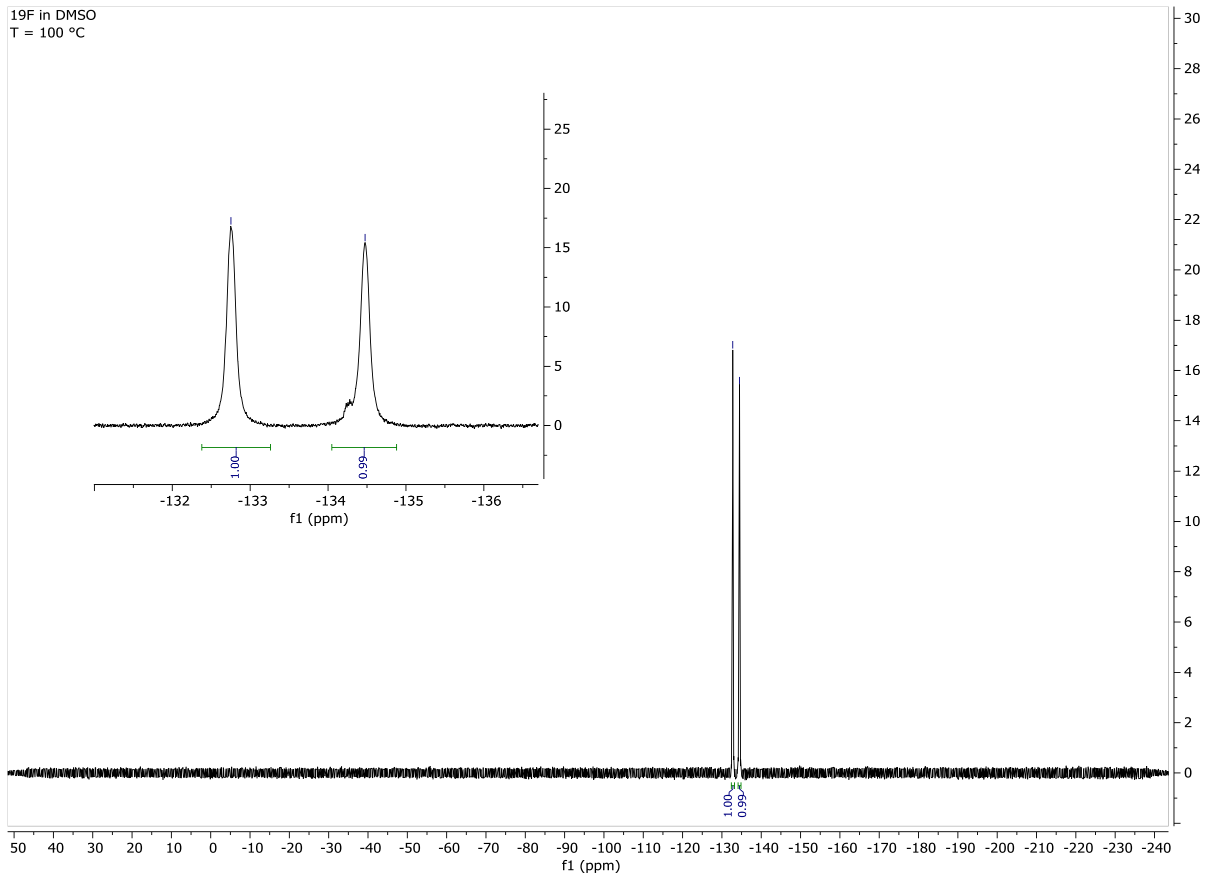
19F in DMSO
T = 100 °C
f1 (ppm)
f1 (ppm)
1.00
0.99

^{1}H, ^{13}C APT and ^{19}F NMR spectra of (2i)

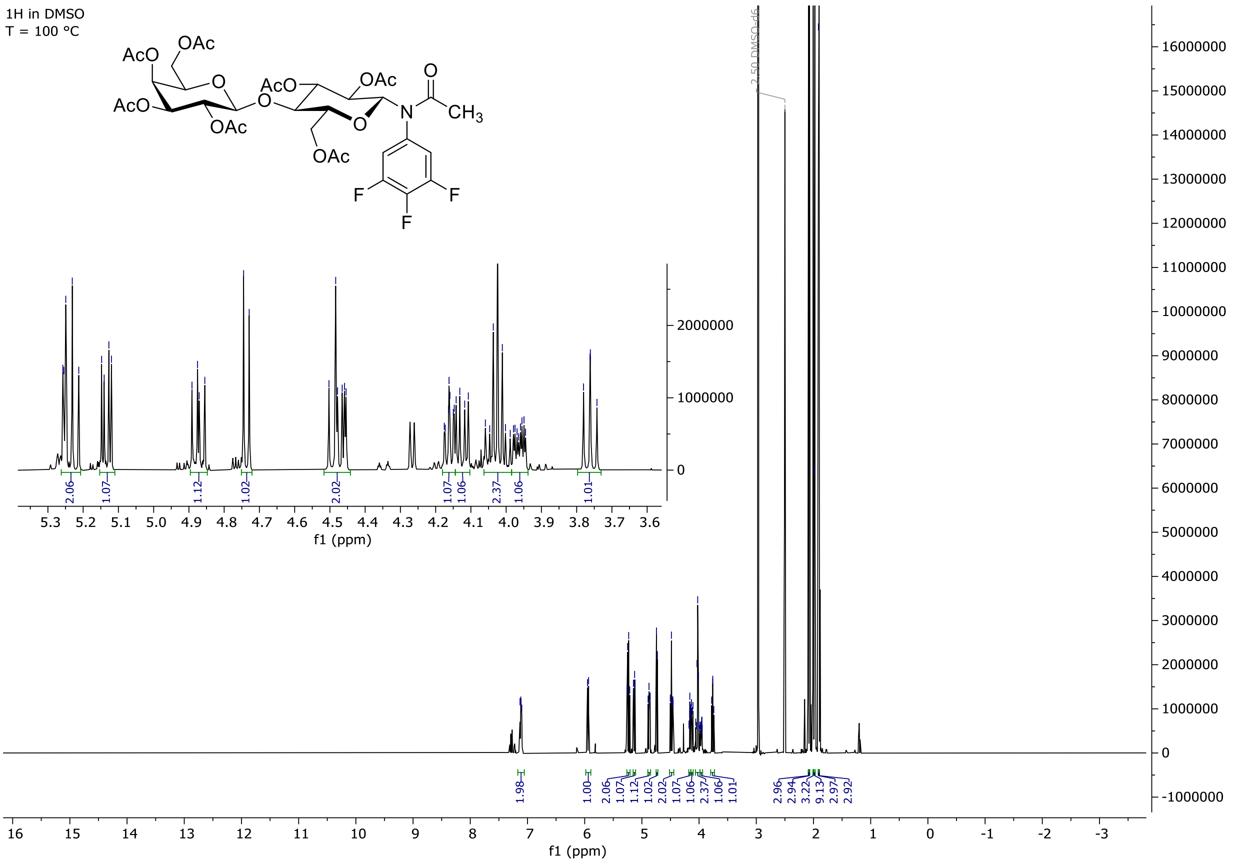

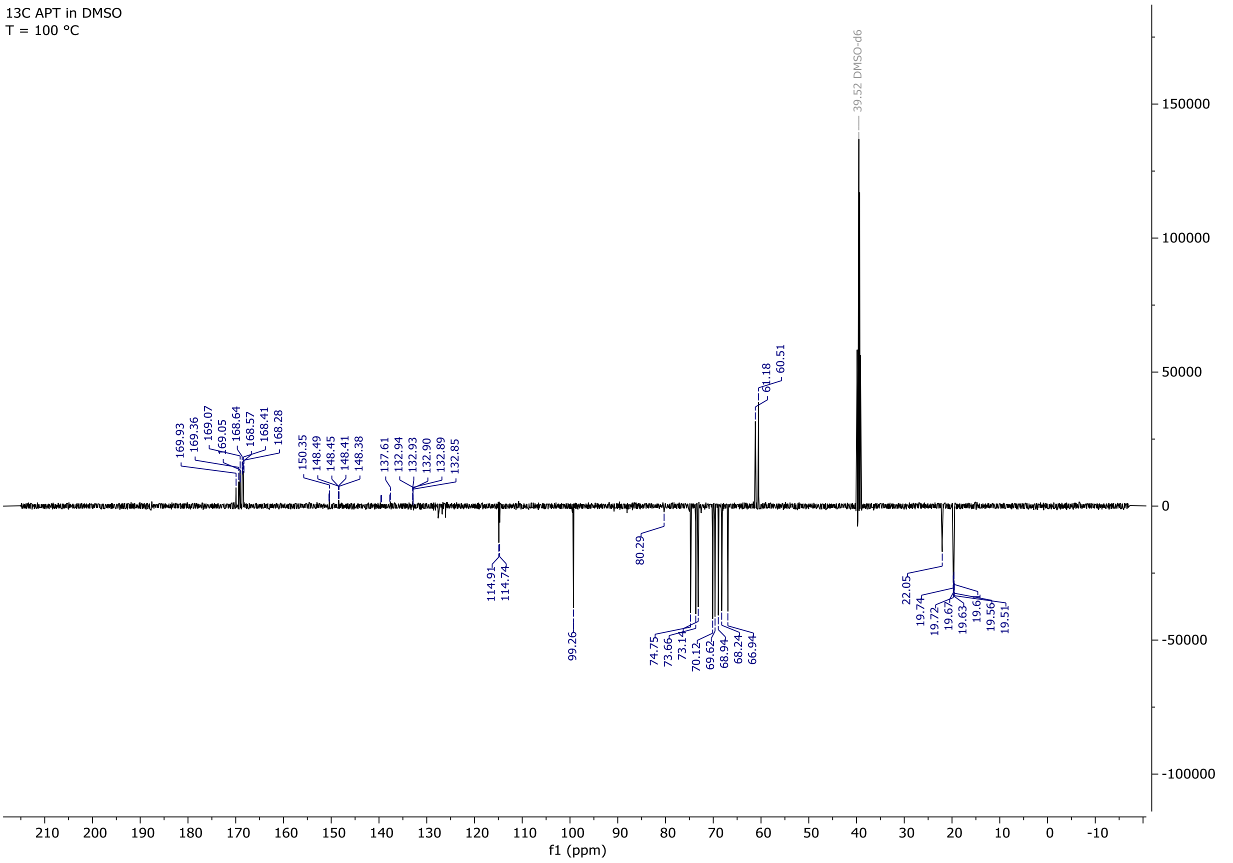

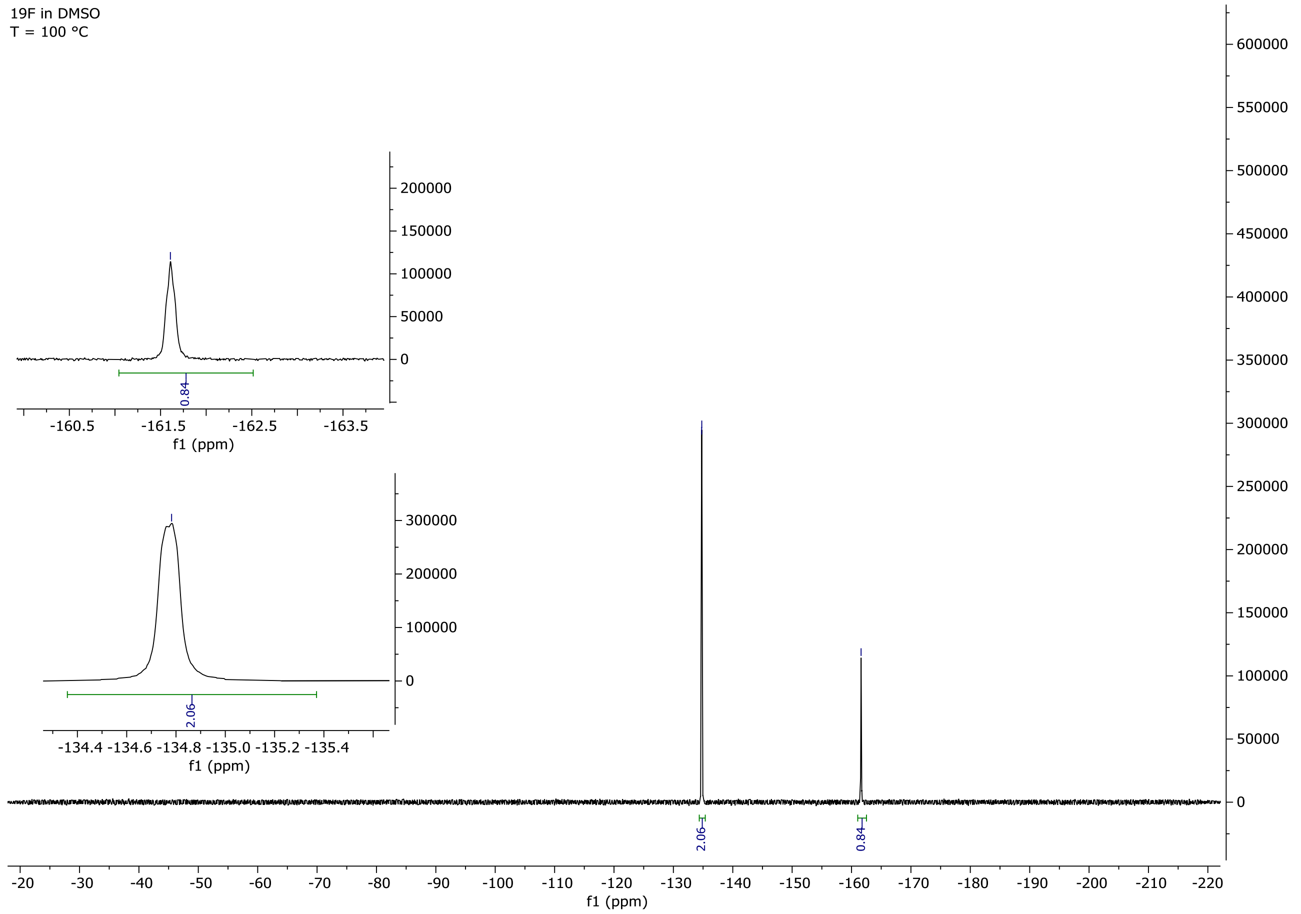

19F in DMSO
T = 100 °C
0.84
-160.5
-161.5
-162.5
-163.5
f1 (ppm)
2.06
-134.4 -134.6 -134.8 -135.0 -135.2 -135.4
f1 (ppm)
2.06
0.84
f1 (ppm)

^{1}H and ^{13}C APT NMR spectra of (2j)

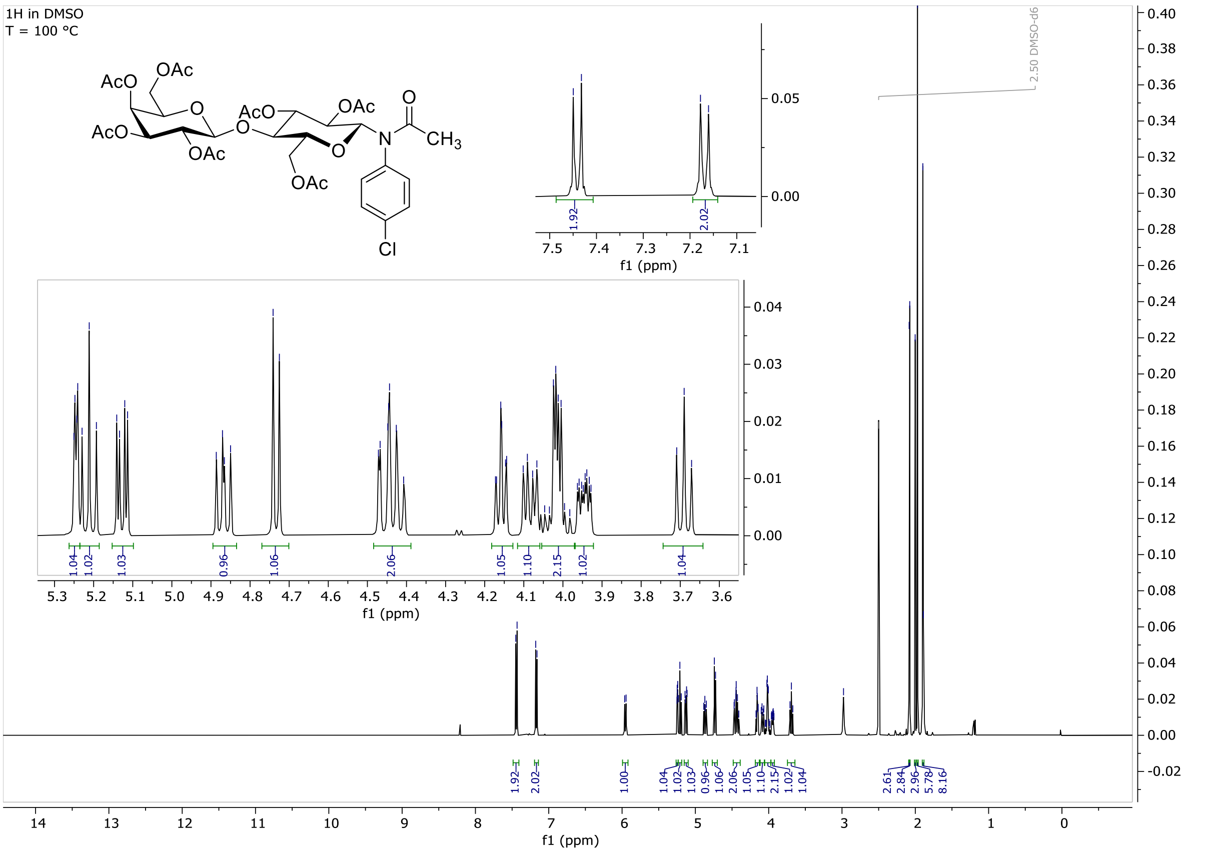

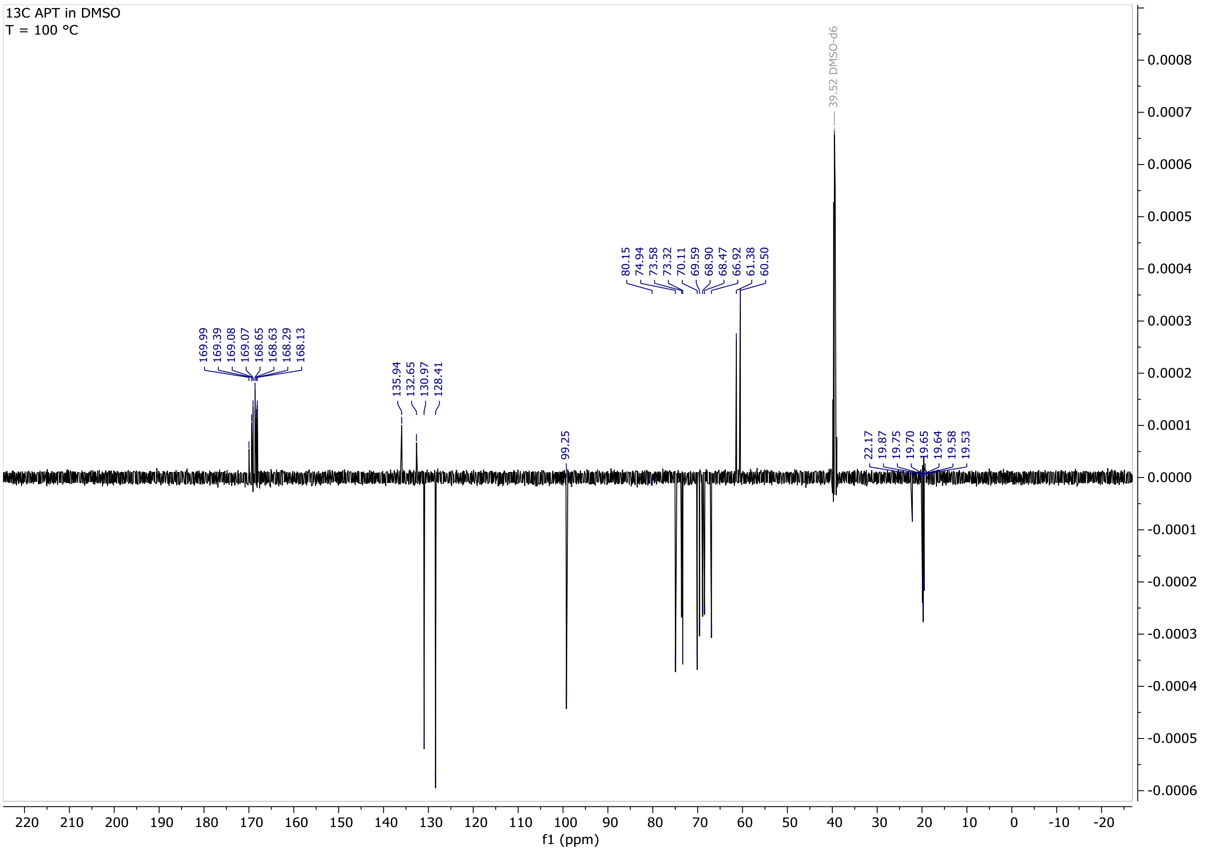

^1H and ^{13}C APT NMR spectra of (2k)

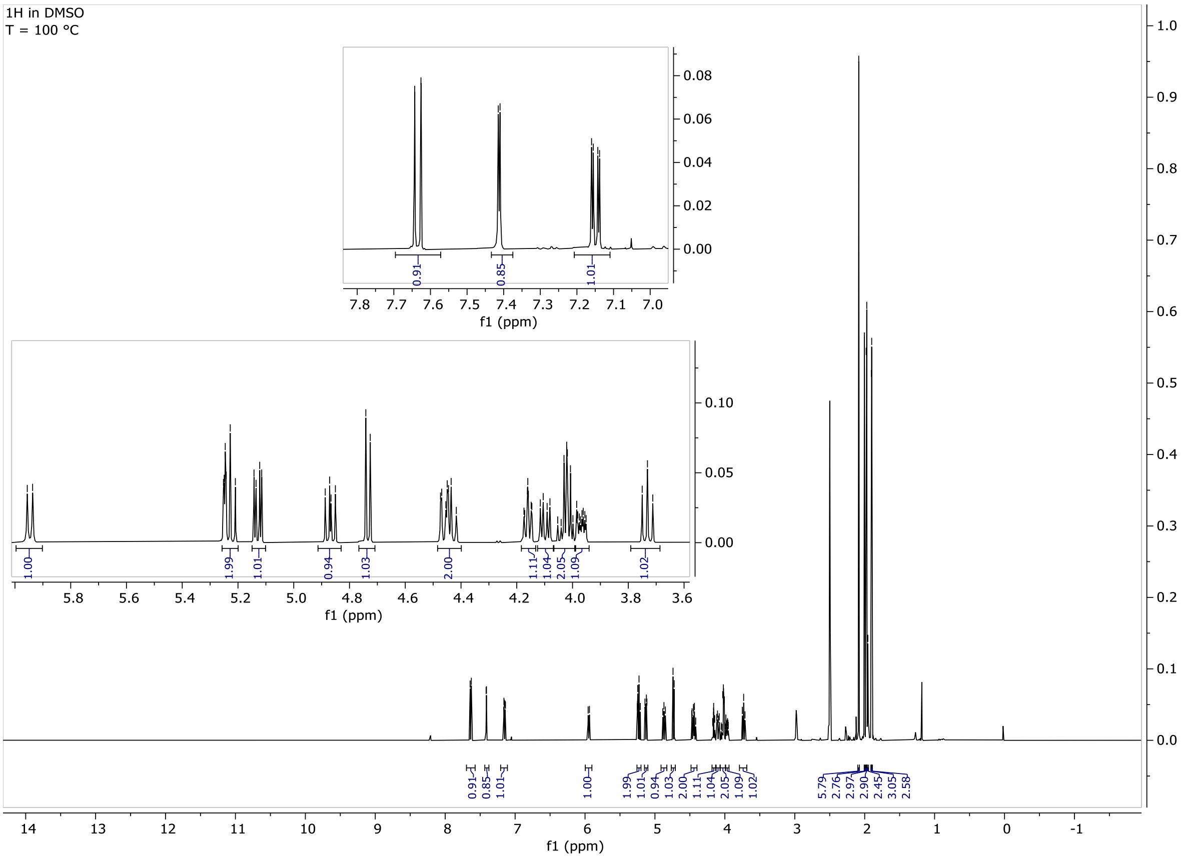

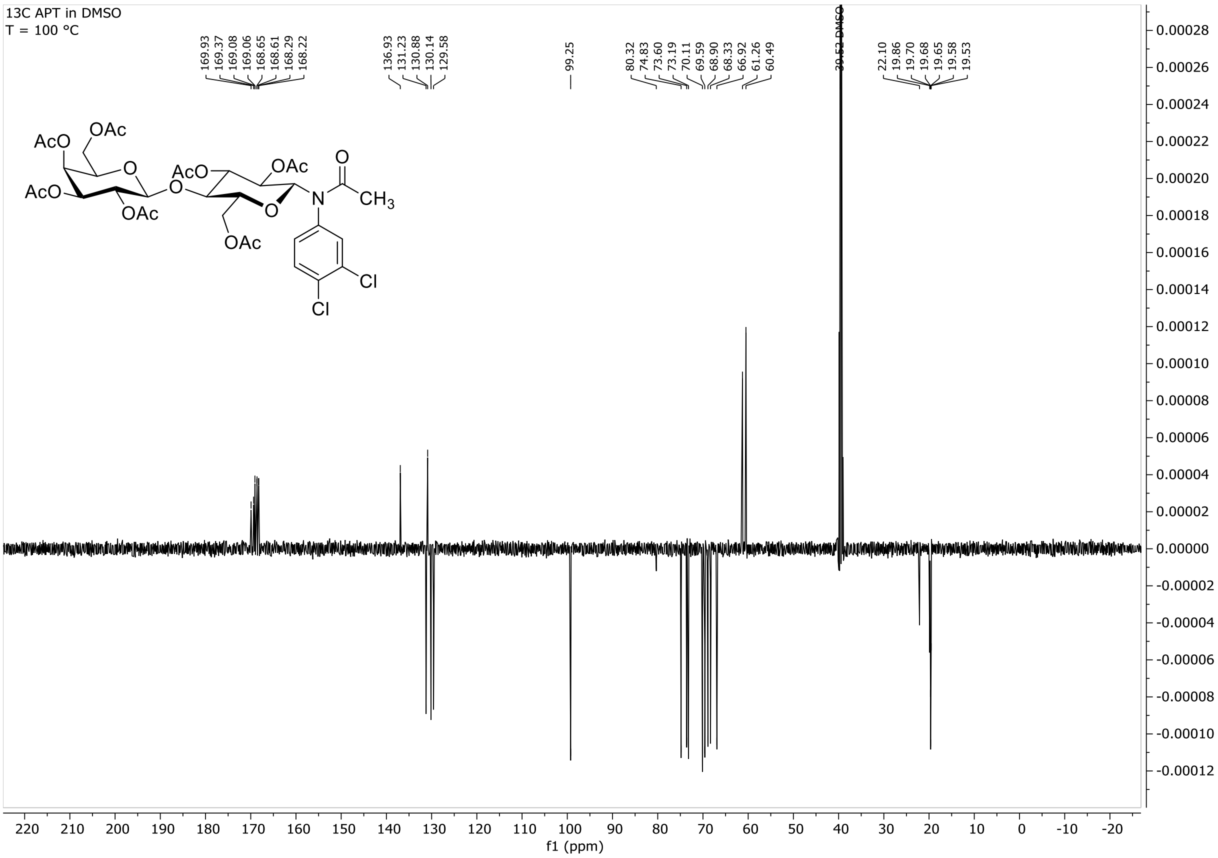

^{1}H and ^{13}C APT NMR spectra of (2l)

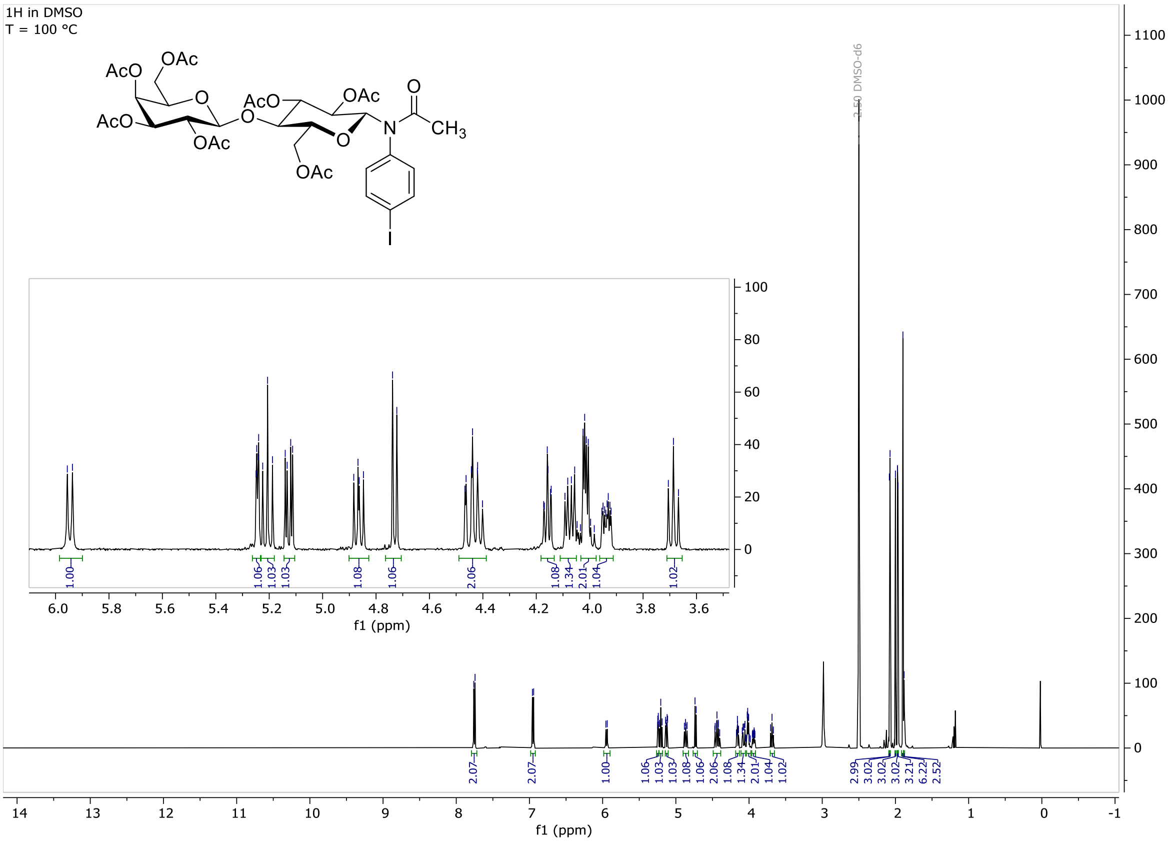

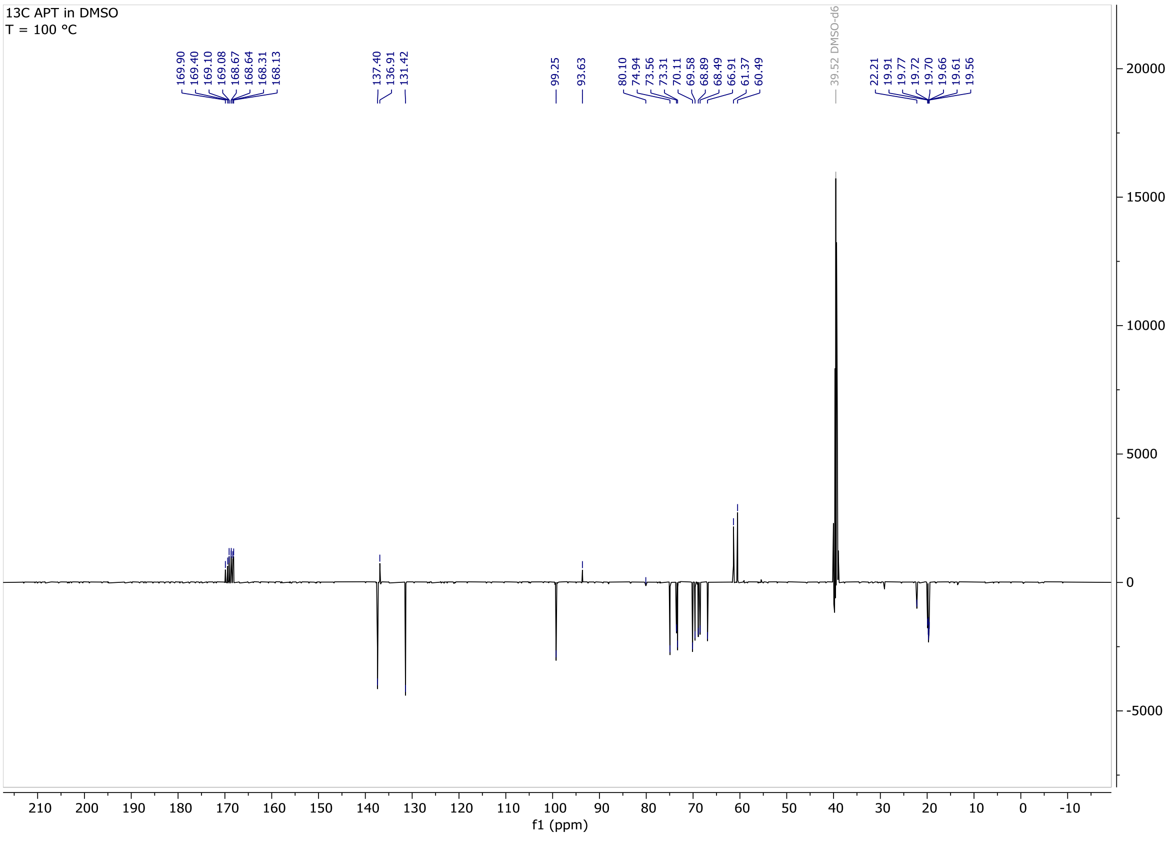

^{1}H and ^{13}C APT NMR spectra of (2m)

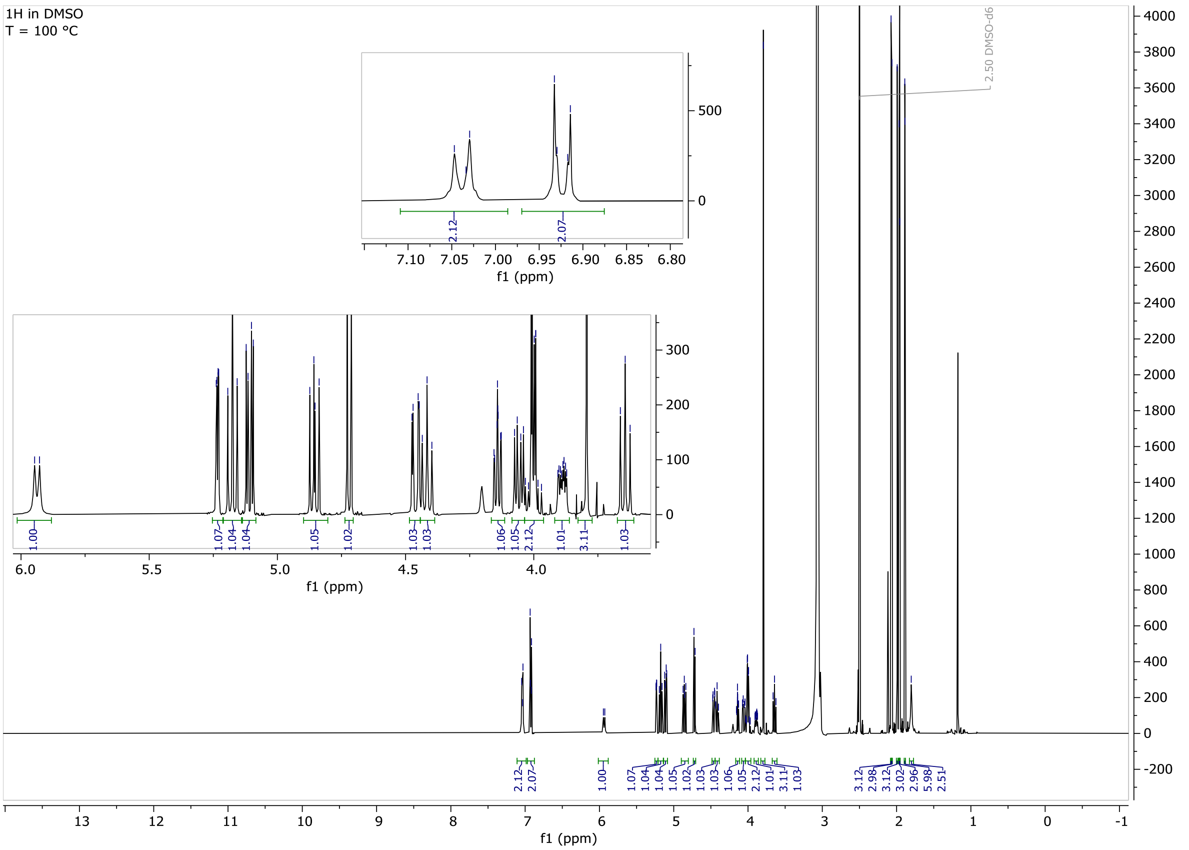

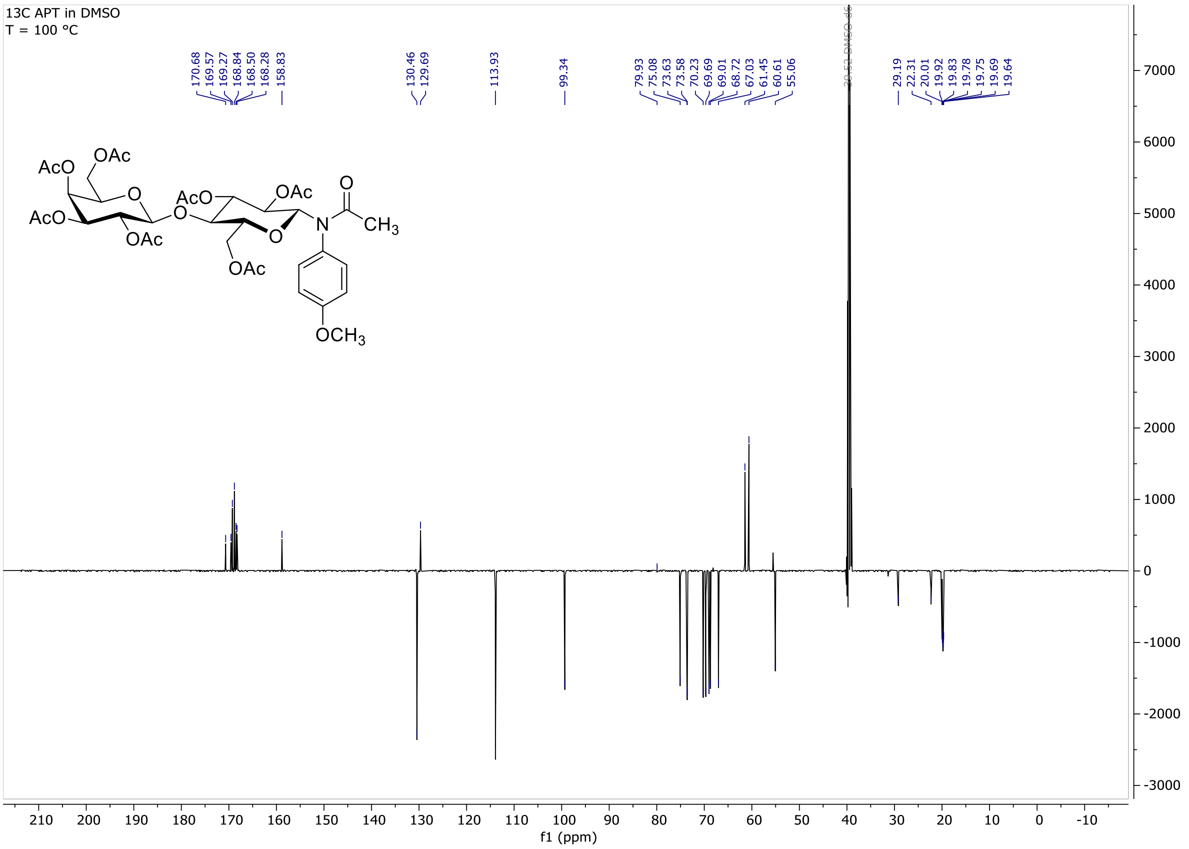

^1H and ^{13}C APT NMR spectra of (2n)

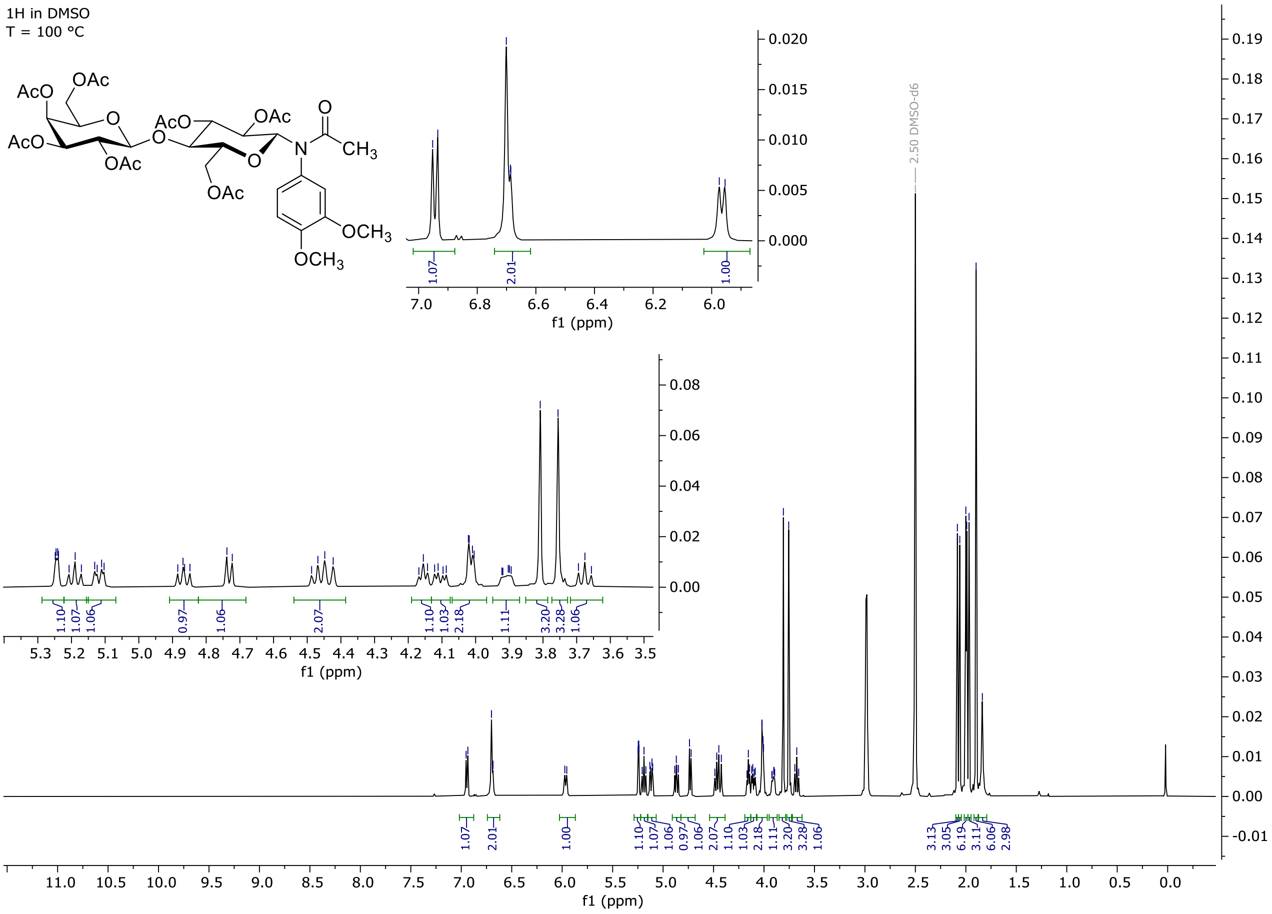

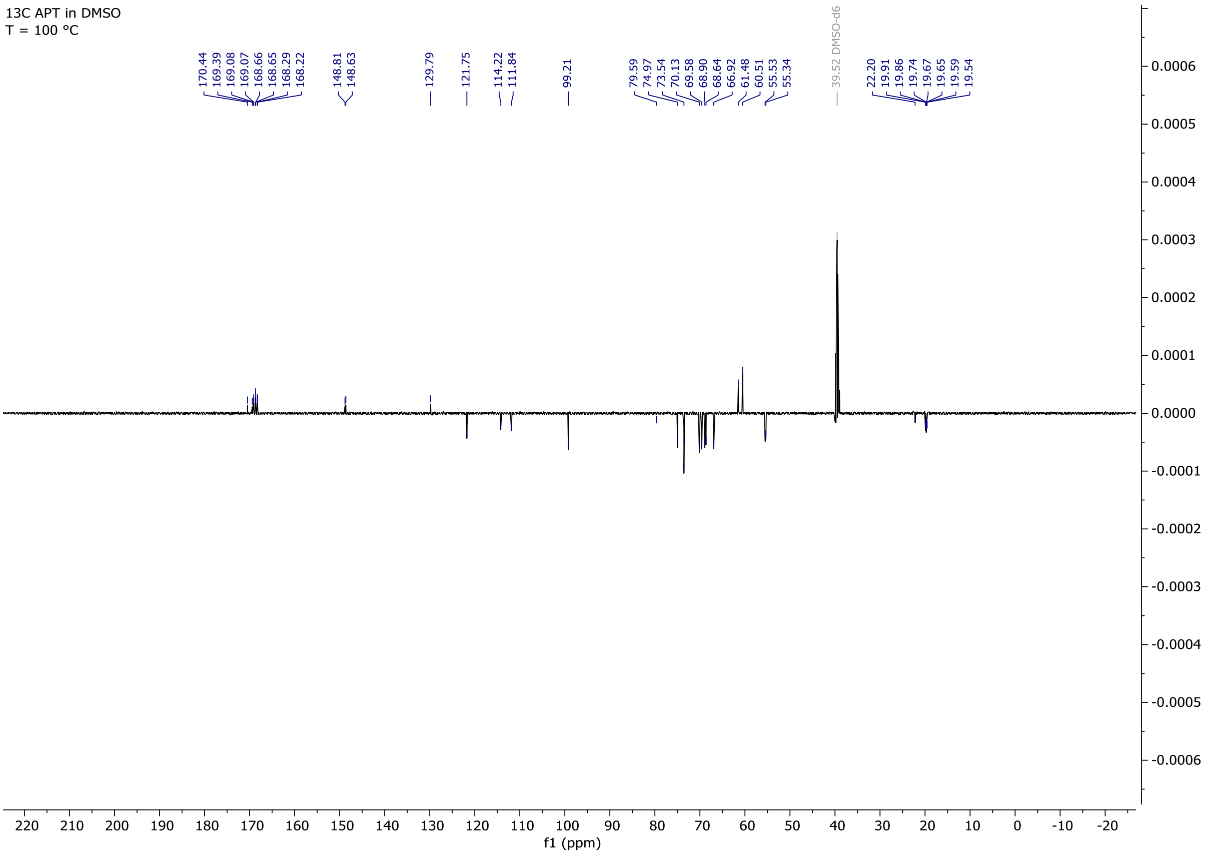

^{1}H and ^{13}C APT NMR spectra of (2o)

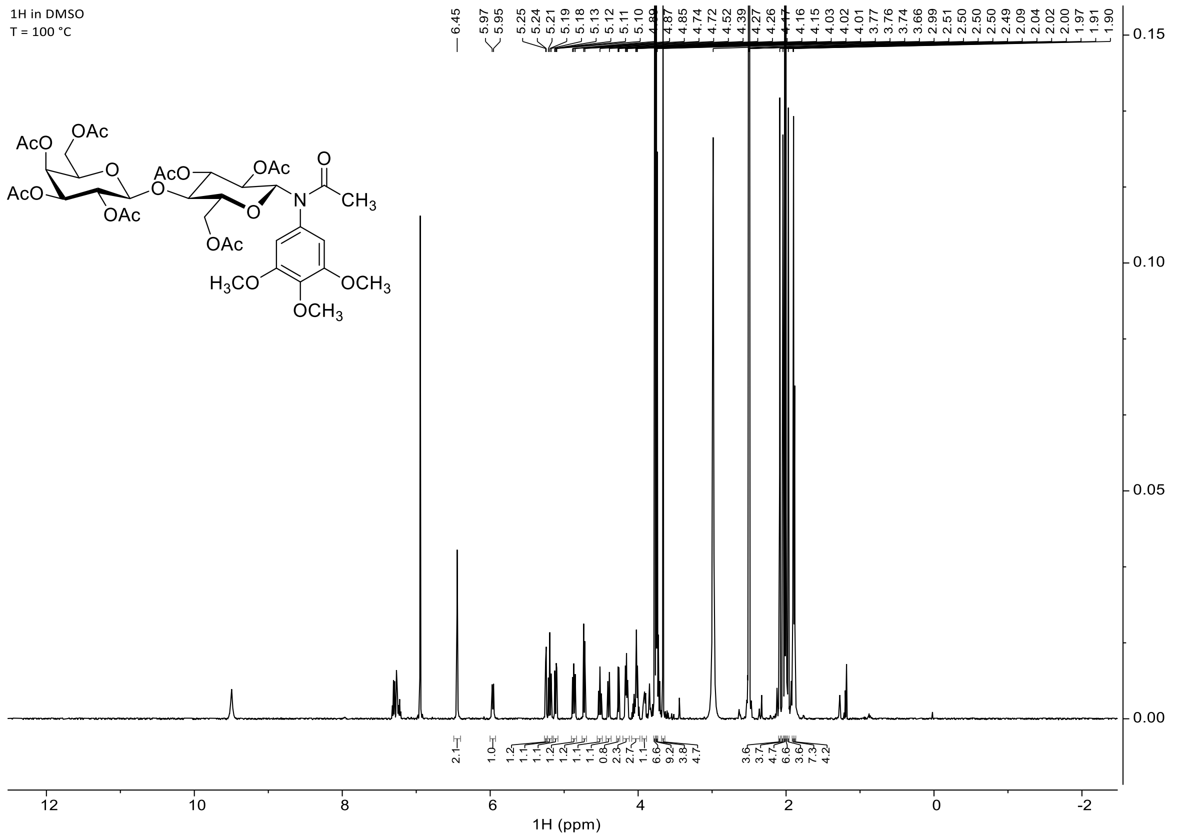

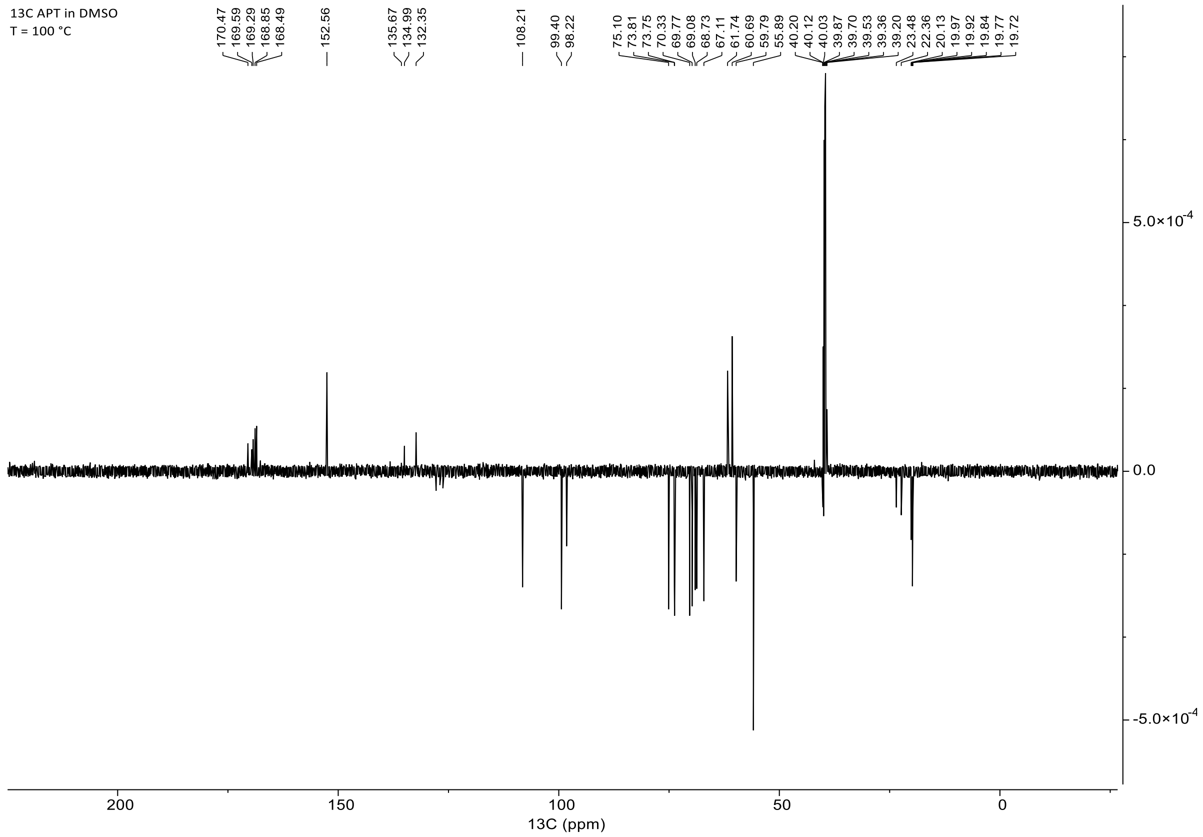

^{1}H and ^{13}C APT NMR spectra of (2q)

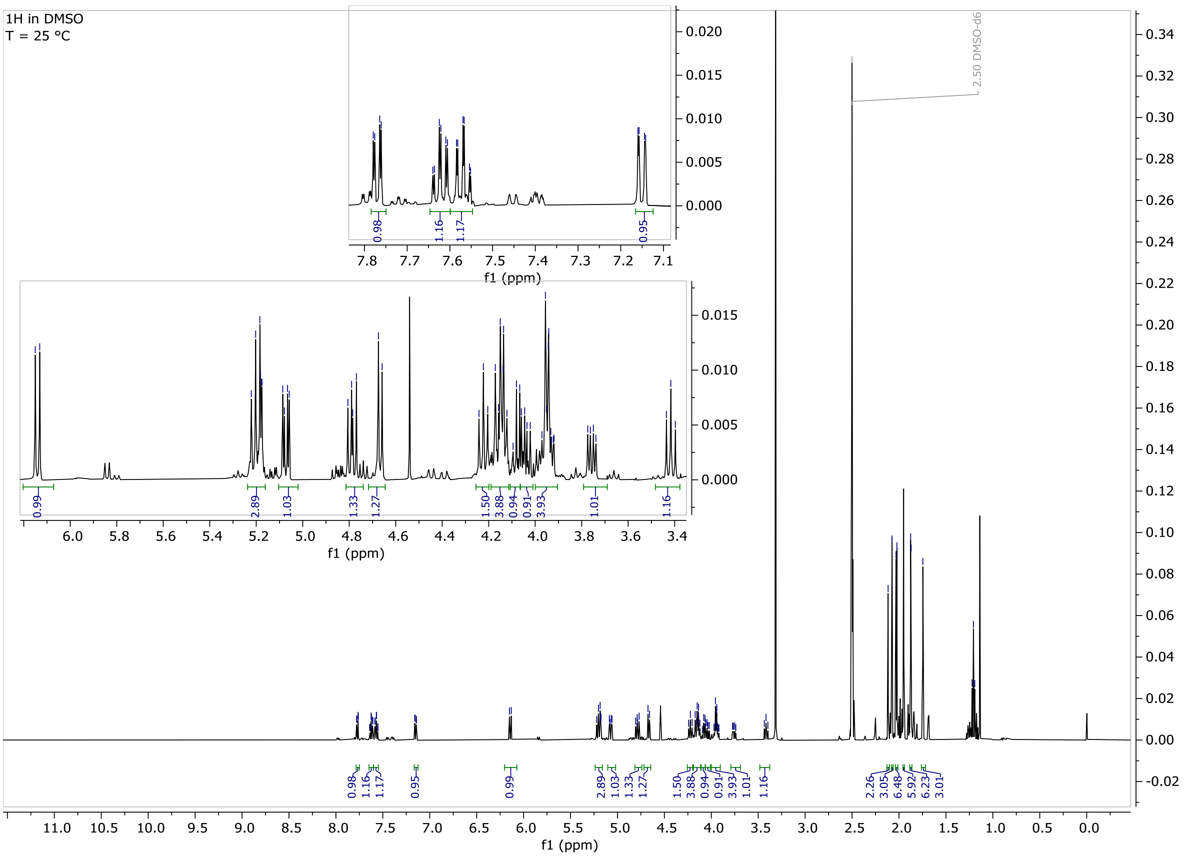

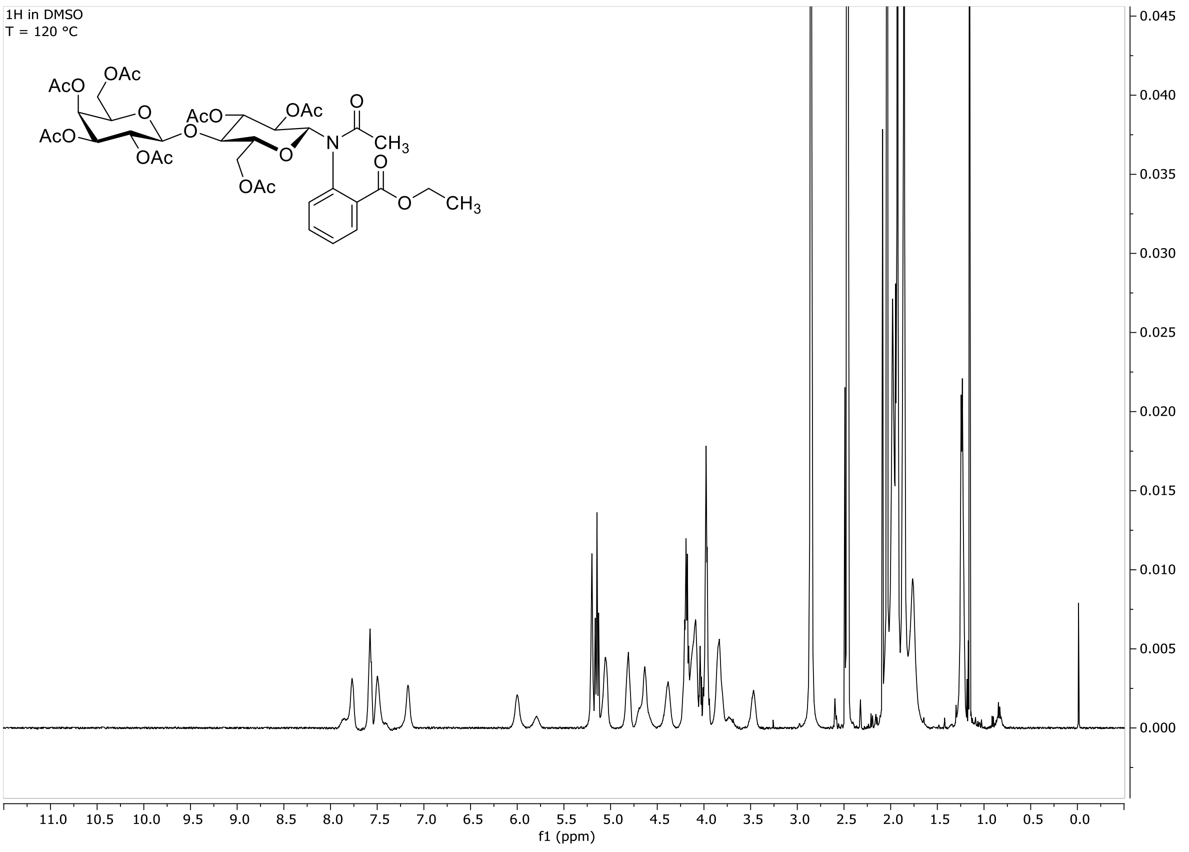

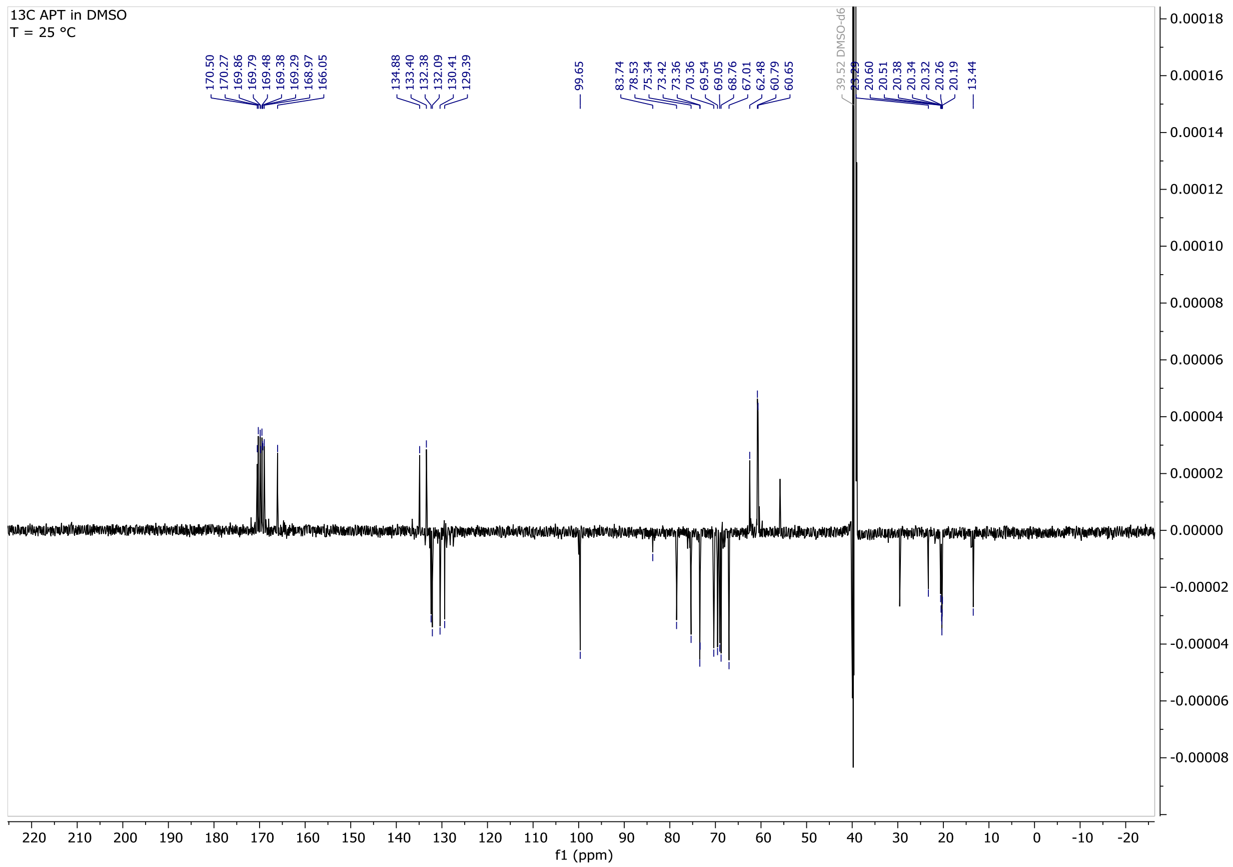
13C APT in DMSO
T = 25 °C
170.50
170.27
169.86
169.79
169.48
169.38
169.29
168.97
166.05
134.88
133.40
132.38
132.09
130.41
129.39
99.65
83.74
78.53
75.34
73.42
73.36
70.36
69.54
69.05
68.76
67.01
62.48
60.79
60.65
39.52 DMSO-d6
23.29
20.60
20.51
20.38
20.34
20.32
20.26
20.19
13.44
f1 (ppm)

^{1}H and ^{13}C APT NMR spectra of (2r)

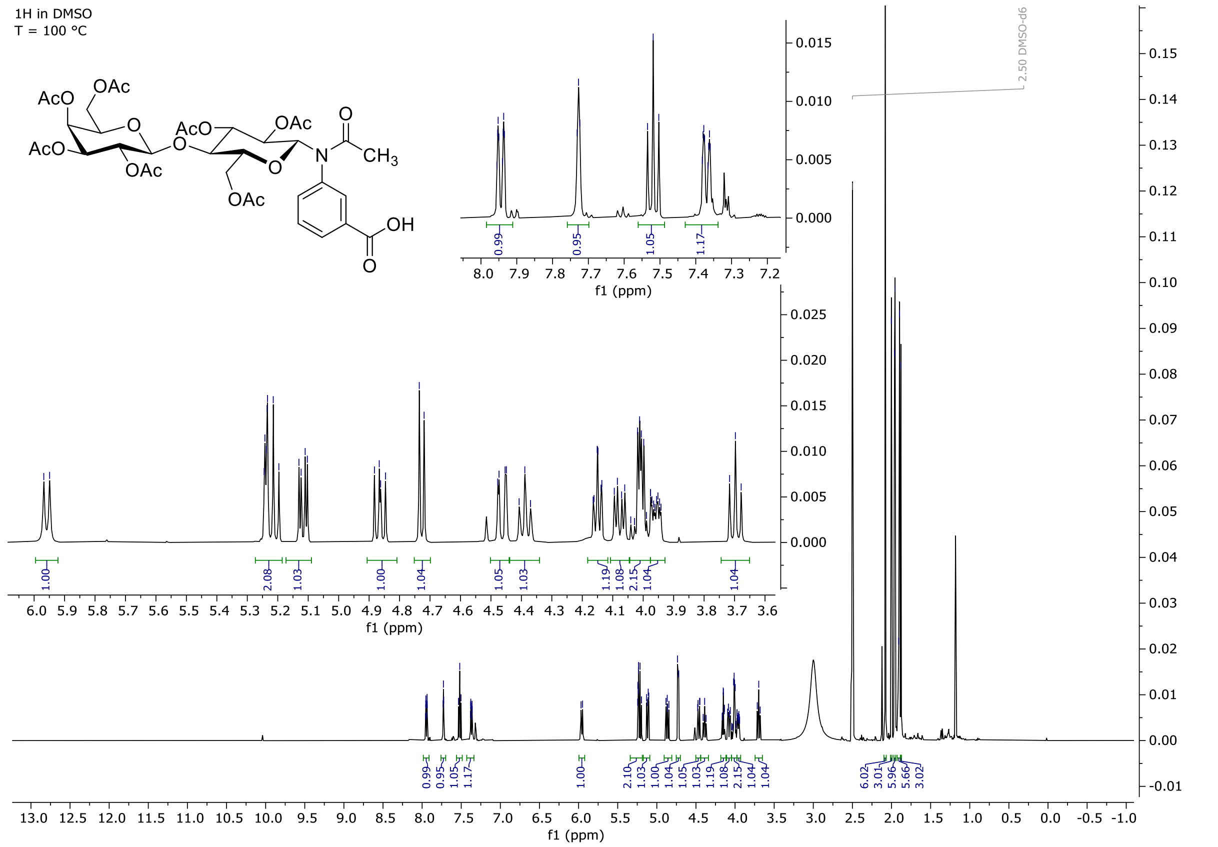

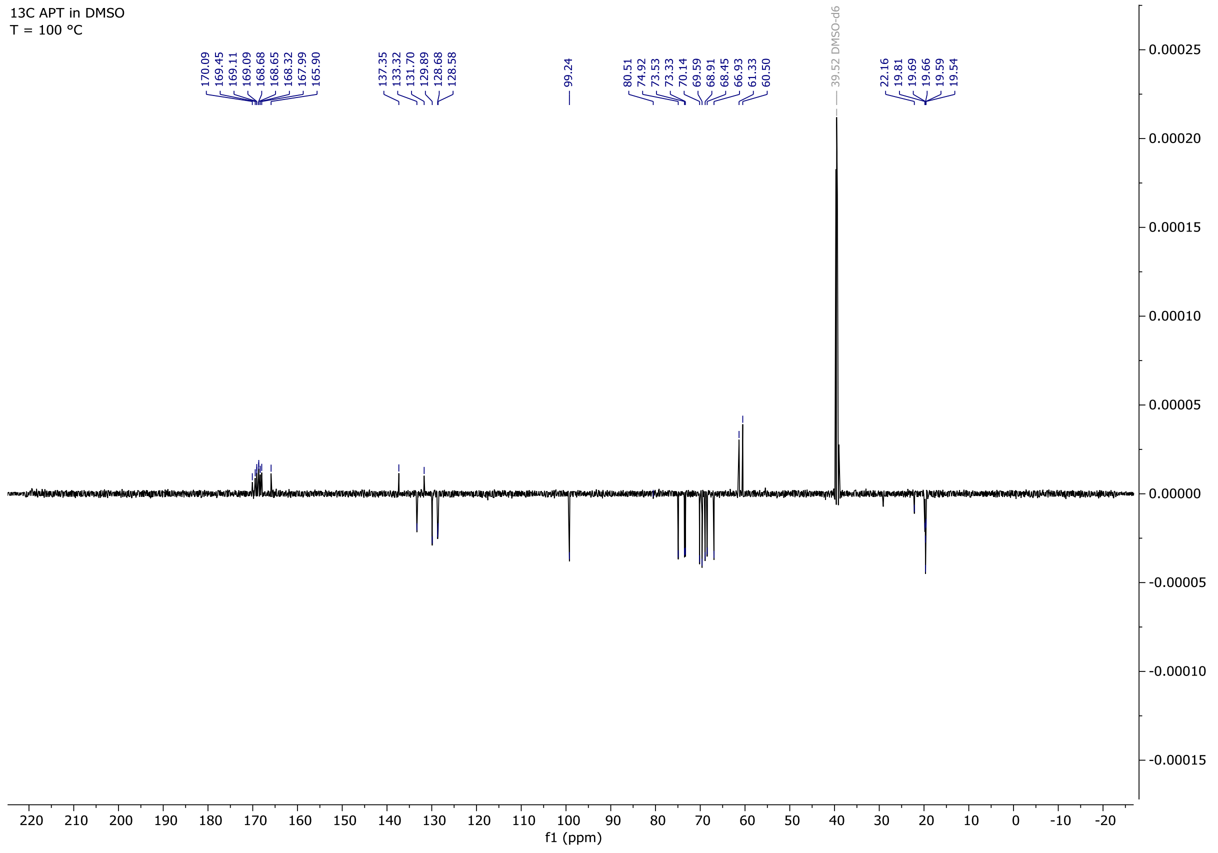

^{1}H and ^{13}C APT NMR spectra of (S1)

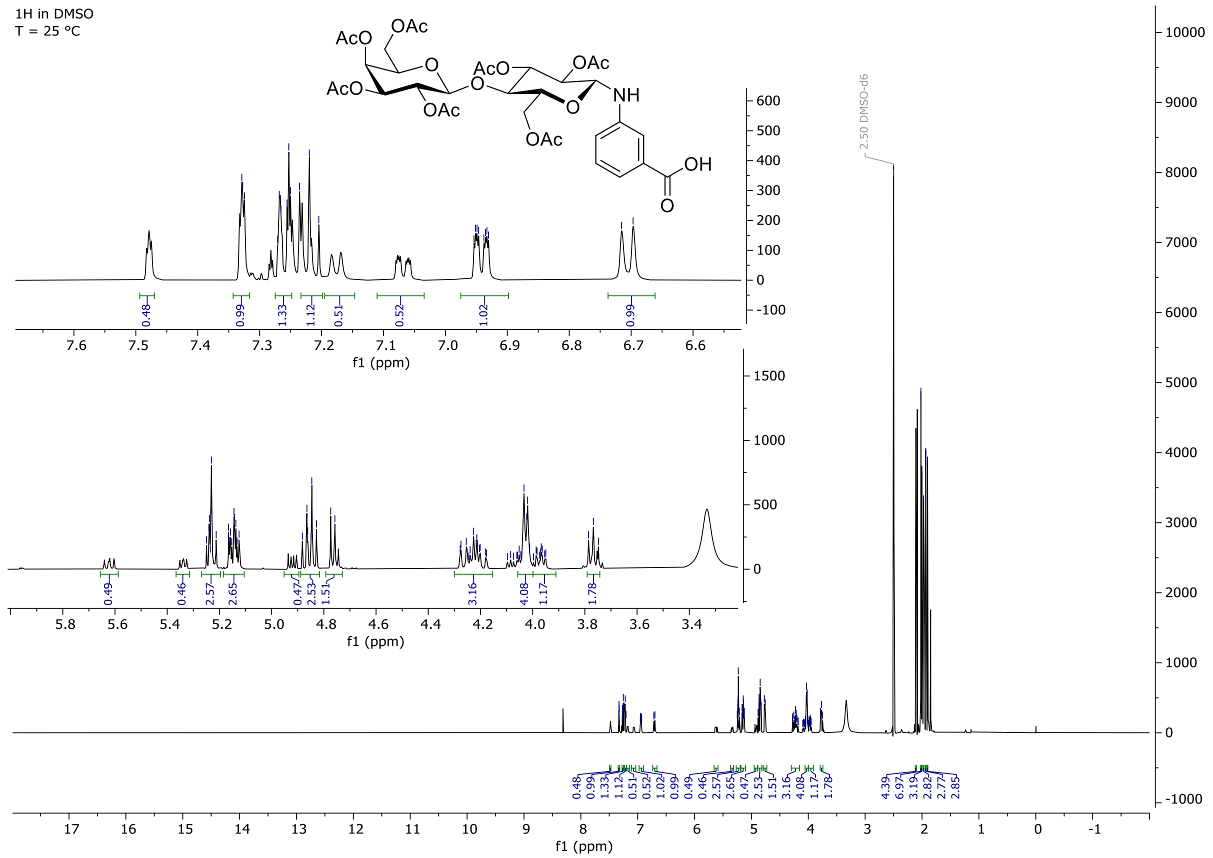

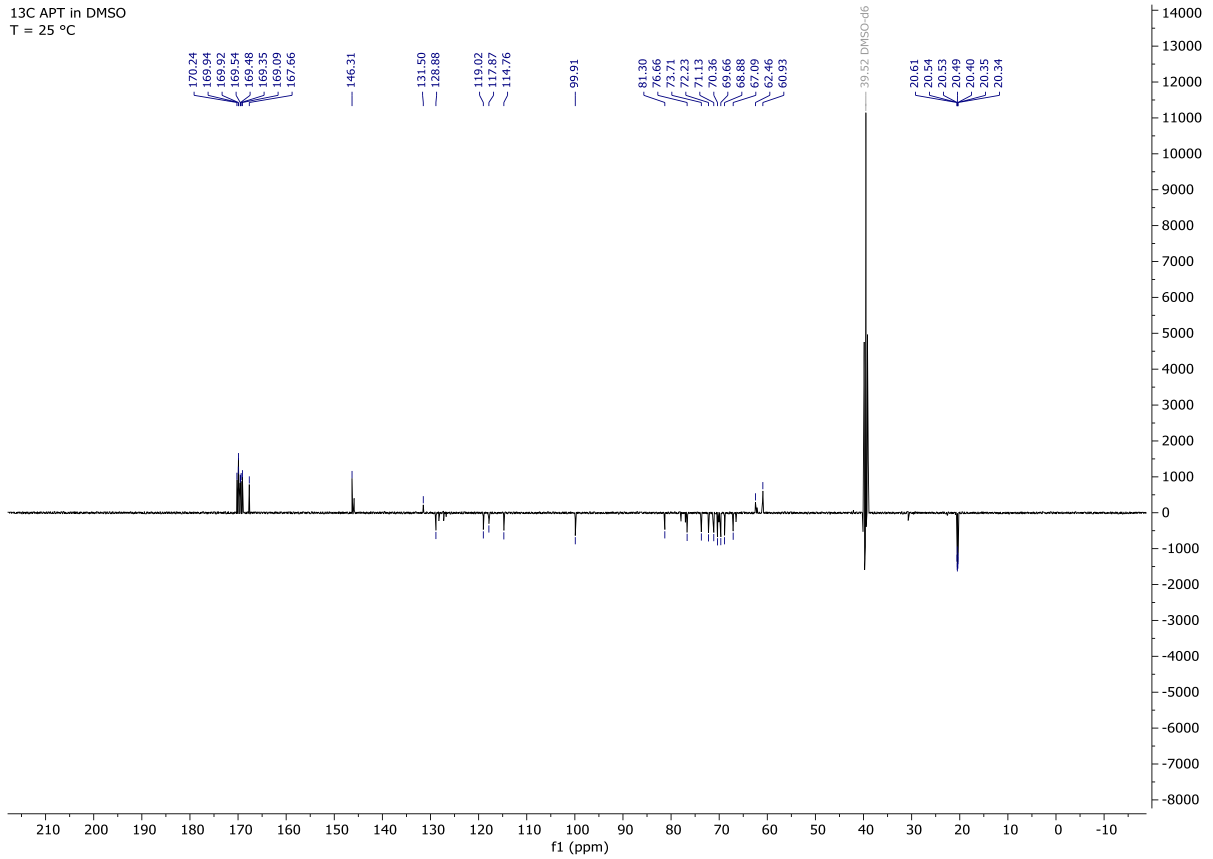

^{1}H and ^{13}C APT NMR spectra of (2s)

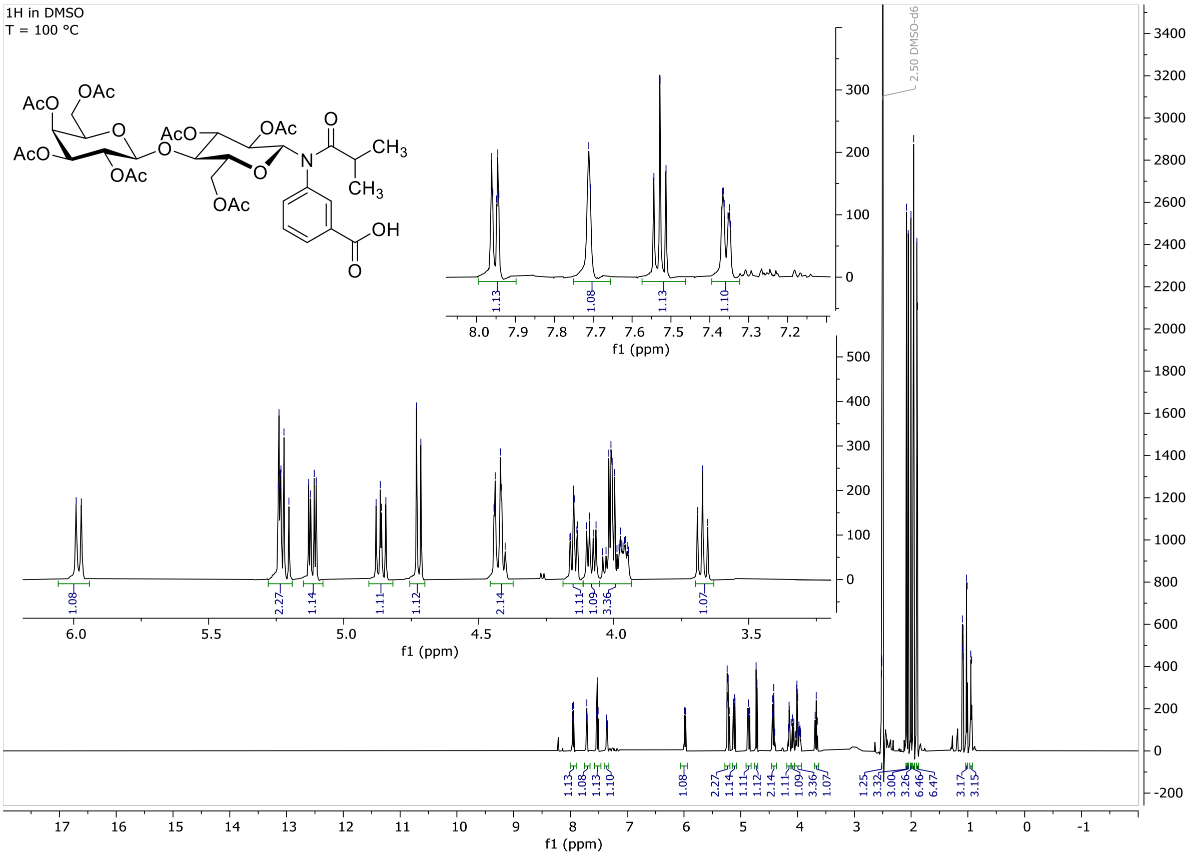

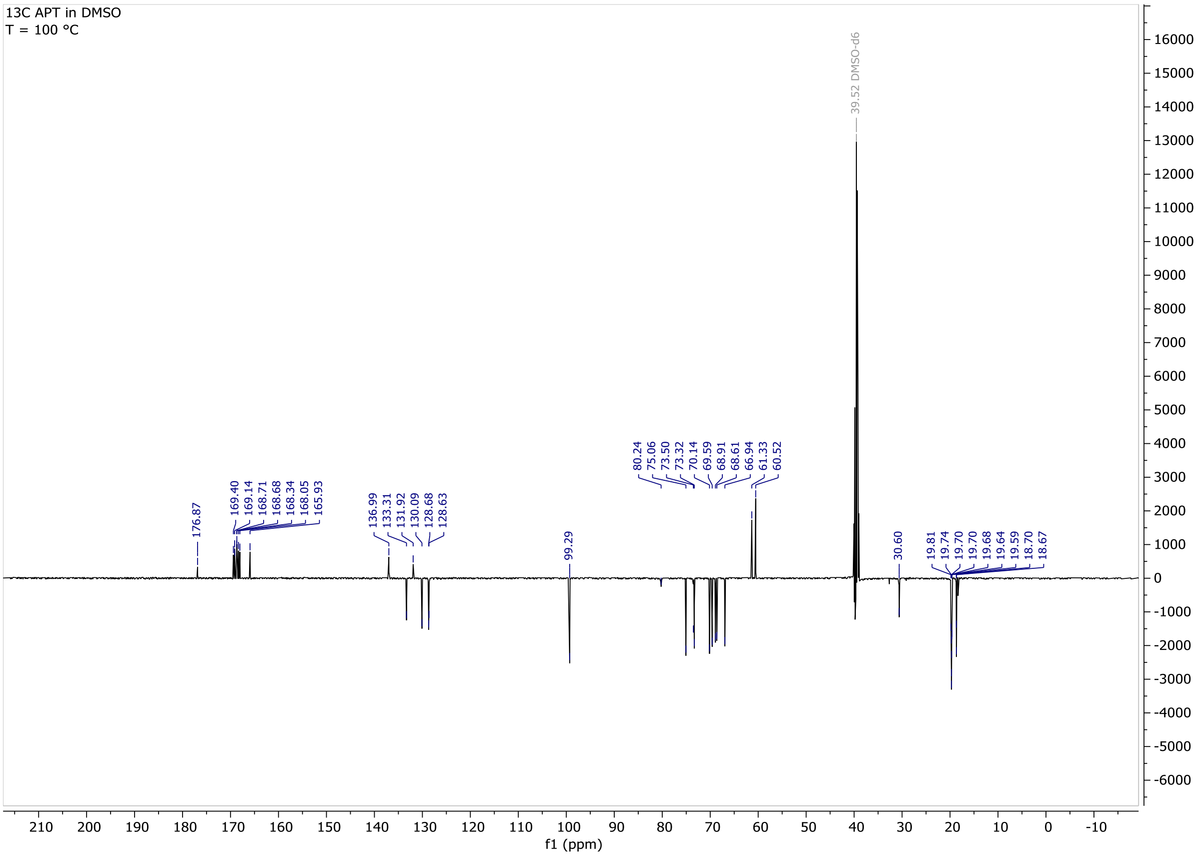

^{1}H and ^{13}C APT NMR spectra of (2t)

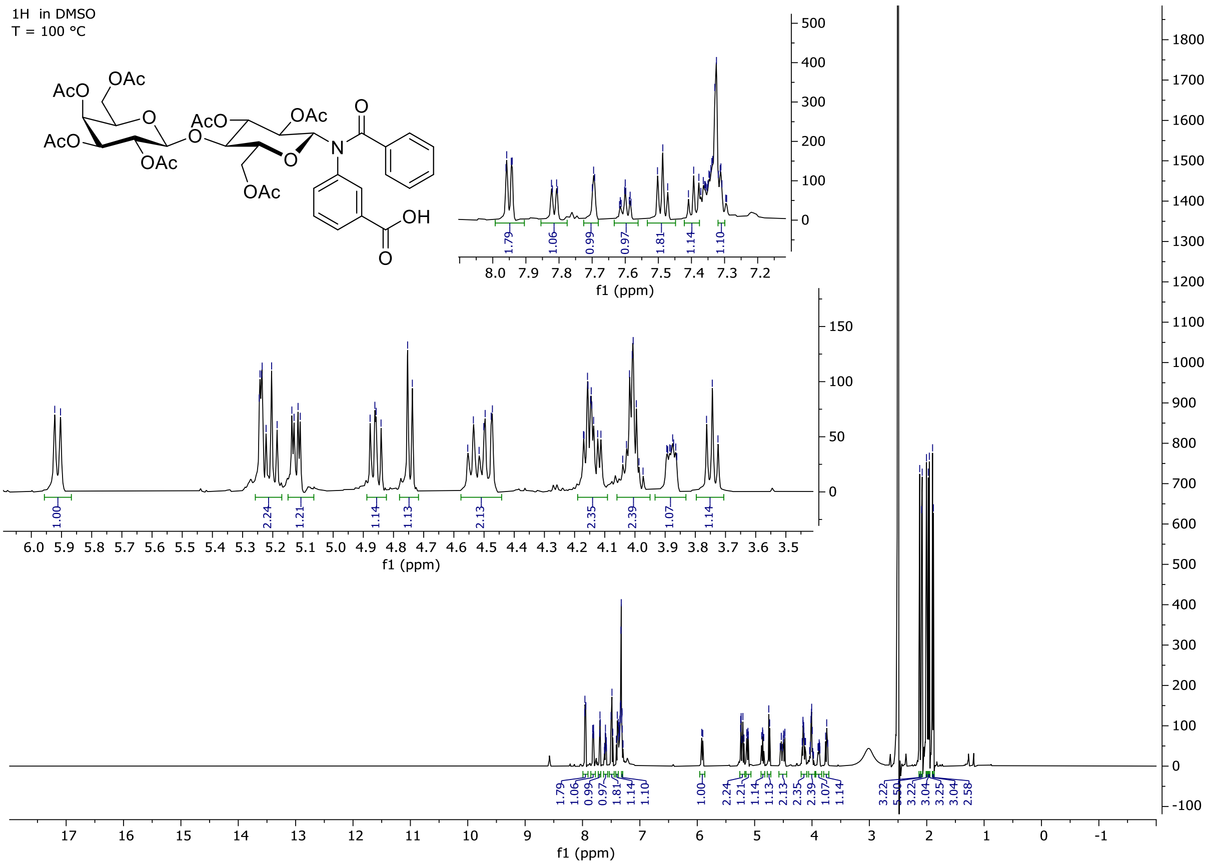

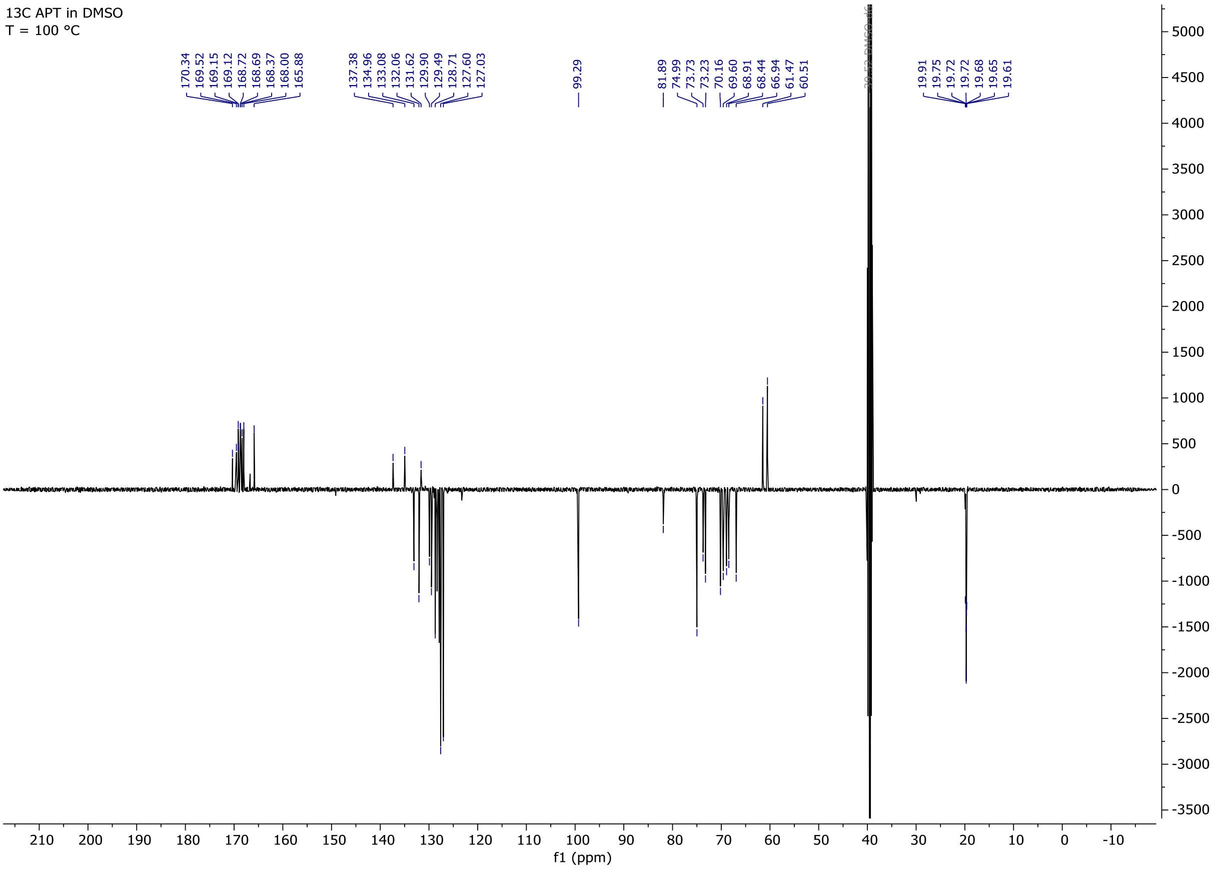

^{1}H and ^{13}C APT NMR spectra of (2u)

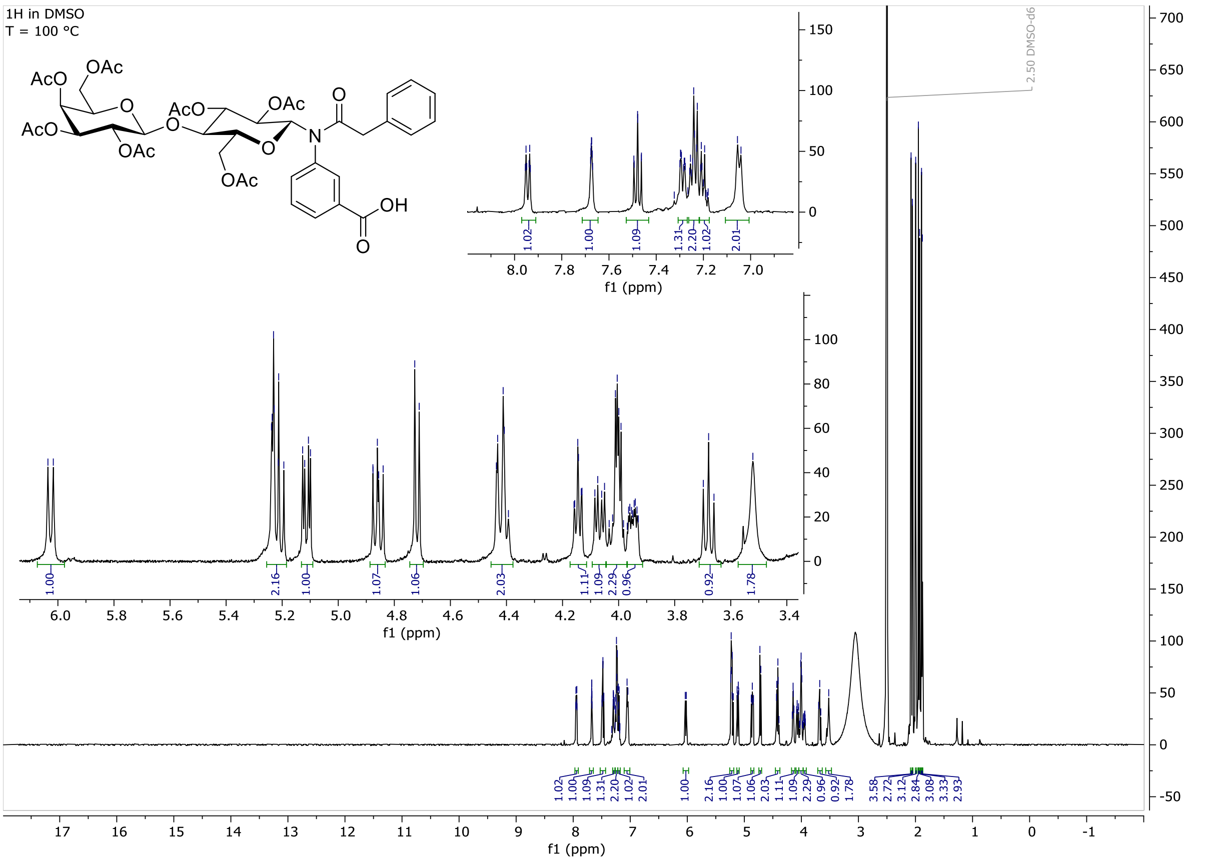

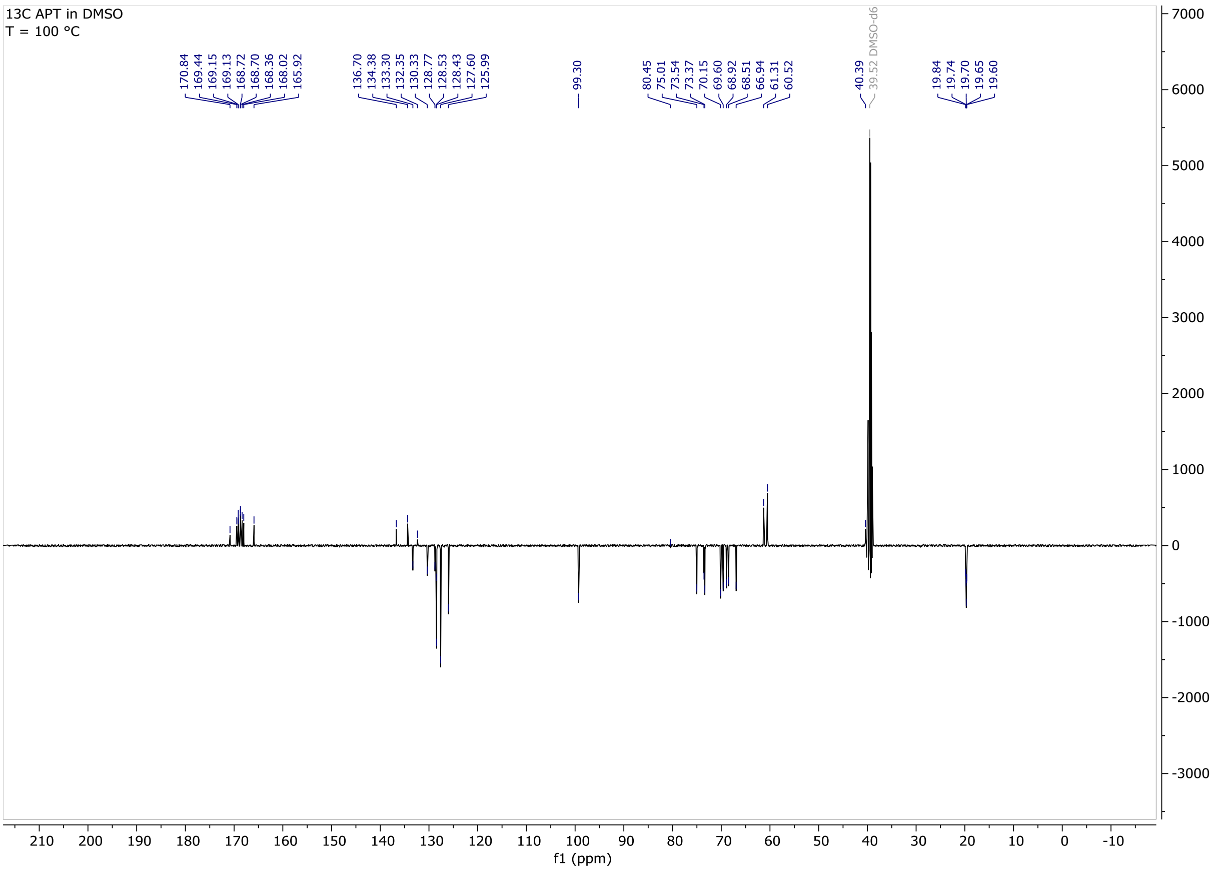

^{1}H and ^{13}C APT NMR spectra of (2v)

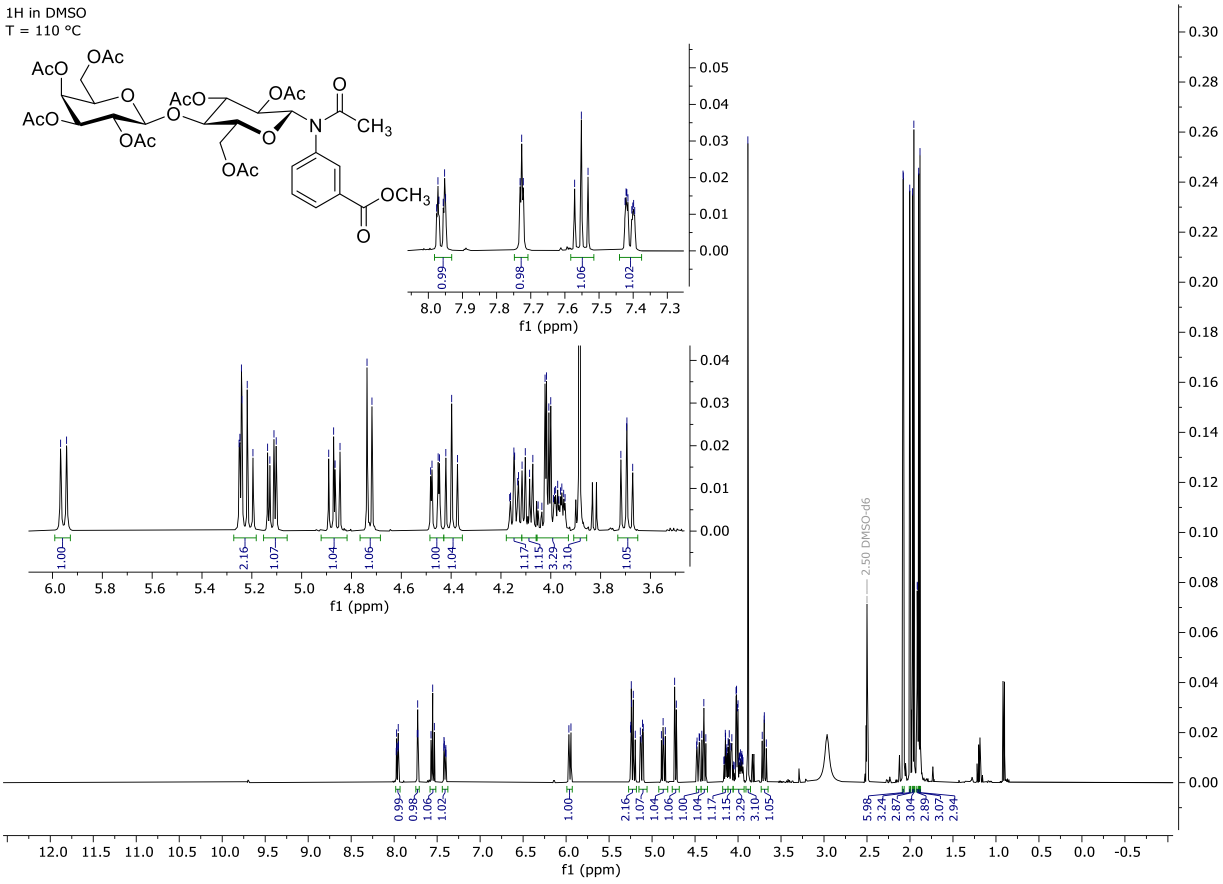

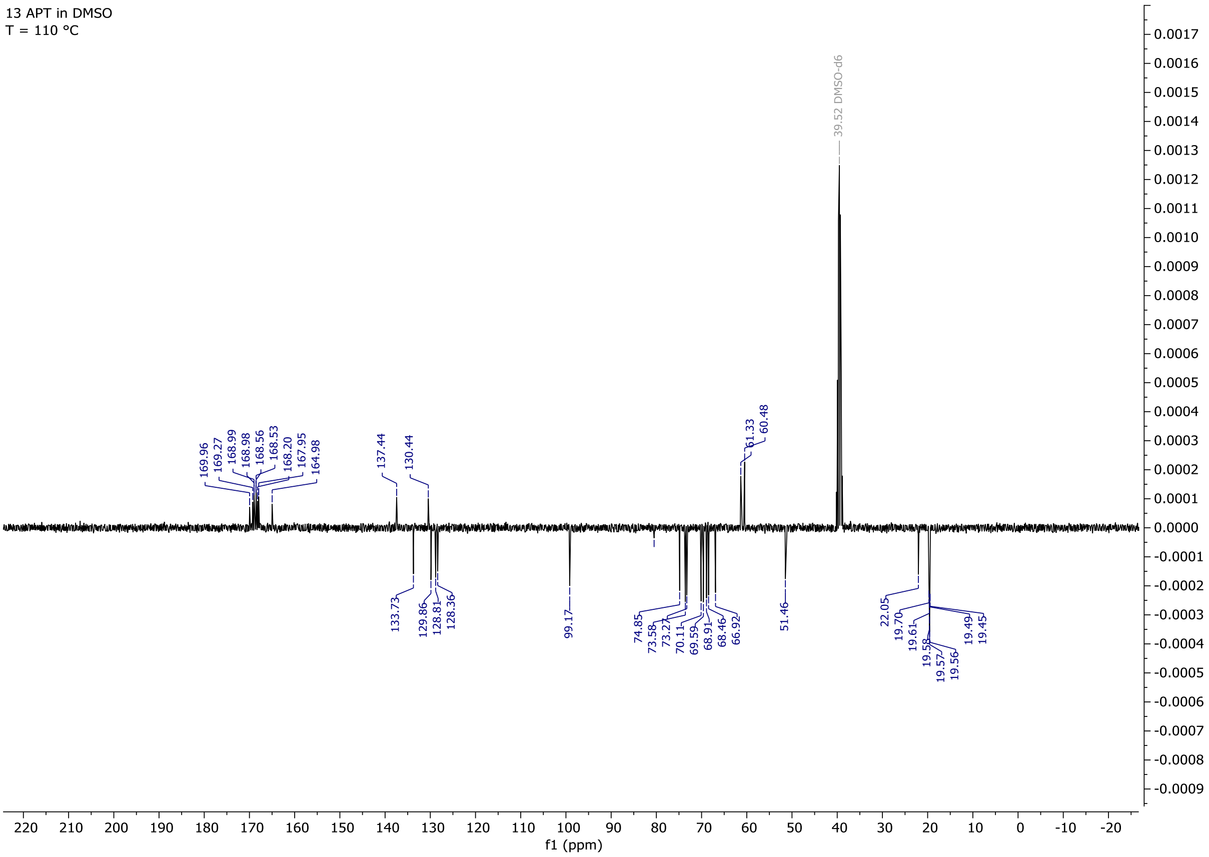

^{1}H and ^{13}C APT NMR spectra of (2x)

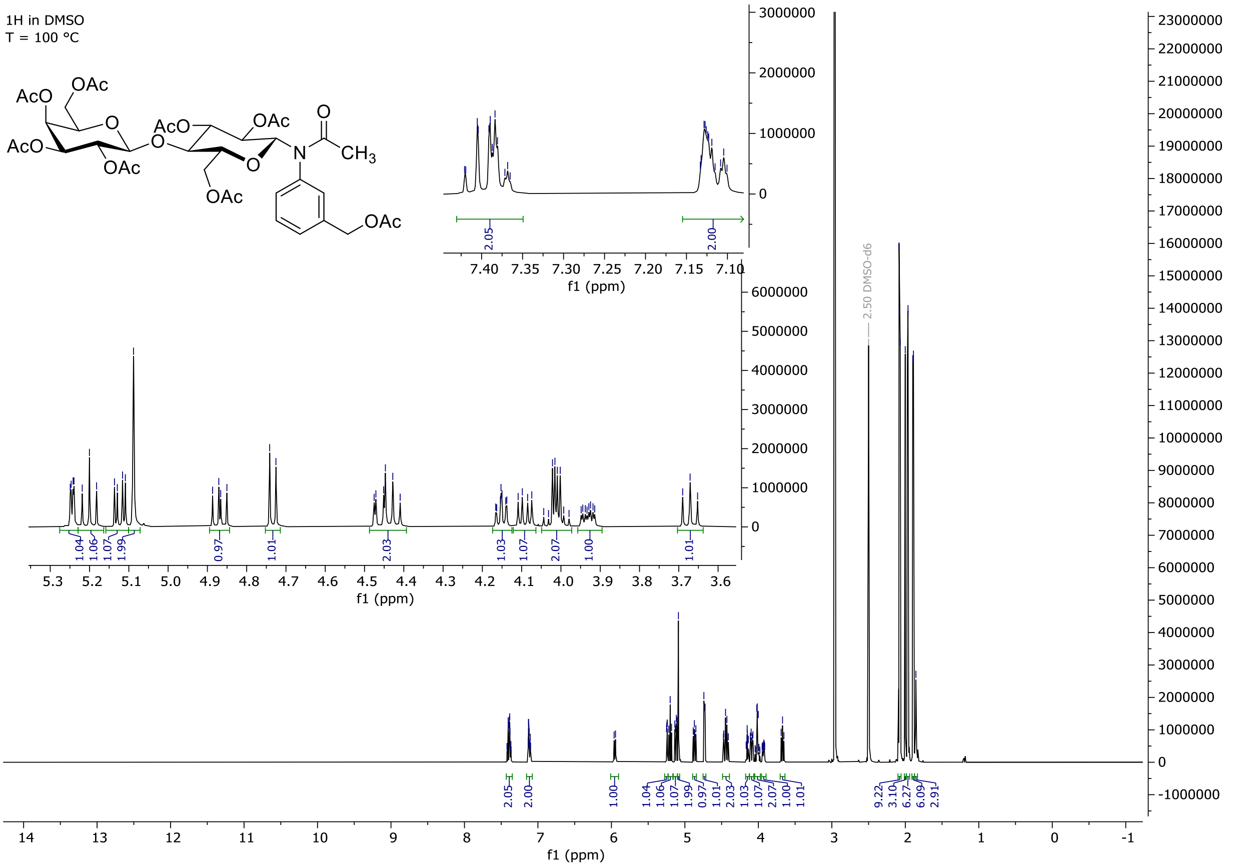

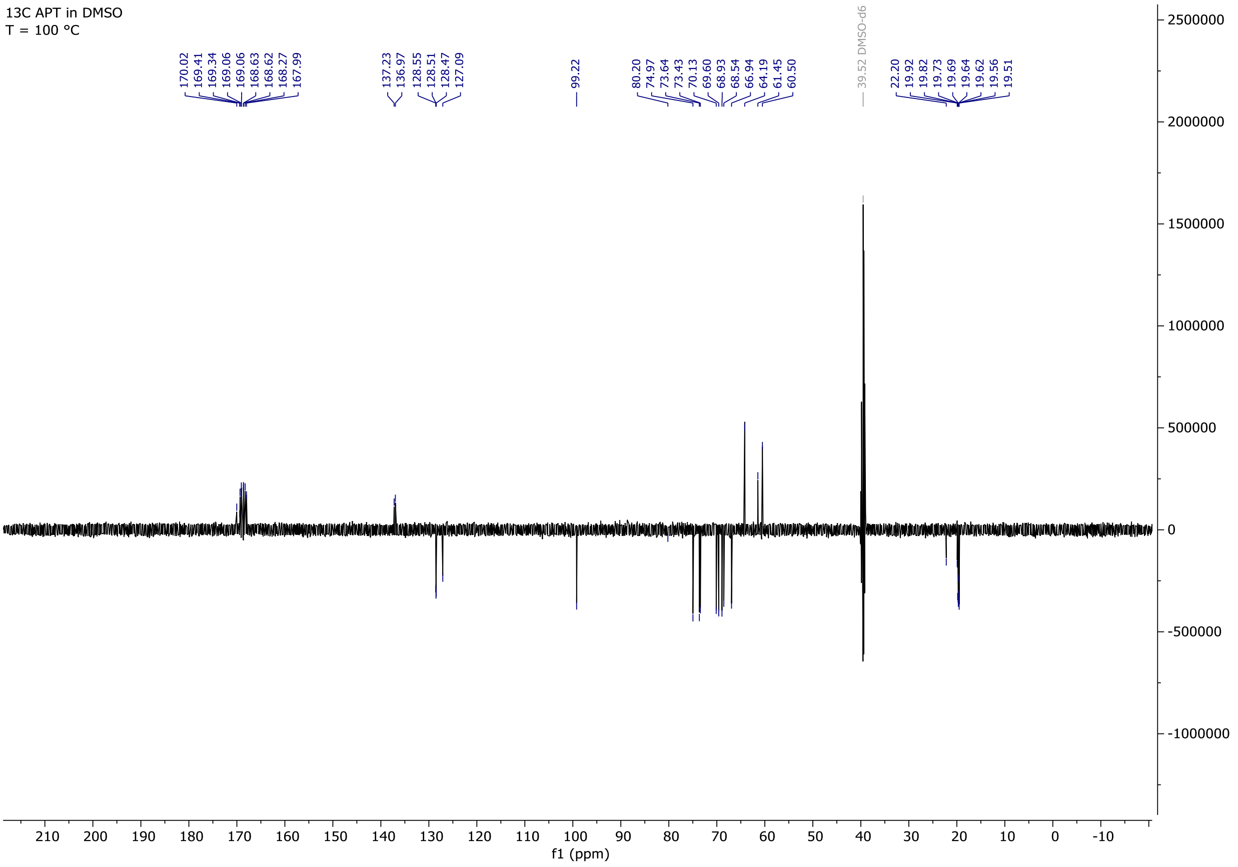

^1H, ^{13}C APT and HSQC NMR spectra of (2y)

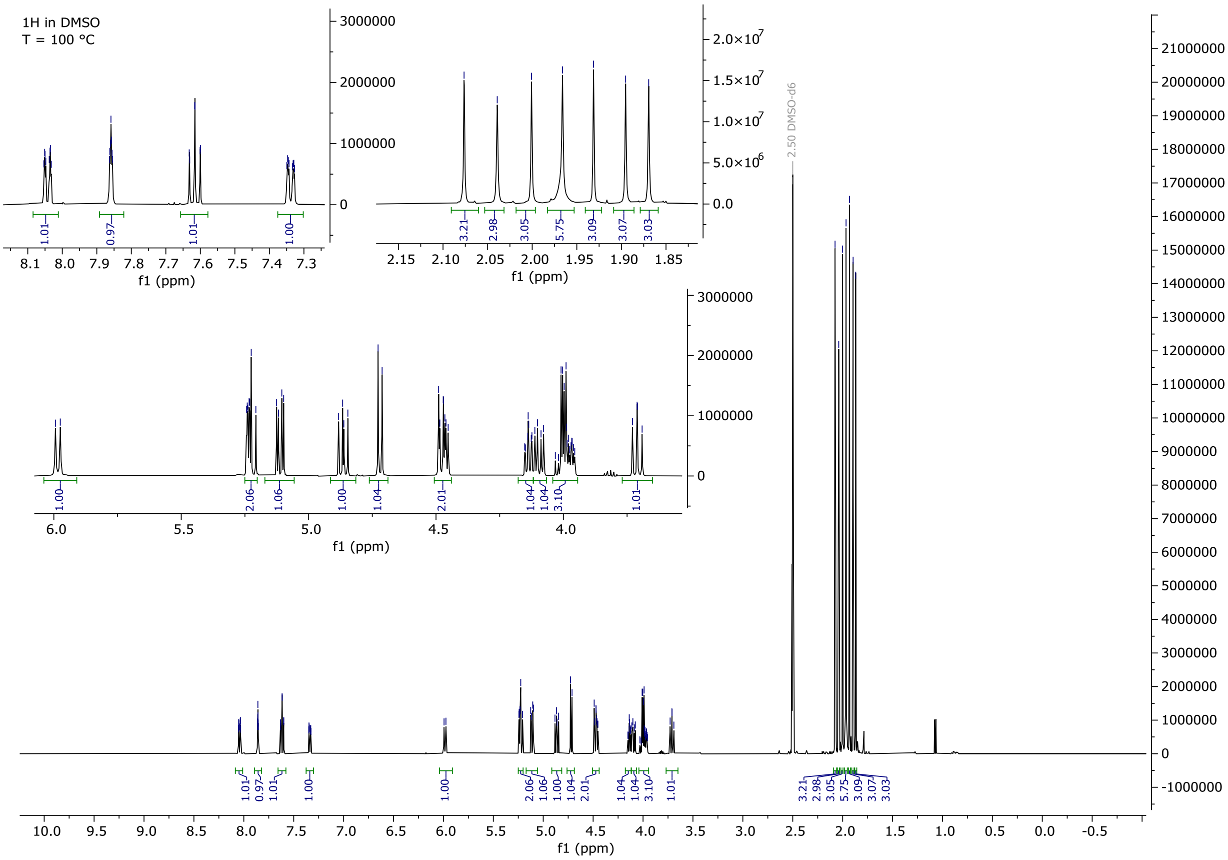

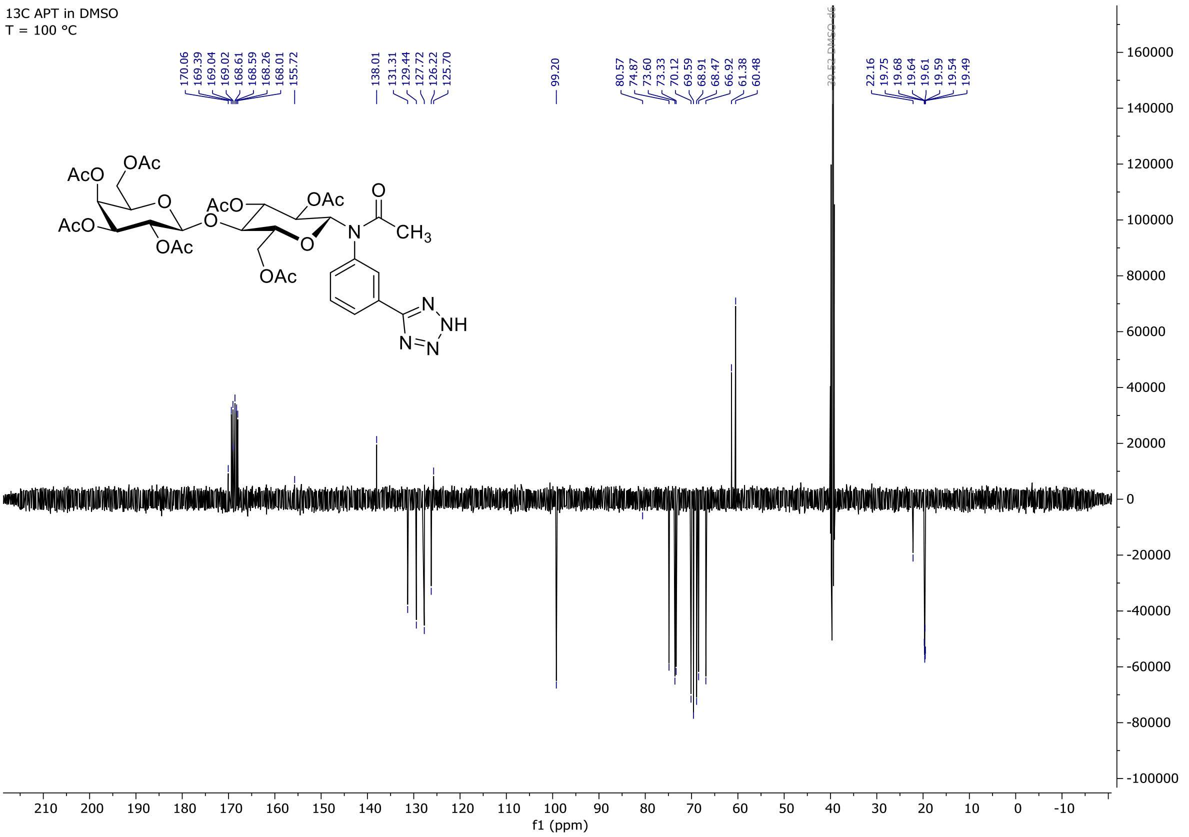

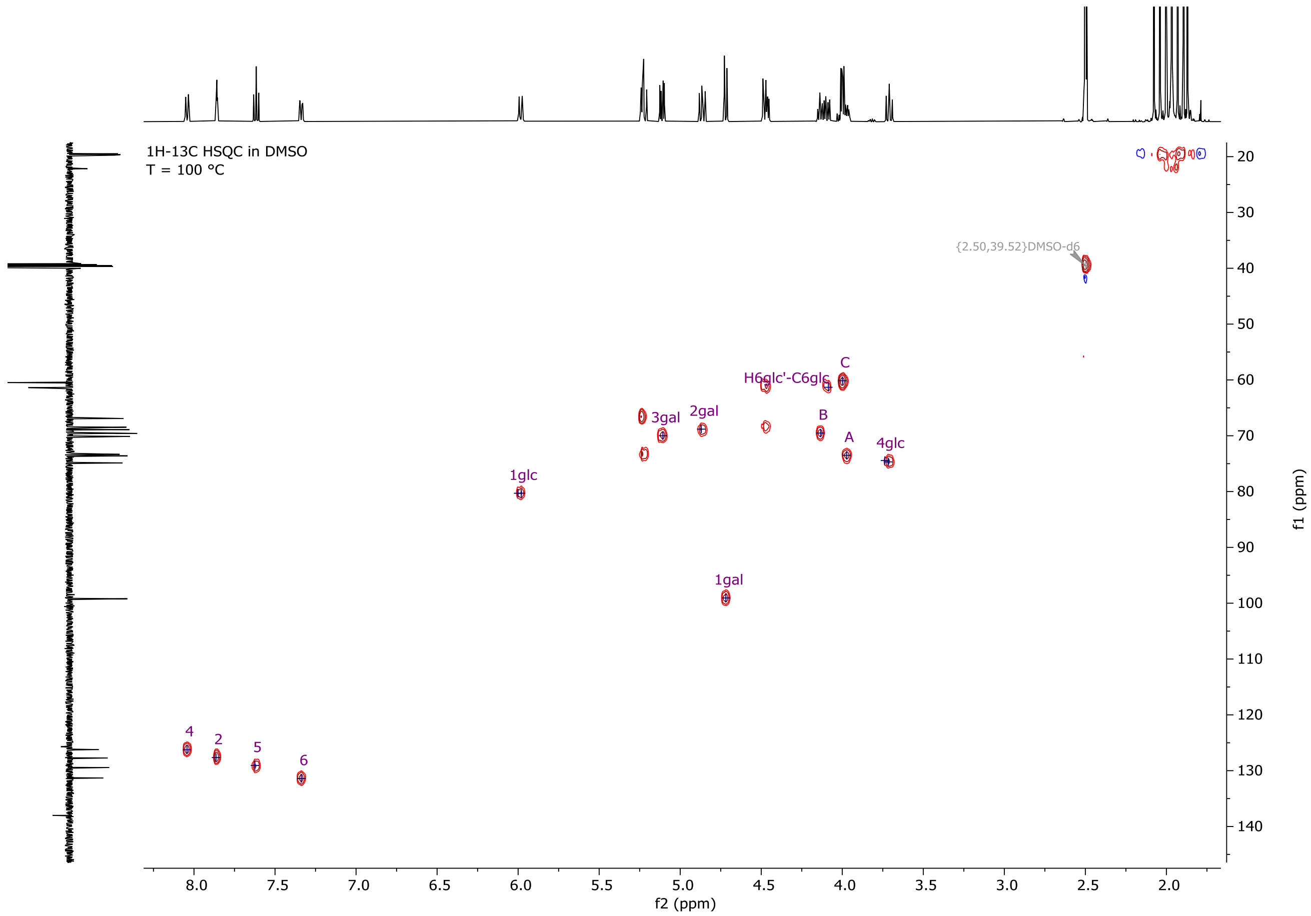
1H-13C HSQC in DMSO
T = 100 °C
{2.50,39.52}DMSO-d6
C
H6glc'-C6glc
B
A
4glc
3gal
2gal
1glc
1gal
4
2
5
6
f2 (ppm)
f1 (ppm)

^{1}H and ^{13}C APT NMR spectra of (S3)

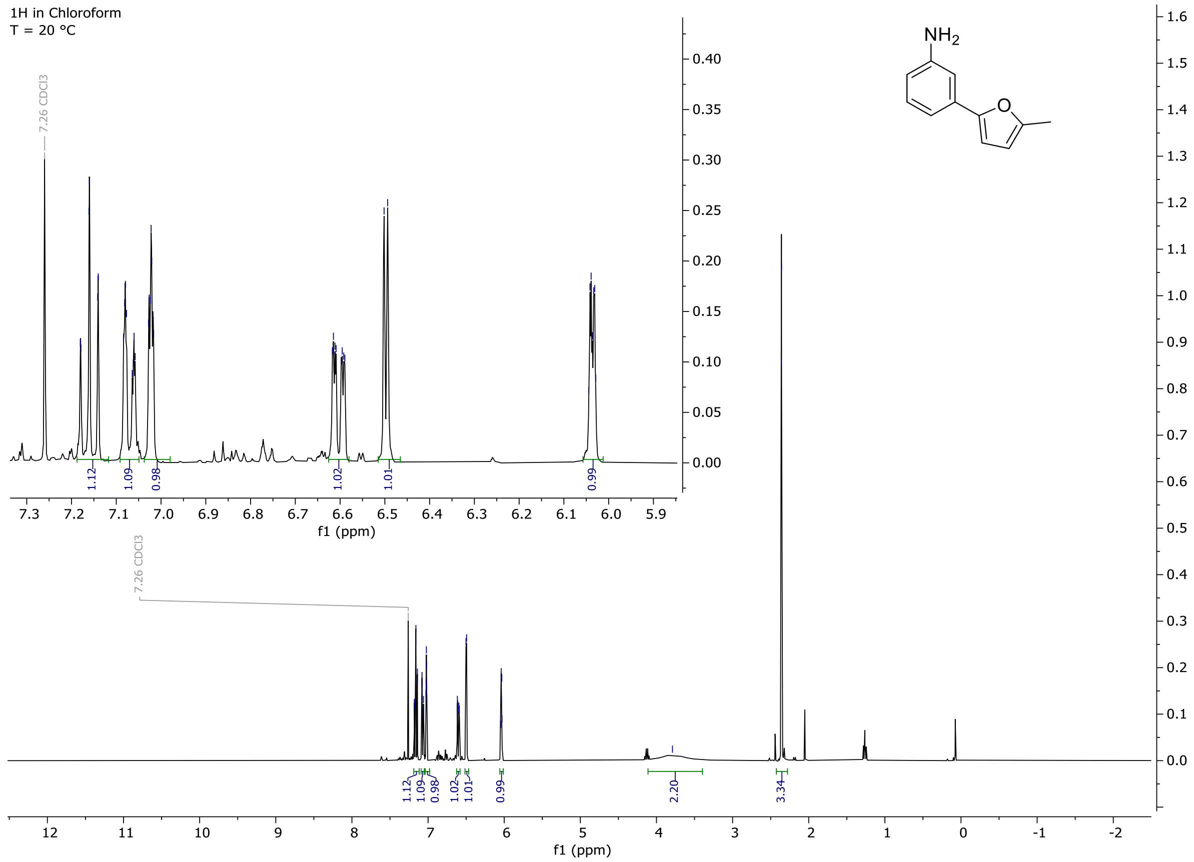

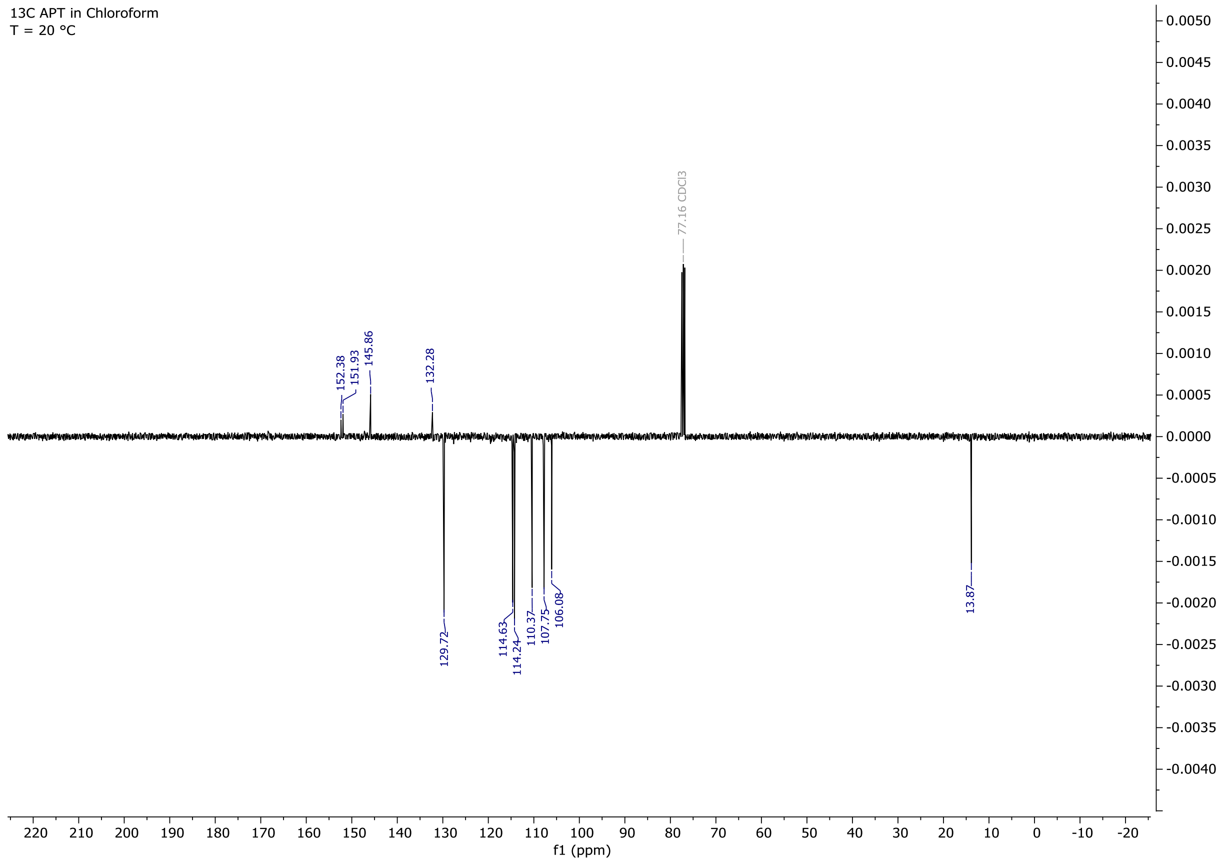

^{1}H, ^{13}C APT and HSQC NMR spectra of (2z)

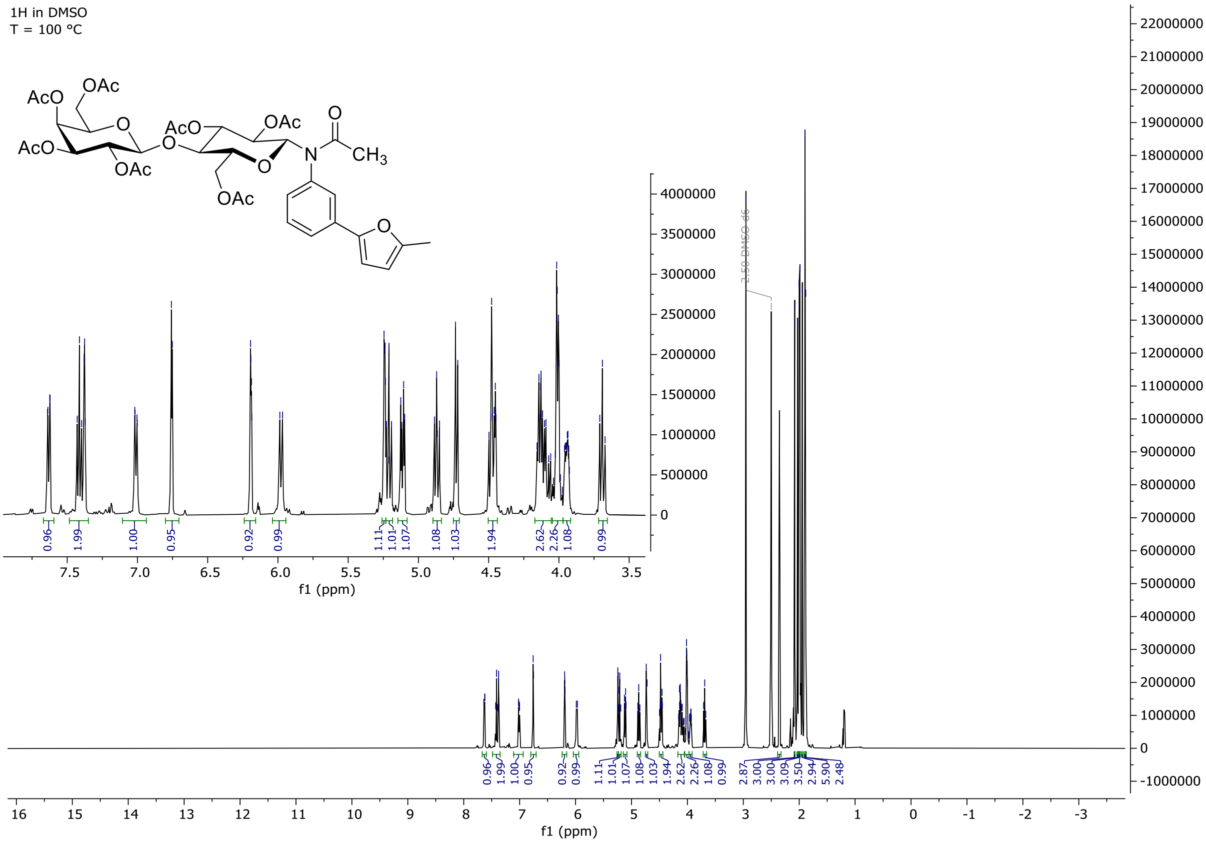

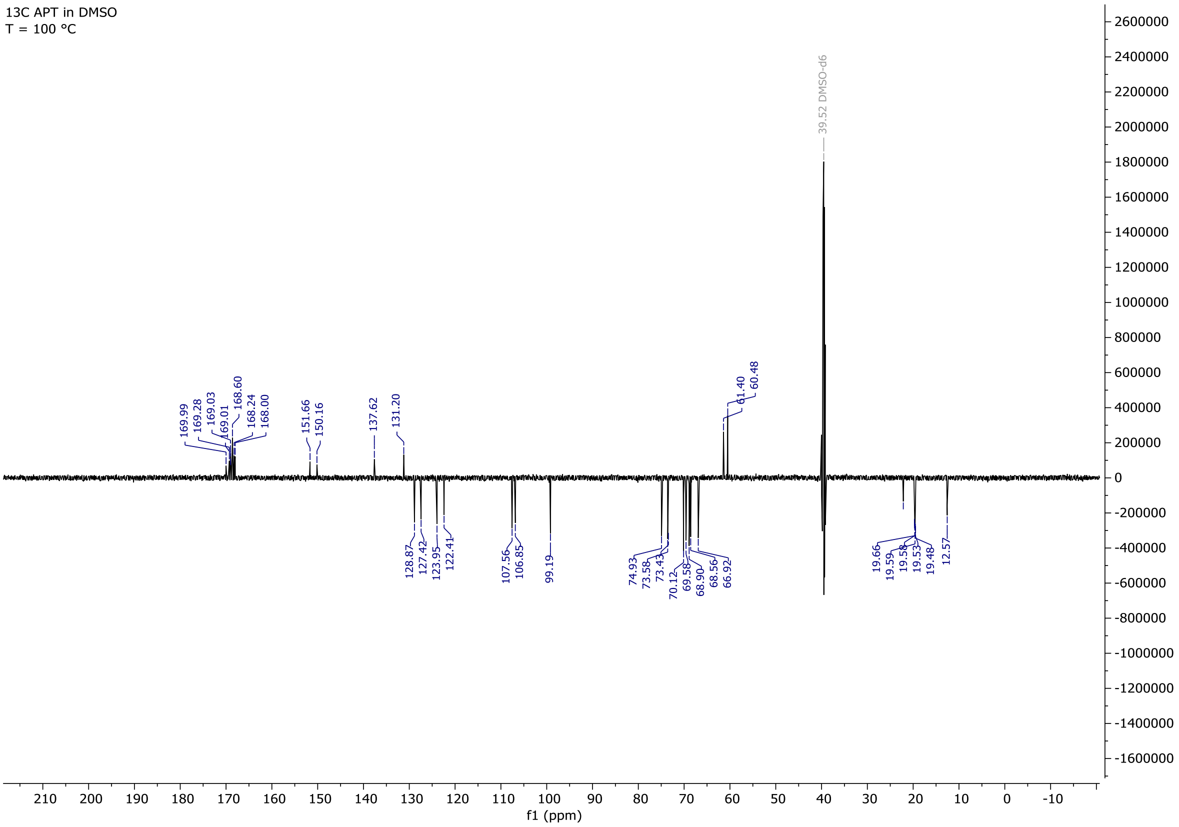

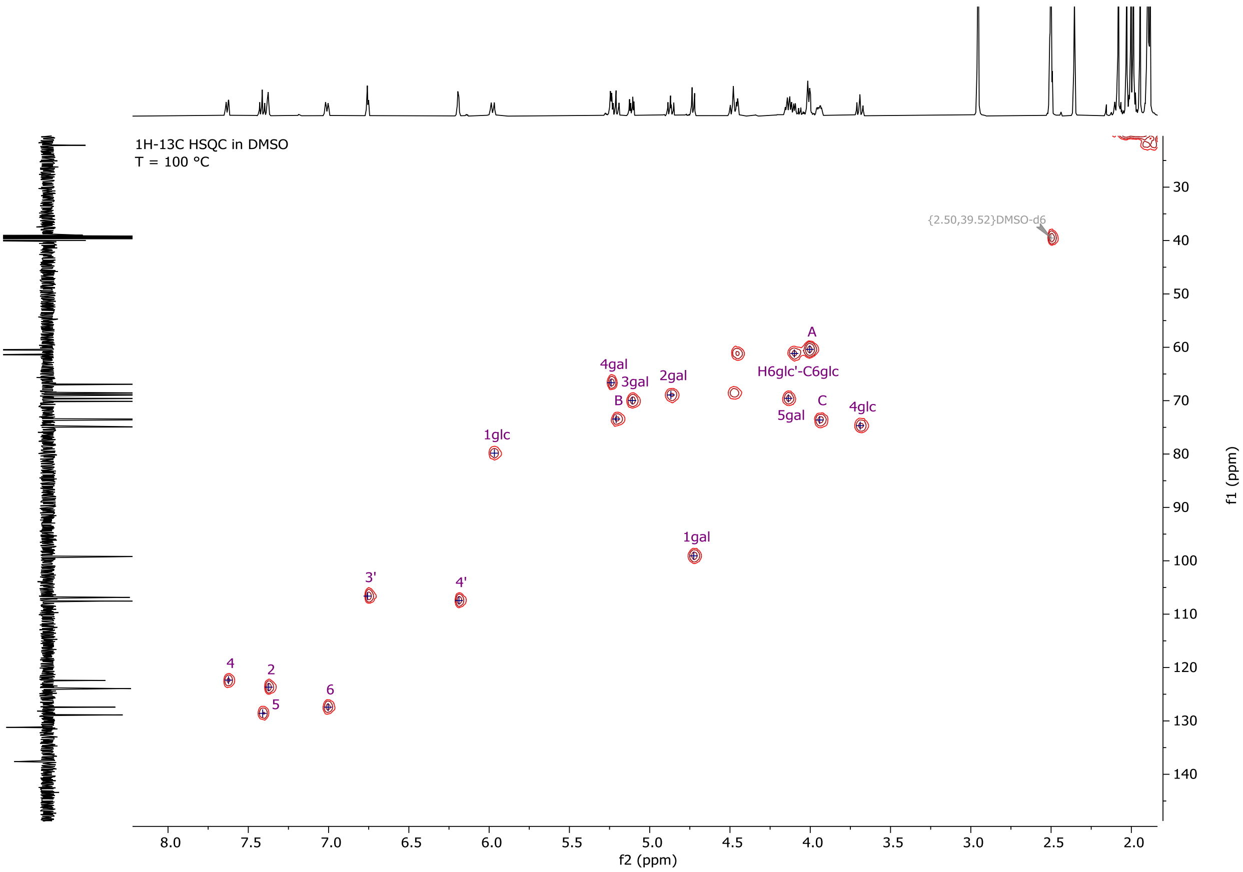

1H-13C HSQC in DMSO
T = 100 °C
{2.50,39.52}DMSO-d6
A
H6glc'-C6glc
4gal
3gal
2gal
B
5gal
C
4glc
1glc
1gal
3'
4'
4
2
5
6
f2 (ppm)
f1 (ppm)

^1H, ^{13}C APT and HSQC NMR spectra of (2aa)

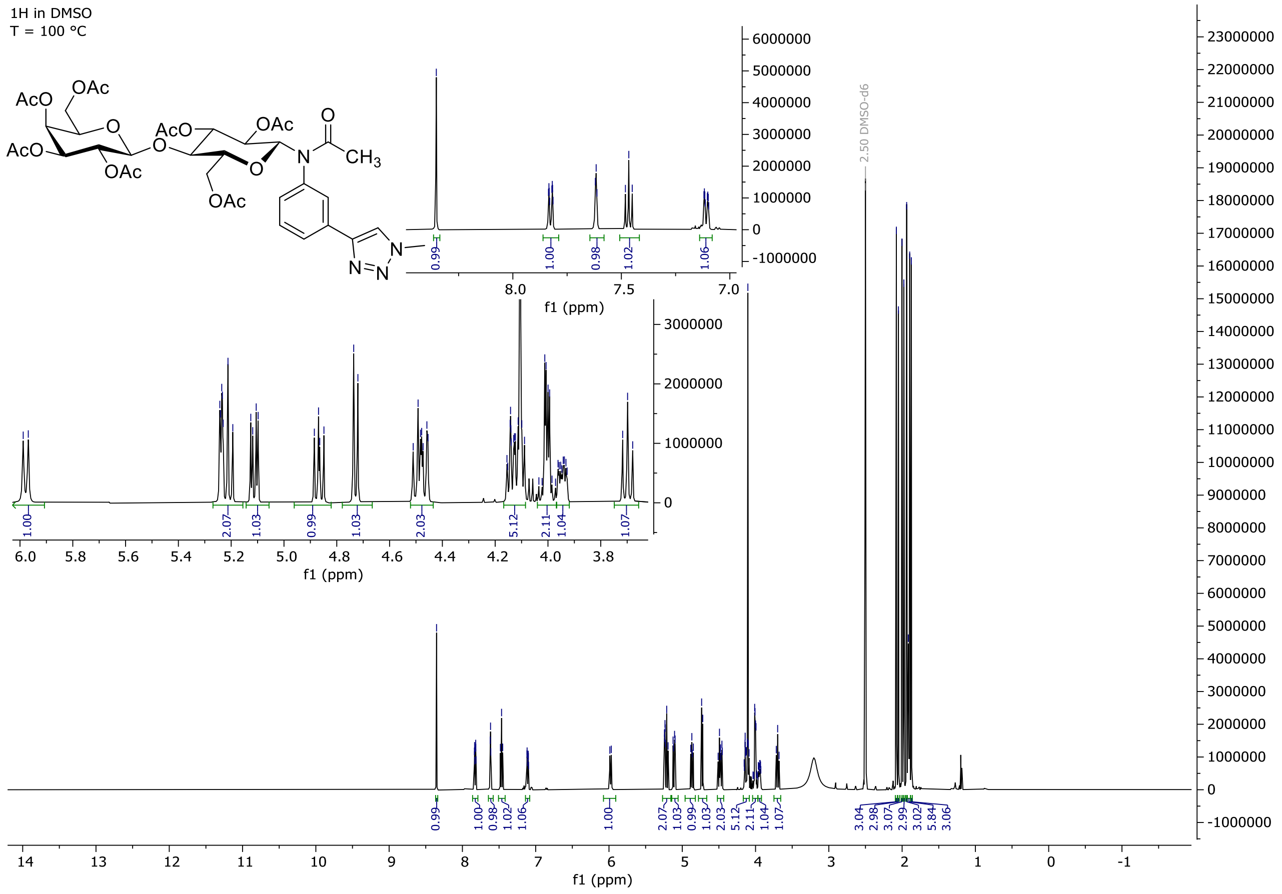

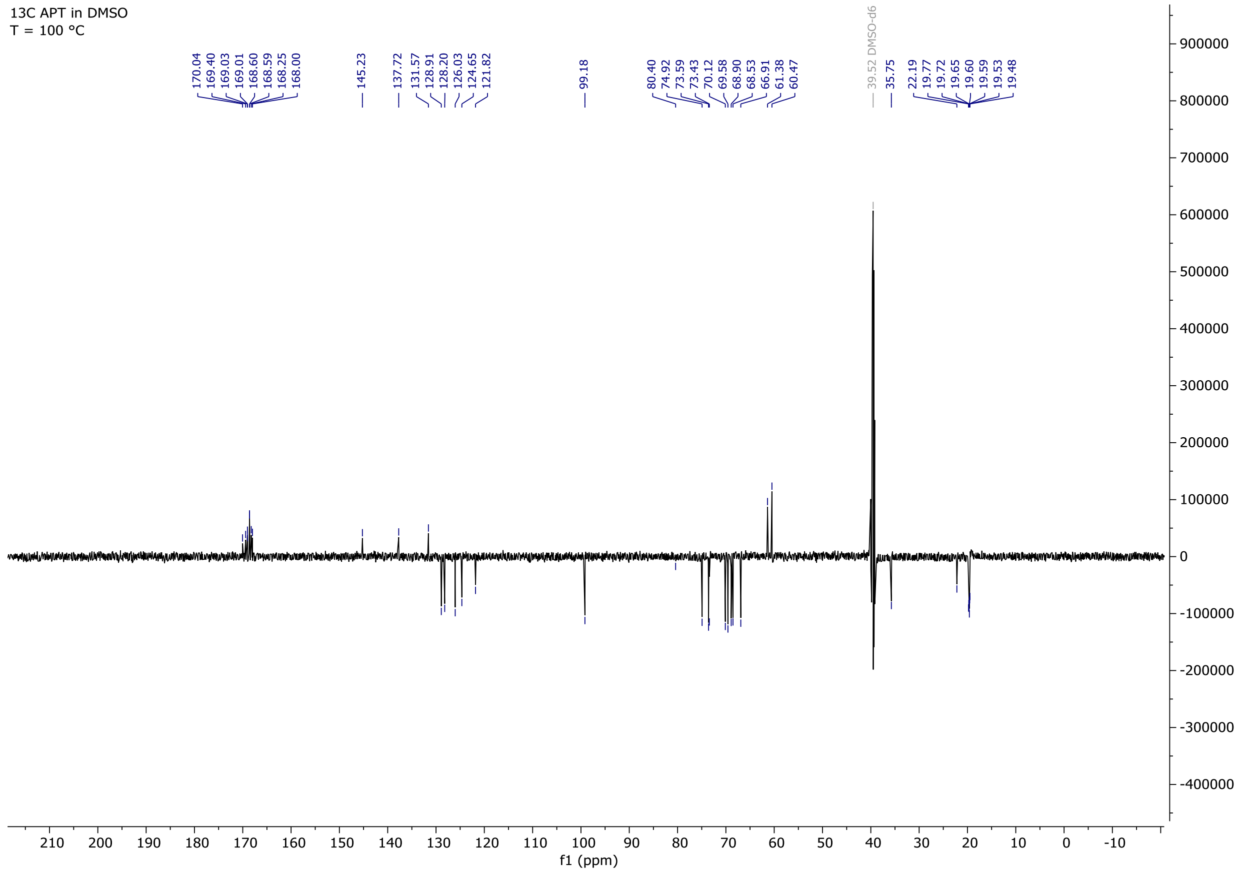

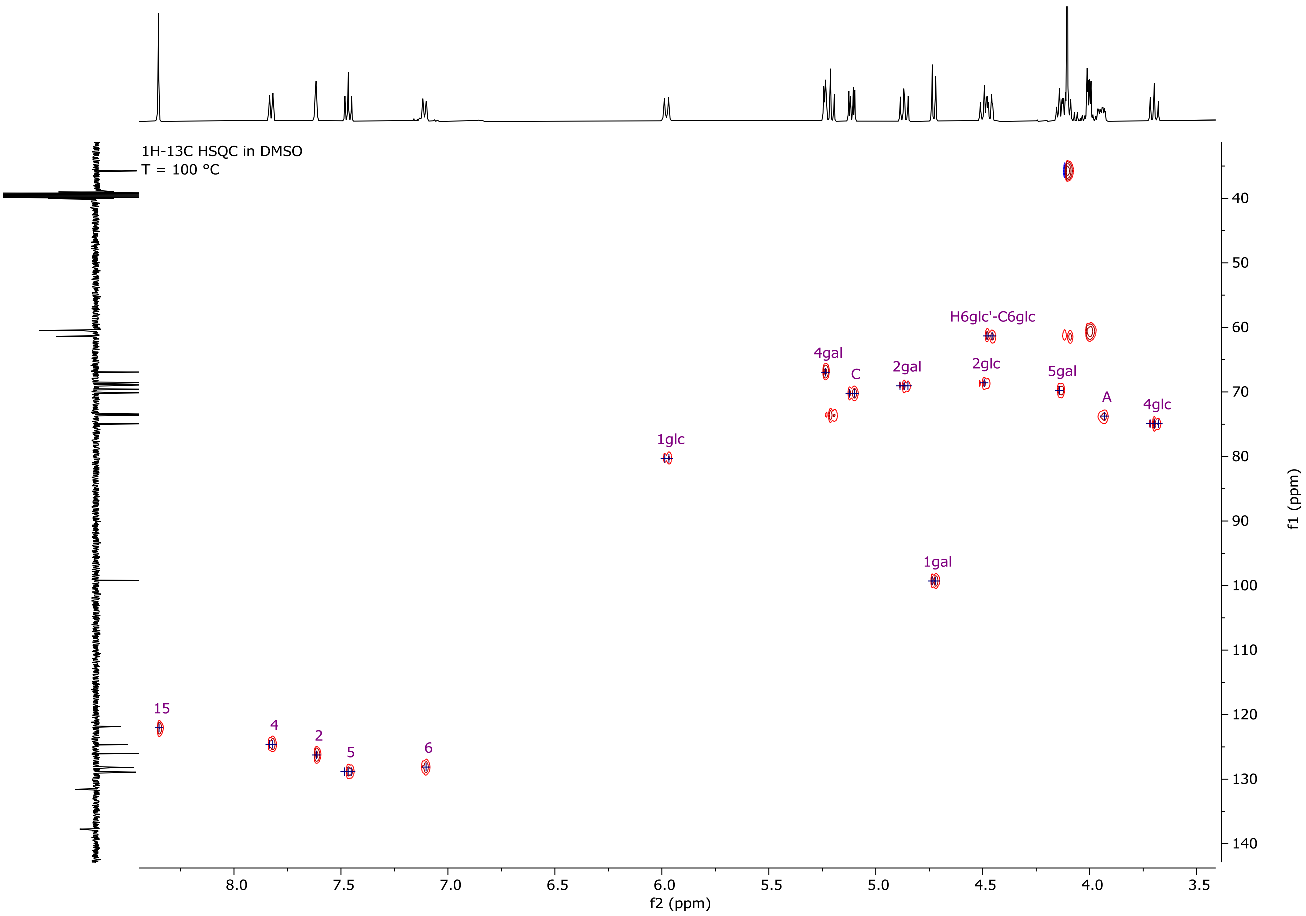

1H-13C HSQC in DMSO
T = 100 °C
H6glc'-C6glc
4gal
C
2gal
2glc
5gal
A
4glc
1glc
1gal
15
4
2
5
6
f2 (ppm)
f1 (ppm)

^{1}H and ^{13}C APT NMR spectra of (2ab)

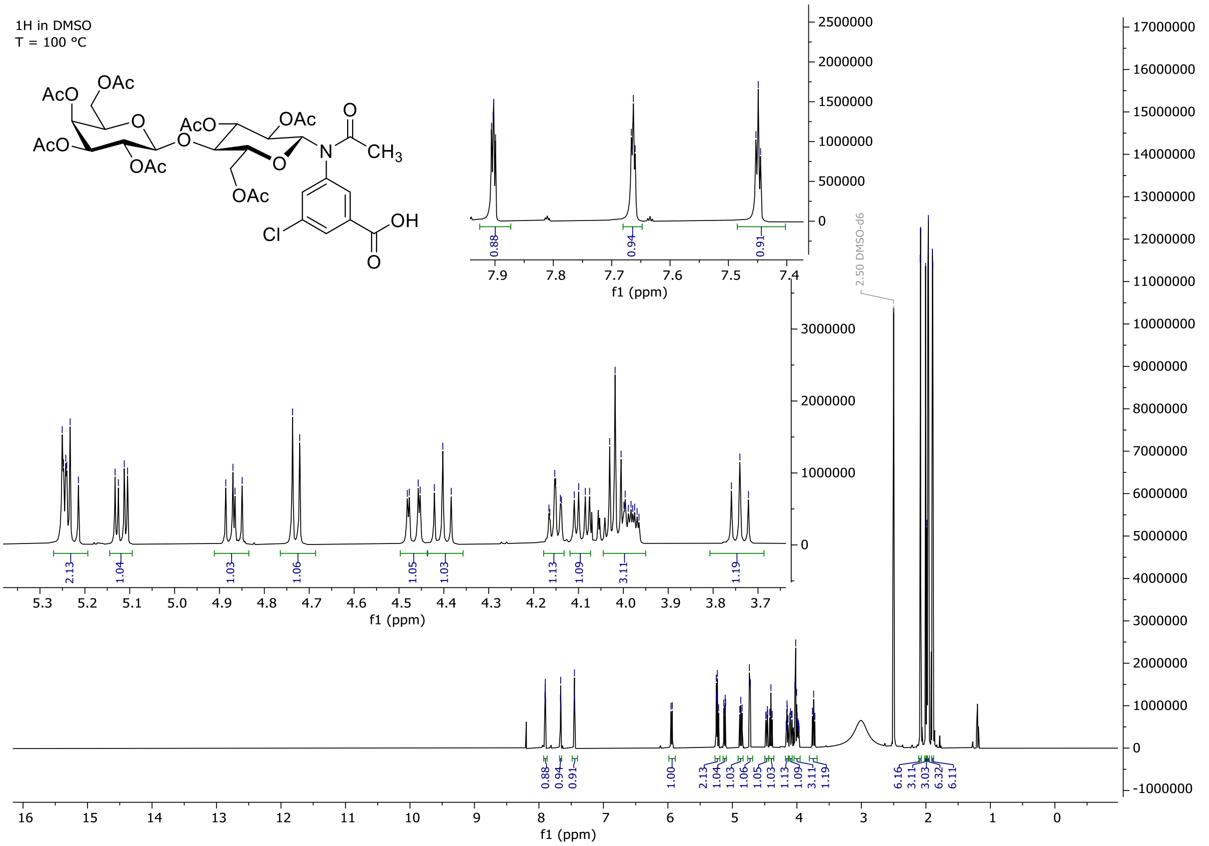

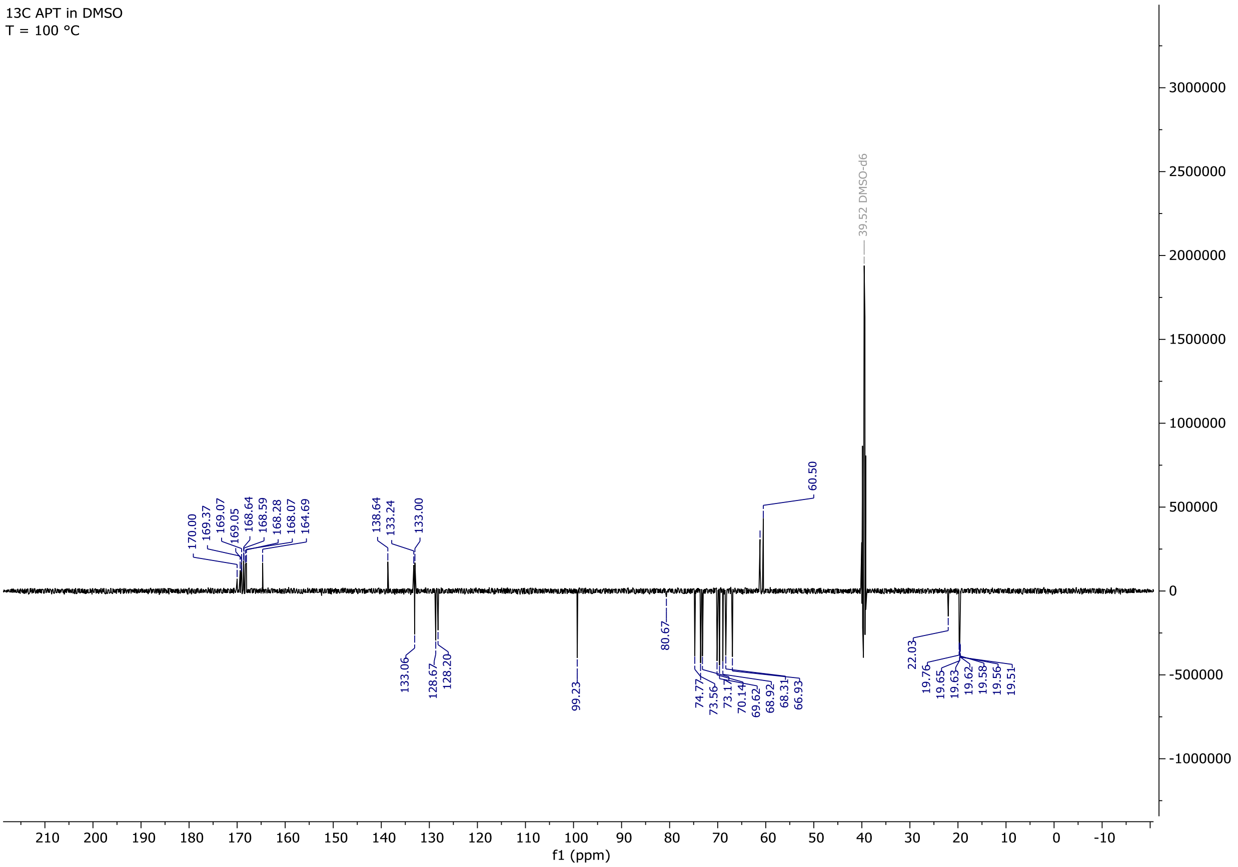

^{1}H and ^{13}C APT NMR spectra of (2ac)

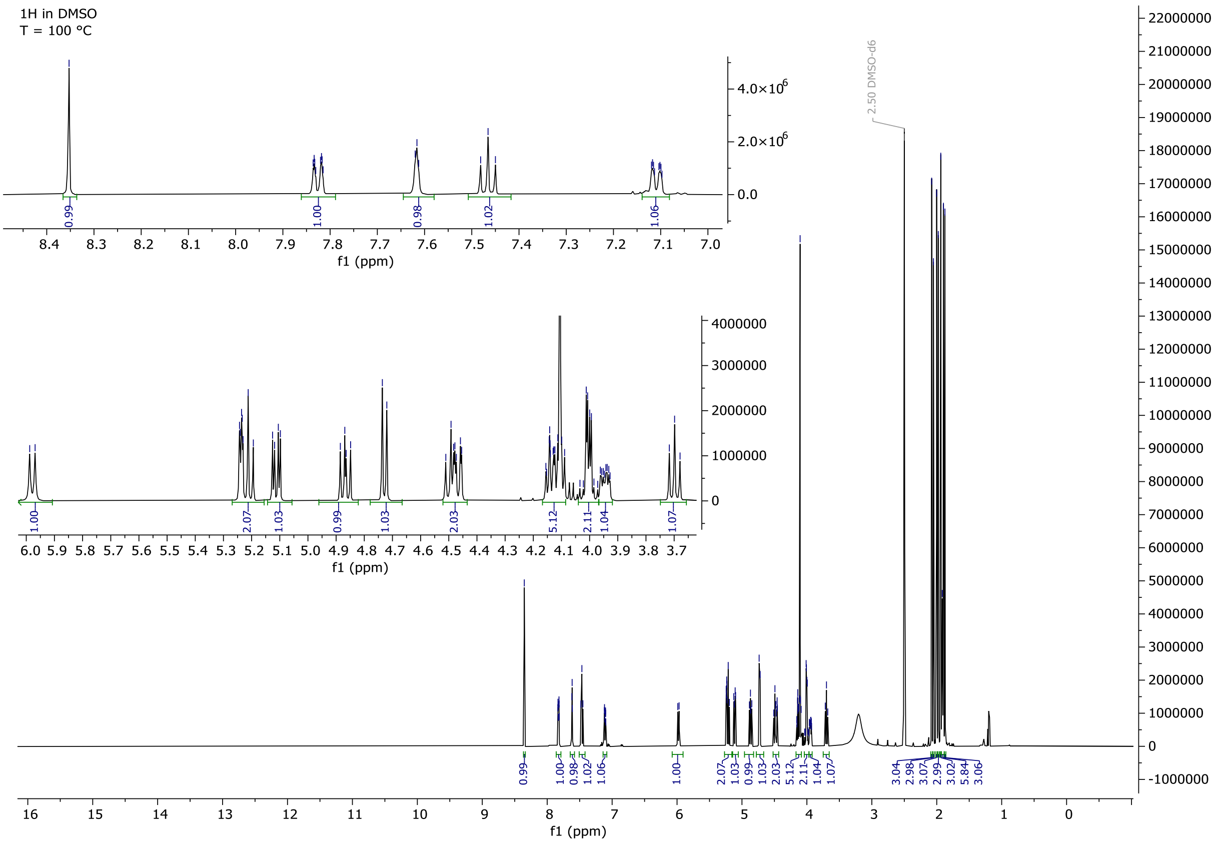

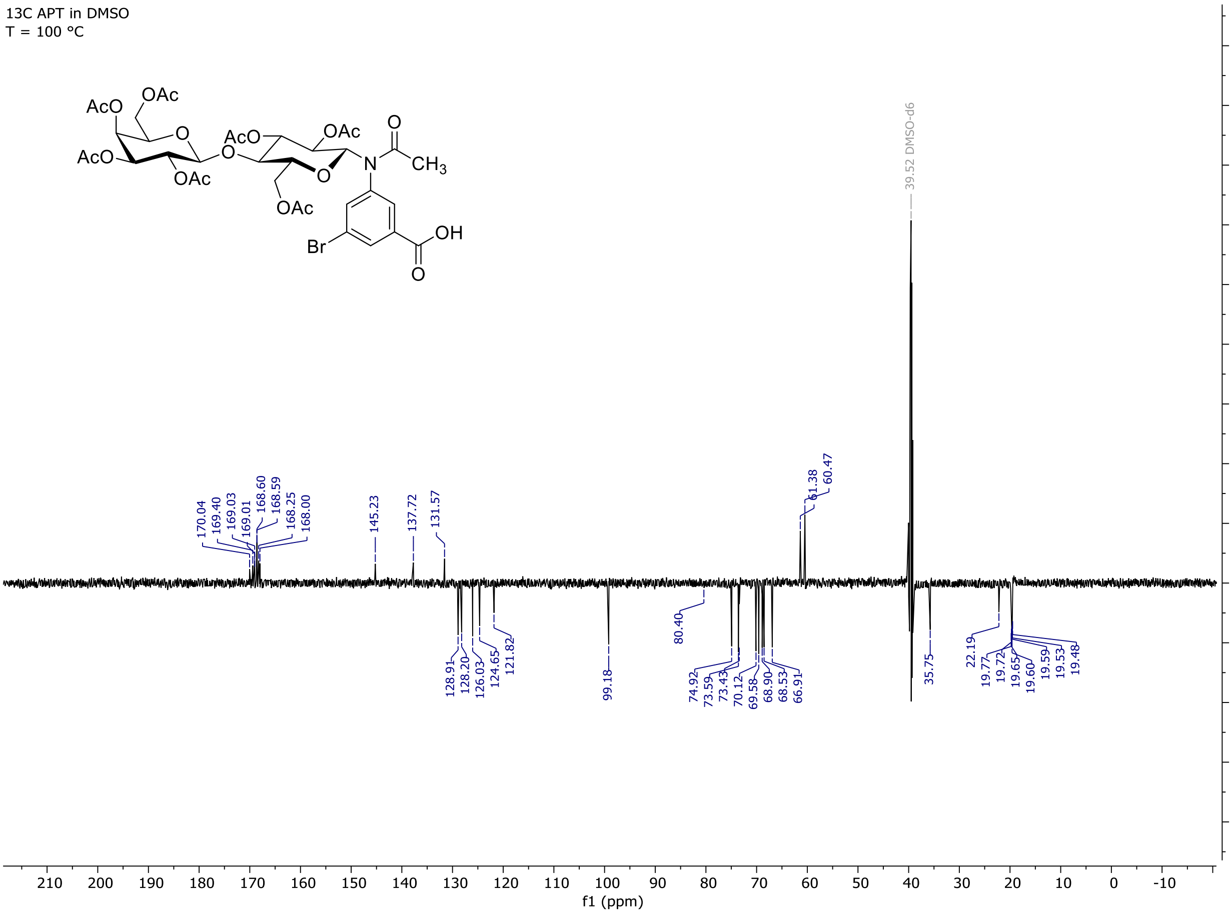

^{1}H and ^{13}C APT NMR spectra of (2ad)

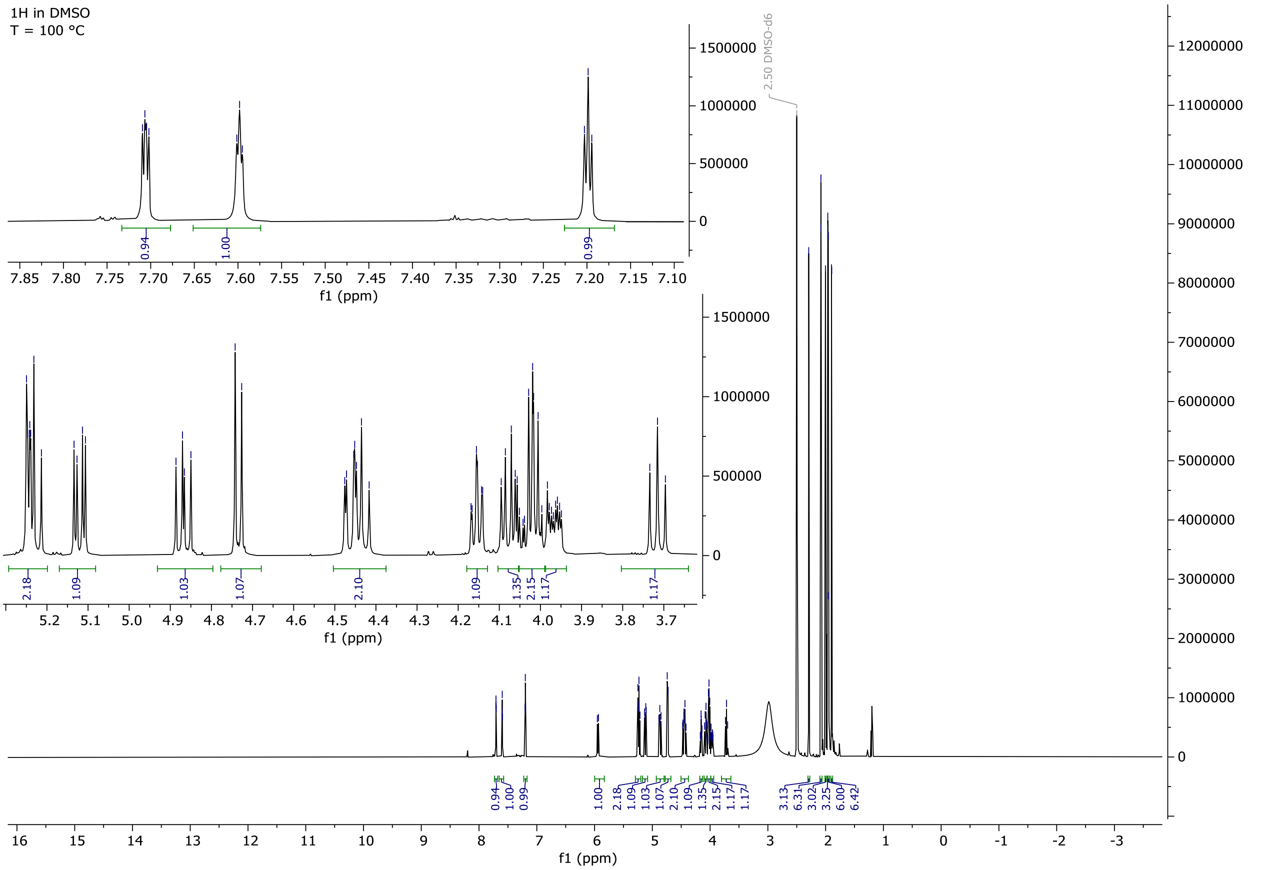

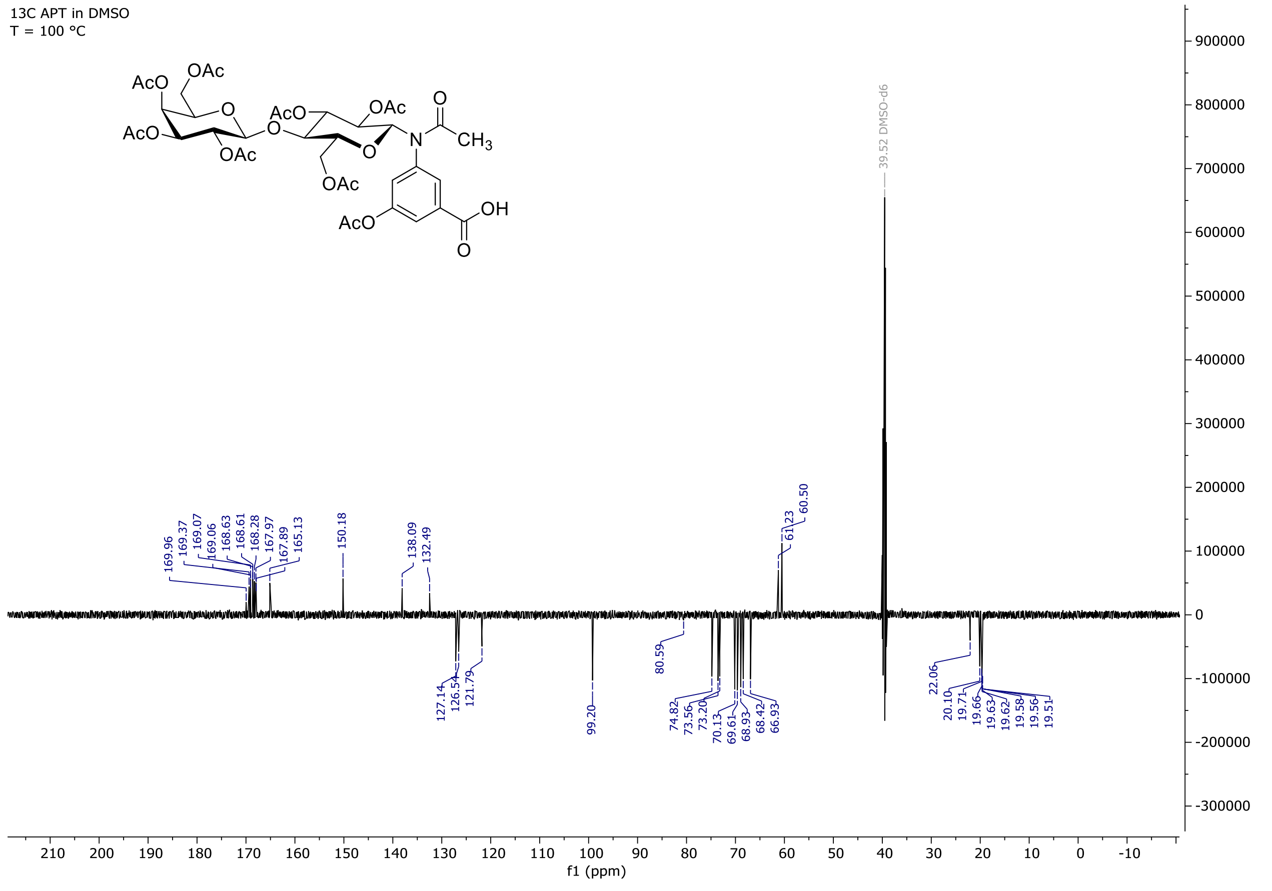

^{1}H, ^{13}C APT and HSQC NMR spectra of (2ae)

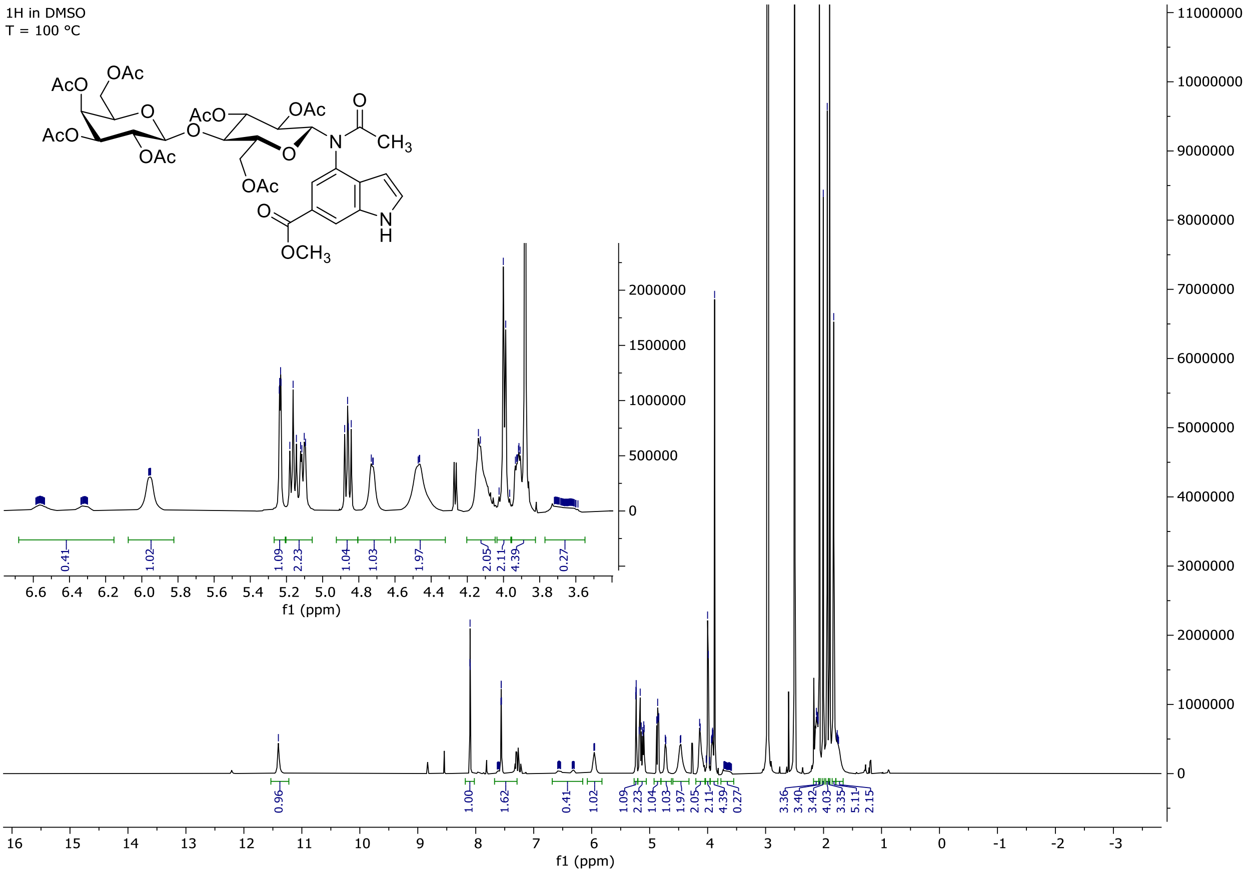

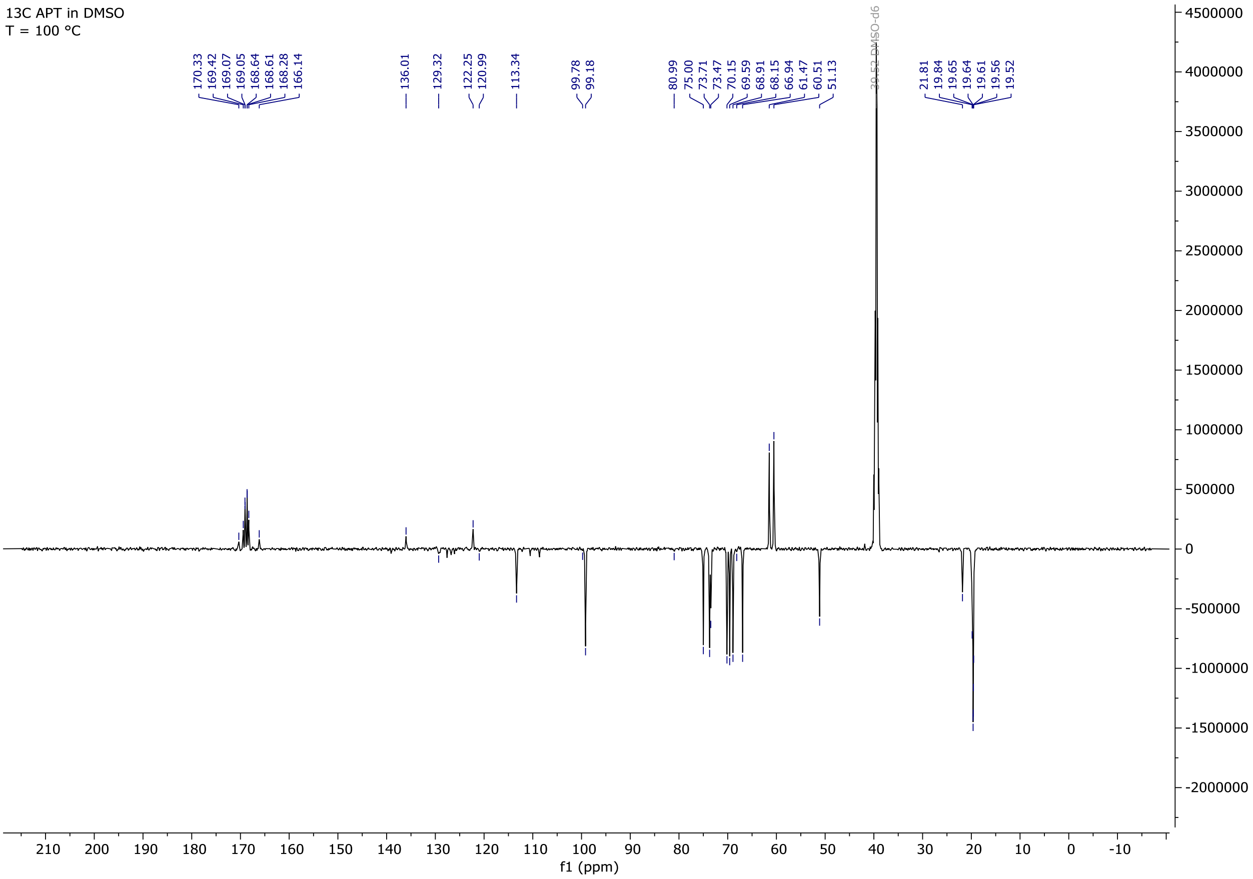

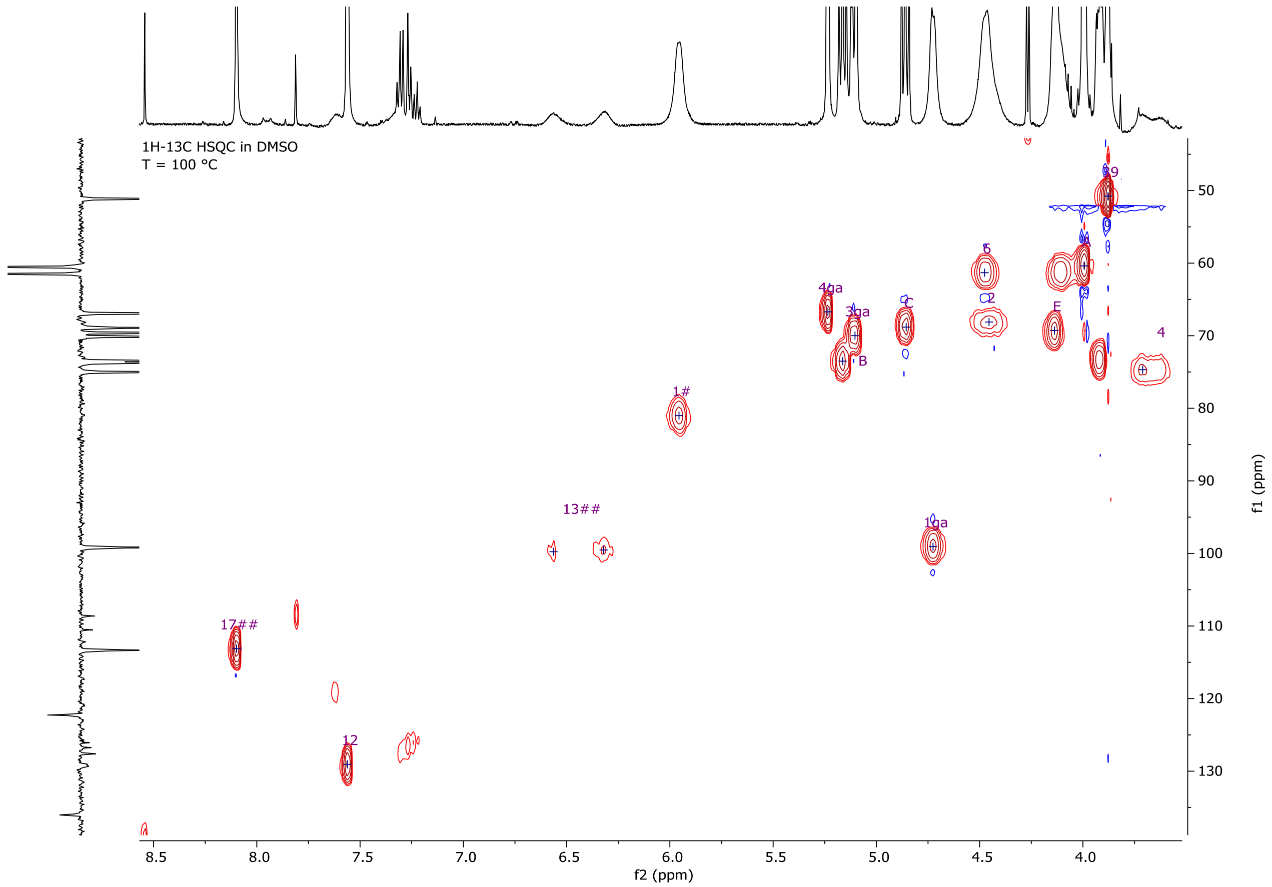
1H-13C HSQC in DMSO
T = 100 °C
f2 (ppm)
f1 (ppm)

^{1}H, ^{13}C APT and HSQC NMR spectra of (3a)

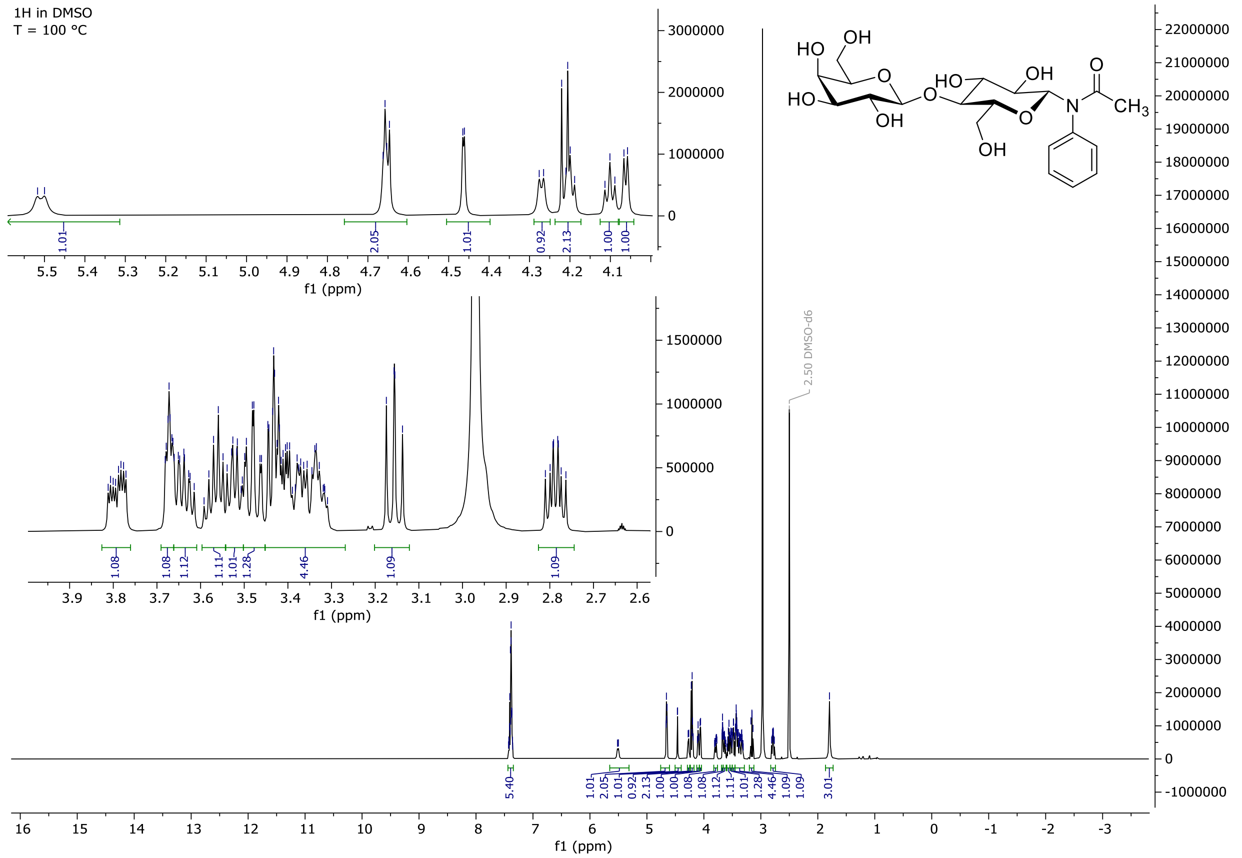

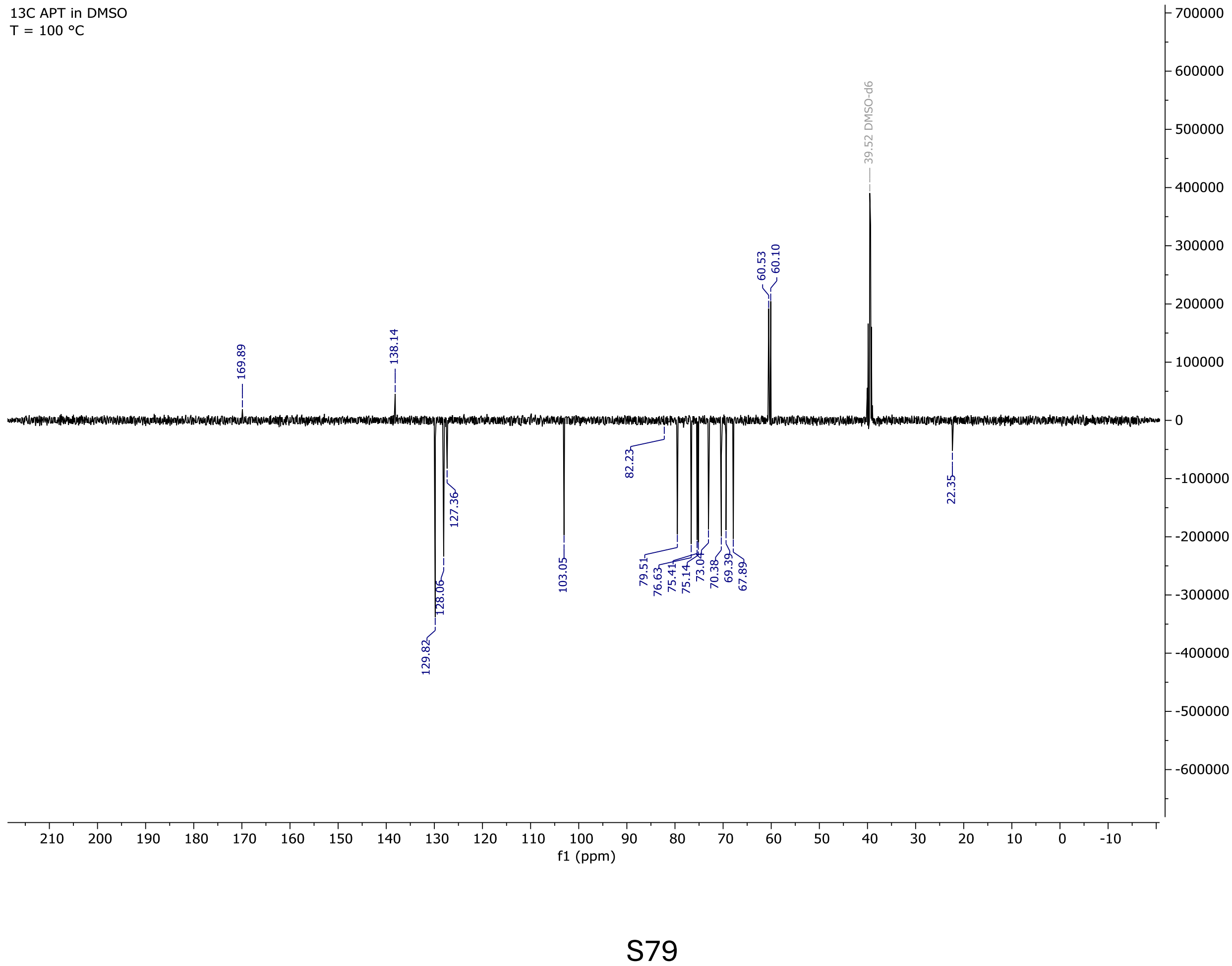

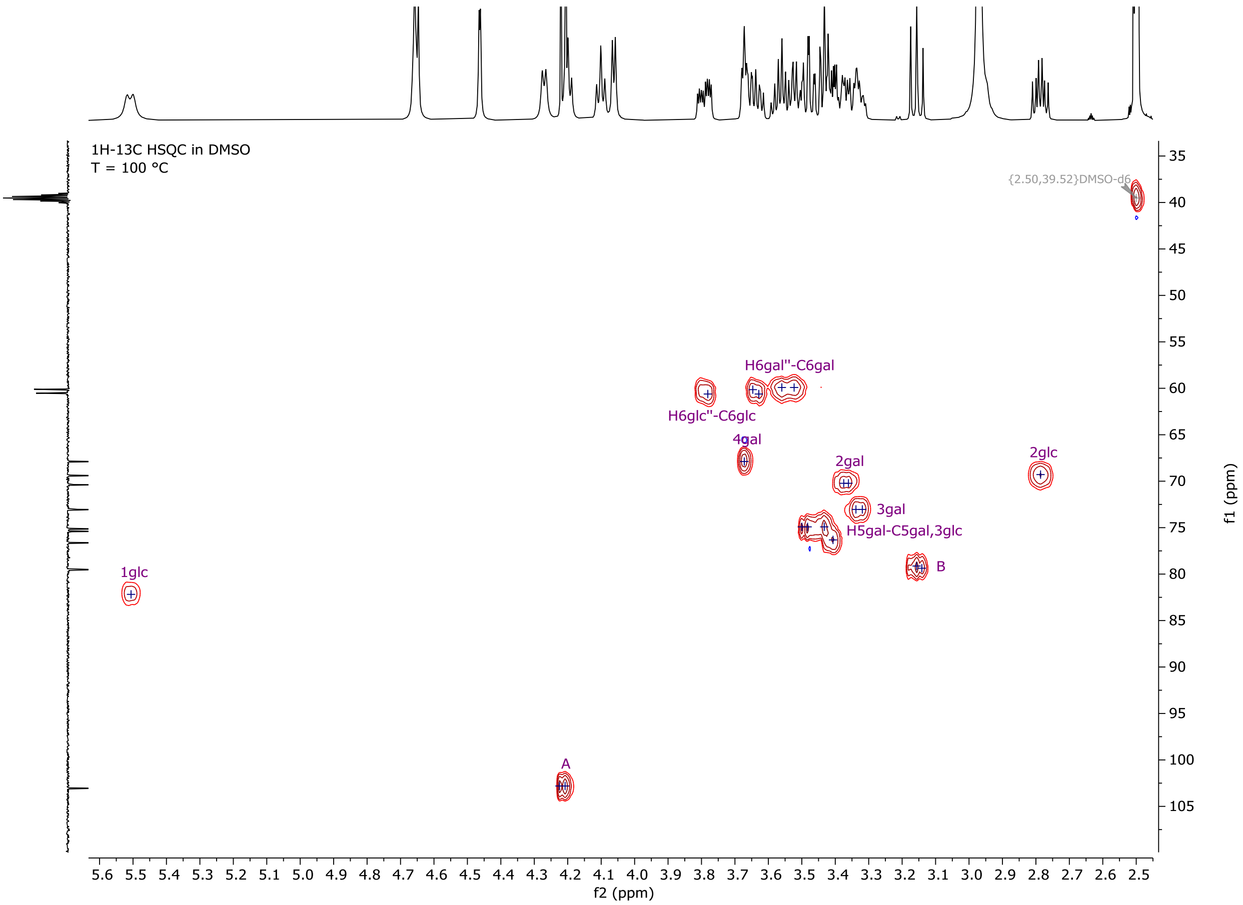
1H-13C HSQC in DMSO
T = 100 °C
{2.50,39.52}DMSO-d6
H6gal''-C6gal
H6glc''-C6glc
4gal
2gal
2glc
3gal
H5gal-C5gal,3glc
1glc
B
A
f1 (ppm)
f2 (ppm)

^{1}H and ^{13}C APT NMR spectra of (3b)

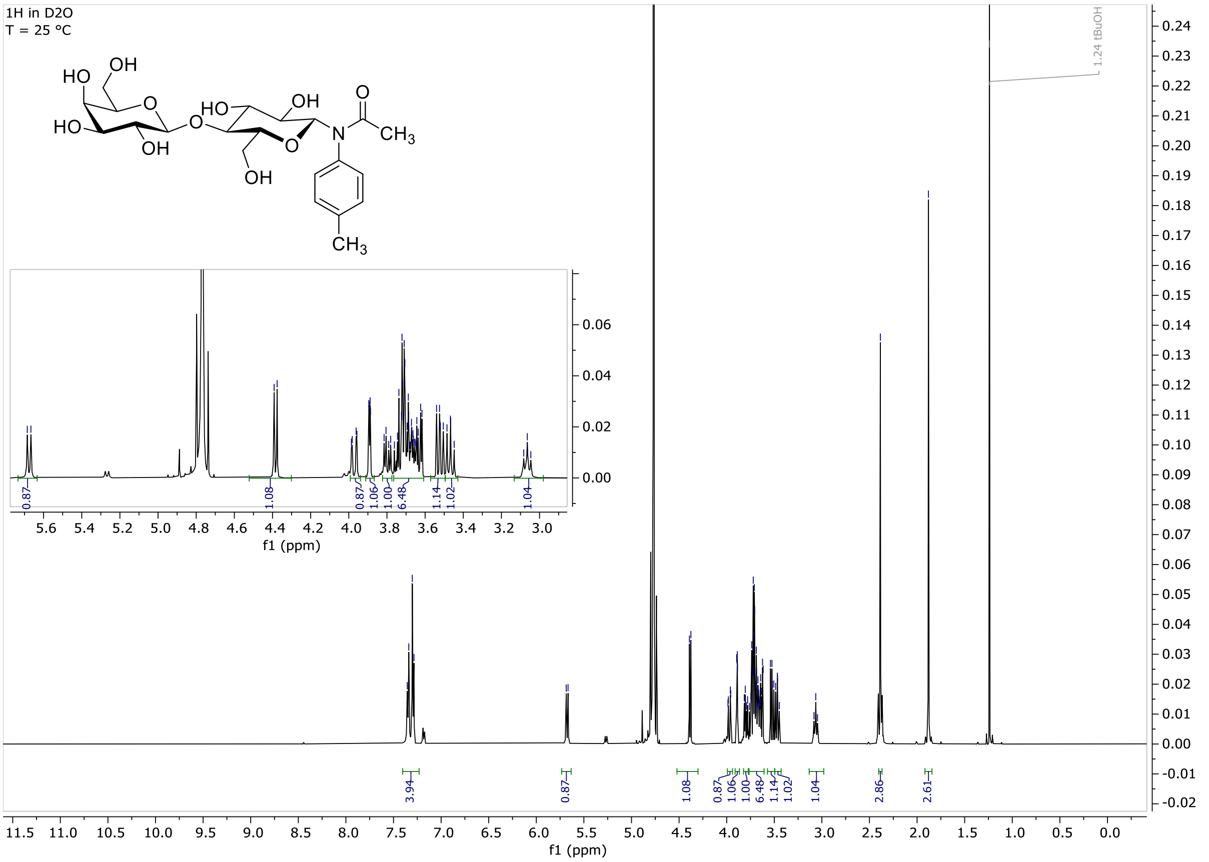

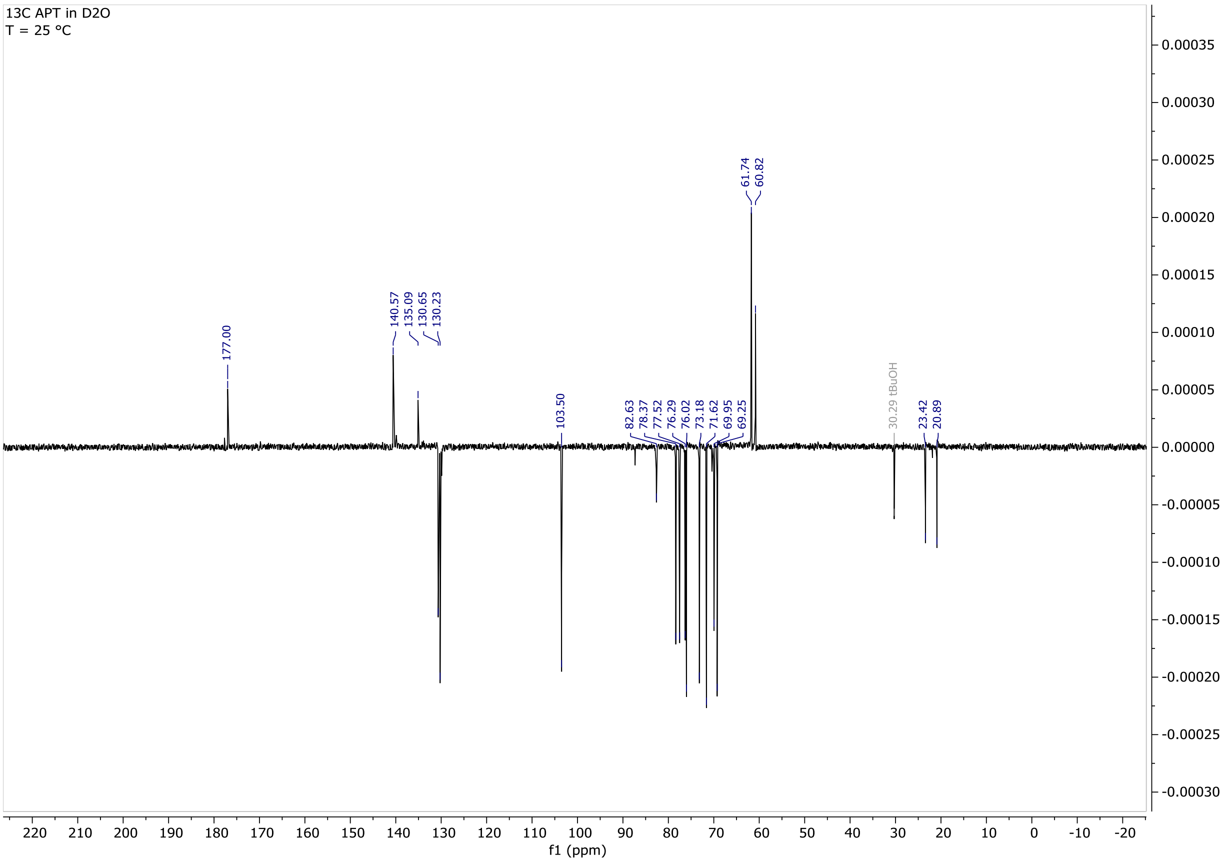

^{1}H, ^{13}C APT, and HSQC NMR spectra of (3c)

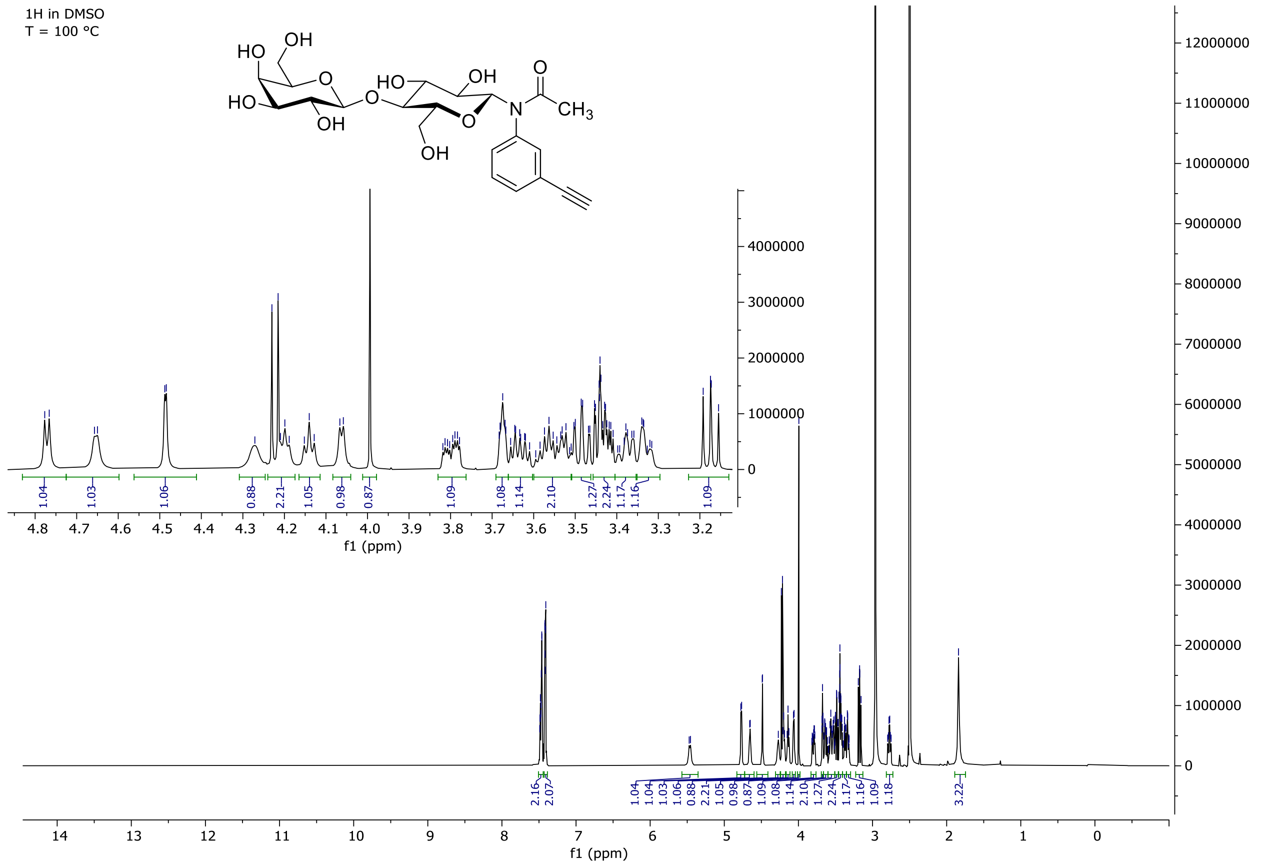

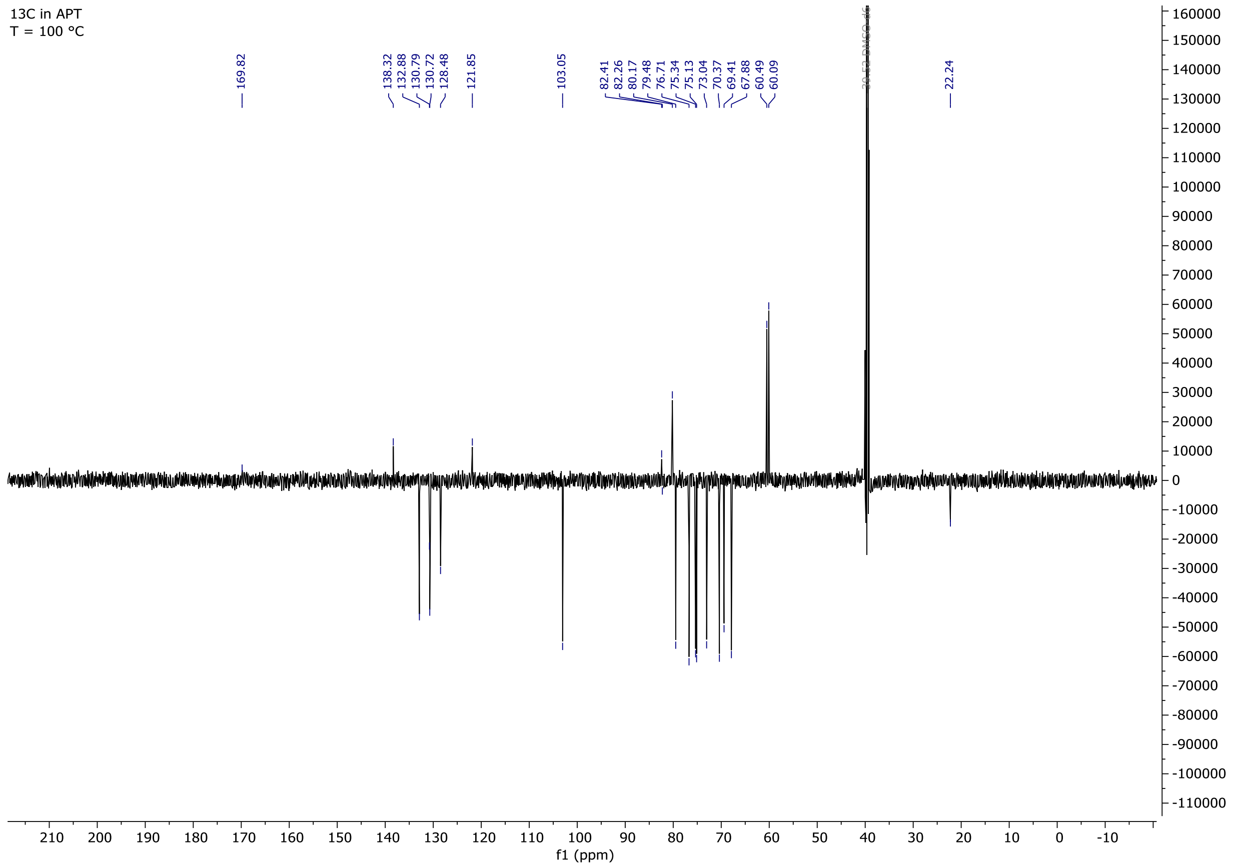

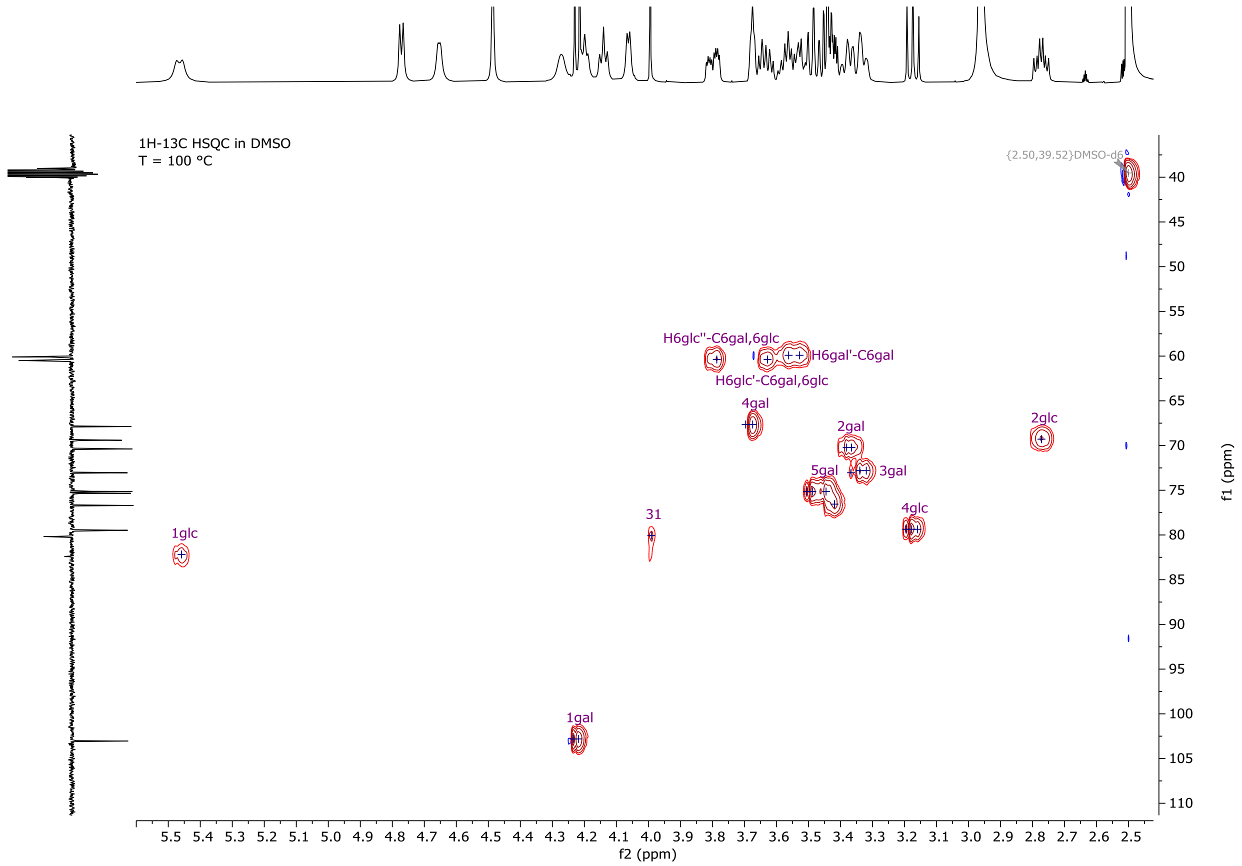

1H-13C HSQC in DMSO
T = 100 °C
{2.50,39.52}DMSO-d6
H6glc''-C6gal,6glc
H6gal'-C6gal
H6glc'-C6gal,6glc
4gal
2gal
2glc
5gal
3gal
31
4glc
1glc
1gal
f2 (ppm)
f1 (ppm)

^{1}H, ^{13}C APT and ^{19}F NMR spectra of (3d)

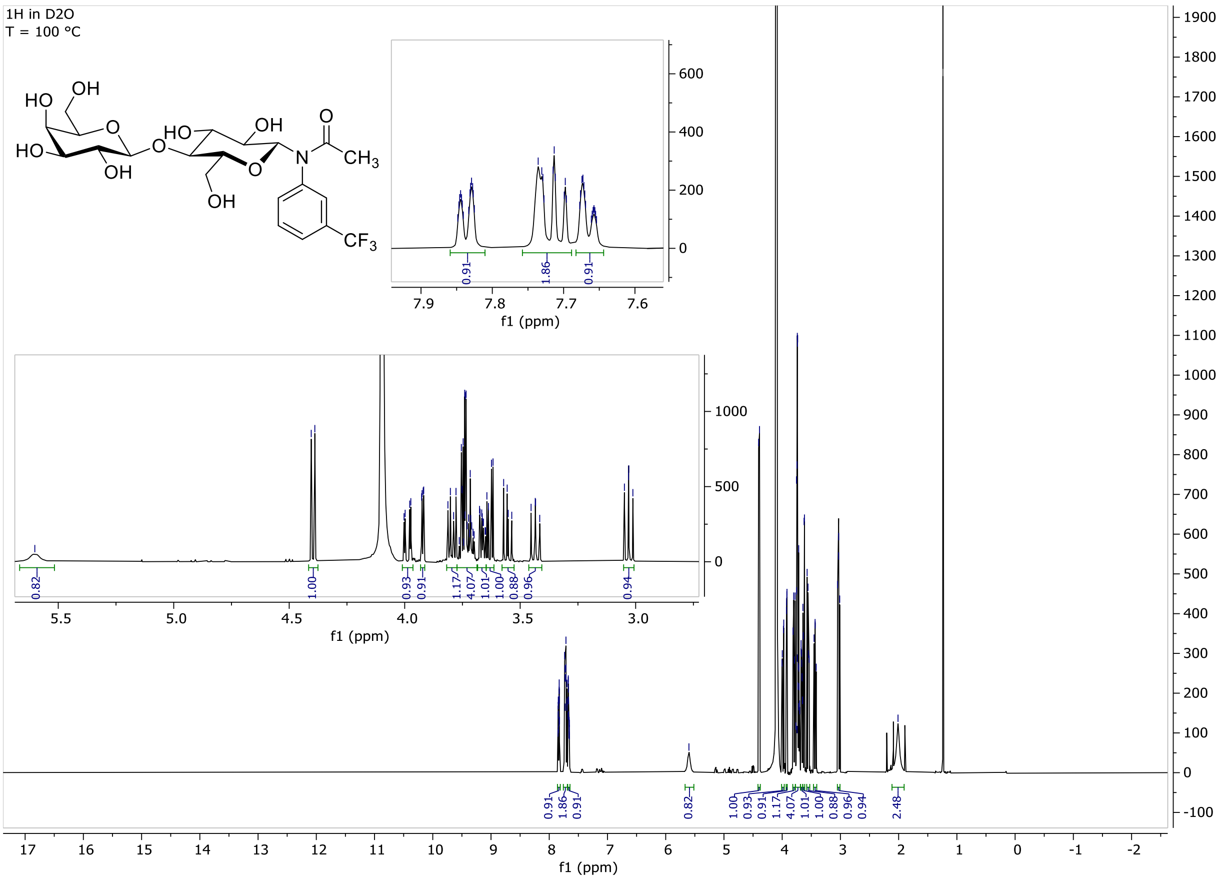

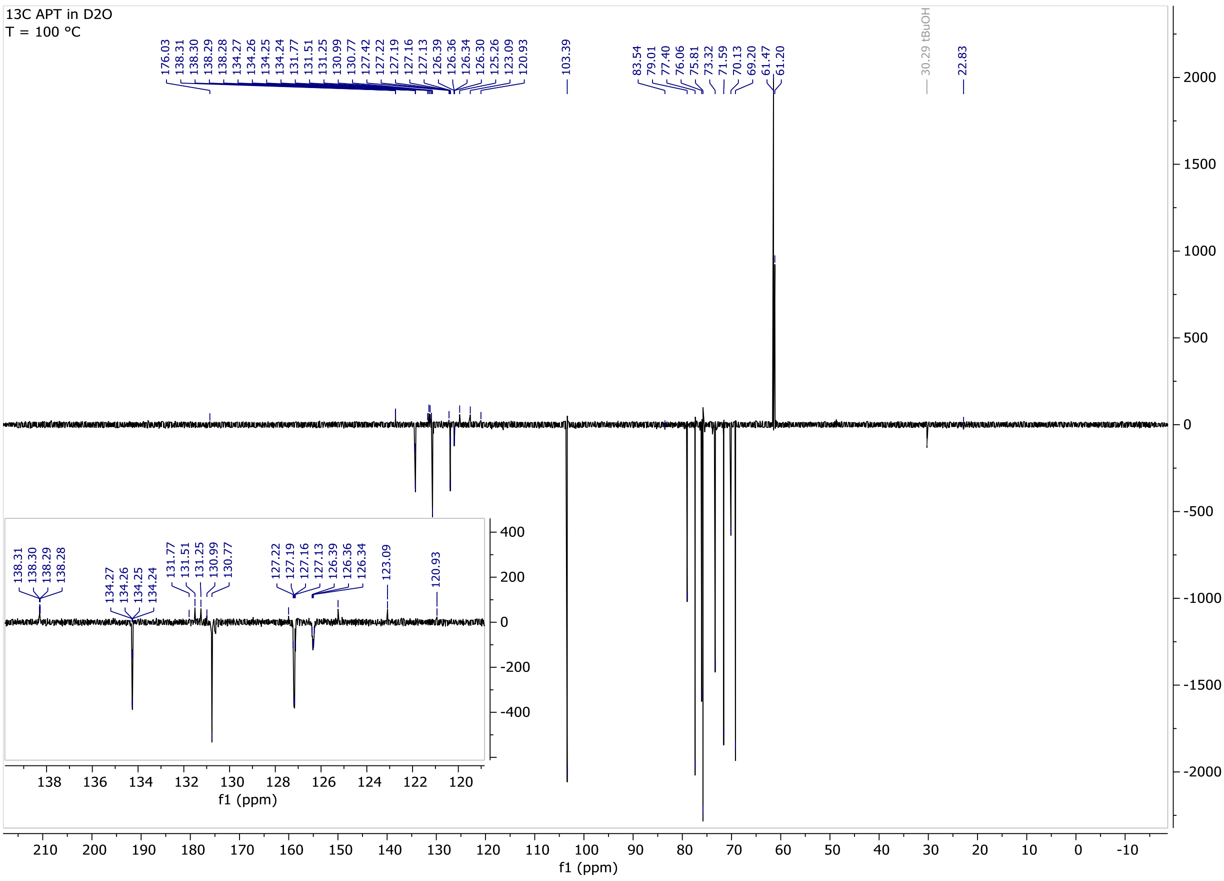

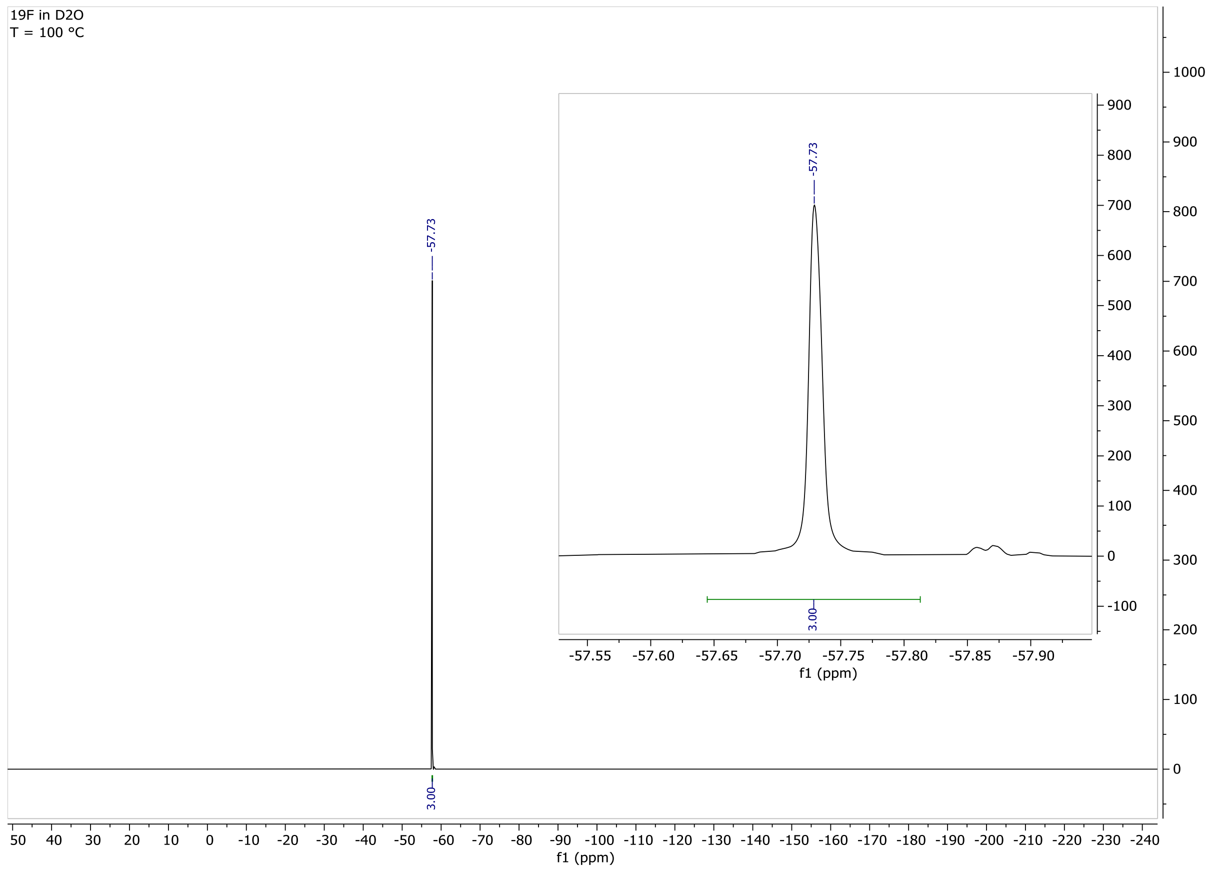

19F in D2O
T = 100 °C
-57.73
3.00
f1 (ppm)

^{1}H, ^{13}C APT, HSQC and ^{19}F NMR spectra of (3e)

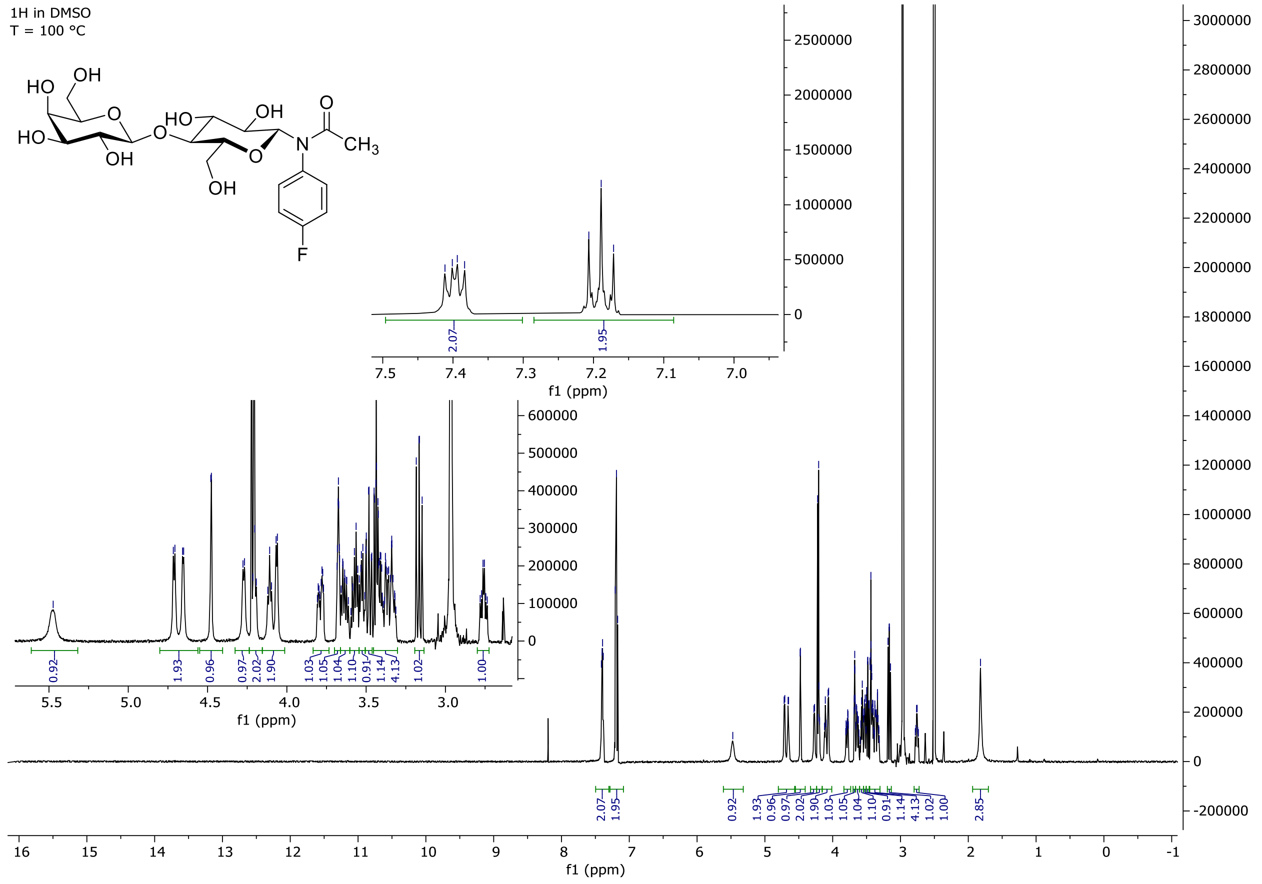

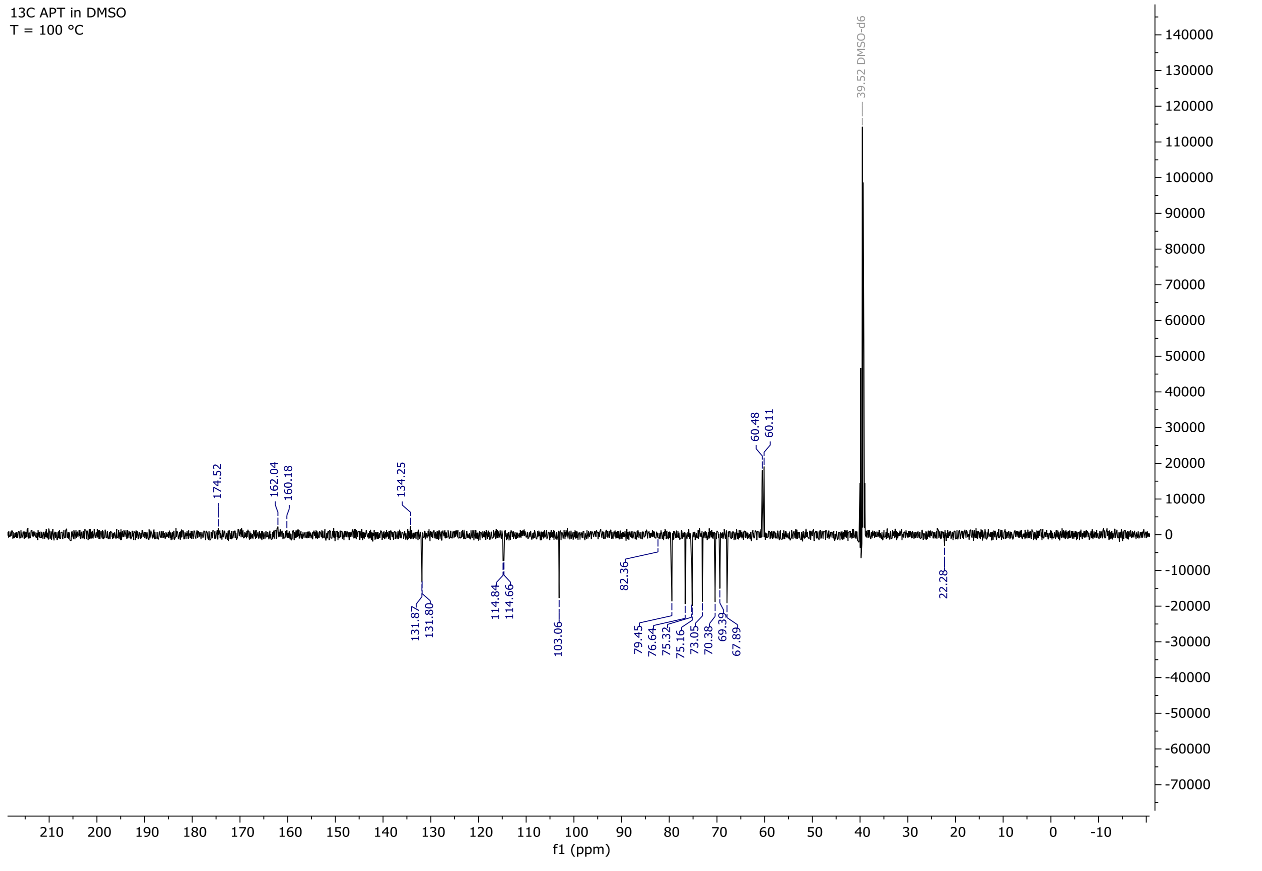

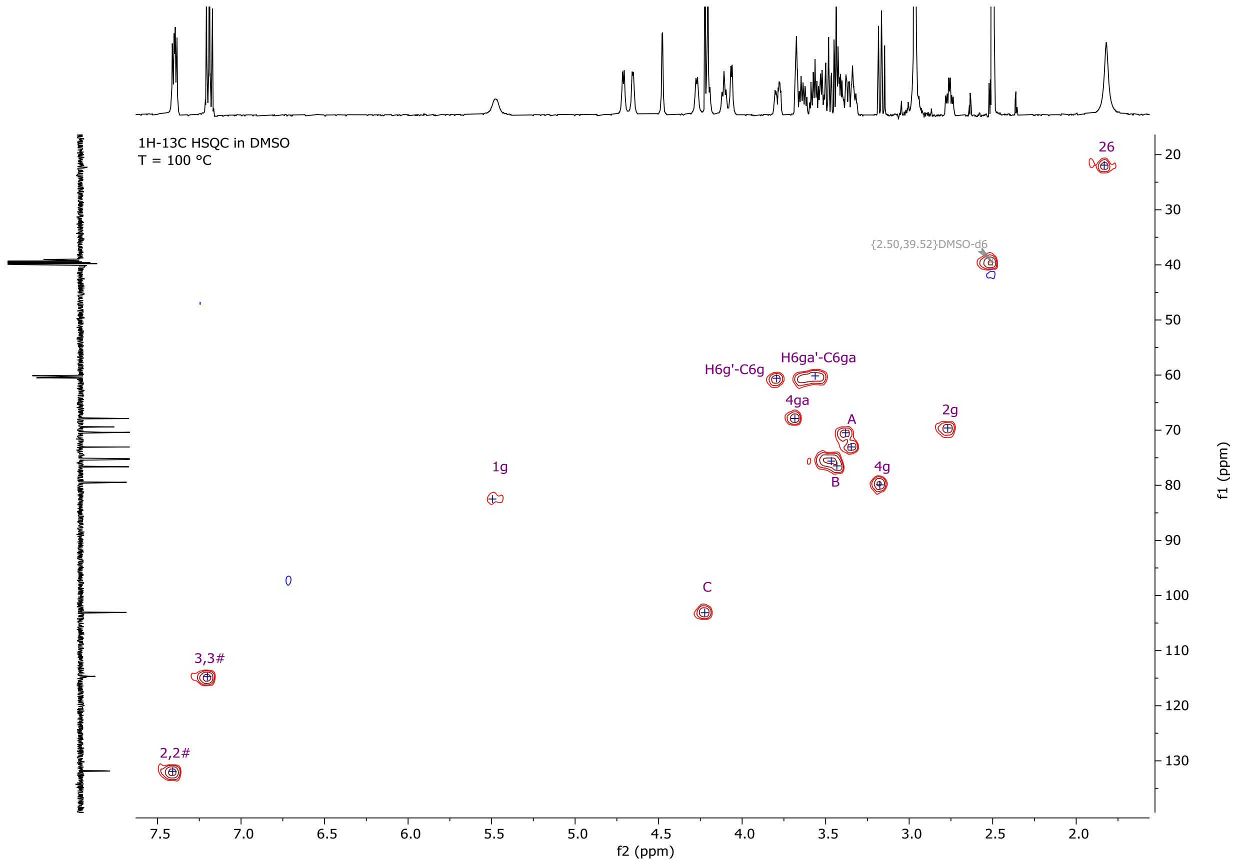
1H-13C HSQC in DMSO
T = 100 °C
26
{2.50,39.52}DMSO-d6
H6ga'-C6ga
H6g'-C6g
4ga
2g
A
1g
4g
B
C
3,3#
2,2#
f2 (ppm)
f1 (ppm)

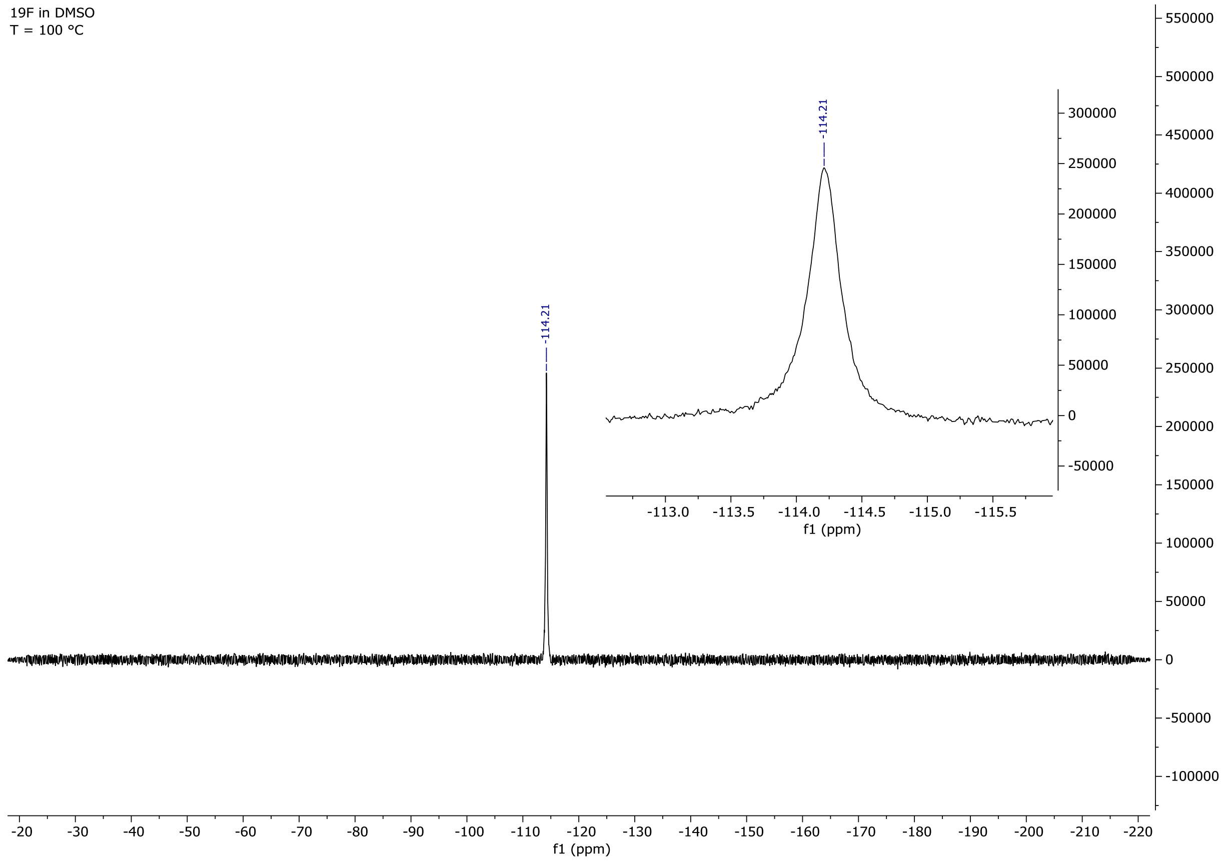
19F in DMSO
T = 100 °C
-114.21
-114.21
f1 (ppm)
f1 (ppm)

^{1}H, ^{13}C APT and ^{19}F NMR spectra of (3f)

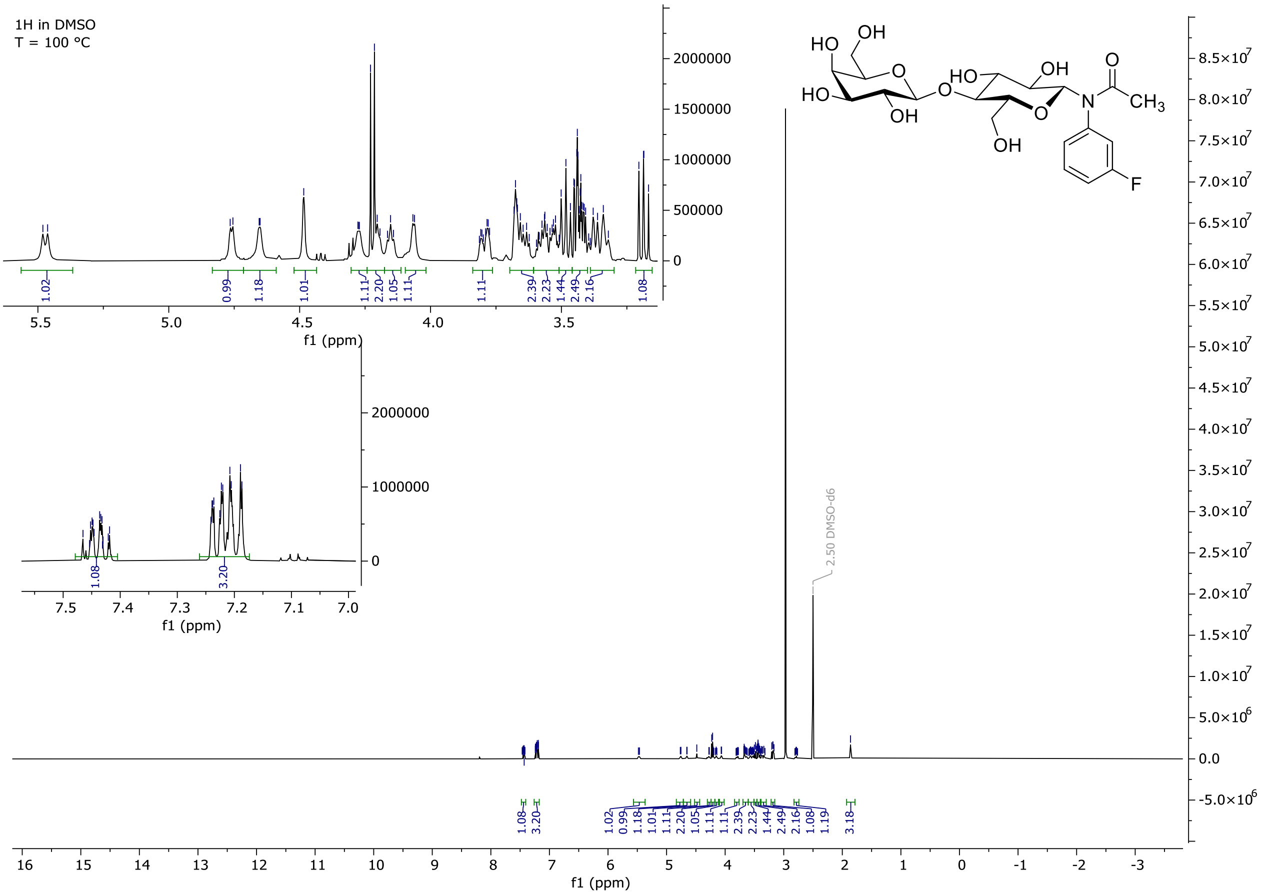

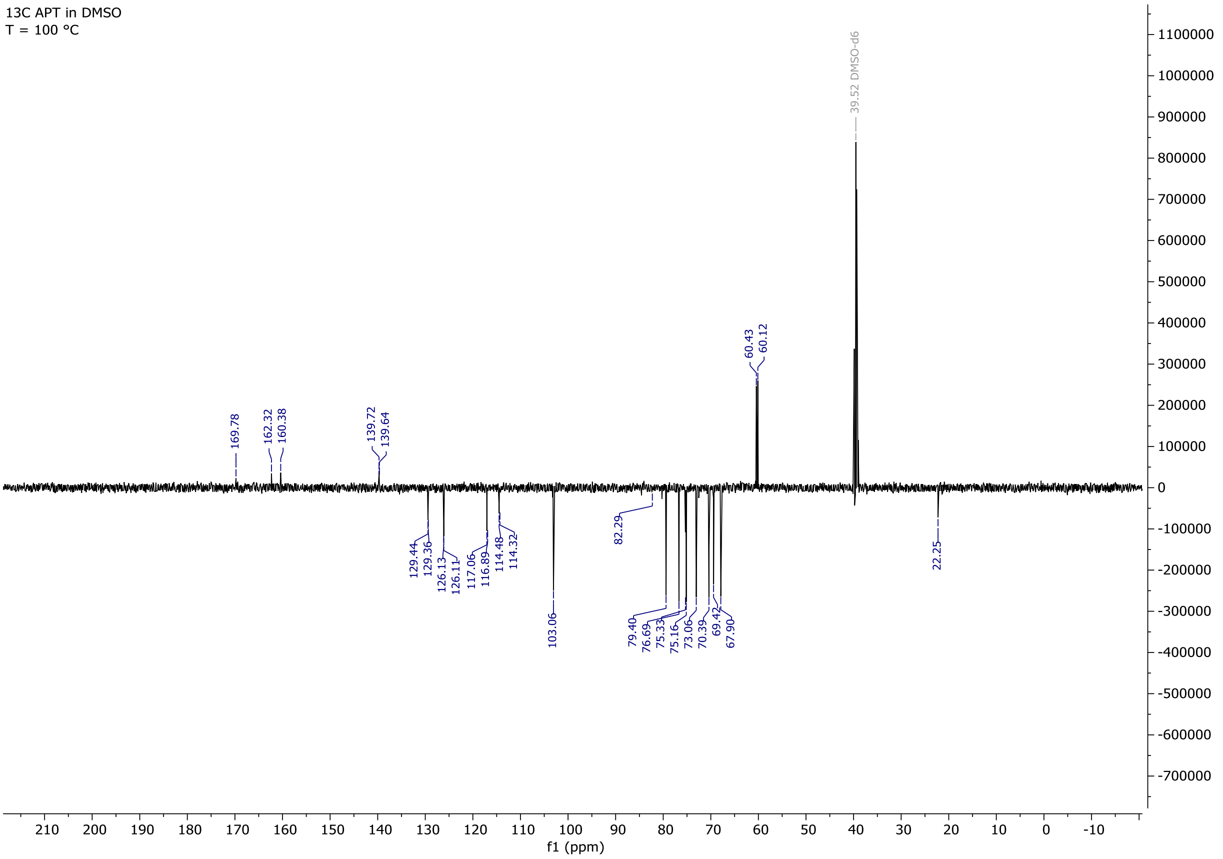

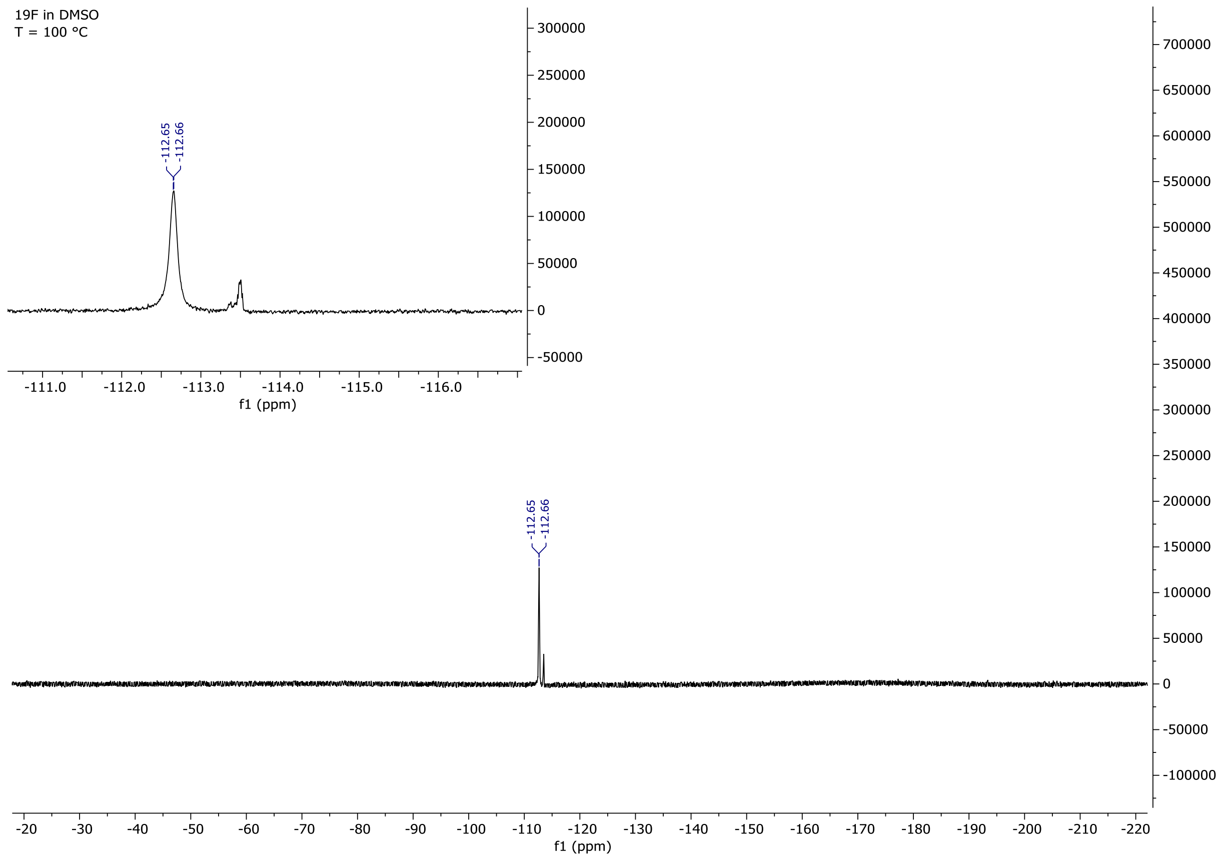
19F in DMSO
T = 100 °C
-112.65
-112.66
f1 (ppm)

^{1}H, ^{13}C APT and ROESY NMR spectra of (3g)

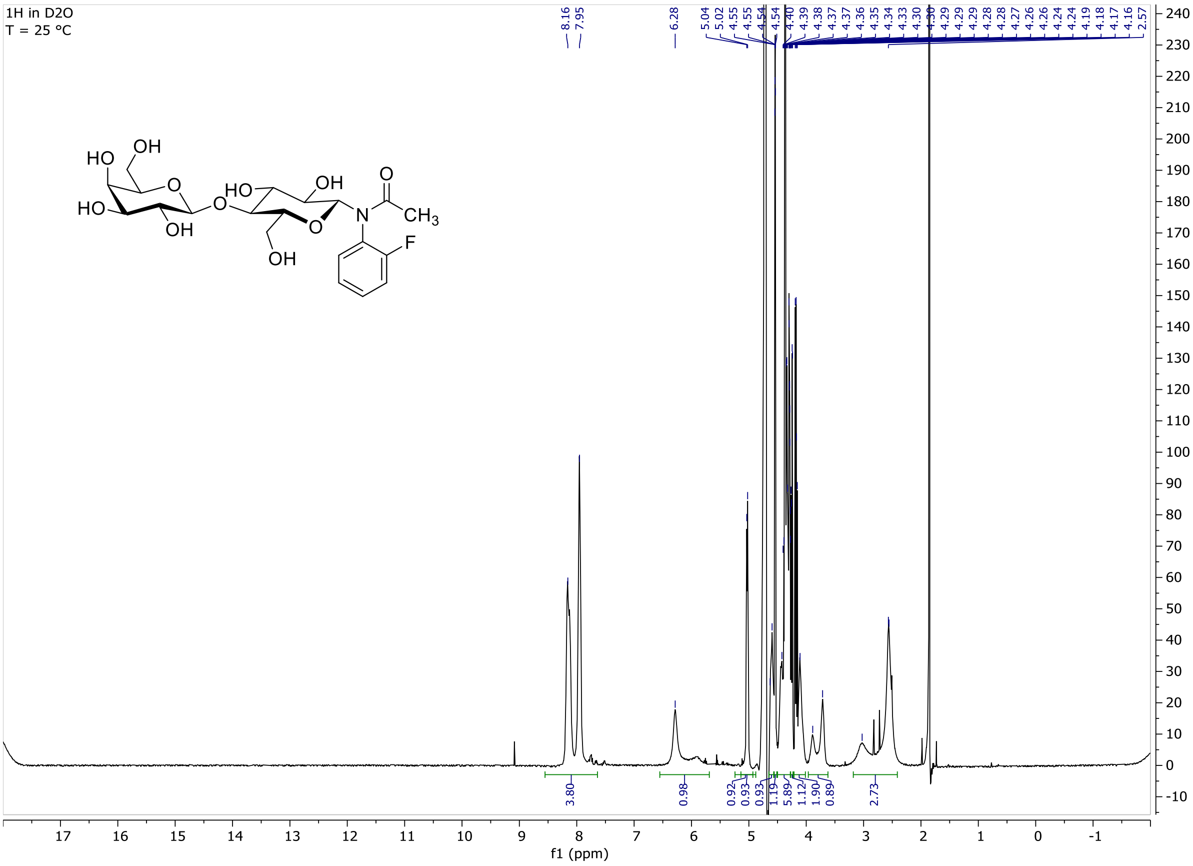

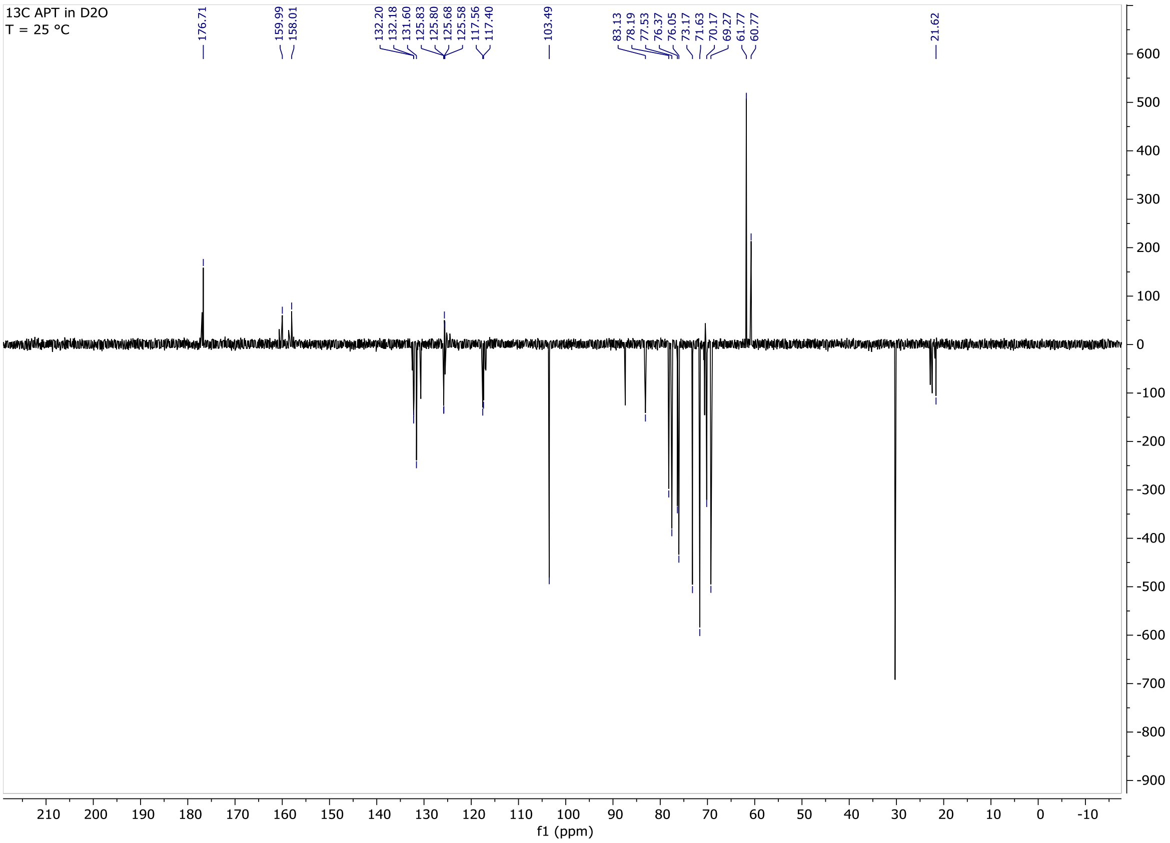

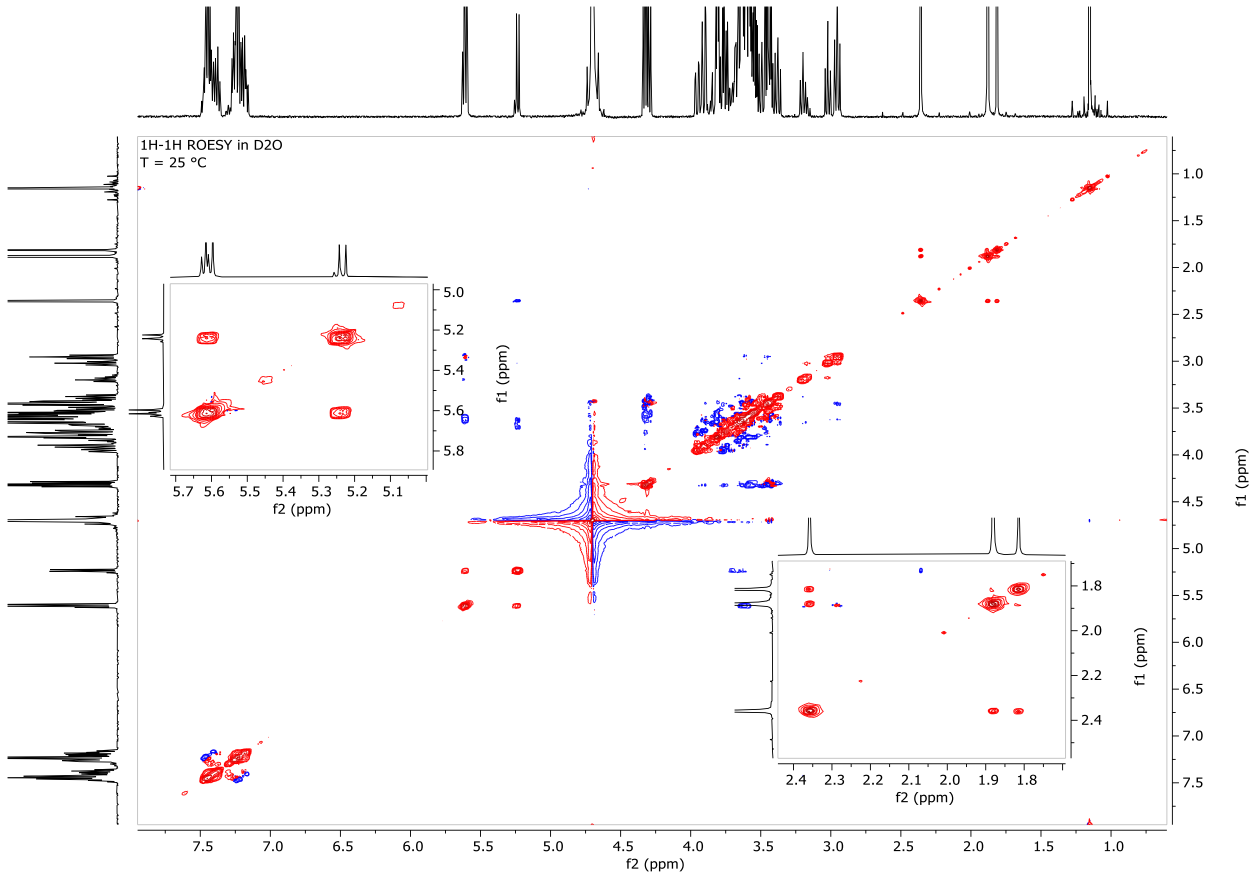

1H-1H ROESY in D2O
T = 25 °C
f2 (ppm)
f1 (ppm)

^{1}H, ^{13}C APT, ^{19}F and HSQC NMR spectra of (3h)

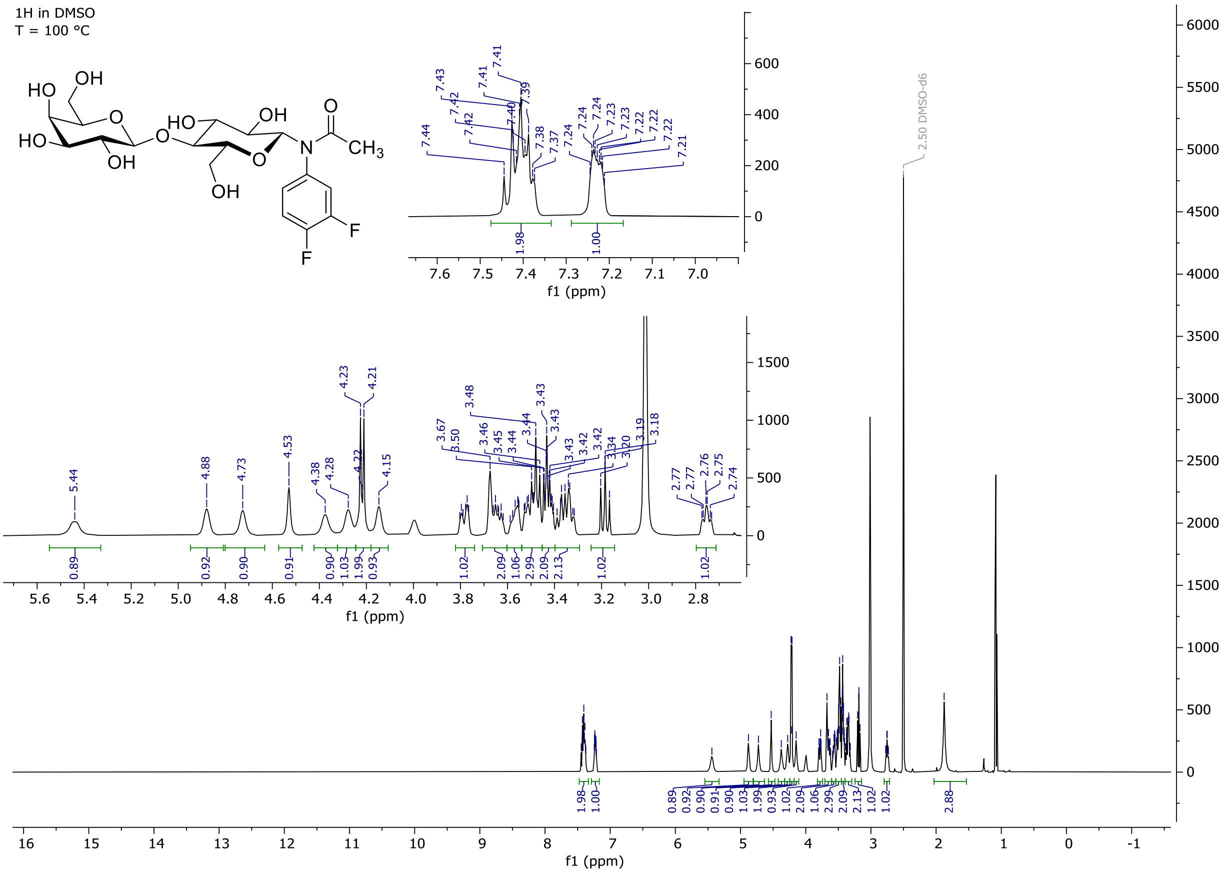

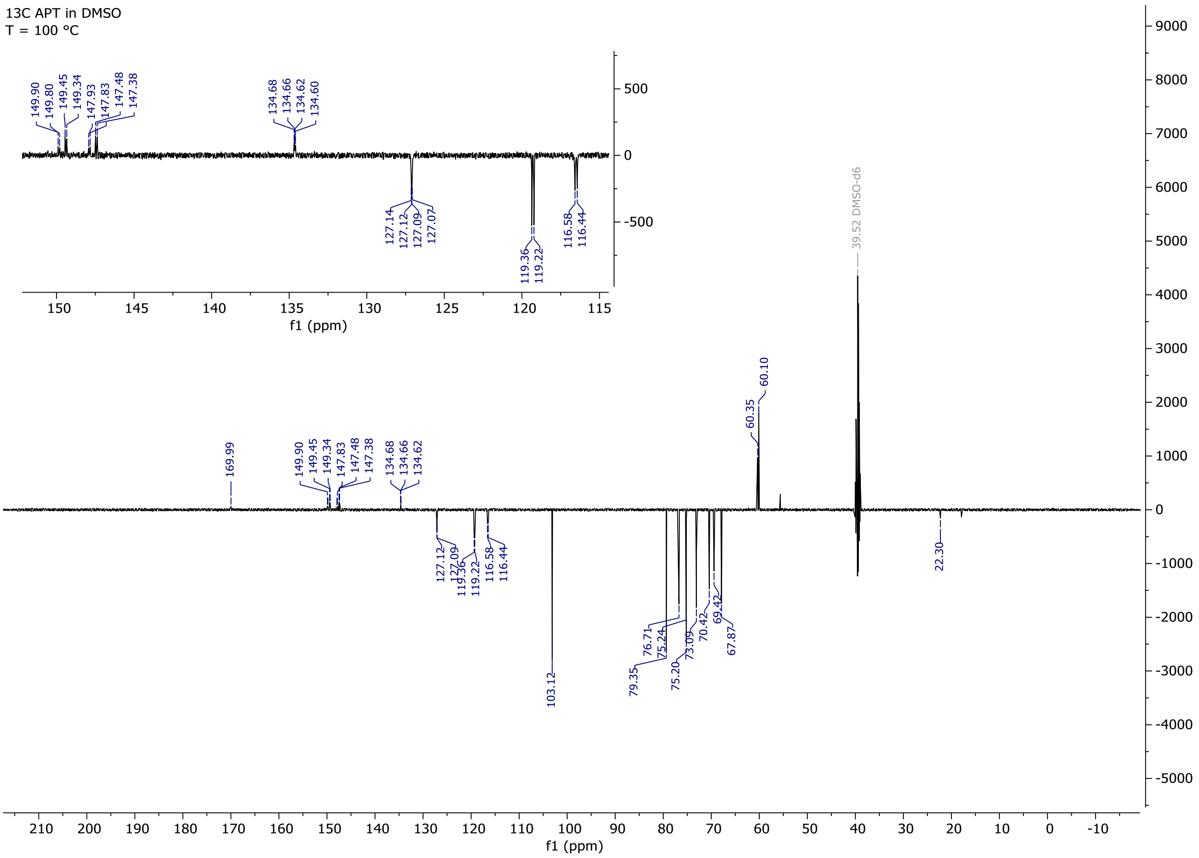

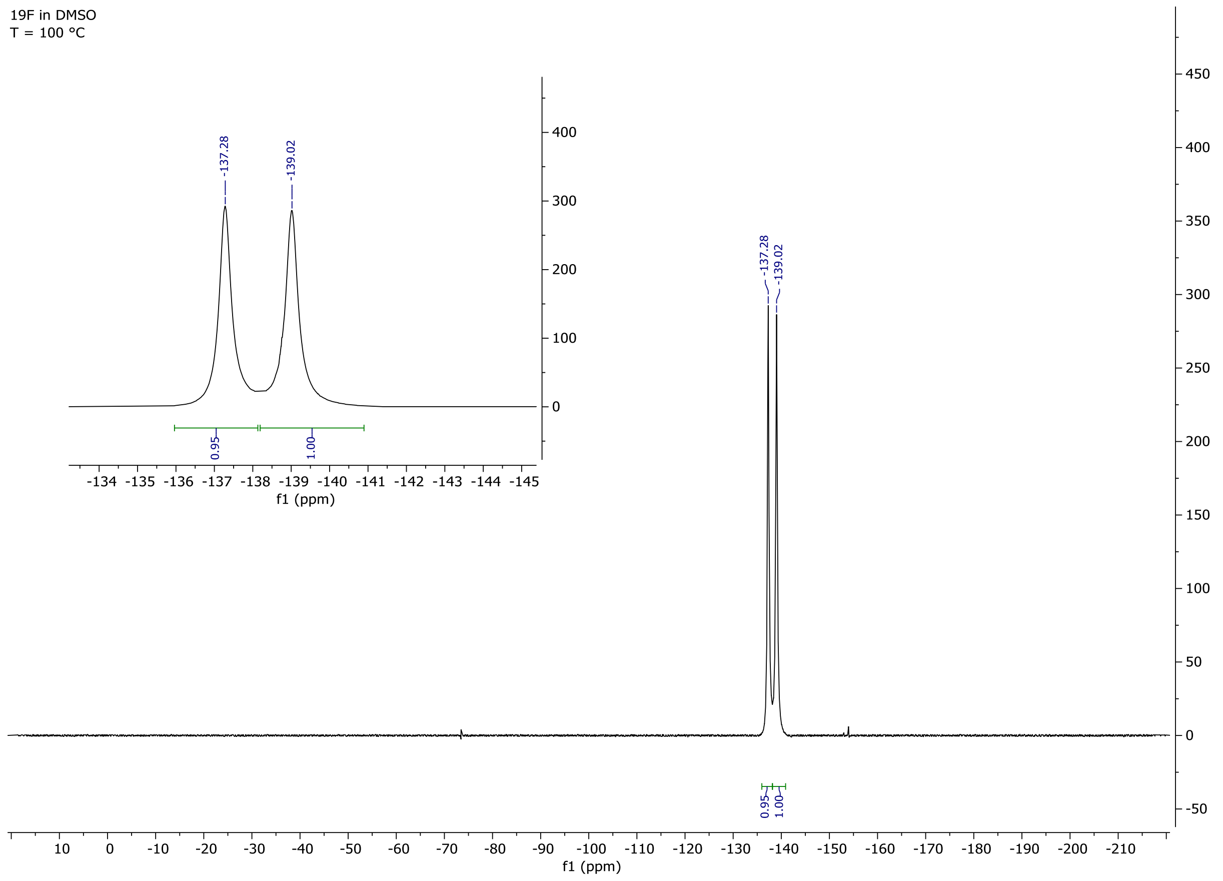
19F in DMSO
T = 100 °C
-137.28
-139.02
0.95
1.00
f1 (ppm)

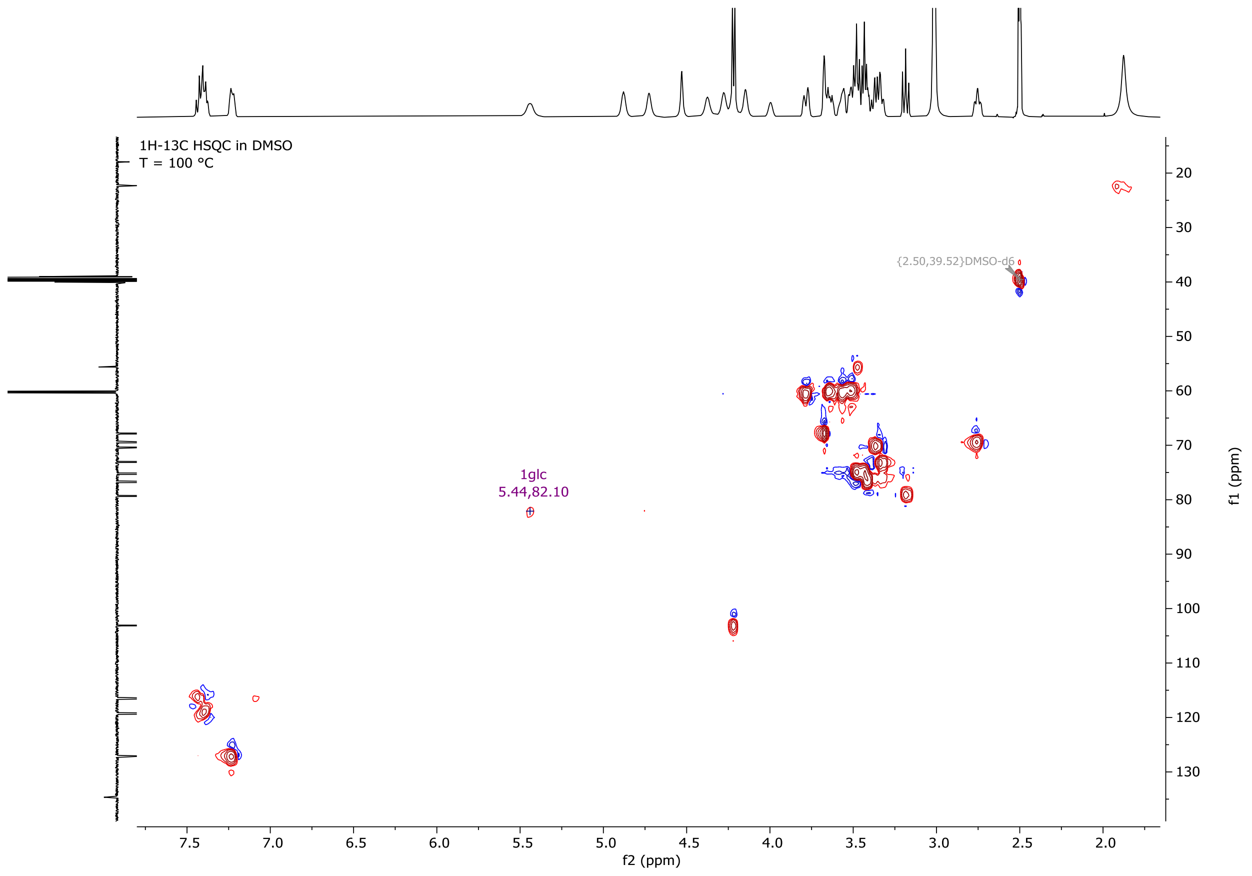
1H-13C HSQC in DMSO
T = 100 °C
{2.50,39.52}DMSO-d6
1glc
5.44,82.10
f2 (ppm)
f1 (ppm)

^{1}H, ^{13}C APT, ^{19}F and HSQC NMR spectra of (3i)

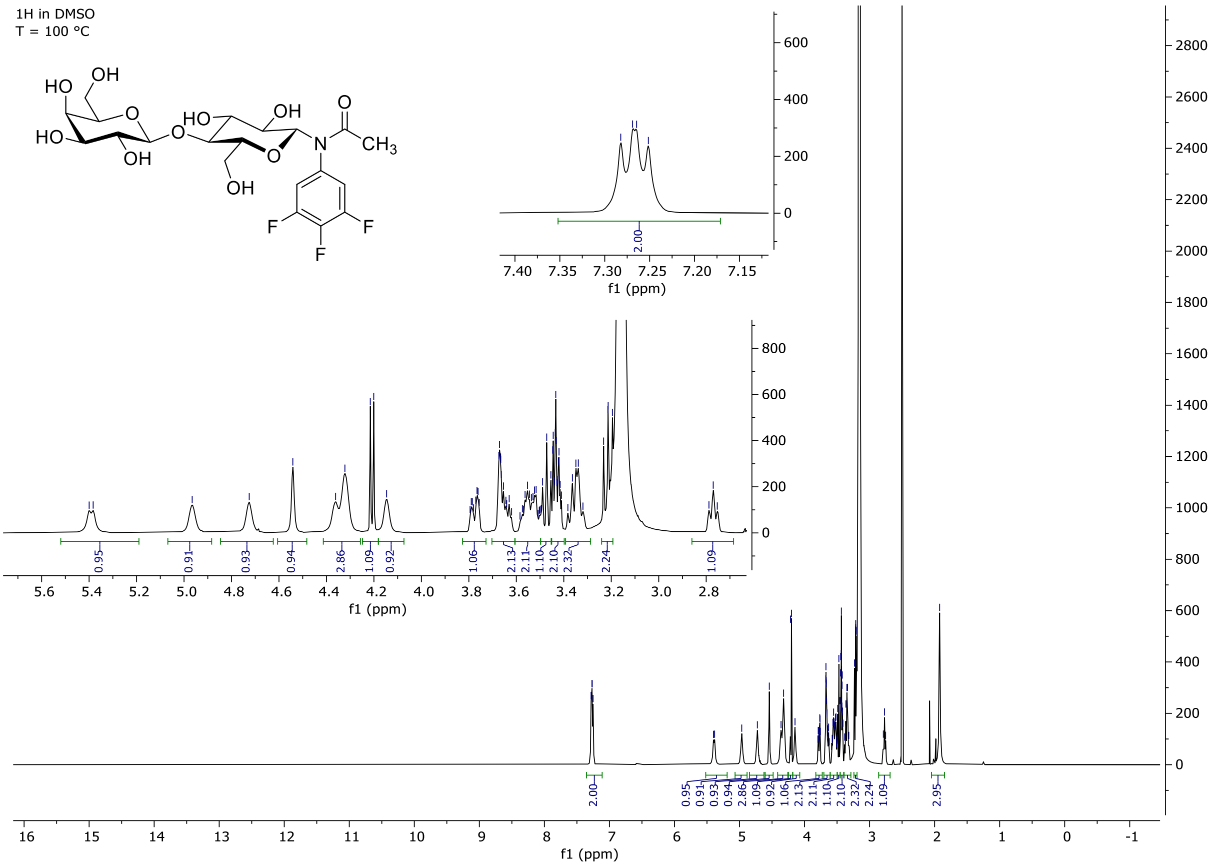

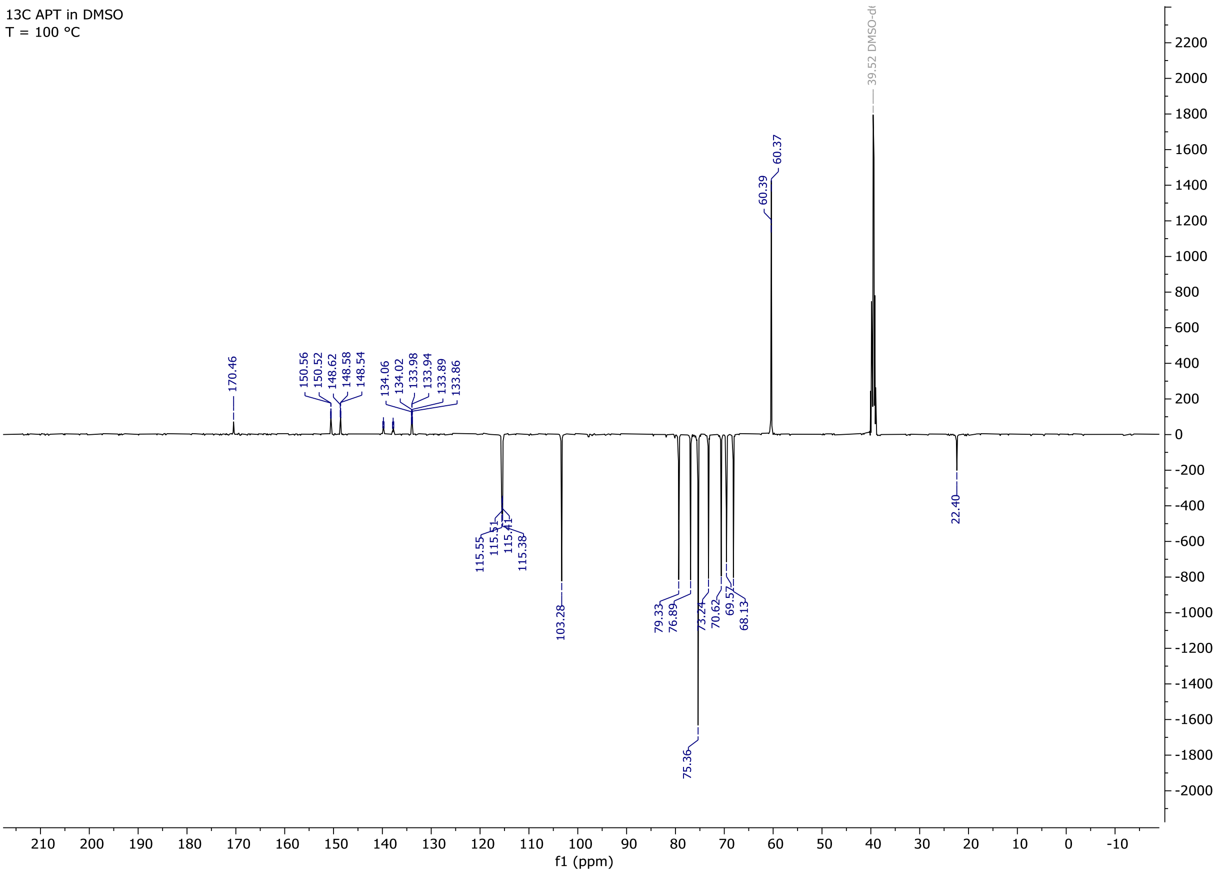

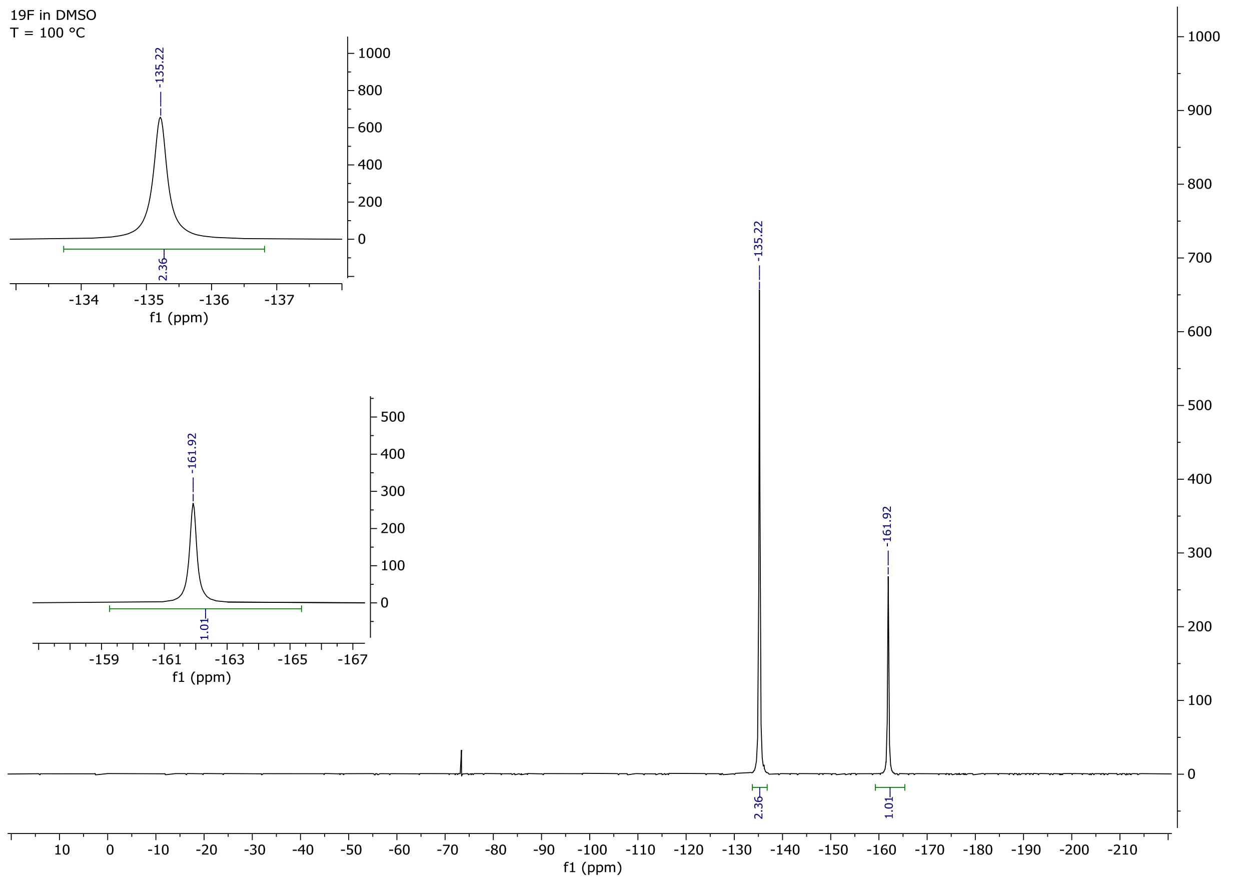

19F in DMSO
T = 100 °C
-135.22
-161.92
2.36
1.01
f1 (ppm)

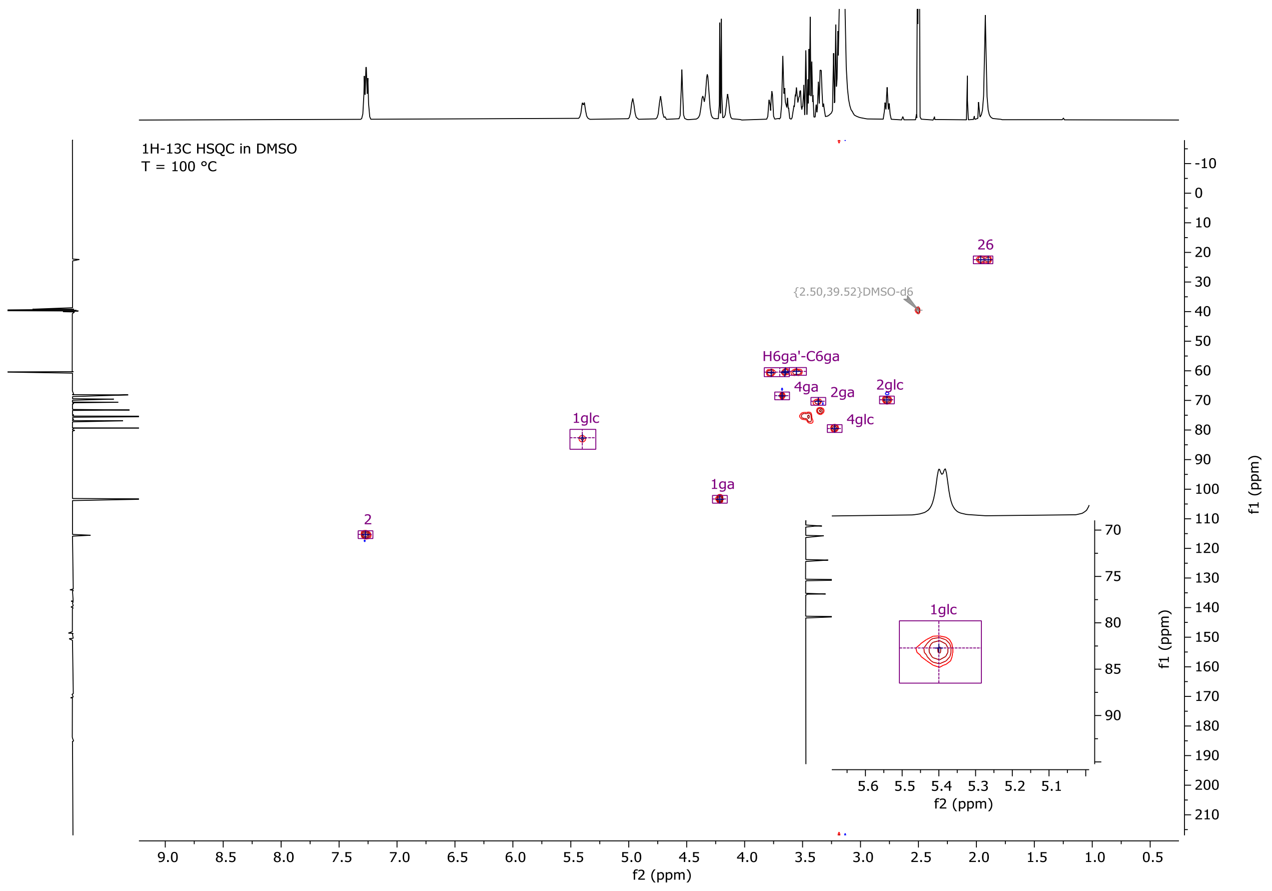

1H-13C HSQC in DMSO
T = 100 °C
26
{2.50,39.52}DMSO-d6
H6ga'-C6ga
4ga
2ga
2glc
1glc
4glc
1ga
2
f2 (ppm)
f1 (ppm)

^{1}H, ^{13}C APT and ROESY spectra of (3j)

1H in D2O
T = 25 °C

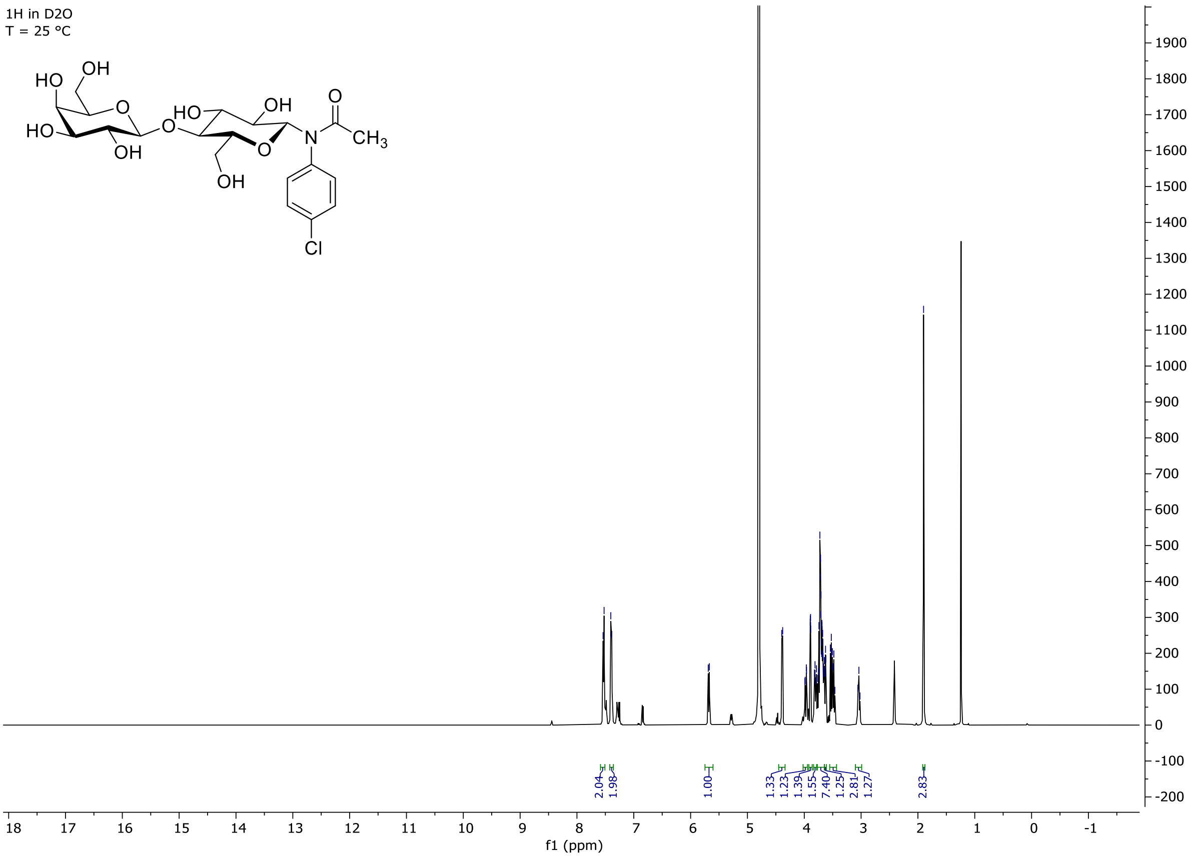

13C APT in D2O
T = 25 °C

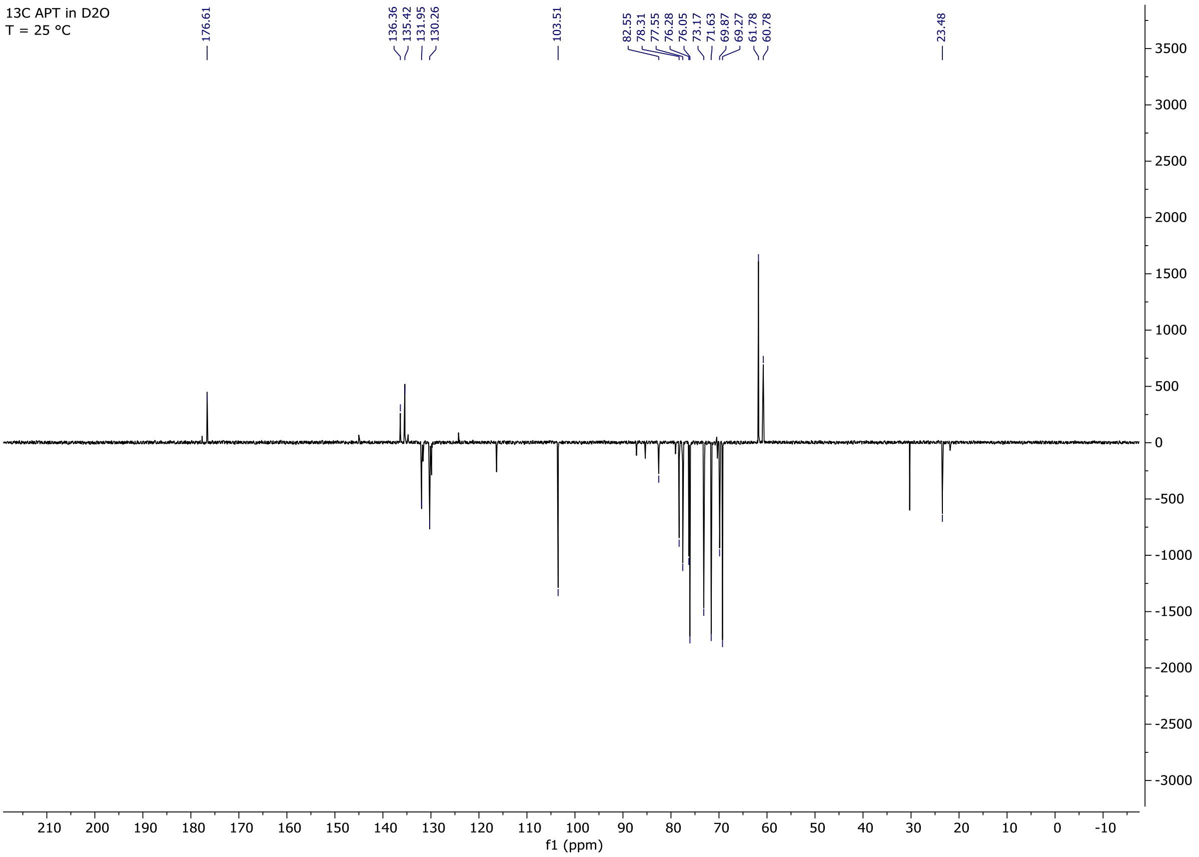

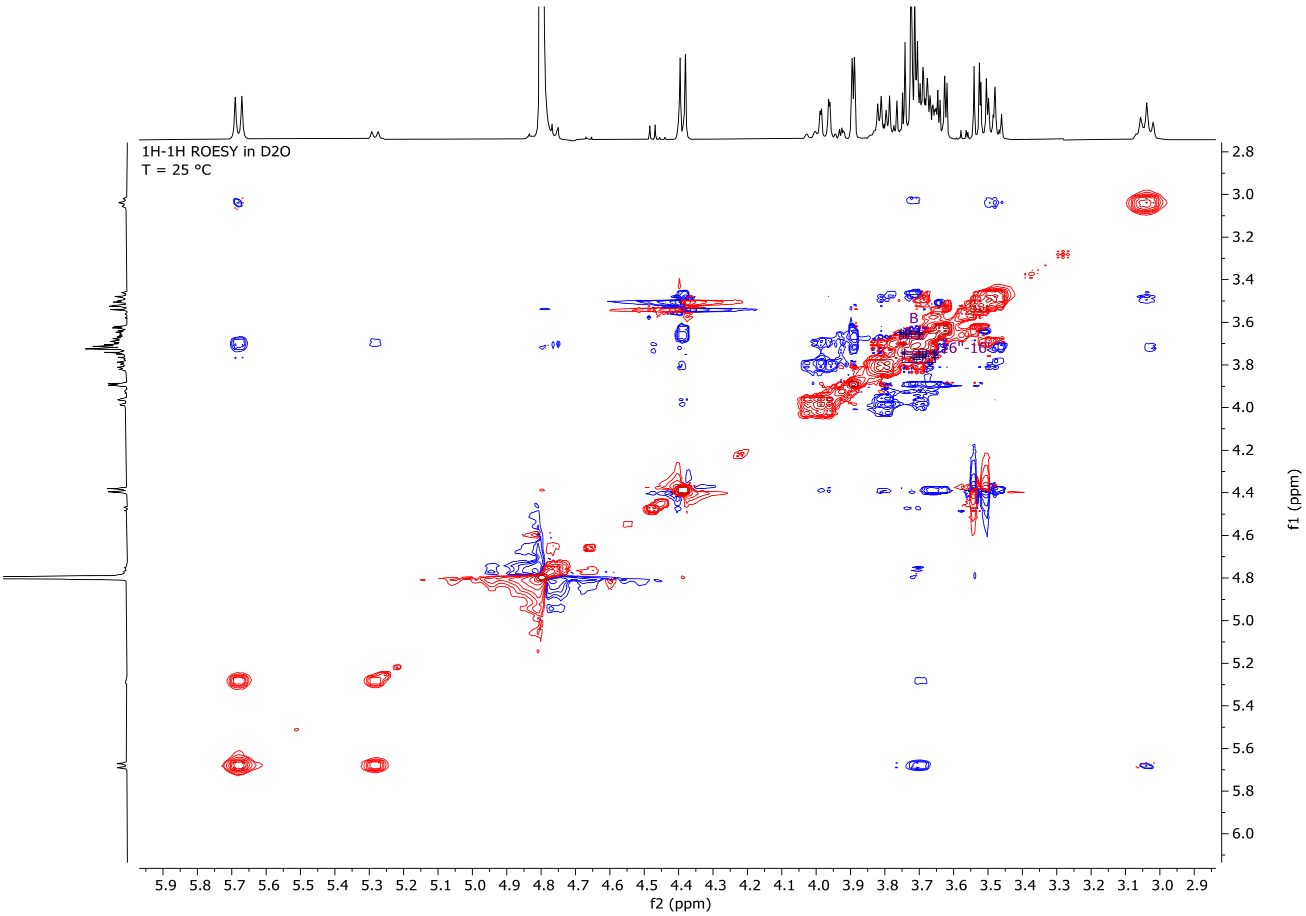
1H-1H ROESY in D2O
T = 25 °C
f2 (ppm)
f1 (ppm)

^{1}H and ^{13}C APT NMR spectra of (3k)

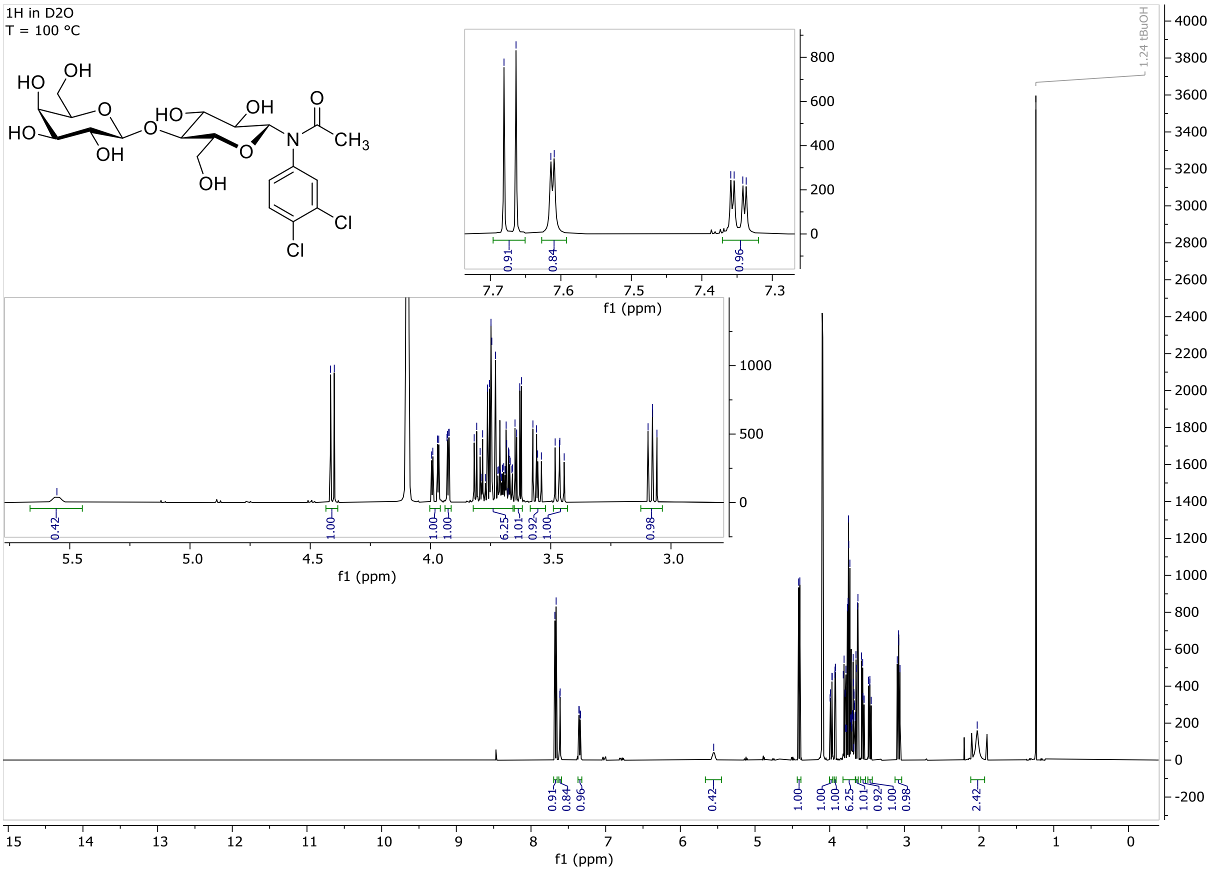

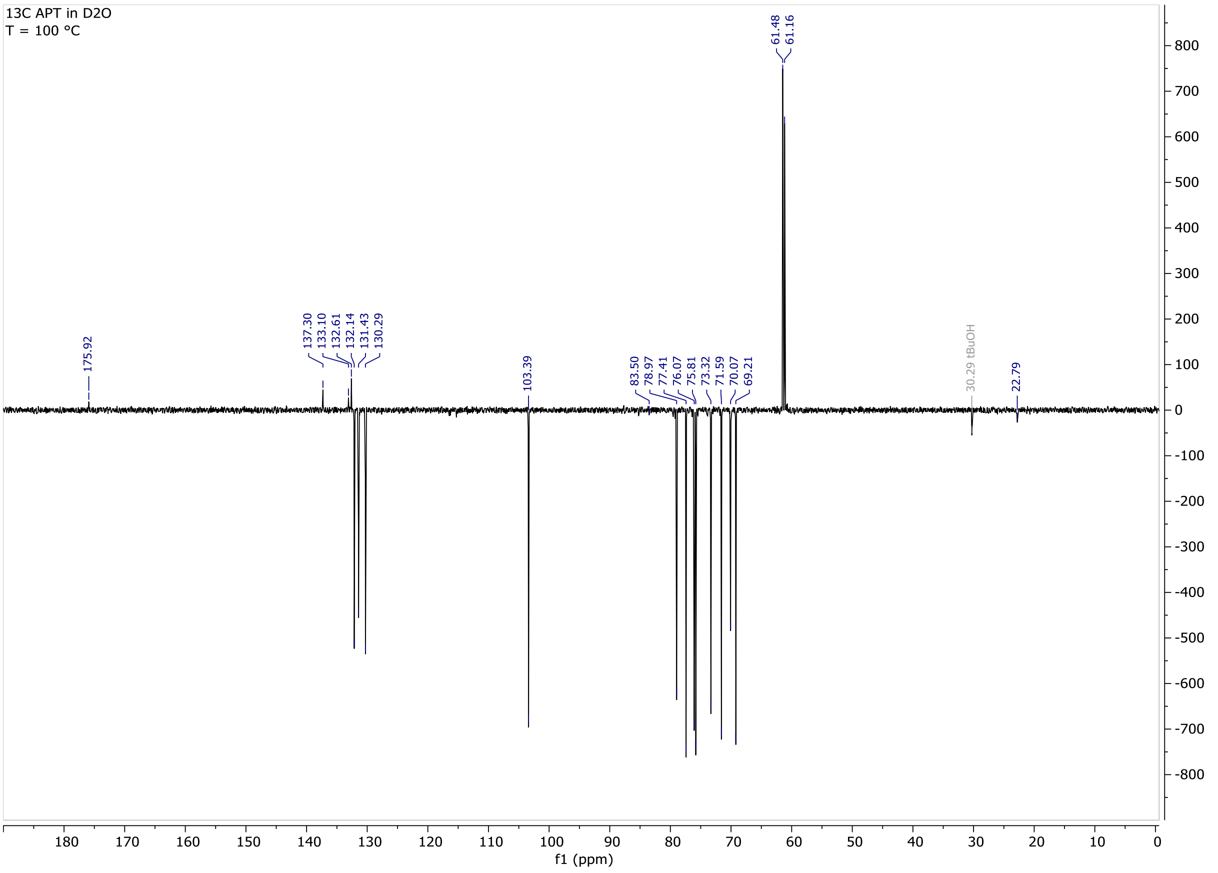

^{1}H and ^{13}C APT NMR spectra of (3l)

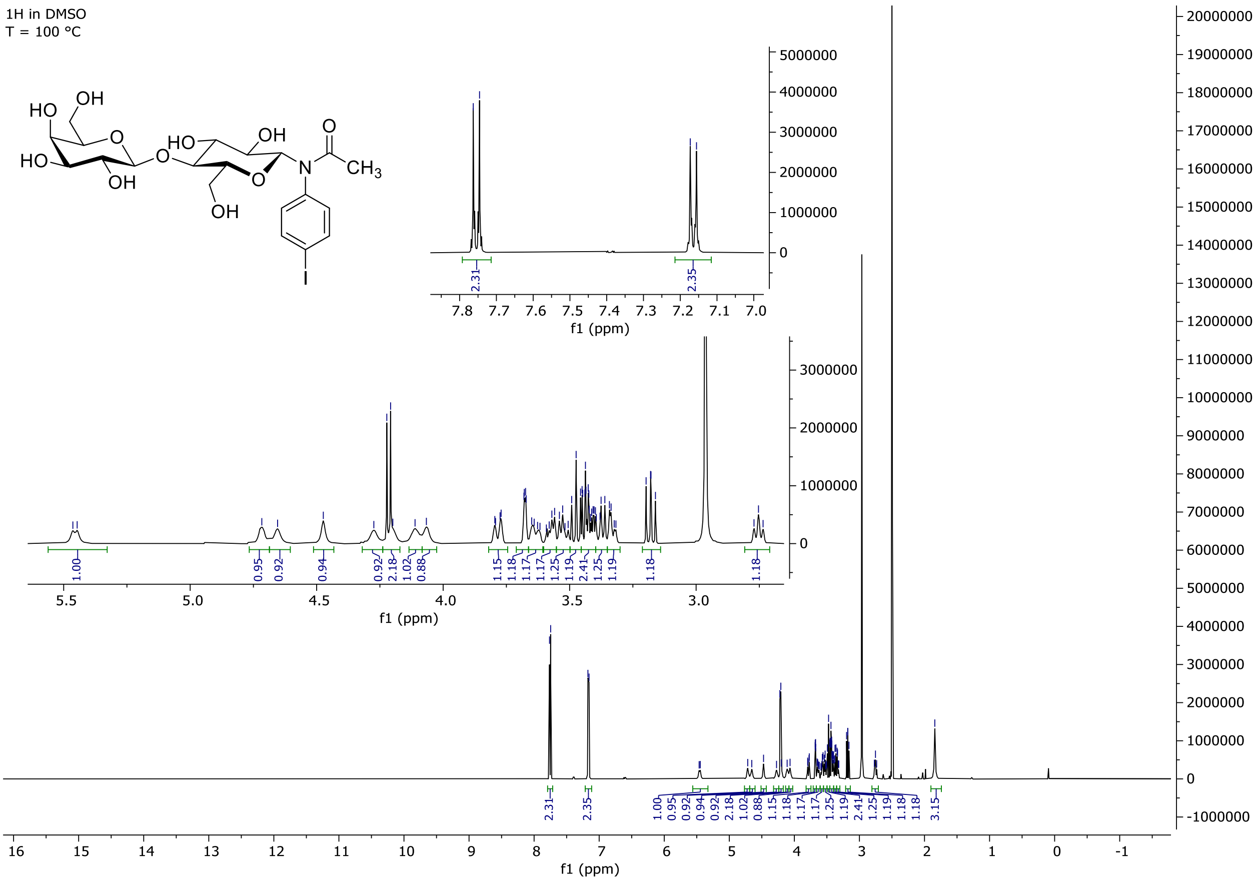

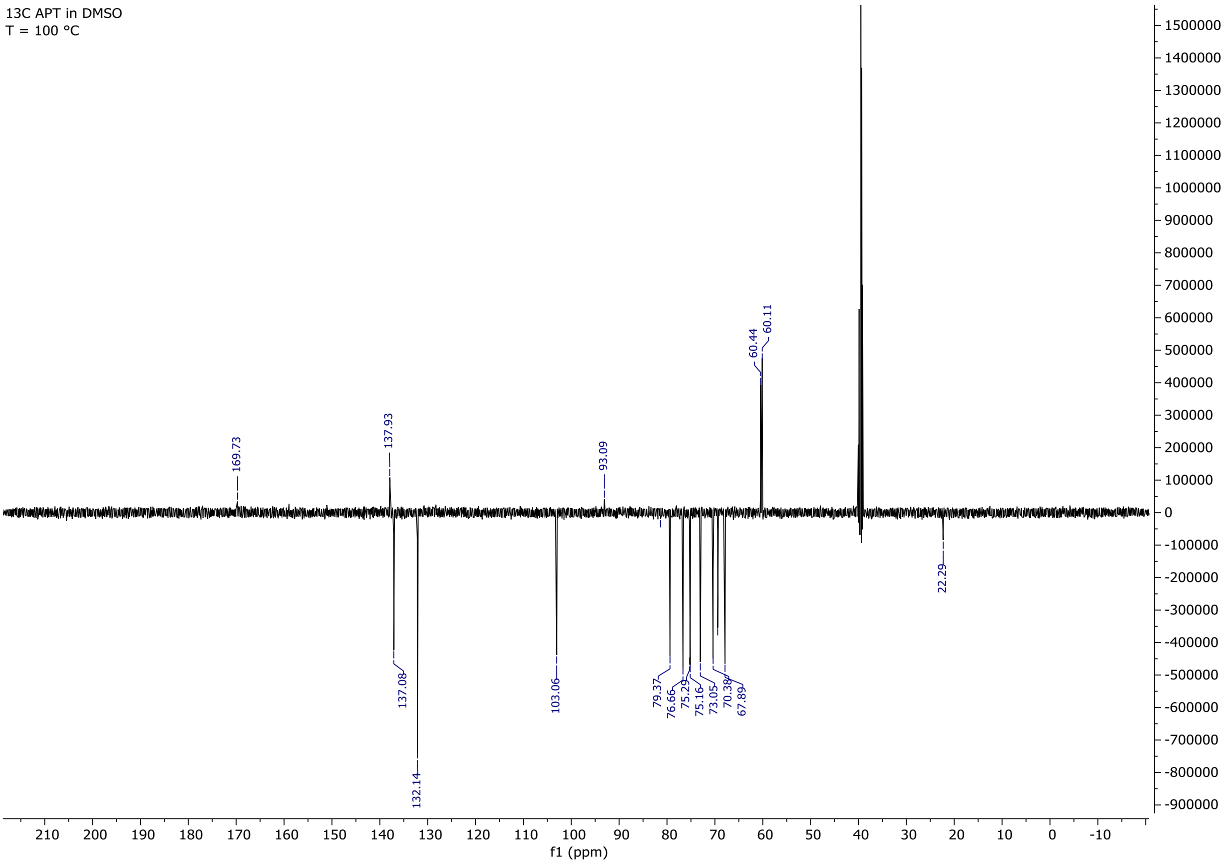

^{1}H, ^{13}C APT and HSQC NMR spectra of (3m)

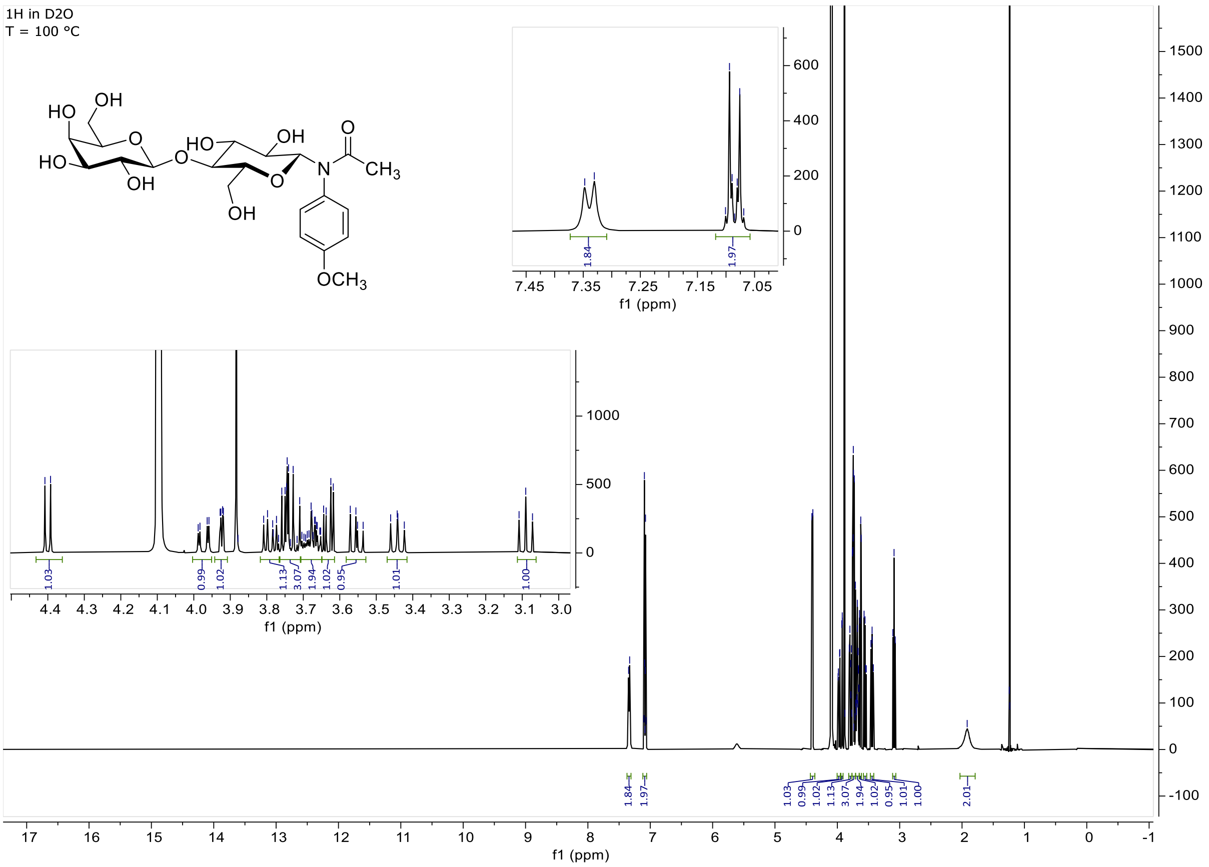

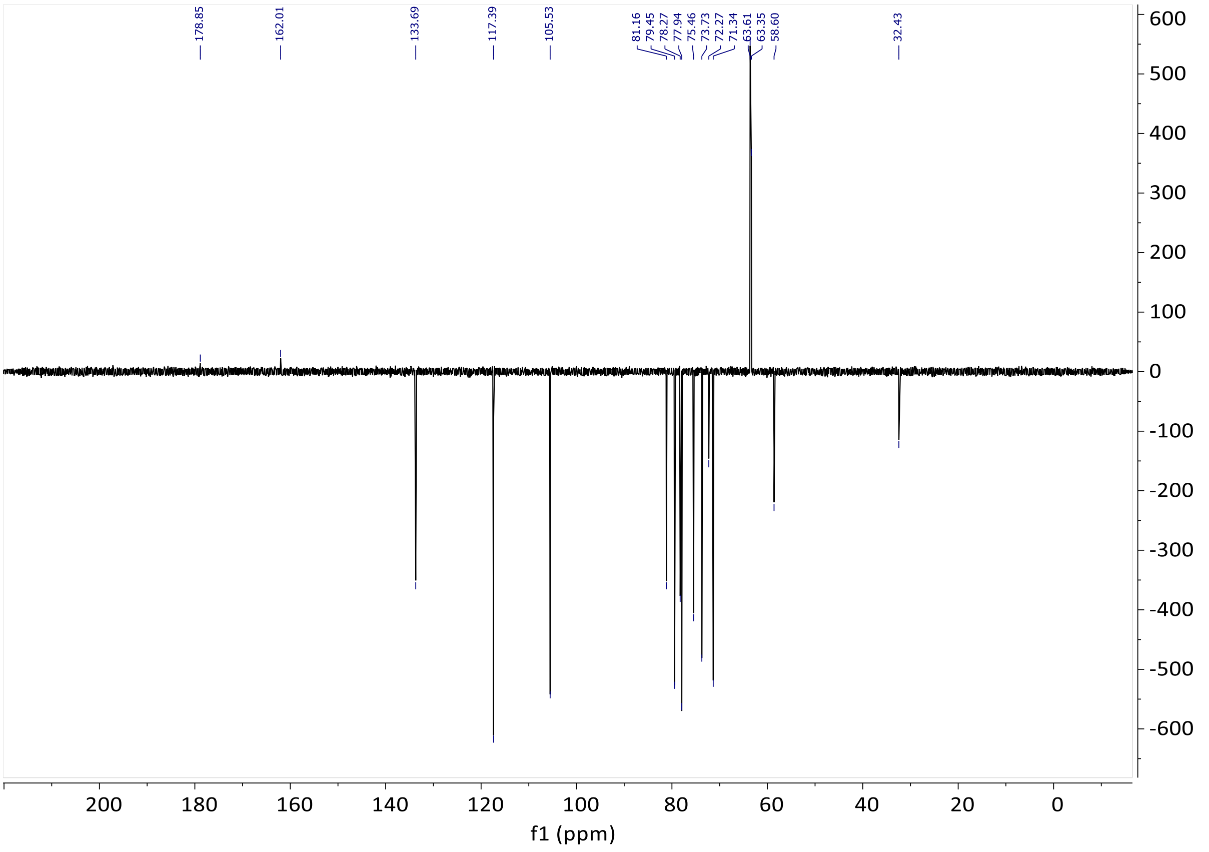

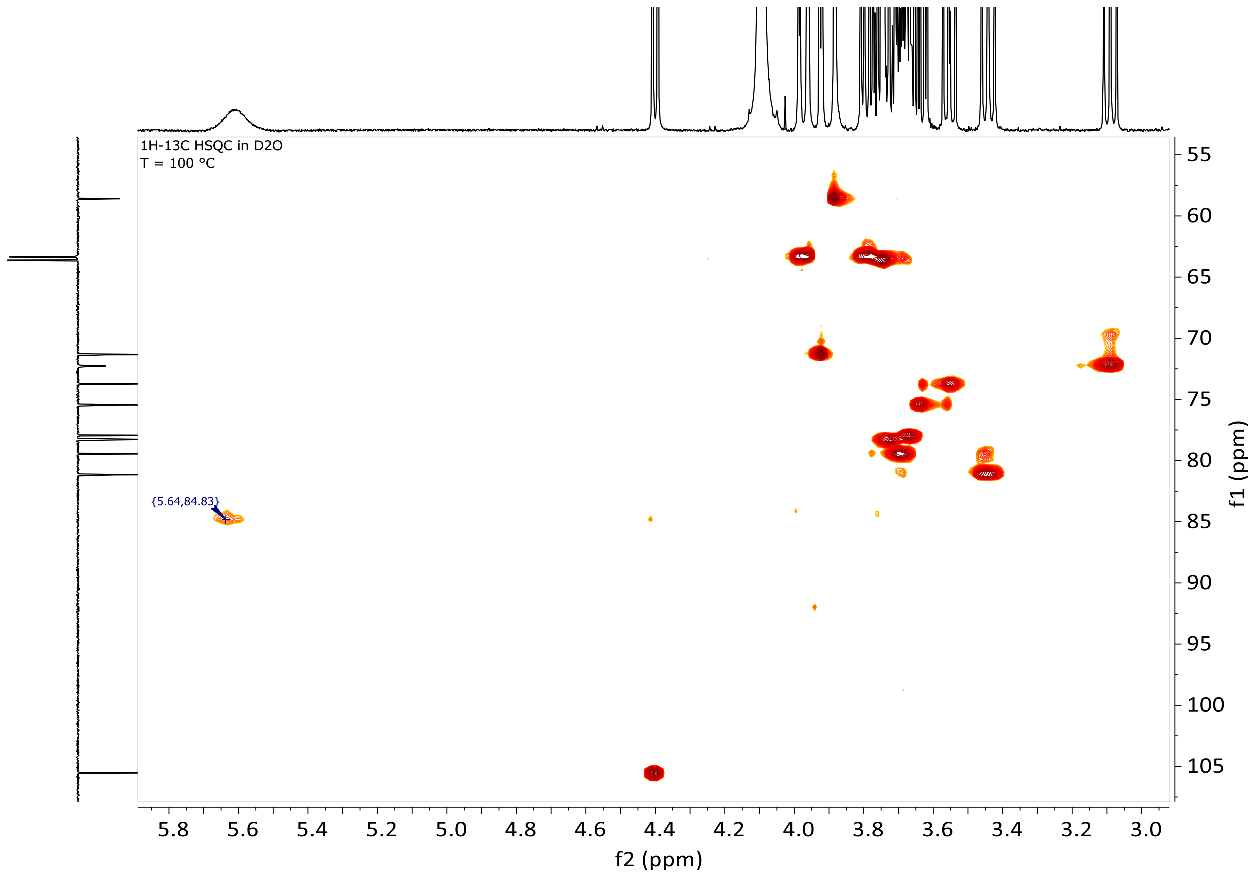
1H-13C HSQC in D2O
T = 100 °C
{5.64,84.83}
f2 (ppm)
f1 (ppm)

^{1}H and ^{13}C APT NMR spectra of (3n)

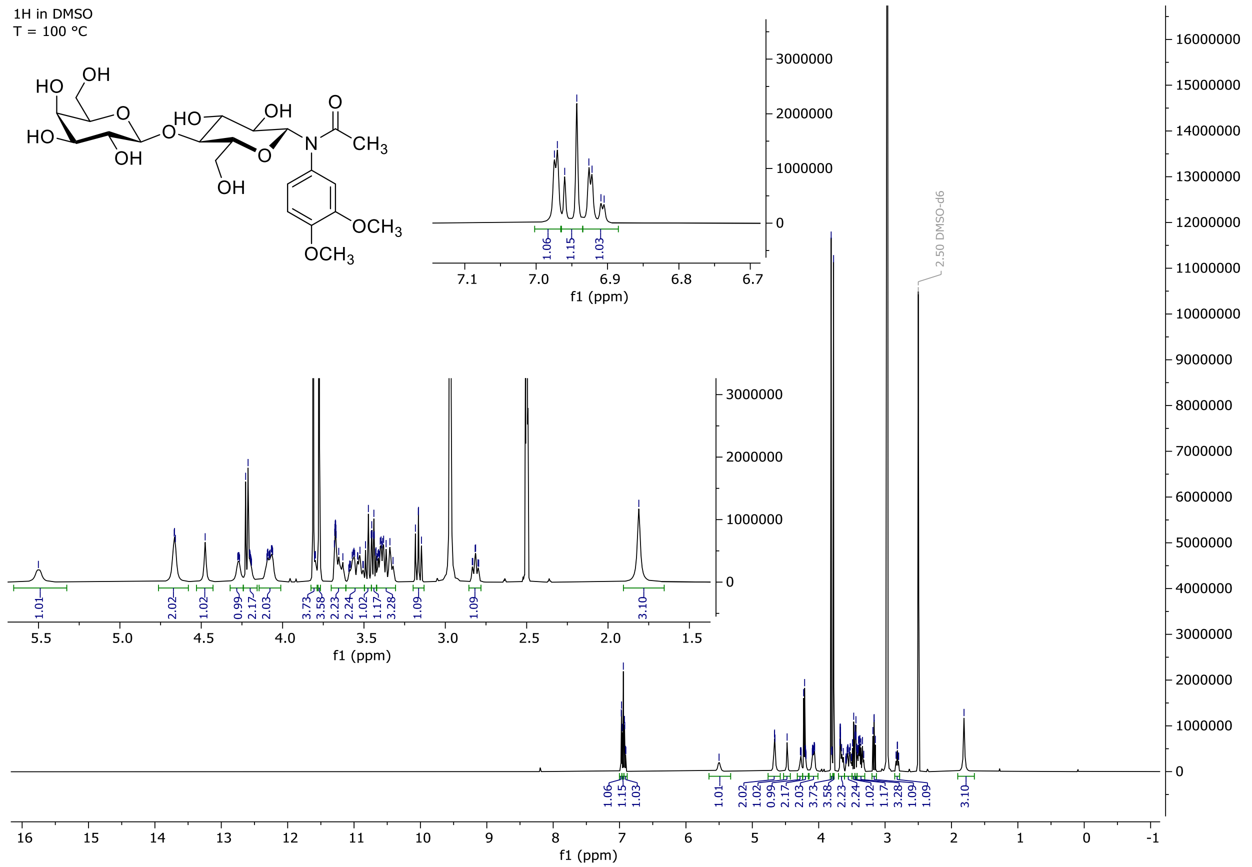

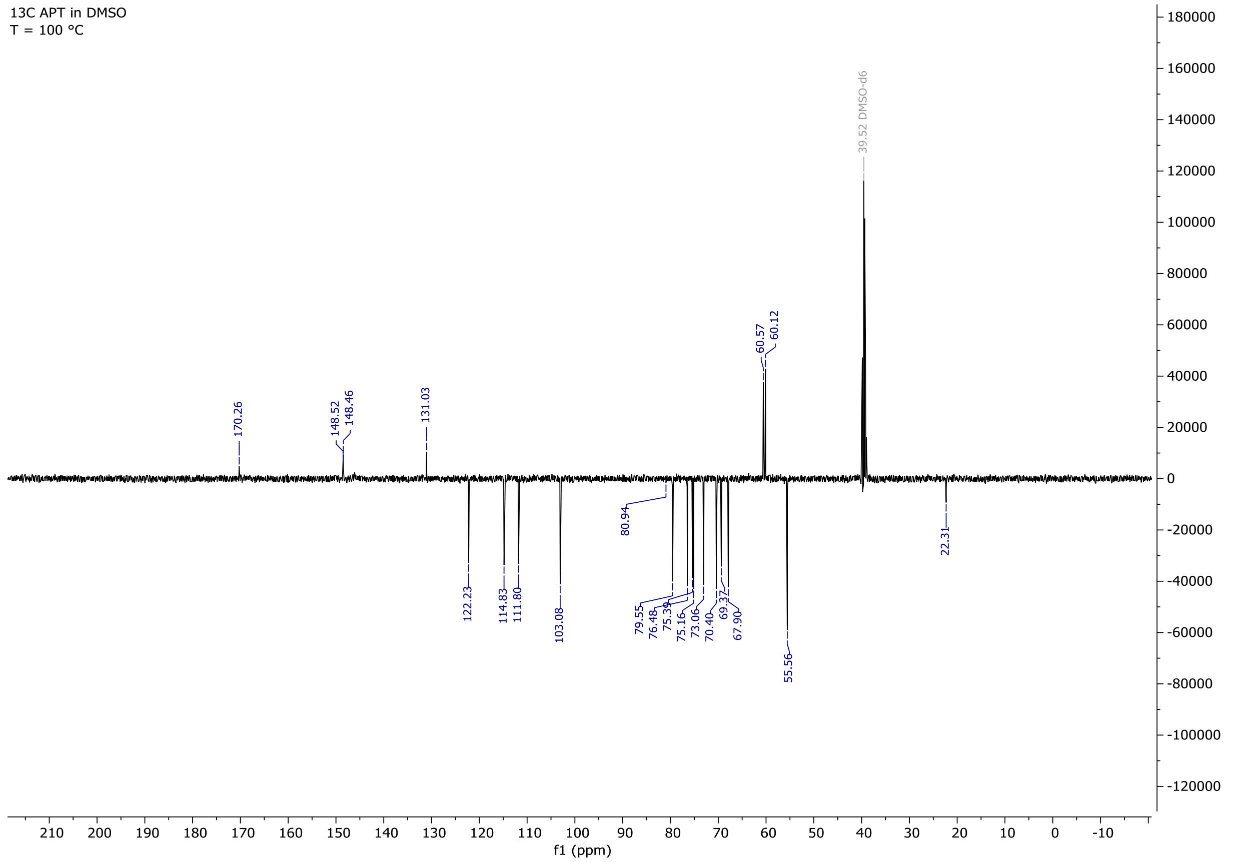

^{1}H and ^{13}C APT NMR spectra of (3o)

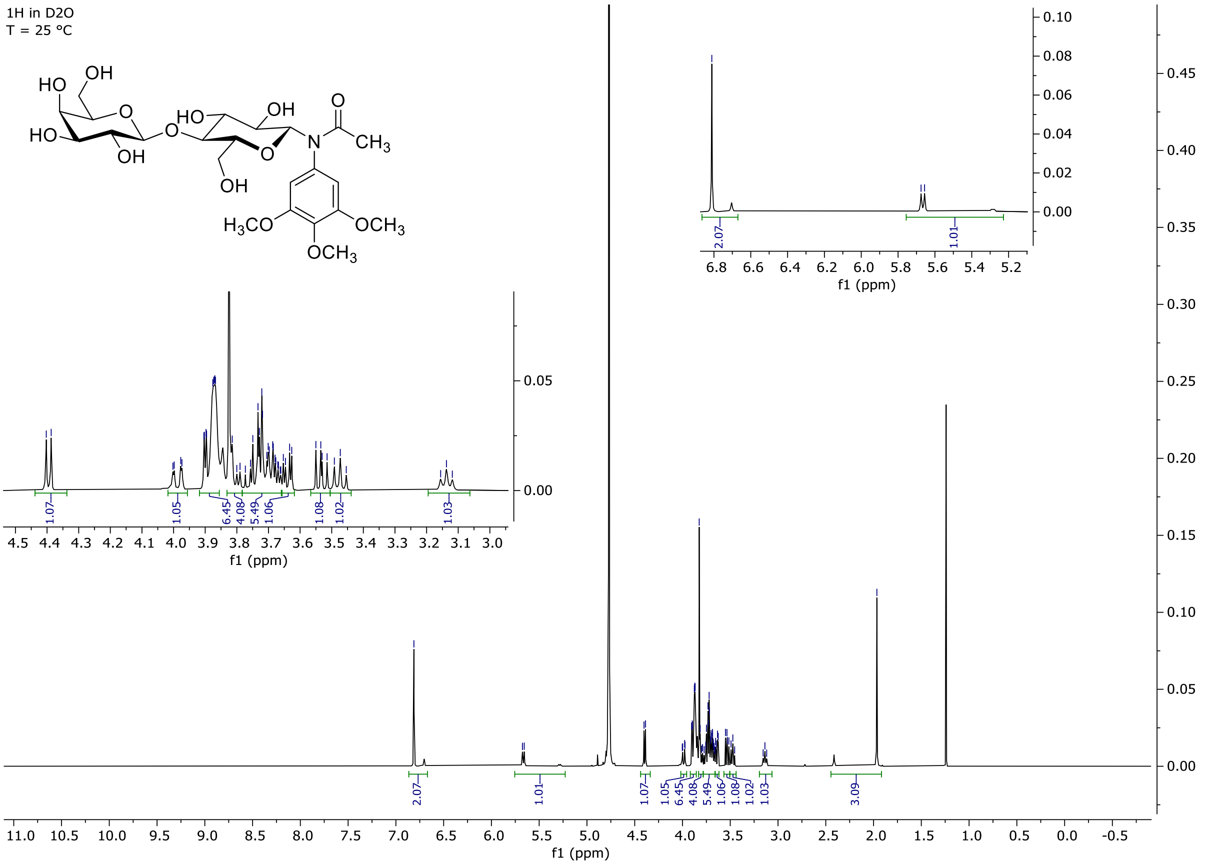

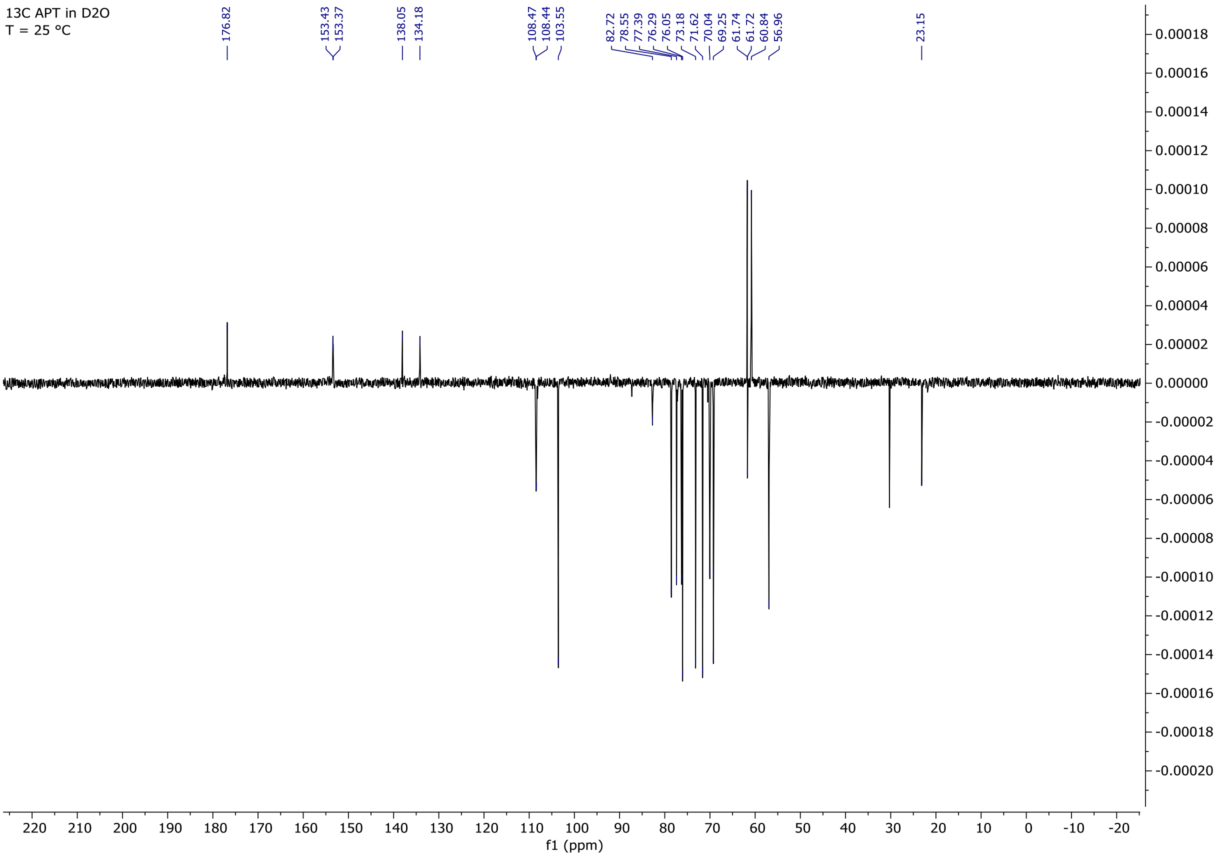

^{1}H and ^{13}C APT NMR spectra of (3p)

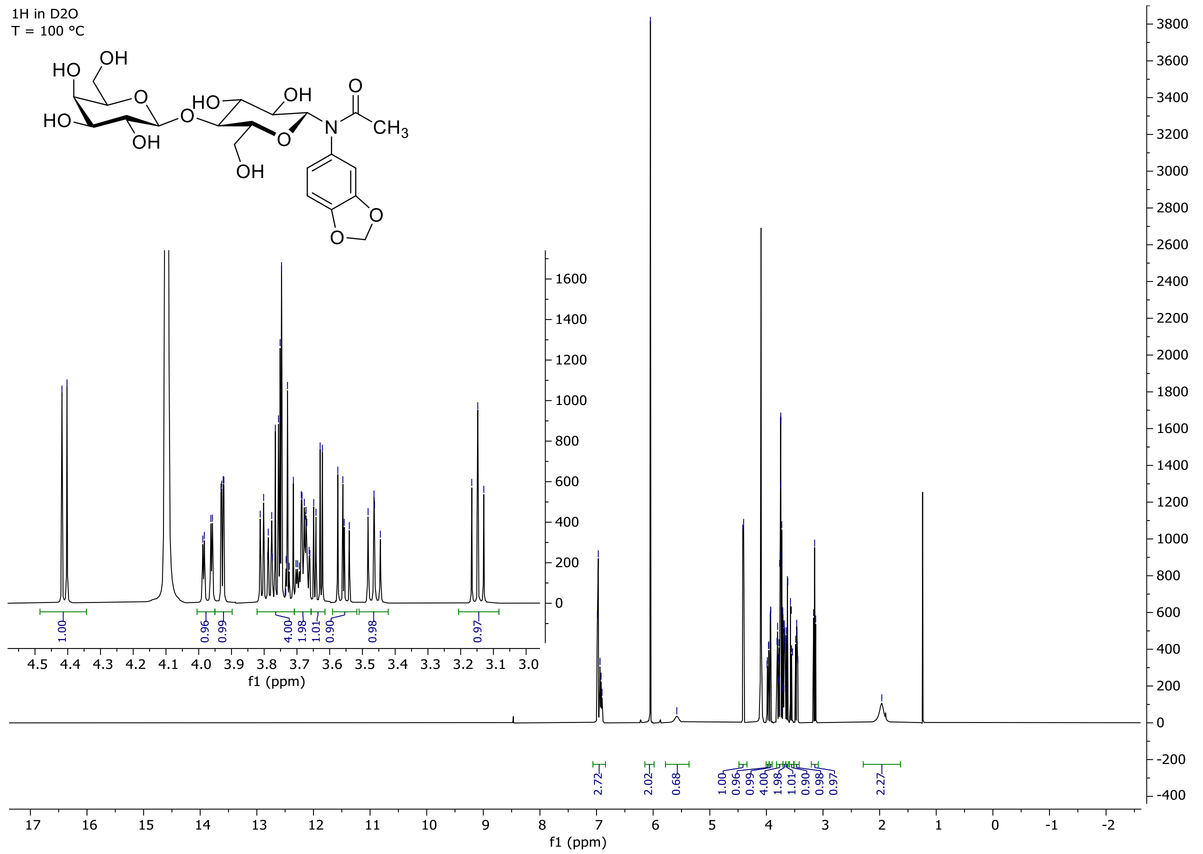

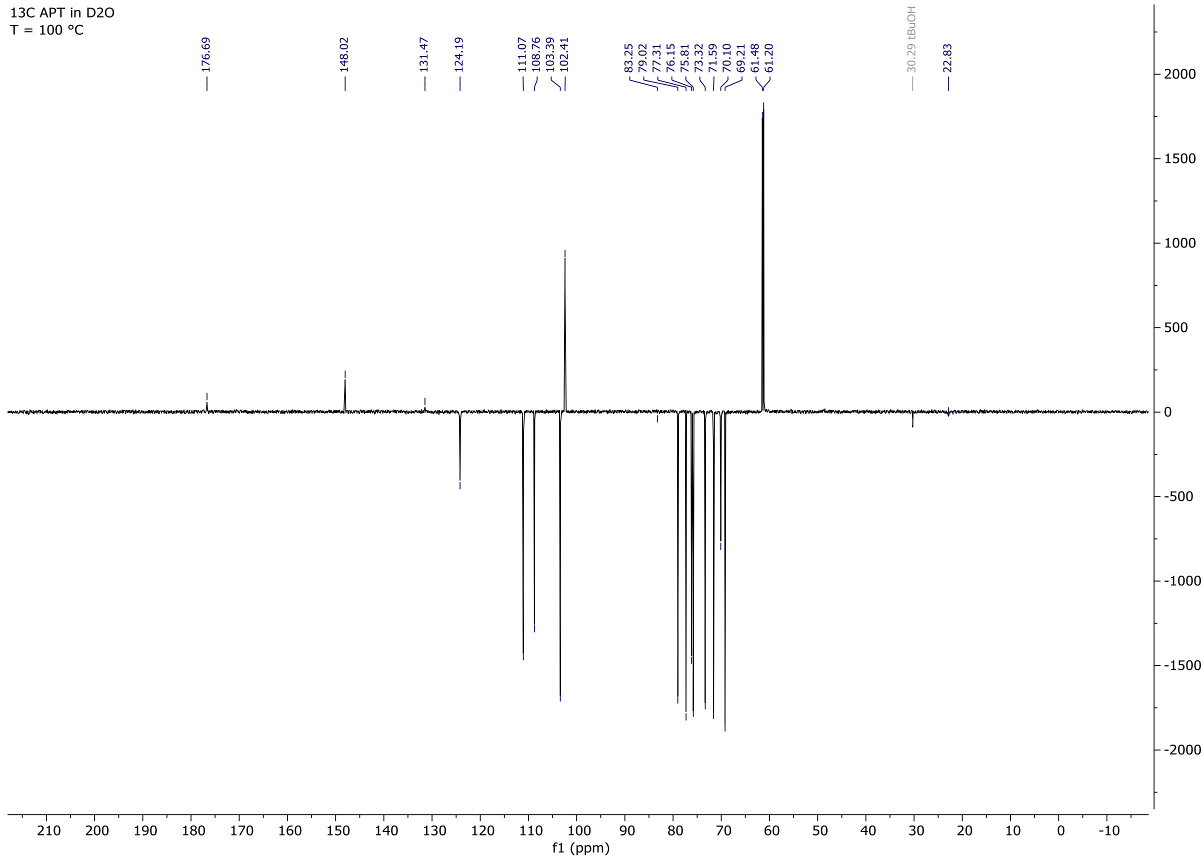

^{1}H and ^{13}C APT NMR spectra of (3q)

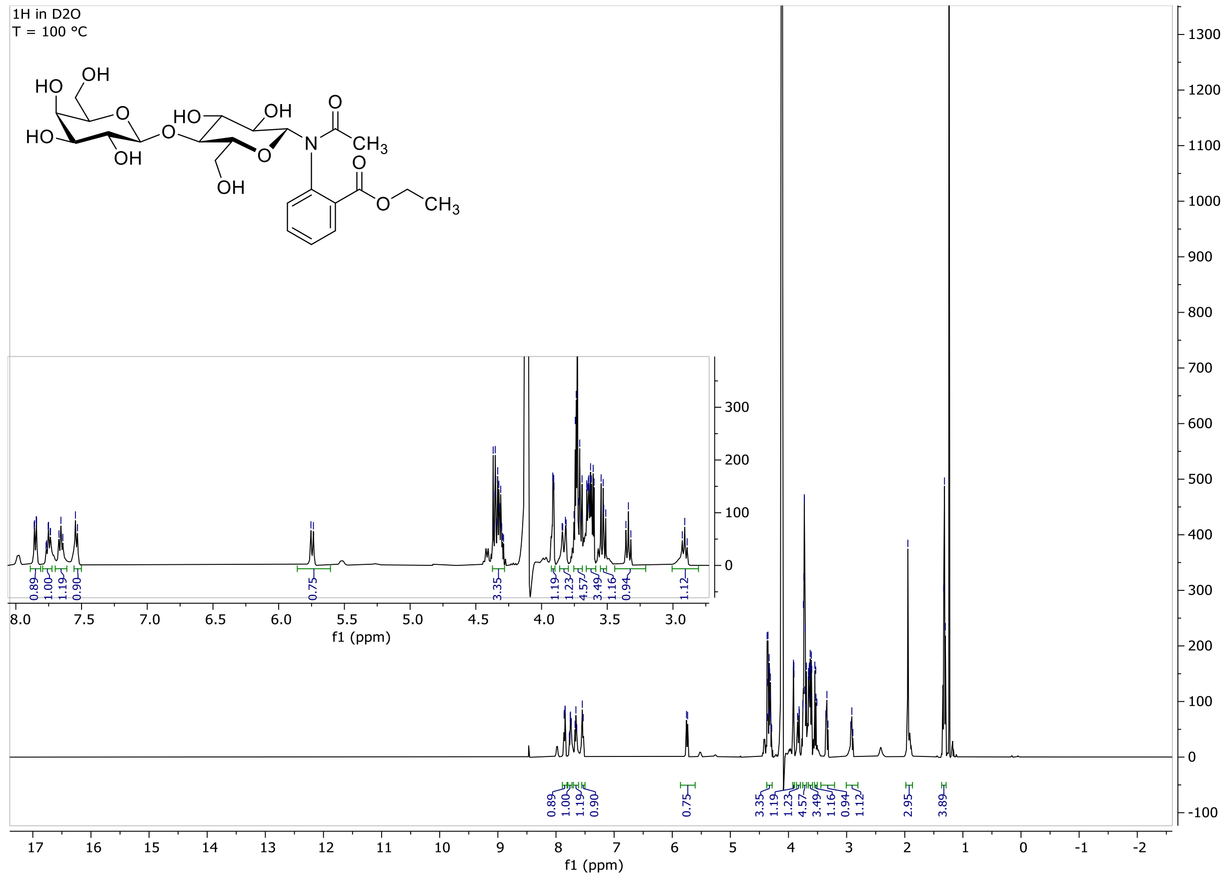

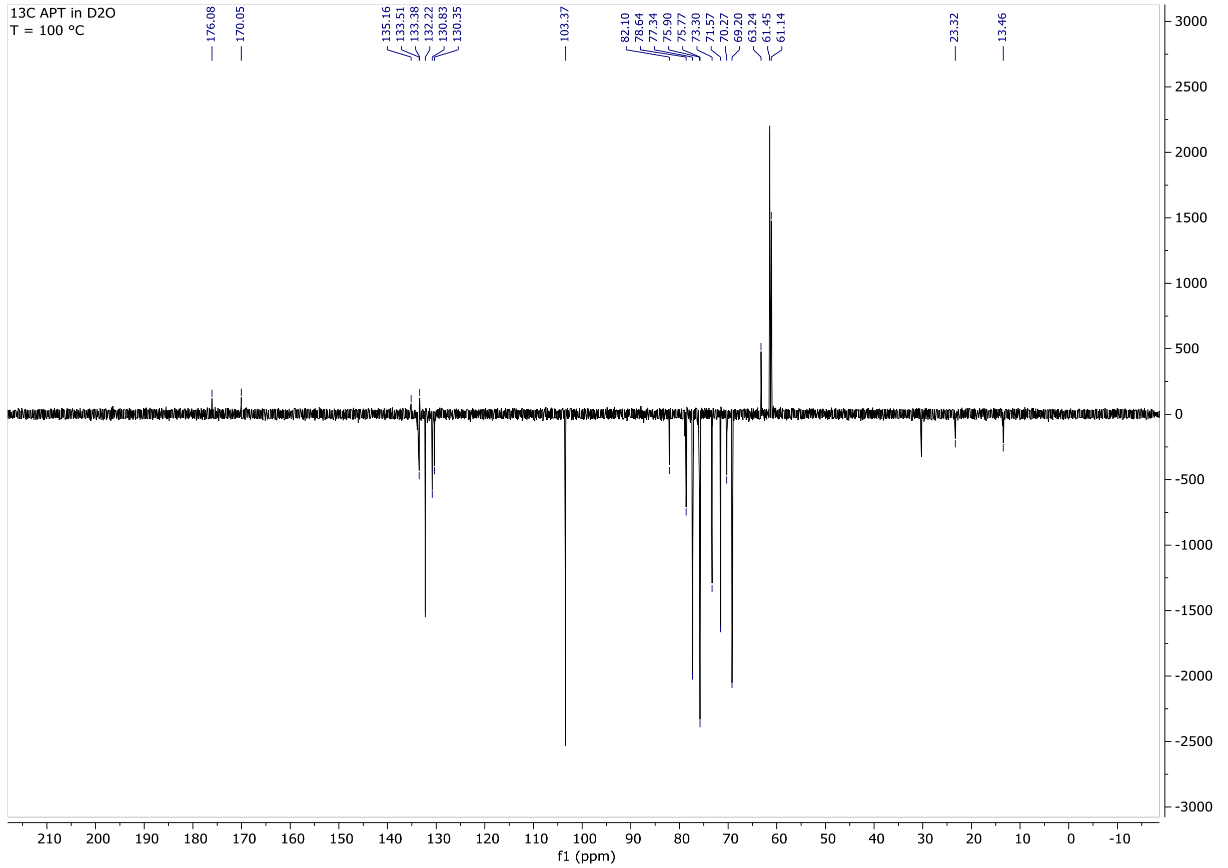

^{1}H and ^{13}C APT NMR spectra of (3r)

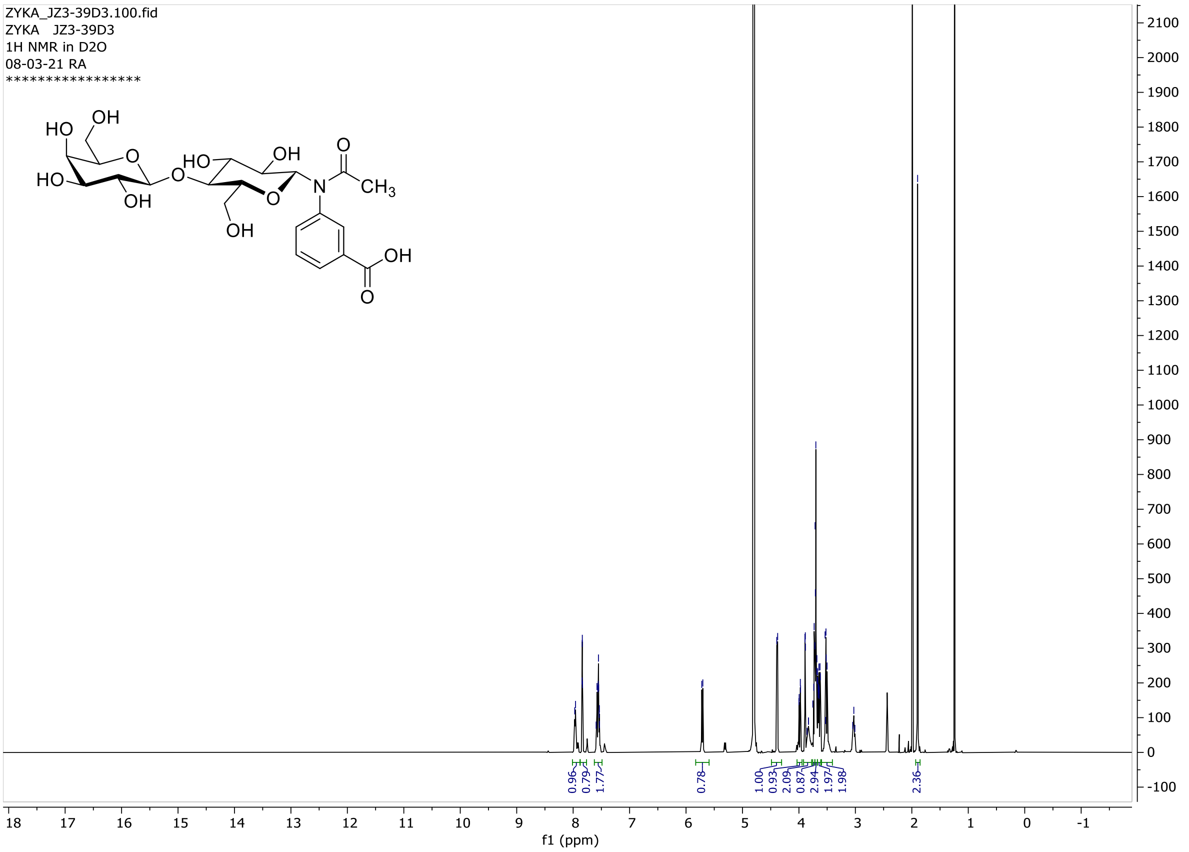

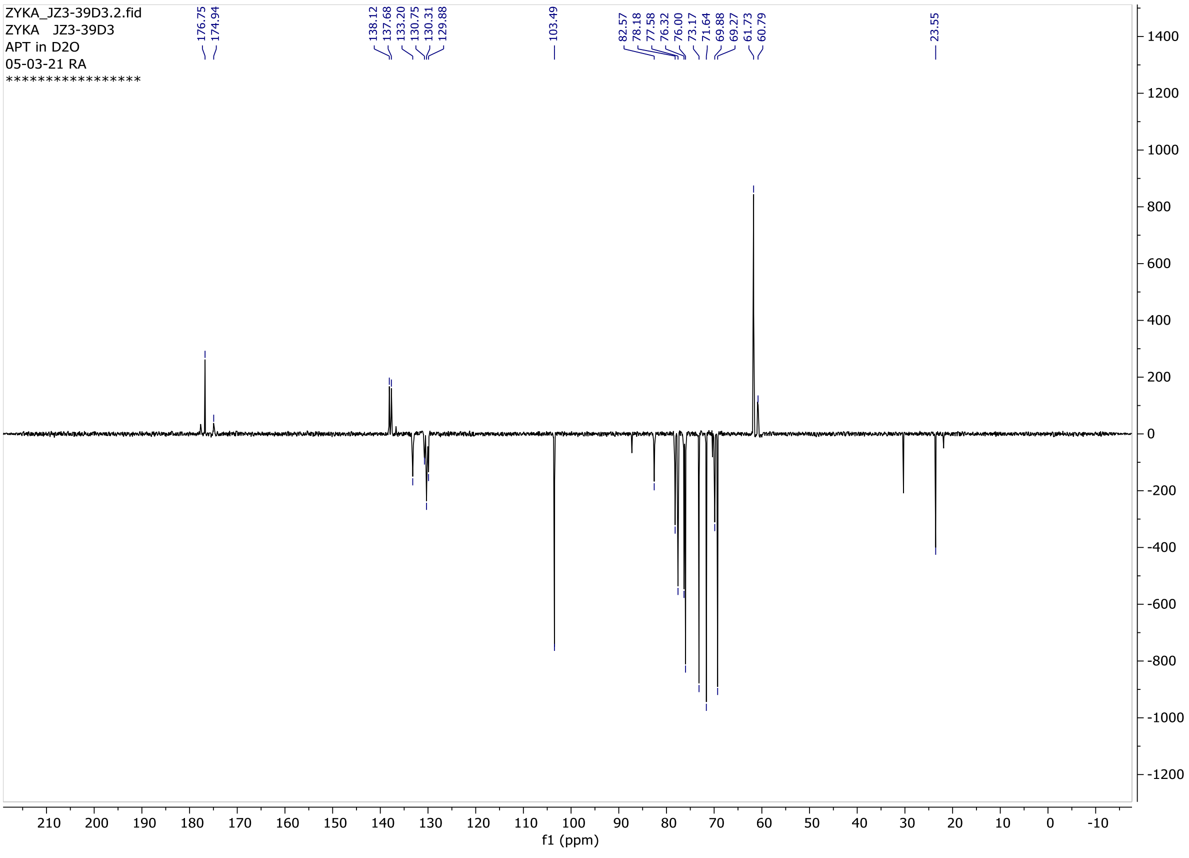

^{1}H and ^{13}C APT NMR spectra of (3s)

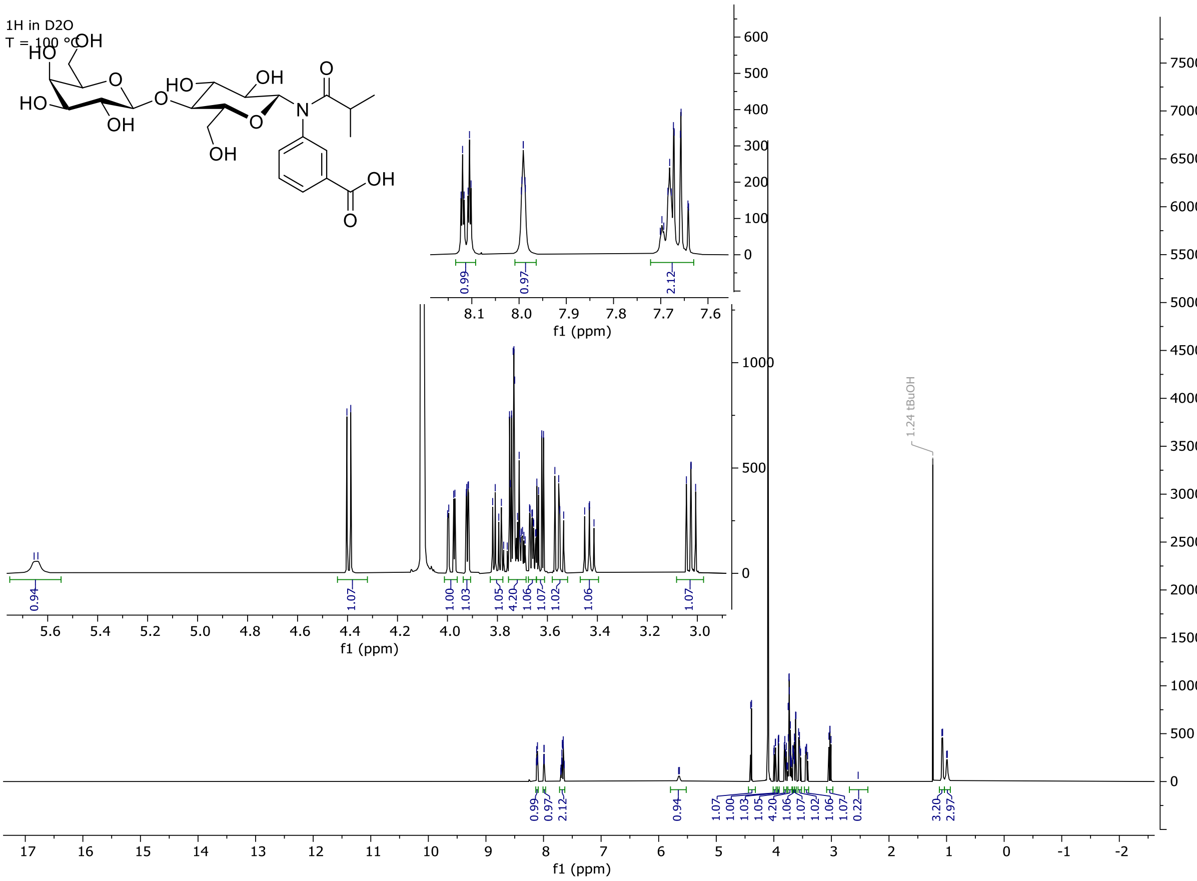

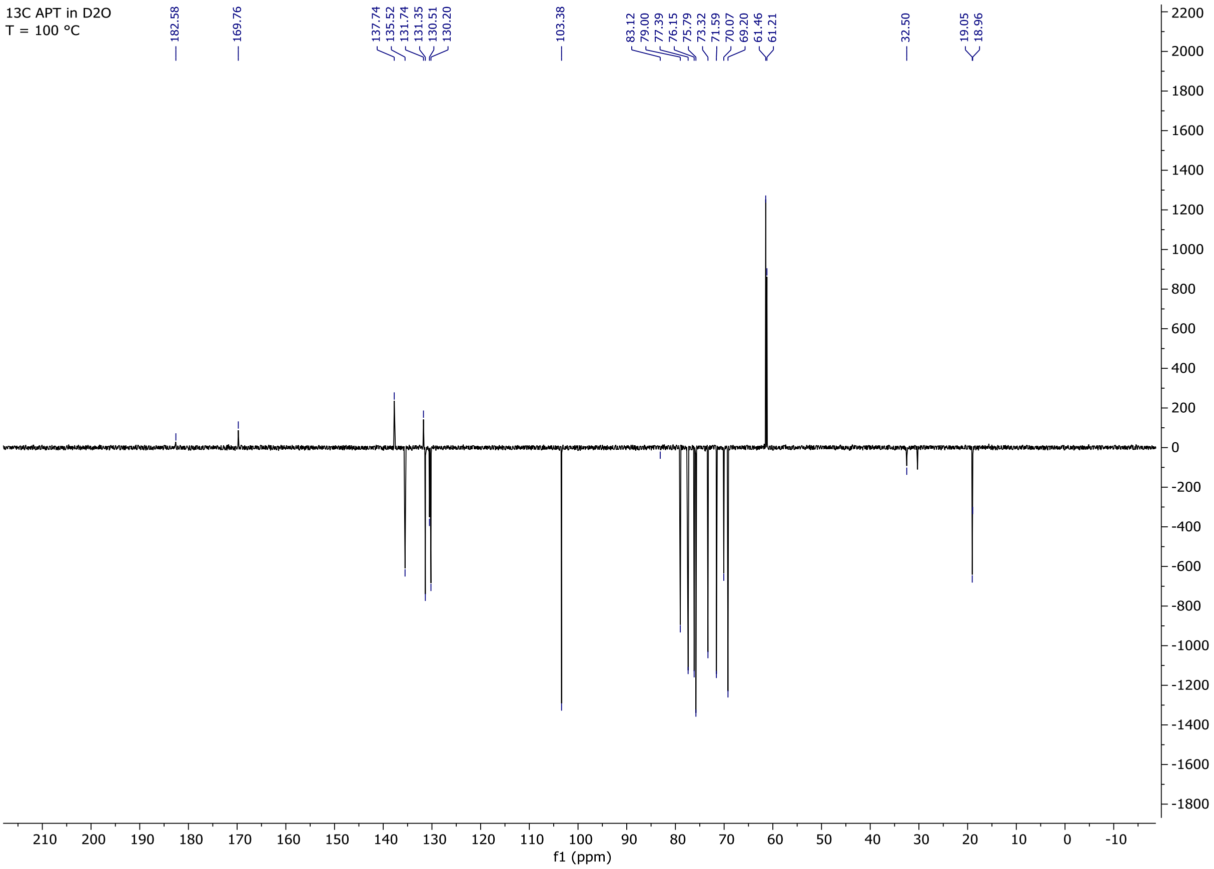

¹H and ¹³C APT NMR spectra of (3t)

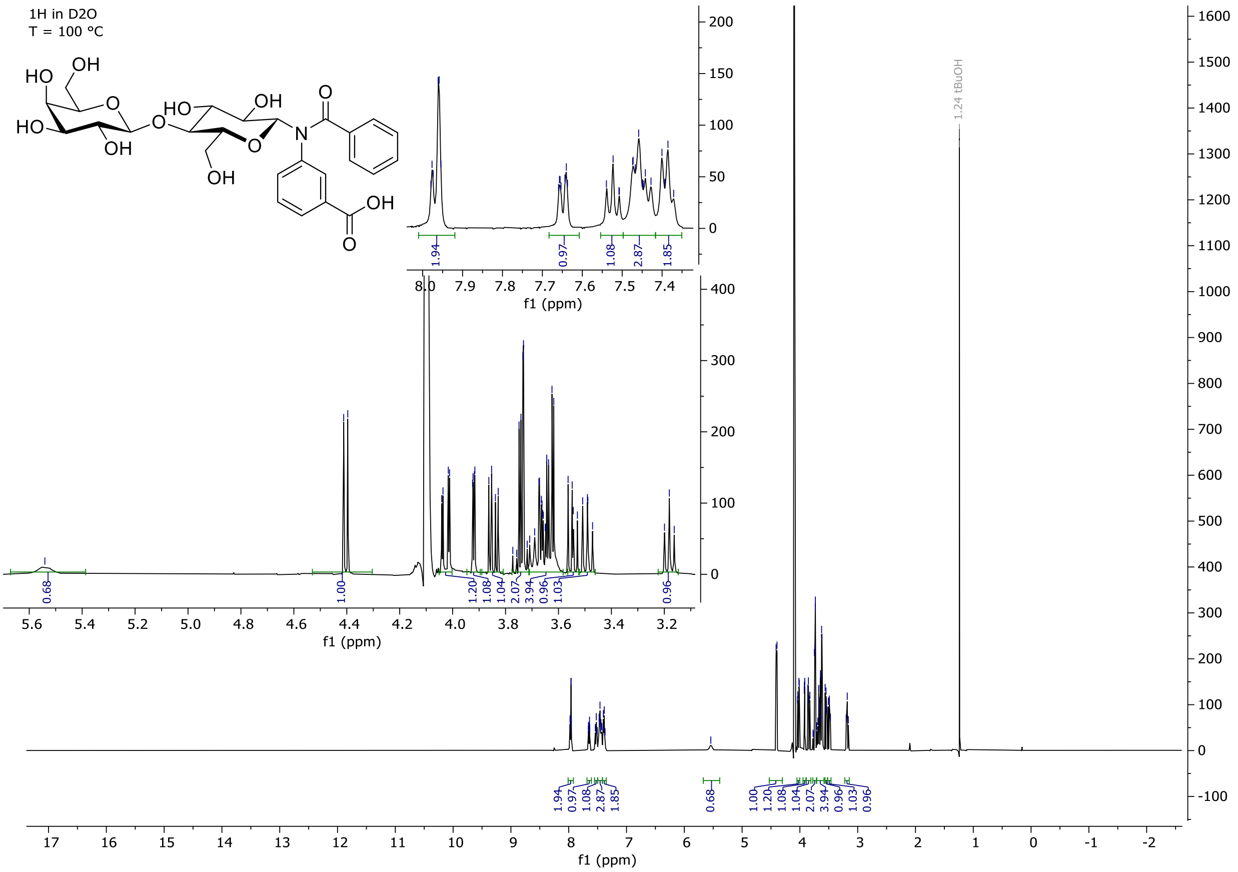

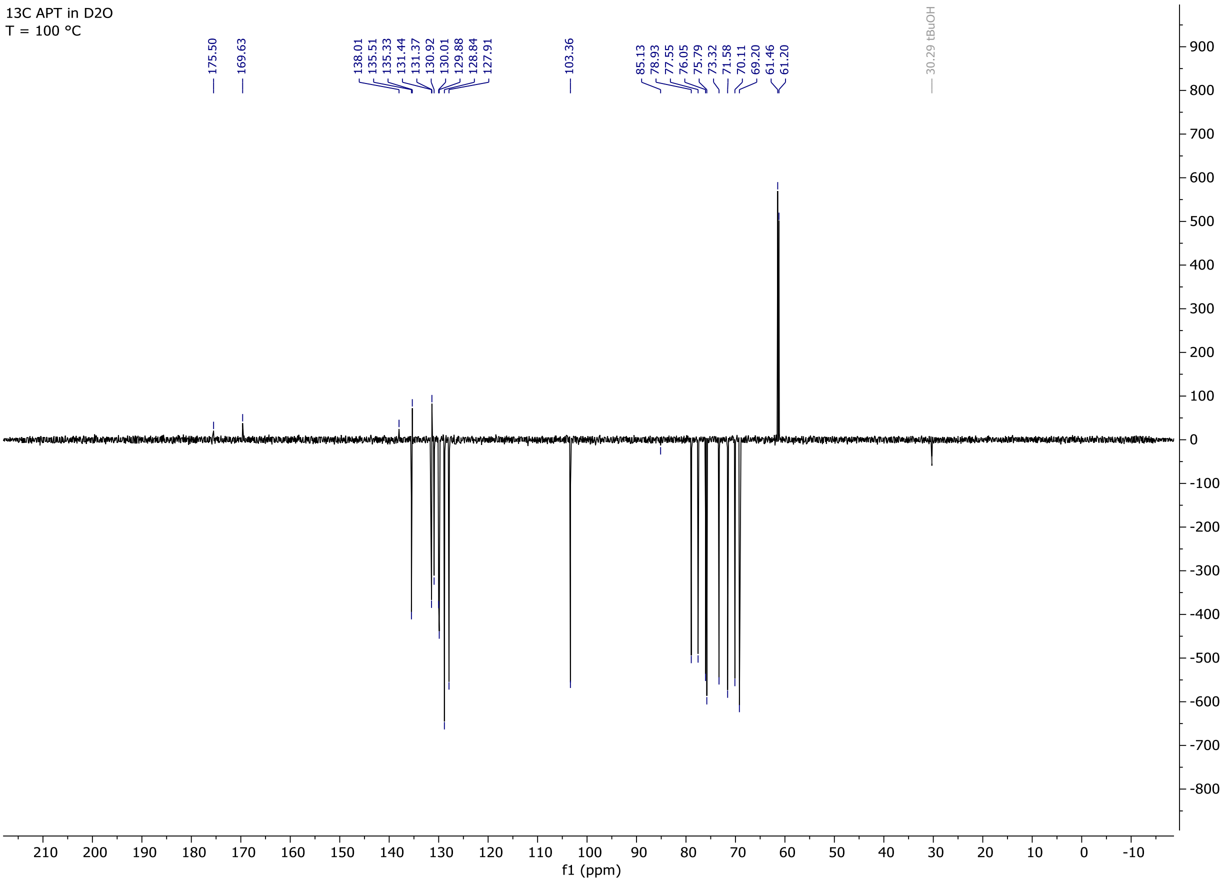

^{1}H and ^{13}C APT NMR spectra of (3u)

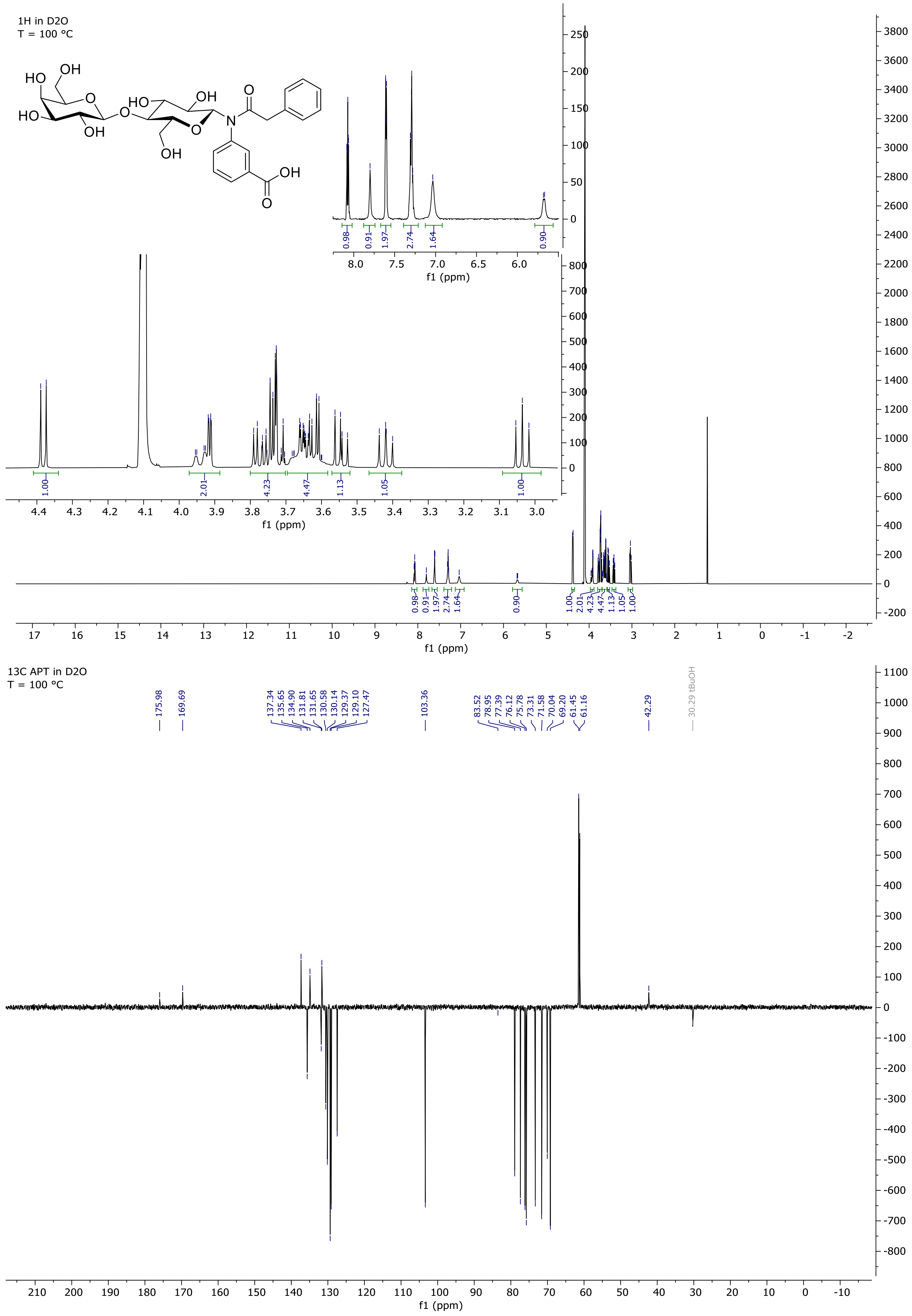

^{1}H and ^{13}C APT NMR spectra of (3v)

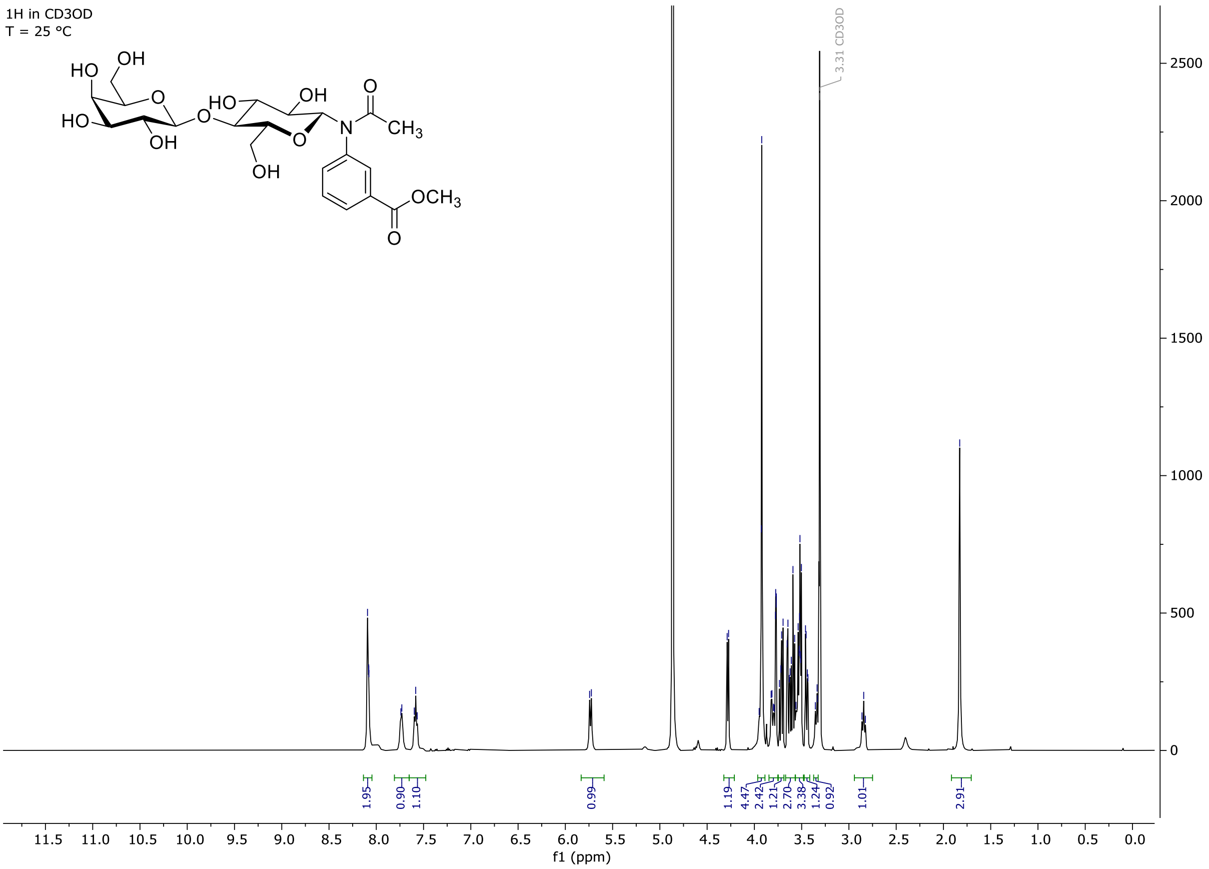

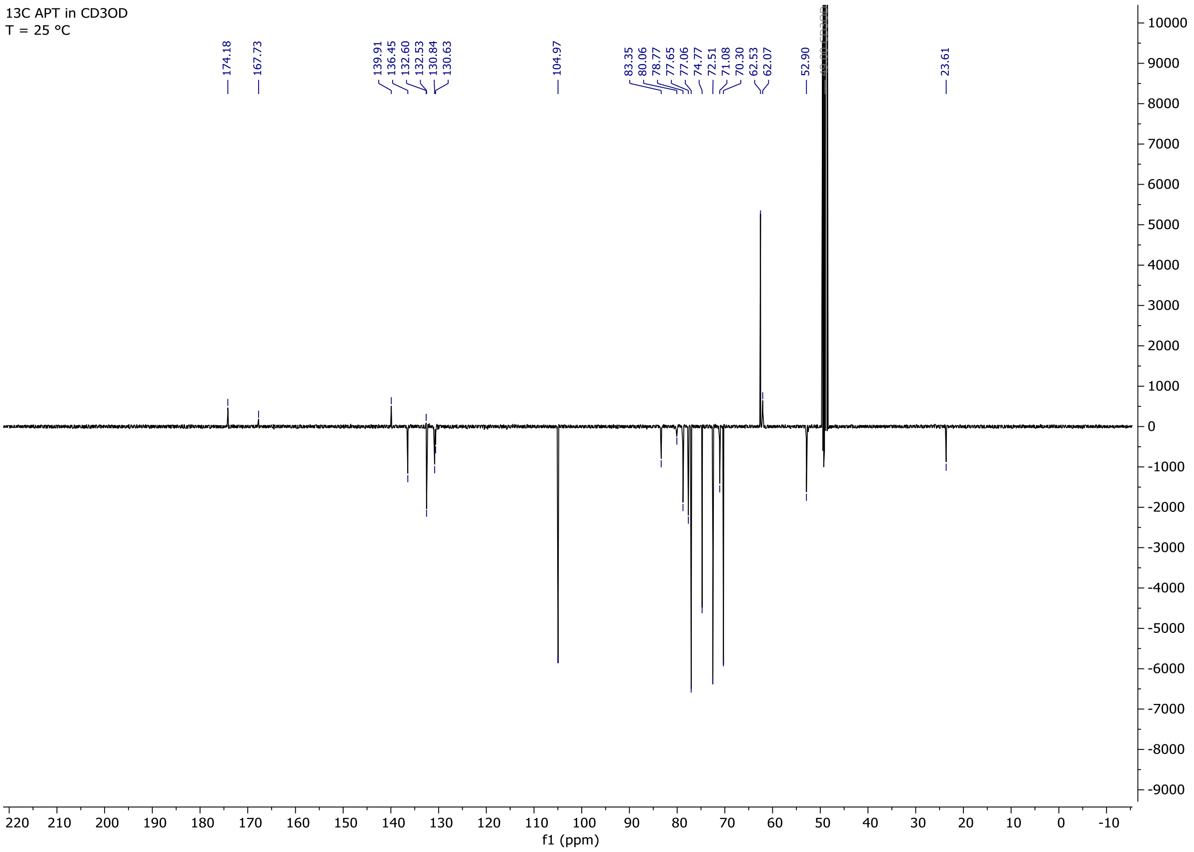

^{1}H and ^{13}C APT NMR spectra of (3w)

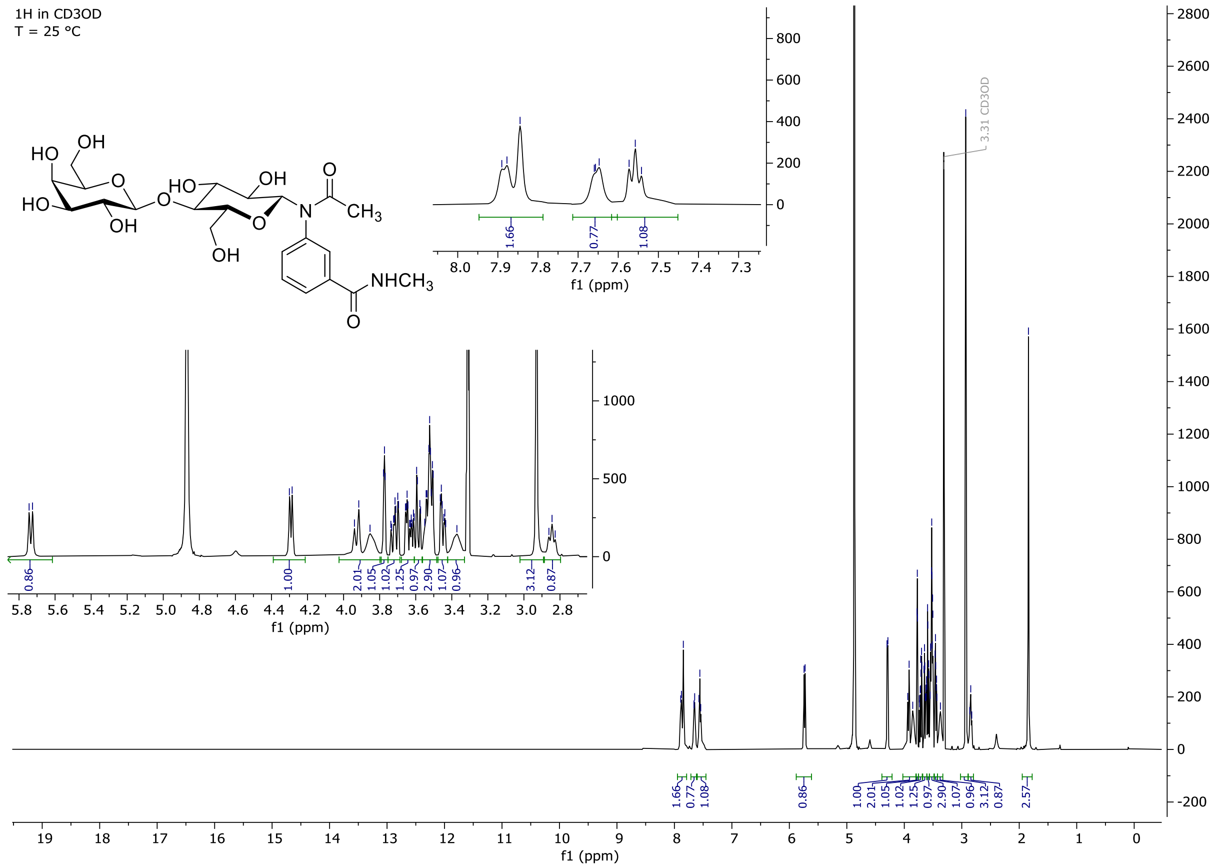

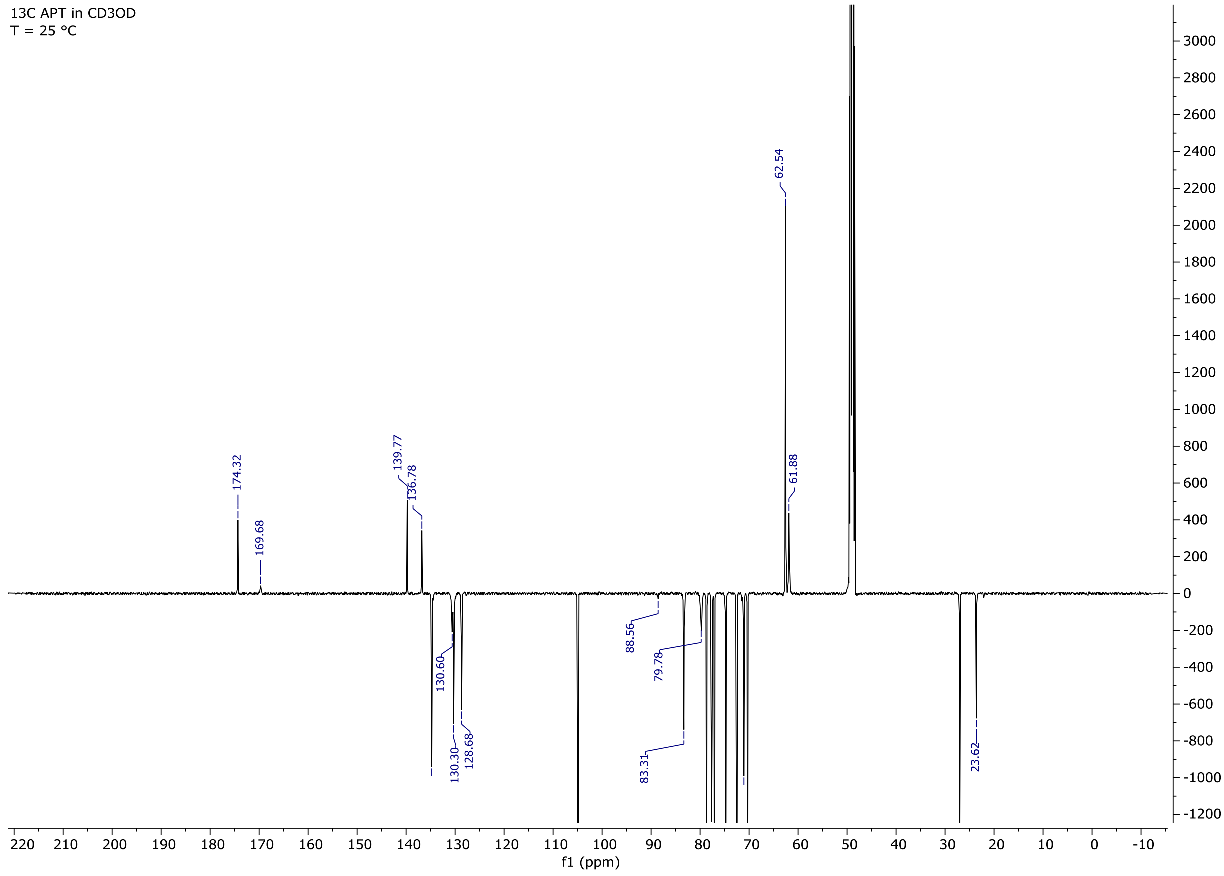

^{1}H and ^{13}C APT NMR spectra of (3x)

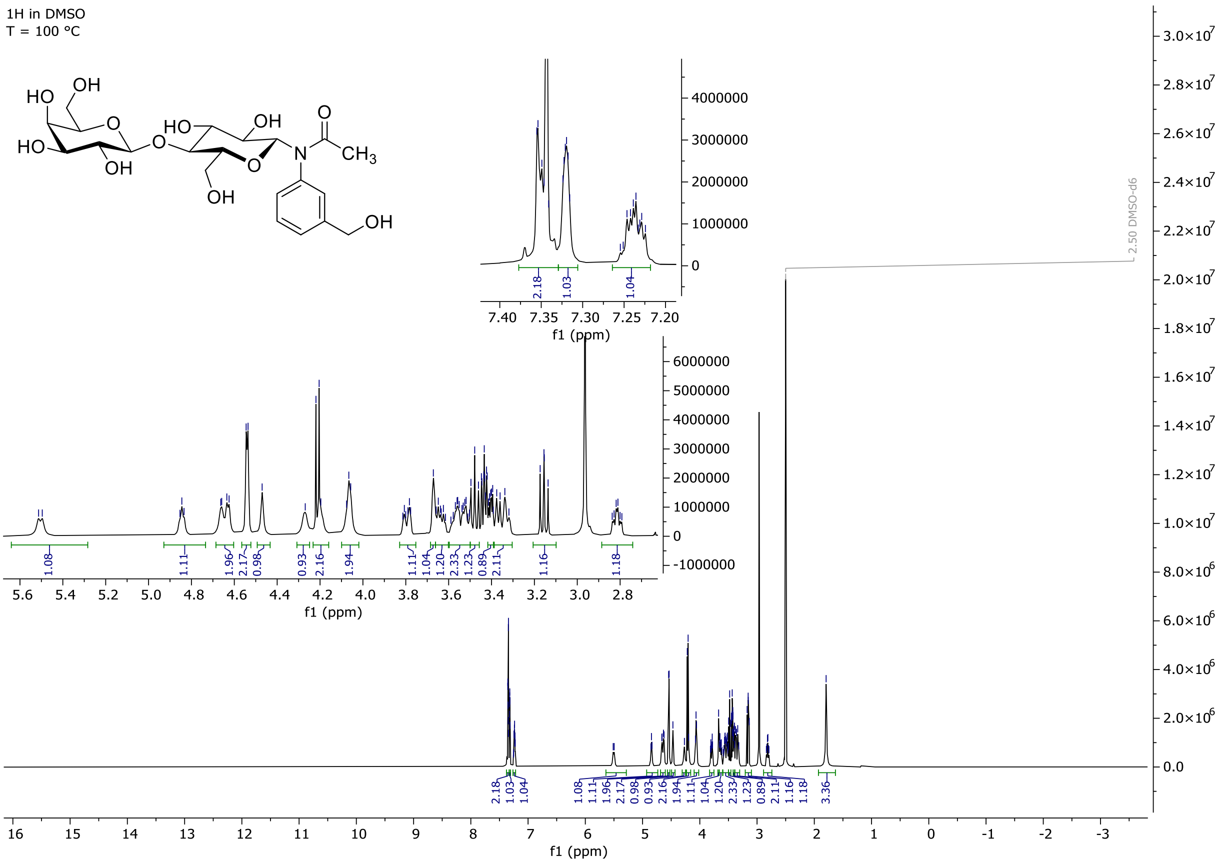

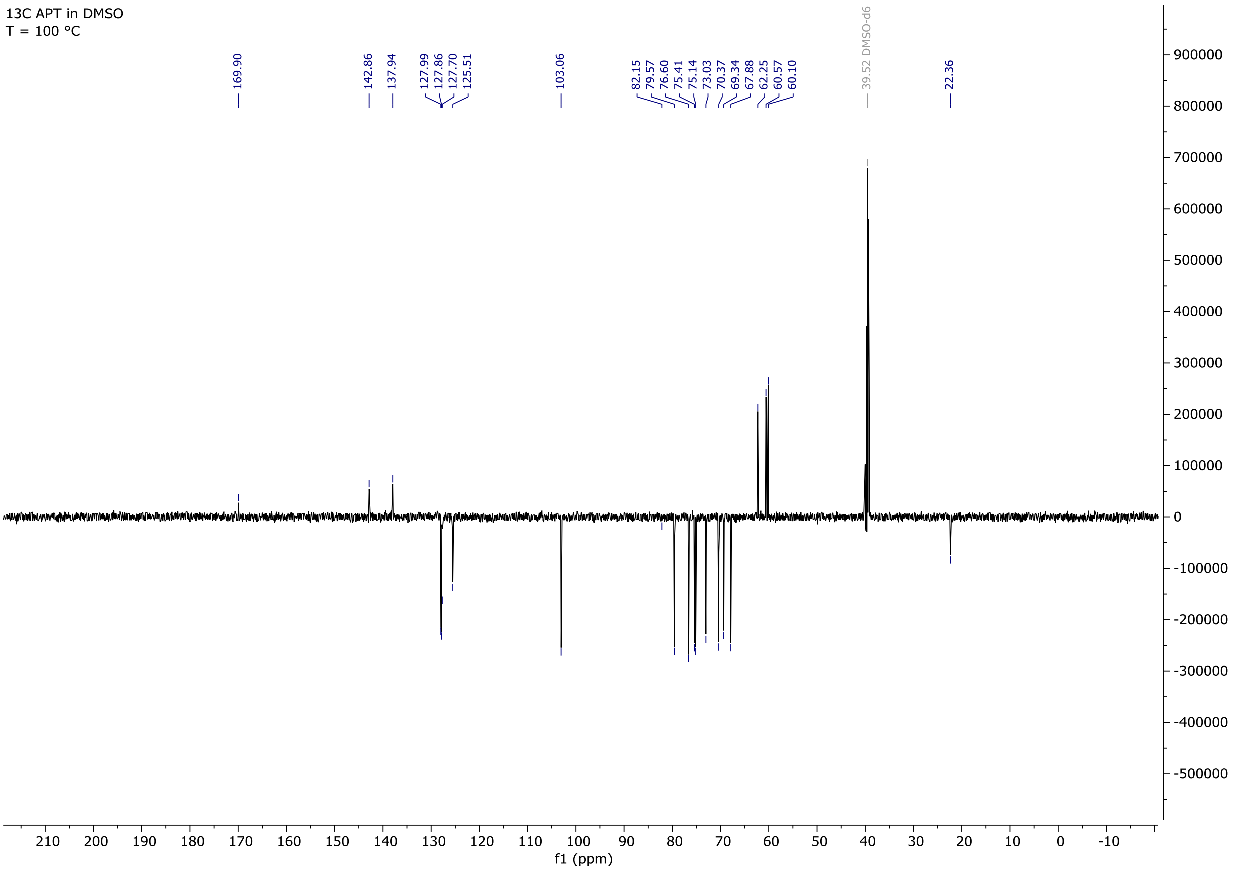

^{1}H, ^{13}C APT, HSQC and HMBC NMR spectra of (3y)

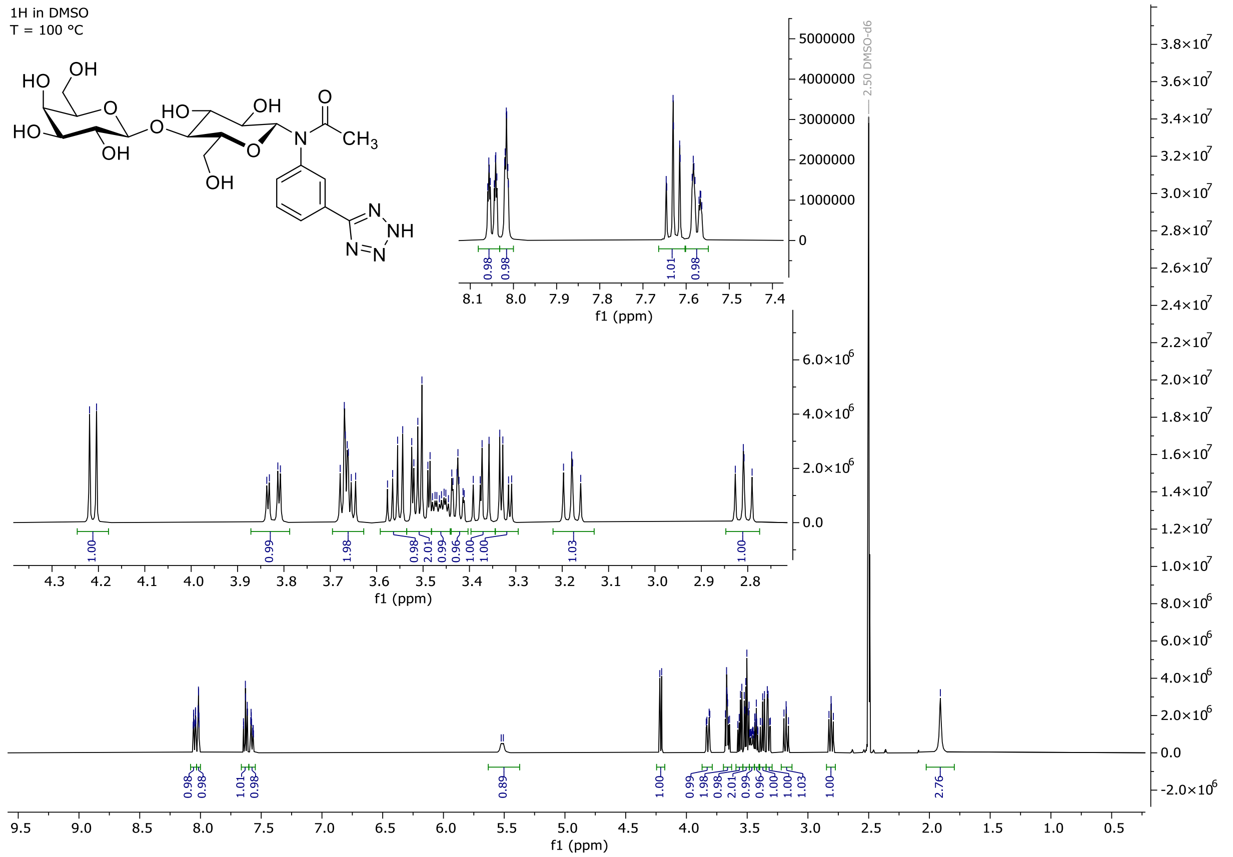

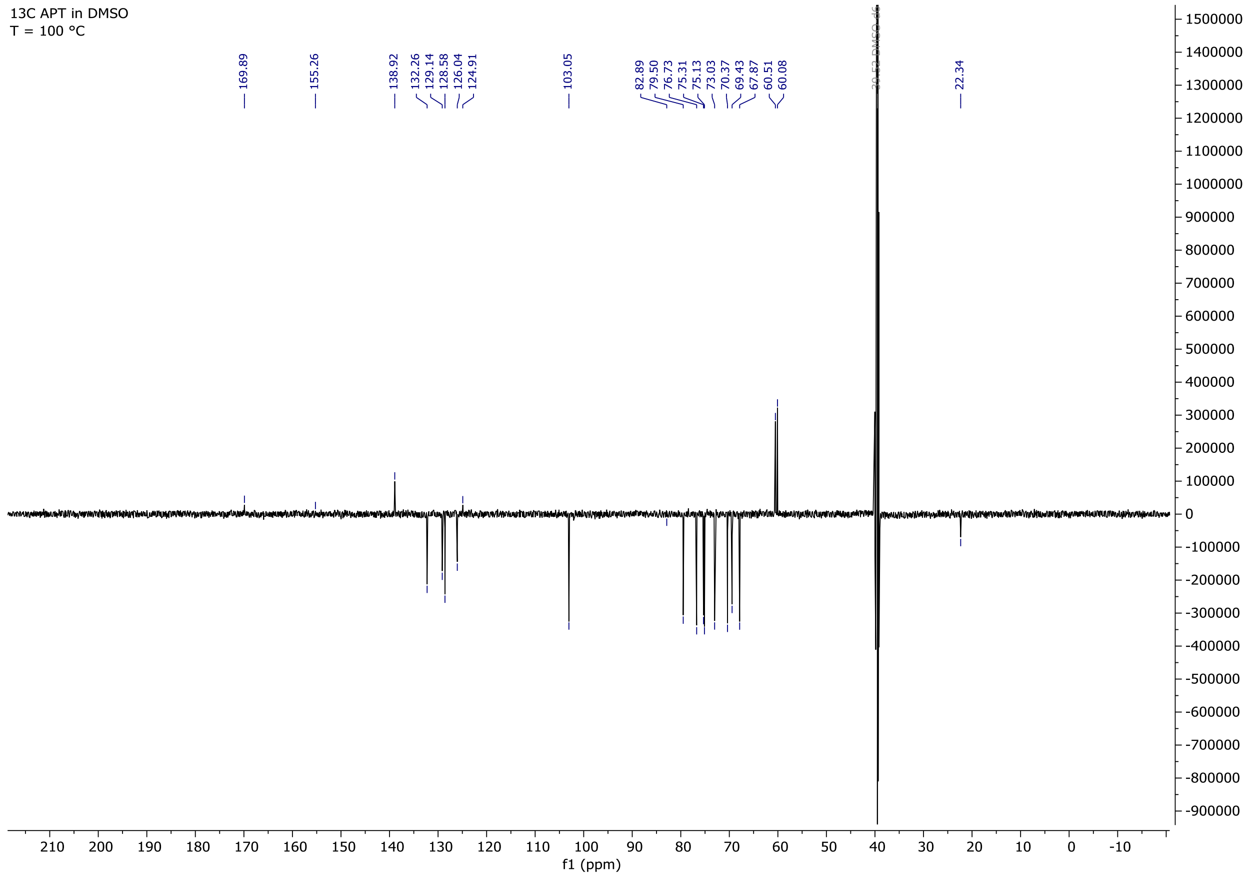

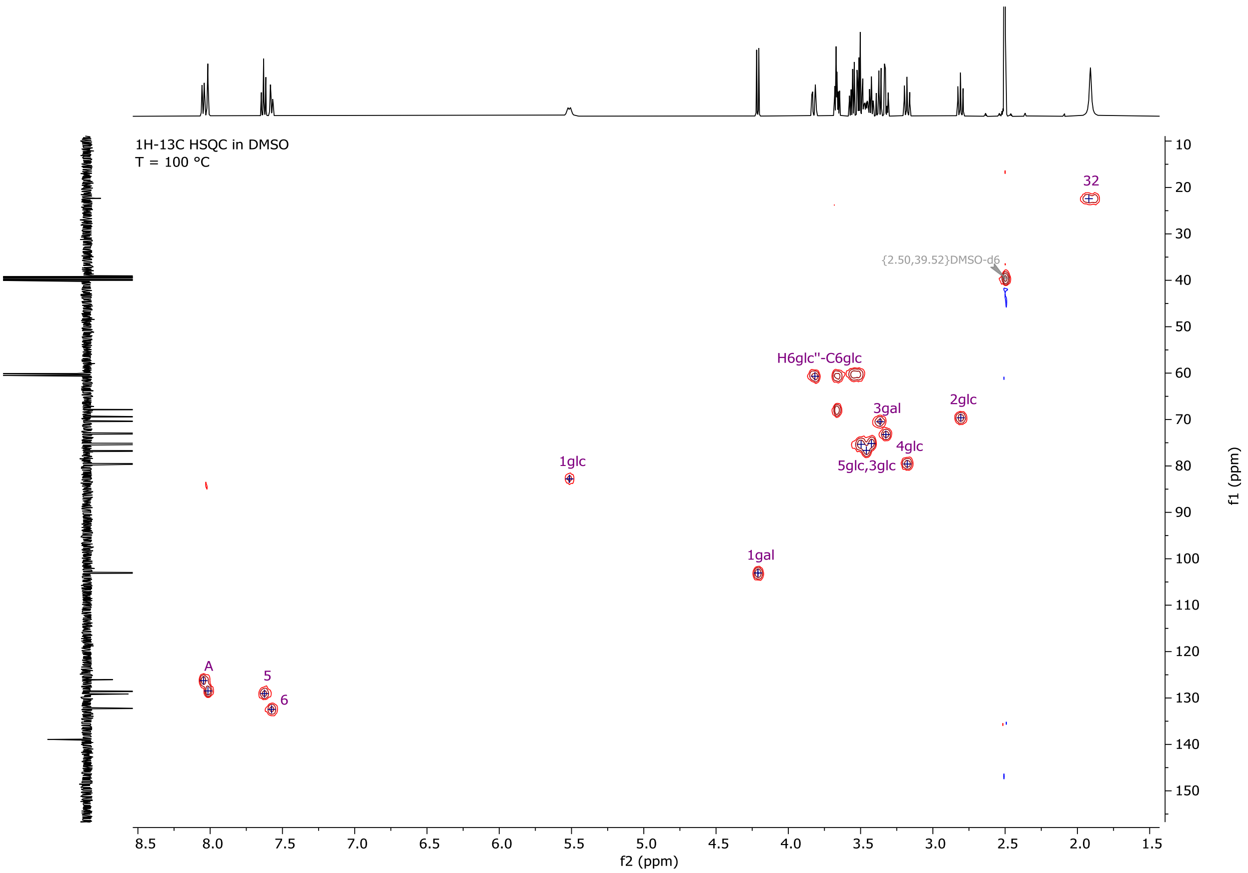
1H-13C HSQC in DMSO
T = 100 °C
32
{2.50,39.52}DMSO-d6
H6glc''-C6glc
3gal
2glc
4glc
5glc,3glc
1glc
1gal
A
5
6
f2 (ppm)
f1 (ppm)

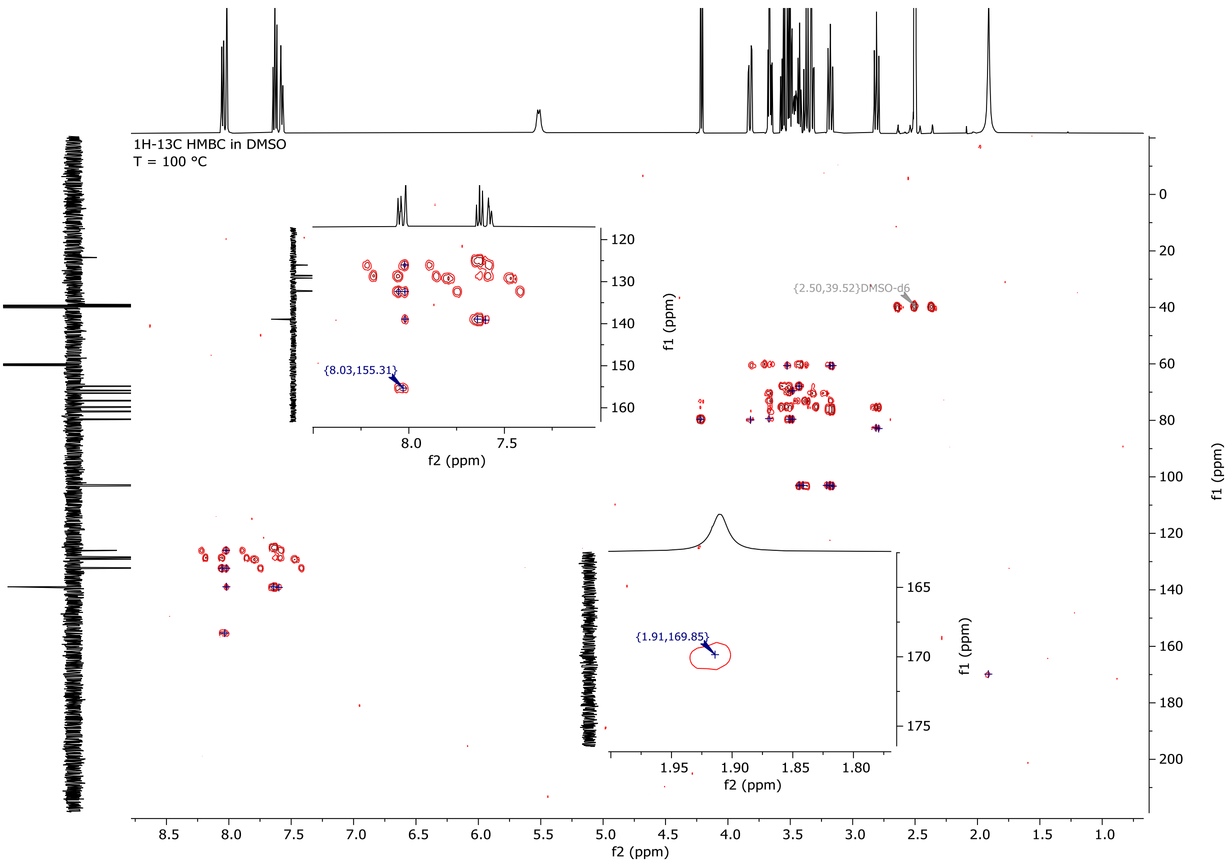
1H-13C HMBC in DMSO
T = 100 °C
{2.50,39.52}DMSO-d6
{8.03,155.31}
{1.91,169.85}
f2 (ppm)
f1 (ppm)

^{1}H, ^{13}C APT and HSQC NMR spectra of (3z)

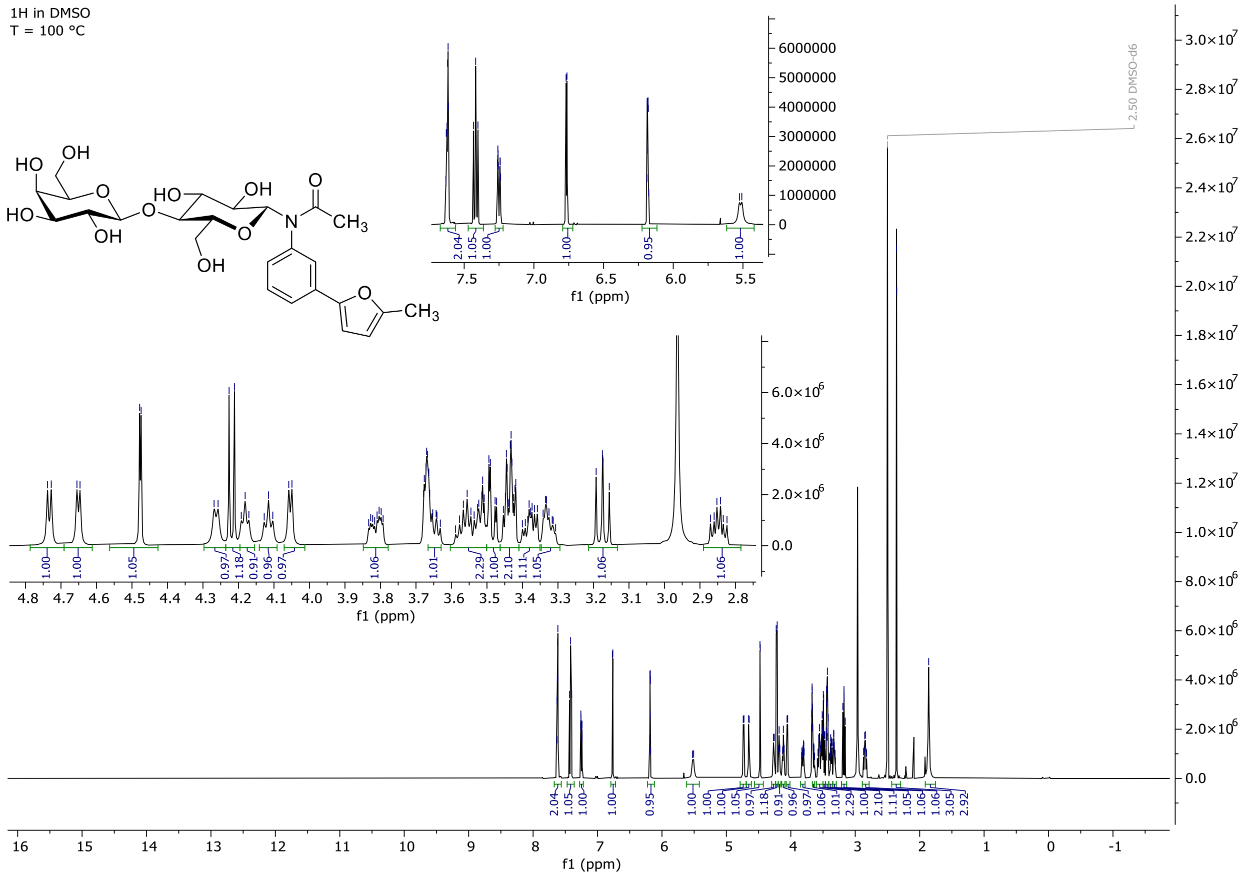

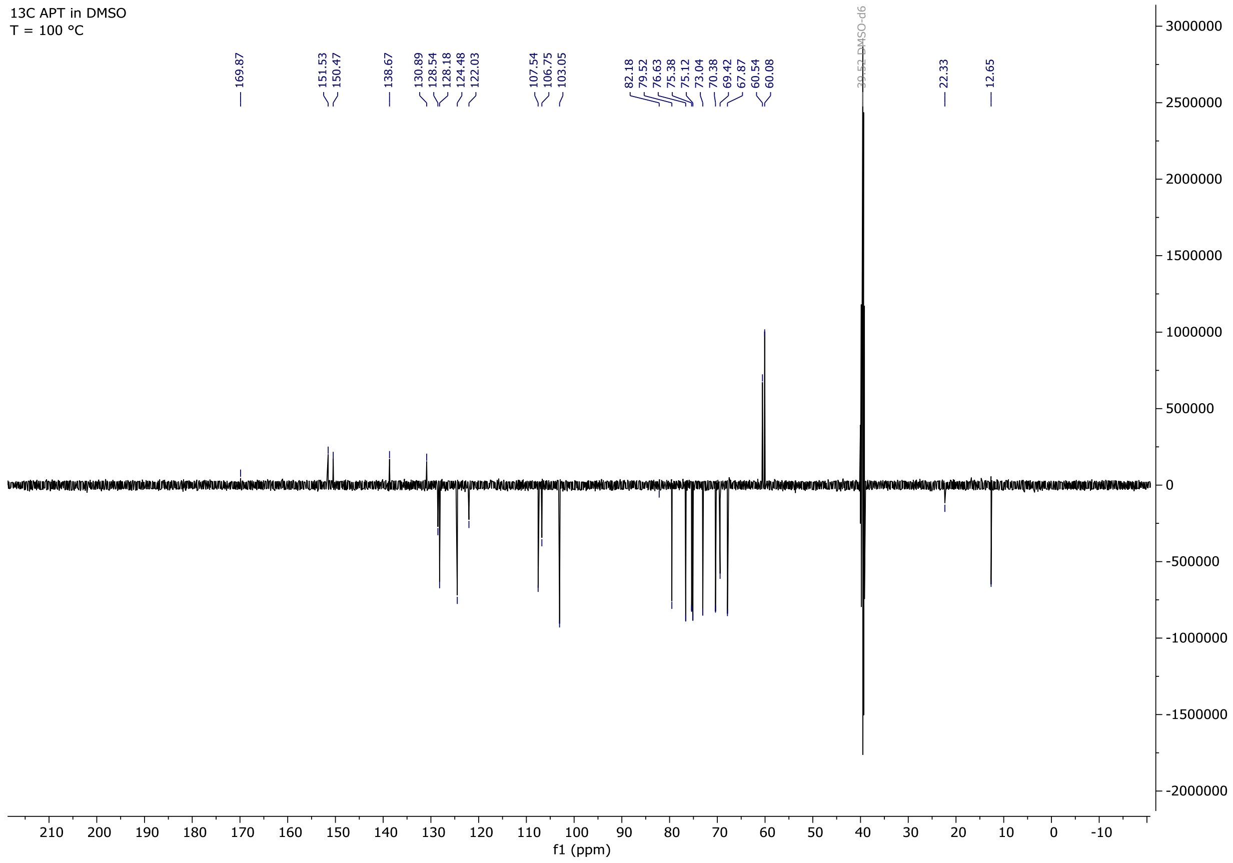

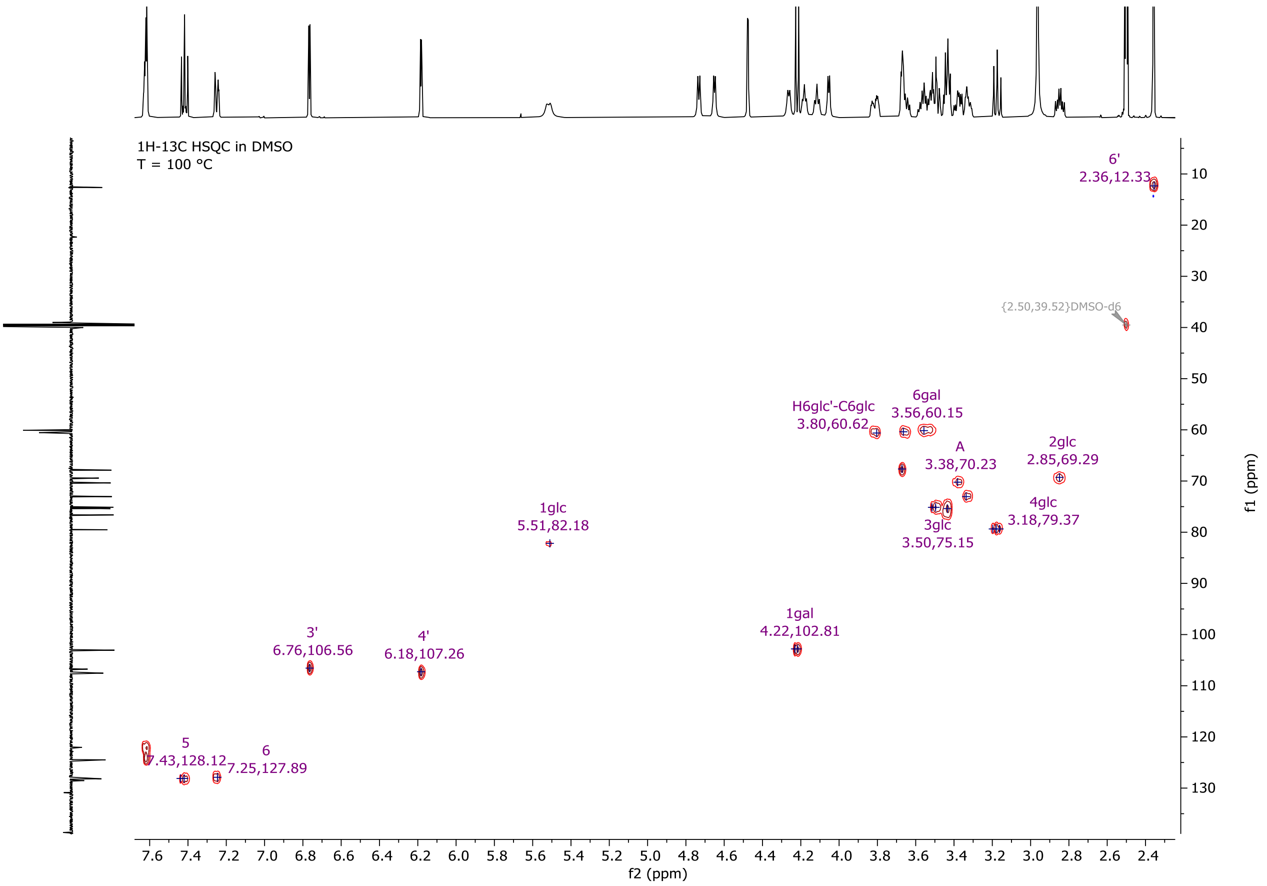
1H-13C HSQC in DMSO
T = 100 °C
6'
2.36,12.33
{2.50,39.52}DMSO-d6
6gal
3.56,60.15
H6glc'-C6glc
3.80,60.62
A
3.38,70.23
2glc
2.85,69.29
4glc
3.18,79.37
3glc
3.50,75.15
1glc
5.51,82.18
1gal
4.22,102.81
3'
6.76,106.56
4'
6.18,107.26
5
7.43,128.12
6
7.25,127.89
f2 (ppm)
f1 (ppm)

^{1}H and ^{13}C APT NMR spectra of (3aa)

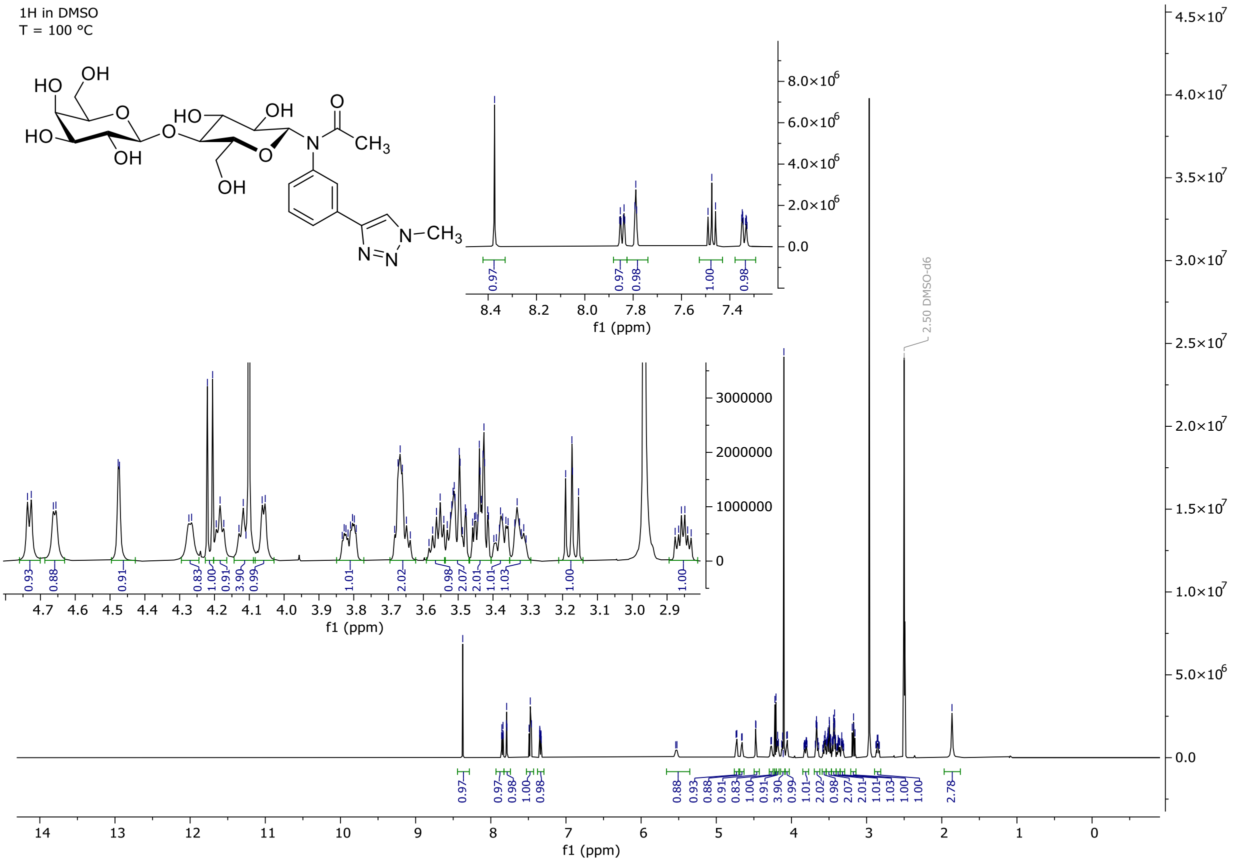

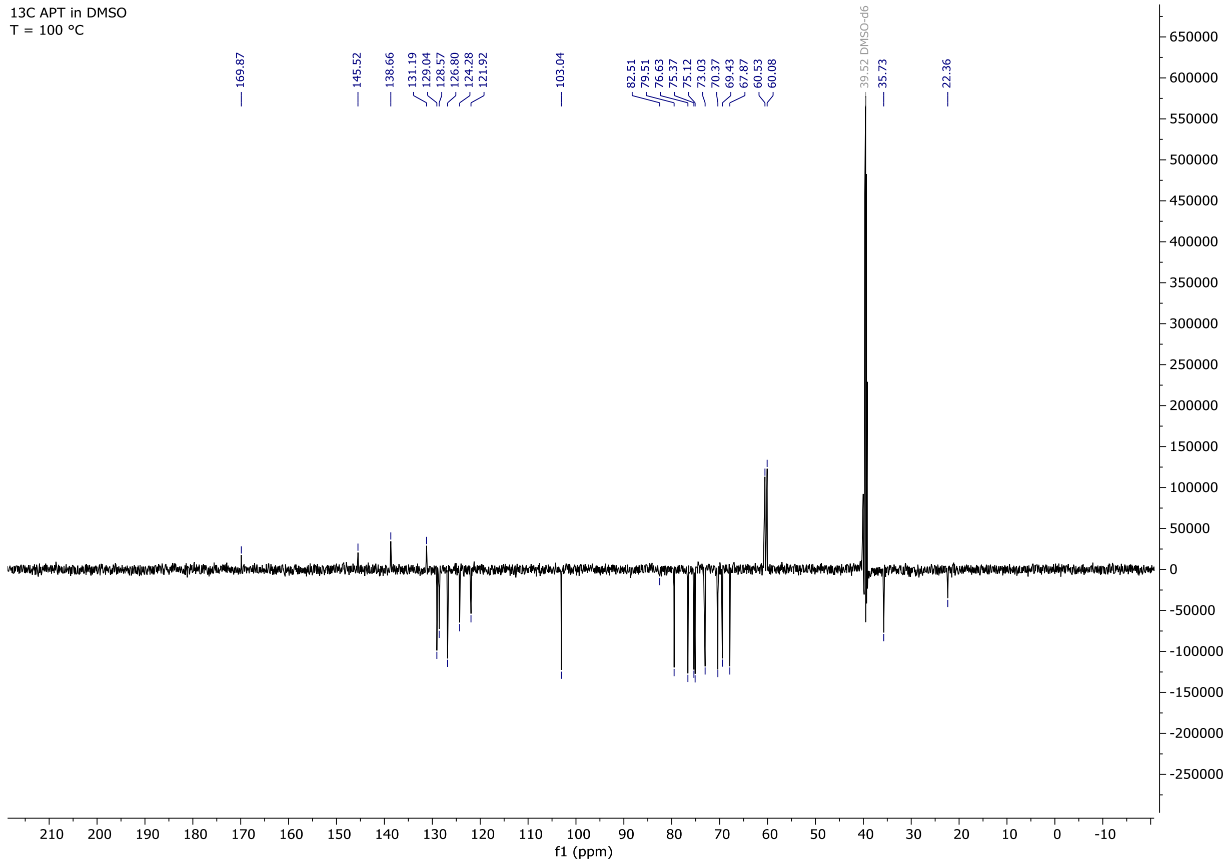

^{1}H and ^{13}C APT NMR spectra of (3ab)

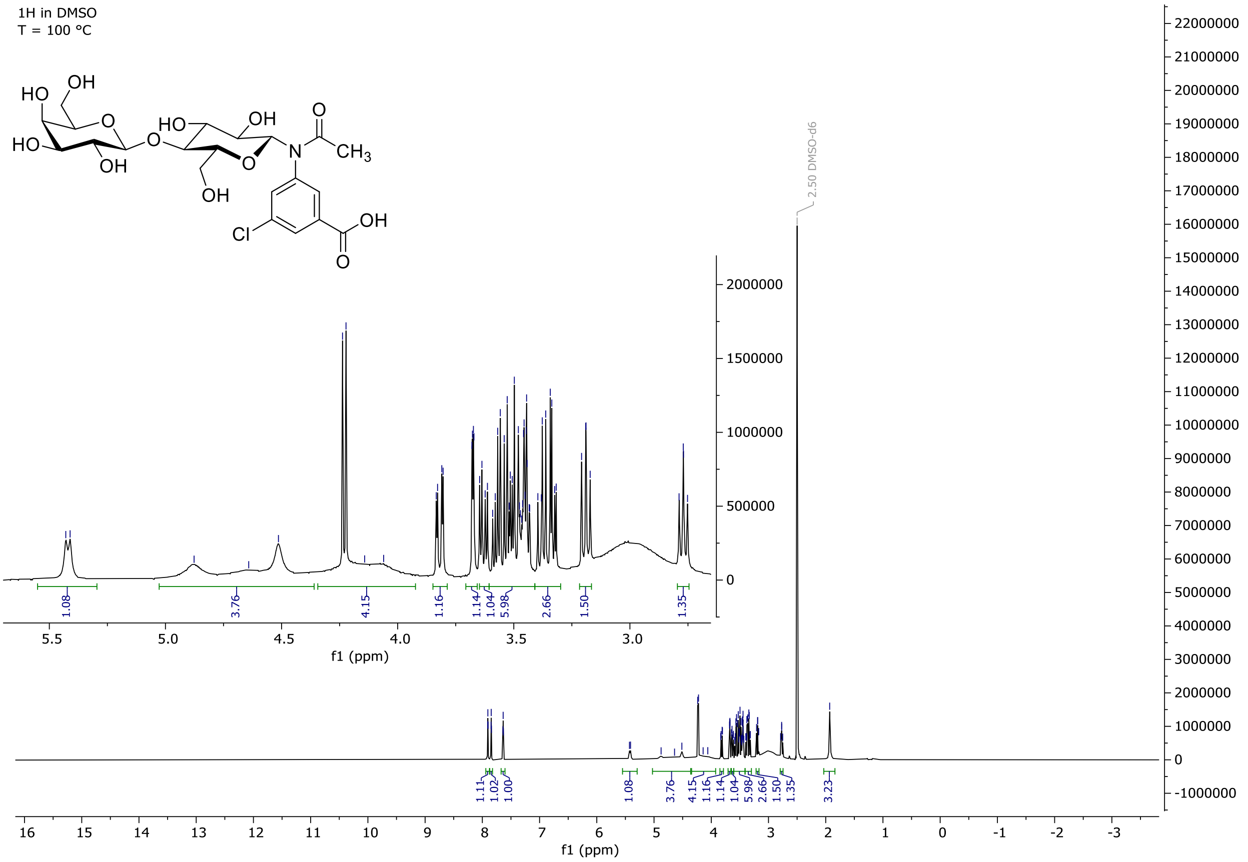

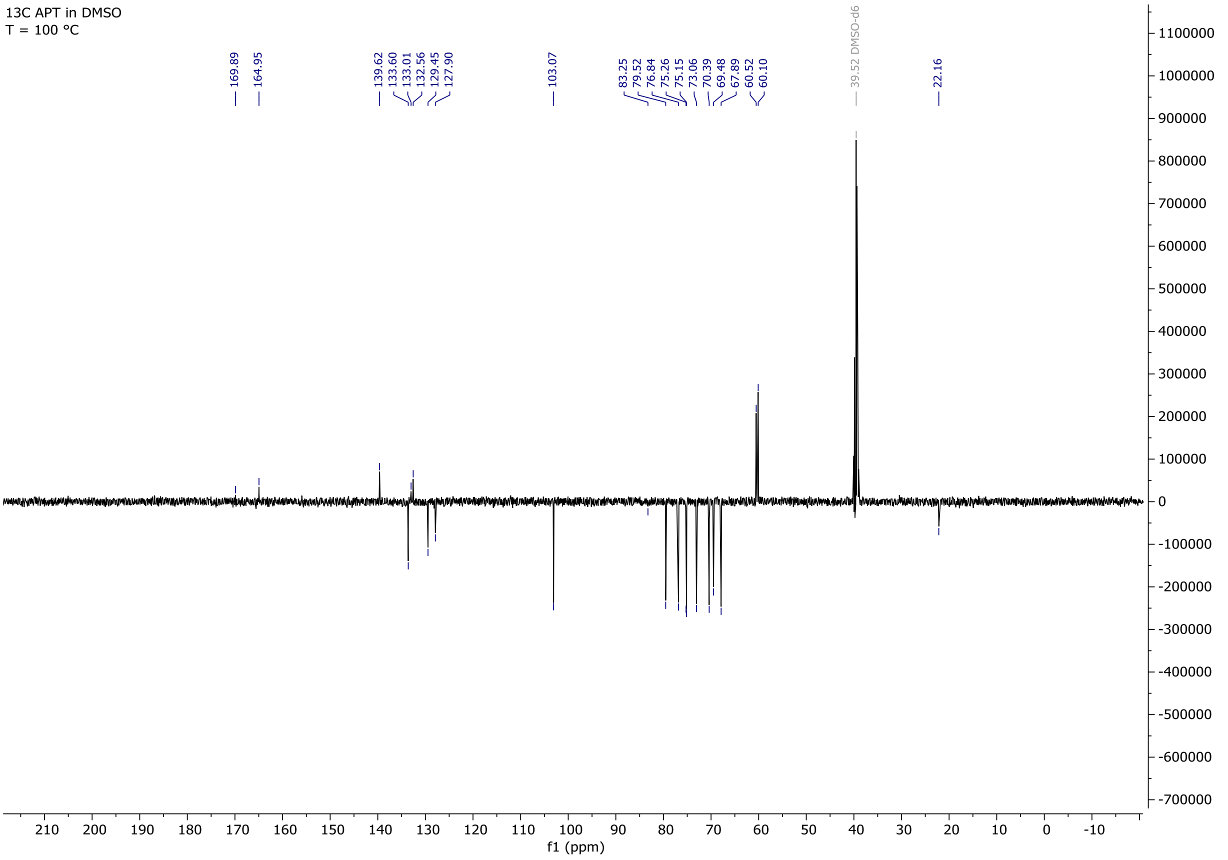

^1H and ^{13}C APT NMR spectra of (3ac)

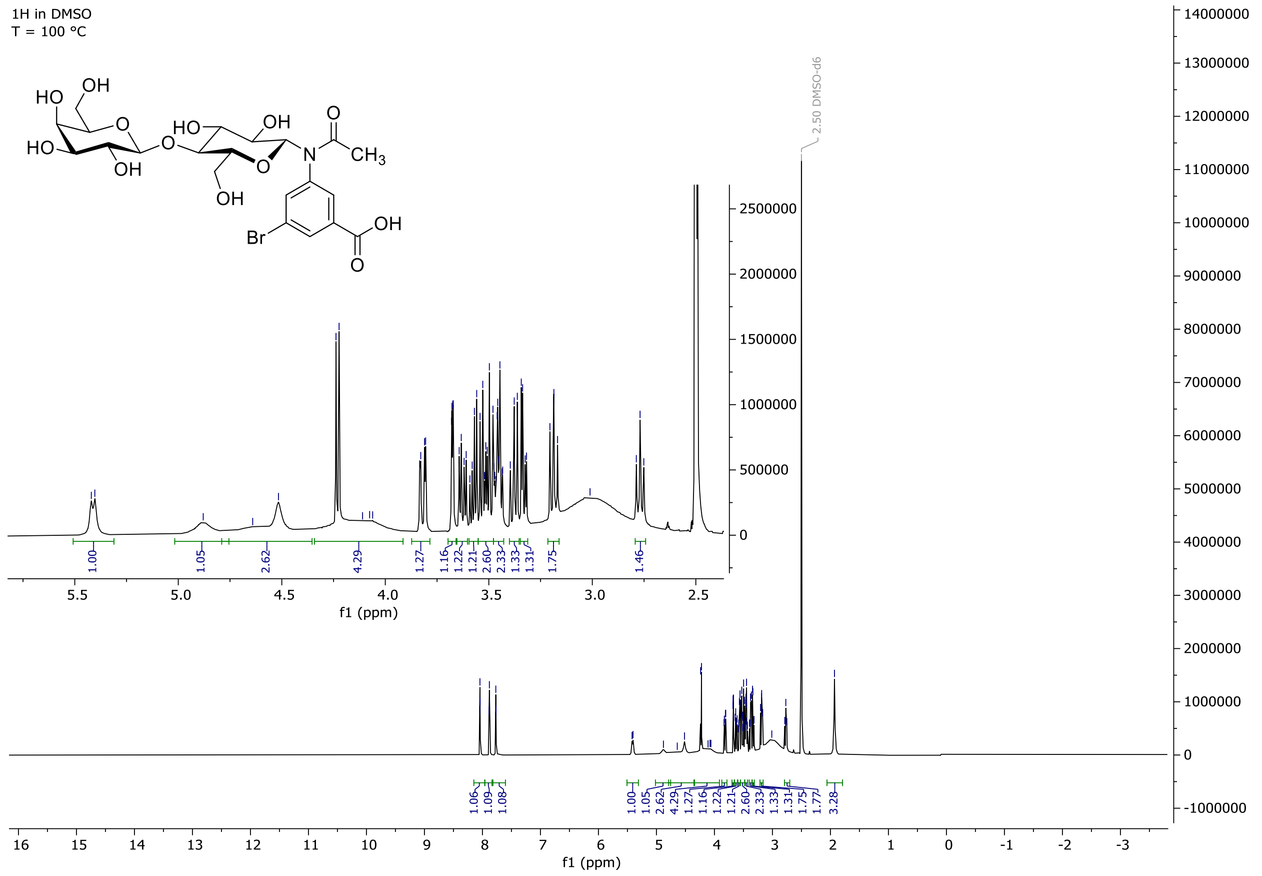

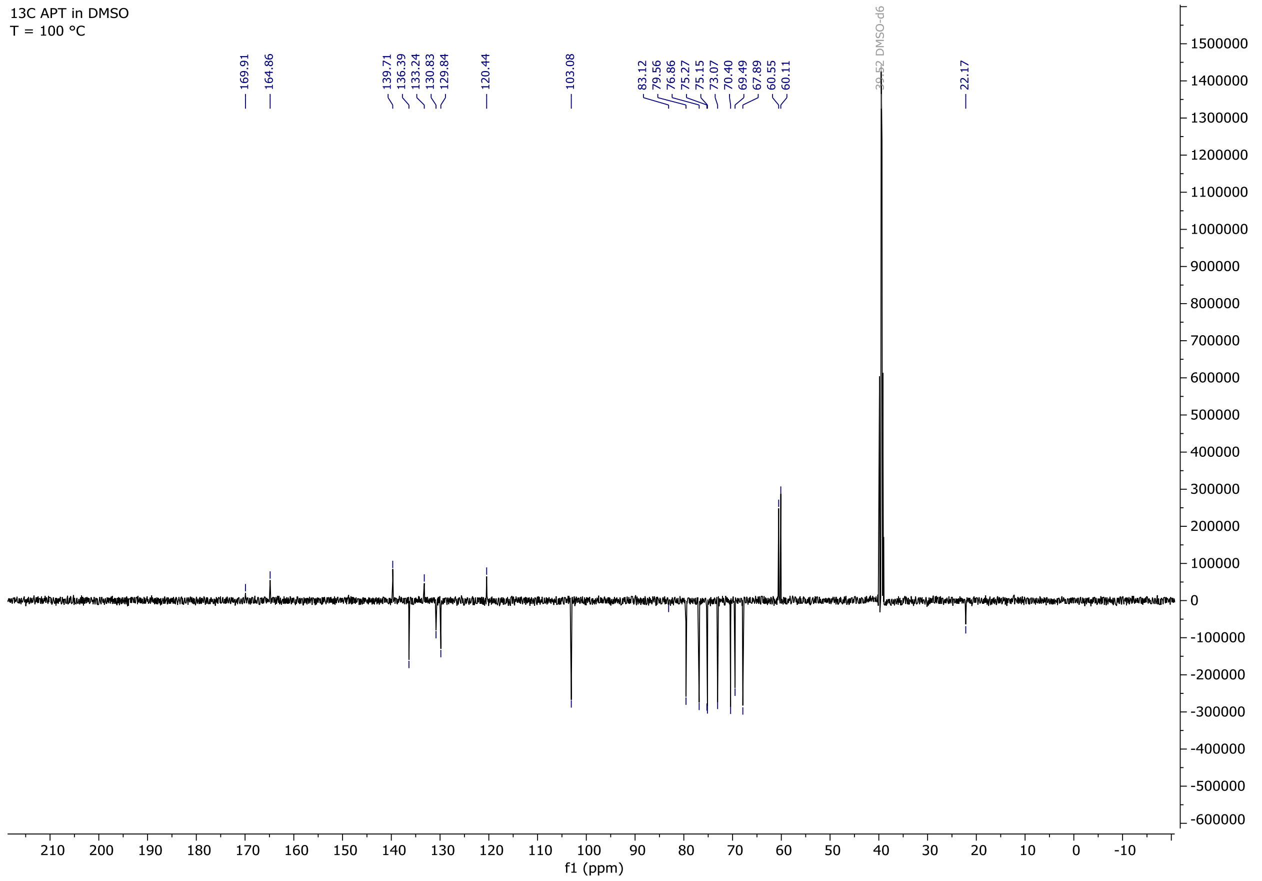

^{1}H and ^{13}C APT NMR spectra of (3ad)

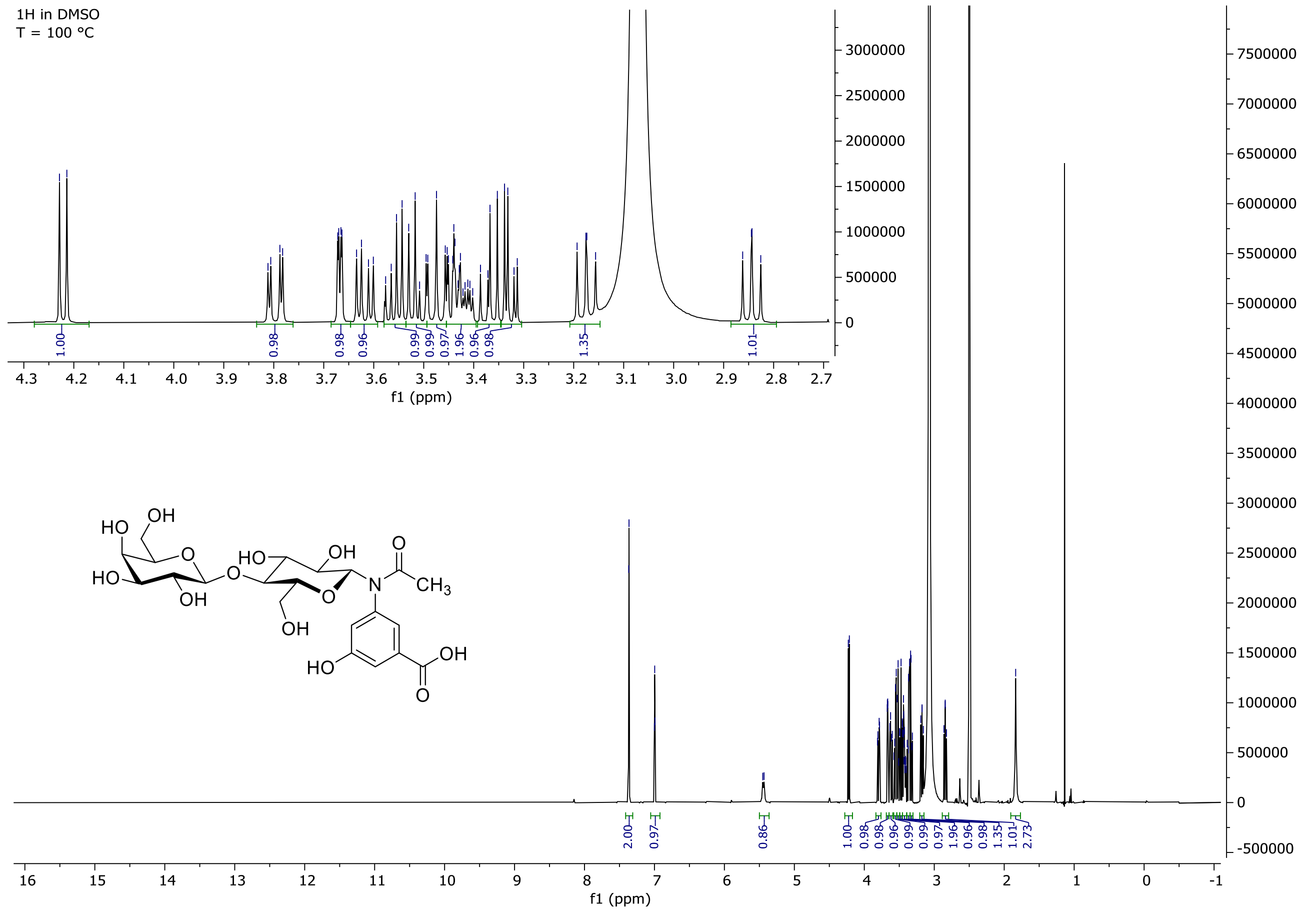

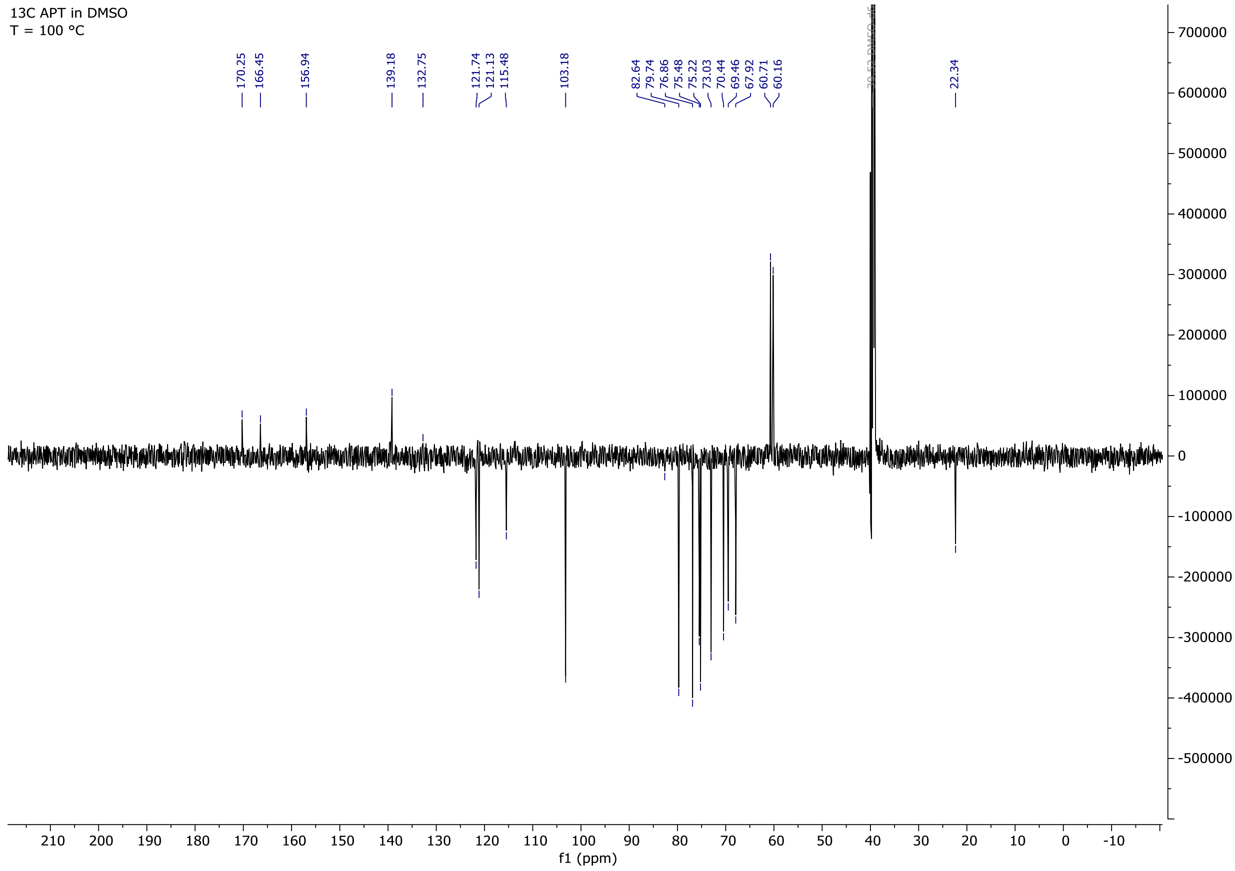

^{1}H, ^{13}C APT and HSQC NMR spectra of (3ae)

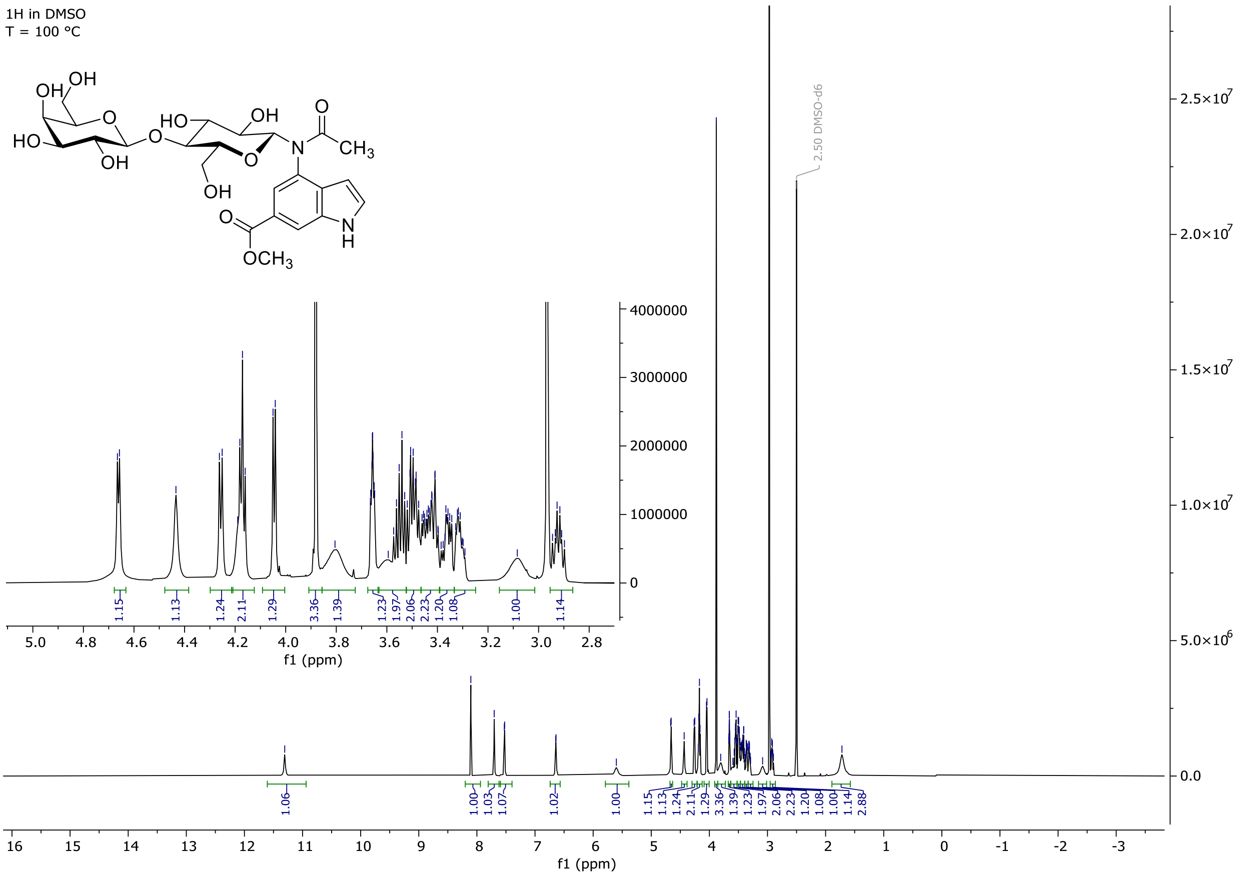

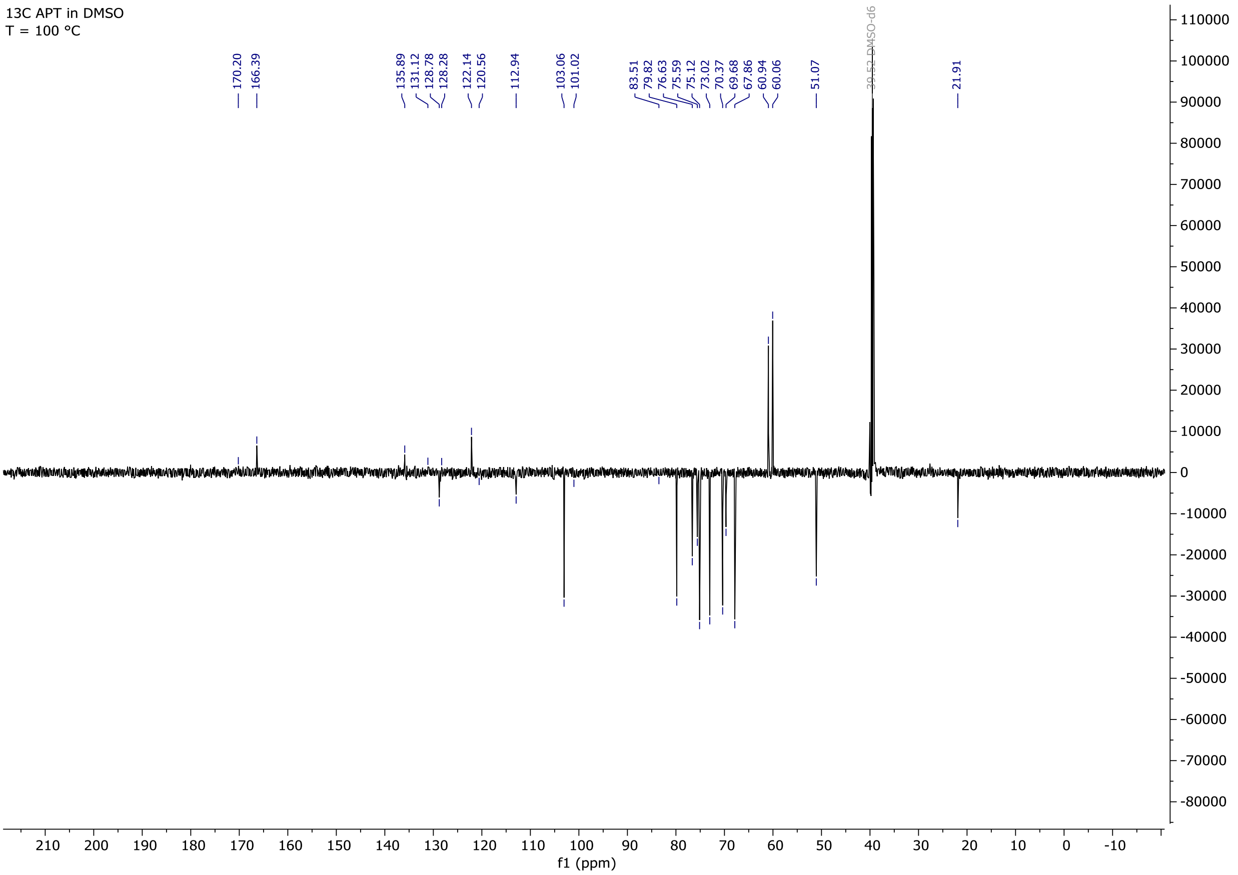

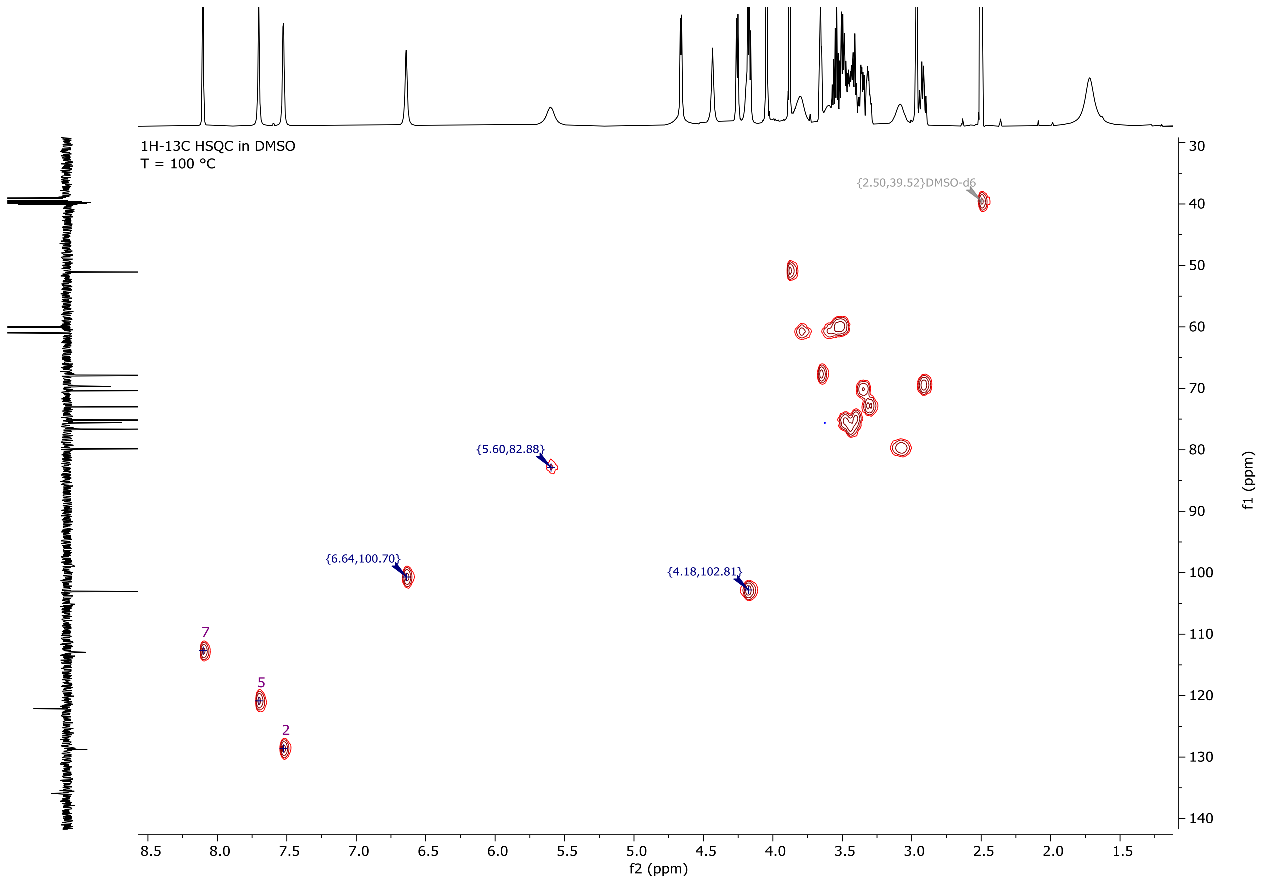

1H-13C HSQC in DMSO
T = 100 °C
{2.50,39.52}DMSO-d6
{5.60,82.88}
{6.64,100.70}
{4.18,102.81}
7
5
2
f2 (ppm)
f1 (ppm)

^1H and ^{13}C APT NMR spectra of (3af)

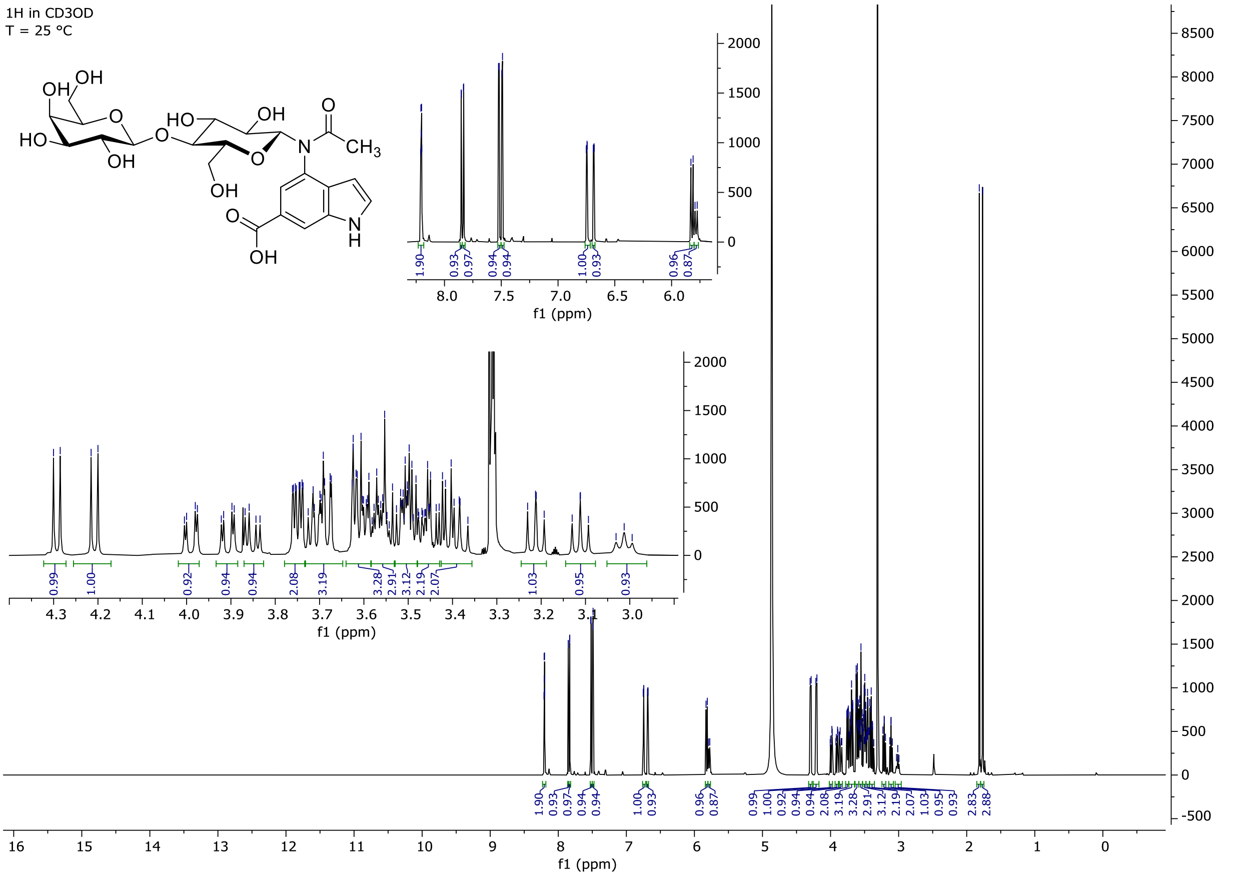

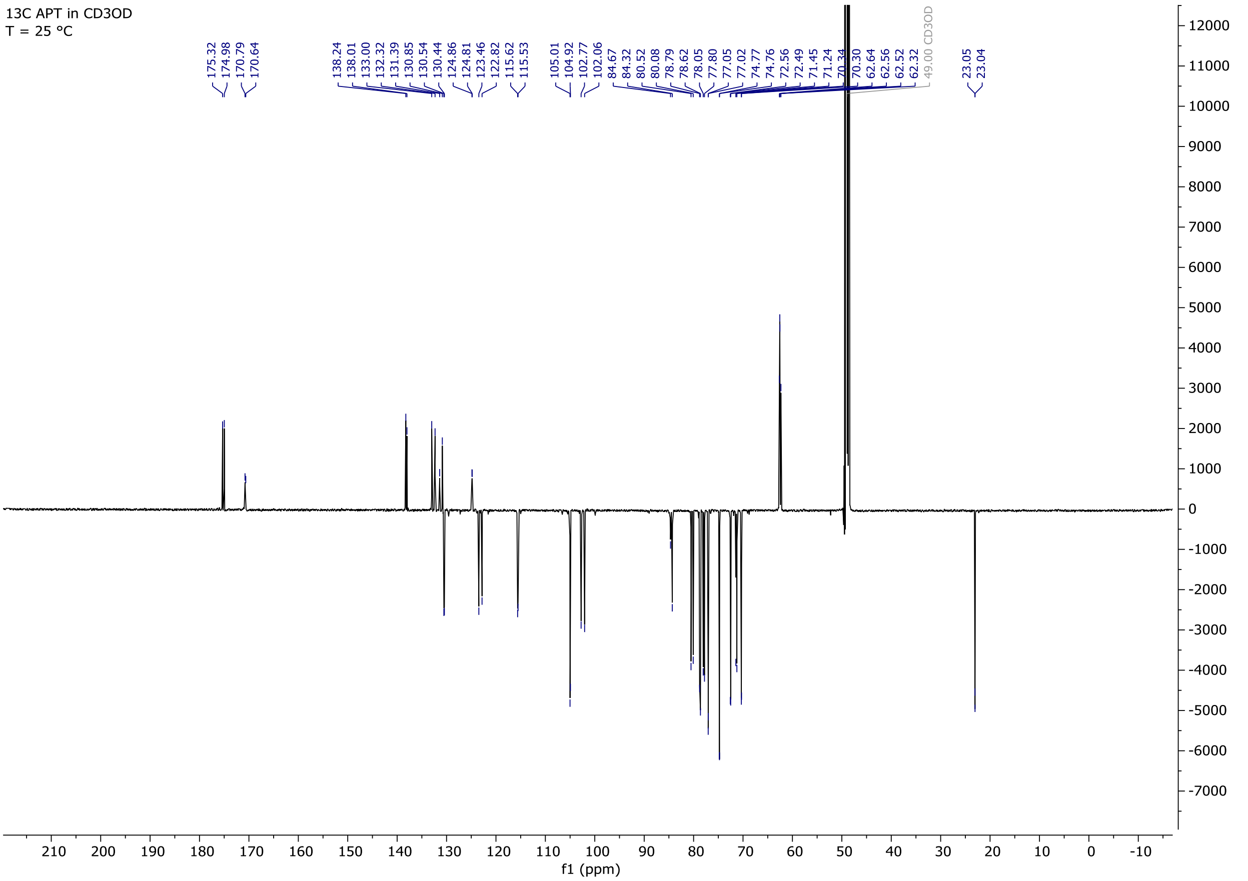

^{1}H, ^{13}C APT and ^{19}F NMR spectra of (6)

1H in Chloroform
T = 25 °C

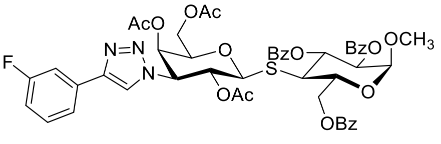

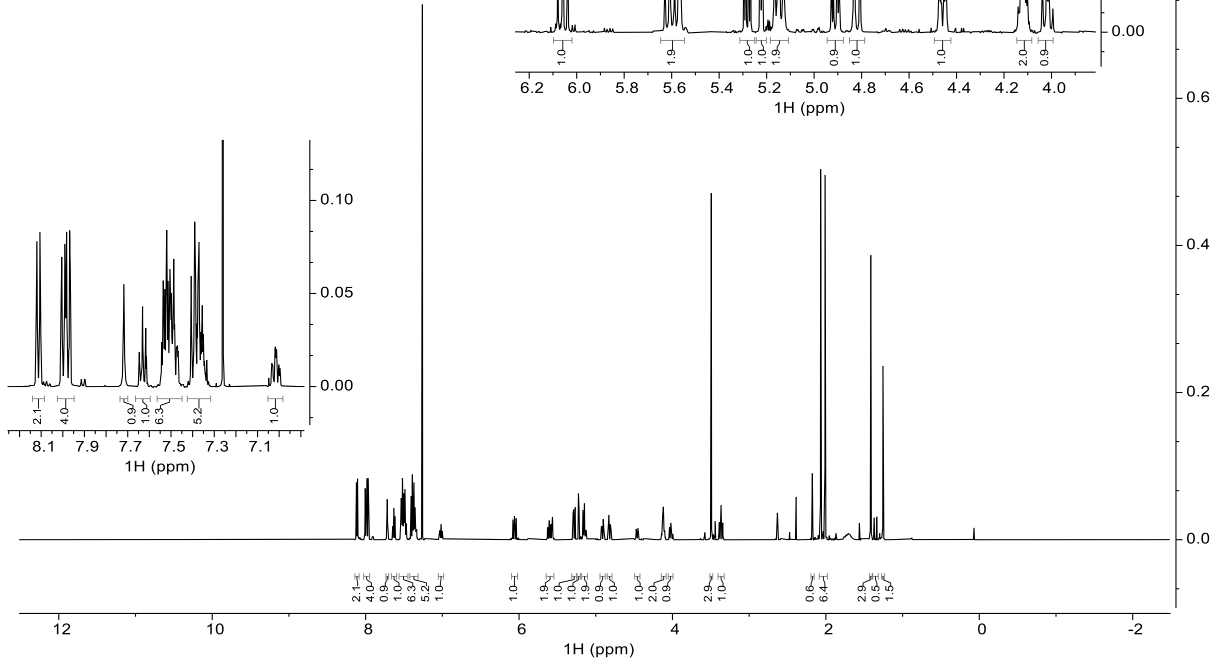

13C APT in Chloroform
T = 25 °C

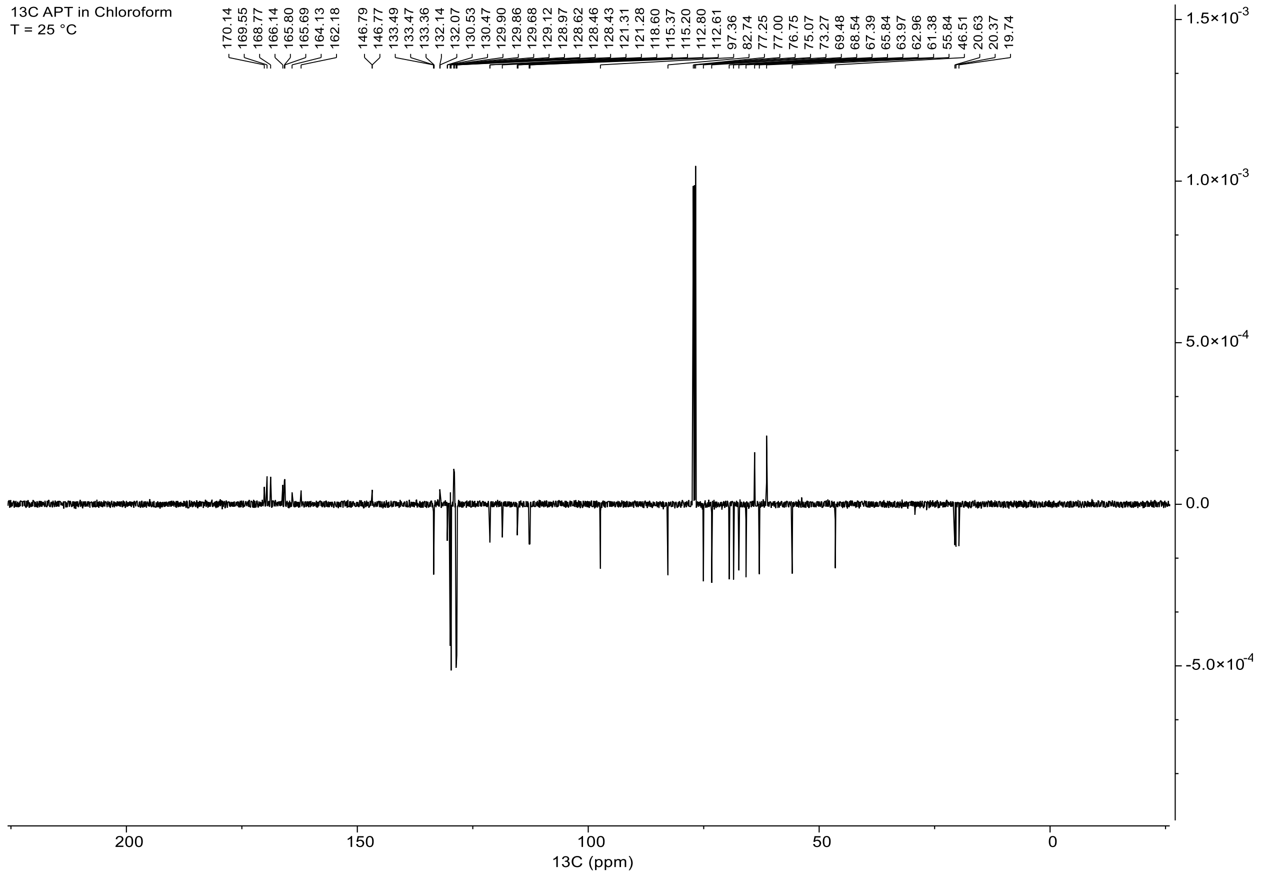

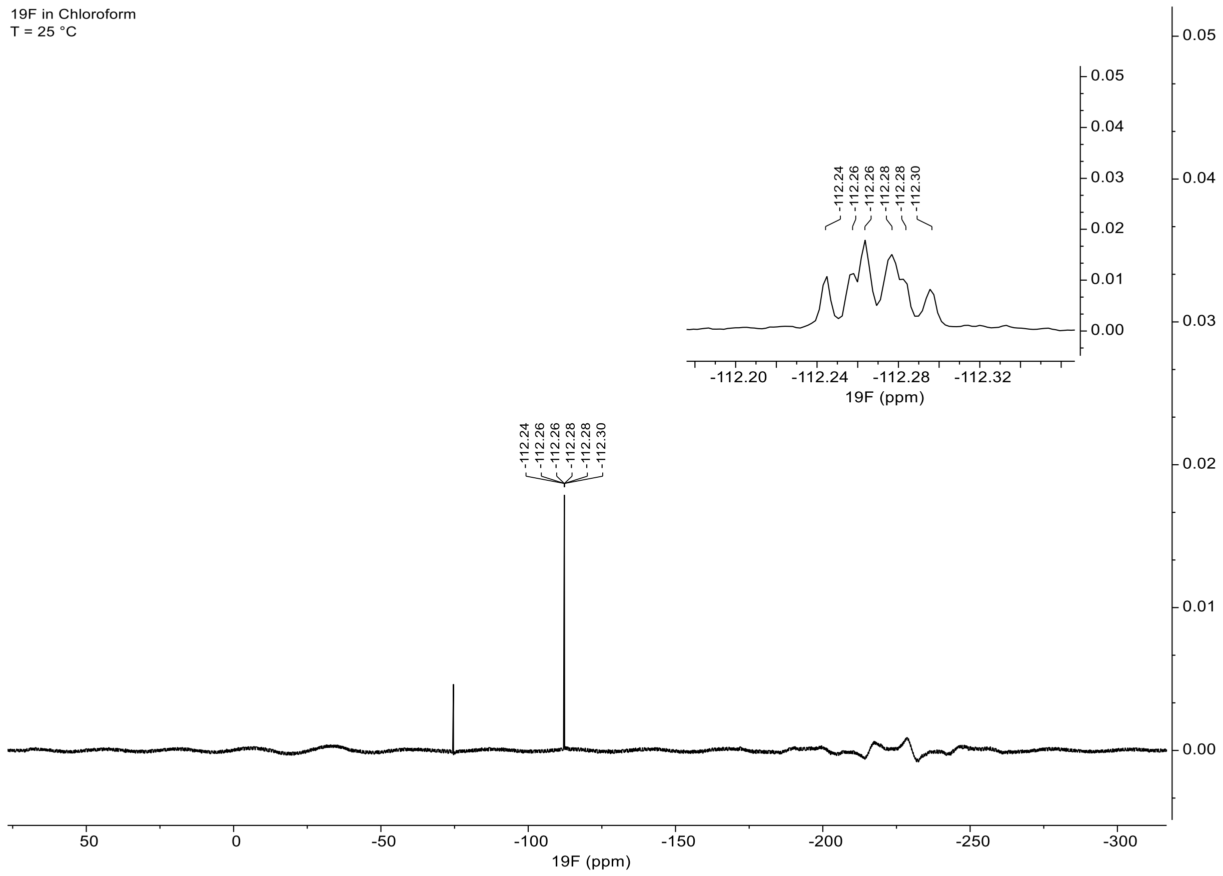

19F in Chloroform
T = 25 °C
-112.24
-112.26
-112.26
-112.28
-112.28
-112.30
0.05
0.04
0.03
0.02
0.01
0.00
-112.20
-112.24
-112.28
-112.32
19F (ppm)
50
0
-50
-100
-150
-200
-250
-300
19F (ppm)

^{1}H, ^{13}C APT and ^{19}F NMR spectra of (S4)

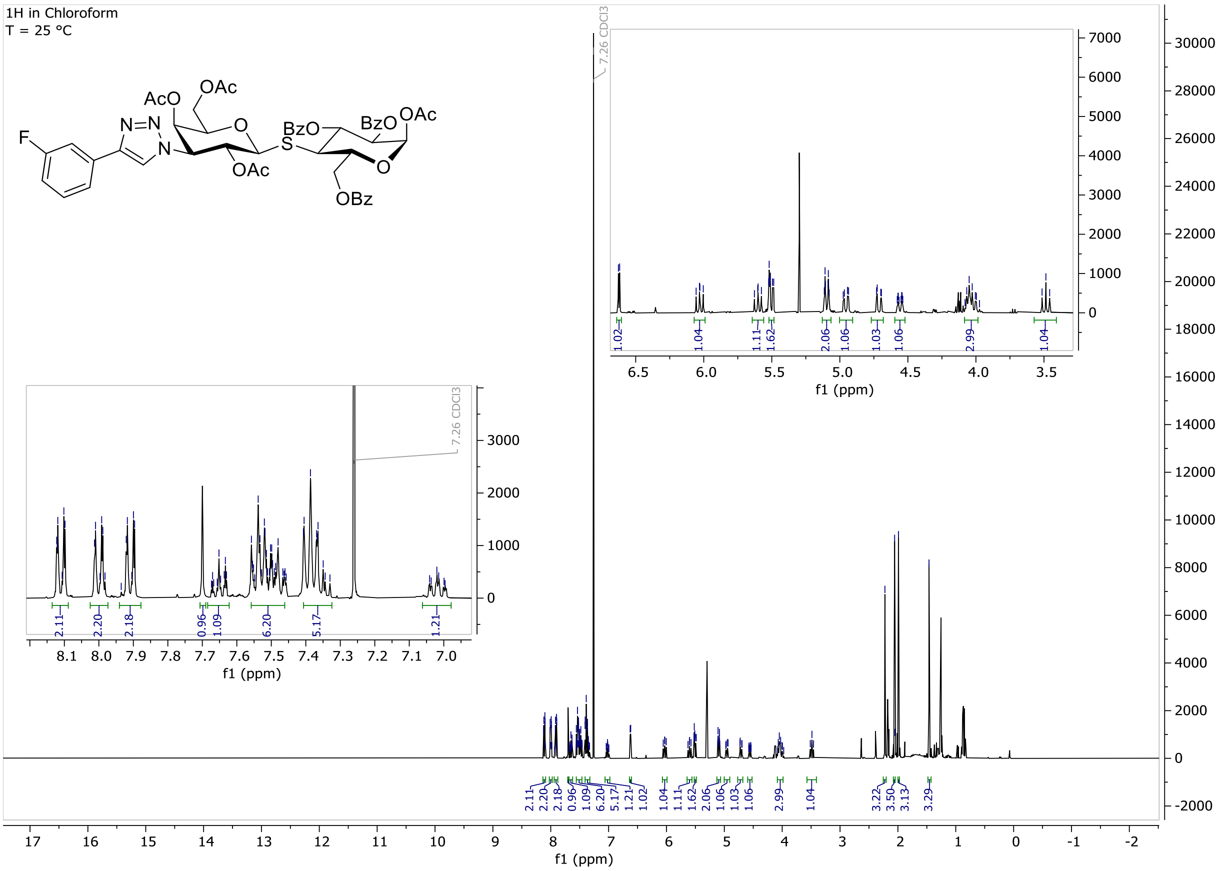

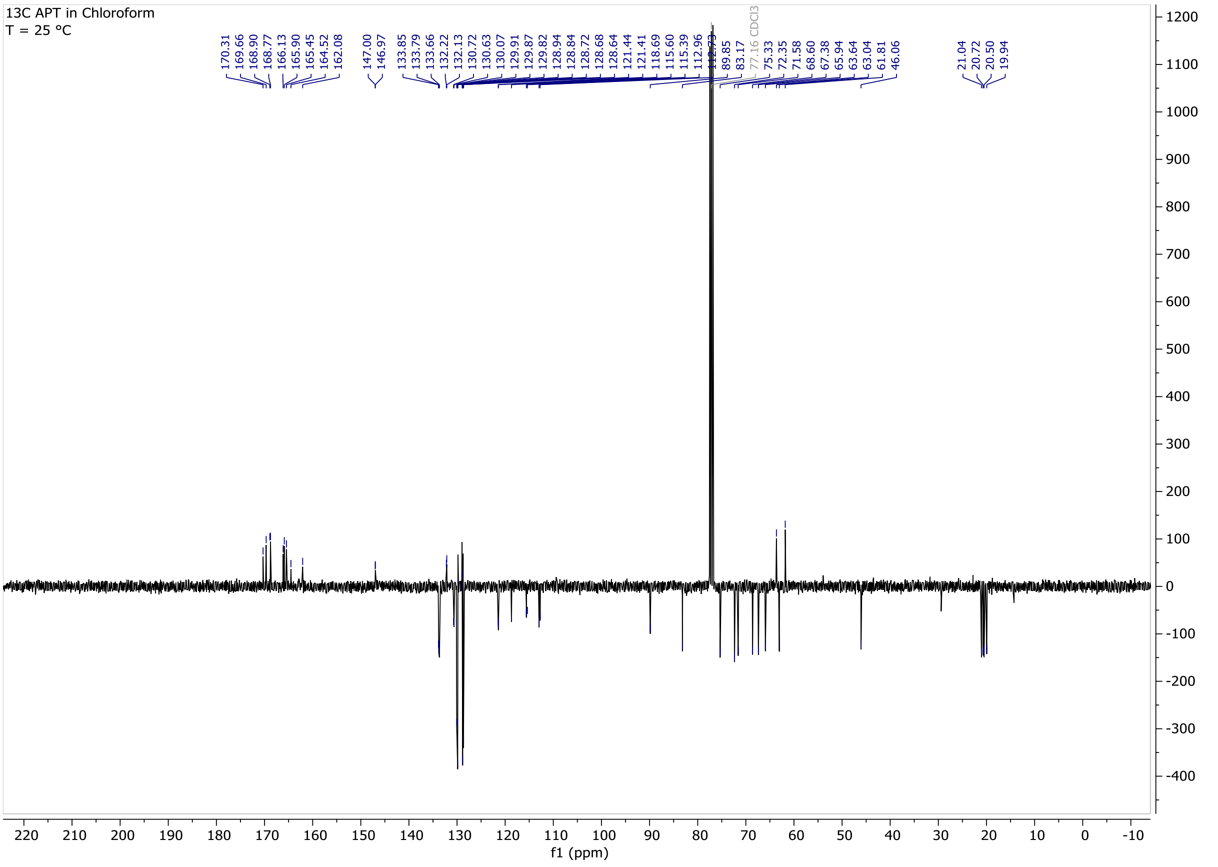

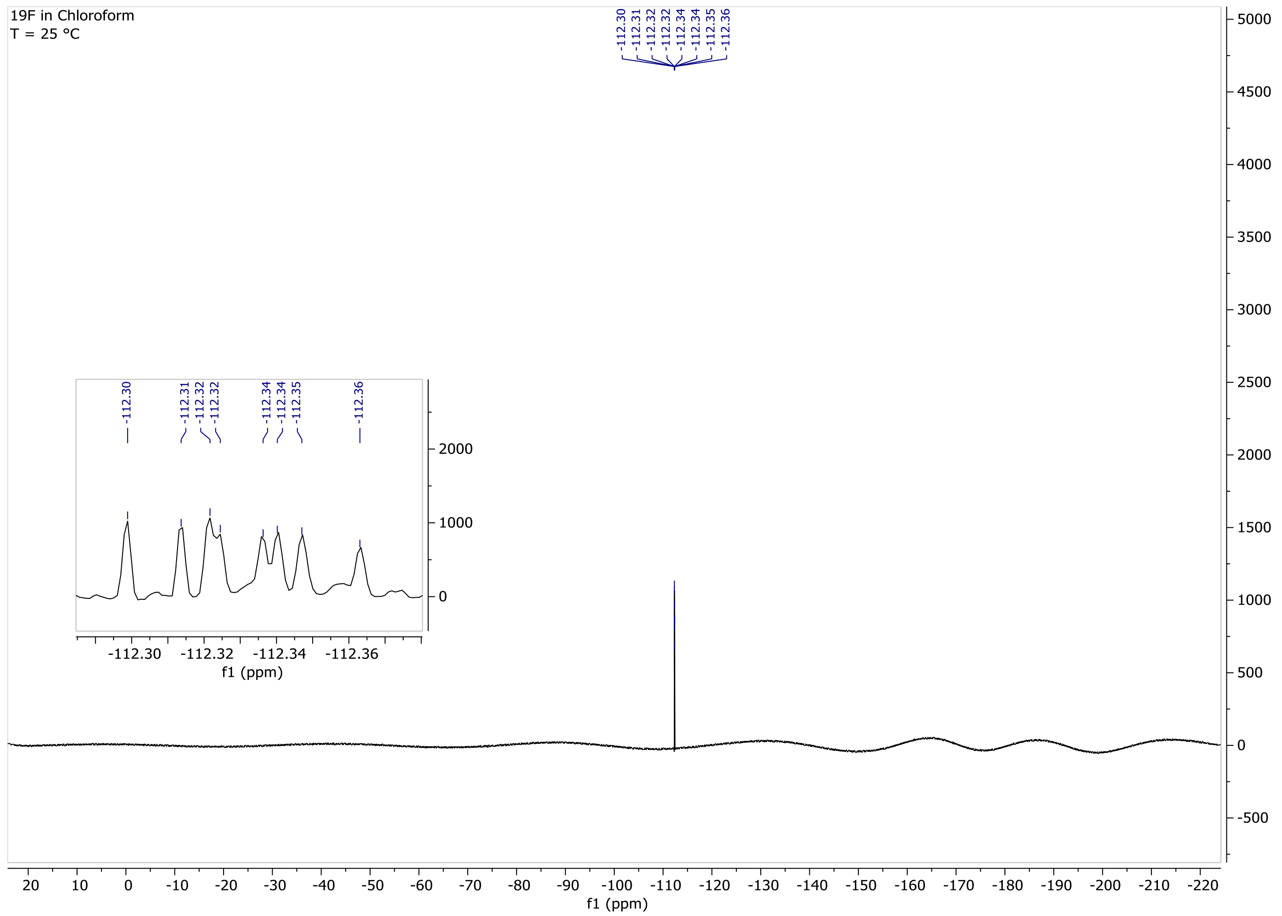
19F in Chloroform
T = 25 °C
-112.30
-112.31
-112.32
-112.32
-112.34
-112.34
-112.35
-112.36
f1 (ppm)

^{1}H, ^{13}C APT and ^{19}F NMR spectra of (7)

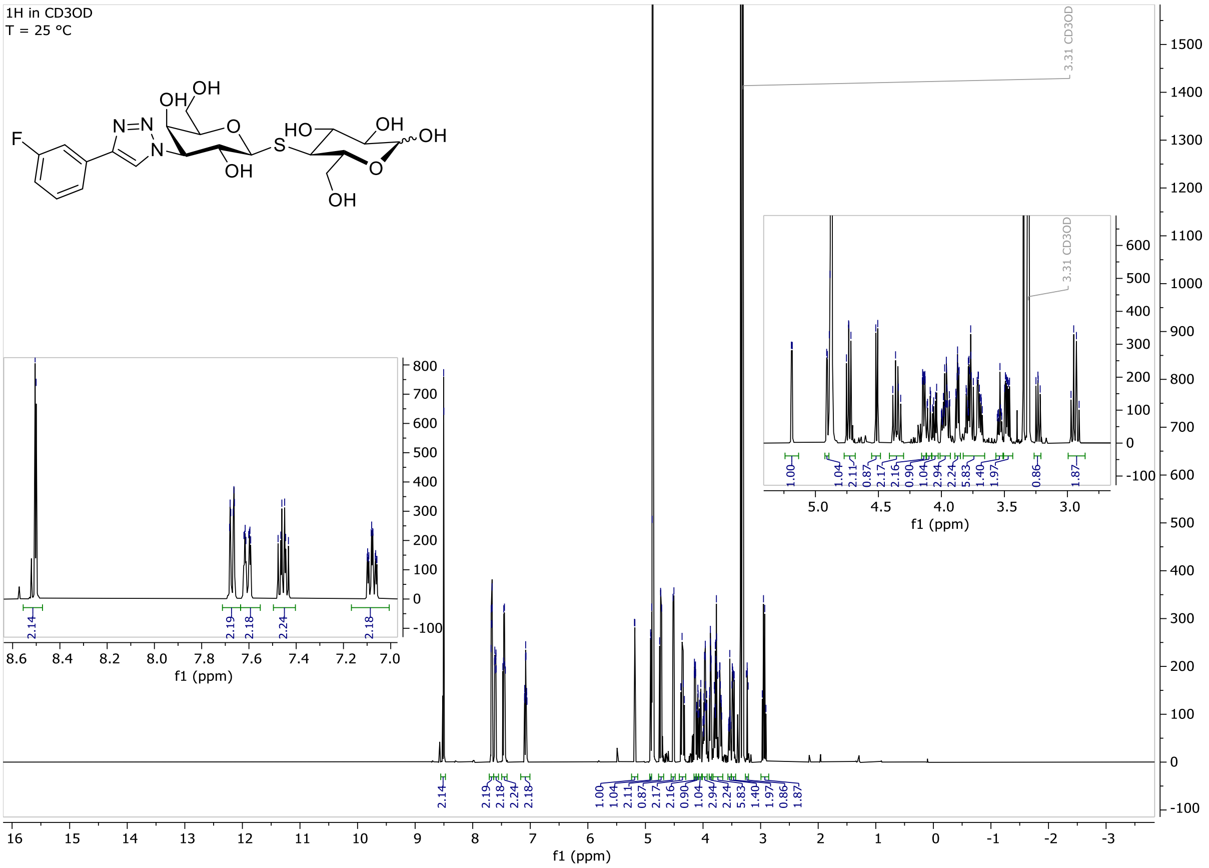

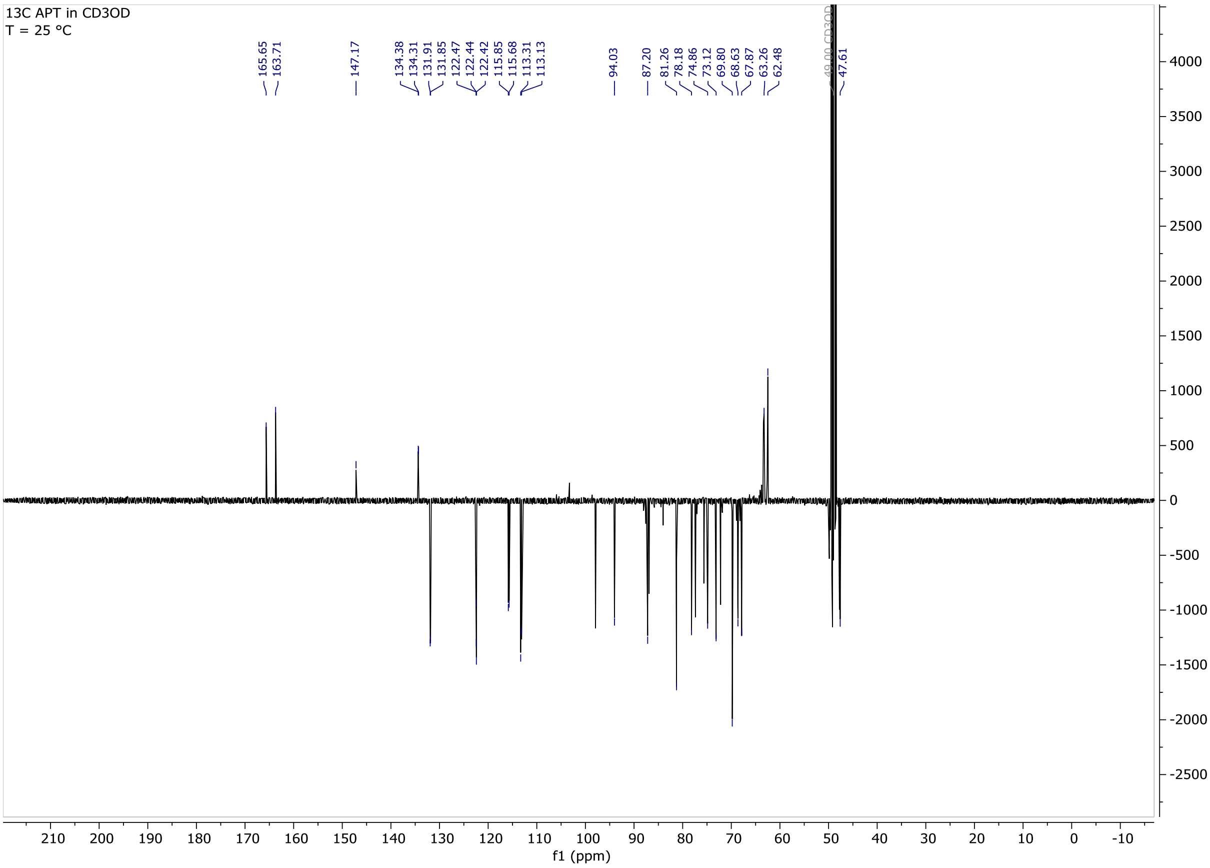

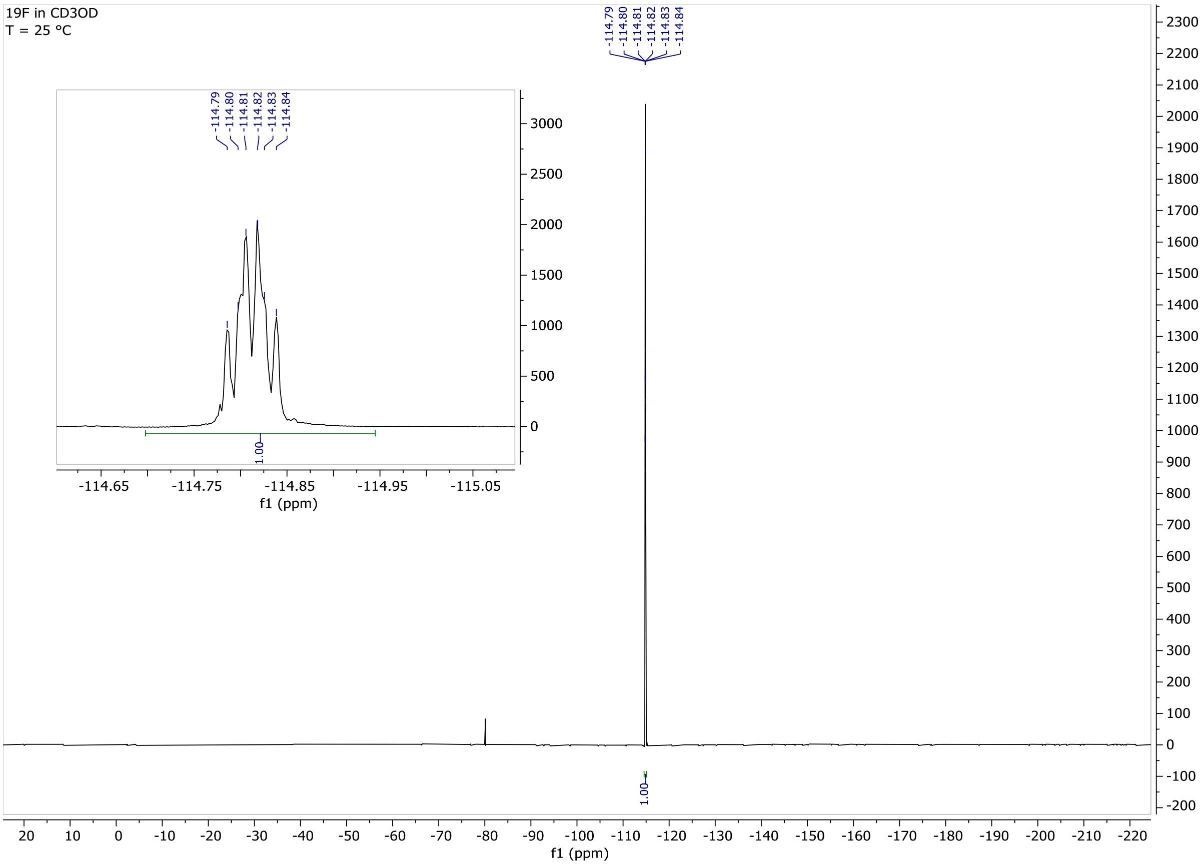
19F in CD3OD
T = 25 °C
-114.79
-114.80
-114.81
-114.82
-114.83
-114.84
1.00
f1 (ppm)

^{1}H, ^{13}C APT, ^{19}F NMR and HSQC spectra of (9)

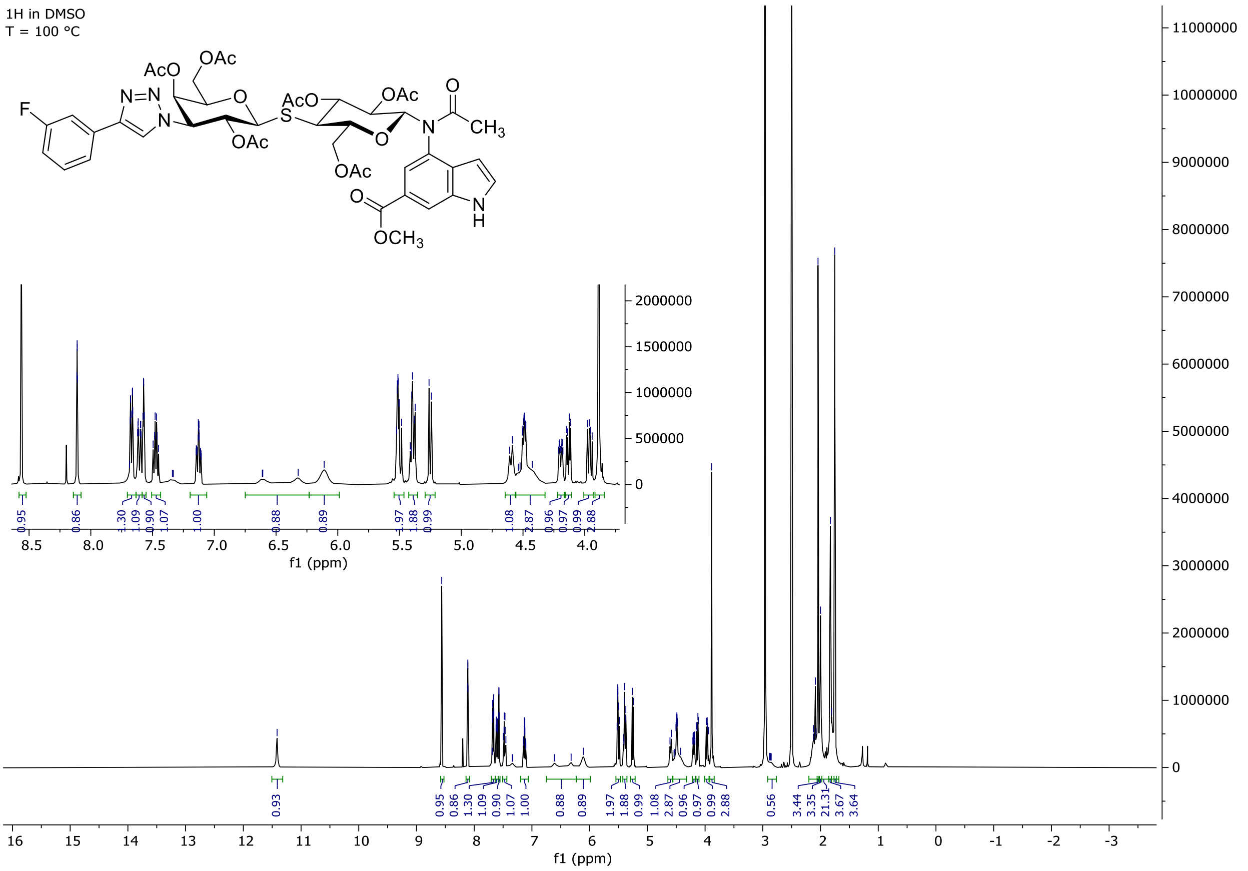

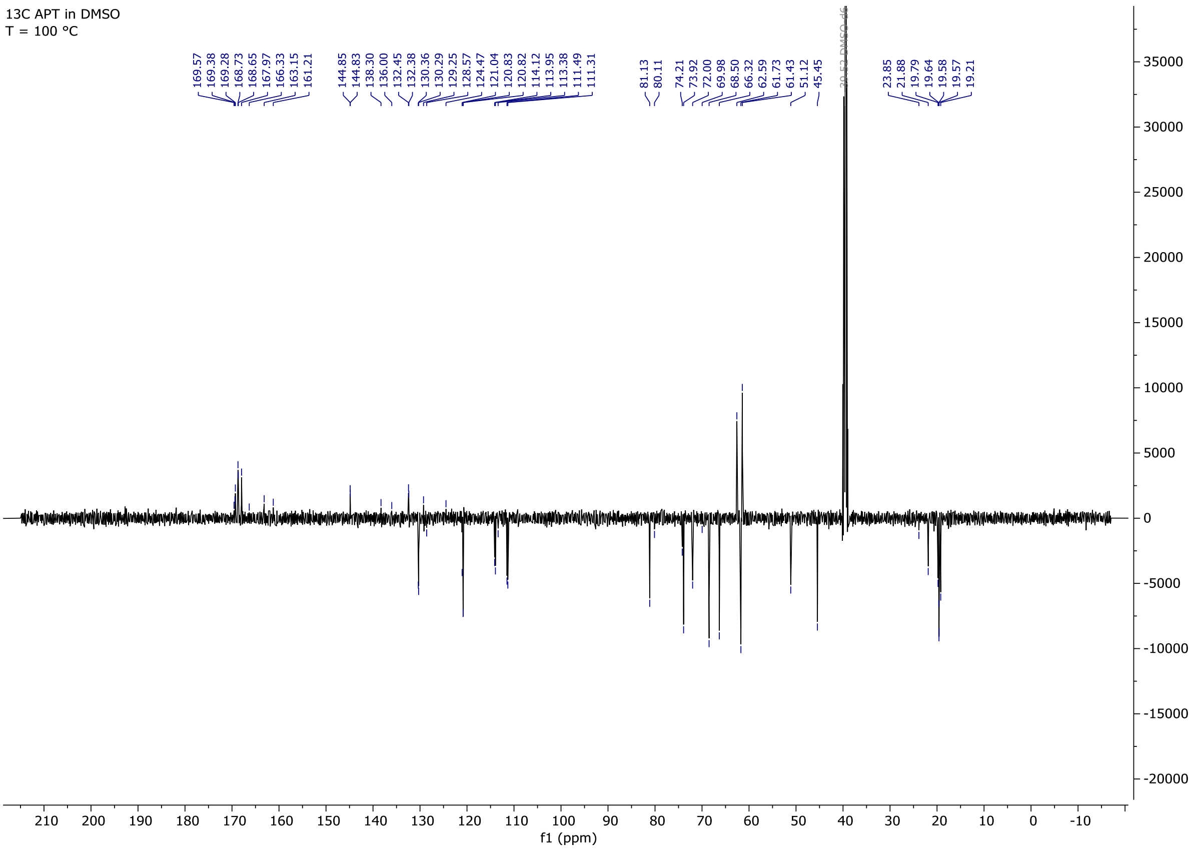

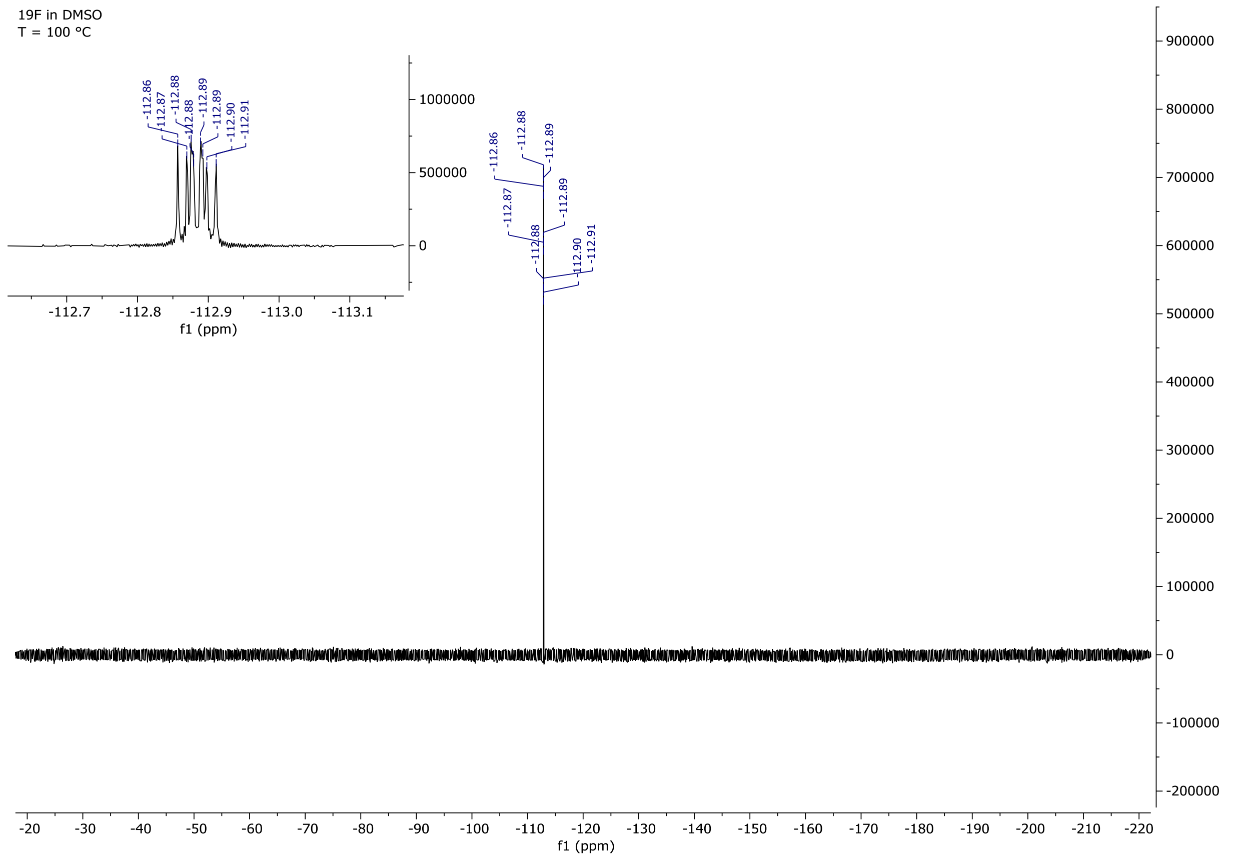
19F in DMSO
T = 100 °C
-112.86
-112.87
-112.88
-112.88
-112.89
-112.89
-112.90
-112.91
f1 (ppm)

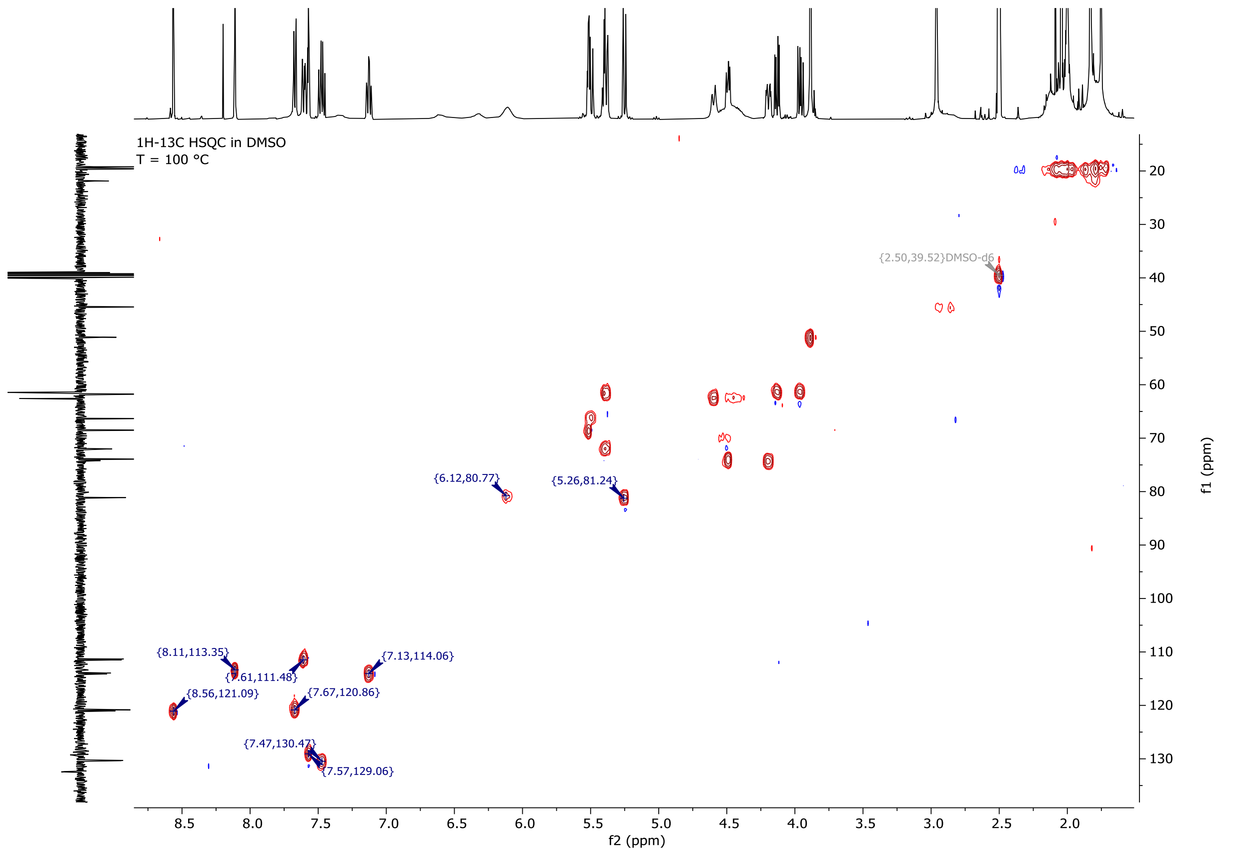
1H-13C HSQC in DMSO
T = 100 °C
{2.50,39.52}DMSO-d6
{6.12,80.77}
{5.26,81.24}
{8.11,113.35}
{7.61,111.48}
{7.13,114.06}
{8.56,121.09}
{7.67,120.86}
{7.47,130.47}
{7.57,129.06}
f2 (ppm)
f1 (ppm)

^{1}H, ^{13}C APT and ^{19}F NMR spectra of (10)

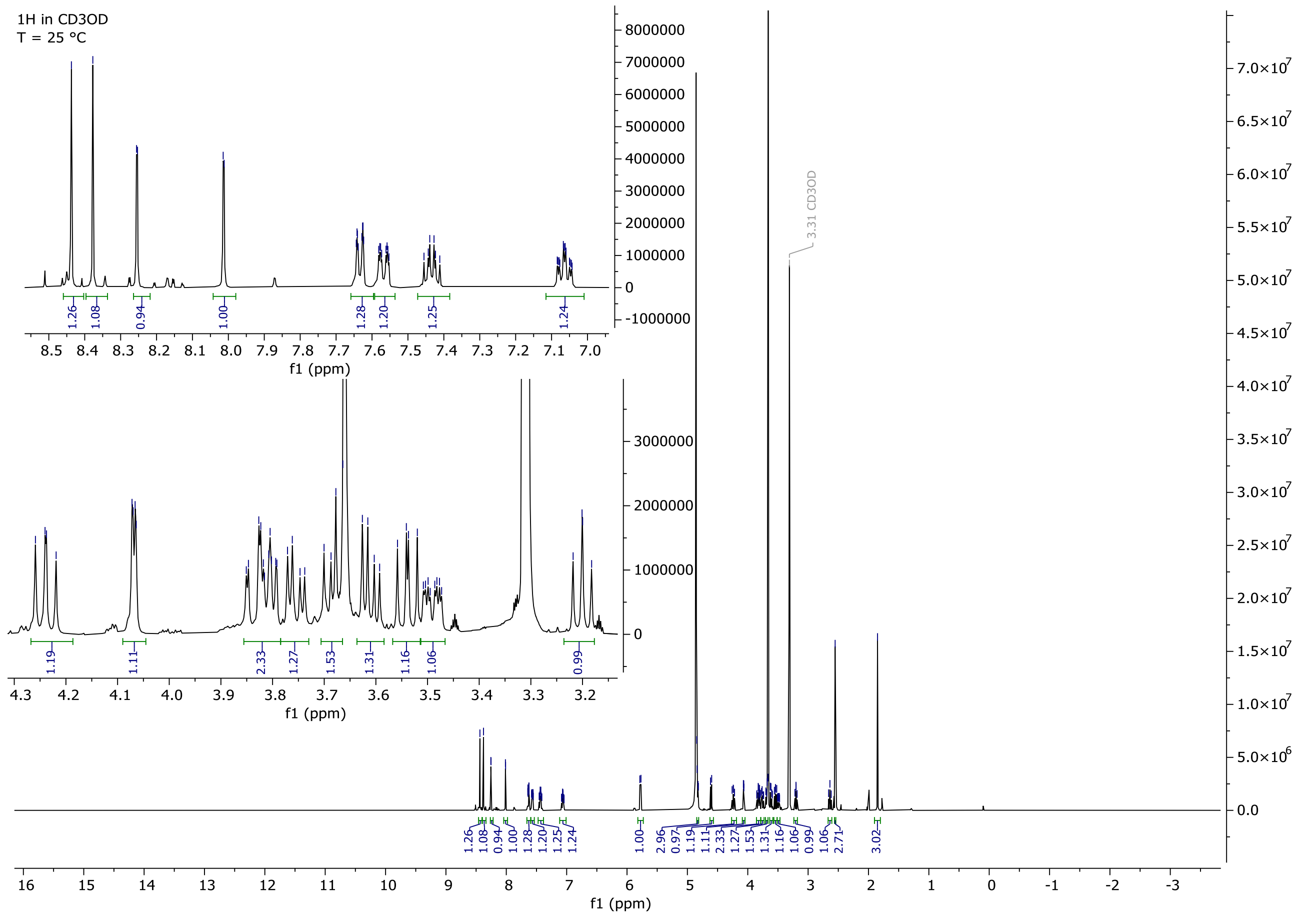

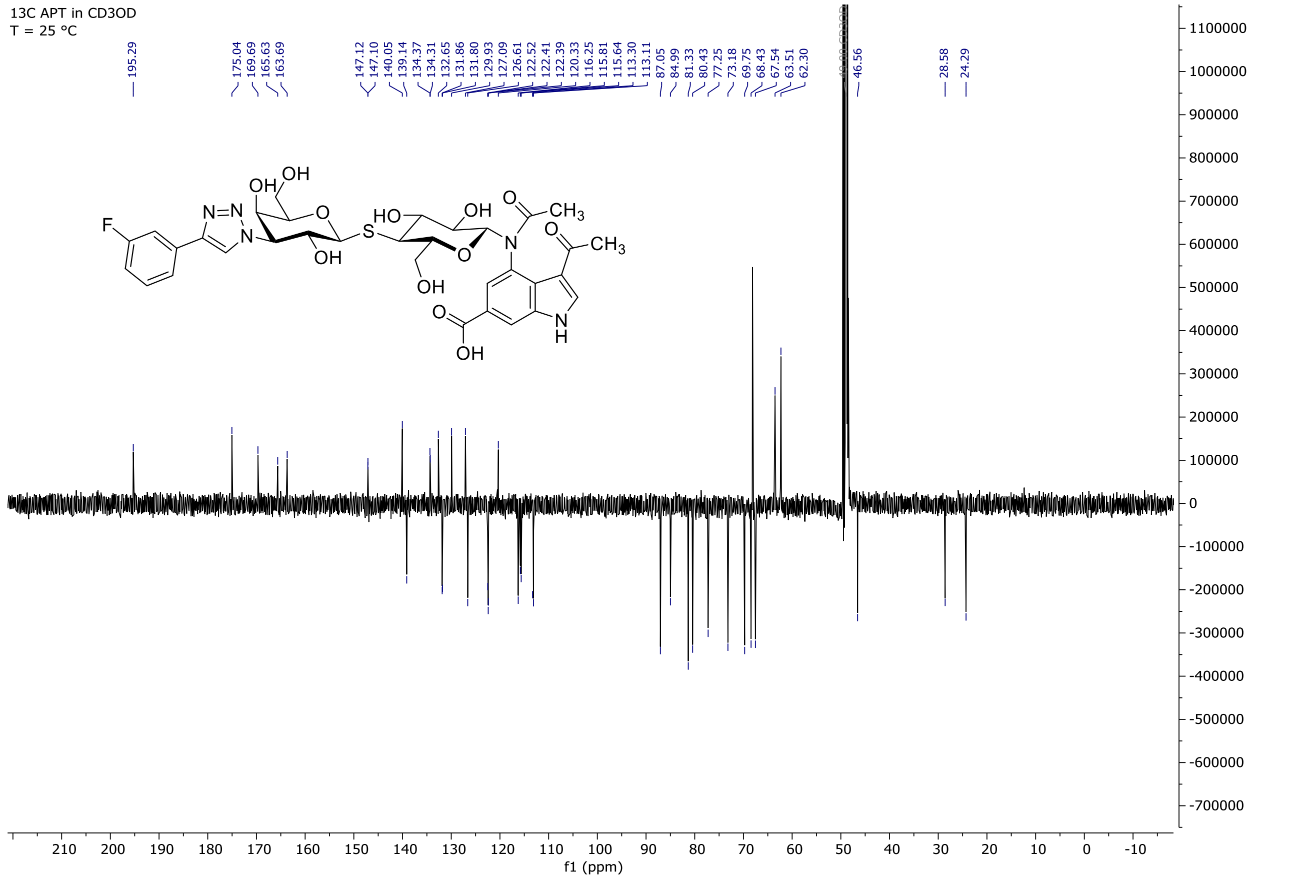

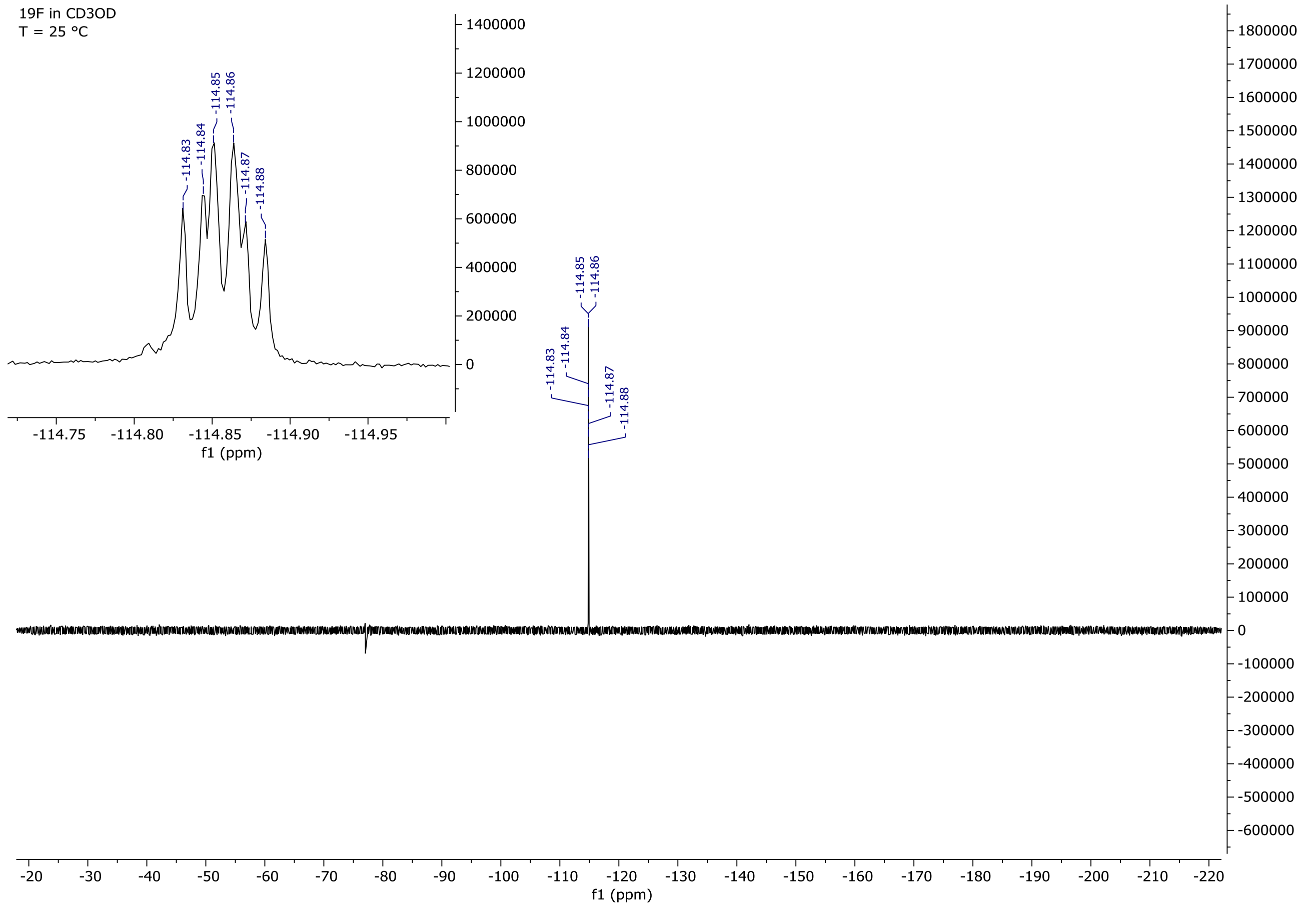
19F in CD3OD
T = 25 °C
-114.83
-114.84
-114.85
-114.86
-114.87
-114.88
f1 (ppm)

^{1}H, ^{13}C APT, ^{19}F NMR and HSQC spectra of (11)

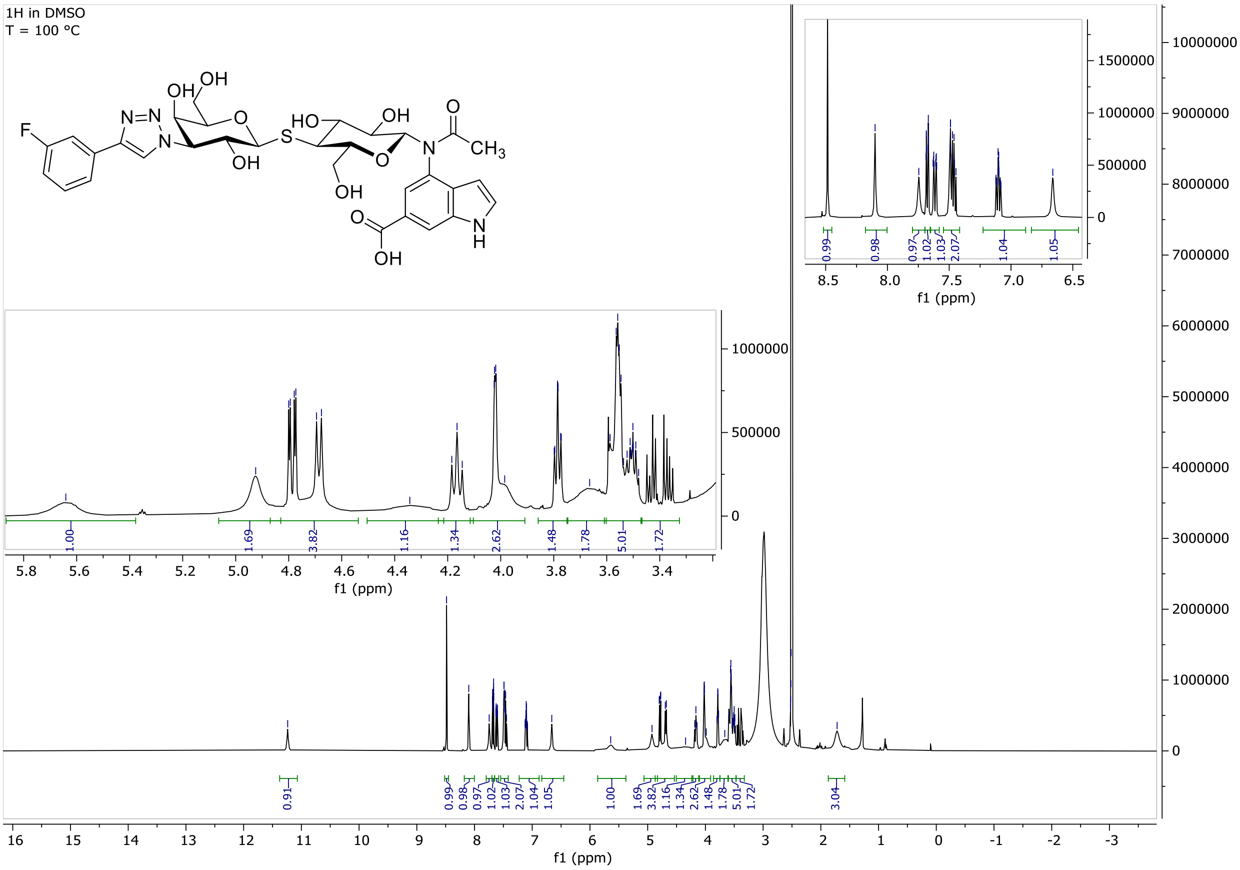

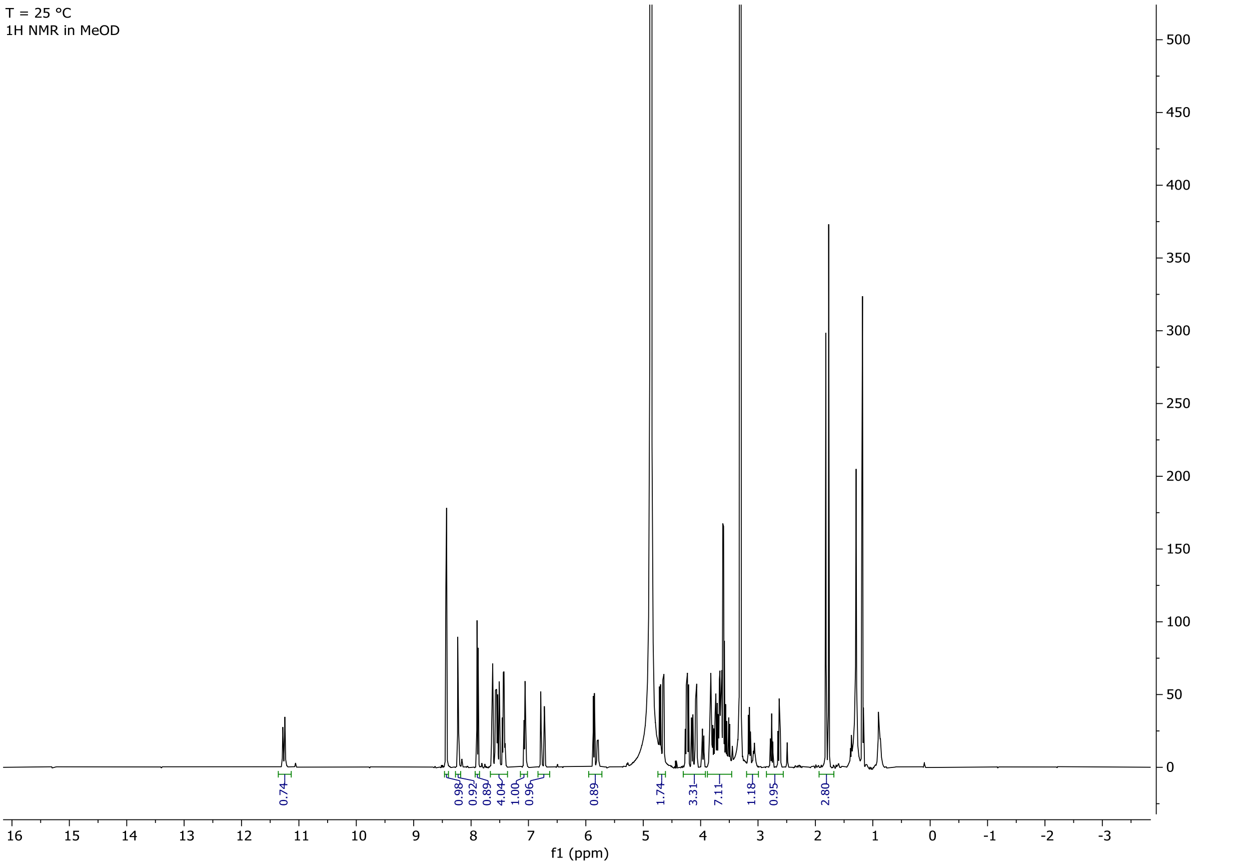

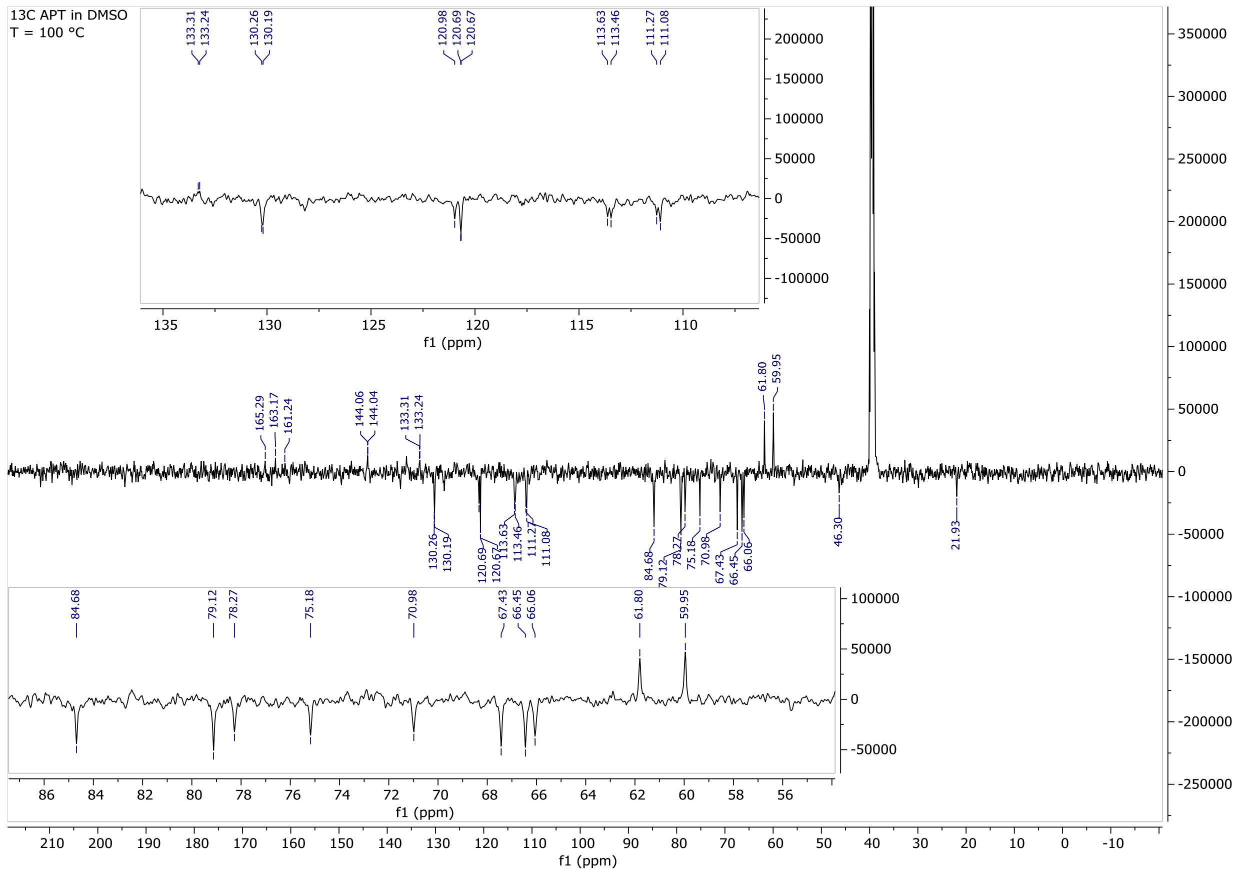
13C APT in DMSO
T = 100 °C
f1 (ppm)

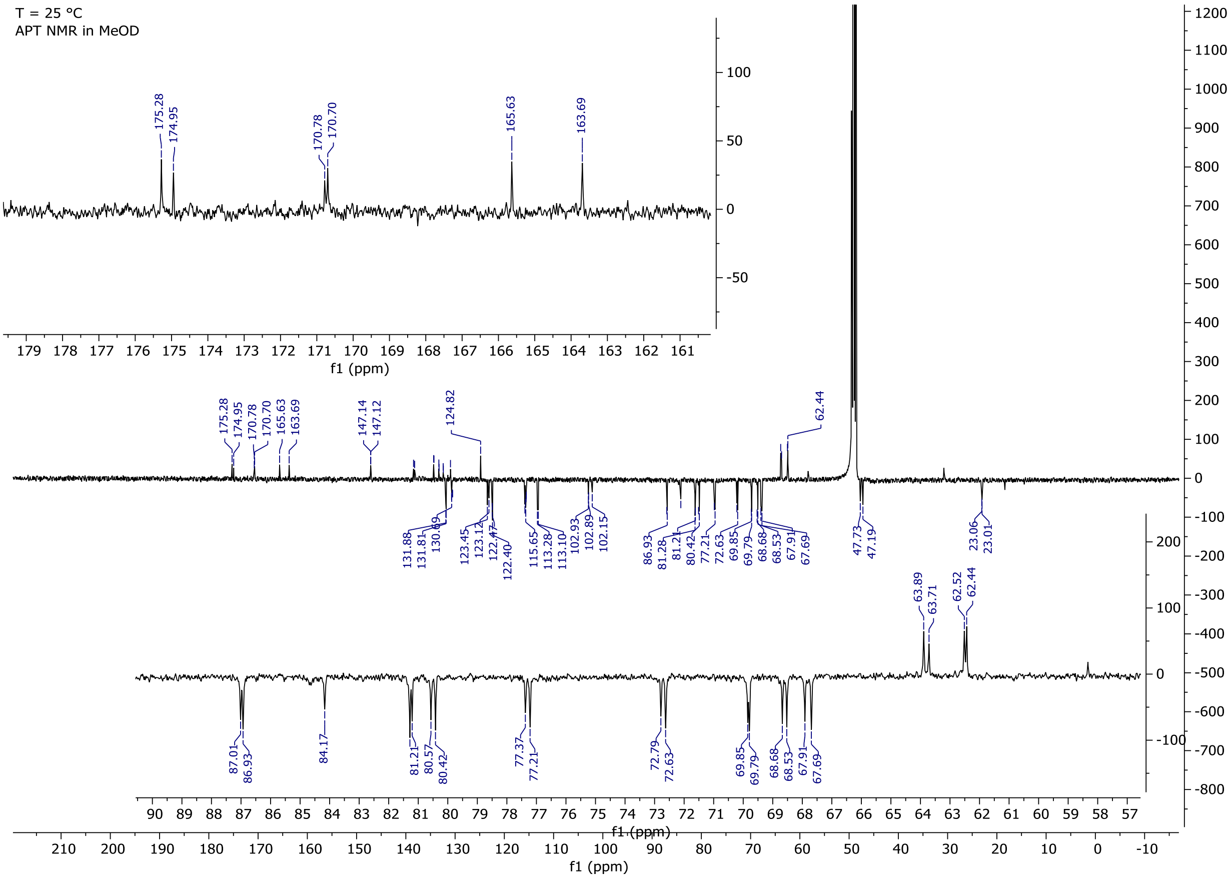
T = 25 °C
APT NMR in MeOD
f1 (ppm)

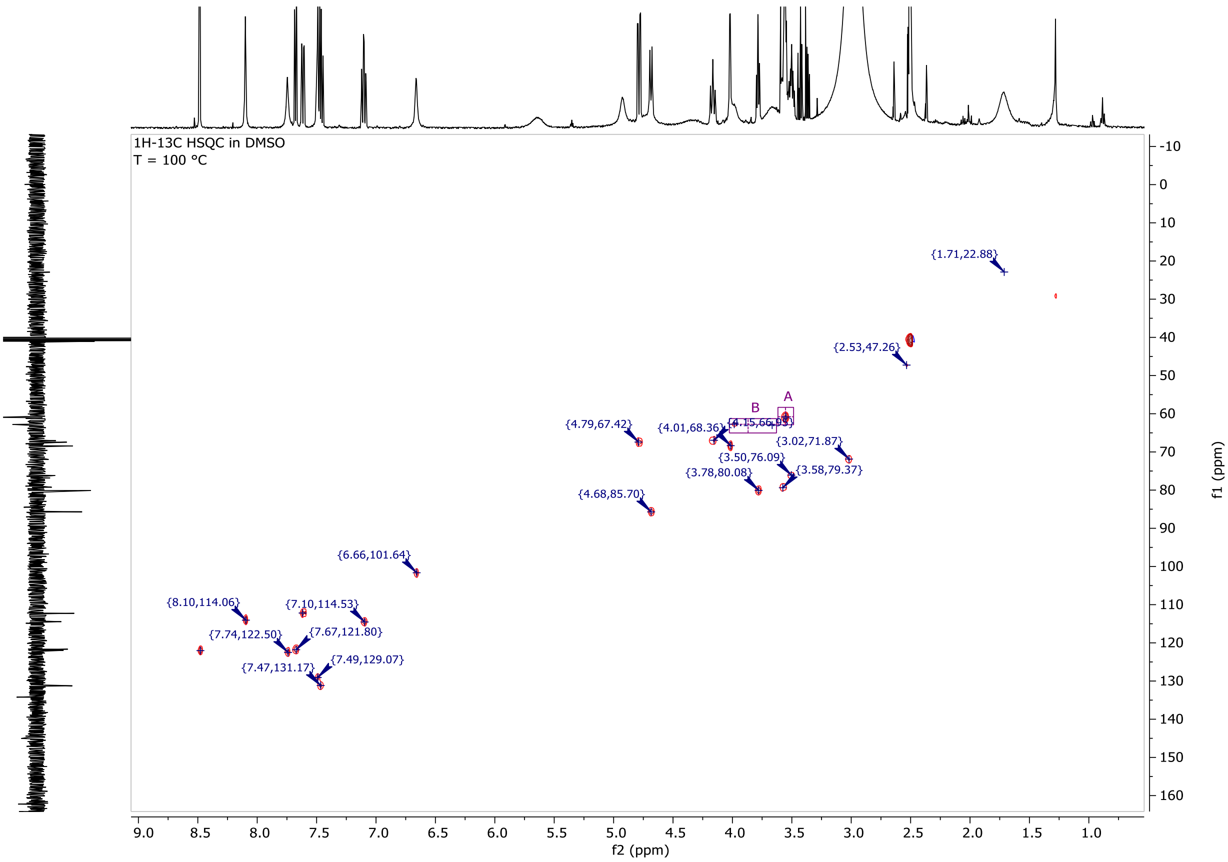
1H-13C HSQC in DMSO
T = 100 °C
{1.71,22.88}
{2.53,47.26}
A
B
{4.79,67.42}
{4.01,68.36}
{3.02,71.87}
{3.50,76.09}
{3.78,80.08}
{3.58,79.37}
{4.68,85.70}
{6.66,101.64}
{8.10,114.06}
{7.10,114.53}
{7.74,122.50}
{7.67,121.80}
{7.49,129.07}
{7.47,131.17}
f2 (ppm)
f1 (ppm)

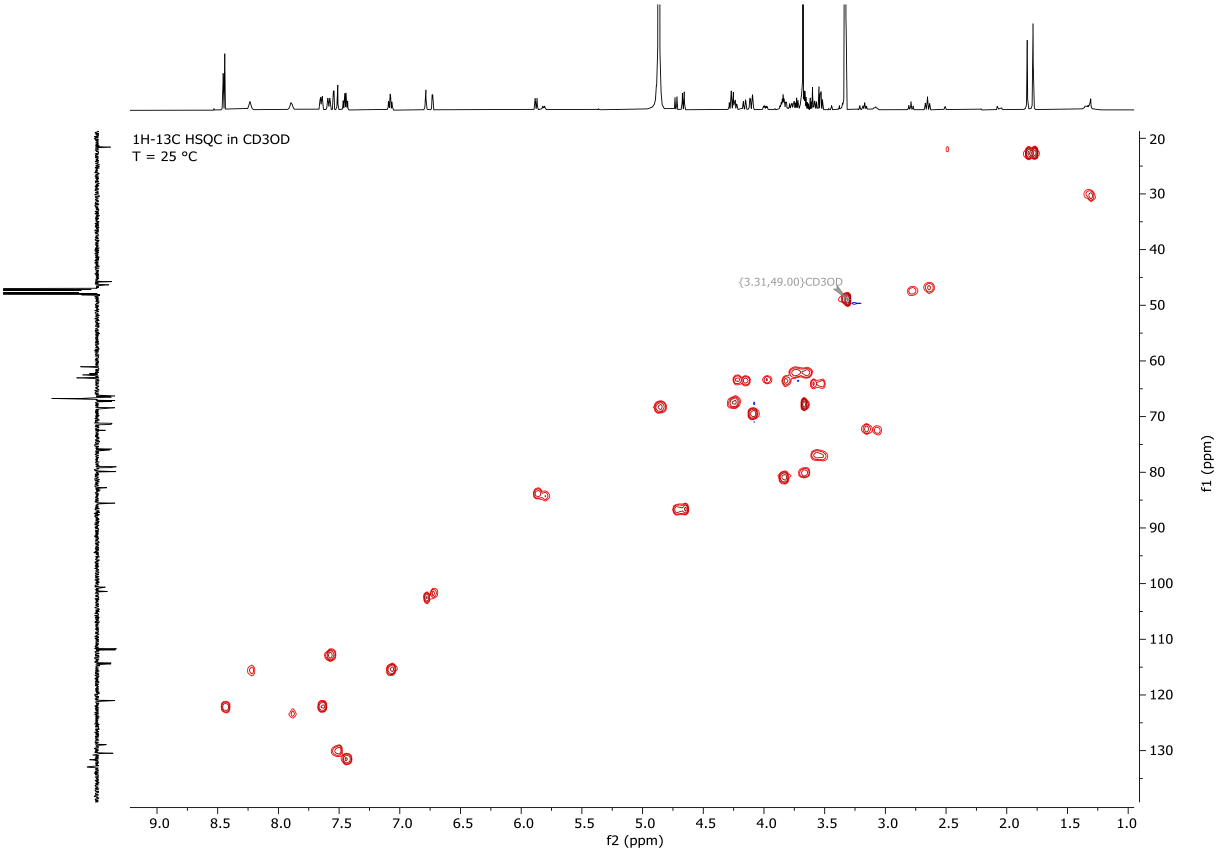
1H-13C HSQC in CD3OD
T = 25 °C
{3.31,49.00}CD3OD
f2 (ppm)
f1 (ppm)

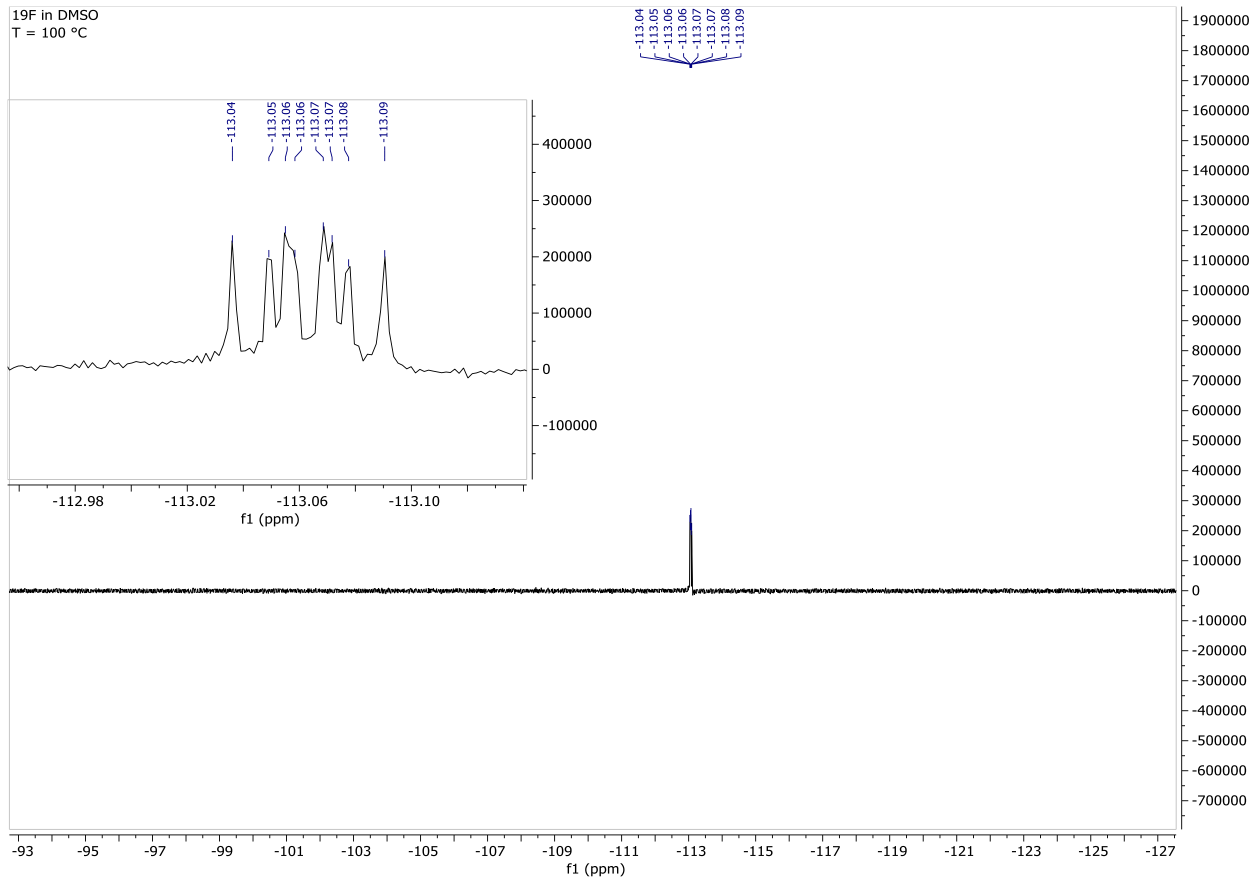
19F in DMSO
T = 100 °C
-113.04
-113.05
-113.06
-113.06
-113.07
-113.07
-113.08
-113.09
f1 (ppm)

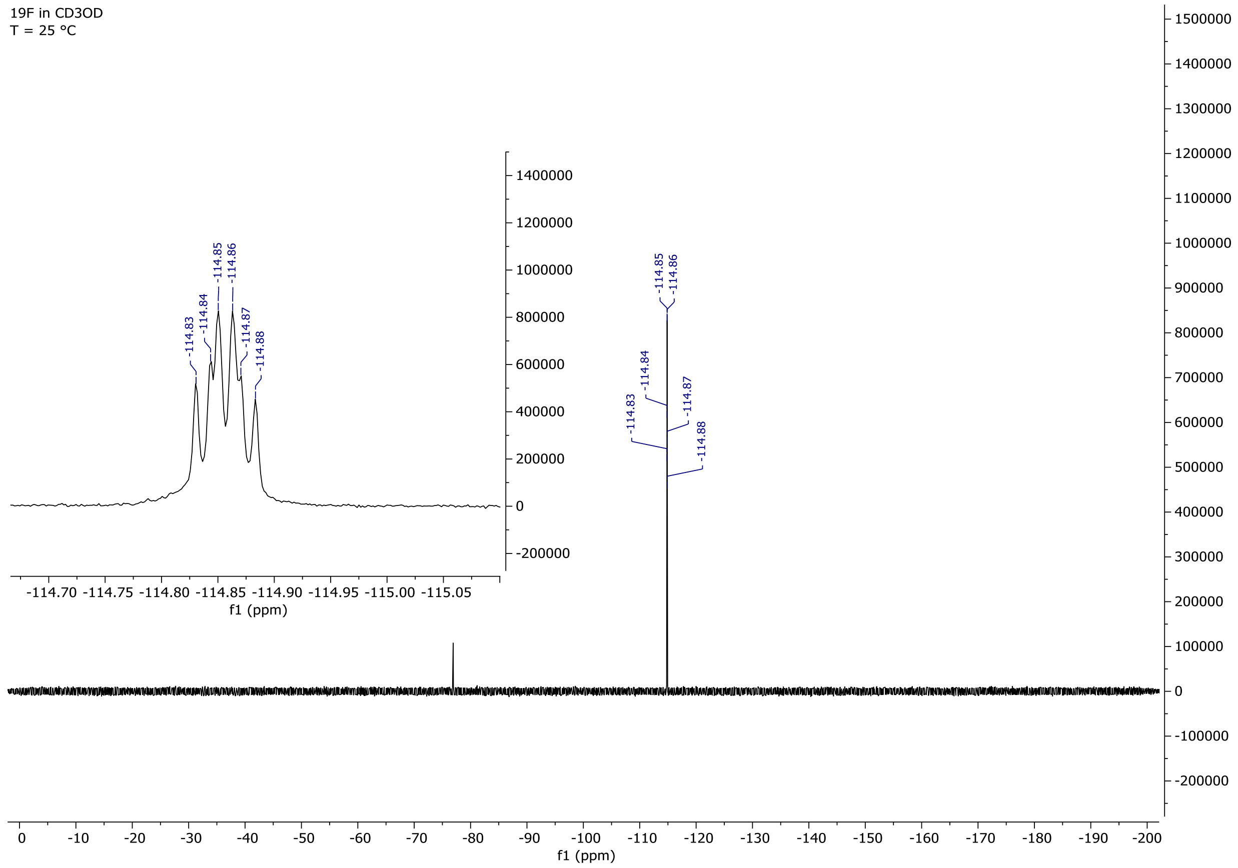
19F in CD3OD
T = 25 °C
-114.83
-114.84
-114.85
-114.86
-114.87
-114.88
f1 (ppm)

Representative HPLC traces for key investigated compounds.

*N-[4-O-(β-D-Galactopyranosyl)-β-D-glucopyranosyl]-N-(3-carboxyphenyl)acetamide (**3r**)*

Sample Name:	JZ6-92H	Acquired By:	System
Sample Type:	Unknown	Date Acquired:	7/10/2024 10:01:36 AM CET
Vial:	46	Acq. Method Set:	10to90MeOHin25m
Injection #:	1	Date Processed:	1/23/2025 1:16:30 PM CET
Injection Volume:	10.00 ul	Processing Method:	JZ_processing
Run Time:	25.0 Minutes	Channel Name:	224.0nm
Sample Set Name:	240710_JZ_6samplesTest	Proc. Chnl. Descr.:	PDA 224.0 nm

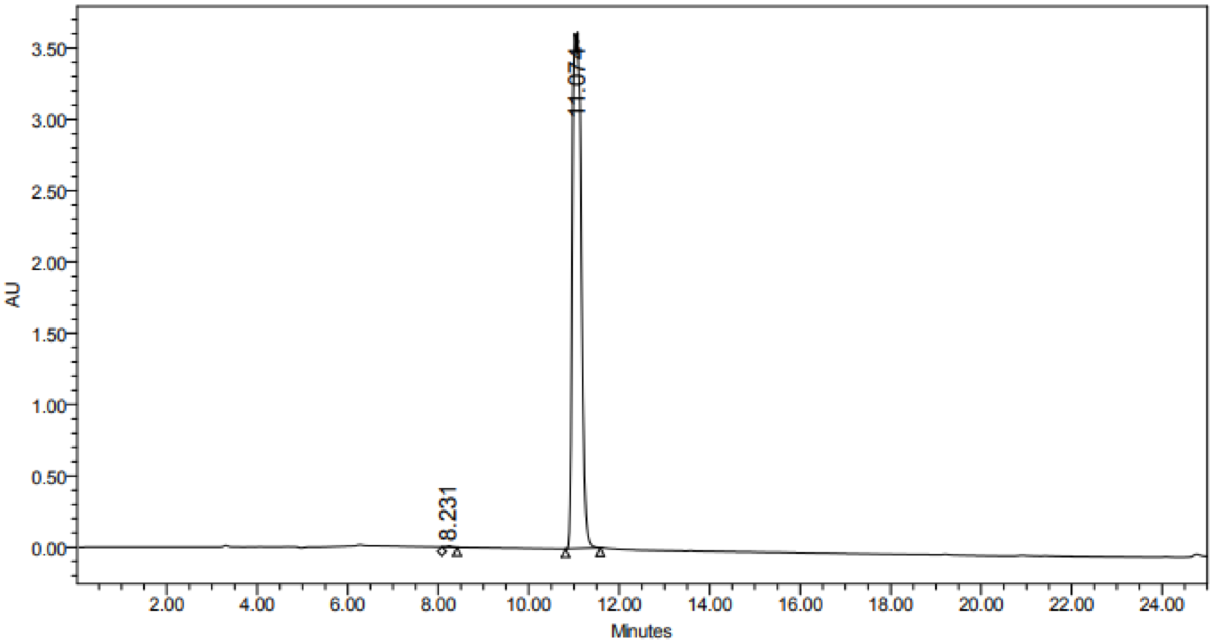

	RT	Area	% Area	Height
1	8.231	64163	0.13	7601
2	11.074	49259429	99.87	3563260

*N-[4-O-(β-D-Galactopyranosyl)-β-D-glucopyranosyl]-N-(3-carboxy-5-hydroxyphenyl)acetamide (**3ad**)*

Sample Name:	JZ6-92C	Acquired By:	System
Sample Type:	Unknown	Date Acquired:	7/11/2024 12:05:05 PM CET
Vial:	55	Acq. Method Set:	10to90MeOHin25m
Injection #:	1	Date Processed:	1/23/2025 1:01:08 PM CET
Injection Volume:	10.00 ul	Processing Method:	JZ_processing
Run Time:	25.0 Minutes	Channel Name:	224.0nm
Sample Set Name:	240711_JZ_dalsi6	Proc. Chnl. Descr.:	PDA 224.0 nm

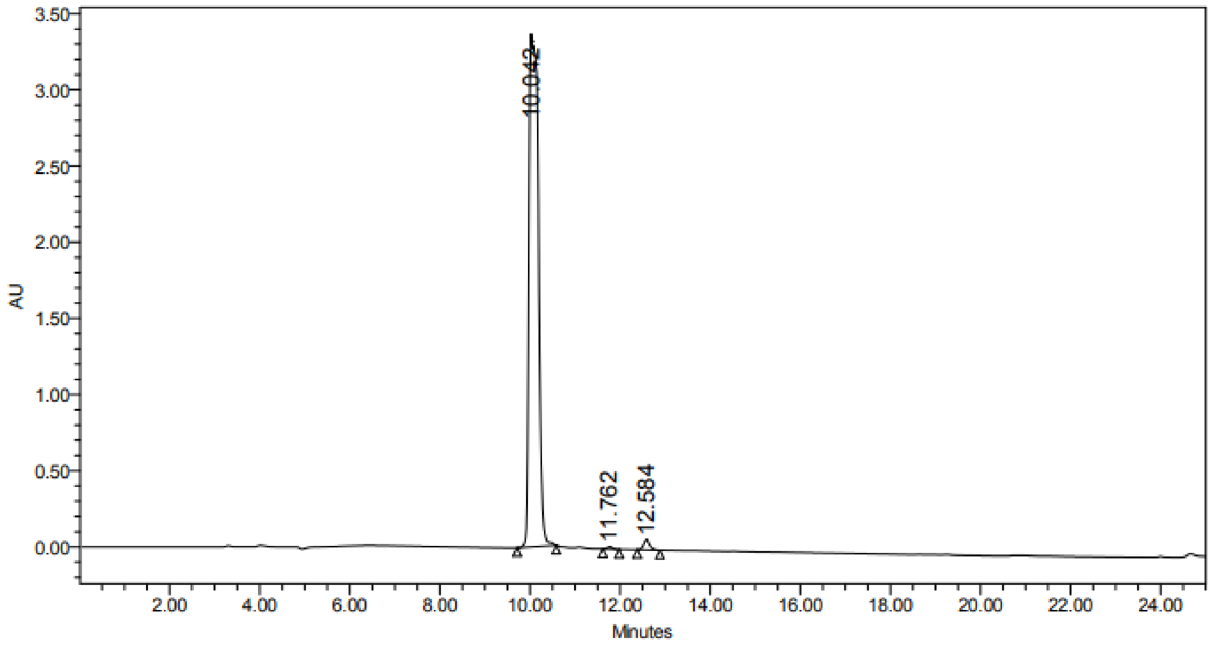

	RT	Area	% Area	Height
1	10.042	47801629	98.47	3328766
2	11.762	122382	0.25	14388
3	12.584	621472	1.28	65156

*N-[4-O-(β-D-Galactopyranosyl)-β-D-glucopyranosyl]-N-[6-(methylcarboxy)-1H-indol-4-yl]acetamide (**3ae**)*

Sample Name:	JZ7-23L	Acquired By:	System
Sample Type:	Unknown	Date Acquired:	7/11/2024 10:13:23 AM CET
Vial:	53	Acq. Method Set:	10to90MeOHin25m
Injection #:	1	Date Processed:	1/23/2025 1:02:13 PM CET
Injection Volume:	10.00 ul	Processing Method:	JZ_processing
Run Time:	25.0 Minutes	Channel Name:	224.0nm
Sample Set Name:	240711_JZ_dalsi3	Proc. Chnl. Descr.:	PDA 224.0 nm

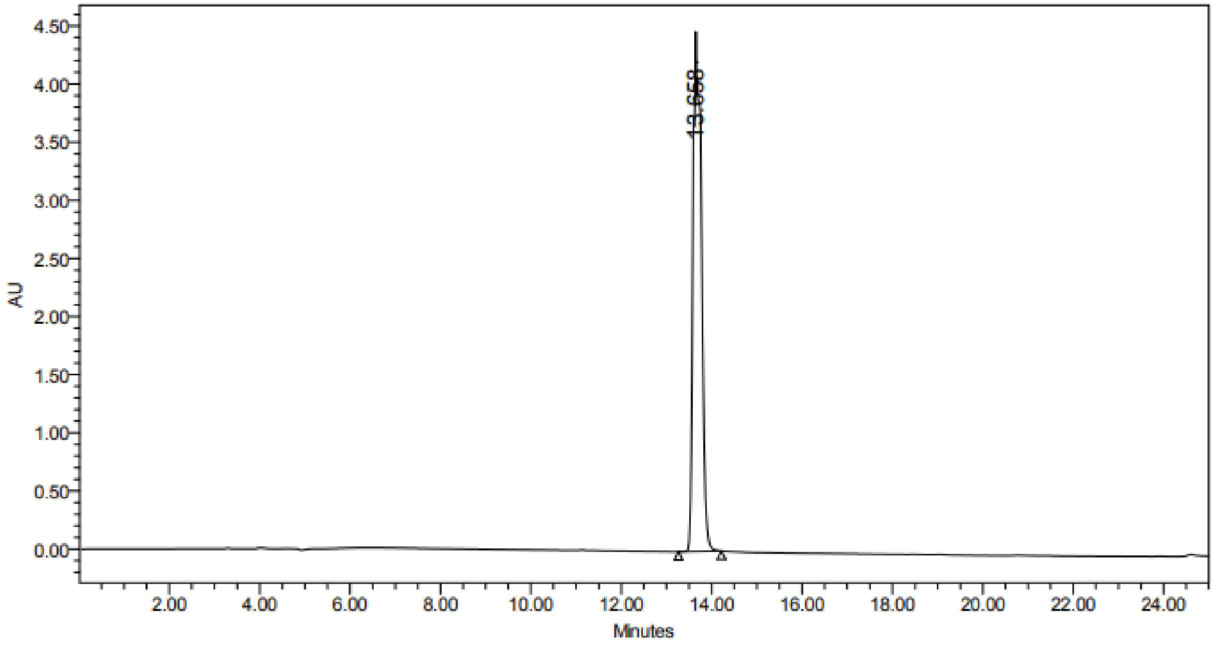

	RT	Area	% Area	Height
1	13.658	57030768	100.00	4205396

*N-[4-O-(β-D-Galactopyranosyl)-β-D-glucopyranosyl]-N-(6-carboxy-1H-indol-4-yl)acetamide (**3af**)*

Sample Name:	JZ7-25L	Acquired By:	System
Sample Type:	Unknown	Date Acquired:	7/10/2024 8:58:50 AM CET
Vial:	45	Acq. Method Set:	10to90MeOHin25m
Injection #:	1	Date Processed:	1/23/2025 1:17:15 PM CET
Injection Volume:	10.00 ul	Processing Method:	JZ_processing
Run Time:	25.0 Minutes	Channel Name:	224.0nm
Sample Set Name:	240710_JZ_6samplesTest	Proc. Chnl. Descr.:	PDA 224.0 nm

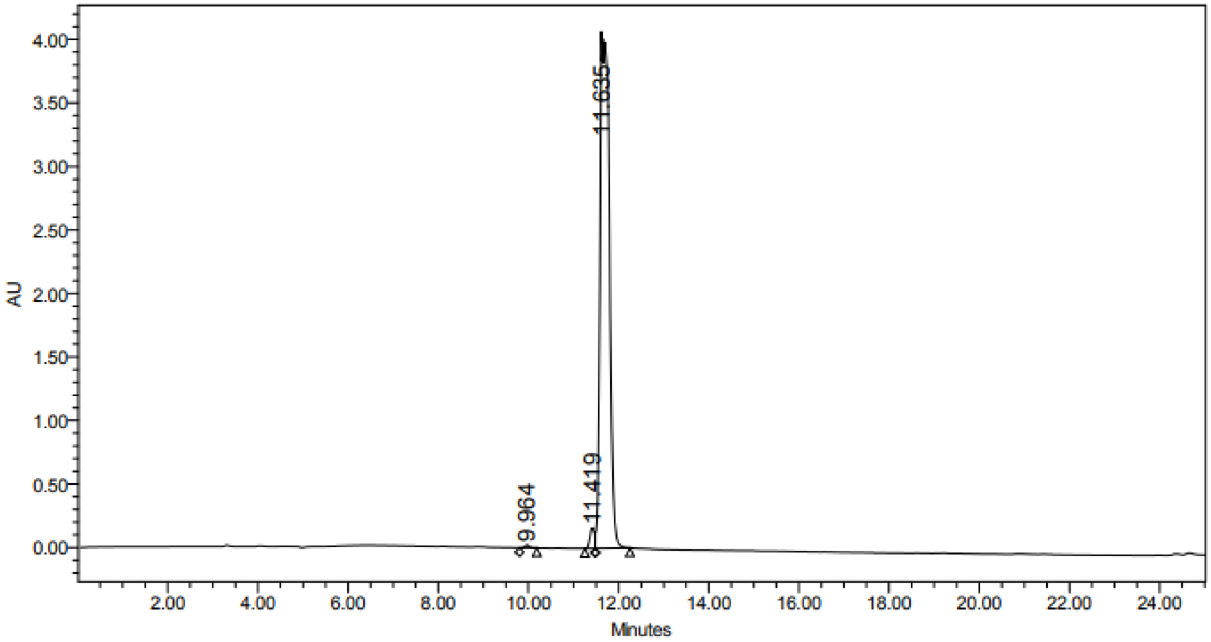

	RT	Area	% Area	Height
1	9.964	160416	0.27	18551
2	11.419	1110366	1.84	158908
3	11.635	58946657	97.89	4085755

N-[4-*S*-(3-Deoxy-3-(4-(3-fluorophenyl)-1*H*-1,2,3-triazol-1-yl)-β-D-galactopyranosyl)-4-thio-β-D-glucopyranosyl]-*N*-(3-acetyl-6-carboxy-1*H*-indol-4-yl)acetamide (**10**)

Sample Name:	JZ7-47-4	Acquired By:	System
Sample Type:	Unknown	Date Acquired:	7/16/2024 12:14:40 PM CET
Vial:	72	Acq. Method Set:	10to90MeOHin25m
Injection #:	1	Date Processed:	1/23/2025 1:22:55 PM CET
Injection Volume:	10.00 ul	Processing Method:	JZ_processing
Run Time:	25.0 Minutes	Channel Name:	224.0nm
Sample Set Name:	240710_JZ_doubleFlap2	Proc. Chnl. Descr.:	PDA 224.0 nm

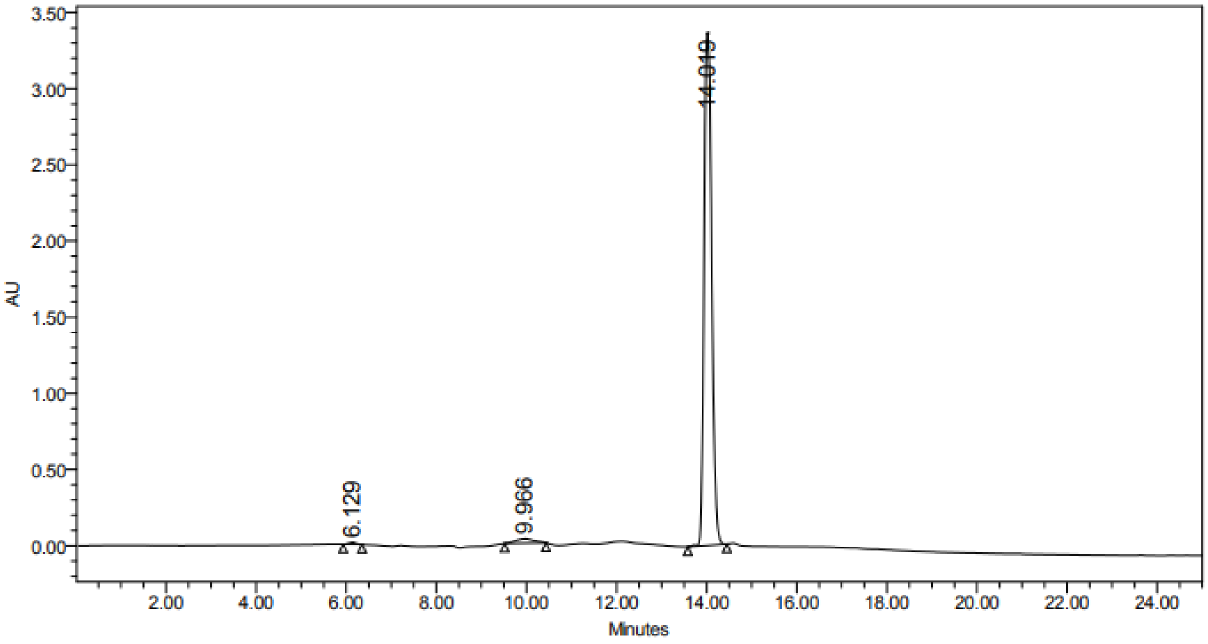

	RT	Area	% Area	Height
1	6.129	129968	0.32	11983
2	9.966	842863	2.09	28964
3	14.019	39268306	97.58	3371207

*N-[4-S-(3-Deoxy-3-(4-(3-fluorophenyl)-1H-1,2,3-triazol-1-yl)-β-D-galactopyranosyl)-4-thio-β-D-glucopyranosyl]-N-(6-carboxy-1H-indol-4-yl)acetamide (**11**)*

Sample Name:	JZ7-50-2	Acquired By:	System
Sample Type:	Unknown	Date Acquired:	7/16/2024 12:51:48 PM CET
Vial:	48	Acq. Method Set:	10to90MeOHin25m
Injection #:	1	Date Processed:	1/23/2025 1:21:51 PM CET
Injection Volume:	10.00 ul	Processing Method:	JZ_processing
Run Time:	25.0 Minutes	Channel Name:	224.0nm
Sample Set Name:	240710_JZ_doubleFlap2	Proc. Chnl. Descr.:	PDA 224.0 nm

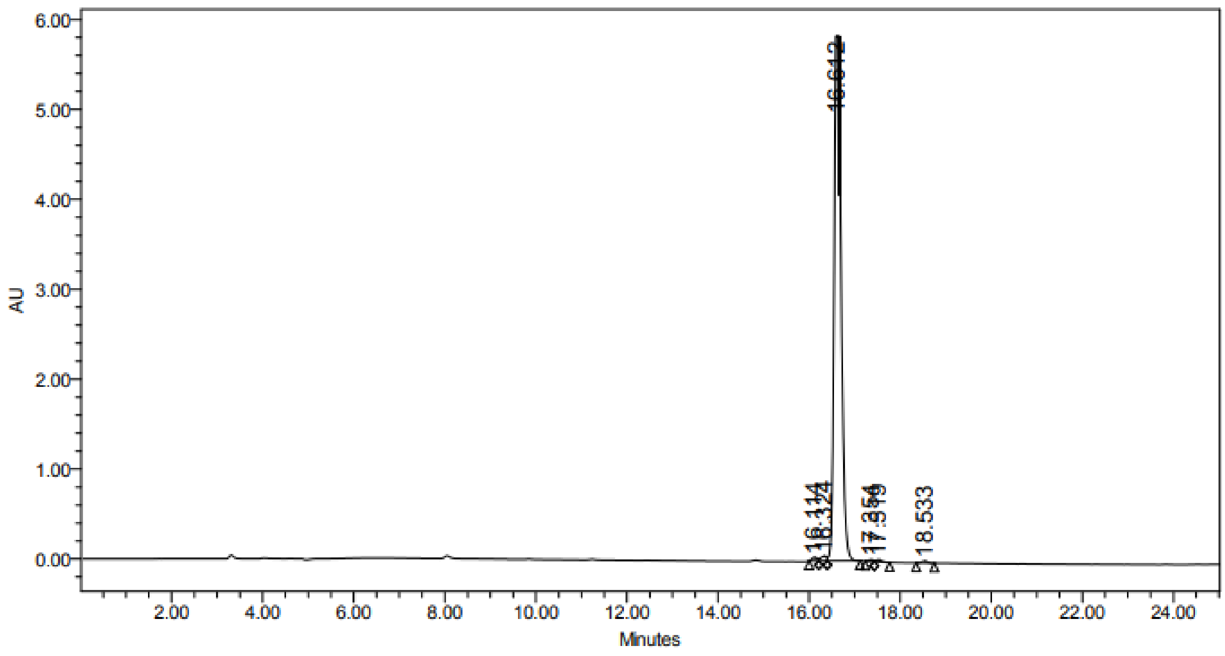

	RT	Area	% Area	Height
1	16.114	311275	0.49	36814
2	16.324	436720	0.69	51564
3	16.612	62462988	98.02	6027909
4	17.354	129369	0.20	16281
5	17.519	209090	0.33	19966
6	18.533	173555	0.27	19848

References

(1) Peterson, K.; Kumar, R.; Stenström, O.; Verma, P.; Verma, P. R.; Håkansson, M.; Kahl-Knutsson, B.; Zetterberg, F.; Leffler, H.; Akke, M.; et al. Systematic Tuning of Fluoro-galectin-3 Interactions Provides Thiodigalactoside Derivatives with Single-Digit nM Affinity and High Selectivity. *J. Med. Chem.* **2018**, *61* (3), 1164-1175.
(2) Hamala, V.; Kurfiřt, M.; Červenková Šťastná, L.; Hujerová, H.; Bernášková, J.; Parkan, K.; Kaminský, J.; Habanová, N.; Kozák, J.; Magdolenová, A.; et al. Ferrocene- and ruthenium arene-containing glycomimetics as selective inhibitors of human galectin-1 and -3. *Inorganic Chemistry Frontiers* **2024**, *11* (21), 7588-7609.
(3) Wang, Z.-X. An exact mathematical expression for describing competitive binding of two different ligands to a protein molecule. *FEBS Lett.* **1995**, *360* (2), 111-114.
(4) Diederichs, K.; Karplus, P. A. Improved R-factors for diffraction data analysis in macromolecular crystallography. *Nat. Struct. Biol.* **1997**, *4* (4), 269-275.
(5) Agirre, J.; Atanasova, M.; Bagdonas, H.; Ballard, C. B.; Basle, A.; Beilsten-Edmands, J.; Borges, R. J.; Brown, D. G.; Burgos-Marmol, J. J.; Berrisford, J. M.; et al. The CCP4 suite: integrative software for macromolecular crystallography. *Acta Crystallographica Section D* **2023**, *79* (6), 449-461.
(6) Murshudov, G. N.; Skubak, P.; Lebedev, A. A.; Pannu, N. S.; Steiner, R. A.; Nicholls, R. A.; Winn, M. D.; Long, F.; Vagin, A. A. REFMAC5 for the refinement of macromolecular crystal structures. *Acta Crystallographica Section D* **2011**, *67* (4), 355-367.
(7) Huang, Y.; Shaw, M. A.; Mullins, E. S.; Kirley, T. L.; Ayres, N. Synthesis and Anticoagulant Activity of Polyureas Containing Sulfated Carbohydrates. *Biomacromolecules* **2014**, *15* (12), 4455-4466.
(8) Koguro, K.; Oga, T.; Mitsui, S.; Orita, R. Novel Synthesis of 5-Substituted Tetrazoles from Nitriles. *Synthesis* **1998**, *1998* (06), 910-914.
(9) Beladhria, A.; Beydoun, K.; Ammar, H. B.; Salem, R. B.; Doucet, H. Palladium-Catalysed Direct Arylation of Heteroaromatics Using Unprotected Iodoanilines with Inhibition of the Amination Reaction. *Synthesis* **2012**, *44* (14), 2264-2276.
(10) Kovalová, A.; Prouza, V.; Zavřel, M.; Hájek, M.; Dzijak, R.; Magdolenová, A.; Pohl, R.; Voburka, Z.; Parkan, K.; Vrabel, M. Selection of Galectin-Binding Ligands from Synthetic Glycopeptide Libraries. *ChemPlusChem* **2024**, *89* (7), e202300567.
